# Supplementary material for: Clinicopathological characteristics, treatment and survival of pulmonary large cell neuroendocrine carcinoma: a SEER population-based study
Source: PeerJ. 2019 Mar 27;7:e6539. doi: 10.7717/peerj.6539 (PMC6441320; doi:10.7717/peerj.6539)
Supplement: Supplemental Information 2 — International Classification of Diseases for Oncology (ICD-O) -03 site/histology validation list. [file peerj-07-6539-s002.pdf]

March 23, 2018

ICD-0-3 SEER SITE/HISTOLOGY VALIDATION LIST

This file is intended as a reference file for ICD-O-3 only and is not to be used for casefinding purposes. The ICD-O-3 site/type validation program was modified to allow only for the site/histology/behavior combinations listed in this publication. All other cases must be reviewed.

Submit any comments or questions about this document to:

Ask A SEER Registrar

https://seer.cancer.gov/registrars/contact.html

or

seerweb@imsweb.com

LIP C000-C006,C008-C009

NEOPLASM	800	8000/3	Neoplasm, malignant
		8001/3	Tumor cells, malignant
		8002/3	Malignant tumor, small cell type
		8003/3	Malignant tumor, giant cell type
		8004/3	Malignant tumor, spindle cell type
		8005/3	Malignant tumor, clear cell type
CARCINOMA, NOS	801	8010/2	Carcinoma in situ, NOS
		8010/3	Carcinoma, NOS
		8011/3	Epithelioma, malignant
		8012/3	Large cell carcinoma, NOS
		8013/3	Large cell neuroendocrine carcinoma
		8014/3	Large cell carcinoma with rhabdoid phenotype
		8015/3	Glassy cell carcinoma
CARCINOMA, UNDIFF., NOS	802	8020/3	Carcinoma, undifferentiated type, NOS
		8021/3	Carcinoma, anaplastic type, NOS
		8022/3	Pleomorphic carcinoma
GIANT & SPINDLE CELL CARCINOMA	803	8030/3	Giant cell and spindle cell carcinoma
		8031/3	Giant cell carcinoma
		8032/3	Spindle cell carcinoma
		8033/3	Pseudosarcomatous carcinoma
		8034/3	Polygonal cell carcinoma
		8035/3	Carcinoma with osteoclast-like giant cells
PAPILLARY CARCINOMA, NOS	805	8050/2	Papillary carcinoma in situ
		8050/3	Papillary carcinoma, NOS
		8051/3	Verrucous carcinoma, NOS
		8052/2	Papillary squamous cell carcinoma, non-invasive
		8052/3	Papillary squamous cell carcinoma
SQUAMOUS CELL CARCINOMA, NOS	807	8070/2	Squamous cell carcinoma in situ, NOS
		8070/3	Squamous cell carcinoma, NOS
		8071/3	Sq. cell carcinoma, keratinizing, NOS
		8072/3	Sq. cell carcinoma, lg. cell, non-ker.
		8073/3	Sq. cell carcinoma, sm. cell, non-ker.
		8074/3	Sq. cell carcinoma, spindle cell
		8075/3	Squamous cell carcinoma, adenoid
		8076/2	Sq. cell carc. in situ with question. stromal invas.
		8076/3	Sq. cell carcinoma, micro-invasive
		8078/3	Squamous cell carcinoma with horn formation

LIP C000-C006,C008-C009

LYMPHOEPITHELIAL CARCINOMA	808	8081/2	Bowen disease
		8082/3	Lymphoepithelial carcinoma
		8083/3	Basaloid squamous cell carcinoma
		8084/3	Squamous cell carcinoma, clear cell type
ADENOCARCINOMA, NOS	814	8140/2	Adenocarcinoma in situ
		8140/3	Adenocarcinoma, NOS
		8141/3	Scirrhous adenocarcinoma
		8143/3	Superficial spreading adenocarcinoma
		8147/3	Basal cell adenocarcinoma
ADENOID CYSTIC & CRIBRIFORM CA.	820	8200/3	Adenoid cystic carcinoma
		8201/2	Cribriform carcinoma in situ
		8201/3	Cribriform carcinoma
BRONCHIOLO-ALVEOLAR ADENOCA.	825	8255/3	Adenocarcinoma with mixed subtypes
PAPILLARY ADENOCARCINOMA, NOS	826	8260/3	Papillary adenocarcinoma, NOS
		8261/2	Adenocarcinoma in situ in villous adenoma
		8261/3	Adenocarcinoma in villous adenoma
		8262/3	Villous adenocarcinoma
		8263/2	Adenocarcinoma in situ in tubulovillous adenoma
		8263/3	Adenocarcinoma in tubulovillous adenoma
MUCOEPIDERMOID CARCINOMA	843	8430/3	Mucoepidermoid carcinoma
MUCINOUS ADENOCARCINOMA	848	8480/3	Mucinous adenocarcinoma
		8481/3	Mucin-producing adenocarcinoma
NEVI & MELANOMAS	872	8720/2	Melanoma in situ
		8720/3	Malignant melanoma, NOS
		8721/3	Nodular melanoma
		8722/3	Balloon cell melanoma
		8723/3	Malignant melanoma, regressing
AMELANOTIC MELANOMA	873	8730/3	Amelanotic melanoma
MAL. MEL. IN JUNCT. NEVUS	874	8743/3	Superficial spreading melanoma
		8745/3	Desmoplastic melanoma, malignant
		8746/3	Mucosal lentiginous melanoma
EPITHELIOID CELL MELANOMA	877	8770/3	Mixed epithel. & spindle cell melanoma
		8771/3	Epithelioid cell melanoma
		8772/3	Spindle cell melanoma, NOS

LIP C000-C006,C008-C009

MIXED TUMOR, MALIGNANT, NOS	894	8940/3	Mixed tumor, malignant, NOS
		8941/3	Carcinoma in pleomorphic adenoma
KAPOSI SARCOMA	914	9140/3	Kaposi sarcoma
FOLLIC. & MARGINAL LYMPH, NOS	969	9699/3	Marginal zone B-cell lymphoma, NOS
LYMPHOID LEUKEMIA, NOS	982	9823/3	Chronic lymphocytic leukemia/small lymphocytic lymphoma

BASE OF TONGUE C019

NEOPLASM	800	8000/3	Neoplasm, malignant
		8001/3	Tumor cells, malignant
		8002/3	Malignant tumor, small cell type
		8003/3	Malignant tumor, giant cell type
		8004/3	Malignant tumor, spindle cell type
		8005/3	Malignant tumor, clear cell type
CARCINOMA, NOS	801	8010/2	Carcinoma in situ, NOS
		8010/3	Carcinoma, NOS
		8011/3	Epithelioma, malignant
		8012/3	Large cell carcinoma, NOS
		8013/3	Large cell neuroendocrine carcinoma
		8014/3	Large cell carcinoma with rhabdoid phenotype
		8015/3	Glassy cell carcinoma
CARCINOMA, UNDIFF., NOS	802	8020/3	Carcinoma, undifferentiated type, NOS
		8021/3	Carcinoma, anaplastic type, NOS
		8022/3	Pleomorphic carcinoma
GIANT & SPINDLE CELL CARCINOMA	803	8030/3	Giant cell and spindle cell carcinoma
		8031/3	Giant cell carcinoma
		8032/3	Spindle cell carcinoma
		8033/3	Pseudosarcomatous carcinoma
		8034/3	Polygonal cell carcinoma
		8035/3	Carcinoma with osteoclast-like giant cells
PAPILLARY CARCINOMA, NOS	805	8050/2	Papillary carcinoma in situ
		8050/3	Papillary carcinoma, NOS
		8051/3	Verrucous carcinoma, NOS
		8052/2	Papillary squamous cell carcinoma, non-invasive
		8052/3	Papillary squamous cell carcinoma
SQUAMOUS CELL CARCINOMA, NOS	807	8070/2	Squamous cell carcinoma in situ, NOS
		8070/3	Squamous cell carcinoma, NOS
		8071/3	Sq. cell carcinoma, keratinizing, NOS
		8072/3	Sq. cell carcinoma, lg. cell, non-ker.
		8073/3	Sq. cell carcinoma, sm. cell, non-ker.
		8074/3	Sq. cell carcinoma, spindle cell
		8075/3	Squamous cell carcinoma, adenoid
		8076/2	Sq. cell carc. in situ with question. stromal invas.
		8076/3	Sq. cell carcinoma, micro-invasive
		8078/3	Squamous cell carcinoma with horn formation

BASE OF TONGUE C019

LYMPHOEPITHELIAL CARCINOMA	808	8081/2	Bowen disease
		8082/3	Lymphoepithelial carcinoma
		8083/3	Basaloid squamous cell carcinoma
		8084/3	Squamous cell carcinoma, clear cell type
		8085/3	Squamous cell carcinoma, HPV-positive
		8086/3	Squamous cell carcinoma, HPV-negative
ADENOCARCINOMA, NOS	814	8140/2	Adenocarcinoma in situ
		8140/3	Adenocarcinoma, NOS
		8141/3	Scirrhous adenocarcinoma
		8143/3	Superficial spreading adenocarcinoma
		8147/3	Basal cell adenocarcinoma
ADENOID CYSTIC & CRIBRIFORM CA.	820	8200/3	Adenoid cystic carcinoma
		8201/2	Cribriform carcinoma in situ
		8201/3	Cribriform carcinoma
BRONCHIOLO-ALVEOLAR ADENOCA.	825	8255/3	Adenocarcinoma with mixed subtypes
MUCOEPIDERMOID CARCINOMA	843	8430/3	Mucoepidermoid carcinoma
MUCINOUS ADENOCARCINOMA	848	8480/3	Mucinous adenocarcinoma
		8481/3	Mucin-producing adenocarcinoma
ADENOSQUAMOUS CARCINOMA	856	8560/3	Adenosquamous carcinoma
		8562/3	Epithelial-myoepithelial carcinoma
ADENOCA. WITH METAPLASIA	857	8570/3	Adenocarcinoma with squamous metaplasia
		8571/3	Adenocarcinoma w cartilag. & oss. metaplas.
		8572/3	Adenocarcinoma with spindle cell mataplasia
		8573/3	Adenocarcinoma with apocrine metaplasia
		8574/3	Adenocarcinoma with neuroendocrine differen.
		8575/3	Metaplastic carcinoma, NOS
NEVI & MELANOMAS	872	8720/2	Melanoma in situ
		8720/3	Malignant melanoma, NOS
		8721/3	Nodular melanoma
		8722/3	Balloon cell melanoma
		8723/3	Malignant melanoma, regressing
SARCOMA, NOS	882	8825/3	Myofibroblastic sarcoma
RHABDOMYOSARCOMA, NOS	890	8900/3	Rhabdomyosarcoma, NOS
		8901/3	Pleomorphic rhabdomyosarcoma, adult type
		8902/3	Mixed type rhabdomyosarcoma

BASE OF TONGUE C019

EMBRYONAL RHABDOMYOSARCOMA	891	8910/3	Embryonal rhabdomyosarcoma
		8912/3	Spindle cell rhabdomyosarcoma
MIXED TUMOR, MALIGNANT, NOS	894	8940/3	Mixed tumor, malignant, NOS
		8941/3	Carcinoma in pleomorphic adenoma
KAPOSI SARCOMA	914	9140/3	Kaposi sarcoma
MALIGNANT LYMPHOMA, NOS	959	9590/3	Malignant lymphoma, NOS
		9591/3	Malignant lymphoma, non-Hodgkin
		9596/3	Composite Hodgkin and non-Hodgkin lymphoma
HODGKIN LYMPHOMA	965	9650/3	Hodgkin lymphoma, NOS
		9651/3	Hodgkin lymphoma, lymphocyte-rich
		9652/3	Hodgkin lymphoma, mixed cellularity, NOS
		9653/3	Hodgkin lymphoma, lymphocytic deplet., NOS
		9654/3	Hodgkin lymph., lymphocyt. deplet., diffuse fibrosis
		9655/3	Hodgkin lymphoma, lymphocyt. deplet., reticular
		9659/3	Hodgkin lymph., nodular lymphocyte predom.
HODGKIN LYMPHOMA, NOD. SCLER.	966	9661/3	Hodgkin granuloma [obs]
		9662/3	Hodgkin sarcoma [obs]
		9663/3	Hodgkin lymphoma, nodular sclerosis, NOS
		9664/3	Hodgkin lymphoma, nod. scler., cellular phase
		9665/3	Hodgkin lymphoma, nod. scler., grade 1
		9667/3	Hodgkin lymphoma, nod. scler., grade 2
ML, SMALL B-CELL LYMPHOCYTIC	967	9670/3	ML, small B lymphocytic, NOS
		9671/3	ML, lymphoplasmacytic
		9673/3	Mantle cell lymphoma
		9675/3	ML, mixed sm. and lg. cell, diffuse
ML, LARGE B-CELL, DIFFUSE	968	9680/3	ML, large B-cell, diffuse
		9684/3	ML, large B-cell, diffuse, immunoblastic, NOS
		9687/3	Burkitt lymphoma, NOS
		9688/3	T-cell histiocyte rich large B-cell lymphoma
FOLLIC. & MARGINAL LYMPH, NOS	969	9690/3	Follicular lymphoma, NOS
		9691/3	Follicular lymphoma, grade 2
		9695/3	Follicular lymphoma, grade 1
		9698/3	Follicular lymphoma, grade 3
		9699/3	Marginal zone B-cell lymphoma, NOS

BASE OF TONGUE C019

T-CELL LYMPHOMAS	970	9701/3	Sezary syndrome
		9702/3	Mature T-cell lymphoma, NOS
		9705/3	Angioimmunoblastic T-cell lymphoma
OTHER SPEC. NON-HODGKIN LYMPHOMA	971	9712/3	Intravascular large B-cell lymphoma
		9714/3	Anaplastic large cell lymphoma, T-cell and Null cell type
		9719/3	NK/T-cell lymphoma, nasal and nasal-type
PRECURS. CELL LYMPHOBLASTIC LYMPH.	972	9724/3	SystemicEBV pos. T-cell lymphoproliferative disease of childhood
		9727/3	Precursor cell lymphoblastic lymphoma, NOS
		9728/3	Precursor B-cell lymphoblastic lymphoma
		9729/3	Precursor T-cell lymphoblastic lymphoma
PLASMA CELL TUMORS	973	9731/3	Plasmacytoma, NOS
		9734/3	Plasmacytoma, extramedullary
		9735/3	Plasmablastic lymphoma
		9737/3	ALK positive large B-cell lymphoma
		9738/3	Lrg B-cell lymphoma in HHV8-assoc. multicentric Castleman DZ
MAST CELL TUMORS	974	9740/3	Mast cell sarcoma
		9741/3	Malignant mastocytosis
NEOPLASMS OF HISTIOCYTES AND ACCESSORY LYMPHOID CELLS	975	9750/3	Malignant histiocytosis
		9751/3	Langerhans cell histiocytosis, NOS
		9754/3	Langerhans cell histiocytosis, disseminated
		9755/3	Histiocytic sarcoma
		9756/3	Langerhans cell sarcoma
		9757/3	Interdigitating dendritic cell sarcoma
		9758/3	Follicular dendritic cell sarcoma
		9759/3	Fibroblastic reticular cell tumor
PRECURSOR LYMPHOID NEOPLASMS	981	9811/3	B lymphoblastic leukemia/lymphoma, NOS
		9812/3	Leukemia/lymphoma with t(9;22)(q34;q11.2);BCR-ABL1
		9813/3	Leukemia/lymphoma with t(v;11q23);MLL rearranged
		9814/3	Leukemia/lymphoma with t(12;21)(p13;q22);TEL-AML1(ETV6-RUNX1)
		9815/3	B lymphoblastic leukemia/lymphoma with hyperdiploidy
		9816/3	Leukemia/lymphoma with hypodiploidy (hypodiploid ALL)
		9817/3	B lymphblastic leukemia/lymphoma with t(5;14)(q31;q32);IL3-IGH
		9818/3	Leukemia/lymphoma with t(1;19)(q23;p13.3); E2A PBX1 (TCF3 PBX1)
LYMPHOID LEUKEMIA, NOS	982	9823/3	Chronic lymphocytic leukemia/small lymphocytic lymphoma
PROLYMPH/PRECURS LEUKEMIA	983	9831/3	T-cell large granular lymphocytic leukemia
		9837/3	T lymphoblastic leukemia/lymphoma

BASE OF TONGUE C019

CHRONIC MYELOPROLIFERATIVE DIS.	996	9965/3	Myeloid and lymphoid neoplasms with PDGFRB rearrangement
		9967/3	Myeloid and lymphoid neoplasm with FGFR1 abnormalities
MYELOPLASTIC/MYELOPROLIFERATIVE NEOPLASMS	997	9971/3	Polymorphic PTLD
		9975/3	Myelodysplastic/Myeloproliferative neoplasm, unclassifiable

TONGUE (EXCL BASE OF TONGUE) C020-C024,C028-C029

NEOPLASM	800	8000/3	Neoplasm, malignant
		8001/3	Tumor cells, malignant
		8002/3	Malignant tumor, small cell type
		8003/3	Malignant tumor, giant cell type
		8004/3	Malignant tumor, spindle cell type
		8005/3	Malignant tumor, clear cell type
CARCINOMA, NOS	801	8010/2	Carcinoma in situ, NOS
		8010/3	Carcinoma, NOS
		8011/3	Epithelioma, malignant
		8012/3	Large cell carcinoma, NOS
		8013/3	Large cell neuroendocrine carcinoma
		8014/3	Large cell carcinoma with rhabdoid phenotype
		8015/3	Glassy cell carcinoma
CARCINOMA, UNDIFF., NOS	802	8020/3	Carcinoma, undifferentiated type, NOS
		8021/3	Carcinoma, anaplastic type, NOS
		8022/3	Pleomorphic carcinoma
GIANT & SPINDLE CELL CARCINOMA	803	8030/3	Giant cell and spindle cell carcinoma
		8031/3	Giant cell carcinoma
		8032/3	Spindle cell carcinoma
		8033/3	Pseudosarcomatous carcinoma
		8034/3	Polygonal cell carcinoma
		8035/3	Carcinoma with osteoclast-like giant cells
PAPILLARY CARCINOMA, NOS	805	8050/2	Papillary carcinoma in situ
		8050/3	Papillary carcinoma, NOS
		8051/3	Verrucous carcinoma, NOS
		8052/2	Papillary squamous cell carcinoma, non-invasive
		8052/3	Papillary squamous cell carcinoma
SQUAMOUS CELL CARCINOMA, NOS	807	8070/2	Squamous cell carcinoma in situ, NOS
		8070/3	Squamous cell carcinoma, NOS
		8071/3	Sq. cell carcinoma, keratinizing, NOS
		8072/3	Sq. cell carcinoma, lg. cell, non-ker.
		8073/3	Sq. cell carcinoma, sm. cell, non-ker.
		8074/3	Sq. cell carcinoma, spindle cell
		8075/3	Squamous cell carcinoma, adenoid
		8076/2	Sq. cell carc. in situ with question. stromal invas.
		8076/3	Sq. cell carcinoma, micro-invasive
		8078/3	Squamous cell carcinoma with horn formation

TONGUE (EXCL BASE OF TONGUE) C020-C024,C028-C029

LYMPHOEPITHELIAL CARCINOMA	808	8081/2	Bowen disease
		8082/3	Lymphoepithelial carcinoma
		8083/3	Basaloid squamous cell carcinoma
		8084/3	Squamous cell carcinoma, clear cell type
ADENOCARCINOMA, NOS	814	8140/2	Adenocarcinoma in situ
		8140/3	Adenocarcinoma, NOS
		8141/3	Scirrhous adenocarcinoma
		8143/3	Superficial spreading adenocarcinoma
		8147/3	Basal cell adenocarcinoma
ADENOID CYSTIC & CRIBRIFORM CA.	820	8200/3	Adenoid cystic carcinoma
		8201/2	Cribriform carcinoma in situ
		8201/3	Cribriform carcinoma
BRONCHIOLO-ALVEOLAR ADENOCA.	825	8255/3	Adenocarcinoma with mixed subtypes
MUCOEPIDERMOID CARCINOMA	843	8430/3	Mucoepidermoid carcinoma
MUCINOUS ADENOCARCINOMA	848	8480/3	Mucinous adenocarcinoma
		8481/3	Mucin-producing adenocarcinoma
ADENOSQUAMOUS CARCINOMA	856	8560/3	Adenosquamous carcinoma
		8562/3	Epithelial-myoepithelial carcinoma
ADENOCA. WITH METAPLASIA	857	8570/3	Adenocarcinoma with squamous metaplasia
		8571/3	Adenocarcinoma w cartilag. & oss. metaplas.
		8572/3	Adenocarcinoma with spindle cell mataplasia
		8573/3	Adenocarcinoma with apocrine metaplasia
		8574/3	Adenocarcinoma with neuroendocrine differen.
		8575/3	Metaplastic carcinoma, NOS
NEVI & MELANOMAS	872	8720/2	Melanoma in situ
		8720/3	Malignant melanoma, NOS
		8721/3	Nodular melanoma
		8722/3	Balloon cell melanoma
		8723/3	Malignant melanoma, regressing
SARCOMA, NOS	882	8825/3	Myofibroblastic sarcoma
RHABDOMYOSARCOMA, NOS	890	8900/3	Rhabdomyosarcoma, NOS
		8901/3	Pleomorphic rhabdomyosarcoma, adult type
		8902/3	Mixed type rhabdomyosarcoma

TONGUE (EXCL BASE OF TONGUE) C020-C024,C028-C029

EMBRYONAL RHABDOMYOSARCOMA	891	8910/3	Embryonal rhabdomyosarcoma
		8912/3	Spindle cell rhabdomyosarcoma
MIXED TUMOR, MALIGNANT, NOS	894	8940/3	Mixed tumor, malignant, NOS
		8941/3	Carcinoma in pleomorphic adenoma
KAPOSI SARCOMA	914	9140/3	Kaposi sarcoma
MALIGNANT LYMPHOMA, NOS	959	9590/3	Malignant lymphoma, NOS
		9591/3	Malignant lymphoma, non-Hodgkin
		9596/3	Composite Hodgkin and non-Hodgkin lymphoma
HODGKIN LYMPHOMA	965	9650/3	Hodgkin lymphoma, NOS
		9651/3	Hodgkin lymphoma, lymphocyte-rich
		9652/3	Hodgkin lymphoma, mixed cellularity, NOS
		9653/3	Hodgkin lymphoma, lymphocytic deplet., NOS
		9654/3	Hodgkin lymph., lymphocyt. deplet., diffuse fibrosis
		9655/3	Hodgkin lymphoma, lymphocyt. deplet., reticular
		9659/3	Hodgkin lymph., nodular lymphocyte predom.
HODGKIN LYMPHOMA, NOD. SCLER.	966	9661/3	Hodgkin granuloma [obs]
		9662/3	Hodgkin sarcoma [obs]
		9663/3	Hodgkin lymphoma, nodular sclerosis, NOS
		9664/3	Hodgkin lymphoma, nod. scler., cellular phase
		9665/3	Hodgkin lymphoma, nod. scler., grade 1
		9667/3	Hodgkin lymphoma, nod. scler., grade 2
ML, SMALL B-CELL LYMPHOCYTIC	967	9670/3	ML, small B lymphocytic, NOS
		9671/3	ML, lymphoplasmacytic
		9673/3	Mantle cell lymphoma
		9675/3	ML, mixed sm. and lg. cell, diffuse
ML, LARGE B-CELL, DIFFUSE	968	9680/3	ML, large B-cell, diffuse
		9684/3	ML, large B-cell, diffuse, immunoblastic, NOS
		9687/3	Burkitt lymphoma, NOS
		9688/3	T-cell histiocyte rich large B-cell lymphoma
FOLLIC. & MARGINAL LYMPH, NOS	969	9690/3	Follicular lymphoma, NOS
		9691/3	Follicular lymphoma, grade 2
		9695/3	Follicular lymphoma, grade 1
		9698/3	Follicular lymphoma, grade 3
		9699/3	Marginal zone B-cell lymphoma, NOS

TONGUE (EXCL BASE OF TONGUE) C020-C024,C028-C029

T-CELL LYMPHOMAS	970	9701/3	Sezary syndrome
		9702/3	Mature T-cell lymphoma, NOS
		9705/3	Angioimmunoblastic T-cell lymphoma
OTHER SPEC. NON-HODGKIN LYMPHOMA	971	9712/3	Intravascular large B-cell lymphoma
		9714/3	Anaplastic large cell lymphoma, T-cell and Null cell type
		9719/3	NK/T-cell lymphoma, nasal and nasal-type
PRECURS. CELL LYMPHOBLASTIC LYMPH.	972	9724/3	SystemicEBV pos. T-cell lymphoproliferative disease of childhood
		9727/3	Precursor cell lymphoblastic lymphoma, NOS
		9728/3	Precursor B-cell lymphoblastic lymphoma
		9729/3	Precursor T-cell lymphoblastic lymphoma
PLASMA CELL TUMORS	973	9731/3	Plasmacytoma, NOS
		9734/3	Plasmacytoma, extramedullary
		9735/3	Plasmablastic lymphoma
		9737/3	ALK positive large B-cell lymphoma
		9738/3	Lrg B-cell lymphoma in HHV8-assoc. multicentric Castleman DZ
MAST CELL TUMORS	974	9740/3	Mast cell sarcoma
		9741/3	Malignant mastocytosis
NEOPLASMS OF HISTIOCYTES AND ACCESSORY LYMPHOID CELLS	975	9750/3	Malignant histiocytosis
		9751/3	Langerhans cell histiocytosis, NOS
		9754/3	Langerhans cell histiocytosis, disseminated
		9755/3	Histiocytic sarcoma
		9756/3	Langerhans cell sarcoma
		9757/3	Interdigitating dendritic cell sarcoma
		9758/3	Follicular dendritic cell sarcoma
		9759/3	Fibroblastic reticular cell tumor
PRECURSOR LYMPHOID NEOPLASMS	981	9811/3	B lymphoblastic leukemia/lymphoma, NOS
		9812/3	Leukemia/lymphoma with t(9;22)(q34;q11.2);BCR-ABL1
		9813/3	Leukemia/lymphoma with t(v;11q23);MLL rearranged
		9814/3	Leukemia/lymphoma with t(12;21)(p13;q22);TEL-AML1(ETV6-RUNX1)
		9815/3	B lymphoblastic leukemia/lymphoma with hyperdiploidy
		9816/3	Leukemia/lymphoma with hypodiploidy (hypodiploid ALL)
		9817/3	B lymphblastic leukemia/lymphoma with t(5;14)(q31;q32);IL3-IGH
		9818/3	Leukemia/lymphoma with t(1;19)(q23;p13.3); E2A PBX1 (TCF3 PBX1)
LYMPHOID LEUKEMIA, NOS	982	9823/3	Chronic lymphocytic leukemia/small lymphocytic lymphoma
PROLYMPH/PRECURS LEUKEMIA	983	9831/3	T-cell large granular lymphocytic leukemia
		9837/3	T lymphoblastic leukemia/lymphoma

TONGUE (EXCL BASE OF TONGUE) C020-C024,C028-C029

CHRONIC MYELOPROLIFERATIVE DIS.	996	9965/3	Myeloid and lymphoid neoplasms with PDGFRB rearrangement
		9967/3	Myeloid and lymphoid neoplasm with FGFR1 abnormalities
MYELOPLASTIC/MYELOPROLIFERATIVE NEOPLASMS	997	9971/3	Polymorphic PTLD
		9975/3	Myelodysplastic/Myeloproliferative neoplasm, unclassifiable

GUM, FLOOR OF MOUTH, & OTHER MOUTH C030-C031,C039-C041,C048-C052,C058-C062,C068-C069

NEOPLASM	800	8000/3	Neoplasm, malignant
		8001/3	Tumor cells, malignant
		8002/3	Malignant tumor, small cell type
		8003/3	Malignant tumor, giant cell type
		8004/3	Malignant tumor, spindle cell type
		8005/3	Malignant tumor, clear cell type
CARCINOMA, NOS	801	8010/2	Carcinoma in situ, NOS
		8010/3	Carcinoma, NOS
		8011/3	Epithelioma, malignant
		8012/3	Large cell carcinoma, NOS
		8013/3	Large cell neuroendocrine carcinoma
		8014/3	Large cell carcinoma with rhabdoid phenotype
		8015/3	Glassy cell carcinoma
CARCINOMA, UNDIFF., NOS	802	8020/3	Carcinoma, undifferentiated type, NOS
		8021/3	Carcinoma, anaplastic type, NOS
		8022/3	Pleomorphic carcinoma
GIANT & SPINDLE CELL CARCINOMA	803	8030/3	Giant cell and spindle cell carcinoma
		8031/3	Giant cell carcinoma
		8032/3	Spindle cell carcinoma
		8033/3	Pseudosarcomatous carcinoma
		8034/3	Polygonal cell carcinoma
		8035/3	Carcinoma with osteoclast-like giant cells
PAPILLARY CARCINOMA, NOS	805	8050/2	Papillary carcinoma in situ
		8050/3	Papillary carcinoma, NOS
		8051/3	Verrucous carcinoma, NOS
		8052/2	Papillary squamous cell carcinoma, non-invasive
		8052/3	Papillary squamous cell carcinoma
SQUAMOUS CELL CARCINOMA, NOS	807	8070/2	Squamous cell carcinoma in situ, NOS
		8070/3	Squamous cell carcinoma, NOS
		8071/3	Sq. cell carcinoma, keratinizing, NOS
		8072/3	Sq. cell carcinoma, lg. cell, non-ker.
		8073/3	Sq. cell carcinoma, sm. cell, non-ker.
		8074/3	Sq. cell carcinoma, spindle cell
		8075/3	Squamous cell carcinoma, adenoid
		8076/2	Sq. cell carc. in situ with question. stromal invas.
		8076/3	Sq. cell carcinoma, micro-invasive
		8078/3	Squamous cell carcinoma with horn formation

GUM, FLOOR OF MOUTH, & OTHER MOUTH C030-C031,C039-C041,C048-C052,C058-C062,C068-C069

LYMPHOEPITHELIAL CARCINOMA	808	8081/2	Bowen disease
		8082/3	Lymphoepithelial carcinoma
		8083/3	Basaloid squamous cell carcinoma
		8084/3	Squamous cell carcinoma, clear cell type
ADENOCARCINOMA, NOS	814	8140/2	Adenocarcinoma in situ
		8140/3	Adenocarcinoma, NOS
		8141/3	Scirrhous adenocarcinoma
		8143/3	Superficial spreading adenocarcinoma
		8147/3	Basal cell adenocarcinoma
ADENOID CYSTIC & CRIBRIFORM CA.	820	8200/3	Adenoid cystic carcinoma
		8201/2	Cribriform carcinoma in situ
		8201/3	Cribriform carcinoma
BRONCHIOLO-ALVEOLAR ADENOCA.	825	8255/3	Adenocarcinoma with mixed subtypes
PAPILLARY ADENOCARCINOMA, NOS	826	8260/3	Papillary adenocarcinoma, NOS
		8261/2	Adenocarcinoma in situ in villous adenoma
		8261/3	Adenocarcinoma in villous adenoma
		8262/3	Villous adenocarcinoma
		8263/2	Adenocarcinoma in situ in tubulovillous adenoma
		8263/3	Adenocarcinoma in tubulovillous adenoma
CLEAR CELL ADENOCARCINOMA, NOS	831	8310/3	Clear cell adenocarcinoma, NOS
MUCOEPIDERMOID CARCINOMA	843	8430/3	Mucoepidermoid carcinoma
CYSTADENOCARCINOMA, NOS	844	8440/3	Cystadenocarcinoma, NOS
MUCINOUS ADENOCARCINOMA	848	8480/3	Mucinous adenocarcinoma
		8481/3	Mucin-producing adenocarcinoma
ACINAR CELL CARCINOMA	855	8550/3	Acinar cell carcinoma
		8551/3	Acinar cell cystadenocarcinoma
NEVI & MELANOMAS	872	8720/2	Melanoma in situ
		8720/3	Malignant melanoma, NOS
		8721/3	Nodular melanoma
		8722/3	Balloon cell melanoma
		8723/3	Malignant melanoma, regressing
AMELANOTIC MELANOMA	873	8730/3	Amelanotic melanoma

GUM, FLOOR OF MOUTH, & OTHER MOUTH C030-C031,C039-C041,C048-C052,C058-C062,C068-C069

MAL. MEL. IN JUNCT. NEVUS	874	8743/3	Superficial spreading melanoma
		8745/3	Desmoplastic melanoma, malignant
		8746/3	Mucosal lentiginous melanoma
EPITHELIOID CELL MELANOMA	877	8770/3	Mixed epithel. & spindle cell melanoma
		8771/3	Epithelioid cell melanoma
		8772/3	Spindle cell melanoma, NOS
FIBROMATOUS NEOPLASMS	881	8810/3	Fibrosarcoma, NOS
		8811/3	Fibromyxosarcoma
		8813/3	Fascial fibrosarcoma
		8814/3	Infantile fibrosarcoma
		8815/3	Solitary fibrous tumor, malignant
SARCOMA, NOS	882	8825/3	Myofibroblastic sarcoma
RHABDOMYOSARCOMA, NOS	890	8900/3	Rhabdomyosarcoma, NOS
		8901/3	Pleomorphic rhabdomyosarcoma, adult type
		8902/3	Mixed type rhabdomyosarcoma
EMBRYONAL RHABDOMYOSARCOMA	891	8910/3	Embryonal rhabdomyosarcoma
		8912/3	Spindle cell rhabdomyosarcoma
MIXED TUMOR, MALIGNANT, NOS	894	8940/3	Mixed tumor, malignant, NOS
		8941/3	Carcinoma in pleomorphic adenoma
CARCINOSARCOMA, NOS	898	8980/3	Carcinosarcoma, NOS
		8981/3	Carcinosarcoma, embryonal type
		8982/3	Malignant myoepithelioma
KAPOSI SARCOMA	914	9140/3	Kaposi sarcoma
MALIGNANT LYMPHOMA, NOS	959	9590/3	Malignant lymphoma, NOS
		9591/3	Malignant lymphoma, non-Hodgkin
		9596/3	Composite Hodgkin and non-Hodgkin lymphoma
HODGKIN LYMPHOMA	965	9650/3	Hodgkin lymphoma, NOS
		9651/3	Hodgkin lymphoma, lymphocyte-rich
		9652/3	Hodgkin lymphoma, mixed cellularity, NOS
		9653/3	Hodgkin lymphoma, lymphocytic deplet., NOS
		9654/3	Hodgkin lymph., lymphocyt. deplet., diffuse fibrosis
		9655/3	Hodgkin lymphoma, lymphocyt. deplet., reticular
		9659/3	Hodgkin lymph., nodular lymphocyte predom.

GUM, FLOOR OF MOUTH, & OTHER MOUTH C030-C031,C039-C041,C048-C052,C058-C062,C068-C069

HODGKIN LYMPHOMA, NOD. SCLER.	966	9661/3	Hodgkin granuloma [obs]
		9662/3	Hodgkin sarcoma [obs]
		9663/3	Hodgkin lymphoma, nodular sclerosis, NOS
		9664/3	Hodgkin lymphoma, nod. scler., cellular phase
		9665/3	Hodgkin lymphoma, nod. scler., grade 1
		9667/3	Hodgkin lymphoma, nod. scler., grade 2
ML, SMALL B-CELL LYMPHOCYTIC	967	9670/3	ML, small B lymphocytic, NOS
		9671/3	ML, lymphoplasmacytic
		9673/3	Mantle cell lymphoma
		9675/3	ML, mixed sm. and lg. cell, diffuse
ML, LARGE B-CELL, DIFFUSE	968	9680/3	ML, large B-cell, diffuse
		9684/3	ML, large B-cell, diffuse, immunoblastic, NOS
		9687/3	Burkitt lymphoma, NOS
		9688/3	T-cell histiocyte rich large B-cell lymphoma
FOLLIC. & MARGINAL LYMPH, NOS	969	9690/3	Follicular lymphoma, NOS
		9691/3	Follicular lymphoma, grade 2
		9695/3	Follicular lymphoma, grade 1
		9698/3	Follicular lymphoma, grade 3
		9699/3	Marginal zone B-cell lymphoma, NOS
T-CELL LYMPHOMAS	970	9701/3	Sezary syndrome
		9702/3	Mature T-cell lymphoma, NOS
		9705/3	Angioimmunoblastic T-cell lymphoma
OTHER SPEC. NON-HODGKIN LYMPHOMA	971	9712/3	Intravascular large B-cell lymphoma
		9714/3	Anaplastic large cell lymphoma, T-cell and Null cell type
		9719/3	NK/T-cell lymphoma, nasal and nasal-type
PRECURS. CELL LYMPHOBLASTIC LYMPH.	972	9724/3	SystemicEBV pos. T-cell lymphoproliferative disease of childhood
		9727/3	Precursor cell lymphoblastic lymphoma, NOS
		9728/3	Precursor B-cell lymphoblastic lymphoma
		9729/3	Precursor T-cell lymphoblastic lymphoma
PLASMA CELL TUMORS	973	9731/3	Plasmacytoma, NOS
		9734/3	Plasmacytoma, extramedullary
		9735/3	Plasmablastic lymphoma
		9737/3	ALK positive large B-cell lymphoma
MAST CELL TUMORS	974	9740/3	Mast cell sarcoma
		9741/3	Malignant mastocytosis

GUM, FLOOR OF MOUTH, & OTHER MOUTH C030-C031,C039-C041,C048-C052,C058-C062,C068-C069

NEOPLASMS OF HISTIOCYTES AND ACCESSORY LYMPHOID CELLS	975	9750/3	Malignant histiocytosis
		9751/3	Langerhans cell histiocytosis, NOS
		9754/3	Langerhans cell histiocytosis, disseminated
		9755/3	Histiocytic sarcoma
		9756/3	Langerhans cell sarcoma
		9757/3	Interdigitating dendritic cell sarcoma
		9758/3	Follicular dendritic cell sarcoma
		9759/3	Fibroblastic reticular cell tumor
PRECURSOR LYMPHOID NEOPLASMS	981	9811/3	B lymphoblastic leukemia/lymphoma, NOS
		9812/3	Leukemia/lymphoma with t(9;22)(q34;q11.2);BCR-ABL1
		9813/3	Leukemia/lymphoma with t(v;11q23);MLL rearranged
		9814/3	Leukemia/lymphoma with t(12;21)(p13;q22);TEL-AML1(ETV6-RUNX1)
		9815/3	B lymphoblastic leukemia/lymphoma with hyperdiploidy
		9816/3	Leukemia/lymphoma with hypodiploidy (hypodiploid ALL)
		9817/3	B lymphblastic leukemia/lymphoma with t(5;14)(q31;q32);IL3-IGH
		9818/3	Leukemia/lymphoma with t(1;19)(q23;p13.3); E2A PBX1 (TCF3 PBX1)
LYMPHOID LEUKEMIA, NOS	982	9823/3	Chronic lymphocytic leukemia/small lymphocytic lymphoma
PROLYMPH/PRECURS LEUKEMIA	983	9831/3	T-cell large granular lymphocytic leukemia
		9837/3	T lymphoblastic leukemia/lymphoma
CHRONIC MYELOPROLIFERATIVE DIS.	996	9965/3	Myeloid and lymphoid neoplasms with PDGFRB rearrangement
		9967/3	Myeloid and lymphoid neoplasm with FGFR1 abnormalities
MYELOPLASTIC/MYELOPROLIFERATIVE NEOPLASMS	997	9971/3	Polymorphic PTLD
		9975/3	Myelodysplastic/Myeloproliferative neoplasm, unclassifiable

SALIVARYGLAND C079-C081,C088-C089

NEOPLASM	800	8000/3	Neoplasm, malignant
		8001/3	Tumor cells, malignant
		8002/3	Malignant tumor, small cell type
		8003/3	Malignant tumor, giant cell type
		8004/3	Malignant tumor, spindle cell type
		8005/3	Malignant tumor, clear cell type
CARCINOMA, NOS	801	8010/2	Carcinoma in situ, NOS
		8010/3	Carcinoma, NOS
		8011/3	Epithelioma, malignant
		8012/3	Large cell carcinoma, NOS
		8013/3	Large cell neuroendocrine carcinoma
		8014/3	Large cell carcinoma with rhabdoid phenotype
		8015/3	Glassy cell carcinoma
CARCINOMA, UNDIFF., NOS	802	8020/3	Carcinoma, undifferentiated type, NOS
		8021/3	Carcinoma, anaplastic type, NOS
		8022/3	Pleomorphic carcinoma
GIANT & SPINDLE CELL CARCINOMA	803	8030/3	Giant cell and spindle cell carcinoma
		8031/3	Giant cell carcinoma
		8032/3	Spindle cell carcinoma
		8033/3	Pseudosarcomatous carcinoma
		8034/3	Polygonal cell carcinoma
		8035/3	Carcinoma with osteoclast-like giant cells
PAPILLARY CARCINOMA, NOS	805	8050/2	Papillary carcinoma in situ
		8050/3	Papillary carcinoma, NOS
		8052/2	Papillary squamous cell carcinoma, non-invasive
		8052/3	Papillary squamous cell carcinoma
SQUAMOUS CELL CARCINOMA, NOS	807	8070/2	Squamous cell carcinoma in situ, NOS
		8070/3	Squamous cell carcinoma, NOS
		8071/3	Sq. cell carcinoma, keratinizing, NOS
		8072/3	Sq. cell carcinoma, lg. cell, non-ker.
		8073/3	Sq. cell carcinoma, sm. cell, non-ker.
		8074/3	Sq. cell carcinoma, spindle cell
		8075/3	Squamous cell carcinoma, adenoid
		8076/2	Sq. cell carc. in situ with question. stromal invas.
		8076/3	Sq. cell carcinoma, micro-invasive
		8078/3	Squamous cell carcinoma with horn formation
LYMPHOEPITHELIAL CARCINOMA	808	8082/3	Lymphoepithelial carcinoma
		8083/3	Basaloid squamous cell carcinoma
		8084/3	Squamous cell carcinoma, clear cell type

SALIVARYGLAND C079-C081,C088-C089

ADENOCARCINOMA, NOS	814	8140/2	Adenocarcinoma in situ
		8140/3	Adenocarcinoma, NOS
		8141/3	Scirrhous adenocarcinoma
		8143/3	Superficial spreading adenocarcinoma
		8147/3	Basal cell adenocarcinoma
TRABECULAR ADENOCARCINOMA	819	8190/3	Trabecular adenocarcinoma
ADENOID CYSTIC & CRIBRIFORM CA.	820	8200/3	Adenoid cystic carcinoma
		8201/2	Cribriform carcinoma in situ
		8201/3	Cribriform carcinoma
SOLID CARCINOMA, NOS	823	8230/2	Duct carcinoma in situ, solid type
		8230/3	Solid carcinoma, NOS
		8231/3	Carcinoma simplex
BRONCHIOLO-ALVEOLAR ADENOCA.	825	8255/3	Adenocarcinoma with mixed subtypes
PAPILLARY ADENOCARCINOMA, NOS	826	8260/3	Papillary adenocarcinoma, NOS
		8261/2	Adenocarcinoma in situ in villous adenoma
		8261/3	Adenocarcinoma in villous adenoma
		8262/3	Villous adenocarcinoma
		8263/2	Adenocarcinoma in situ in tubulovillous adenoma
		8263/3	Adenocarcinoma in tubulovillous adenoma
OXYPHILIC ADENOCARCINOMA	829	8290/3	Oxyphilic adenocarcinoma
CLEAR CELL ADENOCARCINOMA, NOS	831	8310/3	Clear cell adenocarcinoma, NOS
MUCOEPIDERMOID CARCINOMA	843	8430/3	Mucoepidermoid carcinoma
CYSTADENOCARCINOMA, NOS	844	8440/3	Cystadenocarcinoma, NOS
MUCINOUS ADENOCARCINOMA	848	8480/3	Mucinous adenocarcinoma
		8481/3	Mucin-producing adenocarcinoma
DUCT CARCINOMA	850	8500/3	Invasive carcinoma of no special type
ACINAR CELL CARCINOMA	855	8550/3	Acinar cell carcinoma
		8551/3	Acinar cell cystadenocarcinoma
ADENOSQUAMOUS CARCINOMA	856	8560/3	Adenosquamous carcinoma
		8561/3	Warthin tumor, malignant
		8562/3	Epithelial-myoepithelial carcinoma

SALIVARYGLAND C079-C081,C088-C089

ADENOCA. WITH METAPLASIA	857	8570/3	Adenocarcinoma with squamous metaplasia
		8571/3	Adenocarcinoma w cartilag. & oss. metaplas.
		8572/3	Adenocarcinoma with spindle cell mataplasia
		8573/3	Adenocarcinoma with apocrine metaplasia
		8574/3	Adenocarcinoma with neuroendocrine differen.
		8575/3	Metaplastic carcinoma, NOS
SARCOMA, NOS	880	8800/3	Sarcoma, NOS
		8801/3	Spindle cell sarcoma
		8802/3	Giant cell sarcoma
		8803/3	Small cell sarcoma
		8804/3	Epithelioid sarcoma
		8805/3	Undifferentiated sarcoma
		8806/3	Desmoplastic small round cell tumor
FIBROMATOUS NEOPLASMS	881	8810/3	Fibrosarcoma, NOS
		8811/3	Fibromyxosarcoma
		8813/3	Fascial fibrosarcoma
		8814/3	Infantile fibrosarcoma
		8815/3	Solitary fibrous tumor, malignant
SARCOMA, NOS	882	8825/3	Myofibroblastic sarcoma
MYXOSARCOMA	884	8840/3	Myxosarcoma
LIPOSARCOMA NEOPLASMS	885	8850/3	Liposarcoma, NOS
		8851/3	Liposarcoma, well differentiated
		8852/3	Myxoid liposarcoma
		8853/3	Round cell liposarcoma
		8854/3	Pleomorphic liposarcoma
		8855/3	Mixed type liposarcoma
		8857/3	Fibroblastic liposarcoma
		8858/3	Dedifferentiated liposarcoma
MYOMATOUS NEOPLASMS	889	8890/3	Leiomyosarcoma, NOS
		8891/3	Epithelioid leiomyosarcoma
		8894/3	Angiomyosarcoma
		8895/3	Myosarcoma
		8896/3	Myxoid leiomyosarcoma
RHABDOMYOSARCOMA, NOS	890	8900/3	Rhabdomyosarcoma, NOS
		8901/3	Pleomorphic rhabdomyosarcoma, adult type
		8902/3	Mixed type rhabdomyosarcoma

SALIVARYGLAND C079-C081,C088-C089

EMBRYONAL RHABDOMYOSARCOMA	891	8910/3	Embryonal rhabdomyosarcoma
		8912/3	Spindle cell rhabdomyosarcoma
MIXED TUMOR, MALIGNANT, NOS	894	8940/3	Mixed tumor, malignant, NOS
		8941/3	Carcinoma in pleomorphic adenoma
CARCINOSARCOMA, NOS	898	8980/3	Carcinosarcoma, NOS
		8981/3	Carcinosarcoma, embryonal type
		8982/3	Malignant myoepithelioma
MESENCHYMOMA, MALIGNANT	899	8990/3	Mesenchymoma, malignant
		8991/3	Embryonal sarcoma
KAPOSI SARCOMA	914	9140/3	Kaposi sarcoma
MALIGNANT LYMPHOMA, NOS	959	9590/3	Malignant lymphoma, NOS
		9591/3	Malignant lymphoma, non-Hodgkin
		9596/3	Composite Hodgkin and non-Hodgkin lymphoma
HODGKIN LYMPHOMA	965	9650/3	Hodgkin lymphoma, NOS
		9651/3	Hodgkin lymphoma, lymphocyte-rich
		9652/3	Hodgkin lymphoma, mixed cellularity, NOS
		9653/3	Hodgkin lymphoma, lymphocytic deplet., NOS
		9654/3	Hodgkin lymph., lymphocyt. deplet., diffuse fibrosis
		9655/3	Hodgkin lymphoma, lymphocyt. deplet., reticular
		9659/3	Hodgkin lymph., nodular lymphocyte predom.
HODGKIN LYMPHOMA, NOD. SCLER.	966	9661/3	Hodgkin granuloma [obs]
		9662/3	Hodgkin sarcoma [obs]
		9663/3	Hodgkin lymphoma, nodular sclerosis, NOS
		9664/3	Hodgkin lymphoma, nod. scler., cellular phase
		9665/3	Hodgkin lymphoma, nod. scler., grade 1
		9667/3	Hodgkin lymphoma, nod. scler., grade 2
ML, SMALL B-CELL LYMPHOCYTIC	967	9670/3	ML, small B lymphocytic, NOS
		9671/3	ML, lymphoplasmacytic
		9673/3	Mantle cell lymphoma
		9675/3	ML, mixed sm. and lg. cell, diffuse
ML, LARGE B-CELL, DIFFUSE	968	9680/3	ML, large B-cell, diffuse
		9684/3	ML, large B-cell, diffuse, immunoblastic, NOS
		9687/3	Burkitt lymphoma, NOS
		9688/3	T-cell histiocyte rich large B-cell lymphoma

SALIVARYGLAND C079-C081,C088-C089

FOLLIC. & MARGINAL LYMPH, NOS	969	9690/3	Follicular lymphoma, NOS
		9691/3	Follicular lymphoma, grade 2
		9695/3	Follicular lymphoma, grade 1
		9698/3	Follicular lymphoma, grade 3
		9699/3	Marginal zone B-cell lymphoma, NOS
T-CELL LYMPHOMAS	970	9701/3	Sezary syndrome
		9702/3	Mature T-cell lymphoma, NOS
		9705/3	Angioimmunoblastic T-cell lymphoma
OTHER SPEC. NON-HODGKIN LYMPHOMA	971	9712/3	Intravascular large B-cell lymphoma
		9714/3	Anaplastic large cell lymphoma, T-cell and Null cell type
		9719/3	NK/T-cell lymphoma, nasal and nasal-type
PRECURS. CELL LYMPHOBLASTIC LYMPH.	972	9724/3	SystemicEBV pos. T-cell lymphoproliferative disease of childhood
		9727/3	Precursor cell lymphoblastic lymphoma, NOS
		9728/3	Precursor B-cell lymphoblastic lymphoma
		9729/3	Precursor T-cell lymphoblastic lymphoma
PLASMA CELL TUMORS	973	9731/3	Plasmacytoma, NOS
		9734/3	Plasmacytoma, extramedullary
		9735/3	Plasmablastic lymphoma
		9737/3	ALK positive large B-cell lymphoma
		9738/3	Lrg B-cell lymphoma in HHV8-assoc. multicentric Castleman DZ
MAST CELL TUMORS	974	9740/3	Mast cell sarcoma
		9741/3	Malignant mastocytosis
NEOPLASMS OF HISTIOCYTES AND ACCESSORY LYMPHOID CELLS	975	9750/3	Malignant histiocytosis
		9751/3	Langerhans cell histiocytosis, NOS
		9754/3	Langerhans cell histiocytosis, disseminated
		9755/3	Histiocytic sarcoma
		9756/3	Langerhans cell sarcoma
		9757/3	Interdigitating dendritic cell sarcoma
		9758/3	Follicular dendritic cell sarcoma
		9759/3	Fibroblastic reticular cell tumor
PRECURSOR LYMPHOID NEOPLASMS	981	9811/3	B lymphoblastic leukemia/lymphoma, NOS
		9812/3	Leukemia/lymphoma with t(9;22)(q34;q11.2);BCR-ABL1
		9813/3	Leukemia/lymphoma with t(v;11q23);MLL rearranged
		9814/3	Leukemia/lymphoma with t(12;21)(p13;q22);TEL-AML1(ETV6-RUNX1)
		9815/3	B lymphoblastic leukemia/lymphoma with hyperdiploidy
		9816/3	Leukemia/lymphoma with hypodiploidy (hypodiploid ALL)
		9817/3	B lymphblastic leukemia/lymphoma with t(5;14)(q31;q32);IL3-IGH
		9818/3	Leukemia/lymphoma with t(1;19)(q23;p13.3); E2A PBX1 (TCF3 PBX1)
LYMPHOID LEUKEMIA, NOS	982	9823/3	Chronic lymphocytic leukemia/small lymphocytic lymphoma

SALIVARYGLAND C079-C081,C088-C089

PROLYMPH/PRECURS LEUKEMIA	983	9831/3	T-cell large granular lymphocytic leukemia
		9837/3	T lymphoblastic leukemia/lymphoma
CHRONIC MYELOPROLIFERATIVE DIS.	996	9965/3	Myeloid and lymphoid neoplasms with PDGFRB rearrangement
		9967/3	Myeloid and lymphoid neoplasm with FGFR1 abnormalities
MYELOPLASTIC/MYELOPROLIFERATIVE NEOPLASMS	997	9971/3	Polymorphic PTLD
		9975/3	Myelodysplastic/Myeloproliferative neoplasm, unclassifiable

OROPHARNYX C090-C091,C098-C104,C108-C109

NEOPLASM	800	8000/3	Neoplasm, malignant
		8001/3	Tumor cells, malignant
		8002/3	Malignant tumor, small cell type
		8003/3	Malignant tumor, giant cell type
		8004/3	Malignant tumor, spindle cell type
		8005/3	Malignant tumor, clear cell type
CARCINOMA, NOS	801	8010/2	Carcinoma in situ, NOS
		8010/3	Carcinoma, NOS
		8011/3	Epithelioma, malignant
		8012/3	Large cell carcinoma, NOS
		8013/3	Large cell neuroendocrine carcinoma
		8014/3	Large cell carcinoma with rhabdoid phenotype
		8015/3	Glassy cell carcinoma
CARCINOMA, UNDIFF., NOS	802	8020/3	Carcinoma, undifferentiated type, NOS
		8021/3	Carcinoma, anaplastic type, NOS
		8022/3	Pleomorphic carcinoma
GIANT & SPINDLE CELL CARCINOMA	803	8030/3	Giant cell and spindle cell carcinoma
		8031/3	Giant cell carcinoma
		8032/3	Spindle cell carcinoma
		8033/3	Pseudosarcomatous carcinoma
		8034/3	Polygonal cell carcinoma
		8035/3	Carcinoma with osteoclast-like giant cells
PAPILLARY CARCINOMA, NOS	805	8050/2	Papillary carcinoma in situ
		8050/3	Papillary carcinoma, NOS
		8051/3	Verrucous carcinoma, NOS
		8052/2	Papillary squamous cell carcinoma, non-invasive
		8052/3	Papillary squamous cell carcinoma
SQUAMOUS CELL CARCINOMA, NOS	807	8070/2	Squamous cell carcinoma in situ, NOS
		8070/3	Squamous cell carcinoma, NOS
		8071/3	Sq. cell carcinoma, keratinizing, NOS
		8072/3	Sq. cell carcinoma, lg. cell, non-ker.
		8073/3	Sq. cell carcinoma, sm. cell, non-ker.
		8074/3	Sq. cell carcinoma, spindle cell
		8075/3	Squamous cell carcinoma, adenoid
		8076/2	Sq. cell carc. in situ with question. stromal invas.
		8076/3	Sq. cell carcinoma, micro-invasive
		8078/3	Squamous cell carcinoma with horn formation

OROPHARNYX C090-C091,C098-C104,C108-C109

LYMPHOEPITHELIAL CARCINOMA	808	8081/2	Bowen disease
		8082/3	Lymphoepithelial carcinoma
		8083/3	Basaloid squamous cell carcinoma
		8084/3	Squamous cell carcinoma, clear cell type
		8085/3	Squamous cell carcinoma, HPV-positive
		8086/3	Squamous cell carcinoma, HPV-negative
TRANSITIONAL CELL CARCINOMA, NOS	812	8120/2	Transitional cell carcinoma in situ
		8120/3	Transitional cell carcinoma, NOS
		8121/3	Schneiderian carcinoma
		8122/3	Trans. cell carcinoma, spindle cell
		8123/3	Basaloid carcinoma
		8124/3	Cloacogenic carcinoma
ADENOCARCINOMA, NOS	814	8140/2	Adenocarcinoma in situ
		8140/3	Adenocarcinoma, NOS
		8141/3	Scirrhous adenocarcinoma
		8143/3	Superficial spreading adenocarcinoma
		8147/3	Basal cell adenocarcinoma
ADENOID CYSTIC & CRIBRIFORM CA.	820	8200/3	Adenoid cystic carcinoma
		8201/2	Cribriform carcinoma in situ
		8201/3	Cribriform carcinoma
BRONCHIOLO-ALVEOLAR ADENOCA.	825	8255/3	Adenocarcinoma with mixed subtypes
PAPILLARY ADENOCARCINOMA, NOS	826	8260/3	Papillary adenocarcinoma, NOS
		8261/2	Adenocarcinoma in situ in villous adenoma
		8261/3	Adenocarcinoma in villous adenoma
		8262/3	Villous adenocarcinoma
		8263/2	Adenocarcinoma in situ in tubulovillous adenoma
		8263/3	Adenocarcinoma in tubulovillous adenoma
MUCOEPIDERMOID CARCINOMA	843	8430/3	Mucoepidermoid carcinoma
MUCINOUS ADENOCARCINOMA	848	8480/3	Mucinous adenocarcinoma
		8481/3	Mucin-producing adenocarcinoma
NEVI & MELANOMAS	872	8720/2	Melanoma in situ
		8720/3	Malignant melanoma, NOS
		8721/3	Nodular melanoma
		8722/3	Balloon cell melanoma
		8723/3	Malignant melanoma, regressing
AMELANOTIC MELANOMA	873	8730/3	Amelanotic melanoma

OROPHARYNX C090-C091,C098-C104,C108-C109

MAL. MEL. IN JUNCT. NEVUS	874	8743/3	Superficial spreading melanoma
		8745/3	Desmoplastic melanoma, malignant
		8746/3	Mucosal lentiginous melanoma
MAL. MELAN. IN GIANT PIGMT. NEVUS	876	8761/3	Mal. melanoma in giant pigmented nevus
EPITHELIOID CELL MELANOMA	877	8770/3	Mixed epithel. & spindle cell melanoma
		8771/3	Epithelioid cell melanoma
		8772/3	Spindle cell melanoma, NOS
SARCOMA, NOS	880	8800/3	Sarcoma, NOS
		8801/3	Spindle cell sarcoma
		8802/3	Giant cell sarcoma
		8803/3	Small cell sarcoma
		8804/3	Epithelioid sarcoma
		8805/3	Undifferentiated sarcoma
		8806/3	Desmoplastic small round cell tumor
LIPOSARCOMA NEOPLASMS	885	8850/3	Liposarcoma, NOS
		8851/3	Liposarcoma, well differentiated
		8852/3	Myxoid liposarcoma
		8853/3	Round cell liposarcoma
		8854/3	Pleomorphic liposarcoma
		8855/3	Mixed type liposarcoma
		8857/3	Fibroblastic liposarcoma
		8858/3	Dedifferentiated liposarcoma
RHABDOMYOSARCOMA, NOS	890	8900/3	Rhabdomyosarcoma, NOS
		8901/3	Pleomorphic rhabdomyosarcoma, adult type
		8902/3	Mixed type rhabdomyosarcoma
EMBRYONAL RHABDOMYOSARCOMA	891	8910/3	Embryonal rhabdomyosarcoma
		8912/3	Spindle cell rhabdomyosarcoma
MIXED TUMOR, MALIGNANT, NOS	894	8940/3	Mixed tumor, malignant, NOS
		8941/3	Carcinoma in pleomorphic adenoma
CARCINOSARCOMA, NOS	898	8980/3	Carcinosarcoma, NOS
		8981/3	Carcinosarcoma, embryonal type
		8982/3	Malignant myoepithelioma
KAPOSI SARCOMA	914	9140/3	Kaposi sarcoma

OROPHARNYX C090-C091,C098-C104,C108-C109

MALIGNANT LYMPHOMA, NOS	959	9590/3	Malignant lymphoma, NOS
		9591/3	Malignant lymphoma, non-Hodgkin
		9596/3	Composite Hodgkin and non-Hodgkin lymphoma
HODGKIN LYMPHOMA	965	9650/3	Hodgkin lymphoma, NOS
		9651/3	Hodgkin lymphoma, lymphocyte-rich
		9652/3	Hodgkin lymphoma, mixed cellularity, NOS
		9653/3	Hodgkin lymphoma, lymphocytic deplet., NOS
		9654/3	Hodgkin lymph., lymphocyt. deplet., diffuse fibrosis
		9655/3	Hodgkin lymphoma, lymphocyt. deplet., reticular
		9659/3	Hodgkin lymph., nodular lymphocyte predom.
HODGKIN LYMPHOMA, NOD. SCLER.	966	9661/3	Hodgkin granuloma [obs]
		9662/3	Hodgkin sarcoma [obs]
		9663/3	Hodgkin lymphoma, nodular sclerosis, NOS
		9664/3	Hodgkin lymphoma, nod. scler., cellular phase
		9665/3	Hodgkin lymphoma, nod. scler., grade 1
		9667/3	Hodgkin lymphoma, nod. scler., grade 2
ML, SMALL B-CELL LYMPHOCYTIC	967	9670/3	ML, small B lymphocytic, NOS
		9671/3	ML, lymphoplasmacytic
		9673/3	Mantle cell lymphoma
		9675/3	ML, mixed sm. and lg. cell, diffuse
ML, LARGE B-CELL, DIFFUSE	968	9680/3	ML, large B-cell, diffuse
		9684/3	ML, large B-cell, diffuse, immunoblastic, NOS
		9687/3	Burkitt lymphoma, NOS
		9688/3	T-cell histiocyte rich large B-cell lymphoma
FOLLIC. & MARGINAL LYMPH, NOS	969	9690/3	Follicular lymphoma, NOS
		9691/3	Follicular lymphoma, grade 2
		9695/3	Follicular lymphoma, grade 1
		9698/3	Follicular lymphoma, grade 3
		9699/3	Marginal zone B-cell lymphoma, NOS
T-CELL LYMPHOMAS	970	9701/3	Sezary syndrome
		9702/3	Mature T-cell lymphoma, NOS
		9705/3	Angioimmunoblastic T-cell lymphoma
OTHER SPEC. NON-HODGKIN LYMPHOMA	971	9712/3	Intravascular large B-cell lymphoma
		9714/3	Anaplastic large cell lymphoma, T-cell and Null cell type
		9719/3	NK/T-cell lymphoma, nasal and nasal-type

OROPHARNYX C090-C091,C098-C104,C108-C109

PRECURS. CELL LYMPHOBLASTIC LYMPH.	972	9724/3	SystemicEBV pos. T-cell lymphoproliferative disease of childhood
		9727/3	Precursor cell lymphoblastic lymphoma, NOS
		9728/3	Precursor B-cell lymphoblastic lymphoma
		9729/3	Precursor T-cell lymphoblastic lymphoma
PLASMA CELL TUMORS	973	9731/3	Plasmacytoma, NOS
		9734/3	Plasmacytoma, extramedullary
		9735/3	Plasmablastic lymphoma
		9737/3	ALK positive large B-cell lymphoma
		9738/3	Lrg B-cell lymphoma in HHV8-assoc. multicentric Castleman DZ
MAST CELL TUMORS	974	9740/3	Mast cell sarcoma
		9741/3	Malignant mastocytosis
NEOPLASMS OF HISTIOCYTES AND ACCESSORY LYMPHOID CELLS	975	9750/3	Malignant histiocytosis
		9751/3	Langerhans cell histiocytosis, NOS
		9754/3	Langerhans cell histiocytosis, disseminated
		9755/3	Histiocytic sarcoma
		9756/3	Langerhans cell sarcoma
		9757/3	Interdigitating dendritic cell sarcoma
		9758/3	Follicular dendritic cell sarcoma
		9759/3	Fibroblastic reticular cell tumor
PRECURSOR LYMPHOID NEOPLASMS	981	9811/3	B lymphoblastic leukemia/lymphoma, NOS
		9812/3	Leukemia/lymphoma with t(9;22)(q34;q11.2);BCR-ABL1
		9813/3	Leukemia/lymphoma with t(v;11q23);MLL rearranged
		9814/3	Leukemia/lymphoma with t(12;21)(p13;q22);TEL-AML1(ETV6-RUNX1)
		9815/3	B lymphoblastic leukemia/lymphoma with hyperdiploidy
		9816/3	Leukemia/lymphoma with hypodiploidy (hypodiploid ALL)
		9817/3	B lymphblastic leukemia/lymphoma with t(5;14)(q31;q32);IL3-IGH
		9818/3	Leukemia/lymphoma with t(1;19)(q23;p13.3); E2A PBX1 (TCF3 PBX1)
LYMPHOID LEUKEMIA, NOS	982	9823/3	Chronic lymphocytic leukemia/small lymphocytic lymphoma
PROLYMPH/PRECURS LEUKEMIA	983	9831/3	T-cell large granular lymphocytic leukemia
		9837/3	T lymphoblastic leukemia/lymphoma
CHRONIC MYELOPROLIFERATIVE DIS.	996	9965/3	Myeloid and lymphoid neoplasms with PDGFRB rearrangement
		9967/3	Myeloid and lymphoid neoplasm with FGFR1 abnormalities
MYELOPLASTIC/MYELOPROLIFERATIVE NEOPLASMS	997	9971/3	Polymorphic PTLD
		9975/3	Myelodysplastic/Myeloproliferative neoplasm, unclassifiable

NASOPHARYNX(EXCL POSTERIOR WALL) C110,C112-C113,C118-C119

NEOPLASM	800	8000/3	Neoplasm, malignant
		8001/3	Tumor cells, malignant
		8002/3	Malignant tumor, small cell type
		8003/3	Malignant tumor, giant cell type
		8004/3	Malignant tumor, spindle cell type
		8005/3	Malignant tumor, clear cell type
CARCINOMA, NOS	801	8010/2	Carcinoma in situ, NOS
		8010/3	Carcinoma, NOS
		8011/3	Epithelioma, malignant
		8012/3	Large cell carcinoma, NOS
		8013/3	Large cell neuroendocrine carcinoma
		8014/3	Large cell carcinoma with rhabdoid phenotype
		8015/3	Glassy cell carcinoma
CARCINOMA, UNDIFF., NOS	802	8020/3	Carcinoma, undifferentiated type, NOS
		8021/3	Carcinoma, anaplastic type, NOS
		8022/3	Pleomorphic carcinoma
GIANT & SPINDLE CELL CARCINOMA	803	8030/3	Giant cell and spindle cell carcinoma
		8031/3	Giant cell carcinoma
		8032/3	Spindle cell carcinoma
		8033/3	Pseudosarcomatous carcinoma
		8034/3	Polygonal cell carcinoma
		8035/3	Carcinoma with osteoclast-like giant cells
SMALL CELL CARCINOMA, NOS	804	8041/3	Small cell carcinoma, NOS
		8043/3	Small cell carcinoma, fusiform cell
PAPILLARY CARCINOMA, NOS	805	8050/2	Papillary carcinoma in situ
		8050/3	Papillary carcinoma, NOS
		8051/3	Verrucous carcinoma, NOS
		8052/2	Papillary squamous cell carcinoma, non-invasive
		8052/3	Papillary squamous cell carcinoma
SQUAMOUS CELL CARCINOMA, NOS	807	8070/2	Squamous cell carcinoma in situ, NOS
		8070/3	Squamous cell carcinoma, NOS
		8071/3	Sq. cell carcinoma, keratinizing, NOS
		8072/3	Sq. cell carcinoma, lg. cell, non-ker.
		8073/3	Sq. cell carcinoma, sm. cell, non-ker.
		8074/3	Sq. cell carcinoma, spindle cell
		8075/3	Squamous cell carcinoma, adenoid
		8076/2	Sq. cell carc. in situ with question. stromal invas.
		8076/3	Sq. cell carcinoma, micro-invasive
		8078/3	Squamous cell carcinoma with horn formation

NASOPHARYNX(EXCL POSTERIOR WALL) C110,C112-C113,C118-C119

LYMPHOEPITHELIAL CARCINOMA	808	8081/2	Bowen disease
		8082/3	Lymphoepithelial carcinoma
		8083/3	Basaloid squamous cell carcinoma
		8084/3	Squamous cell carcinoma, clear cell type
TRANSITIONAL CELL CARCINOMA, NOS	812	8120/2	Transitional cell carcinoma in situ
		8120/3	Transitional cell carcinoma, NOS
		8121/3	Schneiderian carcinoma
		8122/3	Trans. cell carcinoma, spindle cell
		8123/3	Basaloid carcinoma
		8124/3	Cloacogenic carcinoma
ADENOCARCINOMA, NOS	814	8140/2	Adenocarcinoma in situ
		8140/3	Adenocarcinoma, NOS
		8141/3	Scirrhous adenocarcinoma
		8143/3	Superficial spreading adenocarcinoma
		8147/3	Basal cell adenocarcinoma
ADENOID CYSTIC & CRIBRIFORM CA.	820	8200/3	Adenoid cystic carcinoma
		8201/2	Cribriform carcinoma in situ
		8201/3	Cribriform carcinoma
BRONCHIOLO-ALVEOLAR ADENOCA.	825	8255/3	Adenocarcinoma with mixed subtypes
PAPILLARY ADENOCARCINOMA, NOS	826	8260/3	Papillary adenocarcinoma, NOS
		8261/2	Adenocarcinoma in situ in villous adenoma
		8261/3	Adenocarcinoma in villous adenoma
		8262/3	Villous adenocarcinoma
		8263/2	Adenocarcinoma in situ in tubulovillous adenoma
		8263/3	Adenocarcinoma in tubulovillous adenoma
CLEAR CELL ADENOCARCINOMA, NOS	831	8310/3	Clear cell adenocarcinoma, NOS
MUCOEPIDERMOID CARCINOMA	843	8430/3	Mucoepidermoid carcinoma
MUCINOUS ADENOCARCINOMA	848	8480/3	Mucinous adenocarcinoma
		8481/3	Mucin-producing adenocarcinoma
NEVI & MELANOMAS	872	8720/2	Melanoma in situ
		8720/3	Malignant melanoma, NOS
		8721/3	Nodular melanoma
		8722/3	Balloon cell melanoma
		8723/3	Malignant melanoma, regressing
AMELANOTIC MELANOMA	873	8730/3	Amelanotic melanoma

NASOPHARYNX(EXCL POSTERIOR WALL) C110,C112-C113,C118-C119

MAL. MEL. IN JUNCT. NEVUS	874	8743/3	Superficial spreading melanoma
		8745/3	Desmoplastic melanoma, malignant
		8746/3	Mucosal lentiginous melanoma
EPITHELIOID CELL MELANOMA	877	8770/3	Mixed epithel. & spindle cell melanoma
		8771/3	Epithelioid cell melanoma
		8772/3	Spindle cell melanoma, NOS
SARCOMA, NOS	880	8800/3	Sarcoma, NOS
		8801/3	Spindle cell sarcoma
		8802/3	Giant cell sarcoma
		8803/3	Small cell sarcoma
		8804/3	Epithelioid sarcoma
		8805/3	Undifferentiated sarcoma
		8806/3	Desmoplastic small round cell tumor
FIBROMATOUS NEOPLASMS	881	8810/3	Fibrosarcoma, NOS
		8811/3	Fibromyxosarcoma
		8813/3	Fascial fibrosarcoma
		8814/3	Infantile fibrosarcoma
		8815/3	Solitary fibrous tumor, malignant
SARCOMA, NOS	882	8825/3	Myofibroblastic sarcoma
RHABDOMYOSARCOMA, NOS	890	8900/3	Rhabdomyosarcoma, NOS
		8901/3	Pleomorphic rhabdomyosarcoma, adult type
		8902/3	Mixed type rhabdomyosarcoma
EMBRYONAL RHABDOMYOSARCOMA	891	8910/3	Embryonal rhabdomyosarcoma
		8912/3	Spindle cell rhabdomyosarcoma
MIXED TUMOR, MALIGNANT, NOS	894	8940/3	Mixed tumor, malignant, NOS
		8941/3	Carcinoma in pleomorphic adenoma
CARCINOSARCOMA, NOS	898	8980/3	Carcinosarcoma, NOS
		8981/3	Carcinosarcoma, embryonal type
		8982/3	Malignant myoepithelioma
EMBRYONAL CARCINOMA, NOS	907	9070/3	Embryonal carcinoma, NOS
		9071/3	Yolk sac tumor
		9072/3	Polyembryoma
KAPOSI SARCOMA	914	9140/3	Kaposi sarcoma

NASOPHARYNX(EXCL POSTERIOR WALL) C110,C112-C113,C118-C119

CHORDOMA	937	9370/3	Chordoma, NOS
		9371/3	Chondroid chordoma
		9372/3	Dedifferentiated chordoma
MALIGNANT LYMPHOMA, NOS	959	9590/3	Malignant lymphoma, NOS
		9591/3	Malignant lymphoma, non-Hodgkin
		9596/3	Composite Hodgkin and non-Hodgkin lymphoma
HODGKIN LYMPHOMA	965	9650/3	Hodgkin lymphoma, NOS
		9651/3	Hodgkin lymphoma, lymphocyte-rich
		9652/3	Hodgkin lymphoma, mixed cellularity, NOS
		9653/3	Hodgkin lymphoma, lymphocytic deplet., NOS
		9654/3	Hodgkin lymph., lymphocyt. deplet., diffuse fibrosis
		9655/3	Hodgkin lymphoma, lymphocyt. deplet., reticular
		9659/3	Hodgkin lymph., nodular lymphocyte predom.
HODGKIN LYMPHOMA, NOD. SCLER.	966	9661/3	Hodgkin granuloma [obs]
		9662/3	Hodgkin sarcoma [obs]
		9663/3	Hodgkin lymphoma, nodular sclerosis, NOS
		9664/3	Hodgkin lymphoma, nod. scler., cellular phase
		9665/3	Hodgkin lymphoma, nod. scler., grade 1
		9667/3	Hodgkin lymphoma, nod. scler., grade 2
ML, SMALL B-CELL LYMPHOCYTIC	967	9670/3	ML, small B lymphocytic, NOS
		9671/3	ML, lymphoplasmacytic
		9673/3	Mantle cell lymphoma
		9675/3	ML, mixed sm. and lg. cell, diffuse
ML, LARGE B-CELL, DIFFUSE	968	9680/3	ML, large B-cell, diffuse
		9684/3	ML, large B-cell, diffuse, immunoblastic, NOS
		9687/3	Burkitt lymphoma, NOS
		9688/3	T-cell histiocyte rich large B-cell lymphoma
FOLLIC. & MARGINAL LYMPH, NOS	969	9690/3	Follicular lymphoma, NOS
		9691/3	Follicular lymphoma, grade 2
		9695/3	Follicular lymphoma, grade 1
		9698/3	Follicular lymphoma, grade 3
		9699/3	Marginal zone B-cell lymphoma, NOS
T-CELL LYMPHOMAS	970	9701/3	Sezary syndrome
		9702/3	Mature T-cell lymphoma, NOS
		9705/3	Angioimmunoblastic T-cell lymphoma
OTHER SPEC. NON-HODGKIN LYMPHOMA	971	9714/3	Anaplastic large cell lymphoma, T-cell and Null cell type
		9719/3	NK/T-cell lymphoma, nasal and nasal-type

NASOPHARYNX(EXCL POSTERIOR WALL) C110,C112-C113,C118-C119

PRECURS. CELL LYMPHOBLASTIC LYMPH.	972	9724/3	SystemicEBV pos. T-cell lymphoproliferative disease of childhood
		9727/3	Precursor cell lymphoblastic lymphoma, NOS
		9728/3	Precursor B-cell lymphoblastic lymphoma
		9729/3	Precursor T-cell lymphoblastic lymphoma
PLASMA CELL TUMORS	973	9731/3	Plasmacytoma, NOS
		9734/3	Plasmacytoma, extramedullary
		9735/3	Plasmablastic lymphoma
		9737/3	ALK positive large B-cell lymphoma
		9738/3	Lrg B-cell lymphoma in HHV8-assoc. multicentric Castleman DZ
MAST CELL TUMORS	974	9740/3	Mast cell sarcoma
		9741/3	Malignant mastocytosis
NEOPLASMS OF HISTIOCYTES AND ACCESSORY LYMPHOID CELLS	975	9750/3	Malignant histiocytosis
		9751/3	Langerhans cell histiocytosis, NOS
		9754/3	Langerhans cell histiocytosis, disseminated
		9755/3	Histiocytic sarcoma
		9756/3	Langerhans cell sarcoma
		9757/3	Interdigitating dendritic cell sarcoma
		9758/3	Follicular dendritic cell sarcoma
		9759/3	Fibroblastic reticular cell tumor
PRECURSOR LYMPHOID NEOPLASMS	981	9811/3	B lymphoblastic leukemia/lymphoma, NOS
		9812/3	Leukemia/lymphoma with t(9;22)(q34;q11.2);BCR-ABL1
		9813/3	Leukemia/lymphoma with t(v;11q23);MLL rearranged
		9814/3	Leukemia/lymphoma with t(12;21)(p13;q22);TEL-AML1(ETV6-RUNX1)
		9815/3	B lymphoblastic leukemia/lymphoma with hyperdiploidy
		9816/3	Leukemia/lymphoma with hypodiploidy (hypodiploid ALL)
		9817/3	B lymphblastic leukemia/lymphoma with t(5;14)(q31;q32);IL3-IGH
		9818/3	Leukemia/lymphoma with t(1;19)(q23;p13.3); E2A PBX1 (TCF3 PBX1)
LYMPHOID LEUKEMIA, NOS	982	9823/3	Chronic lymphocytic leukemia/small lymphocytic lymphoma
PROLYMPH/PRECURS LEUKEMIA	983	9831/3	T-cell large granular lymphocytic leukemia
		9837/3	T lymphoblastic leukemia/lymphoma
CHRONIC MYELOPROLIFERATIVE DIS.	996	9965/3	Myeloid and lymphoid neoplasms with PDGFRB rearrangement
		9967/3	Myeloid and lymphoid neoplasm with FGFR1 abnormalities
MYELOPLASTIC/MYELOPROLIFERATIVE NEOPLASMS	997	9971/3	Polymorphic PTLD
		9975/3	Myelodysplastic/Myeloproliferative neoplasm, unclassifiable

POSTERIOR WALL OF NASOPHARYNX C111

NEOPLASM	800	8000/3	Neoplasm, malignant
		8001/3	Tumor cells, malignant
		8002/3	Malignant tumor, small cell type
		8003/3	Malignant tumor, giant cell type
		8004/3	Malignant tumor, spindle cell type
		8005/3	Malignant tumor, clear cell type
CARCINOMA, NOS	801	8010/2	Carcinoma in situ, NOS
		8010/3	Carcinoma, NOS
		8011/3	Epithelioma, malignant
		8012/3	Large cell carcinoma, NOS
		8013/3	Large cell neuroendocrine carcinoma
		8014/3	Large cell carcinoma with rhabdoid phenotype
		8015/3	Glassy cell carcinoma
CARCINOMA, UNDIFF., NOS	802	8020/3	Carcinoma, undifferentiated type, NOS
		8021/3	Carcinoma, anaplastic type, NOS
		8022/3	Pleomorphic carcinoma
GIANT & SPINDLE CELL CARCINOMA	803	8030/3	Giant cell and spindle cell carcinoma
		8031/3	Giant cell carcinoma
		8032/3	Spindle cell carcinoma
		8033/3	Pseudosarcomatous carcinoma
		8034/3	Polygonal cell carcinoma
		8035/3	Carcinoma with osteoclast-like giant cells
SMALL CELL CARCINOMA, NOS	804	8041/3	Small cell carcinoma, NOS
		8043/3	Small cell carcinoma, fusiform cell
PAPILLARY CARCINOMA, NOS	805	8050/2	Papillary carcinoma in situ
		8050/3	Papillary carcinoma, NOS
		8051/3	Verrucous carcinoma, NOS
		8052/2	Papillary squamous cell carcinoma, non-invasive
		8052/3	Papillary squamous cell carcinoma
SQUAMOUS CELL CARCINOMA, NOS	807	8070/2	Squamous cell carcinoma in situ, NOS
		8070/3	Squamous cell carcinoma, NOS
		8071/3	Sq. cell carcinoma, keratinizing, NOS
		8072/3	Sq. cell carcinoma, lg. cell, non-ker.
		8073/3	Sq. cell carcinoma, sm. cell, non-ker.
		8074/3	Sq. cell carcinoma, spindle cell
		8075/3	Squamous cell carcinoma, adenoid
		8076/2	Sq. cell carc. in situ with question. stromal invas.
		8076/3	Sq. cell carcinoma, micro-invasive
		8078/3	Squamous cell carcinoma with horn formation

POSTERIOR WALL OF NASOPHARYNX C111

LYMPHOEPITHELIAL CARCINOMA	808	8081/2	Bowen disease
		8082/3	Lymphoepithelial carcinoma
		8083/3	Basaloid squamous cell carcinoma
		8084/3	Squamous cell carcinoma, clear cell type
		8085/3	Squamous cell carcinoma, HPV-positive
		8086/3	Squamous cell carcinoma, HPV-negative
TRANSITIONAL CELL CARCINOMA, NOS	812	8120/2	Transitional cell carcinoma in situ
		8120/3	Transitional cell carcinoma, NOS
		8121/3	Schneiderian carcinoma
		8122/3	Trans. cell carcinoma, spindle cell
		8123/3	Basaloid carcinoma
		8124/3	Cloacogenic carcinoma
ADENOCARCINOMA, NOS	814	8140/2	Adenocarcinoma in situ
		8140/3	Adenocarcinoma, NOS
		8141/3	Scirrhous adenocarcinoma
		8143/3	Superficial spreading adenocarcinoma
		8147/3	Basal cell adenocarcinoma
ADENOID CYSTIC & CRIBRIFORM CA.	820	8200/3	Adenoid cystic carcinoma
		8201/2	Cribriform carcinoma in situ
		8201/3	Cribriform carcinoma
BRONCHIOLO-ALVEOLAR ADENOCA.	825	8255/3	Adenocarcinoma with mixed subtypes
PAPILLARY ADENOCARCINOMA, NOS	826	8260/3	Papillary adenocarcinoma, NOS
		8261/2	Adenocarcinoma in situ in villous adenoma
		8261/3	Adenocarcinoma in villous adenoma
		8262/3	Villous adenocarcinoma
		8263/2	Adenocarcinoma in situ in tubulovillous adenoma
		8263/3	Adenocarcinoma in tubulovillous adenoma
CLEAR CELL ADENOCARCINOMA, NOS	831	8310/3	Clear cell adenocarcinoma, NOS
MUCOEPIDERMOID CARCINOMA	843	8430/3	Mucoepidermoid carcinoma
MUCINOUS ADENOCARCINOMA	848	8480/3	Mucinous adenocarcinoma
		8481/3	Mucin-producing adenocarcinoma
NEVI & MELANOMAS	872	8720/2	Melanoma in situ
		8720/3	Malignant melanoma, NOS
		8721/3	Nodular melanoma
		8722/3	Balloon cell melanoma
		8723/3	Malignant melanoma, regressing
AMELANOTIC MELANOMA	873	8730/3	Amelanotic melanoma

POSTERIOR WALL OF NASOPHARYNX C111

MAL. MEL. IN JUNCT. NEVUS	874	8743/3	Superficial spreading melanoma
		8745/3	Desmoplastic melanoma, malignant
		8746/3	Mucosal lentiginous melanoma
EPITHELIOID CELL MELANOMA	877	8770/3	Mixed epithel. & spindle cell melanoma
		8771/3	Epithelioid cell melanoma
		8772/3	Spindle cell melanoma, NOS
SARCOMA, NOS	880	8800/3	Sarcoma, NOS
		8801/3	Spindle cell sarcoma
		8802/3	Giant cell sarcoma
		8803/3	Small cell sarcoma
		8804/3	Epithelioid sarcoma
		8805/3	Undifferentiated sarcoma
		8806/3	Desmoplastic small round cell tumor
FIBROMATOUS NEOPLASMS	881	8810/3	Fibrosarcoma, NOS
		8811/3	Fibromyxosarcoma
		8813/3	Fascial fibrosarcoma
		8814/3	Infantile fibrosarcoma
		8815/3	Solitary fibrous tumor, malignant
SARCOMA, NOS	882	8825/3	Myofibroblastic sarcoma
RHABDOMYOSARCOMA, NOS	890	8900/3	Rhabdomyosarcoma, NOS
		8901/3	Pleomorphic rhabdomyosarcoma, adult type
		8902/3	Mixed type rhabdomyosarcoma
EMBRYONAL RHABDOMYOSARCOMA	891	8910/3	Embryonal rhabdomyosarcoma
		8912/3	Spindle cell rhabdomyosarcoma
MIXED TUMOR, MALIGNANT, NOS	894	8940/3	Mixed tumor, malignant, NOS
		8941/3	Carcinoma in pleomorphic adenoma
CARCINOSARCOMA, NOS	898	8980/3	Carcinosarcoma, NOS
		8981/3	Carcinosarcoma, embryonal type
		8982/3	Malignant myoepithelioma
EMBRYONAL CARCINOMA, NOS	907	9070/3	Embryonal carcinoma, NOS
		9071/3	Yolk sac tumor
		9072/3	Polyembryoma
KAPOSI SARCOMA	914	9140/3	Kaposi sarcoma

POSTERIOR WALL OF NASOPHARYNX C111

CHORDOMA	937	9370/3	Chordoma, NOS
		9371/3	Chondroid chordoma
		9372/3	Dedifferentiated chordoma
MALIGNANT LYMPHOMA, NOS	959	9590/3	Malignant lymphoma, NOS
		9591/3	Malignant lymphoma, non-Hodgkin
		9596/3	Composite Hodgkin and non-Hodgkin lymphoma
HODGKIN LYMPHOMA	965	9650/3	Hodgkin lymphoma, NOS
		9651/3	Hodgkin lymphoma, lymphocyte-rich
		9652/3	Hodgkin lymphoma, mixed cellularity, NOS
		9653/3	Hodgkin lymphoma, lymphocytic deplet., NOS
		9654/3	Hodgkin lymph., lymphocyt. deplet., diffuse fibrosis
		9655/3	Hodgkin lymphoma, lymphocyt. deplet., reticular
		9659/3	Hodgkin lymph., nodular lymphocyte predom.
HODGKIN LYMPHOMA, NOD. SCLER.	966	9661/3	Hodgkin granuloma [obs]
		9662/3	Hodgkin sarcoma [obs]
		9663/3	Hodgkin lymphoma, nodular sclerosis, NOS
		9664/3	Hodgkin lymphoma, nod. scler., cellular phase
		9665/3	Hodgkin lymphoma, nod. scler., grade 1
		9667/3	Hodgkin lymphoma, nod. scler., grade 2
ML, SMALL B-CELL LYMPHOCYTIC	967	9670/3	ML, small B lymphocytic, NOS
		9671/3	ML, lymphoplasmacytic
		9673/3	Mantle cell lymphoma
		9675/3	ML, mixed sm. and lg. cell, diffuse
ML, LARGE B-CELL, DIFFUSE	968	9680/3	ML, large B-cell, diffuse
		9684/3	ML, large B-cell, diffuse, immunoblastic, NOS
		9687/3	Burkitt lymphoma, NOS
		9688/3	T-cell histiocyte rich large B-cell lymphoma
FOLLIC. & MARGINAL LYMPH, NOS	969	9690/3	Follicular lymphoma, NOS
		9691/3	Follicular lymphoma, grade 2
		9695/3	Follicular lymphoma, grade 1
		9698/3	Follicular lymphoma, grade 3
		9699/3	Marginal zone B-cell lymphoma, NOS
T-CELL LYMPHOMAS	970	9701/3	Sezary syndrome
		9702/3	Mature T-cell lymphoma, NOS
		9705/3	Angioimmunoblastic T-cell lymphoma
OTHER SPEC. NON-HODGKIN LYMPHOMA	971	9714/3	Anaplastic large cell lymphoma, T-cell and Null cell type
		9719/3	NK/T-cell lymphoma, nasal and nasal-type

POSTERIOR WALL OF NASOPHARYNX C111

PRECURS. CELL LYMPHOBLASTIC LYMPH.	972	9724/3	SystemicEBV pos. T-cell lymphoproliferative disease of childhood
		9727/3	Precursor cell lymphoblastic lymphoma, NOS
		9728/3	Precursor B-cell lymphoblastic lymphoma
		9729/3	Precursor T-cell lymphoblastic lymphoma
PLASMA CELL TUMORS	973	9731/3	Plasmacytoma, NOS
		9734/3	Plasmacytoma, extramedullary
		9735/3	Plasmablastic lymphoma
		9737/3	ALK positive large B-cell lymphoma
		9738/3	Lrg B-cell lymphoma in HHV8-assoc. multicentric Castleman DZ
MAST CELL TUMORS	974	9740/3	Mast cell sarcoma
		9741/3	Malignant mastocytosis
NEOPLASMS OF HISTIOCYTES AND ACCESSORY LYMPHOID CELLS	975	9750/3	Malignant histiocytosis
		9751/3	Langerhans cell histiocytosis, NOS
		9754/3	Langerhans cell histiocytosis, disseminated
		9755/3	Histiocytic sarcoma
		9756/3	Langerhans cell sarcoma
		9757/3	Interdigitating dendritic cell sarcoma
		9758/3	Follicular dendritic cell sarcoma
		9759/3	Fibroblastic reticular cell tumor
PRECURSOR LYMPHOID NEOPLASMS	981	9811/3	B lymphoblastic leukemia/lymphoma, NOS
		9812/3	Leukemia/lymphoma with t(9;22)(q34;q11.2);BCR-ABL1
		9813/3	Leukemia/lymphoma with t(v;11q23);MLL rearranged
		9814/3	Leukemia/lymphoma with t(12;21)(p13;q22);TEL-AML1(ETV6-RUNX1)
		9815/3	B lymphoblastic leukemia/lymphoma with hyperdiploidy
		9816/3	Leukemia/lymphoma with hypodiploidy (hypodiploid ALL)
		9817/3	B lymphblastic leukemia/lymphoma with t(5;14)(q31;q32);IL3-IGH
		9818/3	Leukemia/lymphoma with t(1;19)(q23;p13.3); E2A PBX1 (TCF3 PBX1)
LYMPHOID LEUKEMIA, NOS	982	9823/3	Chronic lymphocytic leukemia/small lymphocytic lymphoma
PROLYMPH/PRECURS LEUKEMIA	983	9831/3	T-cell large granular lymphocytic leukemia
		9837/3	T lymphoblastic leukemia/lymphoma
CHRONIC MYELOPROLIFERATIVE DIS.	996	9965/3	Myeloid and lymphoid neoplasms with PDGFRB rearrangement
		9967/3	Myeloid and lymphoid neoplasm with FGFR1 abnormalities
MYELOPLASTIC/MYELOPROLIFERATIVE NEOPLASMS	997	9971/3	Polymorphic PTLD
		9975/3	Myelodysplastic/Myeloproliferative neoplasm, unclassifiable

HYPOPHARYNX C129-C132,C138-C139

NEOPLASM	800	8000/3	Neoplasm, malignant
		8001/3	Tumor cells, malignant
		8002/3	Malignant tumor, small cell type
		8003/3	Malignant tumor, giant cell type
		8004/3	Malignant tumor, spindle cell type
		8005/3	Malignant tumor, clear cell type
CARCINOMA, NOS	801	8010/2	Carcinoma in situ, NOS
		8010/3	Carcinoma, NOS
		8011/3	Epithelioma, malignant
		8012/3	Large cell carcinoma, NOS
		8013/3	Large cell neuroendocrine carcinoma
		8014/3	Large cell carcinoma with rhabdoid phenotype
		8015/3	Glassy cell carcinoma
CARCINOMA, UNDIFF., NOS	802	8020/3	Carcinoma, undifferentiated type, NOS
		8021/3	Carcinoma, anaplastic type, NOS
		8022/3	Pleomorphic carcinoma
PAPILLARY CARCINOMA, NOS	805	8050/2	Papillary carcinoma in situ
		8050/3	Papillary carcinoma, NOS
		8051/3	Verrucous carcinoma, NOS
		8052/2	Papillary squamous cell carcinoma, non-invasive
		8052/3	Papillary squamous cell carcinoma
SQUAMOUS CELL CARCINOMA, NOS	807	8070/2	Squamous cell carcinoma in situ, NOS
		8070/3	Squamous cell carcinoma, NOS
		8071/3	Sq. cell carcinoma, keratinizing, NOS
		8072/3	Sq. cell carcinoma, lg. cell, non-ker.
		8073/3	Sq. cell carcinoma, sm. cell, non-ker.
		8074/3	Sq. cell carcinoma, spindle cell
		8075/3	Squamous cell carcinoma, adenoid
		8076/2	Sq. cell carc. in situ with question. stromal invas.
		8076/3	Sq. cell carcinoma, micro-invasive
		8078/3	Squamous cell carcinoma with horn formation
LYMPHOEPITHELIAL CARCINOMA	808	8081/2	Bowen disease
		8082/3	Lymphoepithelial carcinoma
		8083/3	Basaloid squamous cell carcinoma
		8084/3	Squamous cell carcinoma, clear cell type
TRANSITIONAL CELL CARCINOMA, NOS	812	8120/2	Transitional cell carcinoma in situ
		8120/3	Transitional cell carcinoma, NOS
		8121/3	Schneiderian carcinoma
		8122/3	Trans. cell carcinoma, spindle cell
		8123/3	Basaloid carcinoma
		8124/3	Cloacogenic carcinoma

HYPOPHARYNX C129-C132,C138-C139

ADENOCARCINOMA, NOS	814	8140/2	Adenocarcinoma in situ
		8140/3	Adenocarcinoma, NOS
		8141/3	Scirrhous adenocarcinoma
		8143/3	Superficial spreading adenocarcinoma
		8147/3	Basal cell adenocarcinoma
ADENOID CYSTIC & CRIBRIFORM CA.	820	8200/3	Adenoid cystic carcinoma
		8201/2	Cribriform carcinoma in situ
		8201/3	Cribriform carcinoma
BRONCHIOLO-ALVEOLAR ADENOCA.	825	8255/3	Adenocarcinoma with mixed subtypes
MUCOEPIDERMOID CARCINOMA	843	8430/3	Mucoepidermoid carcinoma
FIBROMATOUS NEOPLASMS	881	8810/3	Fibrosarcoma, NOS
		8811/3	Fibromyxosarcoma
		8813/3	Fascial fibrosarcoma
		8814/3	Infantile fibrosarcoma
		8815/3	Solitary fibrous tumor, malignant
SARCOMA, NOS	882	8825/3	Myofibroblastic sarcoma
MIXED TUMOR, MALIGNANT, NOS	894	8940/3	Mixed tumor, malignant, NOS
		8941/3	Carcinoma in pleomorphic adenoma
CARCINOSARCOMA, NOS	898	8980/3	Carcinosarcoma, NOS
		8981/3	Carcinosarcoma, embryonal type
		8982/3	Malignant myoepithelioma
KAPOSI SARCOMA	914	9140/3	Kaposi sarcoma
MALIGNANT LYMPHOMA, NOS	959	9590/3	Malignant lymphoma, NOS
		9591/3	Malignant lymphoma, non-Hodgkin
		9596/3	Composite Hodgkin and non-Hodgkin lymphoma
HODGKIN LYMPHOMA	965	9650/3	Hodgkin lymphoma, NOS
		9651/3	Hodgkin lymphoma, lymphocyte-rich
		9652/3	Hodgkin lymphoma, mixed cellularity, NOS
		9653/3	Hodgkin lymphoma, lymphocytic deplet., NOS
		9654/3	Hodgkin lymph., lymphocyt. deplet., diffuse fibrosis
		9655/3	Hodgkin lymphoma, lymphocyt. deplet., reticular
		9659/3	Hodgkin lymph., nodular lymphocyte predom.

HYPOPHARYNX C129-C132,C138-C139

HODGKIN LYMPHOMA, NOD. SCLER.	966	9661/3	Hodgkin granuloma [obs]
		9662/3	Hodgkin sarcoma [obs]
		9663/3	Hodgkin lymphoma, nodular sclerosis, NOS
		9664/3	Hodgkin lymphoma, nod. scler., cellular phase
		9665/3	Hodgkin lymphoma, nod. scler., grade 1
		9667/3	Hodgkin lymphoma, nod. scler., grade 2
ML, SMALL B-CELL LYMPHOCYTIC	967	9670/3	ML, small B lymphocytic, NOS
		9671/3	ML, lymphoplasmacytic
		9673/3	Mantle cell lymphoma
		9675/3	ML, mixed sm. and lg. cell, diffuse
ML, LARGE B-CELL, DIFFUSE	968	9680/3	ML, large B-cell, diffuse
		9684/3	ML, large B-cell, diffuse, immunoblastic, NOS
		9687/3	Burkitt lymphoma, NOS
		9688/3	T-cell histiocyte rich large B-cell lymphoma
FOLLIC. & MARGINAL LYMPH, NOS	969	9690/3	Follicular lymphoma, NOS
		9691/3	Follicular lymphoma, grade 2
		9695/3	Follicular lymphoma, grade 1
		9698/3	Follicular lymphoma, grade 3
		9699/3	Marginal zone B-cell lymphoma, NOS
T-CELL LYMPHOMAS	970	9701/3	Sezary syndrome
		9702/3	Mature T-cell lymphoma, NOS
		9705/3	Angioimmunoblastic T-cell lymphoma
OTHER SPEC. NON-HODGKIN LYMPHOMA	971	9712/3	Intravascular large B-cell lymphoma
		9714/3	Anaplastic large cell lymphoma, T-cell and Null cell type
		9719/3	NK/T-cell lymphoma, nasal and nasal-type
PRECURS. CELL LYMPHOBLASTIC LYMPH.	972	9724/3	SystemicEBV pos. T-cell lymphoproliferative disease of childhood
		9727/3	Precursor cell lymphoblastic lymphoma, NOS
		9728/3	Precursor B-cell lymphoblastic lymphoma
		9729/3	Precursor T-cell lymphoblastic lymphoma
PLASMA CELL TUMORS	973	9731/3	Plasmacytoma, NOS
		9734/3	Plasmacytoma, extramedullary
		9735/3	Plasmablastic lymphoma
		9737/3	ALK positive large B-cell lymphoma
		9738/3	Lrg B-cell lymphoma in HHV8-assoc. multicentric Castleman DZ
MAST CELL TUMORS	974	9740/3	Mast cell sarcoma
		9741/3	Malignant mastocytosis

HYPOPHARYNX C129-C132,C138-C139

NEOPLASMS OF HISTIOCYTES AND ACCESSORY LYMPHOID CELLS	975	9750/3	Malignant histiocytosis
		9751/3	Langerhans cell histiocytosis, NOS
		9754/3	Langerhans cell histiocytosis, disseminated
		9755/3	Histiocytic sarcoma
		9756/3	Langerhans cell sarcoma
		9757/3	Interdigitating dendritic cell sarcoma
		9758/3	Follicular dendritic cell sarcoma
		9759/3	Fibroblastic reticular cell tumor
PRECURSOR LYMPHOID NEOPLASMS	981	9811/3	B lymphoblastic leukemia/lymphoma, NOS
		9812/3	Leukemia/lymphoma with t(9;22)(q34;q11.2);BCR-ABL1
		9813/3	Leukemia/lymphoma with t(v;11q23);MLL rearranged
		9814/3	Leukemia/lymphoma with t(12;21)(p13;q22);TEL-AML1(ETV6-RUNX1)
		9815/3	B lymphoblastic leukemia/lymphoma with hyperdiploidy
		9816/3	Leukemia/lymphoma with hypodiploidy (hypodiploid ALL)
		9817/3	B lymphblastic leukemia/lymphoma with t(5;14)(q31;q32);IL3-IGH
		9818/3	Leukemia/lymphoma with t(1;19)(q23;p13.3); E2A PBX1 (TCF3 PBX1)
LYMPHOID LEUKEMIA, NOS	982	9823/3	Chronic lymphocytic leukemia/small lymphocytic lymphoma
PROLYMPH/PRECURS LEUKEMIA	983	9831/3	T-cell large granular lymphocytic leukemia
		9837/3	T lymphoblastic leukemia/lymphoma
CHRONIC MYELOPROLIFERATIVE DIS.	996	9965/3	Myeloid and lymphoid neoplasms with PDGFRB rearrangement
		9967/3	Myeloid and lymphoid neoplasm with FGFR1 abnormalities
MYELOPLASTIC/MYELOPROLIFERATIVE NEOPLASMS	997	9971/3	Polymorphic PTLD
		9975/3	Myelodysplastic/Myeloproliferative neoplasm, unclassifiable

PHARYNX C140,C142,C148

NEOPLASM	800	8000/3	Neoplasm, malignant
		8001/3	Tumor cells, malignant
		8002/3	Malignant tumor, small cell type
		8003/3	Malignant tumor, giant cell type
		8004/3	Malignant tumor, spindle cell type
		8005/3	Malignant tumor, clear cell type
CARCINOMA, NOS	801	8010/2	Carcinoma in situ, NOS
		8010/3	Carcinoma, NOS
		8011/3	Epithelioma, malignant
		8012/3	Large cell carcinoma, NOS
		8013/3	Large cell neuroendocrine carcinoma
		8014/3	Large cell carcinoma with rhabdoid phenotype
		8015/3	Glassy cell carcinoma
CARCINOMA, UNDIFF., NOS	802	8020/3	Carcinoma, undifferentiated type, NOS
		8021/3	Carcinoma, anaplastic type, NOS
		8022/3	Pleomorphic carcinoma
PAPILLARY CARCINOMA, NOS	805	8050/2	Papillary carcinoma in situ
		8050/3	Papillary carcinoma, NOS
		8051/3	Verrucous carcinoma, NOS
		8052/2	Papillary squamous cell carcinoma, non-invasive
		8052/3	Papillary squamous cell carcinoma
SQUAMOUS CELL CARCINOMA, NOS	807	8070/2	Squamous cell carcinoma in situ, NOS
		8070/3	Squamous cell carcinoma, NOS
		8071/3	Sq. cell carcinoma, keratinizing, NOS
		8072/3	Sq. cell carcinoma, lg. cell, non-ker.
		8073/3	Sq. cell carcinoma, sm. cell, non-ker.
		8074/3	Sq. cell carcinoma, spindle cell
		8075/3	Squamous cell carcinoma, adenoid
		8076/2	Sq. cell carc. in situ with question. stromal invas.
		8076/3	Sq. cell carcinoma, micro-invasive
		8078/3	Squamous cell carcinoma with horn formation
LYMPHOEPITHELIAL CARCINOMA	808	8081/2	Bowen disease
		8082/3	Lymphoepithelial carcinoma
		8083/3	Basaloid squamous cell carcinoma
		8084/3	Squamous cell carcinoma, clear cell type
ADENOCARCINOMA, NOS	814	8140/2	Adenocarcinoma in situ
		8140/3	Adenocarcinoma, NOS
		8141/3	Scirrhous adenocarcinoma
		8143/3	Superficial spreading adenocarcinoma
		8147/3	Basal cell adenocarcinoma

PHARYNX C140,C142,C148

ADENOID CYSTIC & CRIBRIFORM CA.	820	8200/3	Adenoid cystic carcinoma
		8201/2	Cribriform carcinoma in situ
		8201/3	Cribriform carcinoma
BRONCHIOLO-ALVEOLAR ADENOCA.	825	8255/3	Adenocarcinoma with mixed subtypes
MUCOEPIDERMOID CARCINOMA	843	8430/3	Mucoepidermoid carcinoma
MYOMATOUS NEOPLASMS	889	8890/3	Leiomyosarcoma, NOS
		8891/3	Epithelioid leiomyosarcoma
		8894/3	Angiomyosarcoma
		8895/3	Myosarcoma
		8896/3	Myxoid leiomyosarcoma
MIXED TUMOR, MALIGNANT, NOS	894	8940/3	Mixed tumor, malignant, NOS
		8941/3	Carcinoma in pleomorphic adenoma
CARCINOSARCOMA, NOS	898	8980/3	Carcinosarcoma, NOS
		8981/3	Carcinosarcoma, embryonal type
		8982/3	Malignant myoepithelioma
CHORDOMA	937	9370/3	Chordoma, NOS
		9371/3	Chondroid chordoma
		9372/3	Dedifferentiated chordoma
MALIGNANT LYMPHOMA, NOS	959	9590/3	Malignant lymphoma, NOS
		9591/3	Malignant lymphoma, non-Hodgkin
		9596/3	Composite Hodgkin and non-Hodgkin lymphoma
HODGKIN LYMPHOMA	965	9650/3	Hodgkin lymphoma, NOS
		9651/3	Hodgkin lymphoma, lymphocyte-rich
		9652/3	Hodgkin lymphoma, mixed cellularity, NOS
		9653/3	Hodgkin lymphoma, lymphocytic deplet., NOS
		9654/3	Hodgkin lymph., lymphocyt. deplet., diffuse fibrosis
		9655/3	Hodgkin lymphoma, lymphocyt. deplet., reticular
		9659/3	Hodgkin lymph., nodular lymphocyte predom.
HODGKIN LYMPHOMA, NOD. SCLER.	966	9661/3	Hodgkin granuloma [obs]
		9662/3	Hodgkin sarcoma [obs]
		9663/3	Hodgkin lymphoma, nodular sclerosis, NOS
		9664/3	Hodgkin lymphoma, nod. scler., cellular phase
		9665/3	Hodgkin lymphoma, nod. scler., grade 1
		9667/3	Hodgkin lymphoma, nod. scler., grade 2

PHARYNX C140,C142,C148

ML, SMALL B-CELL LYMPHOCYTIC	967	9670/3	ML, small B lymphocytic, NOS
		9671/3	ML, lymphoplasmacytic
		9673/3	Mantle cell lymphoma
		9675/3	ML, mixed sm. and lg. cell, diffuse
ML, LARGE B-CELL, DIFFUSE	968	9680/3	ML, large B-cell, diffuse
		9684/3	ML, large B-cell, diffuse, immunoblastic, NOS
		9687/3	Burkitt lymphoma, NOS
		9688/3	T-cell histiocyte rich large B-cell lymphoma
FOLLIC. & MARGINAL LYMPH, NOS	969	9690/3	Follicular lymphoma, NOS
		9691/3	Follicular lymphoma, grade 2
		9695/3	Follicular lymphoma, grade 1
		9698/3	Follicular lymphoma, grade 3
		9699/3	Marginal zone B-cell lymphoma, NOS
T-CELL LYMPHOMAS	970	9701/3	Sezary syndrome
		9702/3	Mature T-cell lymphoma, NOS
		9705/3	Angioimmunoblastic T-cell lymphoma
OTHER SPEC. NON-HODGKIN LYMPHOMA	971	9712/3	Intravascular large B-cell lymphoma
		9714/3	Anaplastic large cell lymphoma, T-cell and Null cell type
		9719/3	NK/T-cell lymphoma, nasal and nasal-type
PRECURS. CELL LYMPHOBLASTIC LYMPH.	972	9724/3	SystemicEBV pos. T-cell lymphoproliferative disease of childhood
		9727/3	Precursor cell lymphoblastic lymphoma, NOS
		9728/3	Precursor B-cell lymphoblastic lymphoma
		9729/3	Precursor T-cell lymphoblastic lymphoma
PLASMA CELL TUMORS	973	9731/3	Plasmacytoma, NOS
		9734/3	Plasmacytoma, extramedullary
		9735/3	Plasmablastic lymphoma
		9737/3	ALK positive large B-cell lymphoma
		9738/3	Lrg B-cell lymphoma in HHV8-assoc. multicentric Castleman DZ
MAST CELL TUMORS	974	9740/3	Mast cell sarcoma
		9741/3	Malignant mastocytosis
NEOPLASMS OF HISTIOCYTES AND ACCESSORY LYMPHOID CELLS	975	9750/3	Malignant histiocytosis
		9751/3	Langerhans cell histiocytosis, NOS
		9754/3	Langerhans cell histiocytosis, disseminated
		9755/3	Histiocytic sarcoma
		9756/3	Langerhans cell sarcoma
		9757/3	Interdigitating dendritic cell sarcoma
		9758/3	Follicular dendritic cell sarcoma
		9759/3	Fibroblastic reticular cell tumor

PHARYNX C140,C142,C148

PRECURSOR LYMPHOID NEOPLASMS	981	9811/3	B lymphoblastic leukemia/lymphoma, NOS
		9812/3	Leukemia/lymphoma with t(9;22)(q34;q11.2);BCR-ABL1
		9813/3	Leukemia/lymphoma with t(v;11q23);MLL rearranged
		9814/3	Leukemia/lymphoma with t(12;21)(p13;q22);TEL-AML1(ETV6-RUNX1)
		9815/3	B lymphoblastic leukemia/lymphoma with hyperdiploidy
		9816/3	Leukemia/lymphoma with hypodiploidy (hypodiploid ALL)
		9817/3	B lymphblastic leukemia/lymphoma with t(5;14)(q31;q32);IL3-IGH
		9818/3	Leukemia/lymphoma with t(1;19)(q23;p13.3); E2A PBX1 (TCF3 PBX1)
LYMPHOID LEUKEMIA, NOS	982	9823/3	Chronic lymphocytic leukemia/small lymphocytic lymphoma
PROLYMPH/PRECURS LEUKEMIA	983	9831/3	T-cell large granular lymphocytic leukemia
		9837/3	T lymphoblastic leukemia/lymphoma
CHRONIC MYELOPROLIFERATIVE DIS.	996	9965/3	Myeloid and lymphoid neoplasms with PDGFRB rearrangement
		9967/3	Myeloid and lymphoid neoplasm with FGFR1 abnormalities
MYELOPLASTIC/MYELOPROLIFERATIVE NEOPLASMS	997	9971/3	Polymorphic PTLD
		9975/3	Myelodysplastic/Myeloproliferative neoplasm, unclassifiable

ESOPHAGUS C150-C155,C158-C159

NEOPLASM	800	8000/3	Neoplasm, malignant
		8001/3	Tumor cells, malignant
		8002/3	Malignant tumor, small cell type
		8003/3	Malignant tumor, giant cell type
		8004/3	Malignant tumor, spindle cell type
		8005/3	Malignant tumor, clear cell type
CARCINOMA, NOS	801	8010/2	Carcinoma in situ, NOS
		8010/3	Carcinoma, NOS
		8011/3	Epithelioma, malignant
		8012/3	Large cell carcinoma, NOS
		8013/3	Large cell neuroendocrine carcinoma
		8014/3	Large cell carcinoma with rhabdoid phenotype
		8015/3	Glassy cell carcinoma
CARCINOMA, UNDIFF., NOS	802	8020/3	Carcinoma, undifferentiated type, NOS
		8021/3	Carcinoma, anaplastic type, NOS
		8022/3	Pleomorphic carcinoma
GIANT & SPINDLE CELL CARCINOMA	803	8030/3	Giant cell and spindle cell carcinoma
		8031/3	Giant cell carcinoma
		8032/3	Spindle cell carcinoma
		8033/3	Pseudosarcomatous carcinoma
		8034/3	Polygonal cell carcinoma
		8035/3	Carcinoma with osteoclast-like giant cells
SMALL CELL CARCINOMA, NOS	804	8041/3	Small cell carcinoma, NOS
		8043/3	Small cell carcinoma, fusiform cell
PAPILLARY CARCINOMA, NOS	805	8050/2	Papillary carcinoma in situ
		8050/3	Papillary carcinoma, NOS
		8051/3	Verrucous carcinoma, NOS
		8052/2	Papillary squamous cell carcinoma, non-invasive
		8052/3	Papillary squamous cell carcinoma
SQUAMOUS CELL CARCINOMA, NOS	807	8070/2	Squamous cell carcinoma in situ, NOS
		8070/3	Squamous cell carcinoma, NOS
		8071/3	Sq. cell carcinoma, keratinizing, NOS
		8072/3	Sq. cell carcinoma, lg. cell, non-ker.
		8073/3	Sq. cell carcinoma, sm. cell, non-ker.
		8074/3	Sq. cell carcinoma, spindle cell
		8075/3	Squamous cell carcinoma, adenoid
		8076/2	Sq. cell carc. in situ with question. stromal invas.
		8076/3	Sq. cell carcinoma, micro-invasive
		8078/3	Squamous cell carcinoma with horn formation

ESOPHAGUS C150-C155,C158-C159

ADENOCARCINOMA, NOS	814	8140/2	Adenocarcinoma in situ
		8140/3	Adenocarcinoma, NOS
		8141/3	Scirrhous adenocarcinoma
		8143/3	Superficial spreading adenocarcinoma
		8145/3	Carcinoma, diffuse type
		8147/3	Basal cell adenocarcinoma
ADENOID CYSTIC & CRIBRIFORM CA.	820	8200/3	Adenoid cystic carcinoma
		8201/2	Cribriform carcinoma in situ
		8201/3	Cribriform carcinoma
BRONCHIOLO-ALVEOLAR ADENOCA.	825	8255/3	Adenocarcinoma with mixed subtypes
PAPILLARY ADENOCARCINOMA, NOS	826	8260/3	Papillary adenocarcinoma, NOS
		8261/2	Adenocarcinoma in situ in villous adenoma
		8261/3	Adenocarcinoma in villous adenoma
		8262/3	Villous adenocarcinoma
		8263/2	Adenocarcinoma in situ in tubulovillous adenoma
		8263/3	Adenocarcinoma in tubulovillous adenoma
MUCOEPIDERMOID CARCINOMA	843	8430/3	Mucoepidermoid carcinoma
MUCINOUS ADENOCARCINOMA	848	8480/3	Mucinous adenocarcinoma
		8481/3	Mucin-producing adenocarcinoma
ADENOSQUAMOUS CARCINOMA	856	8560/3	Adenosquamous carcinoma
		8562/3	Epithelial-myoepithelial carcinoma
ADENOCA. WITH METAPLASIA	857	8570/3	Adenocarcinoma with squamous metaplasia
		8571/3	Adenocarcinoma w cartilag. & oss. metaplas.
		8572/3	Adenocarcinoma with spindle cell mataplasia
		8573/3	Adenocarcinoma with apocrine metaplasia
		8574/3	Adenocarcinoma with neuroendocrine differen.
		8575/3	Metaplastic carcinoma, NOS
NEVI & MELANOMAS	872	8720/2	Melanoma in situ
		8720/3	Malignant melanoma, NOS
		8721/3	Nodular melanoma
		8722/3	Balloon cell melanoma
		8723/3	Malignant melanoma, regressing
AMELANOTIC MELANOMA	873	8730/3	Amelanotic melanoma

ESOPHAGUS C150-C155,C158-C159

MAL. MEL. IN JUNCT. NEVUS	874	8743/3	Superficial spreading melanoma
		8745/3	Desmoplastic melanoma, malignant
		8746/3	Mucosal lentiginous melanoma
EPITHELIOID CELL MELANOMA	877	8770/3	Mixed epithel. & spindle cell melanoma
		8771/3	Epithelioid cell melanoma
		8772/3	Spindle cell melanoma, NOS
MYOMATOUS NEOPLASMS	889	8890/3	Leiomyosarcoma, NOS
		8891/3	Epithelioid leiomyosarcoma
		8894/3	Angiomyosarcoma
		8895/3	Myosarcoma
		8896/3	Myxoid leiomyosarcoma
CARCINOSARCOMA, NOS	898	8980/3	Carcinosarcoma, NOS
		8981/3	Carcinosarcoma, embryonal type
		8982/3	Malignant myoepithelioma
ML, LARGE B-CELL, DIFFUSE	968	9680/3	ML, large B-cell, diffuse
		9688/3	T-cell histiocyte rich large B-cell lymphoma
FOLLIC. & MARGINAL LYMPH, NOS	969	9699/3	Marginal zone B-cell lymphoma, NOS
T-CELL LYMPHOMAS	970	9701/3	Sezary syndrome
		9702/3	Mature T-cell lymphoma, NOS
		9705/3	Angioimmunoblastic T-cell lymphoma
OTHER SPEC. NON-HODGKIN LYMPHOMA	971	9712/3	Intravascular large B-cell lymphoma
		9714/3	Anaplastic large cell lymphoma, T-cell and Null cell type
		9719/3	NK/T-cell lymphoma, nasal and nasal-type
PRECURS. CELL LYMPHOBLASTIC LYMPH.	972	9724/3	SystemicEBV pos. T-cell lymphoproliferative disease of childhood
		9727/3	Precursor cell lymphoblastic lymphoma, NOS
		9728/3	Precursor B-cell lymphoblastic lymphoma
		9729/3	Precursor T-cell lymphoblastic lymphoma
PLASMA CELL TUMORS	973	9731/3	Plasmacytoma, NOS
		9734/3	Plasmacytoma, extramedullary
		9735/3	Plasmablastic lymphoma
		9737/3	ALK positive large B-cell lymphoma
		9738/3	Lrg B-cell lymphoma in HHV8-assoc. multicentric Castleman DZ
MAST CELL TUMORS	974	9740/3	Mast cell sarcoma
		9741/3	Malignant mastocytosis

ESOPHAGUS C150-C155,C158-C159

NEOPLASMS OF HISTIOCYTES AND ACCESSORY LYMPHOID CELLS	975	9750/3	Malignant histiocytosis
		9751/3	Langerhans cell histiocytosis, NOS
		9754/3	Langerhans cell histiocytosis, disseminated
		9755/3	Histiocytic sarcoma
		9756/3	Langerhans cell sarcoma
		9757/3	Interdigitating dendritic cell sarcoma
		9758/3	Follicular dendritic cell sarcoma
		9759/3	Fibroblastic reticular cell tumor
PRECURSOR LYMPHOID NEOPLASMS	981	9811/3	B lymphoblastic leukemia/lymphoma, NOS
		9812/3	Leukemia/lymphoma with t(9;22)(q34;q11.2);BCR-ABL1
		9813/3	Leukemia/lymphoma with t(v;11q23);MLL rearranged
		9814/3	Leukemia/lymphoma with t(12;21)(p13;q22);TEL-AML1(ETV6-RUNX1)
		9815/3	B lymphoblastic leukemia/lymphoma with hyperdiploidy
		9816/3	Leukemia/lymphoma with hypodiploidy (hypodiploid ALL)
		9817/3	B lymphblastic leukemia/lymphoma with t(5;14)(q31;q32);IL3-IGH
		9818/3	Leukemia/lymphoma with t(1;19)(q23;p13.3); E2A PBX1 (TCF3 PBX1)
LYMPHOID LEUKEMIA, NOS	982	9823/3	Chronic lymphocytic leukemia/small lymphocytic lymphoma
PROLYMPH/PRECURS LEUKEMIA	983	9831/3	T-cell large granular lymphocytic leukemia
		9837/3	T lymphoblastic leukemia/lymphoma
CHRONIC MYELOPROLIFERATIVE DIS.	996	9965/3	Myeloid and lymphoid neoplasms with PDGFRB rearrangement
		9967/3	Myeloid and lymphoid neoplasm with FGFR1 abnormalities
MYELOPLASTIC/MYELOPROLIFERATIVE NEOPLASMS	997	9971/3	Polymorphic PTLD
		9975/3	Myelodysplastic/Myeloproliferative neoplasm, unclassifiable

STOMACH C160-C166,C168-C169

NEOPLASM	800	8000/3	Neoplasm, malignant
		8001/3	Tumor cells, malignant
		8002/3	Malignant tumor, small cell type
		8003/3	Malignant tumor, giant cell type
		8004/3	Malignant tumor, spindle cell type
		8005/3	Malignant tumor, clear cell type
CARCINOMA, NOS	801	8010/2	Carcinoma in situ, NOS
		8010/3	Carcinoma, NOS
		8011/3	Epithelioma, malignant
		8012/3	Large cell carcinoma, NOS
		8013/3	Large cell neuroendocrine carcinoma
		8014/3	Large cell carcinoma with rhabdoid phenotype
		8015/3	Glassy cell carcinoma
CARCINOMA, UNDIFF., NOS	802	8020/3	Carcinoma, undifferentiated type, NOS
		8021/3	Carcinoma, anaplastic type, NOS
		8022/3	Pleomorphic carcinoma
GIANT & SPINDLE CELL CARCINOMA	803	8030/3	Giant cell and spindle cell carcinoma
		8031/3	Giant cell carcinoma
		8032/3	Spindle cell carcinoma
		8033/3	Pseudosarcomatous carcinoma
		8034/3	Polygonal cell carcinoma
		8035/3	Carcinoma with osteoclast-like giant cells
SMALL CELL CARCINOMA, NOS	804	8041/3	Small cell carcinoma, NOS
		8043/3	Small cell carcinoma, fusiform cell
PAPILLARY CARCINOMA, NOS	805	8050/2	Papillary carcinoma in situ
		8050/3	Papillary carcinoma, NOS
		8051/3	Verrucous carcinoma, NOS
		8052/2	Papillary squamous cell carcinoma, non-invasive
		8052/3	Papillary squamous cell carcinoma
SQUAMOUS CELL CARCINOMA, NOS	807	8070/2	Squamous cell carcinoma in situ, NOS
		8070/3	Squamous cell carcinoma, NOS
		8071/3	Sq. cell carcinoma, keratinizing, NOS
		8072/3	Sq. cell carcinoma, lg. cell, non-ker.
		8073/3	Sq. cell carcinoma, sm. cell, non-ker.
		8074/3	Sq. cell carcinoma, spindle cell
		8075/3	Squamous cell carcinoma, adenoid
		8076/2	Sq. cell carc. in situ with question. stromal invas.
		8076/3	Sq. cell carcinoma, micro-invasive
		8078/3	Squamous cell carcinoma with horn formation

STOMACH C160-C166,C168-C169

ADENOCARCINOMA, NOS	814	8140/2	Adenocarcinoma in situ
		8140/3	Adenocarcinoma, NOS
		8141/3	Scirrhous adenocarcinoma
		8142/3	Linitis plastica
		8143/3	Superficial spreading adenocarcinoma
		8144/3	Adenocarcinoma, intestinal type
		8145/3	Carcinoma, diffuse type
		8147/3	Basal cell adenocarcinoma
ADENOCA. IN ADENOMA. POLYP	821	8210/2	Adenocarcinoma in situ in adenomatous polyp
		8210/3	Adenocarcinoma in adenomatous polyp
		8211/3	Tubular adenocarcinoma
		8214/3	Parietal cell carcinoma
ADENOCA IN FAMIL POLYP COLI	822	8220/2	Adenocarcinoma in situ in familial polyp. coli
		8220/3	Adenocarcinoma in adenoma. polyposis coli
		8221/2	Adenocarc. in situ in mult. adenomatous polyps
		8221/3	Adenocarcinoma in mult. adenomatous polyps
SOLID CARCINOMA, NOS	823	8230/2	Duct carcinoma in situ, solid type
		8230/3	Solid carcinoma, NOS
		8231/3	Carcinoma simplex
CARCINOID TUMOR, MALIGNANT	824	8240/3	Carcinoid tumor, malignant
		8241/3	Enterochromaffin cell carcinoid
		8242/3	Enterochromaffin-like cell tumor, malignant
		8243/3	Goblet cell carcinoid
		8244/3	Composite carcinoid
		8245/3	Adenocarcinoid tumor
		8246/3	Neuroendocrine carcinoma
		8249/3	Atypical carcinoid tumor
BRONCHIOLO-ALVEOLAR ADENOCA.	825	8255/3	Adenocarcinoma with mixed subtypes
PAPILLARY ADENOCARCINOMA, NOS	826	8260/3	Papillary adenocarcinoma, NOS
		8261/2	Adenocarcinoma in situ in villous adenoma
		8261/3	Adenocarcinoma in villous adenoma
		8262/3	Villous adenocarcinoma
		8263/2	Adenocarcinoma in situ in tubulovillous adenoma
		8263/3	Adenocarcinoma in tubulovillous adenoma
CLEAR CELL ADENOCARCINOMA, NOS	831	8310/3	Clear cell adenocarcinoma, NOS

STOMACH C160-C166,C168-C169

MUCINOUS ADENOCARCINOMA	848	8480/3	Mucinous adenocarcinoma
		8481/3	Mucin-producing adenocarcinoma
SIGNET RING CELL CARCINOMA	849	8490/3	Signet ring cell carcinoma
MEDULLARY CARCINOMA, NOS	851	8510/3	Medullary carcinoma, NOS
ADENOSQUAMOUS CARCINOMA	856	8560/3	Adenosquamous carcinoma
		8562/3	Epithelial-myoepithelial carcinoma
ADENOCA. WITH METAPLASIA	857	8570/3	Adenocarcinoma with squamous metaplasia
		8571/3	Adenocarcinoma w cartilag. & oss. metaplas.
		8572/3	Adenocarcinoma with spindle cell mataplasia
		8573/3	Adenocarcinoma with apocrine metaplasia
		8574/3	Adenocarcinoma with neuroendocrine differen.
		8575/3	Metaplastic carcinoma, NOS
		8576/3	Hepatoid adenocarcinoma
SARCOMA, NOS	880	8800/3	Sarcoma, NOS
		8801/3	Spindle cell sarcoma
		8802/3	Giant cell sarcoma
		8803/3	Small cell sarcoma
		8804/3	Epithelioid sarcoma
		8805/3	Undifferentiated sarcoma
		8806/3	Desmoplastic small round cell tumor
MYOMATOUS NEOPLASMS	889	8890/3	Leiomyosarcoma, NOS
		8891/3	Epithelioid leiomyosarcoma
		8894/3	Angiomyosarcoma
		8895/3	Myosarcoma
		8896/3	Myxoid leiomyosarcoma
STROMAL SARCOMA	893	8934/3	Carcinofibroma
		8935/3	Stromal sarcoma, NOS
		8936/3	Gastrointestinal stromal sarcoma
CARCINOSARCOMA, NOS	898	8980/3	Carcinosarcoma, NOS
		8981/3	Carcinosarcoma, embryonal type
		8982/3	Malignant myoepithelioma
MALIGNANT LYMPHOMA, NOS	959	9590/3	Malignant lymphoma, NOS
		9591/3	Malignant lymphoma, non-Hodgkin
		9596/3	Composite Hodgkin and non-Hodgkin lymphoma

STOMACH C160-C166,C168-C169

HODGKIN LYMPHOMA	965	9650/3	Hodgkin lymphoma, NOS
		9651/3	Hodgkin lymphoma, lymphocyte-rich
		9652/3	Hodgkin lymphoma, mixed cellularity, NOS
		9653/3	Hodgkin lymphoma, lymphocytic deplet., NOS
		9654/3	Hodgkin lymph., lymphocyt. deplet., diffuse fibrosis
		9655/3	Hodgkin lymphoma, lymphocyt. deplet., reticular
		9659/3	Hodgkin lymph., nodular lymphocyte predom.
HODGKIN LYMPHOMA, NOD. SCLER.	966	9661/3	Hodgkin granuloma [obs]
		9662/3	Hodgkin sarcoma [obs]
		9663/3	Hodgkin lymphoma, nodular sclerosis, NOS
		9664/3	Hodgkin lymphoma, nod. scler., cellular phase
		9665/3	Hodgkin lymphoma, nod. scler., grade 1
		9667/3	Hodgkin lymphoma, nod. scler., grade 2
ML, SMALL B-CELL LYMPHOCYTIC	967	9670/3	ML, small B lymphocytic, NOS
		9671/3	ML, lymphoplasmacytic
		9673/3	Mantle cell lymphoma
		9675/3	ML, mixed sm. and lg. cell, diffuse
ML, LARGE B-CELL, DIFFUSE	968	9680/3	ML, large B-cell, diffuse
		9684/3	ML, large B-cell, diffuse, immunoblastic, NOS
		9687/3	Burkitt lymphoma, NOS
		9688/3	T-cell histiocyte rich large B-cell lymphoma
FOLLIC. & MARGINAL LYMPH, NOS	969	9690/3	Follicular lymphoma, NOS
		9691/3	Follicular lymphoma, grade 2
		9695/3	Follicular lymphoma, grade 1
		9698/3	Follicular lymphoma, grade 3
		9699/3	Marginal zone B-cell lymphoma, NOS
T-CELL LYMPHOMAS	970	9701/3	Sezary syndrome
		9702/3	Mature T-cell lymphoma, NOS
		9705/3	Angioimmunoblastic T-cell lymphoma
OTHER SPEC. NON-HODGKIN LYMPHOMA	971	9712/3	Intravascular large B-cell lymphoma
		9714/3	Anaplastic large cell lymphoma, T-cell and Null cell type
		9719/3	NK/T-cell lymphoma, nasal and nasal-type
PRECURS. CELL LYMPHOBLASTIC LYMPH.	972	9724/3	SystemicEBV pos. T-cell lymphoproliferative disease of childhood
		9727/3	Precursor cell lymphoblastic lymphoma, NOS
		9728/3	Precursor B-cell lymphoblastic lymphoma
		9729/3	Precursor T-cell lymphoblastic lymphoma

STOMACH C160-C166,C168-C169

PLASMA CELL TUMORS	973	9731/3	Plasmacytoma, NOS
		9732/3	Multiple myeloma
		9734/3	Plasmacytoma, extramedullary
		9735/3	Plasmablastic lymphoma
		9737/3	ALK positive large B-cell lymphoma
		9738/3	Lrg B-cell lymphoma in HHV8-assoc. multicentric Castleman DZ
MAST CELL TUMORS	974	9740/3	Mast cell sarcoma
		9741/3	Malignant mastocytosis
NEOPLASMS OF HISTIOCYTES AND ACCESSORY LYMPHOID CELLS	975	9750/3	Malignant histiocytosis
		9751/3	Langerhans cell histiocytosis, NOS
		9754/3	Langerhans cell histiocytosis, disseminated
		9755/3	Histiocytic sarcoma
		9756/3	Langerhans cell sarcoma
		9757/3	Interdigitating dendritic cell sarcoma
		9758/3	Follicular dendritic cell sarcoma
		9759/3	Fibroblastic reticular cell tumor
PRECURSOR LYMPHOID NEOPLASMS	981	9811/3	B lymphoblastic leukemia/lymphoma, NOS
		9812/3	Leukemia/lymphoma with t(9;22)(q34;q11.2);BCR-ABL1
		9813/3	Leukemia/lymphoma with t(v;11q23);MLL rearranged
		9814/3	Leukemia/lymphoma with t(12;21)(p13;q22);TEL-AML1(ETV6-RUNX1)
		9815/3	B lymphoblastic leukemia/lymphoma with hyperdiploidy
		9816/3	Leukemia/lymphoma with hypodiploidy (hypodiploid ALL)
		9817/3	B lymphblastic leukemia/lymphoma with t(5;14)(q31;q32);IL3-IGH
		9818/3	Leukemia/lymphoma with t(1;19)(q23;p13.3); E2A PBX1 (TCF3 PBX1)
LYMPHOID LEUKEMIA, NOS	982	9823/3	Chronic lymphocytic leukemia/small lymphocytic lymphoma
PROLYMPH/PRECURS LEUKEMIA	983	9831/3	T-cell large granular lymphocytic leukemia
		9837/3	T lymphoblastic leukemia/lymphoma
CHRONIC MYELOPROLIFERATIVE DIS.	996	9965/3	Myeloid and lymphoid neoplasms with PDGFRB rearrangement
		9967/3	Myeloid and lymphoid neoplasm with FGFR1 abnormalities
MYELOPLASTIC/MYELOPROLIFERATIVE NEOPLASMS	997	9971/3	Polymorphic PTLD
		9975/3	Myelodysplastic/Myeloproliferative neoplasm, unclassifiable

SMALL INTESTINE C170-C173,C178-C179

NEOPLASM	800	8000/3	Neoplasm, malignant
		8001/3	Tumor cells, malignant
		8002/3	Malignant tumor, small cell type
		8003/3	Malignant tumor, giant cell type
		8004/3	Malignant tumor, spindle cell type
		8005/3	Malignant tumor, clear cell type
CARCINOMA, NOS	801	8010/2	Carcinoma in situ, NOS
		8010/3	Carcinoma, NOS
		8011/3	Epithelioma, malignant
		8012/3	Large cell carcinoma, NOS
		8013/3	Large cell neuroendocrine carcinoma
		8014/3	Large cell carcinoma with rhabdoid phenotype
		8015/3	Glassy cell carcinoma
CARCINOMA, UNDIFF., NOS	802	8020/3	Carcinoma, undifferentiated type, NOS
		8021/3	Carcinoma, anaplastic type, NOS
		8022/3	Pleomorphic carcinoma
SMALL CELL CARCINOMA, NOS	804	8041/3	Small cell carcinoma, NOS
		8043/3	Small cell carcinoma, fusiform cell
PAPILLARY CARCINOMA, NOS	805	8050/2	Papillary carcinoma in situ
		8050/3	Papillary carcinoma, NOS
		8051/3	Verrucous carcinoma, NOS
		8052/2	Papillary squamous cell carcinoma, non-invasive
		8052/3	Papillary squamous cell carcinoma
ADENOCARCINOMA, NOS	814	8140/2	Adenocarcinoma in situ
		8140/3	Adenocarcinoma, NOS
		8141/3	Scirrhous adenocarcinoma
		8143/3	Superficial spreading adenocarcinoma
		8145/3	Carcinoma, diffuse type
		8147/3	Basal cell adenocarcinoma
ENDOCRINOMAS	815	8152/3	Glucagonoma, malignant
		8153/3	Gastrinoma, malignant
		8156/3	Somatostatinoma, malignant
		8157/3	Enteroglucagonoma, malignant
CARCINOID TUMOR, MALIGNANT	824	8240/3	Carcinoid tumor, malignant
		8241/3	Enterochromaffin cell carcinoid
		8242/3	Enterochromaffin-like cell tumor, malignant
		8243/3	Goblet cell carcinoid
		8244/3	Composite carcinoid
		8245/3	Adenocarcinoid tumor
		8246/3	Neuroendocrine carcinoma

SMALL INTESTINE C170-C173,C178-C179

CARCINOID TUMOR, MALIGNANT	824	8249/3	Atypical carcinoid tumor
BRONCHIOLO-ALVEOLAR ADENOCA.	825	8255/3	Adenocarcinoma with mixed subtypes
PAPILLARY ADENOCARCINOMA, NOS	826	8260/3	Papillary adenocarcinoma, NOS
		8261/2	Adenocarcinoma in situ in villous adenoma
		8261/3	Adenocarcinoma in villous adenoma
		8262/3	Villous adenocarcinoma
		8263/2	Adenocarcinoma in situ in tubulovillous adenoma
		8263/3	Adenocarcinoma in tubulovillous adenoma
MUCINOUS ADENOCARCINOMA	848	8480/3	Mucinous adenocarcinoma
		8481/3	Mucin-producing adenocarcinoma
SIGNET RING CELL CARCINOMA	849	8490/3	Signet ring cell carcinoma
ADENOCA. WITH METAPLASIA	857	8570/3	Adenocarcinoma with squamous metaplasia
		8571/3	Adenocarcinoma w cartilag. & oss. metaplas.
		8572/3	Adenocarcinoma with spindle cell mataplasia
		8573/3	Adenocarcinoma with apocrine metaplasia
		8574/3	Adenocarcinoma with neuroendocrine differen.
		8575/3	Metaplastic carcinoma, NOS
		8576/3	Hepatoid adenocarcinoma
GLOMANGIOSARCOMA	871	8710/3	Glomangiosarcoma
SARCOMA, NOS	880	8800/3	Sarcoma, NOS
		8801/3	Spindle cell sarcoma
		8802/3	Giant cell sarcoma
		8803/3	Small cell sarcoma
		8804/3	Epithelioid sarcoma
		8805/3	Undifferentiated sarcoma
		8806/3	Desmoplastic small round cell tumor
FIBROMATOUS NEOPLASMS	881	8810/3	Fibrosarcoma, NOS
		8811/3	Fibromyxosarcoma
		8813/3	Fascial fibrosarcoma
		8814/3	Infantile fibrosarcoma
		8815/3	Solitary fibrous tumor, malignant
SARCOMA, NOS	882	8825/3	Myofibroblastic sarcoma
LIPOSARCOMA NEOPLASMS	885	8850/3	Liposarcoma, NOS
		8851/3	Liposarcoma, well differentiated
		8852/3	Myxoid liposarcoma
		8853/3	Round cell liposarcoma
		8854/3	Pleomorphic liposarcoma

SMALL INTESTINE C170-C173,C178-C179

LIPOSARCOMA NEOPLASMS	885	8855/3	Mixed type liposarcoma
		8857/3	Fibroblastic liposarcoma
		8858/3	Dedifferentiated liposarcoma
MYOMATOUS NEOPLASMS	889	8890/3	Leiomyosarcoma, NOS
		8891/3	Epithelioid leiomyosarcoma
		8894/3	Angiomyosarcoma
		8895/3	Myosarcoma
		8896/3	Myxoid leiomyosarcoma
STROMAL SARCOMA	893	8934/3	Carcinofibroma
		8935/3	Stromal sarcoma, NOS
		8936/3	Gastrointestinal stromal sarcoma
MESENCHYMOMA, MALIGNANT	899	8990/3	Mesenchymoma, malignant
		8991/3	Embryonal sarcoma
MALIGNANT LYMPHOMA, NOS	959	9590/3	Malignant lymphoma, NOS
		9591/3	Malignant lymphoma, non-Hodgkin
		9596/3	Composite Hodgkin and non-Hodgkin lymphoma
HODGKIN LYMPHOMA	965	9650/3	Hodgkin lymphoma, NOS
		9651/3	Hodgkin lymphoma, lymphocyte-rich
		9652/3	Hodgkin lymphoma, mixed cellularity, NOS
		9653/3	Hodgkin lymphoma, lymphocytic deplet., NOS
		9654/3	Hodgkin lymph., lymphocyt. deplet., diffuse fibrosis
		9655/3	Hodgkin lymphoma, lymphocyt. deplet., reticular
		9659/3	Hodgkin lymph., nodular lymphocyte predom.
HODGKIN LYMPHOMA, NOD. SCLER.	966	9661/3	Hodgkin granuloma [obs]
		9662/3	Hodgkin sarcoma [obs]
		9663/3	Hodgkin lymphoma, nodular sclerosis, NOS
		9664/3	Hodgkin lymphoma, nod. scler., cellular phase
		9665/3	Hodgkin lymphoma, nod. scler., grade 1
		9667/3	Hodgkin lymphoma, nod. scler., grade 2
ML, SMALL B-CELL LYMPHOCYTIC	967	9670/3	ML, small B lymphocytic, NOS
		9671/3	ML, lymphoplasmacytic
		9673/3	Mantle cell lymphoma
		9675/3	ML, mixed sm. and lg. cell, diffuse
ML, LARGE B-CELL, DIFFUSE	968	9680/3	ML, large B-cell, diffuse
		9684/3	ML, large B-cell, diffuse, immunoblastic, NOS
		9687/3	Burkitt lymphoma, NOS
		9688/3	T-cell histiocyte rich large B-cell lymphoma

SMALL INTESTINE C170-C173,C178-C179

FOLLIC. & MARGINAL LYMPH, NOS	969	9690/3	Follicular lymphoma, NOS
		9691/3	Follicular lymphoma, grade 2
		9695/3	Follicular lymphoma, grade 1
		9698/3	Follicular lymphoma, grade 3
		9699/3	Marginal zone B-cell lymphoma, NOS
T-CELL LYMPHOMAS	970	9701/3	Sezary syndrome
		9702/3	Mature T-cell lymphoma, NOS
		9705/3	Angioimmunoblastic T-cell lymphoma
OTHER SPEC. NON-HODGKIN LYMPHOMA	971	9714/3	Anaplastic large cell lymphoma, T-cell and Null cell type
		9717/3	Intestinal T-cell lymphoma
		9719/3	NK/T-cell lymphoma, nasal and nasal-type
PRECURS. CELL LYMPHOBLASTIC LYMPH.	972	9724/3	SystemicEBV pos. T-cell lymphoproliferative disease of childhood
		9727/3	Precursor cell lymphoblastic lymphoma, NOS
		9728/3	Precursor B-cell lymphoblastic lymphoma
		9729/3	Precursor T-cell lymphoblastic lymphoma
PLASMA CELL TUMORS	973	9731/3	Plasmacytoma, NOS
		9734/3	Plasmacytoma, extramedullary
		9735/3	Plasmablastic lymphoma
		9737/3	ALK positive large B-cell lymphoma
		9738/3	Lrg B-cell lymphoma in HHV8-assoc. multicentric Castleman DZ
MAST CELL TUMORS	974	9740/3	Mast cell sarcoma
		9741/3	Malignant mastocytosis
NEOPLASMS OF HISTIOCYTES AND ACCESSORY LYMPHOID CELLS	975	9750/3	Malignant histiocytosis
		9751/3	Langerhans cell histiocytosis, NOS
		9754/3	Langerhans cell histiocytosis, disseminated
		9755/3	Histiocytic sarcoma
		9756/3	Langerhans cell sarcoma
		9757/3	Interdigitating dendritic cell sarcoma
		9758/3	Follicular dendritic cell sarcoma
		9759/3	Fibroblastic reticular cell tumor
IMMUNOPROLIFERATIVE DISEASES	976	9764/3	Immunoproliferative small intestinal disease
PRECURSOR LYMPHOID NEOPLASMS	981	9811/3	B lymphoblastic leukemia/lymphoma, NOS
		9812/3	Leukemia/lymphoma with t(9;22)(q34;q11.2);BCR-ABL1
		9813/3	Leukemia/lymphoma with t(v;11q23);MLL rearranged
		9814/3	Leukemia/lymphoma with t(12;21)(p13;q22);TEL-AML1(ETV6-RUNX1)
		9815/3	B lymphoblastic leukemia/lymphoma with hyperdiploidy
		9816/3	Leukemia/lymphoma with hypodiploidy (hypodiploid ALL)
		9817/3	B lymphblastic leukemia/lymphoma with t(5;14)(q31;q32);IL3-IGH
		9818/3	Leukemia/lymphoma with t(1;19)(q23;p13.3); E2A PBX1 (TCF3 PBX1)

SMALL INTESTINE C170-C173,C178-C179

LYMPHOID LEUKEMIA, NOS	982	9823/3	Chronic lymphocytic leukemia/small lymphocytic lymphoma
PROLYMPH/PRECURS LEUKEMIA	983	9831/3	T-cell large granular lymphocytic leukemia
		9837/3	T lymphoblastic leukemia/lymphoma
CHRONIC MYELOPROLIFERATIVE DIS.	996	9965/3	Myeloid and lymphoid neoplasms with PDGFRB rearrangement
		9967/3	Myeloid and lymphoid neoplasm with FGFR1 abnormalities
MYELOPLASTIC/MYELOPROLIFERATIVE NEOPLASMS	997	9971/3	Polymorphic PTLD
		9975/3	Myelodysplastic/Myeloproliferative neoplasm, unclassifiable

LARGE INTESTINE, (EXCL. APPENDIX) C180, C182-C189, C199

NEOPLASM	800	8000/3	Neoplasm, malignant
		8001/3	Tumor cells, malignant
		8002/3	Malignant tumor, small cell type
		8003/3	Malignant tumor, giant cell type
		8004/3	Malignant tumor, spindle cell type
		8005/3	Malignant tumor, clear cell type
CARCINOMA, NOS	801	8010/2	Carcinoma in situ, NOS
		8010/3	Carcinoma, NOS
		8011/3	Epithelioma, malignant
		8012/3	Large cell carcinoma, NOS
		8013/3	Large cell neuroendocrine carcinoma
		8014/3	Large cell carcinoma with rhabdoid phenotype
		8015/3	Glassy cell carcinoma
CARCINOMA, UNDIFF., NOS	802	8020/3	Carcinoma, undifferentiated type, NOS
		8021/3	Carcinoma, anaplastic type, NOS
		8022/3	Pleomorphic carcinoma
GIANT & SPINDLE CELL CARCINOMA	803	8030/3	Giant cell and spindle cell carcinoma
		8031/3	Giant cell carcinoma
		8032/3	Spindle cell carcinoma
		8033/3	Pseudosarcomatous carcinoma
		8034/3	Polygonal cell carcinoma
		8035/3	Carcinoma with osteoclast-like giant cells
SMALL CELL CARCINOMA, NOS	804	8041/3	Small cell carcinoma, NOS
		8043/3	Small cell carcinoma, fusiform cell
PAPILLARY CARCINOMA, NOS	805	8050/2	Papillary carcinoma in situ
		8050/3	Papillary carcinoma, NOS
		8051/3	Verrucous carcinoma, NOS
		8052/2	Papillary squamous cell carcinoma, non-invasive
		8052/3	Papillary squamous cell carcinoma
SQUAMOUS CELL CARCINOMA, NOS	807	8070/2	Squamous cell carcinoma in situ, NOS
		8070/3	Squamous cell carcinoma, NOS
		8071/3	Sq. cell carcinoma, keratinizing, NOS
		8072/3	Sq. cell carcinoma, lg. cell, non-ker.
		8073/3	Sq. cell carcinoma, sm. cell, non-ker.
		8074/3	Sq. cell carcinoma, spindle cell
		8075/3	Squamous cell carcinoma, adenoid
		8076/2	Sq. cell carc. in situ with question. stromal invas.
		8076/3	Sq. cell carcinoma, micro-invasive
		8078/3	Squamous cell carcinoma with horn formation

LARGE INTESTINE, (EXCL. APPENDIX) C180, C182-C189, C199

ADENOCARCINOMA, NOS	814	8140/2	Adenocarcinoma in situ
		8140/3	Adenocarcinoma, NOS
		8141/3	Scirrhous adenocarcinoma
		8143/3	Superficial spreading adenocarcinoma
		8145/3	Carcinoma, diffuse type
		8147/3	Basal cell adenocarcinoma
ADENOCA. IN ADENOMA. POLYP	821	8210/2	Adenocarcinoma in situ in adenomatous polyp
		8210/3	Adenocarcinoma in adenomatous polyp
		8211/3	Tubular adenocarcinoma
		8213/3	Serrated adenocarcinoma
ADENOCA IN FAMIL POLYP COLI	822	8220/2	Adenocarcinoma in situ in familial polyp. coli
		8220/3	Adenocarcinoma in adenoma. polyposis coli
		8221/2	Adenocarc. in situ in mult. adenomatous polyps
		8221/3	Adenocarcinoma in mult. adenomatous polyps
SOLID CARCINOMA, NOS	823	8230/2	Duct carcinoma in situ, solid type
		8230/3	Solid carcinoma, NOS
		8231/3	Carcinoma simplex
CARCINOID TUMOR, MALIGNANT	824	8240/3	Carcinoid tumor, malignant
		8241/3	Enterochromaffin cell carcinoid
		8242/3	Enterochromaffin-like cell tumor, malignant
		8243/3	Goblet cell carcinoid
		8244/3	Composite carcinoid
		8245/3	Adenocarcinoid tumor
		8246/3	Neuroendocrine carcinoma
		8249/3	Atypical carcinoid tumor
BRONCHIOLO-ALVEOLAR ADENOCA.	825	8255/3	Adenocarcinoma with mixed subtypes
PAPILLARY ADENOCARCINOMA, NOS	826	8260/3	Papillary adenocarcinoma, NOS
		8261/2	Adenocarcinoma in situ in villous adenoma
		8261/3	Adenocarcinoma in villous adenoma
		8262/3	Villous adenocarcinoma
		8263/2	Adenocarcinoma in situ in tubulovillous adenoma
		8263/3	Adenocarcinoma in tubulovillous adenoma
		8265/3	Micropapillary carcinoma, NOS
MUCOEPIDERMOID CARCINOMA	843	8430/3	Mucoepidermoid carcinoma
CYSTADENOCARCINOMA, NOS	844	8440/3	Cystadenocarcinoma, NOS

LARGE INTESTINE, (EXCL. APPENDIX) C180, C182-C189, C199

MUCINOUS ADENOCARCINOMA	848	8480/3	Mucinous adenocarcinoma
		8481/3	Mucin-producing adenocarcinoma
SIGNET RING CELL CARCINOMA	849	8490/3	Signet ring cell carcinoma
MEDULLARY CARCINOMA, NOS	851	8510/3	Medullary carcinoma, NOS
ACINAR CELL CARCINOMA	855	8550/3	Acinar cell carcinoma
		8551/3	Acinar cell cystadenocarcinoma
ADENOSQUAMOUS CARCINOMA	856	8560/3	Adenosquamous carcinoma
		8562/3	Epithelial-myoepithelial carcinoma
ADENOCA. WITH METAPLASIA	857	8570/3	Adenocarcinoma with squamous metaplasia
		8571/3	Adenocarcinoma w cartilag. & oss. metaplas.
		8572/3	Adenocarcinoma with spindle cell mataplasia
		8573/3	Adenocarcinoma with apocrine metaplasia
		8574/3	Adenocarcinoma with neuroendocrine differen.
		8575/3	Metaplastic carcinoma, NOS
		8576/3	Hepatoid adenocarcinoma
SARCOMA, NOS	880	8800/3	Sarcoma, NOS
		8801/3	Spindle cell sarcoma
		8802/3	Giant cell sarcoma
		8803/3	Small cell sarcoma
		8804/3	Epithelioid sarcoma
		8805/3	Undifferentiated sarcoma
		8806/3	Desmoplastic small round cell tumor
FIBROMATOUS NEOPLASMS	881	8810/3	Fibrosarcoma, NOS
		8811/3	Fibromyxosarcoma
		8813/3	Fascial fibrosarcoma
		8814/3	Infantile fibrosarcoma
		8815/3	Solitary fibrous tumor, malignant
SARCOMA, NOS	882	8825/3	Myofibroblastic sarcoma
LIPOSARCOMA NEOPLASMS	885	8850/3	Liposarcoma, NOS
		8851/3	Liposarcoma, well differentiated
		8852/3	Myxoid liposarcoma
		8853/3	Round cell liposarcoma
		8854/3	Pleomorphic liposarcoma
		8855/3	Mixed type liposarcoma
		8857/3	Fibroblastic liposarcoma
		8858/3	Dedifferentiated liposarcoma

LARGE INTESTINE, (EXCL. APPENDIX) C180, C182-C189, C199

MYOMATOUS NEOPLASMS	889	8890/3	Leiomyosarcoma, NOS
		8891/3	Epithelioid leiomyosarcoma
		8894/3	Angiomyosarcoma
		8895/3	Myosarcoma
		8896/3	Myxoid leiomyosarcoma
STROMAL SARCOMA	893	8934/3	Carcinofibroma
		8935/3	Stromal sarcoma, NOS
		8936/3	Gastrointestinal stromal sarcoma
CARCINOSARCOMA, NOS	898	8980/3	Carcinosarcoma, NOS
		8981/3	Carcinosarcoma, embryonal type
		8982/3	Malignant myoepithelioma
MALIGNANT LYMPHOMA, NOS	959	9590/3	Malignant lymphoma, NOS
		9591/3	Malignant lymphoma, non-Hodgkin
		9596/3	Composite Hodgkin and non-Hodgkin lymphoma
HODGKIN LYMPHOMA	965	9650/3	Hodgkin lymphoma, NOS
		9651/3	Hodgkin lymphoma, lymphocyte-rich
		9652/3	Hodgkin lymphoma, mixed cellularity, NOS
		9653/3	Hodgkin lymphoma, lymphocytic deplet., NOS
		9654/3	Hodgkin lymph., lymphocyt. deplet., diffuse fibrosis
		9655/3	Hodgkin lymphoma, lymphocyt. deplet., reticular
		9659/3	Hodgkin lymph., nodular lymphocyte predom.
HODGKIN LYMPHOMA, NOD. SCLER.	966	9661/3	Hodgkin granuloma [obs]
		9662/3	Hodgkin sarcoma [obs]
		9663/3	Hodgkin lymphoma, nodular sclerosis, NOS
		9664/3	Hodgkin lymphoma, nod. scler., cellular phase
		9665/3	Hodgkin lymphoma, nod. scler., grade 1
		9667/3	Hodgkin lymphoma, nod. scler., grade 2
ML, SMALL B-CELL LYMPHOCYTIC	967	9670/3	ML, small B lymphocytic, NOS
		9671/3	ML, lymphoplasmacytic
		9673/3	Mantle cell lymphoma
		9675/3	ML, mixed sm. and lg. cell, diffuse
ML, LARGE B-CELL, DIFFUSE	968	9680/3	ML, large B-cell, diffuse
		9684/3	ML, large B-cell, diffuse, immunoblastic, NOS
		9687/3	Burkitt lymphoma, NOS
		9688/3	T-cell histiocyte rich large B-cell lymphoma

LARGE INTESTINE, (EXCL. APPENDIX) C180, C182-C189, C199

FOLLIC. & MARGINAL LYMPH, NOS	969	9690/3	Follicular lymphoma, NOS
		9691/3	Follicular lymphoma, grade 2
		9695/3	Follicular lymphoma, grade 1
		9698/3	Follicular lymphoma, grade 3
		9699/3	Marginal zone B-cell lymphoma, NOS
T-CELL LYMPHOMAS	970	9701/3	Sezary syndrome
		9702/3	Mature T-cell lymphoma, NOS
		9705/3	Angioimmunoblastic T-cell lymphoma
OTHER SPEC. NON-HODGKIN LYMPHOMA	971	9712/3	Intravascular large B-cell lymphoma
		9714/3	Anaplastic large cell lymphoma, T-cell and Null cell type
		9717/3	Intestinal T-cell lymphoma
		9719/3	NK/T-cell lymphoma, nasal and nasal-type
PRECURS. CELL LYMPHOBLASTIC LYMPH.	972	9724/3	SystemicEBV pos. T-cell lymphoproliferative disease of childhood
		9727/3	Precursor cell lymphoblastic lymphoma, NOS
		9728/3	Precursor B-cell lymphoblastic lymphoma
		9729/3	Precursor T-cell lymphoblastic lymphoma
PLASMA CELL TUMORS	973	9731/3	Plasmacytoma, NOS
		9734/3	Plasmacytoma, extramedullary
		9735/3	Plasmablastic lymphoma
		9737/3	ALK positive large B-cell lymphoma
		9738/3	Lrg B-cell lymphoma in HHV8-assoc. multicentric Castleman DZ
MAST CELL TUMORS	974	9740/3	Mast cell sarcoma
		9741/3	Malignant mastocytosis
NEOPLASMS OF HISTIOCYTES AND ACCESSORY LYMPHOID CELLS	975	9750/3	Malignant histiocytosis
		9751/3	Langerhans cell histiocytosis, NOS
		9754/3	Langerhans cell histiocytosis, disseminated
		9755/3	Histiocytic sarcoma
		9756/3	Langerhans cell sarcoma
		9757/3	Interdigitating dendritic cell sarcoma
		9758/3	Follicular dendritic cell sarcoma
		9759/3	Fibroblastic reticular cell tumor
PRECURSOR LYMPHOID NEOPLASMS	981	9811/3	B lymphoblastic leukemia/lymphoma, NOS
		9812/3	Leukemia/lymphoma with t(9;22)(q34;q11.2);BCR-ABL1
		9813/3	Leukemia/lymphoma with t(v;11q23);MLL rearranged
		9814/3	Leukemia/lymphoma with t(12;21)(p13;q22);TEL-AML1(ETV6-RUNX1)
		9815/3	B lymphoblastic leukemia/lymphoma with hyperdiploidy
		9816/3	Leukemia/lymphoma with hypodiploidy (hypodiploid ALL)
		9817/3	B lymphblastic leukemia/lymphoma with t(5;14)(q31;q32);IL3-IGH
		9818/3	Leukemia/lymphoma with t(1;19)(q23;p13.3); E2A PBX1 (TCF3 PBX1)

LARGE INTESTINE, (EXCL. APPENDIX) C180, C182-C189, C199

LYMPHOID LEUKEMIA, NOS	982	9823/3	Chronic lymphocytic leukemia/small lymphocytic lymphoma
PROLYMPH/PRECURS LEUKEMIA	983	9831/3	T-cell large granular lymphocytic leukemia
		9837/3	T lymphoblastic leukemia/lymphoma
CHRONIC MYELOPROLIFERATIVE DIS.	996	9965/3	Myeloid and lymphoid neoplasms with PDGFRB rearrangement
		9967/3	Myeloid and lymphoid neoplasm with FGFR1 abnormalities
MYELOPLASTIC/MYELOPROLIFERATIVE NEOPLASMS	997	9971/3	Polymorphic PTLD
		9975/3	Myelodysplastic/Myeloproliferative neoplasm, unclassifiable

APPENDIX C181

NEOPLASM	800	8000/3	Neoplasm, malignant
		8001/3	Tumor cells, malignant
		8002/3	Malignant tumor, small cell type
		8003/3	Malignant tumor, giant cell type
		8004/3	Malignant tumor, spindle cell type
		8005/3	Malignant tumor, clear cell type
CARCINOMA, NOS	801	8010/2	Carcinoma in situ, NOS
		8010/3	Carcinoma, NOS
		8011/3	Epithelioma, malignant
		8012/3	Large cell carcinoma, NOS
		8013/3	Large cell neuroendocrine carcinoma
		8014/3	Large cell carcinoma with rhabdoid phenotype
		8015/3	Glassy cell carcinoma
CARCINOMA, UNDIFF., NOS	802	8020/3	Carcinoma, undifferentiated type, NOS
		8021/3	Carcinoma, anaplastic type, NOS
		8022/3	Pleomorphic carcinoma
GIANT & SPINDLE CELL CARCINOMA	803	8030/3	Giant cell and spindle cell carcinoma
		8031/3	Giant cell carcinoma
		8032/3	Spindle cell carcinoma
		8033/3	Pseudosarcomatous carcinoma
		8034/3	Polygonal cell carcinoma
		8035/3	Carcinoma with osteoclast-like giant cells
SMALL CELL CARCINOMA, NOS	804	8041/3	Small cell carcinoma, NOS
		8043/3	Small cell carcinoma, fusiform cell
PAPILLARY CARCINOMA, NOS	805	8050/2	Papillary carcinoma in situ
		8050/3	Papillary carcinoma, NOS
		8051/3	Verrucous carcinoma, NOS
		8052/2	Papillary squamous cell carcinoma, non-invasive
		8052/3	Papillary squamous cell carcinoma
SQUAMOUS CELL CARCINOMA, NOS	807	8070/2	Squamous cell carcinoma in situ, NOS
		8070/3	Squamous cell carcinoma, NOS
		8071/3	Sq. cell carcinoma, keratinizing, NOS
		8072/3	Sq. cell carcinoma, lg. cell, non-ker.
		8073/3	Sq. cell carcinoma, sm. cell, non-ker.
		8074/3	Sq. cell carcinoma, spindle cell
		8075/3	Squamous cell carcinoma, adenoid
		8076/2	Sq. cell carc. in situ with question. stromal invas.
		8076/3	Sq. cell carcinoma, micro-invasive
		8078/3	Squamous cell carcinoma with horn formation

APPENDIX C181

ADENOCARCINOMA, NOS	814	8140/2	Adenocarcinoma in situ
		8140/3	Adenocarcinoma, NOS
		8141/3	Scirrhous adenocarcinoma
		8143/3	Superficial spreading adenocarcinoma
		8145/3	Carcinoma, diffuse type
		8147/3	Basal cell adenocarcinoma
ADENOCA. IN ADENOMA. POLYP	821	8210/2	Adenocarcinoma in situ in adenomatous polyp
		8210/3	Adenocarcinoma in adenomatous polyp
		8211/3	Tubular adenocarcinoma
ADENOCA IN FAMIL POLYP COLI	822	8220/2	Adenocarcinoma in situ in familial polyp. coli
		8220/3	Adenocarcinoma in adenoma. polyposis coli
		8221/2	Adenocarc. in situ in mult. adenomatous polyps
		8221/3	Adenocarcinoma in mult. adenomatous polyps
SOLID CARCINOMA, NOS	823	8230/2	Duct carcinoma in situ, solid type
		8230/3	Solid carcinoma, NOS
		8231/3	Carcinoma simplex
CARCINOID TUMOR, MALIGNANT	824	8240/3	Carcinoid tumor, malignant
		8241/3	Enterochromaffin cell carcinoid
		8242/3	Enterochromaffin-like cell tumor, malignant
		8243/3	Goblet cell carcinoid
		8244/3	Composite carcinoid
		8245/3	Adenocarcinoid tumor
		8246/3	Neuroendocrine carcinoma
		8249/3	Atypical carcinoid tumor
BRONCHIOLO-ALVEOLAR ADENOCA.	825	8255/3	Adenocarcinoma with mixed subtypes
PAPILLARY ADENOCARCINOMA, NOS	826	8260/3	Papillary adenocarcinoma, NOS
		8261/2	Adenocarcinoma in situ in villous adenoma
		8261/3	Adenocarcinoma in villous adenoma
		8262/3	Villous adenocarcinoma
		8263/2	Adenocarcinoma in situ in tubulovillous adenoma
		8263/3	Adenocarcinoma in tubulovillous adenoma
MUCOEPIDERMOID CARCINOMA	843	8430/3	Mucoepidermoid carcinoma
CYSTADENOCARCINOMA, NOS	844	8440/3	Cystadenocarcinoma, NOS
MUCINOUS CYSTADENOCARC., NOS	847	8470/2	Mucinous cystadenocarcinoma, non-invasive
		8470/3	Mucinous cystadenocarcinoma, NOS
		8471/3	Papillary mucinous cystadenocarcinoma

APPENDIX C181

MUCINOUS ADENOCARCINOMA	848	8480/3	Mucinous adenocarcinoma
		8481/3	Mucin-producing adenocarcinoma
SIGNET RING CELL CARCINOMA	849	8490/3	Signet ring cell carcinoma
MEDULLARY CARCINOMA, NOS	851	8510/3	Medullary carcinoma, NOS
ACINAR CELL CARCINOMA	855	8550/3	Acinar cell carcinoma
		8551/3	Acinar cell cystadenocarcinoma
ADENOSQUAMOUS CARCINOMA	856	8560/3	Adenosquamous carcinoma
		8562/3	Epithelial-myoepithelial carcinoma
ADENOCA. WITH METAPLASIA	857	8570/3	Adenocarcinoma with squamous metaplasia
		8571/3	Adenocarcinoma w cartilag. & oss. metaplas.
		8572/3	Adenocarcinoma with spindle cell mataplasia
		8573/3	Adenocarcinoma with apocrine metaplasia
		8574/3	Adenocarcinoma with neuroendocrine differen.
		8575/3	Metaplastic carcinoma, NOS
		8576/3	Hepatoid adenocarcinoma
SARCOMA, NOS	880	8800/3	Sarcoma, NOS
		8801/3	Spindle cell sarcoma
		8802/3	Giant cell sarcoma
		8803/3	Small cell sarcoma
		8804/3	Epithelioid sarcoma
		8805/3	Undifferentiated sarcoma
		8806/3	Desmoplastic small round cell tumor
FIBROMATOUS NEOPLASMS	881	8810/3	Fibrosarcoma, NOS
		8811/3	Fibromyxosarcoma
		8813/3	Fascial fibrosarcoma
		8814/3	Infantile fibrosarcoma
		8815/3	Solitary fibrous tumor, malignant
SARCOMA, NOS	882	8825/3	Myofibroblastic sarcoma
LIPOSARCOMA NEOPLASMS	885	8850/3	Liposarcoma, NOS
		8851/3	Liposarcoma, well differentiated
		8852/3	Myxoid liposarcoma
		8853/3	Round cell liposarcoma
		8854/3	Pleomorphic liposarcoma
		8855/3	Mixed type liposarcoma
		8857/3	Fibroblastic liposarcoma
		8858/3	Dedifferentiated liposarcoma

APPENDIX C181

MYOMATOUS NEOPLASMS	889	8890/3	Leiomyosarcoma, NOS
		8891/3	Epithelioid leiomyosarcoma
		8894/3	Angiomyosarcoma
		8895/3	Myosarcoma
		8896/3	Myxoid leiomyosarcoma
STROMAL SARCOMA	893	8934/3	Carcinofibroma
		8935/3	Stromal sarcoma, NOS
		8936/3	Gastrointestinal stromal sarcoma
CARCINOSARCOMA, NOS	898	8980/3	Carcinosarcoma, NOS
		8981/3	Carcinosarcoma, embryonal type
		8982/3	Malignant myoepithelioma
MALIGNANT LYMPHOMA, NOS	959	9590/3	Malignant lymphoma, NOS
		9591/3	Malignant lymphoma, non-Hodgkin
		9596/3	Composite Hodgkin and non-Hodgkin lymphoma
HODGKIN LYMPHOMA	965	9650/3	Hodgkin lymphoma, NOS
		9651/3	Hodgkin lymphoma, lymphocyte-rich
		9652/3	Hodgkin lymphoma, mixed cellularity, NOS
		9653/3	Hodgkin lymphoma, lymphocytic deplet., NOS
		9654/3	Hodgkin lymph., lymphocyt. deplet., diffuse fibrosis
		9655/3	Hodgkin lymphoma, lymphocyt. deplet., reticular
		9659/3	Hodgkin lymph., nodular lymphocyte predom.
HODGKIN LYMPHOMA, NOD. SCLER.	966	9661/3	Hodgkin granuloma [obs]
		9662/3	Hodgkin sarcoma [obs]
		9663/3	Hodgkin lymphoma, nodular sclerosis, NOS
		9664/3	Hodgkin lymphoma, nod. scler., cellular phase
		9665/3	Hodgkin lymphoma, nod. scler., grade 1
		9667/3	Hodgkin lymphoma, nod. scler., grade 2
ML, SMALL B-CELL LYMPHOCYTIC	967	9670/3	ML, small B lymphocytic, NOS
		9671/3	ML, lymphoplasmacytic
		9673/3	Mantle cell lymphoma
		9675/3	ML, mixed sm. and lg. cell, diffuse
ML, LARGE B-CELL, DIFFUSE	968	9680/3	ML, large B-cell, diffuse
		9684/3	ML, large B-cell, diffuse, immunoblastic, NOS
		9687/3	Burkitt lymphoma, NOS
		9688/3	T-cell histiocyte rich large B-cell lymphoma

APPENDIX C181

FOLLIC. & MARGINAL LYMPH, NOS	969	9690/3	Follicular lymphoma, NOS
		9691/3	Follicular lymphoma, grade 2
		9695/3	Follicular lymphoma, grade 1
		9698/3	Follicular lymphoma, grade 3
		9699/3	Marginal zone B-cell lymphoma, NOS
T-CELL LYMPHOMAS	970	9701/3	Sezary syndrome
		9702/3	Mature T-cell lymphoma, NOS
		9705/3	Angioimmunoblastic T-cell lymphoma
OTHER SPEC. NON-HODGKIN LYMPHOMA	971	9712/3	Intravascular large B-cell lymphoma
		9714/3	Anaplastic large cell lymphoma, T-cell and Null cell type
		9717/3	Intestinal T-cell lymphoma
		9719/3	NK/T-cell lymphoma, nasal and nasal-type
PRECURS. CELL LYMPHOBLASTIC LYMPH.	972	9724/3	SystemicEBV pos. T-cell lymphoproliferative disease of childhood
		9727/3	Precursor cell lymphoblastic lymphoma, NOS
		9728/3	Precursor B-cell lymphoblastic lymphoma
		9729/3	Precursor T-cell lymphoblastic lymphoma
PLASMA CELL TUMORS	973	9731/3	Plasmacytoma, NOS
		9734/3	Plasmacytoma, extramedullary
		9735/3	Plasmablastic lymphoma
		9737/3	ALK positive large B-cell lymphoma
		9738/3	Lrg B-cell lymphoma in HHV8-assoc. multicentric Castleman DZ
MAST CELL TUMORS	974	9740/3	Mast cell sarcoma
		9741/3	Malignant mastocytosis
NEOPLASMS OF HISTIOCYTES AND ACCESSORY LYMPHOID CELLS	975	9750/3	Malignant histiocytosis
		9751/3	Langerhans cell histiocytosis, NOS
		9754/3	Langerhans cell histiocytosis, disseminated
		9755/3	Histiocytic sarcoma
		9756/3	Langerhans cell sarcoma
		9757/3	Interdigitating dendritic cell sarcoma
		9758/3	Follicular dendritic cell sarcoma
		9759/3	Fibroblastic reticular cell tumor
PRECURSOR LYMPHOID NEOPLASMS	981	9811/3	B lymphoblastic leukemia/lymphoma, NOS
		9812/3	Leukemia/lymphoma with t(9;22)(q34;q11.2);BCR-ABL1
		9813/3	Leukemia/lymphoma with t(v;11q23);MLL rearranged
		9814/3	Leukemia/lymphoma with t(12;21)(p13;q22);TEL-AML1(ETV6-RUNX1)
		9815/3	B lymphoblastic leukemia/lymphoma with hyperdiploidy
		9816/3	Leukemia/lymphoma with hypodiploidy (hypodiploid ALL)
		9817/3	B lymphblastic leukemia/lymphoma with t(5;14)(q31;q32);IL3-IGH
		9818/3	Leukemia/lymphoma with t(1;19)(q23;p13.3); E2A PBX1 (TCF3 PBX1)

APPENDIX C181

LYMPHOID LEUKEMIA, NOS	982	9823/3	Chronic lymphocytic leukemia/small lymphocytic lymphoma
PROLYMPH/PRECURS LEUKEMIA	983	9831/3	T-cell large granular lymphocytic leukemia
		9837/3	T lymphoblastic leukemia/lymphoma
CHRONIC MYELOPROLIFERATIVE DIS.	996	9965/3	Myeloid and lymphoid neoplasms with PDGFRB rearrangement
		9967/3	Myeloid and lymphoid neoplasm with FGFR1 abnormalities
MYELOPLASTIC/MYELOPROLIFERATIVE NEOPLASMS	997	9971/3	Polymorphic PTLD
		9975/3	Myelodysplastic/Myeloproliferative neoplasm, unclassifiable

RECTUM C209

NEOPLASM	800	8000/3	Neoplasm, malignant
		8001/3	Tumor cells, malignant
		8002/3	Malignant tumor, small cell type
		8003/3	Malignant tumor, giant cell type
		8004/3	Malignant tumor, spindle cell type
		8005/3	Malignant tumor, clear cell type
CARCINOMA, NOS	801	8010/2	Carcinoma in situ, NOS
		8010/3	Carcinoma, NOS
		8011/3	Epithelioma, malignant
		8012/3	Large cell carcinoma, NOS
		8013/3	Large cell neuroendocrine carcinoma
		8014/3	Large cell carcinoma with rhabdoid phenotype
		8015/3	Glassy cell carcinoma
CARCINOMA, UNDIFF., NOS	802	8020/3	Carcinoma, undifferentiated type, NOS
		8021/3	Carcinoma, anaplastic type, NOS
		8022/3	Pleomorphic carcinoma
GIANT & SPINDLE CELL CARCINOMA	803	8030/3	Giant cell and spindle cell carcinoma
		8031/3	Giant cell carcinoma
		8032/3	Spindle cell carcinoma
		8033/3	Pseudosarcomatous carcinoma
		8034/3	Polygonal cell carcinoma
		8035/3	Carcinoma with osteoclast-like giant cells
SMALL CELL CARCINOMA, NOS	804	8041/3	Small cell carcinoma, NOS
		8043/3	Small cell carcinoma, fusiform cell
PAPILLARY CARCINOMA, NOS	805	8050/2	Papillary carcinoma in situ
		8050/3	Papillary carcinoma, NOS
		8051/3	Verrucous carcinoma, NOS
		8052/2	Papillary squamous cell carcinoma, non-invasive
		8052/3	Papillary squamous cell carcinoma
SQUAMOUS CELL CARCINOMA, NOS	807	8070/2	Squamous cell carcinoma in situ, NOS
		8070/3	Squamous cell carcinoma, NOS
		8071/3	Sq. cell carcinoma, keratinizing, NOS
		8072/3	Sq. cell carcinoma, lg. cell, non-ker.
		8073/3	Sq. cell carcinoma, sm. cell, non-ker.
		8074/3	Sq. cell carcinoma, spindle cell
		8075/3	Squamous cell carcinoma, adenoid
		8076/2	Sq. cell carc. in situ with question. stromal invas.
		8076/3	Sq. cell carcinoma, micro-invasive
		8078/3	Squamous cell carcinoma with horn formation

RECTUM C209

TRANSITIONAL CELL CARCINOMA, NOS	812	8120/2	Transitional cell carcinoma in situ
		8120/3	Transitional cell carcinoma, NOS
		8121/3	Schneiderian carcinoma
		8122/3	Trans. cell carcinoma, spindle cell
		8123/3	Basaloid carcinoma
		8124/3	Cloacogenic carcinoma
ADENOCARCINOMA, NOS	814	8140/2	Adenocarcinoma in situ
		8140/3	Adenocarcinoma, NOS
		8141/3	Scirrhous adenocarcinoma
		8143/3	Superficial spreading adenocarcinoma
		8145/3	Carcinoma, diffuse type
		8147/3	Basal cell adenocarcinoma
ADENOCA. IN ADENOMA. POLYP	821	8210/2	Adenocarcinoma in situ in adenomatous polyp
		8210/3	Adenocarcinoma in adenomatous polyp
		8211/3	Tubular adenocarcinoma
		8213/3	Serrated adenocarcinoma
ADENOCA IN FAMIL POLYP COLI	822	8220/2	Adenocarcinoma in situ in familial polyp. coli
		8220/3	Adenocarcinoma in adenoma. polyposis coli
		8221/2	Adenocarc. in situ in mult. adenomatous polyps
		8221/3	Adenocarcinoma in mult. adenomatous polyps
SOLID CARCINOMA, NOS	823	8230/2	Duct carcinoma in situ, solid type
		8230/3	Solid carcinoma, NOS
		8231/3	Carcinoma simplex
CARCINOID TUMOR, MALIGNANT	824	8240/3	Carcinoid tumor, malignant
		8241/3	Enterochromaffin cell carcinoid
		8242/3	Enterochromaffin-like cell tumor, malignant
		8243/3	Goblet cell carcinoid
		8244/3	Composite carcinoid
		8245/3	Adenocarcinoid tumor
		8246/3	Neuroendocrine carcinoma
		8249/3	Atypical carcinoid tumor
BRONCHIOLO-ALVEOLAR ADENOCA.	825	8255/3	Adenocarcinoma with mixed subtypes
PAPILLARY ADENOCARCINOMA, NOS	826	8260/3	Papillary adenocarcinoma, NOS
		8261/2	Adenocarcinoma in situ in villous adenoma
		8261/3	Adenocarcinoma in villous adenoma
		8262/3	Villous adenocarcinoma
		8263/2	Adenocarcinoma in situ in tubulovillous adenoma
		8263/3	Adenocarcinoma in tubulovillous adenoma
		8265/3	Micropapillary carcinoma, NOS

RECTUM C209

MUCOEPIDERMOID CARCINOMA	843	8430/3	Mucoepidermoid carcinoma
CYSTADENOCARCINOMA, NOS	844	8440/3	Cystadenocarcinoma, NOS
MUCINOUS ADENOCARCINOMA	848	8480/3	Mucinous adenocarcinoma
		8481/3	Mucin-producing adenocarcinoma
SIGNET RING CELL CARCINOMA	849	8490/3	Signet ring cell carcinoma
MEDULLARY CARCINOMA, NOS	851	8510/3	Medullary carcinoma, NOS
ACINAR CELL CARCINOMA	855	8550/3	Acinar cell carcinoma
		8551/3	Acinar cell cystadenocarcinoma
ADENOSQUAMOUS CARCINOMA	856	8560/3	Adenosquamous carcinoma
		8562/3	Epithelial-myoepithelial carcinoma
ADENOCA. WITH METAPLASIA	857	8570/3	Adenocarcinoma with squamous metaplasia
		8571/3	Adenocarcinoma w cartilag. & oss. metaplas.
		8572/3	Adenocarcinoma with spindle cell mataplasia
		8573/3	Adenocarcinoma with apocrine metaplasia
		8574/3	Adenocarcinoma with neuroendocrine differen.
		8575/3	Metaplastic carcinoma, NOS
		8576/3	Hepatoid adenocarcinoma
NEVI & MELANOMAS	872	8720/2	Melanoma in situ
		8720/3	Malignant melanoma, NOS
		8721/3	Nodular melanoma
		8722/3	Balloon cell melanoma
		8723/3	Malignant melanoma, regressing
AMELANOTIC MELANOMA	873	8730/3	Amelanotic melanoma
MAL. MEL. IN JUNCT. NEVUS	874	8743/3	Superficial spreading melanoma
		8745/3	Desmoplastic melanoma, malignant
		8746/3	Mucosal lentiginous melanoma
MAL. MELAN. IN GIANT PIGMT. NEVUS	876	8761/3	Mal. melanoma in giant pigmented nevus
EPITHELIOID CELL MELANOMA	877	8770/3	Mixed epithel. & spindle cell melanoma
		8771/3	Epithelioid cell melanoma
		8772/3	Spindle cell melanoma, NOS

RECTUM C209

SARCOMA, NOS	880	8800/3	Sarcoma, NOS
		8801/3	Spindle cell sarcoma
		8802/3	Giant cell sarcoma
		8803/3	Small cell sarcoma
		8804/3	Epithelioid sarcoma
		8805/3	Undifferentiated sarcoma
		8806/3	Desmoplastic small round cell tumor
FIBROMATOUS NEOPLASMS	881	8810/3	Fibrosarcoma, NOS
		8811/3	Fibromyxosarcoma
		8813/3	Fascial fibrosarcoma
		8814/3	Infantile fibrosarcoma
		8815/3	Solitary fibrous tumor, malignant
SARCOMA, NOS	882	8825/3	Myofibroblastic sarcoma
LIPOSARCOMA NEOPLASMS	885	8850/3	Liposarcoma, NOS
		8851/3	Liposarcoma, well differentiated
		8852/3	Myxoid liposarcoma
		8853/3	Round cell liposarcoma
		8854/3	Pleomorphic liposarcoma
		8855/3	Mixed type liposarcoma
		8857/3	Fibroblastic liposarcoma
		8858/3	Dedifferentiated liposarcoma
MYOMATOUS NEOPLASMS	889	8890/3	Leiomyosarcoma, NOS
		8891/3	Epithelioid leiomyosarcoma
		8894/3	Angiomyosarcoma
		8895/3	Myosarcoma
		8896/3	Myxoid leiomyosarcoma
STROMAL SARCOMA	893	8934/3	Carcinofibroma
		8935/3	Stromal sarcoma, NOS
		8936/3	Gastrointestinal stromal sarcoma
CARCINOSARCOMA, NOS	898	8980/3	Carcinosarcoma, NOS
		8981/3	Carcinosarcoma, embryonal type
		8982/3	Malignant myoepithelioma
MALIGNANT LYMPHOMA, NOS	959	9590/3	Malignant lymphoma, NOS
		9591/3	Malignant lymphoma, non-Hodgkin
		9596/3	Composite Hodgkin and non-Hodgkin lymphoma

RECTUM C209

HODGKIN LYMPHOMA	965	9650/3	Hodgkin lymphoma, NOS
		9651/3	Hodgkin lymphoma, lymphocyte-rich
		9652/3	Hodgkin lymphoma, mixed cellularity, NOS
		9653/3	Hodgkin lymphoma, lymphocytic deplet., NOS
		9654/3	Hodgkin lymph., lymphocyt. deplet., diffuse fibrosis
		9655/3	Hodgkin lymphoma, lymphocyt. deplet., reticular
		9659/3	Hodgkin lymph., nodular lymphocyte predom.
HODGKIN LYMPHOMA, NOD. SCLER.	966	9661/3	Hodgkin granuloma [obs]
		9662/3	Hodgkin sarcoma [obs]
		9663/3	Hodgkin lymphoma, nodular sclerosis, NOS
		9664/3	Hodgkin lymphoma, nod. scler., cellular phase
		9665/3	Hodgkin lymphoma, nod. scler., grade 1
		9667/3	Hodgkin lymphoma, nod. scler., grade 2
ML, SMALL B-CELL LYMPHOCYTIC	967	9670/3	ML, small B lymphocytic, NOS
		9671/3	ML, lymphoplasmacytic
		9673/3	Mantle cell lymphoma
		9675/3	ML, mixed sm. and lg. cell, diffuse
ML, LARGE B-CELL, DIFFUSE	968	9680/3	ML, large B-cell, diffuse
		9684/3	ML, large B-cell, diffuse, immunoblastic, NOS
		9687/3	Burkitt lymphoma, NOS
		9688/3	T-cell histiocyte rich large B-cell lymphoma
FOLLIC. & MARGINAL LYMPH, NOS	969	9690/3	Follicular lymphoma, NOS
		9691/3	Follicular lymphoma, grade 2
		9695/3	Follicular lymphoma, grade 1
		9698/3	Follicular lymphoma, grade 3
		9699/3	Marginal zone B-cell lymphoma, NOS
T-CELL LYMPHOMAS	970	9701/3	Sezary syndrome
		9702/3	Mature T-cell lymphoma, NOS
		9705/3	Angioimmunoblastic T-cell lymphoma
OTHER SPEC. NON-HODGKIN LYMPHOMA	971	9712/3	Intravascular large B-cell lymphoma
		9714/3	Anaplastic large cell lymphoma, T-cell and Null cell type
		9717/3	Intestinal T-cell lymphoma
		9719/3	NK/T-cell lymphoma, nasal and nasal-type
PRECURS. CELL LYMPHOBLASTIC LYMPH.	972	9724/3	SystemicEBV pos. T-cell lymphoproliferative disease of childhood
		9727/3	Precursor cell lymphoblastic lymphoma, NOS
		9728/3	Precursor B-cell lymphoblastic lymphoma
		9729/3	Precursor T-cell lymphoblastic lymphoma

RECTUM C209

PLASMA CELL TUMORS	973	9731/3	Plasmacytoma, NOS
		9734/3	Plasmacytoma, extramedullary
		9735/3	Plasmablastic lymphoma
		9737/3	ALK positive large B-cell lymphoma
		9738/3	Lrg B-cell lymphoma in HHV8-assoc. multicentric Castleman DZ
MAST CELL TUMORS	974	9740/3	Mast cell sarcoma
		9741/3	Malignant mastocytosis
NEOPLASMS OF HISTIOCYTES AND ACCESSORY LYMPHOID CELLS	975	9750/3	Malignant histiocytosis
		9751/3	Langerhans cell histiocytosis, NOS
		9754/3	Langerhans cell histiocytosis, disseminated
		9755/3	Histiocytic sarcoma
		9756/3	Langerhans cell sarcoma
		9757/3	Interdigitating dendritic cell sarcoma
		9758/3	Follicular dendritic cell sarcoma
		9759/3	Fibroblastic reticular cell tumor
PRECURSOR LYMPHOID NEOPLASMS	981	9811/3	B lymphoblastic leukemia/lymphoma, NOS
		9812/3	Leukemia/lymphoma with t(9;22)(q34;q11.2);BCR-ABL1
		9813/3	Leukemia/lymphoma with t(v;11q23);MLL rearranged
		9814/3	Leukemia/lymphoma with t(12;21)(p13;q22);TEL-AML1(ETV6-RUNX1)
		9815/3	B lymphoblastic leukemia/lymphoma with hyperdiploidy
		9816/3	Leukemia/lymphoma with hypodiploidy (hypodiploid ALL)
		9817/3	B lymphblastic leukemia/lymphoma with t(5;14)(q31;q32);IL3-IGH
		9818/3	Leukemia/lymphoma with t(1;19)(q23;p13.3); E2A PBX1 (TCF3 PBX1)
LYMPHOID LEUKEMIA, NOS	982	9823/3	Chronic lymphocytic leukemia/small lymphocytic lymphoma
PROLYMPH/PRECURS LEUKEMIA	983	9831/3	T-cell large granular lymphocytic leukemia
		9837/3	T lymphoblastic leukemia/lymphoma
CHRONIC MYELOPROLIFERATIVE DIS.	996	9965/3	Myeloid and lymphoid neoplasms with PDGFRB rearrangement
		9967/3	Myeloid and lymphoid neoplasm with FGFR1 abnormalities
MYELOPLASTIC/MYELOPROLIFERATIVE NEOPLASMS	997	9971/3	Polymorphic PTLD
		9975/3	Myelodysplastic/Myeloproliferative neoplasm, unclassifiable

ANAL CANAL & ANUS C210-C212,C218

NEOPLASM	800	8000/3	Neoplasm, malignant
		8001/3	Tumor cells, malignant
		8002/3	Malignant tumor, small cell type
		8003/3	Malignant tumor, giant cell type
		8004/3	Malignant tumor, spindle cell type
		8005/3	Malignant tumor, clear cell type
CARCINOMA, NOS	801	8010/2	Carcinoma in situ, NOS
		8010/3	Carcinoma, NOS
		8011/3	Epithelioma, malignant
		8012/3	Large cell carcinoma, NOS
		8013/3	Large cell neuroendocrine carcinoma
		8014/3	Large cell carcinoma with rhabdoid phenotype
		8015/3	Glassy cell carcinoma
CARCINOMA, UNDIFF., NOS	802	8020/3	Carcinoma, undifferentiated type, NOS
		8021/3	Carcinoma, anaplastic type, NOS
		8022/3	Pleomorphic carcinoma
SMALL CELL CARCINOMA, NOS	804	8041/3	Small cell carcinoma, NOS
		8043/3	Small cell carcinoma, fusiform cell
PAPILLARY CARCINOMA, NOS	805	8050/2	Papillary carcinoma in situ
		8050/3	Papillary carcinoma, NOS
		8051/3	Verrucous carcinoma, NOS
		8052/2	Papillary squamous cell carcinoma, non-invasive
		8052/3	Papillary squamous cell carcinoma
SQUAMOUS CELL CARCINOMA, NOS	807	8070/2	Squamous cell carcinoma in situ, NOS
		8070/3	Squamous cell carcinoma, NOS
		8071/3	Sq. cell carcinoma, keratinizing, NOS
		8072/3	Sq. cell carcinoma, lg. cell, non-ker.
		8073/3	Sq. cell carcinoma, sm. cell, non-ker.
		8074/3	Sq. cell carcinoma, spindle cell
		8075/3	Squamous cell carcinoma, adenoid
		8076/2	Sq. cell carc. in situ with question. stromal invas.
		8076/3	Sq. cell carcinoma, micro-invasive
		8077/2	Squamous intraepithelial neoplasia, grade III
		8078/3	Squamous cell carcinoma with horn formation
TRANSITIONAL CELL CARCINOMA, NOS	812	8120/2	Transitional cell carcinoma in situ
		8120/3	Transitional cell carcinoma, NOS
		8121/3	Schneiderian carcinoma
		8122/3	Trans. cell carcinoma, spindle cell
		8123/3	Basaloid carcinoma
		8124/3	Cloacogenic carcinoma

ANAL CANAL & ANUS C210-C212,C218

ADENOCARCINOMA, NOS	814	8140/2	Adenocarcinoma in situ
		8140/3	Adenocarcinoma, NOS
		8141/3	Scirrhous adenocarcinoma
		8143/3	Superficial spreading adenocarcinoma
		8145/3	Carcinoma, diffuse type
		8147/3	Basal cell adenocarcinoma
ADENOCA. IN ADENOMA. POLYP	821	8210/2	Adenocarcinoma in situ in adenomatous polyp
		8210/3	Adenocarcinoma in adenomatous polyp
		8215/3	Adenocarcinoma of anal glands
CARCINOID TUMOR, MALIGNANT	824	8240/3	Carcinoid tumor, malignant
		8241/3	Enterochromaffin cell carcinoid
		8242/3	Enterochromaffin-like cell tumor, malignant
		8243/3	Goblet cell carcinoid
		8244/3	Composite carcinoid
		8245/3	Adenocarcinoid tumor
		8246/3	Neuroendocrine carcinoma
		8249/3	Atypical carcinoid tumor
BRONCHIOLO-ALVEOLAR ADENOCA.	825	8255/3	Adenocarcinoma with mixed subtypes
PAPILLARY ADENOCARCINOMA, NOS	826	8260/3	Papillary adenocarcinoma, NOS
		8261/2	Adenocarcinoma in situ in villous adenoma
		8261/3	Adenocarcinoma in villous adenoma
		8262/3	Villous adenocarcinoma
		8263/2	Adenocarcinoma in situ in tubulovillous adenoma
		8263/3	Adenocarcinoma in tubulovillous adenoma
MUCOEPIDERMOID CARCINOMA	843	8430/3	Mucoepidermoid carcinoma
MUCINOUS ADENOCARCINOMA	848	8480/3	Mucinous adenocarcinoma
		8481/3	Mucin-producing adenocarcinoma
DUCT CARCINOMA	850	8500/2	Intraductal carcinoma, noninfiltrating, NOS
		8500/3	Invasive carcinoma of no special type
		8503/2	Noninfiltrating intraductal papillary adenocarcinoma
		8503/3	Intraductal papillary adenocarcinoma with invasion
		8504/2	Noninfiltrating intracystic carcinoma
		8504/3	Intracystic carcinoma, NOS
PAGET DISEASE, EXTRAMAMMARY	854	8542/3	Paget disease, extramammary
ADENOSQUAMOUS CARCINOMA	856	8560/3	Adenosquamous carcinoma
		8562/3	Epithelial-myoepithelial carcinoma

ANAL CANAL & ANUS C210-C212,C218

ADENOCA. WITH METAPLASIA	857	8570/3	Adenocarcinoma with squamous metaplasia
		8571/3	Adenocarcinoma w cartilag. & oss. metaplas.
		8572/3	Adenocarcinoma with spindle cell mataplasia
		8573/3	Adenocarcinoma with apocrine metaplasia
		8574/3	Adenocarcinoma with neuroendocrine differen.
		8575/3	Metaplastic carcinoma, NOS
		8576/3	Hepatoid adenocarcinoma
NEVI & MELANOMAS	872	8720/2	Melanoma in situ
		8720/3	Malignant melanoma, NOS
		8721/3	Nodular melanoma
		8722/3	Balloon cell melanoma
		8723/3	Malignant melanoma, regressing
AMELANOTIC MELANOMA	873	8730/3	Amelanotic melanoma
MAL. MEL. IN JUNCT. NEVUS	874	8743/3	Superficial spreading melanoma
		8745/3	Desmoplastic melanoma, malignant
		8746/3	Mucosal lentiginous melanoma
EPITHELIOID CELL MELANOMA	877	8770/3	Mixed epithel. & spindle cell melanoma
		8771/3	Epithelioid cell melanoma
		8772/3	Spindle cell melanoma, NOS
SARCOMA, NOS	880	8800/3	Sarcoma, NOS
		8801/3	Spindle cell sarcoma
		8802/3	Giant cell sarcoma
		8803/3	Small cell sarcoma
		8804/3	Epithelioid sarcoma
		8805/3	Undifferentiated sarcoma
		8806/3	Desmoplastic small round cell tumor
MYOMATOUS NEOPLASMS	889	8890/3	Leiomyosarcoma, NOS
		8891/3	Epithelioid leiomyosarcoma
		8894/3	Angiomyosarcoma
		8895/3	Myosarcoma
		8896/3	Myxoid leiomyosarcoma
MALIGNANT LYMPHOMA, NOS	959	9590/3	Malignant lymphoma, NOS
		9591/3	Malignant lymphoma, non-Hodgkin
		9596/3	Composite Hodgkin and non-Hodgkin lymphoma
HODGKIN LYMPHOMA	965	9650/3	Hodgkin lymphoma, NOS
		9651/3	Hodgkin lymphoma, lymphocyte-rich
		9652/3	Hodgkin lymphoma, mixed cellularity, NOS
		9653/3	Hodgkin lymphoma, lymphocytic deplet., NOS
		9654/3	Hodgkin lymph., lymphocyt. deplet., diffuse fibrosis

ANAL CANAL & ANUS C210-C212,C218

HODGKIN LYMPHOMA	965	9655/3	Hodgkin lymphoma, lymphocyt. deplet., reticular
		9659/3	Hodgkin lymph., nodular lymphocyte predom.
HODGKIN LYMPHOMA, NOD. SCLER.	966	9661/3	Hodgkin granuloma [obs]
		9662/3	Hodgkin sarcoma [obs]
		9663/3	Hodgkin lymphoma, nodular sclerosis, NOS
		9664/3	Hodgkin lymphoma, nod. scler., cellular phase
		9665/3	Hodgkin lymphoma, nod. scler., grade 1
		9667/3	Hodgkin lymphoma, nod. scler., grade 2
ML, SMALL B-CELL LYMPHOCYTIC	967	9670/3	ML, small B lymphocytic, NOS
		9671/3	ML, lymphoplasmacytic
		9673/3	Mantle cell lymphoma
		9675/3	ML, mixed sm. and lg. cell, diffuse
ML, LARGE B-CELL, DIFFUSE	968	9680/3	ML, large B-cell, diffuse
		9684/3	ML, large B-cell, diffuse, immunoblastic, NOS
		9687/3	Burkitt lymphoma, NOS
		9688/3	T-cell histiocyte rich large B-cell lymphoma
FOLLIC. & MARGINAL LYMPH, NOS	969	9690/3	Follicular lymphoma, NOS
		9691/3	Follicular lymphoma, grade 2
		9695/3	Follicular lymphoma, grade 1
		9698/3	Follicular lymphoma, grade 3
		9699/3	Marginal zone B-cell lymphoma, NOS
T-CELL LYMPHOMAS	970	9701/3	Sezary syndrome
		9702/3	Mature T-cell lymphoma, NOS
		9705/3	Angioimmunoblastic T-cell lymphoma
OTHER SPEC. NON-HODGKIN LYMPHOMA	971	9712/3	Intravascular large B-cell lymphoma
		9714/3	Anaplastic large cell lymphoma, T-cell and Null cell type
		9717/3	Intestinal T-cell lymphoma
		9719/3	NK/T-cell lymphoma, nasal and nasal-type
PRECURS. CELL LYMPHOBLASTIC LYMPH.	972	9724/3	SystemicEBV pos. T-cell lymphoproliferative disease of childhood
		9727/3	Precursor cell lymphoblastic lymphoma, NOS
		9728/3	Precursor B-cell lymphoblastic lymphoma
		9729/3	Precursor T-cell lymphoblastic lymphoma
PLASMA CELL TUMORS	973	9731/3	Plasmacytoma, NOS
		9734/3	Plasmacytoma, extramedullary
		9735/3	Plasmablastic lymphoma
		9737/3	ALK positive large B-cell lymphoma
		9738/3	Lrg B-cell lymphoma in HHV8-assoc. multicentric Castleman DZ

ANAL CANAL & ANUS C210-C212,C218

MAST CELL TUMORS	974	9740/3	Mast cell sarcoma
		9741/3	Malignant mastocytosis
NEOPLASMS OF HISTIOCYTES AND ACCESSORY LYMPHOID CELLS	975	9750/3	Malignant histiocytosis
		9751/3	Langerhans cell histiocytosis, NOS
		9754/3	Langerhans cell histiocytosis, disseminated
		9755/3	Histiocytic sarcoma
		9756/3	Langerhans cell sarcoma
		9757/3	Interdigitating dendritic cell sarcoma
		9758/3	Follicular dendritic cell sarcoma
		9759/3	Fibroblastic reticular cell tumor
PRECURSOR LYMPHOID NEOPLASMS	981	9811/3	B lymphoblastic leukemia/lymphoma, NOS
		9812/3	Leukemia/lymphoma with t(9;22)(q34;q11.2);BCR-ABL1
		9813/3	Leukemia/lymphoma with t(v;11q23);MLL rearranged
		9814/3	Leukemia/lymphoma with t(12;21)(p13;q22);TEL-AML1(ETV6-RUNX1)
		9815/3	B lymphoblastic leukemia/lymphoma with hyperdiploidy
		9816/3	Leukemia/lymphoma with hypodiploidy (hypodiploid ALL)
		9817/3	B lymphblastic leukemia/lymphoma with t(5;14)(q31;q32);IL3-IGH
		9818/3	Leukemia/lymphoma with t(1;19)(q23;p13.3); E2A PBX1 (TCF3 PBX1)
LYMPHOID LEUKEMIA, NOS	982	9823/3	Chronic lymphocytic leukemia/small lymphocytic lymphoma
PROLYMPH/PRECURS LEUKEMIA	983	9831/3	T-cell large granular lymphocytic leukemia
		9837/3	T lymphoblastic leukemia/lymphoma
CHRONIC MYELOPROLIFERATIVE DIS.	996	9965/3	Myeloid and lymphoid neoplasms with PDGFRB rearrangement
		9967/3	Myeloid and lymphoid neoplasm with FGFR1 abnormalities
MYELOPLASTIC/MYELOPROLIFERATIVE NEOPLASMS	997	9971/3	Polymorphic PTLD
		9975/3	Myelodysplastic/Myeloproliferative neoplasm, unclassifiable

LIVER C220

NEOPLASM	800	8000/3	Neoplasm, malignant
		8001/3	Tumor cells, malignant
		8002/3	Malignant tumor, small cell type
		8003/3	Malignant tumor, giant cell type
		8004/3	Malignant tumor, spindle cell type
		8005/3	Malignant tumor, clear cell type
CARCINOMA, NOS	801	8010/2	Carcinoma in situ, NOS
		8010/3	Carcinoma, NOS
		8011/3	Epithelioma, malignant
		8012/3	Large cell carcinoma, NOS
		8013/3	Large cell neuroendocrine carcinoma
		8014/3	Large cell carcinoma with rhabdoid phenotype
		8015/3	Glassy cell carcinoma
CARCINOMA, UNDIFF., NOS	802	8020/3	Carcinoma, undifferentiated type, NOS
		8021/3	Carcinoma, anaplastic type, NOS
		8022/3	Pleomorphic carcinoma
GIANT & SPINDLE CELL CARCINOMA	803	8030/3	Giant cell and spindle cell carcinoma
		8031/3	Giant cell carcinoma
		8032/3	Spindle cell carcinoma
		8033/3	Pseudosarcomatous carcinoma
		8034/3	Polygonal cell carcinoma
		8035/3	Carcinoma with osteoclast-like giant cells
ADENOCARCINOMA, NOS	814	8140/2	Adenocarcinoma in situ
		8140/3	Adenocarcinoma, NOS
		8141/3	Scirrhous adenocarcinoma
		8143/3	Superficial spreading adenocarcinoma
		8147/3	Basal cell adenocarcinoma
CHOLANGIOCARCINOMA	816	8160/3	Cholangiocarcinoma
		8161/3	Bile duct cystadenocarcinoma
HEPATOCELLULAR CARCINOMA, NOS	817	8170/3	Hepatocellular carcinoma, NOS
		8171/3	Hepatocellular carcinoma, fibrolamellar
		8172/3	Hepatocellular carcinoma, scirrhous
		8173/3	Hepatocellular carcinoma, spindle cell variant
		8174/3	Hepatocellular carcinoma, clear cell type
		8175/3	Hepatocellular carcinoma, pleomorphic type
COMB HEPATOCEL CA. & CHOLANG	818	8180/3	Comb. hepatocel. carcinoma & cholangiocarcinoma
TRABECULAR ADENOCARCINOMA	819	8190/3	Trabecular adenocarcinoma
BRONCHIOLO-ALVEOLAR ADENOCA.	825	8255/3	Adenocarcinoma with mixed subtypes

LIVER C220

CLEAR CELL ADENOCARCINOMA, NOS	831	8310/3	Clear cell adenocarcinoma, NOS
MUCINOUS ADENOCARCINOMA	848	8480/3	Mucinous adenocarcinoma
		8481/3	Mucin-producing adenocarcinoma
DUCT CARCINOMA	850	8500/2	Intraductal carcinoma, noninfiltrating, NOS
		8500/3	Invasive carcinoma of no special type
		8501/2	Comedocarcinoma, non-infiltrating
		8501/3	Comedocarcinoma, NOS
		8503/2	Noninfiltrating intraductal papillary adenocarcinoma
		8503/3	Intraductal papillary adenocarcinoma with invasion
		8504/2	Noninfiltrating intracystic carcinoma
		8504/3	Intracystic carcinoma, NOS
		8507/2	Intraductal micropapillary carcinoma
		8508/3	Cystic hypersecretory carcinoma
SARCOMA, NOS	880	8800/3	Sarcoma, NOS
		8801/3	Spindle cell sarcoma
		8802/3	Giant cell sarcoma
		8803/3	Small cell sarcoma
		8804/3	Epithelioid sarcoma
		8805/3	Undifferentiated sarcoma
		8806/3	Desmoplastic small round cell tumor
MYOMATOUS NEOPLASMS	889	8890/3	Leiomyosarcoma, NOS
		8891/3	Epithelioid leiomyosarcoma
		8894/3	Angiomyosarcoma
		8895/3	Myosarcoma
		8896/3	Myxoid leiomyosarcoma
HEPATOBLASTOMA	897	8970/3	Hepatoblastoma
CARCINOSARCOMA, NOS	898	8980/3	Carcinosarcoma, NOS
		8981/3	Carcinosarcoma, embryonal type
		8982/3	Malignant myoepithelioma
MESENCHYMOMA, MALIGNANT	899	8991/3	Embryonal sarcoma
BLOOD VESSEL TUMORS	912	9120/3	Hemangiosarcoma
		9124/3	Kupffer cell sarcoma
HEMANGIOENDOTHELIOMA	913	9130/3	Hemangioendothelioma, malignant
		9133/3	Epithelioid hemangioendothelioma, malignant

LIVER C220

MALIGNANT LYMPHOMA, NOS	959	9590/3	Malignant lymphoma, NOS
		9591/3	Malignant lymphoma, non-Hodgkin
		9596/3	Composite Hodgkin and non-Hodgkin lymphoma
HODGKIN LYMPHOMA	965	9650/3	Hodgkin lymphoma, NOS
		9651/3	Hodgkin lymphoma, lymphocyte-rich
		9652/3	Hodgkin lymphoma, mixed cellularity, NOS
		9653/3	Hodgkin lymphoma, lymphocytic deplet., NOS
		9654/3	Hodgkin lymph., lymphocyt. deplet., diffuse fibrosis
		9655/3	Hodgkin lymphoma, lymphocyt. deplet., reticular
		9659/3	Hodgkin lymph., nodular lymphocyte predom.
HODGKIN LYMPHOMA, NOD. SCLER.	966	9661/3	Hodgkin granuloma [obs]
		9662/3	Hodgkin sarcoma [obs]
		9663/3	Hodgkin lymphoma, nodular sclerosis, NOS
		9664/3	Hodgkin lymphoma, nod. scler., cellular phase
		9665/3	Hodgkin lymphoma, nod. scler., grade 1
		9667/3	Hodgkin lymphoma, nod. scler., grade 2
ML, SMALL B-CELL LYMPHOCYTIC	967	9670/3	ML, small B lymphocytic, NOS
		9671/3	ML, lymphoplasmacytic
		9673/3	Mantle cell lymphoma
		9675/3	ML, mixed sm. and lg. cell, diffuse
ML, LARGE B-CELL, DIFFUSE	968	9680/3	ML, large B-cell, diffuse
		9684/3	ML, large B-cell, diffuse, immunoblastic, NOS
		9687/3	Burkitt lymphoma, NOS
		9688/3	T-cell histiocyte rich large B-cell lymphoma
FOLLIC. & MARGINAL LYMPH, NOS	969	9690/3	Follicular lymphoma, NOS
		9691/3	Follicular lymphoma, grade 2
		9695/3	Follicular lymphoma, grade 1
		9698/3	Follicular lymphoma, grade 3
		9699/3	Marginal zone B-cell lymphoma, NOS
T-CELL LYMPHOMAS	970	9701/3	Sezary syndrome
		9702/3	Mature T-cell lymphoma, NOS
		9705/3	Angioimmunoblastic T-cell lymphoma
OTHER SPEC. NON-HODGKIN LYMPHOMA	971	9712/3	Intravascular large B-cell lymphoma
		9714/3	Anaplastic large cell lymphoma, T-cell and Null cell type
		9716/3	Hepatosplenic gamma-delta cell lymphoma
		9719/3	NK/T-cell lymphoma, nasal and nasal-type

LIVER C220

PRECURS. CELL LYMPHOBLASTIC LYMPH.	972	9724/3	SystemicEBV pos. T-cell lymphoproliferative disease of childhood
		9727/3	Precursor cell lymphoblastic lymphoma, NOS
		9728/3	Precursor B-cell lymphoblastic lymphoma
		9729/3	Precursor T-cell lymphoblastic lymphoma
PLASMA CELL TUMORS	973	9731/3	Plasmacytoma, NOS
		9734/3	Plasmacytoma, extramedullary
		9735/3	Plasmablastic lymphoma
		9737/3	ALK positive large B-cell lymphoma
		9738/3	Lrg B-cell lymphoma in HHV8-assoc. multicentric Castleman DZ
MAST CELL TUMORS	974	9740/3	Mast cell sarcoma
		9741/3	Malignant mastocytosis
NEOPLASMS OF HISTIOCYTES AND ACCESSORY LYMPHOID CELLS	975	9750/3	Malignant histiocytosis
		9751/3	Langerhans cell histiocytosis, NOS
		9754/3	Langerhans cell histiocytosis, disseminated
		9755/3	Histiocytic sarcoma
		9756/3	Langerhans cell sarcoma
		9757/3	Interdigitating dendritic cell sarcoma
		9758/3	Follicular dendritic cell sarcoma
		9759/3	Fibroblastic reticular cell tumor
PRECURSOR LYMPHOID NEOPLASMS	981	9811/3	B lymphoblastic leukemia/lymphoma, NOS
		9812/3	Leukemia/lymphoma with t(9;22)(q34;q11.2);BCR-ABL1
		9813/3	Leukemia/lymphoma with t(v;11q23);MLL rearranged
		9814/3	Leukemia/lymphoma with t(12;21)(p13;q22);TEL-AML1(ETV6-RUNX1)
		9815/3	B lymphoblastic leukemia/lymphoma with hyperdiploidy
		9816/3	Leukemia/lymphoma with hypodiploidy (hypodiploid ALL)
		9817/3	B lymphblastic leukemia/lymphoma with t(5;14)(q31;q32);IL3-IGH
		9818/3	Leukemia/lymphoma with t(1;19)(q23;p13.3); E2A PBX1 (TCF3 PBX1)
LYMPHOID LEUKEMIA, NOS	982	9823/3	Chronic lymphocytic leukemia/small lymphocytic lymphoma
PROLYMPH/PRECURS LEUKEMIA	983	9831/3	T-cell large granular lymphocytic leukemia
		9837/3	T lymphoblastic leukemia/lymphoma
CHRONIC MYELOPROLIFERATIVE DIS.	996	9965/3	Myeloid and lymphoid neoplasms with PDGFRB rearrangement
		9967/3	Myeloid and lymphoid neoplasm with FGFR1 abnormalities
MYELOPLASTIC/MYELOPROLIFERATIVE NEOPLASMS	997	9971/3	Polymorphic PTLD
		9975/3	Myelodysplastic/Myeloproliferative neoplasm, unclassifiable

INTRAHEPATICBILE DUCTS C221

NEOPLASM	800	8000/3	Neoplasm, malignant
		8001/3	Tumor cells, malignant
		8002/3	Malignant tumor, small cell type
		8003/3	Malignant tumor, giant cell type
		8004/3	Malignant tumor, spindle cell type
		8005/3	Malignant tumor, clear cell type
CARCINOMA, NOS	801	8010/2	Carcinoma in situ, NOS
		8010/3	Carcinoma, NOS
		8011/3	Epithelioma, malignant
		8012/3	Large cell carcinoma, NOS
		8013/3	Large cell neuroendocrine carcinoma
		8014/3	Large cell carcinoma with rhabdoid phenotype
		8015/3	Glassy cell carcinoma
CARCINOMA, UNDIFF., NOS	802	8020/3	Carcinoma, undifferentiated type, NOS
		8021/3	Carcinoma, anaplastic type, NOS
		8022/3	Pleomorphic carcinoma
GIANT & SPINDLE CELL CARCINOMA	803	8030/3	Giant cell and spindle cell carcinoma
		8031/3	Giant cell carcinoma
		8032/3	Spindle cell carcinoma
		8033/3	Pseudosarcomatous carcinoma
		8034/3	Polygonal cell carcinoma
		8035/3	Carcinoma with osteoclast-like giant cells
ADENOCARCINOMA, NOS	814	8140/2	Adenocarcinoma in situ
		8140/3	Adenocarcinoma, NOS
		8141/3	Scirrhous adenocarcinoma
		8143/3	Superficial spreading adenocarcinoma
		8147/3	Basal cell adenocarcinoma
CHOLANGIOCARCINOMA	816	8160/3	Cholangiocarcinoma
		8161/3	Bile duct cystadenocarcinoma
		8162/3	Klatskin tumor
HEPATOCELLULAR CARCINOMA, NOS	817	8170/3	Hepatocellular carcinoma, NOS
		8171/3	Hepatocellular carcinoma, fibrolamellar
		8172/3	Hepatocellular carcinoma, scirrhous
		8173/3	Hepatocellular carcinoma, spindle cell variant
		8174/3	Hepatocellular carcinoma, clear cell type
		8175/3	Hepatocellular carcinoma, pleomorphic type
COMB HEPATOCEL CA. & CHOLANG	818	8180/3	Comb. hepatocel. carcinoma & cholangiocarcinoma
TRABECULAR ADENOCARCINOMA	819	8190/3	Trabecular adenocarcinoma

INTRAHEPATICBILE DUCTS C221

BRONCHIOLO-ALVEOLAR ADENOCA.	825	8255/3	Adenocarcinoma with mixed subtypes
CLEAR CELL ADENOCARCINOMA, NOS	831	8310/3	Clear cell adenocarcinoma, NOS
MUCINOUS ADENOCARCINOMA	848	8480/3	Mucinous adenocarcinoma
		8481/3	Mucin-producing adenocarcinoma
DUCT CARCINOMA	850	8500/2	Intraductal carcinoma, noninfiltrating, NOS
		8500/3	Invasive carcinoma of no special type
		8501/2	Comedocarcinoma, non-infiltrating
		8501/3	Comedocarcinoma, NOS
		8503/2	Noninfiltrating intraductal papillary adenocarcinoma
		8503/3	Intraductal papillary adenocarcinoma with invasion
		8504/2	Noninfiltrating intracystic carcinoma
		8504/3	Intracystic carcinoma, NOS
		8507/2	Intraductal micropapillary carcinoma
		8508/3	Cystic hypersecretory carcinoma
SARCOMA, NOS	880	8800/3	Sarcoma, NOS
		8801/3	Spindle cell sarcoma
		8802/3	Giant cell sarcoma
		8803/3	Small cell sarcoma
		8804/3	Epithelioid sarcoma
		8805/3	Undifferentiated sarcoma
		8806/3	Desmoplastic small round cell tumor
MYOMATOUS NEOPLASMS	889	8890/3	Leiomyosarcoma, NOS
		8891/3	Epithelioid leiomyosarcoma
		8894/3	Angiomyosarcoma
		8895/3	Myosarcoma
		8896/3	Myxoid leiomyosarcoma
CARCINOSARCOMA, NOS	898	8980/3	Carcinosarcoma, NOS
		8981/3	Carcinosarcoma, embryonal type
		8982/3	Malignant myoepithelioma
BLOOD VESSEL TUMORS	912	9120/3	Hemangiosarcoma
HEMANGIOENDOTHELIOMA	913	9130/3	Hemangioendothelioma, malignant
		9133/3	Epithelioid hemangioendothelioma, malignant
MALIGNANT LYMPHOMA, NOS	959	9590/3	Malignant lymphoma, NOS
		9591/3	Malignant lymphoma, non-Hodgkin
		9596/3	Composite Hodgkin and non-Hodgkin lymphoma

INTRAHEPATICBILE DUCTS C221

HODGKIN LYMPHOMA	965	9650/3	Hodgkin lymphoma, NOS
		9651/3	Hodgkin lymphoma, lymphocyte-rich
		9652/3	Hodgkin lymphoma, mixed cellularity, NOS
		9653/3	Hodgkin lymphoma, lymphocytic deplet., NOS
		9654/3	Hodgkin lymph., lymphocyt. deplet., diffuse fibrosis
		9655/3	Hodgkin lymphoma, lymphocyt. deplet., reticular
		9659/3	Hodgkin lymph., nodular lymphocyte predom.
HODGKIN LYMPHOMA, NOD. SCLER.	966	9661/3	Hodgkin granuloma [obs]
		9662/3	Hodgkin sarcoma [obs]
		9663/3	Hodgkin lymphoma, nodular sclerosis, NOS
		9664/3	Hodgkin lymphoma, nod. scler., cellular phase
		9665/3	Hodgkin lymphoma, nod. scler., grade 1
		9667/3	Hodgkin lymphoma, nod. scler., grade 2
ML, SMALL B-CELL LYMPHOCYTIC	967	9670/3	ML, small B lymphocytic, NOS
		9671/3	ML, lymphoplasmacytic
		9673/3	Mantle cell lymphoma
		9675/3	ML, mixed sm. and lg. cell, diffuse
ML, LARGE B-CELL, DIFFUSE	968	9680/3	ML, large B-cell, diffuse
		9684/3	ML, large B-cell, diffuse, immunoblastic, NOS
		9687/3	Burkitt lymphoma, NOS
		9688/3	T-cell histiocyte rich large B-cell lymphoma
FOLLIC. & MARGINAL LYMPH, NOS	969	9690/3	Follicular lymphoma, NOS
		9691/3	Follicular lymphoma, grade 2
		9695/3	Follicular lymphoma, grade 1
		9698/3	Follicular lymphoma, grade 3
		9699/3	Marginal zone B-cell lymphoma, NOS
T-CELL LYMPHOMAS	970	9701/3	Sezary syndrome
		9702/3	Mature T-cell lymphoma, NOS
		9705/3	Angioimmunoblastic T-cell lymphoma
OTHER SPEC. NON-HODGKIN LYMPHOMA	971	9712/3	Intravascular large B-cell lymphoma
		9714/3	Anaplastic large cell lymphoma, T-cell and Null cell type
		9716/3	Hepatosplenic gamma-delta cell lymphoma
		9719/3	NK/T-cell lymphoma, nasal and nasal-type
PRECURS. CELL LYMPHOBLASTIC LYMPH.	972	9724/3	SystemicEBV pos. T-cell lymphoproliferative disease of childhood
		9727/3	Precursor cell lymphoblastic lymphoma, NOS
		9728/3	Precursor B-cell lymphoblastic lymphoma
		9729/3	Precursor T-cell lymphoblastic lymphoma

INTRAHEPATICBILE DUCTS C221

PLASMA CELL TUMORS	973	9731/3	Plasmacytoma, NOS
		9734/3	Plasmacytoma, extramedullary
		9735/3	Plasmablastic lymphoma
		9737/3	ALK positive large B-cell lymphoma
		9738/3	Lrg B-cell lymphoma in HHV8-assoc. multicentric Castleman DZ
MAST CELL TUMORS	974	9740/3	Mast cell sarcoma
		9741/3	Malignant mastocytosis
NEOPLASMS OF HISTIOCYTES AND ACCESSORY LYMPHOID CELLS	975	9750/3	Malignant histiocytosis
		9751/3	Langerhans cell histiocytosis, NOS
		9754/3	Langerhans cell histiocytosis, disseminated
		9755/3	Histiocytic sarcoma
		9756/3	Langerhans cell sarcoma
		9757/3	Interdigitating dendritic cell sarcoma
		9758/3	Follicular dendritic cell sarcoma
		9759/3	Fibroblastic reticular cell tumor
PRECURSOR LYMPHOID NEOPLASMS	981	9811/3	B lymphoblastic leukemia/lymphoma, NOS
		9812/3	Leukemia/lymphoma with t(9;22)(q34;q11.2);BCR-ABL1
		9813/3	Leukemia/lymphoma with t(v;11q23);MLL rearranged
		9814/3	Leukemia/lymphoma with t(12;21)(p13;q22);TEL-AML1(ETV6-RUNX1)
		9815/3	B lymphoblastic leukemia/lymphoma with hyperdiploidy
		9816/3	Leukemia/lymphoma with hypodiploidy (hypodiploid ALL)
		9817/3	B lymphblastic leukemia/lymphoma with t(5;14)(q31;q32);IL3-IGH
		9818/3	Leukemia/lymphoma with t(1;19)(q23;p13.3); E2A PBX1 (TCF3 PBX1)
LYMPHOID LEUKEMIA, NOS	982	9823/3	Chronic lymphocytic leukemia/small lymphocytic lymphoma
PROLYMPH/PRECURS LEUKEMIA	983	9831/3	T-cell large granular lymphocytic leukemia
		9837/3	T lymphoblastic leukemia/lymphoma
CHRONIC MYELOPROLIFERATIVE DIS.	996	9965/3	Myeloid and lymphoid neoplasms with PDGFRB rearrangement
		9967/3	Myeloid and lymphoid neoplasm with FGFR1 abnormalities
MYELOPLASTIC/MYELOPROLIFERATIVE NEOPLASMS	997	9971/3	Polymorphic PTLD
		9975/3	Myelodysplastic/Myeloproliferative neoplasm, unclassifiable

GALLBLADDER& EXTRAHEPATICBILE DUCTS C239-C241,C248-C249

NEOPLASM	800	8000/3	Neoplasm, malignant
		8001/3	Tumor cells, malignant
		8002/3	Malignant tumor, small cell type
		8003/3	Malignant tumor, giant cell type
		8004/3	Malignant tumor, spindle cell type
		8005/3	Malignant tumor, clear cell type
CARCINOMA, NOS	801	8010/2	Carcinoma in situ, NOS
		8010/3	Carcinoma, NOS
		8011/3	Epithelioma, malignant
		8012/3	Large cell carcinoma, NOS
		8013/3	Large cell neuroendocrine carcinoma
		8014/3	Large cell carcinoma with rhabdoid phenotype
		8015/3	Glassy cell carcinoma
CARCINOMA, UNDIFF., NOS	802	8020/3	Carcinoma, undifferentiated type, NOS
		8021/3	Carcinoma, anaplastic type, NOS
		8022/3	Pleomorphic carcinoma
SMALL CELL CARCINOMA, NOS	804	8041/3	Small cell carcinoma, NOS
		8043/3	Small cell carcinoma, fusiform cell
PAPILLARY CARCINOMA, NOS	805	8050/2	Papillary carcinoma in situ
		8050/3	Papillary carcinoma, NOS
		8051/3	Verrucous carcinoma, NOS
		8052/2	Papillary squamous cell carcinoma, non-invasive
		8052/3	Papillary squamous cell carcinoma
SQUAMOUS CELL CARCINOMA, NOS	807	8070/2	Squamous cell carcinoma in situ, NOS
		8070/3	Squamous cell carcinoma, NOS
		8071/3	Sq. cell carcinoma, keratinizing, NOS
		8072/3	Sq. cell carcinoma, lg. cell, non-ker.
		8073/3	Sq. cell carcinoma, sm. cell, non-ker.
		8074/3	Sq. cell carcinoma, spindle cell
		8075/3	Squamous cell carcinoma, adenoid
		8076/2	Sq. cell carc. in situ with question. stromal invas.
		8076/3	Sq. cell carcinoma, micro-invasive
		8078/3	Squamous cell carcinoma with horn formation
ADENOCARCINOMA, NOS	814	8140/2	Adenocarcinoma in situ
		8140/3	Adenocarcinoma, NOS
		8141/3	Scirrhous adenocarcinoma
		8143/3	Superficial spreading adenocarcinoma
		8147/3	Basal cell adenocarcinoma

GALLBLADDER& EXTRAHEPATICBILE DUCTS C239-C241,C248-C249

CHOLANGIOCARCINOMA	816	8160/3	Cholangiocarcinoma
		8161/3	Bile duct cystadenocarcinoma
		8162/3	Klatskin tumor
		8163/3	Pancreatobiliary-type carcinoma
BRONCHIOLO-ALVEOLAR ADENOCA.	825	8255/3	Adenocarcinoma with mixed subtypes
PAPILLARY ADENOCARCINOMA, NOS	826	8260/3	Papillary adenocarcinoma, NOS
		8261/2	Adenocarcinoma in situ in villous adenoma
		8261/3	Adenocarcinoma in villous adenoma
		8262/3	Villous adenocarcinoma
		8263/2	Adenocarcinoma in situ in tubulovillous adenoma
		8263/3	Adenocarcinoma in tubulovillous adenoma
MUCINOUS ADENOCARCINOMA	848	8480/3	Mucinous adenocarcinoma
		8481/3	Mucin-producing adenocarcinoma
SIGNET RING CELL CARCINOMA	849	8490/3	Signet ring cell carcinoma
DUCT CARCINOMA	850	8500/2	Intraductal carcinoma, noninfiltrating, NOS
		8500/3	Invasive carcinoma of no special type
		8501/2	Comedocarcinoma, non-infiltrating
		8501/3	Comedocarcinoma, NOS
		8503/2	Noninfiltrating intraductal papillary adenocarcinoma
		8503/3	Intraductal papillary adenocarcinoma with invasion
		8504/2	Noninfiltrating intracystic carcinoma
		8504/3	Intracystic carcinoma, NOS
		8507/2	Intraductal micropapillary carcinoma
		8508/3	Cystic hypersecretory carcinoma
ADENOSQUAMOUS CARCINOMA	856	8560/3	Adenosquamous carcinoma
		8562/3	Epithelial-myoepithelial carcinoma
ADENOCA. WITH METAPLASIA	857	8570/3	Adenocarcinoma with squamous metaplasia
		8571/3	Adenocarcinoma w cartilag. & oss. metaplas.
		8572/3	Adenocarcinoma with spindle cell mataplasia
		8573/3	Adenocarcinoma with apocrine metaplasia
		8574/3	Adenocarcinoma with neuroendocrine differen.
		8575/3	Metaplastic carcinoma, NOS
		8576/3	Hepatoid adenocarcinoma
SARCOMA, NOS	880	8800/3	Sarcoma, NOS
		8801/3	Spindle cell sarcoma
		8802/3	Giant cell sarcoma
		8803/3	Small cell sarcoma
		8804/3	Epithelioid sarcoma
		8805/3	Undifferentiated sarcoma

GALLBLADDER& EXTRAHEPATICBILE DUCTS C239-C241,C248-C249

SARCOMA, NOS	880	8806/3	Desmoplastic small round cell tumor
MYOMATOUS NEOPLASMS	889	8890/3	Leiomyosarcoma, NOS
		8891/3	Epithelioid leiomyosarcoma
		8894/3	Angiomyosarcoma
		8895/3	Myosarcoma
		8896/3	Myxoid leiomyosarcoma
RHABDOMYOSARCOMA, NOS	890	8900/3	Rhabdomyosarcoma, NOS
		8901/3	Pleomorphic rhabdomyosarcoma, adult type
		8902/3	Mixed type rhabdomyosarcoma
CARCINOSARCOMA, NOS	898	8980/3	Carcinosarcoma, NOS
		8981/3	Carcinosarcoma, embryonal type
		8982/3	Malignant myoepithelioma
MALIGNANT LYMPHOMA, NOS	959	9590/3	Malignant lymphoma, NOS
		9591/3	Malignant lymphoma, non-Hodgkin
		9596/3	Composite Hodgkin and non-Hodgkin lymphoma
HODGKIN LYMPHOMA	965	9650/3	Hodgkin lymphoma, NOS
		9651/3	Hodgkin lymphoma, lymphocyte-rich
		9652/3	Hodgkin lymphoma, mixed cellularity, NOS
		9653/3	Hodgkin lymphoma, lymphocytic deplet., NOS
		9654/3	Hodgkin lymph., lymphocyt. deplet., diffuse fibrosis
		9655/3	Hodgkin lymphoma, lymphocyt. deplet., reticular
		9659/3	Hodgkin lymph., nodular lymphocyte predom.
HODGKIN LYMPHOMA, NOD. SCLER.	966	9661/3	Hodgkin granuloma [obs]
		9662/3	Hodgkin sarcoma [obs]
		9663/3	Hodgkin lymphoma, nodular sclerosis, NOS
		9664/3	Hodgkin lymphoma, nod. scler., cellular phase
		9665/3	Hodgkin lymphoma, nod. scler., grade 1
		9667/3	Hodgkin lymphoma, nod. scler., grade 2
ML, SMALL B-CELL LYMPHOCYTIC	967	9670/3	ML, small B lymphocytic, NOS
		9671/3	ML, lymphoplasmacytic
		9673/3	Mantle cell lymphoma
		9675/3	ML, mixed sm. and lg. cell, diffuse
ML, LARGE B-CELL, DIFFUSE	968	9680/3	ML, large B-cell, diffuse
		9684/3	ML, large B-cell, diffuse, immunoblastic, NOS
		9687/3	Burkitt lymphoma, NOS
		9688/3	T-cell histiocyte rich large B-cell lymphoma

GALLBLADDER& EXTRAHEPATICBILE DUCTS C239-C241,C248-C249

FOLLIC. & MARGINAL LYMPH, NOS	969	9690/3	Follicular lymphoma, NOS
		9691/3	Follicular lymphoma, grade 2
		9695/3	Follicular lymphoma, grade 1
		9698/3	Follicular lymphoma, grade 3
		9699/3	Marginal zone B-cell lymphoma, NOS
T-CELL LYMPHOMAS	970	9701/3	Sezary syndrome
		9702/3	Mature T-cell lymphoma, NOS
		9705/3	Angioimmunoblastic T-cell lymphoma
OTHER SPEC. NON-HODGKIN LYMPHOMA	971	9712/3	Intravascular large B-cell lymphoma
		9714/3	Anaplastic large cell lymphoma, T-cell and Null cell type
		9716/3	Hepatosplenic gamma-delta cell lymphoma
		9719/3	NK/T-cell lymphoma, nasal and nasal-type
PRECURS. CELL LYMPHOBLASTIC LYMPH.	972	9724/3	SystemicEBV pos. T-cell lymphoproliferative disease of childhood
		9727/3	Precursor cell lymphoblastic lymphoma, NOS
		9728/3	Precursor B-cell lymphoblastic lymphoma
		9729/3	Precursor T-cell lymphoblastic lymphoma
PLASMA CELL TUMORS	973	9731/3	Plasmacytoma, NOS
		9734/3	Plasmacytoma, extramedullary
		9735/3	Plasmablastic lymphoma
		9737/3	ALK positive large B-cell lymphoma
		9738/3	Lrg B-cell lymphoma in HHV8-assoc. multicentric Castleman DZ
MAST CELL TUMORS	974	9740/3	Mast cell sarcoma
		9741/3	Malignant mastocytosis
NEOPLASMS OF HISTIOCYTES AND ACCESSORY LYMPHOID CELLS	975	9750/3	Malignant histiocytosis
		9751/3	Langerhans cell histiocytosis, NOS
		9754/3	Langerhans cell histiocytosis, disseminated
		9755/3	Histiocytic sarcoma
		9756/3	Langerhans cell sarcoma
		9757/3	Interdigitating dendritic cell sarcoma
		9758/3	Follicular dendritic cell sarcoma
		9759/3	Fibroblastic reticular cell tumor
PRECURSOR LYMPHOID NEOPLASMS	981	9811/3	B lymphoblastic leukemia/lymphoma, NOS
		9812/3	Leukemia/lymphoma with t(9;22)(q34;q11.2);BCR-ABL1
		9813/3	Leukemia/lymphoma with t(v;11q23);MLL rearranged
		9814/3	Leukemia/lymphoma with t(12;21)(p13;q22);TEL-AML1(ETV6-RUNX1)
		9815/3	B lymphoblastic leukemia/lymphoma with hyperdiploidy
		9816/3	Leukemia/lymphoma with hypodiploidy (hypodiploid ALL)
		9817/3	B lymphblastic leukemia/lymphoma with t(5;14)(q31;q32);IL3-IGH
		9818/3	Leukemia/lymphoma with t(1;19)(q23;p13.3); E2A PBX1 (TCF3 PBX1)

GALLBLADDER& EXTRAHEPATICBILE DUCTS C239-C241,C248-C249

LYMPHOID LEUKEMIA, NOS	982	9823/3	Chronic lymphocytic leukemia/small lymphocytic lymphoma
PROLYMPH/PRECURS LEUKEMIA	983	9831/3	T-cell large granular lymphocytic leukemia
		9837/3	T lymphoblastic leukemia/lymphoma
CHRONIC MYELOPROLIFERATIVE DIS.	996	9965/3	Myeloid and lymphoid neoplasms with PDGFRB rearrangement
		9967/3	Myeloid and lymphoid neoplasm with FGFR1 abnormalities
MYELOPLASTIC/MYELOPROLIFERATIVE NEOPLASMS	997	9971/3	Polymorphic PTLD
		9975/3	Myelodysplastic/Myeloproliferative neoplasm, unclassifiable

PANCREAS C250-C254,C257-C259

NEOPLASM	800	8000/3	Neoplasm, malignant
		8001/3	Tumor cells, malignant
		8002/3	Malignant tumor, small cell type
		8003/3	Malignant tumor, giant cell type
		8004/3	Malignant tumor, spindle cell type
		8005/3	Malignant tumor, clear cell type
CARCINOMA, NOS	801	8010/2	Carcinoma in situ, NOS
		8010/3	Carcinoma, NOS
		8011/3	Epithelioma, malignant
		8012/3	Large cell carcinoma, NOS
		8013/3	Large cell neuroendocrine carcinoma
		8014/3	Large cell carcinoma with rhabdoid phenotype
		8015/3	Glassy cell carcinoma
CARCINOMA, UNDIFF., NOS	802	8020/3	Carcinoma, undifferentiated type, NOS
		8021/3	Carcinoma, anaplastic type, NOS
		8022/3	Pleomorphic carcinoma
GIANT & SPINDLE CELL CARCINOMA	803	8030/3	Giant cell and spindle cell carcinoma
		8031/3	Giant cell carcinoma
		8032/3	Spindle cell carcinoma
		8033/3	Pseudosarcomatous carcinoma
		8034/3	Polygonal cell carcinoma
		8035/3	Carcinoma with osteoclast-like giant cells
SMALL CELL CARCINOMA, NOS	804	8041/3	Small cell carcinoma, NOS
		8043/3	Small cell carcinoma, fusiform cell
PAPILLARY CARCINOMA, NOS	805	8050/2	Papillary carcinoma in situ
		8050/3	Papillary carcinoma, NOS
		8051/3	Verrucous carcinoma, NOS
		8052/2	Papillary squamous cell carcinoma, non-invasive
		8052/3	Papillary squamous cell carcinoma
SQUAMOUS CELL CARCINOMA, NOS	807	8070/2	Squamous cell carcinoma in situ, NOS
		8070/3	Squamous cell carcinoma, NOS
		8071/3	Sq. cell carcinoma, keratinizing, NOS
		8072/3	Sq. cell carcinoma, lg. cell, non-ker.
		8073/3	Sq. cell carcinoma, sm. cell, non-ker.
		8074/3	Sq. cell carcinoma, spindle cell
		8075/3	Squamous cell carcinoma, adenoid
		8076/2	Sq. cell carc. in situ with question. stromal invas.
		8076/3	Sq. cell carcinoma, micro-invasive
		8078/3	Squamous cell carcinoma with horn formation

PANCREAS C250-C254,C257-C259

ADENOCARCINOMA, NOS	814	8140/2	Adenocarcinoma in situ
		8140/3	Adenocarcinoma, NOS
		8141/3	Scirrhous adenocarcinoma
		8143/3	Superficial spreading adenocarcinoma
		8147/3	Basal cell adenocarcinoma
ENDOCRINOMAS	815	8150/3	Islet cell carcinoma
		8151/3	Insulinoma, malignant
		8152/3	Glucagonoma, malignant
		8153/3	Gastrinoma, malignant
		8154/3	Mixed islet cell & exocrine adenocarcinoma
		8155/3	Vipoma
		8156/3	Somatostatinoma, malignant
		8157/3	Enteroglucagonoma, malignant
ADENOCA. IN ADENOMA. POLYP	821	8210/2	Adenocarcinoma in situ in adenomatous polyp
		8210/3	Adenocarcinoma in adenomatous polyp
		8211/3	Tubular adenocarcinoma
SOLID CARCINOMA, NOS	823	8230/2	Duct carcinoma in situ, solid type
		8230/3	Solid carcinoma, NOS
		8231/3	Carcinoma simplex
CARCINOID TUMOR, MALIGNANT	824	8240/3	Carcinoid tumor, malignant
		8241/3	Enterochromaffin cell carcinoid
		8242/3	Enterochromaffin-like cell tumor, malignant
		8243/3	Goblet cell carcinoid
		8244/3	Composite carcinoid
		8245/3	Adenocarcinoid tumor
		8246/3	Neuroendocrine carcinoma
		8249/3	Atypical carcinoid tumor
BRONCHIOLO-ALVEOLAR ADENOCA.	825	8255/3	Adenocarcinoma with mixed subtypes
PAPILLARY ADENOCARCINOMA, NOS	826	8260/3	Papillary adenocarcinoma, NOS
		8261/2	Adenocarcinoma in situ in villous adenoma
		8261/3	Adenocarcinoma in villous adenoma
		8262/3	Villous adenocarcinoma
		8263/2	Adenocarcinoma in situ in tubulovillous adenoma
		8263/3	Adenocarcinoma in tubulovillous adenoma
CLEAR CELL ADENOCARCINOMA, NOS	831	8310/3	Clear cell adenocarcinoma, NOS
GRANULAR CELL CARCINOMA	832	8323/3	Mixed cell adenocarcinoma
MUCOEPIDERMOID CARCINOMA	843	8430/3	Mucoepidermoid carcinoma

PANCREAS C250-C254,C257-C259

CYSTADENOCARCINOMA, NOS	844	8440/3	Cystadenocarcinoma, NOS
PAPILLARY CYSTADENOCA., NOS	845	8450/3	Papillary cystadenocarcinoma, NOS
		8452/3	Solid pseudopapillary carcinoma
		8453/2	Intraductal papillary-mucinous carcinoma, non-inv.
		8453/3	Intraductal papillary-mucinous carcinoma, invasive
MUCINOUS CYSTADENOCARC., NOS	847	8470/2	Mucinous cystadenocarcinoma, non-invasive
		8470/3	Mucinous cystadenocarcinoma, NOS
		8471/3	Papillary mucinous cystadenocarcinoma
MUCINOUS ADENOCARCINOMA	848	8480/3	Mucinous adenocarcinoma
		8481/3	Mucin-producing adenocarcinoma
SIGNET RING CELL CARCINOMA	849	8490/3	Signet ring cell carcinoma
DUCT CARCINOMA	850	8500/2	Intraductal carcinoma, noninfiltrating, NOS
		8500/3	Invasive carcinoma of no special type
		8501/2	Comedocarcinoma, non-infiltrating
		8501/3	Comedocarcinoma, NOS
		8503/2	Noninfiltrating intraductal papillary adenocarcinoma
		8503/3	Intraductal papillary adenocarcinoma with invasion
		8504/2	Noninfiltrating intracystic carcinoma
		8504/3	Intracystic carcinoma, NOS
		8507/2	Intraductal micropapillary carcinoma
		8508/3	Cystic hypersecretory carcinoma
MEDULLARY CARCINOMA, NOS	851	8510/3	Medullary carcinoma, NOS
		8514/3	Duct carcinoma, desmoplastic type
LOBULAR AND OTHER DUCTAL CA.	852	8521/3	Infiltrating ductular carcinoma
ACINAR CELL CARCINOMA	855	8550/3	Acinar cell carcinoma
		8551/3	Acinar cell cystadenocarcinoma
		8552/3	Mixed acinar ductal carcinoma
ADENOSQUAMOUS CARCINOMA	856	8560/3	Adenosquamous carcinoma
		8562/3	Epithelial-myoepithelial carcinoma
ADENOCA. WITH METAPLASIA	857	8570/3	Adenocarcinoma with squamous metaplasia
		8571/3	Adenocarcinoma w cartilag. & oss. metaplas.
		8572/3	Adenocarcinoma with spindle cell mataplasia
		8573/3	Adenocarcinoma with apocrine metaplasia
		8574/3	Adenocarcinoma with neuroendocrine differen.
		8575/3	Metaplastic carcinoma, NOS
		8576/3	Hepatoid adenocarcinoma

PANCREAS C250-C254,C257-C259

SARCOMA, NOS	880	8800/3	Sarcoma, NOS
		8801/3	Spindle cell sarcoma
		8802/3	Giant cell sarcoma
		8803/3	Small cell sarcoma
		8804/3	Epithelioid sarcoma
		8805/3	Undifferentiated sarcoma
		8806/3	Desmoplastic small round cell tumor
MYOMATOUS NEOPLASMS	889	8890/3	Leiomyosarcoma, NOS
		8891/3	Epithelioid leiomyosarcoma
		8894/3	Angiomyosarcoma
		8895/3	Myosarcoma
		8896/3	Myxoid leiomyosarcoma
PANCREATOBLASTOMA	897	8971/3	Pancreatoblastoma
CARCINOSARCOMA, NOS	898	8980/3	Carcinosarcoma, NOS
		8981/3	Carcinosarcoma, embryonal type
		8982/3	Malignant myoepithelioma
MALIGNANT LYMPHOMA, NOS	959	9590/3	Malignant lymphoma, NOS
		9591/3	Malignant lymphoma, non-Hodgkin
		9596/3	Composite Hodgkin and non-Hodgkin lymphoma
HODGKIN LYMPHOMA	965	9650/3	Hodgkin lymphoma, NOS
		9651/3	Hodgkin lymphoma, lymphocyte-rich
		9652/3	Hodgkin lymphoma, mixed cellularity, NOS
		9653/3	Hodgkin lymphoma, lymphocytic deplet., NOS
		9654/3	Hodgkin lymph., lymphocyt. deplet., diffuse fibrosis
		9655/3	Hodgkin lymphoma, lymphocyt. deplet., reticular
		9659/3	Hodgkin lymph., nodular lymphocyte predom.
HODGKIN LYMPHOMA, NOD. SCLER.	966	9661/3	Hodgkin granuloma [obs]
		9662/3	Hodgkin sarcoma [obs]
		9663/3	Hodgkin lymphoma, nodular sclerosis, NOS
		9664/3	Hodgkin lymphoma, nod. scler., cellular phase
		9665/3	Hodgkin lymphoma, nod. scler., grade 1
		9667/3	Hodgkin lymphoma, nod. scler., grade 2
ML, SMALL B-CELL LYMPHOCYTIC	967	9670/3	ML, small B lymphocytic, NOS
		9671/3	ML, lymphoplasmacytic
		9673/3	Mantle cell lymphoma
		9675/3	ML, mixed sm. and lg. cell, diffuse

PANCREAS C250-C254,C257-C259

ML, LARGE B-CELL, DIFFUSE	968	9680/3	ML, large B-cell, diffuse
		9684/3	ML, large B-cell, diffuse, immunoblastic, NOS
		9687/3	Burkitt lymphoma, NOS
		9688/3	T-cell histiocyte rich large B-cell lymphoma
FOLLIC. & MARGINAL LYMPH, NOS	969	9690/3	Follicular lymphoma, NOS
		9691/3	Follicular lymphoma, grade 2
		9695/3	Follicular lymphoma, grade 1
		9698/3	Follicular lymphoma, grade 3
		9699/3	Marginal zone B-cell lymphoma, NOS
T-CELL LYMPHOMAS	970	9701/3	Sezary syndrome
		9702/3	Mature T-cell lymphoma, NOS
		9705/3	Angioimmunoblastic T-cell lymphoma
OTHER SPEC. NON-HODGKIN LYMPHOMA	971	9712/3	Intravascular large B-cell lymphoma
		9714/3	Anaplastic large cell lymphoma, T-cell and Null cell type
		9719/3	NK/T-cell lymphoma, nasal and nasal-type
PRECURS. CELL LYMPHOBLASTIC LYMPH.	972	9724/3	SystemicEBV pos. T-cell lymphoproliferative disease of childhood
		9727/3	Precursor cell lymphoblastic lymphoma, NOS
		9728/3	Precursor B-cell lymphoblastic lymphoma
		9729/3	Precursor T-cell lymphoblastic lymphoma
PLASMA CELL TUMORS	973	9731/3	Plasmacytoma, NOS
		9734/3	Plasmacytoma, extramedullary
		9735/3	Plasmablastic lymphoma
		9737/3	ALK positive large B-cell lymphoma
		9738/3	Lrg B-cell lymphoma in HHV8-assoc. multicentric Castleman DZ
MAST CELL TUMORS	974	9740/3	Mast cell sarcoma
		9741/3	Malignant mastocytosis
NEOPLASMS OF HISTIOCYTES AND ACCESSORY LYMPHOID CELLS	975	9750/3	Malignant histiocytosis
		9751/3	Langerhans cell histiocytosis, NOS
		9754/3	Langerhans cell histiocytosis, disseminated
		9755/3	Histiocytic sarcoma
		9756/3	Langerhans cell sarcoma
		9757/3	Interdigitating dendritic cell sarcoma
		9758/3	Follicular dendritic cell sarcoma
		9759/3	Fibroblastic reticular cell tumor
PRECURSOR LYMPHOID NEOPLASMS	981	9811/3	B lymphoblastic leukemia/lymphoma, NOS
		9812/3	Leukemia/lymphoma with t(9;22)(q34;q11.2);BCR-ABL1
		9813/3	Leukemia/lymphoma with t(v;11q23);MLL rearranged
		9814/3	Leukemia/lymphoma with t(12;21)(p13;q22);TEL-AML1(ETV6-RUNX1)
		9815/3	B lymphoblastic leukemia/lymphoma with hyperdiploidy

PANCREAS C250-C254,C257-C259

PRECURSOR LYMPHOID NEOPLASMS	981	9816/3	Leukemia/lymphoma with hypodiploidy (hypodiploid ALL)
		9817/3	B lymphblastic leukemia/lymphoma with t(5;14)(q31;q32);IL3-IGH
		9818/3	Leukemia/lymphoma with t(1;19)(q23;p13.3); E2A PBX1 (TCF3 PBX1)
LYMPHOID LEUKEMIA, NOS	982	9823/3	Chronic lymphocytic leukemia/small lymphocytic lymphoma
PROLYMPH/PRECURS LEUKEMIA	983	9831/3	T-cell large granular lymphocytic leukemia
		9837/3	T lymphoblastic leukemia/lymphoma
CHRONIC MYELOPROLIFERATIVE DIS.	996	9965/3	Myeloid and lymphoid neoplasms with PDGFRB rearrangement
		9967/3	Myeloid and lymphoid neoplasm with FGFR1 abnormalities
MYELOPLASTIC/MYELOPROLIFERATIVE NEOPLASMS	997	9971/3	Polymorphic PTLD
		9975/3	Myelodysplastic/Myeloproliferative neoplasm, unclassifiable

UNSPECIFIED DIGEST. ORGANS C260,C268-C269

NEOPLASM	800	8000/3	Neoplasm, malignant
		8001/3	Tumor cells, malignant
		8002/3	Malignant tumor, small cell type
		8003/3	Malignant tumor, giant cell type
		8004/3	Malignant tumor, spindle cell type
		8005/3	Malignant tumor, clear cell type
CARCINOMA, NOS	801	8010/2	Carcinoma in situ, NOS
		8010/3	Carcinoma, NOS
		8011/3	Epithelioma, malignant
		8012/3	Large cell carcinoma, NOS
		8013/3	Large cell neuroendocrine carcinoma
		8014/3	Large cell carcinoma with rhabdoid phenotype
		8015/3	Glassy cell carcinoma
CARCINOMA, UNDIFF., NOS	802	8020/3	Carcinoma, undifferentiated type, NOS
		8021/3	Carcinoma, anaplastic type, NOS
		8022/3	Pleomorphic carcinoma
GIANT & SPINDLE CELL CARCINOMA	803	8030/3	Giant cell and spindle cell carcinoma
		8031/3	Giant cell carcinoma
		8032/3	Spindle cell carcinoma
		8033/3	Pseudosarcomatous carcinoma
		8034/3	Polygonal cell carcinoma
		8035/3	Carcinoma with osteoclast-like giant cells
SMALL CELL CARCINOMA, NOS	804	8041/3	Small cell carcinoma, NOS
		8043/3	Small cell carcinoma, fusiform cell
PAPILLARY CARCINOMA, NOS	805	8050/2	Papillary carcinoma in situ
		8050/3	Papillary carcinoma, NOS
		8051/3	Verrucous carcinoma, NOS
		8052/2	Papillary squamous cell carcinoma, non-invasive
		8052/3	Papillary squamous cell carcinoma
SQUAMOUS CELL CARCINOMA, NOS	807	8070/2	Squamous cell carcinoma in situ, NOS
		8070/3	Squamous cell carcinoma, NOS
		8071/3	Sq. cell carcinoma, keratinizing, NOS
		8072/3	Sq. cell carcinoma, lg. cell, non-ker.
		8073/3	Sq. cell carcinoma, sm. cell, non-ker.
		8074/3	Sq. cell carcinoma, spindle cell
		8075/3	Squamous cell carcinoma, adenoid
		8076/2	Sq. cell carc. in situ with question. stromal invas.
		8076/3	Sq. cell carcinoma, micro-invasive
		8078/3	Squamous cell carcinoma with horn formation

UNSPECIFIED DIGEST. ORGANS C260,C268-C269

LYMPHOEPITHELIAL CARCINOMA	808	8082/3	Lymphoepithelial carcinoma
		8083/3	Basaloid squamous cell carcinoma
		8084/3	Squamous cell carcinoma, clear cell type
TRANSITIONAL CELL CARCINOMA, NOS	812	8120/2	Transitional cell carcinoma in situ
		8120/3	Transitional cell carcinoma, NOS
		8121/3	Schneiderian carcinoma
		8122/3	Trans. cell carcinoma, spindle cell
		8123/3	Basaloid carcinoma
		8124/3	Cloacogenic carcinoma
PAPILLARY TRANS. CELL CARCINOMA	813	8130/2	Papillary trans. cell carcinoma, non-invasive
		8130/3	Papillary trans. cell carcinoma
		8131/3	Transitional cell carcinoma, micropapillary
ADENOCARCINOMA, NOS	814	8140/2	Adenocarcinoma in situ
		8140/3	Adenocarcinoma, NOS
		8141/3	Scirrhous adenocarcinoma
		8143/3	Superficial spreading adenocarcinoma
		8147/3	Basal cell adenocarcinoma
ENDOCRINOMAS	815	8152/3	Glucagonoma, malignant
		8153/3	Gastrinoma, malignant
		8156/3	Somatostatinoma, malignant
		8157/3	Enteroglucagonoma, malignant
TRABECULAR ADENOCARCINOMA	819	8190/3	Trabecular adenocarcinoma
ADENOID CYSTIC & CRIBRIFORM CA.	820	8200/3	Adenoid cystic carcinoma
		8201/2	Cribriform carcinoma in situ
		8201/3	Cribriform carcinoma
ADENOCA. IN ADENOMA. POLYP	821	8210/2	Adenocarcinoma in situ in adenomatous polyp
		8210/3	Adenocarcinoma in adenomatous polyp
		8211/3	Tubular adenocarcinoma
SOLID CARCINOMA, NOS	823	8230/2	Duct carcinoma in situ, solid type
		8230/3	Solid carcinoma, NOS
		8231/3	Carcinoma simplex
CARCINOID TUMOR, MALIGNANT	824	8240/3	Carcinoid tumor, malignant
		8241/3	Enterochromaffin cell carcinoid
		8242/3	Enterochromaffin-like cell tumor, malignant
		8243/3	Goblet cell carcinoid
		8244/3	Composite carcinoid
		8245/3	Adenocarcinoid tumor
		8246/3	Neuroendocrine carcinoma

UNSPECIFIED DIGEST. ORGANS C260,C268-C269

CARCINOID TUMOR, MALIGNANT	824	8249/3	Atypical carcinoid tumor
BRONCHIOLO-ALVEOLAR ADENOCA.	825	8251/3	Alveolar adenocarcinoma
		8255/3	Adenocarcinoma with mixed subtypes
PAPILLARY ADENOCARCINOMA, NOS	826	8260/3	Papillary adenocarcinoma, NOS
		8261/2	Adenocarcinoma in situ in villous adenoma
		8261/3	Adenocarcinoma in villous adenoma
		8262/3	Villous adenocarcinoma
		8263/2	Adenocarcinoma in situ in tubulovillous adenoma
		8263/3	Adenocarcinoma in tubulovillous adenoma
OXYPHILIC ADENOCARCINOMA	829	8290/3	Oxyphilic adenocarcinoma
CLEAR CELL ADENOCARCINOMA, NOS	831	8310/3	Clear cell adenocarcinoma, NOS
GRANULAR CELL CARCINOMA	832	8320/3	Granular cell carcinoma
		8323/3	Mixed cell adenocarcinoma
ENDOMETRIOID ADENOCARCINOMA	838	8380/3	Endometrioid carcinoma
SWEAT GLAND ADENOCARCINOMA	840	8401/3	Apocrine adenocarcinoma
MUCOEPIDERMOID CARCINOMA	843	8430/3	Mucoepidermoid carcinoma
CYSTADENOCARCINOMA, NOS	844	8440/3	Cystadenocarcinoma, NOS
MUCINOUS ADENOCARCINOMA	848	8480/3	Mucinous adenocarcinoma
		8481/3	Mucin-producing adenocarcinoma
SIGNET RING CELL CARCINOMA	849	8490/3	Signet ring cell carcinoma
DUCT CARCINOMA	850	8503/2	Noninfiltrating intraductal papillary adenocarcinoma
		8503/3	Intraductal papillary adenocarcinoma with invasion
		8504/2	Noninfiltrating intracystic carcinoma
		8504/3	Intracystic carcinoma, NOS
MEDULLARY CARCINOMA, NOS	851	8510/3	Medullary carcinoma, NOS
LOBULAR AND OTHER DUCTAL CA.	852	8521/3	Infiltrating ductular carcinoma
PAGET DISEASE, EXTRAMAMMARY	854	8542/3	Paget disease, extramammary
ACINAR CELL CARCINOMA	855	8550/3	Acinar cell carcinoma
		8551/3	Acinar cell cystadenocarcinoma

UNSPECIFIED DIGEST. ORGANS C260,C268-C269

ADENOSQUAMOUS CARCINOMA	856	8560/3	Adenosquamous carcinoma
		8562/3	Epithelial-myoepithelial carcinoma
ADENOCA. WITH METAPLASIA	857	8570/3	Adenocarcinoma with squamous metaplasia
		8571/3	Adenocarcinoma w cartilag. & oss. metaplas.
		8572/3	Adenocarcinoma with spindle cell mataplasia
		8573/3	Adenocarcinoma with apocrine metaplasia
		8574/3	Adenocarcinoma with neuroendocrine differen.
		8575/3	Metaplastic carcinoma, NOS
		8576/3	Hepatoid adenocarcinoma
EXTRA-ADRENAL PARAGANG., MAL	869	8693/3	Extra-adrenal paraganglioma, malignant
NEVI & MELANOMAS	872	8720/2	Melanoma in situ
		8720/3	Malignant melanoma, NOS
		8721/3	Nodular melanoma
		8722/3	Balloon cell melanoma
		8723/3	Malignant melanoma, regressing
AMELANOTIC MELANOMA	873	8730/3	Amelanotic melanoma
MAL. MEL. IN JUNCT. NEVUS	874	8743/3	Superficial spreading melanoma
		8745/3	Desmoplastic melanoma, malignant
		8746/3	Mucosal lentiginous melanoma
EPITHELIOID CELL MELANOMA	877	8770/3	Mixed epithel. & spindle cell melanoma
		8771/3	Epithelioid cell melanoma
		8772/3	Spindle cell melanoma, NOS
SARCOMA, NOS	880	8800/3	Sarcoma, NOS
		8801/3	Spindle cell sarcoma
		8802/3	Giant cell sarcoma
		8803/3	Small cell sarcoma
		8804/3	Epithelioid sarcoma
		8805/3	Undifferentiated sarcoma
		8806/3	Desmoplastic small round cell tumor
FIBROMATOUS NEOPLASMS	881	8810/3	Fibrosarcoma, NOS
		8811/3	Fibromyxosarcoma
		8813/3	Fascial fibrosarcoma
		8814/3	Infantile fibrosarcoma
		8815/3	Solitary fibrous tumor, malignant
SARCOMA, NOS	882	8825/3	Myofibroblastic sarcoma
FIBROUS HISTIOCYTOMA, MAL.	883	8830/3	Fibrous histiocytoma, malignant

UNSPECIFIED DIGEST. ORGANS C260,C268-C269

MYXOSARCOMA	884	8840/3	Myxosarcoma
LIPOSARCOMA NEOPLASMS	885	8850/3	Liposarcoma, NOS
		8851/3	Liposarcoma, well differentiated
		8852/3	Myxoid liposarcoma
		8853/3	Round cell liposarcoma
		8854/3	Pleomorphic liposarcoma
		8855/3	Mixed type liposarcoma
		8857/3	Fibroblastic liposarcoma
		8858/3	Dedifferentiated liposarcoma
MYOMATOUS NEOPLASMS	889	8890/3	Leiomyosarcoma, NOS
		8891/3	Epithelioid leiomyosarcoma
		8894/3	Angiomyosarcoma
		8895/3	Myosarcoma
		8896/3	Myxoid leiomyosarcoma
RHABDOMYOSARCOMA, NOS	890	8900/3	Rhabdomyosarcoma, NOS
		8901/3	Pleomorphic rhabdomyosarcoma, adult type
		8902/3	Mixed type rhabdomyosarcoma
EMBRYONAL RHABDOMYOSARCOMA	891	8910/3	Embryonal rhabdomyosarcoma
		8912/3	Spindle cell rhabdomyosarcoma
ALVEOLAR RHABDOMYOSARCOMA	892	8920/3	Alveolar rhabdomyosarcoma
		8921/3	Rhabdomyosarcoma with ganglionic differentiation
STROMAL SARCOMA	893	8935/3	Stromal sarcoma, NOS
		8936/3	Gastrointestinal stromal sarcoma
MIXED TUMOR, MALIGNANT, NOS	894	8940/3	Mixed tumor, malignant, NOS
		8941/3	Carcinoma in pleomorphic adenoma
MULLERIAN MIXED TUMOR	895	8950/3	Mullerian mixed tumor
		8951/3	Mesodermal mixed tumor
CARCINOSARCOMA, NOS	898	8980/3	Carcinosarcoma, NOS
		8981/3	Carcinosarcoma, embryonal type
		8982/3	Malignant myoepithelioma
MESENCHYMOMA, MALIGNANT	899	8990/3	Mesenchymoma, malignant
		8991/3	Embryonal sarcoma

UNSPECIFIED DIGEST. ORGANS C260,C268-C269

SYNOVIAL SARCOMA, NOS	904	9040/3	Synovial sarcoma, NOS
		9041/3	Synovial sarcoma, spindle cell
		9042/3	Synovial sarcoma, epithelioid cell
		9043/3	Synovial sarcoma, biphasic
		9044/3	Clear cell sarcoma,NOS (except of kidney M-8964/3)
GERM CELL TUMORS	906	9060/3	Dysgerminoma
		9064/3	Germinoma
		9065/3	Germ cell tumor, nonseminomatous
EMBRYONAL CARCINOMA, NOS	907	9070/3	Embryonal carcinoma, NOS
		9071/3	Yolk sac tumor
		9072/3	Polyembryoma
TERATOMA	908	9080/3	Teratoma, malignant, NOS
		9081/3	Teratocarcinoma
		9082/3	Malignant teratoma, undiff.
		9083/3	Malignant teratoma, intermediate
		9084/3	Teratoma with malig. transformation
		9085/3	Mixed germ cell tumor
CHORIOCARCINOMA	910	9100/3	Choriocarcinoma
		9101/3	Choriocarcinoma combined w/ other germ cell elements
		9105/3	Trophoblastic tumor, epithelioid
MESONEPHROMA, MALIGNANT	911	9110/3	Mesonephroma, malignant
BLOOD VESSEL TUMORS	912	9120/3	Hemangiosarcoma
HEMANGIOENDOTHELIOMA	913	9130/3	Hemangioendothelioma, malignant
		9133/3	Epithelioid hemangioendothelioma, malignant
KAPOSI SARCOMA	914	9140/3	Kaposi sarcoma
HEMANGIOPERICYTOMA	915	9150/3	Hemangiopericytoma, malignant
LYMPHANGIOSARCOMA	917	9170/3	Lymphangiosarcoma
GT. CELL TUMOR OF BONE, MAL.	925	9251/3	Malignant giant cell tumor of soft parts
		9252/3	Malignant tenosynovial giant cell tumor
CHORDOMA	937	9370/3	Chordoma, NOS
		9371/3	Chondroid chordoma
		9372/3	Dedifferentiated chordoma
GANGLIONEUROBLASTOMA	949	9490/3	Ganglioneuroblastoma

UNSPECIFIED DIGEST. ORGANS C260,C268-C269

NEUROBLASTOMA, NOS	950	9500/3	Neuroblastoma, NOS
		9501/3	Medulloepithelioma, NOS
		9502/3	Teratoid medulloepithelioma
		9503/3	Neuroepithelioma, NOS
		9504/3	Spongioneuroblastoma
		9505/3	Ganglioglioma, anaplastic
NEUROFIBROSARCOMA	954	9540/3	Malignant peripheral nerve sheath tumor
NEURILEMMOMA	956	9560/3	Neurilemmoma, malignant
		9561/3	MPNST with rhabdomyoblastic differentiation
PERINEURIOMA	957	9571/3	Perineurioma, malignant
MALIGNANT LYMPHOMA, NOS	959	9590/3	Malignant lymphoma, NOS
		9591/3	Malignant lymphoma, non-Hodgkin
		9596/3	Composite Hodgkin and non-Hodgkin lymphoma
HODGKIN LYMPHOMA	965	9650/3	Hodgkin lymphoma, NOS
		9651/3	Hodgkin lymphoma, lymphocyte-rich
		9652/3	Hodgkin lymphoma, mixed cellularity, NOS
		9653/3	Hodgkin lymphoma, lymphocytic deplet., NOS
		9654/3	Hodgkin lymph., lymphocyt. deplet., diffuse fibrosis
		9655/3	Hodgkin lymphoma, lymphocyt. deplet., reticular
		9659/3	Hodgkin lymph., nodular lymphocyte predom.
HODGKIN LYMPHOMA, NOD. SCLER.	966	9661/3	Hodgkin granuloma [obs]
		9662/3	Hodgkin sarcoma [obs]
		9663/3	Hodgkin lymphoma, nodular sclerosis, NOS
		9664/3	Hodgkin lymphoma, nod. scler., cellular phase
		9665/3	Hodgkin lymphoma, nod. scler., grade 1
		9667/3	Hodgkin lymphoma, nod. scler., grade 2
ML, SMALL B-CELL LYMPHOCYTIC	967	9670/3	ML, small B lymphocytic, NOS
		9671/3	ML, lymphoplasmacytic
		9673/3	Mantle cell lymphoma
		9675/3	ML, mixed sm. and lg. cell, diffuse
ML, LARGE B-CELL, DIFFUSE	968	9680/3	ML, large B-cell, diffuse
		9684/3	ML, large B-cell, diffuse, immunoblastic, NOS
		9687/3	Burkitt lymphoma, NOS
		9688/3	T-cell histiocyte rich large B-cell lymphoma

UNSPECIFIED DIGEST. ORGANS C260,C268-C269

FOLLIC. & MARGINAL LYMPH, NOS	969	9690/3	Follicular lymphoma, NOS
		9691/3	Follicular lymphoma, grade 2
		9695/3	Follicular lymphoma, grade 1
		9698/3	Follicular lymphoma, grade 3
		9699/3	Marginal zone B-cell lymphoma, NOS
T-CELL LYMPHOMAS	970	9701/3	Sezary syndrome
		9702/3	Mature T-cell lymphoma, NOS
		9705/3	Angioimmunoblastic T-cell lymphoma
OTHER SPEC. NON-HODGKIN LYMPHOMA	971	9712/3	Intravascular large B-cell lymphoma
		9714/3	Anaplastic large cell lymphoma, T-cell and Null cell type
		9716/3	Hepatosplenic gamma-delta cell lymphoma
		9717/3	Intestinal T-cell lymphoma
		9719/3	NK/T-cell lymphoma, nasal and nasal-type
PRECURS. CELL LYMPHOBLASTIC LYMPH.	972	9724/3	SystemicEBV pos. T-cell lymphoproliferative disease of childhood
		9727/3	Precursor cell lymphoblastic lymphoma, NOS
		9728/3	Precursor B-cell lymphoblastic lymphoma
		9729/3	Precursor T-cell lymphoblastic lymphoma
PLASMA CELL TUMORS	973	9731/3	Plasmacytoma, NOS
		9734/3	Plasmacytoma, extramedullary
		9735/3	Plasmablastic lymphoma
		9737/3	ALK positive large B-cell lymphoma
		9738/3	Lrg B-cell lymphoma in HHV8-assoc. multicentric Castleman DZ
MAST CELL TUMORS	974	9740/3	Mast cell sarcoma
		9741/3	Malignant mastocytosis
NEOPLASMS OF HISTIOCYTES AND ACCESSORY LYMPHOID CELLS	975	9750/3	Malignant histiocytosis
		9751/3	Langerhans cell histiocytosis, NOS
		9754/3	Langerhans cell histiocytosis, disseminated
		9755/3	Histiocytic sarcoma
		9756/3	Langerhans cell sarcoma
		9757/3	Interdigitating dendritic cell sarcoma
		9758/3	Follicular dendritic cell sarcoma
		9759/3	Fibroblastic reticular cell tumor
PRECURSOR LYMPHOID NEOPLASMS	981	9811/3	B lymphoblastic leukemia/lymphoma, NOS
		9812/3	Leukemia/lymphoma with t(9;22)(q34;q11.2);BCR-ABL1
		9813/3	Leukemia/lymphoma with t(v;11q23);MLL rearranged
		9814/3	Leukemia/lymphoma with t(12;21)(p13;q22);TEL-AML1(ETV6-RUNX1)
		9815/3	B lymphoblastic leukemia/lymphoma with hyperdiploidy
		9816/3	Leukemia/lymphoma with hypodiploidy (hypodiploid ALL)
		9817/3	B lymphblastic leukemia/lymphoma with t(5;14)(q31;q32);IL3-IGH
		9818/3	Leukemia/lymphoma with t(1;19)(q23;p13.3); E2A PBX1 (TCF3 PBX1)

UNSPECIFIED DIGEST. ORGANS C260,C268-C269

LYMPHOID LEUKEMIA, NOS	982	9823/3	Chronic lymphocytic leukemia/small lymphocytic lymphoma
PROLYMPH/PRECURS LEUKEMIA	983	9831/3	T-cell large granular lymphocytic leukemia
		9837/3	T lymphoblastic leukemia/lymphoma
CHRONIC MYELOPROLIFERATIVE DIS.	996	9965/3	Myeloid and lymphoid neoplasms with PDGFRB rearrangement
		9967/3	Myeloid and lymphoid neoplasm with FGFR1 abnormalities
MYELOPLASTIC/MYELOPROLIFERATIVE NEOPLASMS	997	9971/3	Polymorphic PTLD
		9975/3	Myelodysplastic/Myeloproliferative neoplasm, unclassifiable

NASAL CAVITY (INCLUDING NASAL CARTILAGE) C300

NEOPLASM	800	8000/3	Neoplasm, malignant
		8001/3	Tumor cells, malignant
		8002/3	Malignant tumor, small cell type
		8003/3	Malignant tumor, giant cell type
		8004/3	Malignant tumor, spindle cell type
		8005/3	Malignant tumor, clear cell type
CARCINOMA, NOS	801	8010/2	Carcinoma in situ, NOS
		8010/3	Carcinoma, NOS
		8011/3	Epithelioma, malignant
		8012/3	Large cell carcinoma, NOS
		8013/3	Large cell neuroendocrine carcinoma
		8014/3	Large cell carcinoma with rhabdoid phenotype
		8015/3	Glassy cell carcinoma
CARCINOMA, UNDIFF., NOS	802	8020/3	Carcinoma, undifferentiated type, NOS
		8021/3	Carcinoma, anaplastic type, NOS
		8022/3	Pleomorphic carcinoma
		8023/3	NUT carcinoma
GIANT & SPINDLE CELL CARCINOMA	803	8030/3	Giant cell and spindle cell carcinoma
		8031/3	Giant cell carcinoma
		8032/3	Spindle cell carcinoma
		8033/3	Pseudosarcomatous carcinoma
		8034/3	Polygonal cell carcinoma
		8035/3	Carcinoma with osteoclast-like giant cells
PAPILLARY CARCINOMA, NOS	805	8050/2	Papillary carcinoma in situ
		8050/3	Papillary carcinoma, NOS
		8051/3	Verrucous carcinoma, NOS
		8052/2	Papillary squamous cell carcinoma, non-invasive
		8052/3	Papillary squamous cell carcinoma
SQUAMOUS CELL CARCINOMA, NOS	807	8070/2	Squamous cell carcinoma in situ, NOS
		8070/3	Squamous cell carcinoma, NOS
		8071/3	Sq. cell carcinoma, keratinizing, NOS
		8072/3	Sq. cell carcinoma, lg. cell, non-ker.
		8073/3	Sq. cell carcinoma, sm. cell, non-ker.
		8074/3	Sq. cell carcinoma, spindle cell
		8075/3	Squamous cell carcinoma, adenoid
		8076/2	Sq. cell carc. in situ with question. stromal invas.
		8076/3	Sq. cell carcinoma, micro-invasive
		8078/3	Squamous cell carcinoma with horn formation

NASAL CAVITY (INCLUDING NASAL CARTILAGE) C300

LYMPHOEPITHELIAL CARCINOMA	808	8082/3	Lymphoepithelial carcinoma
		8083/3	Basaloid squamous cell carcinoma
		8084/3	Squamous cell carcinoma, clear cell type
TRANSITIONAL CELL CARCINOMA, NOS	812	8120/2	Transitional cell carcinoma in situ
		8120/3	Transitional cell carcinoma, NOS
		8121/3	Schneiderian carcinoma
		8122/3	Trans. cell carcinoma, spindle cell
		8123/3	Basaloid carcinoma
		8124/3	Cloacogenic carcinoma
PAPILLARY TRANS. CELL CARCINOMA	813	8130/2	Papillary trans. cell carcinoma, non-invasive
		8130/3	Papillary trans. cell carcinoma
		8131/3	Transitional cell carcinoma, micropapillary
ADENOCARCINOMA, NOS	814	8140/2	Adenocarcinoma in situ
		8140/3	Adenocarcinoma, NOS
		8141/3	Scirrhous adenocarcinoma
		8143/3	Superficial spreading adenocarcinoma
		8147/3	Basal cell adenocarcinoma
ADENOID CYSTIC & CRIBRIFORM CA.	820	8200/3	Adenoid cystic carcinoma
		8201/2	Cribriform carcinoma in situ
		8201/3	Cribriform carcinoma
BRONCHIOLO-ALVEOLAR ADENOCA.	825	8255/3	Adenocarcinoma with mixed subtypes
PAPILLARY ADENOCARCINOMA, NOS	826	8260/3	Papillary adenocarcinoma, NOS
		8261/2	Adenocarcinoma in situ in villous adenoma
		8261/3	Adenocarcinoma in villous adenoma
		8262/3	Villous adenocarcinoma
		8263/2	Adenocarcinoma in situ in tubulovillous adenoma
		8263/3	Adenocarcinoma in tubulovillous adenoma
MUCOEPIDERMOID CARCINOMA	843	8430/3	Mucoepidermoid carcinoma
MUCINOUS ADENOCARCINOMA	848	8480/3	Mucinous adenocarcinoma
		8481/3	Mucin-producing adenocarcinoma
ADENOSQUAMOUS CARCINOMA	856	8560/3	Adenosquamous carcinoma
		8562/3	Epithelial-myoepithelial carcinoma
ADENOCA. WITH METAPLASIA	857	8570/3	Adenocarcinoma with squamous metaplasia
		8571/3	Adenocarcinoma w cartilag. & oss. metaplas.
		8572/3	Adenocarcinoma with spindle cell mataplasia
		8573/3	Adenocarcinoma with apocrine metaplasia
		8574/3	Adenocarcinoma with neuroendocrine differen.

NASAL CAVITY (INCLUDING NASAL CARTILAGE) C300

ADENOCA. WITH METAPLASIA	857	8575/3	Metaplastic carcinoma, NOS
NEVI & MELANOMAS	872	8720/2	Melanoma in situ
		8720/3	Malignant melanoma, NOS
		8721/3	Nodular melanoma
		8722/3	Balloon cell melanoma
		8723/3	Malignant melanoma, regressing
AMELANOTIC MELANOMA	873	8730/3	Amelanotic melanoma
MAL. MEL. IN JUNCT. NEVUS	874	8743/3	Superficial spreading melanoma
		8745/3	Desmoplastic melanoma, malignant
		8746/3	Mucosal lentiginous melanoma
EPITHELIOID CELL MELANOMA	877	8770/3	Mixed epithel. & spindle cell melanoma
		8771/3	Epithelioid cell melanoma
		8772/3	Spindle cell melanoma, NOS
SARCOMA, NOS	880	8800/3	Sarcoma, NOS
		8801/3	Spindle cell sarcoma
		8802/3	Giant cell sarcoma
		8803/3	Small cell sarcoma
		8804/3	Epithelioid sarcoma
		8805/3	Undifferentiated sarcoma
		8806/3	Desmoplastic small round cell tumor
FIBROMATOUS NEOPLASMS	881	8810/3	Fibrosarcoma, NOS
		8811/3	Fibromyxosarcoma
		8813/3	Fascial fibrosarcoma
		8814/3	Infantile fibrosarcoma
		8815/3	Solitary fibrous tumor, malignant
SARCOMA, NOS	882	8825/3	Myofibroblastic sarcoma
FIBROUS HISTIOCYTOMA, MAL.	883	8830/3	Fibrous histiocytoma, malignant
MYOMATOUS NEOPLASMS	889	8890/3	Leiomyosarcoma, NOS
		8891/3	Epithelioid leiomyosarcoma
		8894/3	Angiomyosarcoma
		8895/3	Myosarcoma
		8896/3	Myxoid leiomyosarcoma
RHABDOMYOSARCOMA, NOS	890	8900/3	Rhabdomyosarcoma, NOS
		8901/3	Pleomorphic rhabdomyosarcoma, adult type
		8902/3	Mixed type rhabdomyosarcoma

NASAL CAVITY (INCLUDING NASAL CARTILAGE) C300

EMBRYONAL RHABDOMYOSARCOMA	891	8910/3	Embryonal rhabdomyosarcoma
		8912/3	Spindle cell rhabdomyosarcoma
ALVEOLAR RHABDOMYOSARCOMA	892	8920/3	Alveolar rhabdomyosarcoma
		8921/3	Rhabdomyosarcoma with ganglionic differentiation
MIXED TUMOR, MALIGNANT, NOS	894	8940/3	Mixed tumor, malignant, NOS
		8941/3	Carcinoma in pleomorphic adenoma
SYNOVIAL SARCOMA, NOS	904	9045/3	Biphenotypic sinonasal sarcoma
CHONDROSARCOMA, NOS	922	9220/3	Chondrosarcoma, NOS
		9221/3	Juxtacortical chondrosarcoma
CHORDOMA	937	9370/3	Chordoma, NOS
		9371/3	Chondroid chordoma
		9372/3	Dedifferentiated chordoma
NEUROBLASTOMA, NOS	950	9500/3	Neuroblastoma, NOS
		9501/3	Medulloepithelioma, NOS
		9502/3	Teratoid medulloepithelioma
		9503/3	Neuroepithelioma, NOS
		9504/3	Spongioneuroblastoma
		9505/3	Ganglioglioma, anaplastic
OLFACTORY NEUROGENIC TUMOR	952	9520/3	Olfactory neurogenic tumor
		9521/3	Olfactory neurcytoma
		9522/3	Olfactory neuroblastoma
		9523/3	Olfactory neuroepithelioma
NEUROFIBROSARCOMA	954	9540/3	Malignant peripheral nerve sheath tumor
NEURILEMMOMA	956	9560/3	Neurilemmoma, malignant
		9561/3	MPNST with rhabdomyoblastic differentiation
MALIGNANT LYMPHOMA, NOS	959	9590/3	Malignant lymphoma, NOS
		9591/3	Malignant lymphoma, non-Hodgkin
		9596/3	Composite Hodgkin and non-Hodgkin lymphoma
HODGKIN LYMPHOMA	965	9650/3	Hodgkin lymphoma, NOS
		9651/3	Hodgkin lymphoma, lymphocyte-rich
		9652/3	Hodgkin lymphoma, mixed cellularity, NOS
		9653/3	Hodgkin lymphoma, lymphocytic deplet., NOS
		9654/3	Hodgkin lymph., lymphocyt. deplet., diffuse fibrosis
		9655/3	Hodgkin lymphoma, lymphocyt. deplet., reticular
		9659/3	Hodgkin lymph., nodular lymphocyte predom.

NASAL CAVITY (INCLUDING NASAL CARTILAGE) C300

HODGKIN LYMPHOMA, NOD. SCLER.	966	9661/3	Hodgkin granuloma [obs]
		9662/3	Hodgkin sarcoma [obs]
		9663/3	Hodgkin lymphoma, nodular sclerosis, NOS
		9664/3	Hodgkin lymphoma, nod. scler., cellular phase
		9665/3	Hodgkin lymphoma, nod. scler., grade 1
		9667/3	Hodgkin lymphoma, nod. scler., grade 2
ML, SMALL B-CELL LYMPHOCYTIC	967	9670/3	ML, small B lymphocytic, NOS
		9671/3	ML, lymphoplasmacytic
		9673/3	Mantle cell lymphoma
		9675/3	ML, mixed sm. and lg. cell, diffuse
ML, LARGE B-CELL, DIFFUSE	968	9680/3	ML, large B-cell, diffuse
		9684/3	ML, large B-cell, diffuse, immunoblastic, NOS
		9687/3	Burkitt lymphoma, NOS
		9688/3	T-cell histiocyte rich large B-cell lymphoma
FOLLIC. & MARGINAL LYMPH, NOS	969	9690/3	Follicular lymphoma, NOS
		9691/3	Follicular lymphoma, grade 2
		9695/3	Follicular lymphoma, grade 1
		9698/3	Follicular lymphoma, grade 3
		9699/3	Marginal zone B-cell lymphoma, NOS
T-CELL LYMPHOMAS	970	9701/3	Sezary syndrome
		9702/3	Mature T-cell lymphoma, NOS
		9705/3	Angioimmunoblastic T-cell lymphoma
OTHER SPEC. NON-HODGKIN LYMPHOMA	971	9712/3	Intravascular large B-cell lymphoma
		9714/3	Anaplastic large cell lymphoma, T-cell and Null cell type
		9719/3	NK/T-cell lymphoma, nasal and nasal-type
PRECURS. CELL LYMPHOBLASTIC LYMPH.	972	9724/3	SystemicEBV pos. T-cell lymphoproliferative disease of childhood
		9727/3	Precursor cell lymphoblastic lymphoma, NOS
		9728/3	Precursor B-cell lymphoblastic lymphoma
		9729/3	Precursor T-cell lymphoblastic lymphoma
PLASMA CELL TUMORS	973	9731/3	Plasmacytoma, NOS
		9734/3	Plasmacytoma, extramedullary
		9735/3	Plasmablastic lymphoma
		9737/3	ALK positive large B-cell lymphoma
		9738/3	Lrg B-cell lymphoma in HHV8-assoc. multicentric Castleman DZ
MAST CELL TUMORS	974	9740/3	Mast cell sarcoma
		9741/3	Malignant mastocytosis

NASAL CAVITY (INCLUDING NASAL CARTILAGE) C300

NEOPLASMS OF HISTIOCYTES AND ACCESSORY LYMPHOID CELLS	975	9750/3	Malignant histiocytosis
		9751/3	Langerhans cell histiocytosis, NOS
		9754/3	Langerhans cell histiocytosis, disseminated
		9755/3	Histiocytic sarcoma
		9756/3	Langerhans cell sarcoma
		9757/3	Interdigitating dendritic cell sarcoma
		9758/3	Follicular dendritic cell sarcoma
		9759/3	Fibroblastic reticular cell tumor
PRECURSOR LYMPHOID NEOPLASMS	981	9811/3	B lymphoblastic leukemia/lymphoma, NOS
		9812/3	Leukemia/lymphoma with t(9;22)(q34;q11.2);BCR-ABL1
		9813/3	Leukemia/lymphoma with t(v;11q23);MLL rearranged
		9814/3	Leukemia/lymphoma with t(12;21)(p13;q22);TEL-AML1(ETV6-RUNX1)
		9815/3	B lymphoblastic leukemia/lymphoma with hyperdiploidy
		9816/3	Leukemia/lymphoma with hypodiploidy (hypodiploid ALL)
		9817/3	B lymphblastic leukemia/lymphoma with t(5;14)(q31;q32);IL3-IGH
		9818/3	Leukemia/lymphoma with t(1;19)(q23;p13.3); E2A PBX1 (TCF3 PBX1)
LYMPHOID LEUKEMIA, NOS	982	9823/3	Chronic lymphocytic leukemia/small lymphocytic lymphoma
PROLYMPH/PRECURS LEUKEMIA	983	9831/3	T-cell large granular lymphocytic leukemia
		9837/3	T lymphoblastic leukemia/lymphoma
CHRONIC MYELOPROLIFERATIVE DIS.	996	9965/3	Myeloid and lymphoid neoplasms with PDGFRB rearrangement
		9967/3	Myeloid and lymphoid neoplasm with FGFR1 abnormalities
MYELOPLASTIC/MYELOPROLIFERATIVE NEOPLASMS	997	9971/3	Polymorphic PTLD
		9975/3	Myelodysplastic/Myeloproliferative neoplasm, unclassifiable

MIDDLE EAR C301

NEOPLASM	800	8000/3	Neoplasm, malignant
		8001/3	Tumor cells, malignant
		8002/3	Malignant tumor, small cell type
		8003/3	Malignant tumor, giant cell type
		8004/3	Malignant tumor, spindle cell type
		8005/3	Malignant tumor, clear cell type
CARCINOMA, NOS	801	8010/2	Carcinoma in situ, NOS
		8010/3	Carcinoma, NOS
		8011/3	Epithelioma, malignant
		8012/3	Large cell carcinoma, NOS
		8013/3	Large cell neuroendocrine carcinoma
		8014/3	Large cell carcinoma with rhabdoid phenotype
		8015/3	Glassy cell carcinoma
CARCINOMA, UNDIFF., NOS	802	8020/3	Carcinoma, undifferentiated type, NOS
		8021/3	Carcinoma, anaplastic type, NOS
		8022/3	Pleomorphic carcinoma
GIANT & SPINDLE CELL CARCINOMA	803	8030/3	Giant cell and spindle cell carcinoma
		8031/3	Giant cell carcinoma
		8032/3	Spindle cell carcinoma
		8033/3	Pseudosarcomatous carcinoma
		8034/3	Polygonal cell carcinoma
		8035/3	Carcinoma with osteoclast-like giant cells
PAPILLARY CARCINOMA, NOS	805	8050/2	Papillary carcinoma in situ
		8050/3	Papillary carcinoma, NOS
		8051/3	Verrucous carcinoma, NOS
		8052/2	Papillary squamous cell carcinoma, non-invasive
		8052/3	Papillary squamous cell carcinoma
SQUAMOUS CELL CARCINOMA, NOS	807	8070/2	Squamous cell carcinoma in situ, NOS
		8070/3	Squamous cell carcinoma, NOS
		8071/3	Sq. cell carcinoma, keratinizing, NOS
		8072/3	Sq. cell carcinoma, lg. cell, non-ker.
		8073/3	Sq. cell carcinoma, sm. cell, non-ker.
		8074/3	Sq. cell carcinoma, spindle cell
		8075/3	Squamous cell carcinoma, adenoid
		8076/2	Sq. cell carc. in situ with question. stromal invas.
		8076/3	Sq. cell carcinoma, micro-invasive
		8078/3	Squamous cell carcinoma with horn formation

MIDDLE EAR C301

LYMPHOEPITHELIAL CARCINOMA	808	8082/3	Lymphoepithelial carcinoma
		8083/3	Basaloid squamous cell carcinoma
		8084/3	Squamous cell carcinoma, clear cell type
TRANSITIONAL CELL CARCINOMA, NOS	812	8120/2	Transitional cell carcinoma in situ
		8120/3	Transitional cell carcinoma, NOS
		8121/3	Schneiderian carcinoma
		8122/3	Trans. cell carcinoma, spindle cell
		8123/3	Basaloid carcinoma
		8124/3	Cloacogenic carcinoma
PAPILLARY TRANS. CELL CARCINOMA	813	8130/2	Papillary trans. cell carcinoma, non-invasive
		8130/3	Papillary trans. cell carcinoma
		8131/3	Transitional cell carcinoma, micropapillary
ADENOCARCINOMA, NOS	814	8140/2	Adenocarcinoma in situ
		8140/3	Adenocarcinoma, NOS
		8141/3	Scirrhous adenocarcinoma
		8143/3	Superficial spreading adenocarcinoma
		8147/3	Basal cell adenocarcinoma
ADENOID CYSTIC & CRIBRIFORM CA.	820	8200/3	Adenoid cystic carcinoma
		8201/2	Cribriform carcinoma in situ
		8201/3	Cribriform carcinoma
BRONCHIOLO-ALVEOLAR ADENOCA.	825	8255/3	Adenocarcinoma with mixed subtypes
PAPILLARY ADENOCARCINOMA, NOS	826	8260/3	Papillary adenocarcinoma, NOS
		8261/2	Adenocarcinoma in situ in villous adenoma
		8261/3	Adenocarcinoma in villous adenoma
		8262/3	Villous adenocarcinoma
		8263/2	Adenocarcinoma in situ in tubulovillous adenoma
		8263/3	Adenocarcinoma in tubulovillous adenoma
MUCOEPIDERMOID CARCINOMA	843	8430/3	Mucoepidermoid carcinoma
MUCINOUS ADENOCARCINOMA	848	8480/3	Mucinous adenocarcinoma
		8481/3	Mucin-producing adenocarcinoma
ADENOSQUAMOUS CARCINOMA	856	8560/3	Adenosquamous carcinoma
		8562/3	Epithelial-myoepithelial carcinoma
ADENOCA. WITH METAPLASIA	857	8570/3	Adenocarcinoma with squamous metaplasia
		8571/3	Adenocarcinoma w cartilag. & oss. metaplas.
		8572/3	Adenocarcinoma with spindle cell mataplasia
		8573/3	Adenocarcinoma with apocrine metaplasia
		8574/3	Adenocarcinoma with neuroendocrine differen.

MIDDLE EAR C301

ADENOCA. WITH METAPLASIA	857	8575/3	Metaplastic carcinoma, NOS
NEVI & MELANOMAS	872	8720/2	Melanoma in situ
		8720/3	Malignant melanoma, NOS
		8721/3	Nodular melanoma
		8722/3	Balloon cell melanoma
		8723/3	Malignant melanoma, regressing
AMELANOTIC MELANOMA	873	8730/3	Amelanotic melanoma
MAL. MEL. IN JUNCT. NEVUS	874	8743/3	Superficial spreading melanoma
		8745/3	Desmoplastic melanoma, malignant
		8746/3	Mucosal lentiginous melanoma
EPITHELIOID CELL MELANOMA	877	8770/3	Mixed epithel. & spindle cell melanoma
		8771/3	Epithelioid cell melanoma
		8772/3	Spindle cell melanoma, NOS
SARCOMA, NOS	880	8800/3	Sarcoma, NOS
		8801/3	Spindle cell sarcoma
		8802/3	Giant cell sarcoma
		8803/3	Small cell sarcoma
		8804/3	Epithelioid sarcoma
		8805/3	Undifferentiated sarcoma
		8806/3	Desmoplastic small round cell tumor
FIBROMATOUS NEOPLASMS	881	8810/3	Fibrosarcoma, NOS
		8811/3	Fibromyxosarcoma
		8813/3	Fascial fibrosarcoma
		8814/3	Infantile fibrosarcoma
		8815/3	Solitary fibrous tumor, malignant
SARCOMA, NOS	882	8825/3	Myofibroblastic sarcoma
FIBROUS HISTIOCYTOMA, MAL.	883	8830/3	Fibrous histiocytoma, malignant
MYOMATOUS NEOPLASMS	889	8890/3	Leiomyosarcoma, NOS
		8891/3	Epithelioid leiomyosarcoma
		8894/3	Angiomyosarcoma
		8895/3	Myosarcoma
		8896/3	Myxoid leiomyosarcoma
RHABDOMYOSARCOMA, NOS	890	8900/3	Rhabdomyosarcoma, NOS
		8901/3	Pleomorphic rhabdomyosarcoma, adult type
		8902/3	Mixed type rhabdomyosarcoma

MIDDLE EAR C301

EMBRYONAL RHABDOMYOSARCOMA	891	8910/3	Embryonal rhabdomyosarcoma
		8912/3	Spindle cell rhabdomyosarcoma
ALVEOLAR RHABDOMYOSARCOMA	892	8920/3	Alveolar rhabdomyosarcoma
		8921/3	Rhabdomyosarcoma with ganglionic differentiation
MIXED TUMOR, MALIGNANT, NOS	894	8940/3	Mixed tumor, malignant, NOS
		8941/3	Carcinoma in pleomorphic adenoma
CHORDOMA	937	9370/3	Chordoma, NOS
		9371/3	Chondroid chordoma
		9372/3	Dedifferentiated chordoma
NEUROBLASTOMA, NOS	950	9500/3	Neuroblastoma, NOS
		9501/3	Medulloepithelioma, NOS
		9502/3	Teratoid medulloepithelioma
		9503/3	Neuroepithelioma, NOS
		9504/3	Spongioneuroblastoma
		9505/3	Ganglioglioma, anaplastic
OLFACTORY NEUROGENIC TUMOR	952	9520/3	Olfactory neurogenic tumor
		9521/3	Olfactory neurcytoma
		9522/3	Olfactory neuroblastoma
		9523/3	Olfactory neuroepithelioma
NEUROFIBROSARCOMA	954	9540/3	Malignant peripheral nerve sheath tumor
NEURILEMMOMA	956	9560/3	Neurilemmoma, malignant
		9561/3	MPNST with rhabdomyoblastic differentiation
PERINEURIOMA	957	9571/3	Perineurioma, malignant
MALIGNANT LYMPHOMA, NOS	959	9590/3	Malignant lymphoma, NOS
		9591/3	Malignant lymphoma, non-Hodgkin
		9596/3	Composite Hodgkin and non-Hodgkin lymphoma
HODGKIN LYMPHOMA	965	9650/3	Hodgkin lymphoma, NOS
		9651/3	Hodgkin lymphoma, lymphocyte-rich
		9652/3	Hodgkin lymphoma, mixed cellularity, NOS
		9653/3	Hodgkin lymphoma, lymphocytic deplet., NOS
		9654/3	Hodgkin lymph., lymphocyt. deplet., diffuse fibrosis
		9655/3	Hodgkin lymphoma, lymphocyt. deplet., reticular
		9659/3	Hodgkin lymph., nodular lymphocyte predom.

MIDDLE EAR C301

HODGKIN LYMPHOMA, NOD. SCLER.	966	9661/3	Hodgkin granuloma [obs]
		9662/3	Hodgkin sarcoma [obs]
		9663/3	Hodgkin lymphoma, nodular sclerosis, NOS
		9664/3	Hodgkin lymphoma, nod. scler., cellular phase
		9665/3	Hodgkin lymphoma, nod. scler., grade 1
		9667/3	Hodgkin lymphoma, nod. scler., grade 2
ML, SMALL B-CELL LYMPHOCYTIC	967	9670/3	ML, small B lymphocytic, NOS
		9671/3	ML, lymphoplasmacytic
		9673/3	Mantle cell lymphoma
		9675/3	ML, mixed sm. and lg. cell, diffuse
ML, LARGE B-CELL, DIFFUSE	968	9680/3	ML, large B-cell, diffuse
		9684/3	ML, large B-cell, diffuse, immunoblastic, NOS
		9687/3	Burkitt lymphoma, NOS
		9688/3	T-cell histiocyte rich large B-cell lymphoma
FOLLIC. & MARGINAL LYMPH, NOS	969	9690/3	Follicular lymphoma, NOS
		9691/3	Follicular lymphoma, grade 2
		9695/3	Follicular lymphoma, grade 1
		9698/3	Follicular lymphoma, grade 3
		9699/3	Marginal zone B-cell lymphoma, NOS
T-CELL LYMPHOMAS	970	9701/3	Sezary syndrome
		9702/3	Mature T-cell lymphoma, NOS
		9705/3	Angioimmunoblastic T-cell lymphoma
OTHER SPEC. NON-HODGKIN LYMPHOMA	971	9712/3	Intravascular large B-cell lymphoma
		9714/3	Anaplastic large cell lymphoma, T-cell and Null cell type
		9719/3	NK/T-cell lymphoma, nasal and nasal-type
PRECURS. CELL LYMPHOBLASTIC LYMPH.	972	9724/3	SystemicEBV pos. T-cell lymphoproliferative disease of childhood
		9727/3	Precursor cell lymphoblastic lymphoma, NOS
		9728/3	Precursor B-cell lymphoblastic lymphoma
		9729/3	Precursor T-cell lymphoblastic lymphoma
PLASMA CELL TUMORS	973	9731/3	Plasmacytoma, NOS
		9734/3	Plasmacytoma, extramedullary
		9735/3	Plasmablastic lymphoma
		9737/3	ALK positive large B-cell lymphoma
		9738/3	Lrg B-cell lymphoma in HHV8-assoc. multicentric Castleman DZ
MAST CELL TUMORS	974	9740/3	Mast cell sarcoma
		9741/3	Malignant mastocytosis

MIDDLE EAR C301

NEOPLASMS OF HISTIOCYTES AND ACCESSORY LYMPHOID CELLS	975	9750/3	Malignant histiocytosis
		9751/3	Langerhans cell histiocytosis, NOS
		9754/3	Langerhans cell histiocytosis, disseminated
		9755/3	Histiocytic sarcoma
		9756/3	Langerhans cell sarcoma
		9757/3	Interdigitating dendritic cell sarcoma
		9758/3	Follicular dendritic cell sarcoma
		9759/3	Fibroblastic reticular cell tumor
PRECURSOR LYMPHOID NEOPLASMS	981	9811/3	B lymphoblastic leukemia/lymphoma, NOS
		9812/3	Leukemia/lymphoma with t(9;22)(q34;q11.2);BCR-ABL1
		9813/3	Leukemia/lymphoma with t(v;11q23);MLL rearranged
		9814/3	Leukemia/lymphoma with t(12;21)(p13;q22);TEL-AML1(ETV6-RUNX1)
		9815/3	B lymphoblastic leukemia/lymphoma with hyperdiploidy
		9816/3	Leukemia/lymphoma with hypodiploidy (hypodiploid ALL)
		9817/3	B lymphblastic leukemia/lymphoma with t(5;14)(q31;q32);IL3-IGH
		9818/3	Leukemia/lymphoma with t(1;19)(q23;p13.3); E2A PBX1 (TCF3 PBX1)
LYMPHOID LEUKEMIA, NOS	982	9823/3	Chronic lymphocytic leukemia/small lymphocytic lymphoma
PROLYMPH/PRECURS LEUKEMIA	983	9831/3	T-cell large granular lymphocytic leukemia
		9837/3	T lymphoblastic leukemia/lymphoma
CHRONIC MYELOPROLIFERATIVE DIS.	996	9965/3	Myeloid and lymphoid neoplasms with PDGFRB rearrangement
		9967/3	Myeloid and lymphoid neoplasm with FGFR1 abnormalities
MYELOPLASTIC/MYELOPROLIFERATIVE NEOPLASMS	997	9971/3	Polymorphic PTLD
		9975/3	Myelodysplastic/Myeloproliferative neoplasm, unclassifiable

SINUSES C310-C313, C318

NEOPLASM	800	8000/3	Neoplasm, malignant
		8001/3	Tumor cells, malignant
		8002/3	Malignant tumor, small cell type
		8003/3	Malignant tumor, giant cell type
		8004/3	Malignant tumor, spindle cell type
		8005/3	Malignant tumor, clear cell type
CARCINOMA, NOS	801	8010/2	Carcinoma in situ, NOS
		8010/3	Carcinoma, NOS
		8011/3	Epithelioma, malignant
		8012/3	Large cell carcinoma, NOS
		8013/3	Large cell neuroendocrine carcinoma
		8014/3	Large cell carcinoma with rhabdoid phenotype
		8015/3	Glassy cell carcinoma
CARCINOMA, UNDIFF., NOS	802	8020/3	Carcinoma, undifferentiated type, NOS
		8021/3	Carcinoma, anaplastic type, NOS
		8022/3	Pleomorphic carcinoma
GIANT & SPINDLE CELL CARCINOMA	803	8030/3	Giant cell and spindle cell carcinoma
		8031/3	Giant cell carcinoma
		8032/3	Spindle cell carcinoma
		8033/3	Pseudosarcomatous carcinoma
		8034/3	Polygonal cell carcinoma
		8035/3	Carcinoma with osteoclast-like giant cells
PAPILLARY CARCINOMA, NOS	805	8050/2	Papillary carcinoma in situ
		8050/3	Papillary carcinoma, NOS
		8051/3	Verrucous carcinoma, NOS
		8052/2	Papillary squamous cell carcinoma, non-invasive
		8052/3	Papillary squamous cell carcinoma
SQUAMOUS CELL CARCINOMA, NOS	807	8070/2	Squamous cell carcinoma in situ, NOS
		8070/3	Squamous cell carcinoma, NOS
		8071/3	Sq. cell carcinoma, keratinizing, NOS
		8072/3	Sq. cell carcinoma, lg. cell, non-ker.
		8073/3	Sq. cell carcinoma, sm. cell, non-ker.
		8074/3	Sq. cell carcinoma, spindle cell
		8075/3	Squamous cell carcinoma, adenoid
		8076/2	Sq. cell carc. in situ with question. stromal invas.
		8076/3	Sq. cell carcinoma, micro-invasive
		8078/3	Squamous cell carcinoma with horn formation

SINUSES C310-C313, C318

LYMPHOEPITHELIAL CARCINOMA	808	8082/3	Lymphoepithelial carcinoma
		8083/3	Basaloid squamous cell carcinoma
		8084/3	Squamous cell carcinoma, clear cell type
		8085/3	Squamous cell carcinoma, HPV-positive
		8086/3	Squamous cell carcinoma, HPV-negative
TRANSITIONAL CELL CARCINOMA, NOS	812	8120/2	Transitional cell carcinoma in situ
		8120/3	Transitional cell carcinoma, NOS
		8121/3	Schneiderian carcinoma
		8122/3	Trans. cell carcinoma, spindle cell
		8123/3	Basaloid carcinoma
		8124/3	Cloacogenic carcinoma
PAPILLARY TRANS. CELL CARCINOMA	813	8130/2	Papillary trans. cell carcinoma, non-invasive
		8130/3	Papillary trans. cell carcinoma
		8131/3	Transitional cell carcinoma, micropapillary
ADENOCARCINOMA, NOS	814	8140/2	Adenocarcinoma in situ
		8140/3	Adenocarcinoma, NOS
		8141/3	Scirrhous adenocarcinoma
		8143/3	Superficial spreading adenocarcinoma
		8147/3	Basal cell adenocarcinoma
ADENOID CYSTIC & CRIBRIFORM CA.	820	8200/3	Adenoid cystic carcinoma
		8201/2	Cribriform carcinoma in situ
		8201/3	Cribriform carcinoma
BRONCHIOLO-ALVEOLAR ADENOCA.	825	8255/3	Adenocarcinoma with mixed subtypes
PAPILLARY ADENOCARCINOMA, NOS	826	8260/3	Papillary adenocarcinoma, NOS
		8261/2	Adenocarcinoma in situ in villous adenoma
		8261/3	Adenocarcinoma in villous adenoma
		8262/3	Villous adenocarcinoma
		8263/2	Adenocarcinoma in situ in tubulovillous adenoma
		8263/3	Adenocarcinoma in tubulovillous adenoma
MUCOEPIDERMOID CARCINOMA	843	8430/3	Mucoepidermoid carcinoma
MUCINOUS ADENOCARCINOMA	848	8480/3	Mucinous adenocarcinoma
		8481/3	Mucin-producing adenocarcinoma
ADENOSQUAMOUS CARCINOMA	856	8560/3	Adenosquamous carcinoma
		8562/3	Epithelial-myoepithelial carcinoma

SINUSES C310-C313, C318

ADENOCA. WITH METAPLASIA	857	8570/3	Adenocarcinoma with squamous metaplasia
		8571/3	Adenocarcinoma w cartilag. & oss. metaplas.
		8572/3	Adenocarcinoma with spindle cell mataplasia
		8573/3	Adenocarcinoma with apocrine metaplasia
		8574/3	Adenocarcinoma with neuroendocrine differen.
		8575/3	Metaplastic carcinoma, NOS
NEVI & MELANOMAS	872	8720/2	Melanoma in situ
		8720/3	Malignant melanoma, NOS
		8721/3	Nodular melanoma
		8722/3	Balloon cell melanoma
		8723/3	Malignant melanoma, regressing
AMELANOTIC MELANOMA	873	8730/3	Amelanotic melanoma
MAL. MEL. IN JUNCT. NEVUS	874	8743/3	Superficial spreading melanoma
		8745/3	Desmoplastic melanoma, malignant
		8746/3	Mucosal lentiginous melanoma
EPITHELIOID CELL MELANOMA	877	8770/3	Mixed epithel. & spindle cell melanoma
		8771/3	Epithelioid cell melanoma
		8772/3	Spindle cell melanoma, NOS
SARCOMA, NOS	880	8800/3	Sarcoma, NOS
		8801/3	Spindle cell sarcoma
		8802/3	Giant cell sarcoma
		8803/3	Small cell sarcoma
		8804/3	Epithelioid sarcoma
		8805/3	Undifferentiated sarcoma
		8806/3	Desmoplastic small round cell tumor
FIBROMATOUS NEOPLASMS	881	8810/3	Fibrosarcoma, NOS
		8811/3	Fibromyxosarcoma
		8813/3	Fascial fibrosarcoma
		8814/3	Infantile fibrosarcoma
		8815/3	Solitary fibrous tumor, malignant
SARCOMA, NOS	882	8825/3	Myofibroblastic sarcoma
FIBROUS HISTIOCYTOMA, MAL.	883	8830/3	Fibrous histiocytoma, malignant
MYOMATOUS NEOPLASMS	889	8890/3	Leiomyosarcoma, NOS
		8891/3	Epithelioid leiomyosarcoma
		8894/3	Angiomyosarcoma
		8895/3	Myosarcoma
		8896/3	Myxoid leiomyosarcoma

SINUSES C310-C313, C318

RHABDOMYOSARCOMA, NOS	890	8900/3	Rhabdomyosarcoma, NOS
		8901/3	Pleomorphic rhabdomyosarcoma, adult type
		8902/3	Mixed type rhabdomyosarcoma
EMBRYONAL RHABDOMYOSARCOMA	891	8910/3	Embryonal rhabdomyosarcoma
		8912/3	Spindle cell rhabdomyosarcoma
ALVEOLAR RHABDOMYOSARCOMA	892	8920/3	Alveolar rhabdomyosarcoma
		8921/3	Rhabdomyosarcoma with ganglionic differentiation
MIXED TUMOR, MALIGNANT, NOS	894	8940/3	Mixed tumor, malignant, NOS
		8941/3	Carcinoma in pleomorphic adenoma
SYNOVIAL SARCOMA, NOS	904	9045/3	Biphenotypic sinonasal sarcoma
CHORDOMA	937	9370/3	Chordoma, NOS
		9371/3	Chondroid chordoma
		9372/3	Dedifferentiated chordoma
NEUROBLASTOMA, NOS	950	9500/3	Neuroblastoma, NOS
		9501/3	Medulloepithelioma, NOS
		9502/3	Teratoid medulloepithelioma
		9503/3	Neuroepithelioma, NOS
		9504/3	Spongioneuroblastoma
		9505/3	Ganglioglioma, anaplastic
OLFACTORY NEUROGENIC TUMOR	952	9520/3	Olfactory neurogenic tumor
		9521/3	Olfactory neurcytoma
		9522/3	Olfactory neuroblastoma
		9523/3	Olfactory neuroepithelioma
NEUROFIBROSARCOMA	954	9540/3	Malignant peripheral nerve sheath tumor
NEURILEMMOMA	956	9560/3	Neurilemmoma, malignant
		9561/3	MPNST with rhabdomyoblastic differentiation
PERINEURIOMA	957	9571/3	Perineurioma, malignant
MALIGNANT LYMPHOMA, NOS	959	9590/3	Malignant lymphoma, NOS
		9591/3	Malignant lymphoma, non-Hodgkin
		9596/3	Composite Hodgkin and non-Hodgkin lymphoma
HODGKIN LYMPHOMA	965	9650/3	Hodgkin lymphoma, NOS
		9651/3	Hodgkin lymphoma, lymphocyte-rich
		9652/3	Hodgkin lymphoma, mixed cellularity, NOS
		9653/3	Hodgkin lymphoma, lymphocytic deplet., NOS
		9654/3	Hodgkin lymph., lymphocyt. deplet., diffuse fibrosis

SINUSES C310-C313, C318

HODGKIN LYMPHOMA	965	9655/3	Hodgkin lymphoma, lymphocyt. deplet., reticular
		9659/3	Hodgkin lymph., nodular lymphocyte predom.
HODGKIN LYMPHOMA, NOD. SCLER.	966	9661/3	Hodgkin granuloma [obs]
		9662/3	Hodgkin sarcoma [obs]
		9663/3	Hodgkin lymphoma, nodular sclerosis, NOS
		9664/3	Hodgkin lymphoma, nod. scler., cellular phase
		9665/3	Hodgkin lymphoma, nod. scler., grade 1
		9667/3	Hodgkin lymphoma, nod. scler., grade 2
ML, SMALL B-CELL LYMPHOCYTIC	967	9670/3	ML, small B lymphocytic, NOS
		9671/3	ML, lymphoplasmacytic
		9673/3	Mantle cell lymphoma
		9675/3	ML, mixed sm. and lg. cell, diffuse
ML, LARGE B-CELL, DIFFUSE	968	9680/3	ML, large B-cell, diffuse
		9684/3	ML, large B-cell, diffuse, immunoblastic, NOS
		9687/3	Burkitt lymphoma, NOS
		9688/3	T-cell histiocyte rich large B-cell lymphoma
FOLLIC. & MARGINAL LYMPH, NOS	969	9690/3	Follicular lymphoma, NOS
		9691/3	Follicular lymphoma, grade 2
		9695/3	Follicular lymphoma, grade 1
		9698/3	Follicular lymphoma, grade 3
		9699/3	Marginal zone B-cell lymphoma, NOS
T-CELL LYMPHOMAS	970	9701/3	Sezary syndrome
		9702/3	Mature T-cell lymphoma, NOS
		9705/3	Angioimmunoblastic T-cell lymphoma
OTHER SPEC. NON-HODGKIN LYMPHOMA	971	9712/3	Intravascular large B-cell lymphoma
		9714/3	Anaplastic large cell lymphoma, T-cell and Null cell type
		9719/3	NK/T-cell lymphoma, nasal and nasal-type
PRECURS. CELL LYMPHOBLASTIC LYMPH.	972	9724/3	SystemicEBV pos. T-cell lymphoproliferative disease of childhood
		9727/3	Precursor cell lymphoblastic lymphoma, NOS
		9728/3	Precursor B-cell lymphoblastic lymphoma
		9729/3	Precursor T-cell lymphoblastic lymphoma
PLASMA CELL TUMORS	973	9731/3	Plasmacytoma, NOS
		9734/3	Plasmacytoma, extramedullary
		9735/3	Plasmablastic lymphoma
		9737/3	ALK positive large B-cell lymphoma
		9738/3	Lrg B-cell lymphoma in HHV8-assoc. multicentric Castleman DZ

SINUSES C310-C313, C318

MAST CELL TUMORS	974	9740/3	Mast cell sarcoma
		9741/3	Malignant mastocytosis
NEOPLASMS OF HISTIOCYTES AND ACCESSORY LYMPHOID CELLS	975	9750/3	Malignant histiocytosis
		9751/3	Langerhans cell histiocytosis, NOS
		9754/3	Langerhans cell histiocytosis, disseminated
		9755/3	Histiocytic sarcoma
		9756/3	Langerhans cell sarcoma
		9757/3	Interdigitating dendritic cell sarcoma
		9758/3	Follicular dendritic cell sarcoma
		9759/3	Fibroblastic reticular cell tumor
PRECURSOR LYMPHOID NEOPLASMS	981	9811/3	B lymphoblastic leukemia/lymphoma, NOS
		9812/3	Leukemia/lymphoma with t(9;22)(q34;q11.2);BCR-ABL1
		9813/3	Leukemia/lymphoma with t(v;11q23);MLL rearranged
		9814/3	Leukemia/lymphoma with t(12;21)(p13;q22);TEL-AML1(ETV6-RUNX1)
		9815/3	B lymphoblastic leukemia/lymphoma with hyperdiploidy
		9816/3	Leukemia/lymphoma with hypodiploidy (hypodiploid ALL)
		9817/3	B lymphblastic leukemia/lymphoma with t(5;14)(q31;q32);IL3-IGH
		9818/3	Leukemia/lymphoma with t(1;19)(q23;p13.3); E2A PBX1 (TCF3 PBX1)
LYMPHOID LEUKEMIA, NOS	982	9823/3	Chronic lymphocytic leukemia/small lymphocytic lymphoma
PROLYMPH/PRECURS LEUKEMIA	983	9831/3	T-cell large granular lymphocytic leukemia
		9837/3	T lymphoblastic leukemia/lymphoma
CHRONIC MYELOPROLIFERATIVE DIS.	996	9965/3	Myeloid and lymphoid neoplasms with PDGFRB rearrangement
		9967/3	Myeloid and lymphoid neoplasm with FGFR1 abnormalities
MYELOPLASTIC/MYELOPROLIFERATIVE NEOPLASMS	997	9971/3	Polymorphic PTLD
		9975/3	Myelodysplastic/Myeloproliferative neoplasm, unclassifiable

ACCESSORY SINUS, NOS C319

NEOPLASM	800	8000/3	Neoplasm, malignant
		8001/3	Tumor cells, malignant
		8002/3	Malignant tumor, small cell type
		8003/3	Malignant tumor, giant cell type
		8004/3	Malignant tumor, spindle cell type
		8005/3	Malignant tumor, clear cell type
CARCINOMA, NOS	801	8010/2	Carcinoma in situ, NOS
		8010/3	Carcinoma, NOS
		8011/3	Epithelioma, malignant
		8012/3	Large cell carcinoma, NOS
		8013/3	Large cell neuroendocrine carcinoma
		8014/3	Large cell carcinoma with rhabdoid phenotype
		8015/3	Glassy cell carcinoma
CARCINOMA, UNDIFF., NOS	802	8020/3	Carcinoma, undifferentiated type, NOS
		8021/3	Carcinoma, anaplastic type, NOS
		8022/3	Pleomorphic carcinoma
		8023/3	NUT carcinoma
GIANT & SPINDLE CELL CARCINOMA	803	8030/3	Giant cell and spindle cell carcinoma
		8031/3	Giant cell carcinoma
		8032/3	Spindle cell carcinoma
		8033/3	Pseudosarcomatous carcinoma
		8034/3	Polygonal cell carcinoma
		8035/3	Carcinoma with osteoclast-like giant cells
PAPILLARY CARCINOMA, NOS	805	8050/2	Papillary carcinoma in situ
		8050/3	Papillary carcinoma, NOS
		8051/3	Verrucous carcinoma, NOS
		8052/2	Papillary squamous cell carcinoma, non-invasive
		8052/3	Papillary squamous cell carcinoma
SQUAMOUS CELL CARCINOMA, NOS	807	8070/2	Squamous cell carcinoma in situ, NOS
		8070/3	Squamous cell carcinoma, NOS
		8071/3	Sq. cell carcinoma, keratinizing, NOS
		8072/3	Sq. cell carcinoma, lg. cell, non-ker.
		8073/3	Sq. cell carcinoma, sm. cell, non-ker.
		8074/3	Sq. cell carcinoma, spindle cell
		8075/3	Squamous cell carcinoma, adenoid
		8076/2	Sq. cell carc. in situ with question. stromal invas.
		8076/3	Sq. cell carcinoma, micro-invasive
		8078/3	Squamous cell carcinoma with horn formation

ACCESSORY SINUS, NOS C319

LYMPHOEPITHELIAL CARCINOMA	808	8082/3	Lymphoepithelial carcinoma
		8083/3	Basaloid squamous cell carcinoma
		8084/3	Squamous cell carcinoma, clear cell type
		8085/3	Squamous cell carcinoma, HPV-positive
		8086/3	Squamous cell carcinoma, HPV-negative
TRANSITIONAL CELL CARCINOMA, NOS	812	8120/2	Transitional cell carcinoma in situ
		8120/3	Transitional cell carcinoma, NOS
		8121/3	Schneiderian carcinoma
		8122/3	Trans. cell carcinoma, spindle cell
		8123/3	Basaloid carcinoma
		8124/3	Cloacogenic carcinoma
PAPILLARY TRANS. CELL CARCINOMA	813	8130/2	Papillary trans. cell carcinoma, non-invasive
		8130/3	Papillary trans. cell carcinoma
		8131/3	Transitional cell carcinoma, micropapillary
ADENOCARCINOMA, NOS	814	8140/2	Adenocarcinoma in situ
		8140/3	Adenocarcinoma, NOS
		8141/3	Scirrhous adenocarcinoma
		8143/3	Superficial spreading adenocarcinoma
		8147/3	Basal cell adenocarcinoma
ADENOID CYSTIC & CRIBRIFORM CA.	820	8200/3	Adenoid cystic carcinoma
		8201/2	Cribriform carcinoma in situ
		8201/3	Cribriform carcinoma
BRONCHIOLO-ALVEOLAR ADENOCA.	825	8255/3	Adenocarcinoma with mixed subtypes
PAPILLARY ADENOCARCINOMA, NOS	826	8260/3	Papillary adenocarcinoma, NOS
		8261/2	Adenocarcinoma in situ in villous adenoma
		8261/3	Adenocarcinoma in villous adenoma
		8262/3	Villous adenocarcinoma
		8263/2	Adenocarcinoma in situ in tubulovillous adenoma
		8263/3	Adenocarcinoma in tubulovillous adenoma
MUCOEPIDERMOID CARCINOMA	843	8430/3	Mucoepidermoid carcinoma
MUCINOUS ADENOCARCINOMA	848	8480/3	Mucinous adenocarcinoma
		8481/3	Mucin-producing adenocarcinoma
ADENOSQUAMOUS CARCINOMA	856	8560/3	Adenosquamous carcinoma
		8562/3	Epithelial-myoepithelial carcinoma

ACCESSORYSINUS, NOS C319

ADENOCA. WITH METAPLASIA	857	8570/3	Adenocarcinoma with squamous metaplasia
		8571/3	Adenocarcinoma w cartilag. & oss. metaplas.
		8572/3	Adenocarcinoma with spindle cell mataplasia
		8573/3	Adenocarcinoma with apocrine metaplasia
		8574/3	Adenocarcinoma with neuroendocrine differen.
		8575/3	Metaplastic carcinoma, NOS
NEVI & MELANOMAS	872	8720/2	Melanoma in situ
		8720/3	Malignant melanoma, NOS
		8721/3	Nodular melanoma
		8722/3	Balloon cell melanoma
		8723/3	Malignant melanoma, regressing
AMELANOTIC MELANOMA	873	8730/3	Amelanotic melanoma
MAL. MEL. IN JUNCT. NEVUS	874	8743/3	Superficial spreading melanoma
		8745/3	Desmoplastic melanoma, malignant
		8746/3	Mucosal lentiginous melanoma
EPITHELIOID CELL MELANOMA	877	8770/3	Mixed epithel. & spindle cell melanoma
		8771/3	Epithelioid cell melanoma
		8772/3	Spindle cell melanoma, NOS
SARCOMA, NOS	880	8800/3	Sarcoma, NOS
		8801/3	Spindle cell sarcoma
		8802/3	Giant cell sarcoma
		8803/3	Small cell sarcoma
		8804/3	Epithelioid sarcoma
		8805/3	Undifferentiated sarcoma
		8806/3	Desmoplastic small round cell tumor
FIBROMATOUS NEOPLASMS	881	8810/3	Fibrosarcoma, NOS
		8811/3	Fibromyxosarcoma
		8813/3	Fascial fibrosarcoma
		8814/3	Infantile fibrosarcoma
		8815/3	Solitary fibrous tumor, malignant
SARCOMA, NOS	882	8825/3	Myofibroblastic sarcoma
FIBROUS HISTIOCYTOMA, MAL.	883	8830/3	Fibrous histiocytoma, malignant
MYOMATOUS NEOPLASMS	889	8890/3	Leiomyosarcoma, NOS
		8891/3	Epithelioid leiomyosarcoma
		8894/3	Angiomyosarcoma
		8895/3	Myosarcoma
		8896/3	Myxoid leiomyosarcoma

ACCESSORY SINUS, NOS C319

RHABDOMYOSARCOMA, NOS	890	8900/3	Rhabdomyosarcoma, NOS
		8901/3	Pleomorphic rhabdomyosarcoma, adult type
		8902/3	Mixed type rhabdomyosarcoma
EMBRYONAL RHABDOMYOSARCOMA	891	8910/3	Embryonal rhabdomyosarcoma
		8912/3	Spindle cell rhabdomyosarcoma
ALVEOLAR RHABDOMYOSARCOMA	892	8920/3	Alveolar rhabdomyosarcoma
		8921/3	Rhabdomyosarcoma with ganglionic differentiation
MIXED TUMOR, MALIGNANT, NOS	894	8940/3	Mixed tumor, malignant, NOS
		8941/3	Carcinoma in pleomorphic adenoma
SYNOVIAL SARCOMA, NOS	904	9045/3	Biphenotypic sinonasal sarcoma
CHORDOMA	937	9370/3	Chordoma, NOS
		9371/3	Chondroid chordoma
		9372/3	Dedifferentiated chordoma
NEUROBLASTOMA, NOS	950	9500/3	Neuroblastoma, NOS
		9501/3	Medulloepithelioma, NOS
		9502/3	Teratoid medulloepithelioma
		9503/3	Neuroepithelioma, NOS
		9504/3	Spongioneuroblastoma
		9505/3	Ganglioglioma, anaplastic
OLFACTORY NEUROGENIC TUMOR	952	9520/3	Olfactory neurogenic tumor
		9521/3	Olfactory neurcytoma
		9522/3	Olfactory neuroblastoma
		9523/3	Olfactory neuroepithelioma
NEUROFIBROSARCOMA	954	9540/3	Malignant peripheral nerve sheath tumor
NEURILEMMOMA	956	9560/3	Neurilemmoma, malignant
		9561/3	MPNST with rhabdomyoblastic differentiation
PERINEURIOMA	957	9571/3	Perineurioma, malignant
MALIGNANT LYMPHOMA, NOS	959	9590/3	Malignant lymphoma, NOS
		9591/3	Malignant lymphoma, non-Hodgkin
		9596/3	Composite Hodgkin and non-Hodgkin lymphoma
HODGKIN LYMPHOMA	965	9650/3	Hodgkin lymphoma, NOS
		9651/3	Hodgkin lymphoma, lymphocyte-rich
		9652/3	Hodgkin lymphoma, mixed cellularity, NOS
		9653/3	Hodgkin lymphoma, lymphocytic deplet., NOS
		9654/3	Hodgkin lymph., lymphocyt. deplet., diffuse fibrosis

ACCESSORYSINUS, NOS C319

HODGKIN LYMPHOMA	965	9655/3	Hodgkin lymphoma, lymphocyt. deplet., reticular
		9659/3	Hodgkin lymph., nodular lymphocyte predom.
HODGKIN LYMPHOMA, NOD. SCLER.	966	9661/3	Hodgkin granuloma [obs]
		9662/3	Hodgkin sarcoma [obs]
		9663/3	Hodgkin lymphoma, nodular sclerosis, NOS
		9664/3	Hodgkin lymphoma, nod. scler., cellular phase
		9665/3	Hodgkin lymphoma, nod. scler., grade 1
		9667/3	Hodgkin lymphoma, nod. scler., grade 2
ML, SMALL B-CELL LYMPHOCYTIC	967	9670/3	ML, small B lymphocytic, NOS
		9671/3	ML, lymphoplasmacytic
		9673/3	Mantle cell lymphoma
		9675/3	ML, mixed sm. and lg. cell, diffuse
ML, LARGE B-CELL, DIFFUSE	968	9680/3	ML, large B-cell, diffuse
		9684/3	ML, large B-cell, diffuse, immunoblastic, NOS
		9687/3	Burkitt lymphoma, NOS
		9688/3	T-cell histiocyte rich large B-cell lymphoma
FOLLIC. & MARGINAL LYMPH, NOS	969	9690/3	Follicular lymphoma, NOS
		9691/3	Follicular lymphoma, grade 2
		9695/3	Follicular lymphoma, grade 1
		9698/3	Follicular lymphoma, grade 3
		9699/3	Marginal zone B-cell lymphoma, NOS
T-CELL LYMPHOMAS	970	9701/3	Sezary syndrome
		9702/3	Mature T-cell lymphoma, NOS
		9705/3	Angioimmunoblastic T-cell lymphoma
OTHER SPEC. NON-HODGKIN LYMPHOMA	971	9712/3	Intravascular large B-cell lymphoma
		9714/3	Anaplastic large cell lymphoma, T-cell and Null cell type
		9719/3	NK/T-cell lymphoma, nasal and nasal-type
PRECURS. CELL LYMPHOBLASTIC LYMPH.	972	9724/3	SystemicEBV pos. T-cell lymphoproliferative disease of childhood
		9727/3	Precursor cell lymphoblastic lymphoma, NOS
		9728/3	Precursor B-cell lymphoblastic lymphoma
		9729/3	Precursor T-cell lymphoblastic lymphoma
PLASMA CELL TUMORS	973	9731/3	Plasmacytoma, NOS
		9734/3	Plasmacytoma, extramedullary
		9735/3	Plasmablastic lymphoma
		9737/3	ALK positive large B-cell lymphoma
		9738/3	Lrg B-cell lymphoma in HHV8-assoc. multicentric Castleman DZ

ACCESSORY SINUS, NOS C319

MAST CELL TUMORS	974	9740/3	Mast cell sarcoma
		9741/3	Malignant mastocytosis
NEOPLASMS OF HISTIOCYTES AND ACCESSORY LYMPHOID CELLS	975	9750/3	Malignant histiocytosis
		9751/3	Langerhans cell histiocytosis, NOS
		9754/3	Langerhans cell histiocytosis, disseminated
		9755/3	Histiocytic sarcoma
		9756/3	Langerhans cell sarcoma
		9757/3	Interdigitating dendritic cell sarcoma
		9758/3	Follicular dendritic cell sarcoma
		9759/3	Fibroblastic reticular cell tumor
PRECURSOR LYMPHOID NEOPLASMS	981	9811/3	B lymphoblastic leukemia/lymphoma, NOS
		9812/3	Leukemia/lymphoma with t(9;22)(q34;q11.2);BCR-ABL1
		9813/3	Leukemia/lymphoma with t(v;11q23);MLL rearranged
		9814/3	Leukemia/lymphoma with t(12;21)(p13;q22);TEL-AML1(ETV6-RUNX1)
		9815/3	B lymphoblastic leukemia/lymphoma with hyperdiploidy
		9816/3	Leukemia/lymphoma with hypodiploidy (hypodiploid ALL)
		9817/3	B lymphblastic leukemia/lymphoma with t(5;14)(q31;q32);IL3-IGH
		9818/3	Leukemia/lymphoma with t(1;19)(q23;p13.3); E2A PBX1 (TCF3 PBX1)
LYMPHOID LEUKEMIA, NOS	982	9823/3	Chronic lymphocytic leukemia/small lymphocytic lymphoma
PROLYMPH/PRECURS LEUKEMIA	983	9831/3	T-cell large granular lymphocytic leukemia
		9837/3	T lymphoblastic leukemia/lymphoma
CHRONIC MYELOPROLIFERATIVE DIS.	996	9965/3	Myeloid and lymphoid neoplasms with PDGFRB rearrangement
		9967/3	Myeloid and lymphoid neoplasm with FGFR1 abnormalities
MYELOPLASTIC/MYELOPROLIFERATIVE NEOPLASMS	997	9971/3	Polymorphic PTLD
		9975/3	Myelodysplastic/Myeloproliferative neoplasm, unclassifiable

LARYNX C320-C323,C328-C329

NEOPLASM	800	8000/3	Neoplasm, malignant
		8001/3	Tumor cells, malignant
		8002/3	Malignant tumor, small cell type
		8003/3	Malignant tumor, giant cell type
		8004/3	Malignant tumor, spindle cell type
		8005/3	Malignant tumor, clear cell type
CARCINOMA, NOS	801	8010/2	Carcinoma in situ, NOS
		8010/3	Carcinoma, NOS
		8011/3	Epithelioma, malignant
		8012/3	Large cell carcinoma, NOS
		8013/3	Large cell neuroendocrine carcinoma
		8014/3	Large cell carcinoma with rhabdoid phenotype
		8015/3	Glassy cell carcinoma
CARCINOMA, UNDIFF., NOS	802	8020/3	Carcinoma, undifferentiated type, NOS
		8021/3	Carcinoma, anaplastic type, NOS
		8022/3	Pleomorphic carcinoma
GIANT & SPINDLE CELL CARCINOMA	803	8030/3	Giant cell and spindle cell carcinoma
		8031/3	Giant cell carcinoma
		8032/3	Spindle cell carcinoma
		8033/3	Pseudosarcomatous carcinoma
		8034/3	Polygonal cell carcinoma
		8035/3	Carcinoma with osteoclast-like giant cells
SMALL CELL CARCINOMA, NOS	804	8041/3	Small cell carcinoma, NOS
		8043/3	Small cell carcinoma, fusiform cell
PAPILLARY CARCINOMA, NOS	805	8050/2	Papillary carcinoma in situ
		8050/3	Papillary carcinoma, NOS
		8051/3	Verrucous carcinoma, NOS
		8052/2	Papillary squamous cell carcinoma, non-invasive
		8052/3	Papillary squamous cell carcinoma
SQUAMOUS CELL CARCINOMA, NOS	807	8070/2	Squamous cell carcinoma in situ, NOS
		8070/3	Squamous cell carcinoma, NOS
		8071/3	Sq. cell carcinoma, keratinizing, NOS
		8072/3	Sq. cell carcinoma, lg. cell, non-ker.
		8073/3	Sq. cell carcinoma, sm. cell, non-ker.
		8074/3	Sq. cell carcinoma, spindle cell
		8075/3	Squamous cell carcinoma, adenoid
		8076/2	Sq. cell carc. in situ with question. stromal invas.
		8076/3	Sq. cell carcinoma, micro-invasive
		8078/3	Squamous cell carcinoma with horn formation

LARYNX C320-C323,C328-C329

LYMPHOEPITHELIAL CARCINOMA	808	8082/3	Lymphoepithelial carcinoma
		8083/3	Basaloid squamous cell carcinoma
		8084/3	Squamous cell carcinoma, clear cell type
TRANSITIONAL CELL CARCINOMA, NOS	812	8120/2	Transitional cell carcinoma in situ
		8120/3	Transitional cell carcinoma, NOS
		8121/3	Schneiderian carcinoma
		8122/3	Trans. cell carcinoma, spindle cell
		8123/3	Basaloid carcinoma
		8124/3	Cloacogenic carcinoma
ADENOCARCINOMA, NOS	814	8140/2	Adenocarcinoma in situ
		8140/3	Adenocarcinoma, NOS
		8141/3	Scirrhous adenocarcinoma
		8143/3	Superficial spreading adenocarcinoma
		8147/3	Basal cell adenocarcinoma
ADENOID CYSTIC & CRIBRIFORM CA.	820	8200/3	Adenoid cystic carcinoma
		8201/2	Cribriform carcinoma in situ
		8201/3	Cribriform carcinoma
BRONCHIOLO-ALVEOLAR ADENOCA.	825	8255/3	Adenocarcinoma with mixed subtypes
PAPILLARY ADENOCARCINOMA, NOS	826	8260/3	Papillary adenocarcinoma, NOS
		8261/2	Adenocarcinoma in situ in villous adenoma
		8261/3	Adenocarcinoma in villous adenoma
		8262/3	Villous adenocarcinoma
		8263/2	Adenocarcinoma in situ in tubulovillous adenoma
		8263/3	Adenocarcinoma in tubulovillous adenoma
MUCOEPIDERMOID CARCINOMA	843	8430/3	Mucoepidermoid carcinoma
MUCINOUS ADENOCARCINOMA	848	8480/3	Mucinous adenocarcinoma
		8481/3	Mucin-producing adenocarcinoma
ADENOSQUAMOUS CARCINOMA	856	8560/3	Adenosquamous carcinoma
		8562/3	Epithelial-myoepithelial carcinoma
SARCOMA, NOS	880	8800/3	Sarcoma, NOS
		8801/3	Spindle cell sarcoma
		8802/3	Giant cell sarcoma
		8803/3	Small cell sarcoma
		8804/3	Epithelioid sarcoma
		8805/3	Undifferentiated sarcoma
		8806/3	Desmoplastic small round cell tumor

LARYNX C320-C323,C328-C329

FIBROMATOUS NEOPLASMS	881	8810/3	Fibrosarcoma, NOS
		8811/3	Fibromyxosarcoma
		8813/3	Fascial fibrosarcoma
		8814/3	Infantile fibrosarcoma
		8815/3	Solitary fibrous tumor, malignant
SARCOMA, NOS	882	8825/3	Myofibroblastic sarcoma
CARCINOSARCOMA, NOS	898	8980/3	Carcinosarcoma, NOS
		8981/3	Carcinosarcoma, embryonal type
		8982/3	Malignant myoepithelioma
CHONDROSARCOMA, NOS	922	9220/3	Chondrosarcoma, NOS
		9221/3	Juxtacortical chondrosarcoma
MALIGNANT LYMPHOMA, NOS	959	9590/3	Malignant lymphoma, NOS
		9591/3	Malignant lymphoma, non-Hodgkin
		9596/3	Composite Hodgkin and non-Hodgkin lymphoma
HODGKIN LYMPHOMA	965	9650/3	Hodgkin lymphoma, NOS
		9651/3	Hodgkin lymphoma, lymphocyte-rich
		9652/3	Hodgkin lymphoma, mixed cellularity, NOS
		9653/3	Hodgkin lymphoma, lymphocytic deplet., NOS
		9654/3	Hodgkin lymph., lymphocyt. deplet., diffuse fibrosis
		9655/3	Hodgkin lymphoma, lymphocyt. deplet., reticular
		9659/3	Hodgkin lymph., nodular lymphocyte predom.
HODGKIN LYMPHOMA, NOD. SCLER.	966	9661/3	Hodgkin granuloma [obs]
		9662/3	Hodgkin sarcoma [obs]
		9663/3	Hodgkin lymphoma, nodular sclerosis, NOS
		9664/3	Hodgkin lymphoma, nod. scler., cellular phase
		9665/3	Hodgkin lymphoma, nod. scler., grade 1
		9667/3	Hodgkin lymphoma, nod. scler., grade 2
ML, SMALL B-CELL LYMPHOCYTIC	967	9670/3	ML, small B lymphocytic, NOS
		9671/3	ML, lymphoplasmacytic
		9673/3	Mantle cell lymphoma
		9675/3	ML, mixed sm. and lg. cell, diffuse
ML, LARGE B-CELL, DIFFUSE	968	9680/3	ML, large B-cell, diffuse
		9684/3	ML, large B-cell, diffuse, immunoblastic, NOS
		9687/3	Burkitt lymphoma, NOS
		9688/3	T-cell histiocyte rich large B-cell lymphoma

LARYNX C320-C323,C328-C329

FOLLIC. & MARGINAL LYMPH, NOS	969	9690/3	Follicular lymphoma, NOS
		9691/3	Follicular lymphoma, grade 2
		9695/3	Follicular lymphoma, grade 1
		9698/3	Follicular lymphoma, grade 3
		9699/3	Marginal zone B-cell lymphoma, NOS
T-CELL LYMPHOMAS	970	9701/3	Sezary syndrome
		9702/3	Mature T-cell lymphoma, NOS
		9705/3	Angioimmunoblastic T-cell lymphoma
OTHER SPEC. NON-HODGKIN LYMPHOMA	971	9712/3	Intravascular large B-cell lymphoma
		9714/3	Anaplastic large cell lymphoma, T-cell and Null cell type
		9719/3	NK/T-cell lymphoma, nasal and nasal-type
PRECURS. CELL LYMPHOBLASTIC LYMPH.	972	9724/3	SystemicEBV pos. T-cell lymphoproliferative disease of childhood
		9727/3	Precursor cell lymphoblastic lymphoma, NOS
		9728/3	Precursor B-cell lymphoblastic lymphoma
		9729/3	Precursor T-cell lymphoblastic lymphoma
PLASMA CELL TUMORS	973	9731/3	Plasmacytoma, NOS
		9734/3	Plasmacytoma, extramedullary
		9735/3	Plasmablastic lymphoma
		9737/3	ALK positive large B-cell lymphoma
		9738/3	Lrg B-cell lymphoma in HHV8-assoc. multicentric Castleman DZ
MAST CELL TUMORS	974	9740/3	Mast cell sarcoma
		9741/3	Malignant mastocytosis
NEOPLASMS OF HISTIOCYTES AND ACCESSORY LYMPHOID CELLS	975	9750/3	Malignant histiocytosis
		9751/3	Langerhans cell histiocytosis, NOS
		9754/3	Langerhans cell histiocytosis, disseminated
		9755/3	Histiocytic sarcoma
		9756/3	Langerhans cell sarcoma
		9757/3	Interdigitating dendritic cell sarcoma
		9758/3	Follicular dendritic cell sarcoma
		9759/3	Fibroblastic reticular cell tumor
PRECURSOR LYMPHOID NEOPLASMS	981	9811/3	B lymphoblastic leukemia/lymphoma, NOS
		9812/3	Leukemia/lymphoma with t(9;22)(q34;q11.2);BCR-ABL1
		9813/3	Leukemia/lymphoma with t(v;11q23);MLL rearranged
		9814/3	Leukemia/lymphoma with t(12;21)(p13;q22);TEL-AML1(ETV6-RUNX1)
		9815/3	B lymphoblastic leukemia/lymphoma with hyperdiploidy
		9816/3	Leukemia/lymphoma with hypodiploidy (hypodiploid ALL)
		9817/3	B lymphblastic leukemia/lymphoma with t(5;14)(q31;q32);IL3-IGH
		9818/3	Leukemia/lymphoma with t(1;19)(q23;p13.3); E2A PBX1 (TCF3 PBX1)
LYMPHOID LEUKEMIA, NOS	982	9823/3	Chronic lymphocytic leukemia/small lymphocytic lymphoma

LARYNX C320-C323,C328-C329

PROLYMPH/PRECURS LEUKEMIA	983	9831/3	T-cell large granular lymphocytic leukemia
		9837/3	T lymphoblastic leukemia/lymphoma
CHRONIC MYELOPROLIFERATIVE DIS.	996	9965/3	Myeloid and lymphoid neoplasms with PDGFRB rearrangement
		9967/3	Myeloid and lymphoid neoplasm with FGFR1 abnormalities
MYELOPLASTIC/MYELOPROLIFERATIVE NEOPLASMS	997	9971/3	Polymorphic PTLD
		9975/3	Myelodysplastic/Myeloproliferative neoplasm, unclassifiable

TRACHEA C339

NEOPLASM	800	8000/3	Neoplasm, malignant
		8001/3	Tumor cells, malignant
		8002/3	Malignant tumor, small cell type
		8003/3	Malignant tumor, giant cell type
		8004/3	Malignant tumor, spindle cell type
		8005/3	Malignant tumor, clear cell type
CARCINOMA, NOS	801	8010/2	Carcinoma in situ, NOS
		8010/3	Carcinoma, NOS
		8011/3	Epithelioma, malignant
		8012/3	Large cell carcinoma, NOS
		8013/3	Large cell neuroendocrine carcinoma
		8014/3	Large cell carcinoma with rhabdoid phenotype
		8015/3	Glassy cell carcinoma
CARCINOMA, UNDIFF., NOS	802	8020/3	Carcinoma, undifferentiated type, NOS
		8021/3	Carcinoma, anaplastic type, NOS
		8022/3	Pleomorphic carcinoma
GIANT & SPINDLE CELL CARCINOMA	803	8030/3	Giant cell and spindle cell carcinoma
		8031/3	Giant cell carcinoma
		8032/3	Spindle cell carcinoma
		8033/3	Pseudosarcomatous carcinoma
		8034/3	Polygonal cell carcinoma
		8035/3	Carcinoma with osteoclast-like giant cells
NON-SMALL CELL CARCINOMA, NOS	804	8046/3	Non-small cell carcinoma
SMALL CELL CARCINOMA, NOS	804	8041/3	Small cell carcinoma, NOS
		8042/3	Oat cell carcinoma
		8043/3	Small cell carcinoma, fusiform cell
		8044/3	Small cell carcinoma, intermediate cell
		8045/3	Combined small cell carcinoma
PAPILLARY CARCINOMA, NOS	805	8050/2	Papillary carcinoma in situ
		8050/3	Papillary carcinoma, NOS
		8051/3	Verrucous carcinoma, NOS
		8052/2	Papillary squamous cell carcinoma, non-invasive
		8052/3	Papillary squamous cell carcinoma
SQUAMOUS CELL CARCINOMA, NOS	807	8070/2	Squamous cell carcinoma in situ, NOS
		8070/3	Squamous cell carcinoma, NOS
		8071/3	Sq. cell carcinoma, keratinizing, NOS
		8072/3	Sq. cell carcinoma, lg. cell, non-ker.
		8073/3	Sq. cell carcinoma, sm. cell, non-ker.
		8074/3	Sq. cell carcinoma, spindle cell
		8075/3	Squamous cell carcinoma, adenoid

TRACHEA C339

SQUAMOUS CELL CARCINOMA, NOS	807	8076/2	Sq. cell carc. in situ with question. stromal invas.
		8076/3	Sq. cell carcinoma, micro-invasive
		8078/3	Squamous cell carcinoma with horn formation
ADENOCARCINOMA, NOS	814	8140/2	Adenocarcinoma in situ
		8140/3	Adenocarcinoma, NOS
		8141/3	Scirrhous adenocarcinoma
		8143/3	Superficial spreading adenocarcinoma
		8147/3	Basal cell adenocarcinoma
ADENOID CYSTIC & CRIBRIFORM CA.	820	8200/3	Adenoid cystic carcinoma
		8201/2	Cribriform carcinoma in situ
		8201/3	Cribriform carcinoma
BRONCHIOLO-ALVEOLAR ADENOCA.	825	8250/3	Lepidic adenocarcinoma
		8252/3	Bronchiolo-alveolar carcinoma, non-mucinous
		8253/3	Invasive mucinous adenocarcinoma
		8254/3	Mixed invasive mucinous and non-mucinous adenocarcinoma
		8255/3	Adenocarcinoma with mixed subtypes
PAPILLARY ADENOCARCINOMA, NOS	826	8260/3	Papillary adenocarcinoma, NOS
		8261/2	Adenocarcinoma in situ in villous adenoma
		8261/3	Adenocarcinoma in villous adenoma
		8262/3	Villous adenocarcinoma
		8263/2	Adenocarcinoma in situ in tubulovillous adenoma
		8263/3	Adenocarcinoma in tubulovillous adenoma
SARCOMA, NOS	880	8800/3	Sarcoma, NOS
		8801/3	Spindle cell sarcoma
		8802/3	Giant cell sarcoma
		8803/3	Small cell sarcoma
		8804/3	Epithelioid sarcoma
		8805/3	Undifferentiated sarcoma
		8806/3	Desmoplastic small round cell tumor
FIBROMATOUS NEOPLASMS	881	8810/3	Fibrosarcoma, NOS
		8811/3	Fibromyxosarcoma
		8813/3	Fascial fibrosarcoma
		8814/3	Infantile fibrosarcoma
		8815/3	Solitary fibrous tumor, malignant
SARCOMA, NOS	882	8825/3	Myofibroblastic sarcoma
FIBROUS HISTIOCYTOMA, MAL.	883	8830/3	Fibrous histiocytoma, malignant

TRACHEA C339

HEPATOBLASTOMA	897	8972/3	Pulmonary blastoma
		8973/3	Pleuropulmonary blastoma
CHONDROSARCOMA, NOS	922	9220/3	Chondrosarcoma, NOS
		9221/3	Juxtacortical chondrosarcoma
FOLLIC. & MARGINAL LYMPH, NOS	969	9699/3	Marginal zone B-cell lymphoma, NOS
LYMPHOID LEUKEMIA, NOS	982	9823/3	Chronic lymphocytic leukemia/small lymphocytic lymphoma

LUNG & BRONCHUS C340-C343,C348-C349

NEOPLASM	800	8000/3	Neoplasm, malignant
		8001/3	Tumor cells, malignant
		8002/3	Malignant tumor, small cell type
		8003/3	Malignant tumor, giant cell type
		8004/3	Malignant tumor, spindle cell type
		8005/3	Malignant tumor, clear cell type
CARCINOMA, NOS	801	8010/2	Carcinoma in situ, NOS
		8010/3	Carcinoma, NOS
		8011/3	Epithelioma, malignant
		8012/3	Large cell carcinoma, NOS
		8013/3	Large cell neuroendocrine carcinoma
		8014/3	Large cell carcinoma with rhabdoid phenotype
		8015/3	Glassy cell carcinoma
CARCINOMA, UNDIFF., NOS	802	8020/3	Carcinoma, undifferentiated type, NOS
		8021/3	Carcinoma, anaplastic type, NOS
		8022/3	Pleomorphic carcinoma
		8023/3	NUT carcinoma
GIANT & SPINDLE CELL CARCINOMA	803	8030/3	Giant cell and spindle cell carcinoma
		8031/3	Giant cell carcinoma
		8032/3	Spindle cell carcinoma
		8033/3	Pseudosarcomatous carcinoma
		8034/3	Polygonal cell carcinoma
		8035/3	Carcinoma with osteoclast-like giant cells
NON-SMALL CELL CARCINOMA, NOS	804	8046/3	Non-small cell carcinoma
SMALL CELL CARCINOMA, NOS	804	8041/3	Small cell carcinoma, NOS
		8042/3	Oat cell carcinoma
		8043/3	Small cell carcinoma, fusiform cell
		8044/3	Small cell carcinoma, intermediate cell
		8045/3	Combined small cell carcinoma
PAPILLARY CARCINOMA, NOS	805	8050/2	Papillary carcinoma in situ
		8050/3	Papillary carcinoma, NOS
		8051/3	Verrucous carcinoma, NOS
		8052/2	Papillary squamous cell carcinoma, non-invasive
		8052/3	Papillary squamous cell carcinoma
SQUAMOUS CELL CARCINOMA, NOS	807	8070/2	Squamous cell carcinoma in situ, NOS
		8070/3	Squamous cell carcinoma, NOS
		8071/3	Sq. cell carcinoma, keratinizing, NOS
		8072/3	Sq. cell carcinoma, lg. cell, non-ker.
		8073/3	Sq. cell carcinoma, sm. cell, non-ker.
		8074/3	Sq. cell carcinoma, spindle cell

LUNG & BRONCHUS C340-C343,C348-C349

SQUAMOUS CELL CARCINOMA, NOS	807	8075/3	Squamous cell carcinoma, adenoid
		8076/2	Sq. cell carc. in situ with question. stromal invas.
		8076/3	Sq. cell carcinoma, micro-invasive
		8078/3	Squamous cell carcinoma with horn formation
LYMPHOEPITHELIAL CARCINOMA	808	8083/3	Basaloid squamous cell carcinoma
TRANSITIONAL CELL CARCINOMA, NOS	812	8120/2	Transitional cell carcinoma in situ
		8120/3	Transitional cell carcinoma, NOS
		8121/3	Schneiderian carcinoma
		8122/3	Trans. cell carcinoma, spindle cell
		8123/3	Basaloid carcinoma
		8124/3	Cloacogenic carcinoma
ADENOCARCINOMA, NOS	814	8140/2	Adenocarcinoma in situ
		8140/3	Adenocarcinoma, NOS
		8141/3	Scirrhous adenocarcinoma
		8143/3	Superficial spreading adenocarcinoma
		8147/3	Basal cell adenocarcinoma
ADENOID CYSTIC & CRIBRIFORM CA.	820	8200/3	Adenoid cystic carcinoma
		8201/2	Cribriform carcinoma in situ
		8201/3	Cribriform carcinoma
SOLID CARCINOMA, NOS	823	8230/2	Duct carcinoma in situ, solid type
		8230/3	Solid carcinoma, NOS
		8231/3	Carcinoma simplex
CARCINOID TUMOR, MALIGNANT	824	8240/3	Carcinoid tumor, malignant
		8241/3	Enterochromaffin cell carcinoid
		8242/3	Enterochromaffin-like cell tumor, malignant
		8243/3	Goblet cell carcinoid
		8244/3	Composite carcinoid
		8245/3	Adenocarcinoid tumor
		8246/3	Neuroendocrine carcinoma
		8249/3	Atypical carcinoid tumor
BRONCHIOLO-ALVEOLAR ADENOCA.	825	8250/2	Adenocarcinoma in situ, non-mucinous
		8250/3	Lepidic adenocarcinoma
		8251/3	Alveolar adenocarcinoma
		8252/3	Bronchiolo-alveolar carcinoma, non-mucinous
		8253/2	Adenocarcinoma in situ, mucinous
		8253/3	Invasive mucinous adenocarcinoma
		8254/3	Mixed invasive mucinous and non-mucinous adenocarcinoma
		8255/3	Adenocarcinoma with mixed subtypes
		8256/3	Minimally invasive adenocarcinoma, non-mucinous
		8257/3	Minimally invasive adenocarcinoma, mucinous

LUNG & BRONCHUS C340-C343,C348-C349

PAPILLARY ADENOCARCINOMA, NOS	826	8260/3	Papillary adenocarcinoma, NOS
CLEAR CELL ADENOCARCINOMA, NOS	831	8310/3	Clear cell adenocarcinoma, NOS
GRANULAR CELL CARCINOMA	832	8320/3	Granular cell carcinoma
		8323/3	Mixed cell adenocarcinoma
MUCOEPIDERMOID CARCINOMA	843	8430/3	Mucoepidermoid carcinoma
MUCINOUS ADENOCARCINOMA	848	8480/3	Mucinous adenocarcinoma
		8481/3	Mucin-producing adenocarcinoma
SIGNET RING CELL CARCINOMA	849	8490/3	Signet ring cell carcinoma
MEDULLARY CARCINOMA, NOS	851	8510/3	Medullary carcinoma, NOS
ACINAR CELL CARCINOMA	855	8550/3	Acinar cell carcinoma
		8551/3	Acinar cell cystadenocarcinoma
ADENOSQUAMOUS CARCINOMA	856	8560/3	Adenosquamous carcinoma
		8562/3	Epithelial-myoepithelial carcinoma
ADENOCA. WITH METAPLASIA	857	8570/3	Adenocarcinoma with squamous metaplasia
		8571/3	Adenocarcinoma w cartilag. & oss. metaplas.
		8572/3	Adenocarcinoma with spindle cell mataplasia
		8573/3	Adenocarcinoma with apocrine metaplasia
		8574/3	Adenocarcinoma with neuroendocrine differen.
		8575/3	Metaplastic carcinoma, NOS
		8576/3	Hepatoid adenocarcinoma
GLOMANGIOSARCOMA	871	8714/3	PEComa, malignant
SARCOMA, NOS	880	8800/3	Sarcoma, NOS
		8801/3	Spindle cell sarcoma
		8802/3	Giant cell sarcoma
		8803/3	Small cell sarcoma
		8804/3	Epithelioid sarcoma
		8805/3	Undifferentiated sarcoma
		8806/3	Desmoplastic small round cell tumor
FIBROMATOUS NEOPLASMS	881	8810/3	Fibrosarcoma, NOS
		8811/3	Fibromyxosarcoma
		8813/3	Fascial fibrosarcoma
		8814/3	Infantile fibrosarcoma
		8815/3	Solitary fibrous tumor, malignant

LUNG & BRONCHUS C340-C343,C348-C349

SARCOMA, NOS	882	8825/3	Myofibroblastic sarcoma
FIBROUS HISTIOCYTOMA, MAL.	883	8830/3	Fibrous histiocytoma, malignant
MYXOSARCOMA	884	8842/3	Pulmonary myxoid sarcoma with EWSR1-CREB1 translocation
MYOMATOUS NEOPLASMS	889	8890/3	Leiomyosarcoma, NOS
		8891/3	Epithelioid leiomyosarcoma
		8894/3	Angiomyosarcoma
		8895/3	Myosarcoma
		8896/3	Myxoid leiomyosarcoma
RHABDOMYOSARCOMA, NOS	890	8900/3	Rhabdomyosarcoma, NOS
		8901/3	Pleomorphic rhabdomyosarcoma, adult type
		8902/3	Mixed type rhabdomyosarcoma
EMBRYONAL RHABDOMYOSARCOMA	891	8910/3	Embryonal rhabdomyosarcoma
		8912/3	Spindle cell rhabdomyosarcoma
HEPATOBLASTOMA	897	8972/3	Pulmonary blastoma
		8973/3	Pleuropulmonary blastoma
CARCINOSARCOMA, NOS	898	8980/3	Carcinosarcoma, NOS
		8981/3	Carcinosarcoma, embryonal type
		8982/3	Malignant myoepithelioma
MESENCHYMOMA, MALIGNANT	899	8990/3	Mesenchymoma, malignant
		8991/3	Embryonal sarcoma
MESOTHELIOMA, MALIGNANT	905	9050/3	Mesothelioma, malignant
		9051/3	Fibrous mesothelioma, malignant
		9052/3	Epithel. mesothelioma, mal.
		9053/3	Mesothelioma, biphasic, malignant
BLOOD VESSEL TUMORS	912	9120/3	Hemangiosarcoma
HEMANGIOENDOTHELIOMA	913	9133/3	Epithelioid hemangioendothelioma, malignant
		9137/3	Intimal sarcoma
KAPOSI SARCOMA	914	9140/3	Kaposi sarcoma
MALIGNANT LYMPHOMA, NOS	959	9590/3	Malignant lymphoma, NOS
		9591/3	Malignant lymphoma, non-Hodgkin
		9596/3	Composite Hodgkin and non-Hodgkin lymphoma

LUNG & BRONCHUS C340-C343,C348-C349

HODGKIN LYMPHOMA	965	9650/3	Hodgkin lymphoma, NOS
		9651/3	Hodgkin lymphoma, lymphocyte-rich
		9652/3	Hodgkin lymphoma, mixed cellularity, NOS
		9653/3	Hodgkin lymphoma, lymphocytic deplet., NOS
		9654/3	Hodgkin lymph., lymphocyt. deplet., diffuse fibrosis
		9655/3	Hodgkin lymphoma, lymphocyt. deplet., reticular
		9659/3	Hodgkin lymph., nodular lymphocyte predom.
HODGKIN LYMPHOMA, NOD. SCLER.	966	9661/3	Hodgkin granuloma [obs]
		9662/3	Hodgkin sarcoma [obs]
		9663/3	Hodgkin lymphoma, nodular sclerosis, NOS
		9664/3	Hodgkin lymphoma, nod. scler., cellular phase
		9665/3	Hodgkin lymphoma, nod. scler., grade 1
		9667/3	Hodgkin lymphoma, nod. scler., grade 2
ML, SMALL B-CELL LYMPHOCYTIC	967	9670/3	ML, small B lymphocytic, NOS
		9671/3	ML, lymphoplasmacytic
		9673/3	Mantle cell lymphoma
		9675/3	ML, mixed sm. and lg. cell, diffuse
		9678/3	Primary effusion lymphoma
		9679/3	Mediastinal large B-cell lymphoma
ML, LARGE B-CELL, DIFFUSE	968	9680/3	ML, large B-cell, diffuse
		9684/3	ML, large B-cell, diffuse, immunoblastic, NOS
		9687/3	Burkitt lymphoma, NOS
		9688/3	T-cell histiocyte rich large B-cell lymphoma
FOLLIC. & MARGINAL LYMPH, NOS	969	9690/3	Follicular lymphoma, NOS
		9691/3	Follicular lymphoma, grade 2
		9695/3	Follicular lymphoma, grade 1
		9698/3	Follicular lymphoma, grade 3
		9699/3	Marginal zone B-cell lymphoma, NOS
T-CELL LYMPHOMAS	970	9701/3	Sezary syndrome
		9702/3	Mature T-cell lymphoma, NOS
		9705/3	Angioimmunoblastic T-cell lymphoma
OTHER SPEC. NON-HODGKIN LYMPHOMA	971	9712/3	Intravascular large B-cell lymphoma
		9714/3	Anaplastic large cell lymphoma, T-cell and Null cell type
		9719/3	NK/T-cell lymphoma, nasal and nasal-type
PRECURS. CELL LYMPHOBLASTIC LYMPH.	972	9724/3	SystemicEBV pos. T-cell lymphoproliferative disease of childhood
		9727/3	Precursor cell lymphoblastic lymphoma, NOS
		9728/3	Precursor B-cell lymphoblastic lymphoma
		9729/3	Precursor T-cell lymphoblastic lymphoma

LUNG & BRONCHUS C340-C343,C348-C349

PLASMA CELL TUMORS	973	9731/3	Plasmacytoma, NOS
		9734/3	Plasmacytoma, extramedullary
		9735/3	Plasmablastic lymphoma
		9737/3	ALK positive large B-cell lymphoma
		9738/3	Lrg B-cell lymphoma in HHV8-assoc. multicentric Castleman DZ
MAST CELL TUMORS	974	9740/3	Mast cell sarcoma
		9741/3	Malignant mastocytosis
NEOPLASMS OF HISTIOCYTES AND ACCESSORY LYMPHOID CELLS	975	9750/3	Malignant histiocytosis
		9751/3	Langerhans cell histiocytosis, NOS
		9754/3	Langerhans cell histiocytosis, disseminated
		9755/3	Histiocytic sarcoma
		9756/3	Langerhans cell sarcoma
		9757/3	Interdigitating dendritic cell sarcoma
		9758/3	Follicular dendritic cell sarcoma
		9759/3	Fibroblastic reticular cell tumor
PRECURSOR LYMPHOID NEOPLASMS	981	9811/3	B lymphoblastic leukemia/lymphoma, NOS
		9812/3	Leukemia/lymphoma with t(9;22)(q34;q11.2);BCR-ABL1
		9813/3	Leukemia/lymphoma with t(v;11q23);MLL rearranged
		9814/3	Leukemia/lymphoma with t(12;21)(p13;q22);TEL-AML1(ETV6-RUNX1)
		9815/3	B lymphoblastic leukemia/lymphoma with hyperdiploidy
		9816/3	Leukemia/lymphoma with hypodiploidy (hypodiploid ALL)
		9817/3	B lymphblastic leukemia/lymphoma with t(5;14)(q31;q32);IL3-IGH
		9818/3	Leukemia/lymphoma with t(1;19)(q23;p13.3); E2A PBX1 (TCF3 PBX1)
LYMPHOID LEUKEMIA, NOS	982	9823/3	Chronic lymphocytic leukemia/small lymphocytic lymphoma
PROLYMPH/PRECURS LEUKEMIA	983	9831/3	T-cell large granular lymphocytic leukemia
		9837/3	T lymphoblastic leukemia/lymphoma
CHRONIC MYELOPROLIFERATIVE DIS.	996	9965/3	Myeloid and lymphoid neoplasms with PDGFRB rearrangement
		9967/3	Myeloid and lymphoid neoplasm with FGFR1 abnormalities
MYELOPLASTIC/MYELOPROLIFERATIVE NEOPLASMS	997	9971/3	Polymorphic PTLD
		9975/3	Myelodysplastic/Myeloproliferative neoplasm, unclassifiable

THYMUS C379

NEOPLASM	800	8000/3	Neoplasm, malignant
		8001/3	Tumor cells, malignant
		8002/3	Malignant tumor, small cell type
		8003/3	Malignant tumor, giant cell type
		8004/3	Malignant tumor, spindle cell type
		8005/3	Malignant tumor, clear cell type
CARCINOMA, NOS	801	8010/2	Carcinoma in situ, NOS
		8010/3	Carcinoma, NOS
		8011/3	Epithelioma, malignant
		8012/3	Large cell carcinoma, NOS
		8013/3	Large cell neuroendocrine carcinoma
		8014/3	Large cell carcinoma with rhabdoid phenotype
		8015/3	Glassy cell carcinoma
CARCINOMA, UNDIFF., NOS	802	8020/3	Carcinoma, undifferentiated type, NOS
		8021/3	Carcinoma, anaplastic type, NOS
		8022/3	Pleomorphic carcinoma
		8023/3	NUT carcinoma
CARCINOID TUMOR, MALIGNANT	824	8240/3	Carcinoid tumor, malignant
		8241/3	Enterochromaffin cell carcinoid
		8242/3	Enterochromaffin-like cell tumor, malignant
		8243/3	Goblet cell carcinoid
		8244/3	Composite carcinoid
		8245/3	Adenocarcinoid tumor
		8246/3	Neuroendocrine carcinoma
		8249/3	Atypical carcinoid tumor
THYMOMA, MALIGNANT	858	8580/3	Thymoma, malignant, NOS
		8581/3	Thymoma, type A, malignant
		8582/3	Thymoma, type AB, malignant
		8583/3	Thymoma, type B1, malignant
		8584/3	Thymoma, type B2, malignant
		8585/3	Thymoma, type B3, malignant
		8586/3	Thymic carcinoma, NOS
		8588/3	Spindle epithelial tumor with thymus-like element
		8589/3	Carcinoma showing thymus-like element
TERATOMA	908	9086/3	Germ cell tumors with associated hematological malignancy
MALIGNANT LYMPHOMA, NOS	959	9590/3	Malignant lymphoma, NOS
		9591/3	Malignant lymphoma, non-Hodgkin
		9596/3	Composite Hodgkin and non-Hodgkin lymphoma

THYMUS C379

HODGKIN LYMPHOMA	965	9650/3	Hodgkin lymphoma, NOS
		9651/3	Hodgkin lymphoma, lymphocyte-rich
		9652/3	Hodgkin lymphoma, mixed cellularity, NOS
		9653/3	Hodgkin lymphoma, lymphocytic deplet., NOS
		9654/3	Hodgkin lymph., lymphocyt. deplet., diffuse fibrosis
		9655/3	Hodgkin lymphoma, lymphocyt. deplet., reticular
		9659/3	Hodgkin lymph., nodular lymphocyte predom.
HODGKIN LYMPHOMA, NOD. SCLER.	966	9661/3	Hodgkin granuloma [obs]
		9662/3	Hodgkin sarcoma [obs]
		9663/3	Hodgkin lymphoma, nodular sclerosis, NOS
		9664/3	Hodgkin lymphoma, nod. scler., cellular phase
		9665/3	Hodgkin lymphoma, nod. scler., grade 1
		9667/3	Hodgkin lymphoma, nod. scler., grade 2
ML, SMALL B-CELL LYMPHOCYTIC	967	9670/3	ML, small B lymphocytic, NOS
		9671/3	ML, lymphoplasmacytic
		9673/3	Mantle cell lymphoma
		9675/3	ML, mixed sm. and lg. cell, diffuse
		9679/3	Mediastinal large B-cell lymphoma
ML, LARGE B-CELL, DIFFUSE	968	9680/3	ML, large B-cell, diffuse
		9684/3	ML, large B-cell, diffuse, immunoblastic, NOS
		9687/3	Burkitt lymphoma, NOS
		9688/3	T-cell histiocyte rich large B-cell lymphoma
FOLLIC. & MARGINAL LYMPH, NOS	969	9690/3	Follicular lymphoma, NOS
		9691/3	Follicular lymphoma, grade 2
		9695/3	Follicular lymphoma, grade 1
		9698/3	Follicular lymphoma, grade 3
		9699/3	Marginal zone B-cell lymphoma, NOS
T-CELL LYMPHOMAS	970	9701/3	Sezary syndrome
		9702/3	Mature T-cell lymphoma, NOS
		9705/3	Angioimmunoblastic T-cell lymphoma
OTHER SPEC. NON-HODGKIN LYMPHOMA	971	9712/3	Intravascular large B-cell lymphoma
		9714/3	Anaplastic large cell lymphoma, T-cell and Null cell type
		9719/3	NK/T-cell lymphoma, nasal and nasal-type
PRECURS. CELL LYMPHOBLASTIC LYMPH.	972	9724/3	SystemicEBV pos. T-cell lymphoproliferative disease of childhood
		9727/3	Precursor cell lymphoblastic lymphoma, NOS
		9728/3	Precursor B-cell lymphoblastic lymphoma
		9729/3	Precursor T-cell lymphoblastic lymphoma

THYMUS C379

PLASMA CELL TUMORS	973	9731/3	Plasmacytoma, NOS
		9734/3	Plasmacytoma, extramedullary
		9735/3	Plasmablastic lymphoma
		9737/3	ALK positive large B-cell lymphoma
		9738/3	Lrg B-cell lymphoma in HHV8-assoc. multicentric Castleman DZ
MAST CELL TUMORS	974	9740/3	Mast cell sarcoma
		9741/3	Malignant mastocytosis
NEOPLASMS OF HISTIOCYTES AND ACCESSORY LYMPHOID CELLS	975	9750/3	Malignant histiocytosis
		9751/3	Langerhans cell histiocytosis, NOS
		9754/3	Langerhans cell histiocytosis, disseminated
		9755/3	Histiocytic sarcoma
		9756/3	Langerhans cell sarcoma
		9757/3	Interdigitating dendritic cell sarcoma
		9758/3	Follicular dendritic cell sarcoma
		9759/3	Fibroblastic reticular cell tumor
PRECURSOR LYMPHOID NEOPLASMS	981	9811/3	B lymphoblastic leukemia/lymphoma, NOS
		9812/3	Leukemia/lymphoma with t(9;22)(q34;q11.2);BCR-ABL1
		9813/3	Leukemia/lymphoma with t(v;11q23);MLL rearranged
		9814/3	Leukemia/lymphoma with t(12;21)(p13;q22);TEL-AML1(ETV6-RUNX1)
		9815/3	B lymphoblastic leukemia/lymphoma with hyperdiploidy
		9816/3	Leukemia/lymphoma with hypodiploidy (hypodiploid ALL)
		9817/3	B lymphblastic leukemia/lymphoma with t(5;14)(q31;q32);IL3-IGH
		9818/3	Leukemia/lymphoma with t(1;19)(q23;p13.3); E2A PBX1 (TCF3 PBX1)
LYMPHOID LEUKEMIA, NOS	982	9823/3	Chronic lymphocytic leukemia/small lymphocytic lymphoma
PROLYMPH/PRECURS LEUKEMIA	983	9831/3	T-cell large granular lymphocytic leukemia
		9837/3	T lymphoblastic leukemia/lymphoma
CHRONIC MYELOPROLIFERATIVE DIS.	996	9965/3	Myeloid and lymphoid neoplasms with PDGFRB rearrangement
		9967/3	Myeloid and lymphoid neoplasm with FGFR1 abnormalities
MYELOPLASTIC/MYELOPROLIFERATIVE NEOPLASMS	997	9971/3	Polymorphic PTLD
		9975/3	Myelodysplastic/Myeloproliferative neoplasm, unclassifiable

HEART C380

BLOOD VESSEL TUMORS	912	9120/3	Hemangiosarcoma
HEMANGIOENDOTHELIOMA	913	9137/3	Intimal sarcoma
MALIGNANT LYMPHOMA, NOS	959	9590/3	Malignant lymphoma, NOS
		9591/3	Malignant lymphoma, non-Hodgkin
		9596/3	Composite Hodgkin and non-Hodgkin lymphoma
HODGKIN LYMPHOMA	965	9650/3	Hodgkin lymphoma, NOS
		9651/3	Hodgkin lymphoma, lymphocyte-rich
		9652/3	Hodgkin lymphoma, mixed cellularity, NOS
		9653/3	Hodgkin lymphoma, lymphocytic deplet., NOS
		9654/3	Hodgkin lymph., lymphocyt. deplet., diffuse fibrosis
		9655/3	Hodgkin lymphoma, lymphocyt. deplet., reticular
		9659/3	Hodgkin lymph., nodular lymphocyte predom.
HODGKIN LYMPHOMA, NOD. SCLER.	966	9661/3	Hodgkin granuloma [obs]
		9662/3	Hodgkin sarcoma [obs]
		9663/3	Hodgkin lymphoma, nodular sclerosis, NOS
		9664/3	Hodgkin lymphoma, nod. scler., cellular phase
		9665/3	Hodgkin lymphoma, nod. scler., grade 1
		9667/3	Hodgkin lymphoma, nod. scler., grade 2
ML, SMALL B-CELL LYMPHOCYTIC	967	9670/3	ML, small B lymphocytic, NOS
		9671/3	ML, lymphoplasmacytic
		9673/3	Mantle cell lymphoma
		9675/3	ML, mixed sm. and lg. cell, diffuse
		9678/3	Primary effusion lymphoma
		9679/3	Mediastinal large B-cell lymphoma
ML, LARGE B-CELL, DIFFUSE	968	9680/3	ML, large B-cell, diffuse
		9684/3	ML, large B-cell, diffuse, immunoblastic, NOS
		9687/3	Burkitt lymphoma, NOS
		9688/3	T-cell histiocyte rich large B-cell lymphoma
		9689/3	Splenic marginal zone B-cell lymphoma
FOLLIC. & MARGINAL LYMPH, NOS	969	9690/3	Follicular lymphoma, NOS
		9691/3	Follicular lymphoma, grade 2
		9695/3	Follicular lymphoma, grade 1
		9698/3	Follicular lymphoma, grade 3
		9699/3	Marginal zone B-cell lymphoma, NOS
T-CELL LYMPHOMAS	970	9700/3	Mycosis fungoides
		9701/3	Sezary syndrome
		9702/3	Mature T-cell lymphoma, NOS
		9705/3	Angioimmunoblastic T-cell lymphoma
		9708/3	Subcutaneous panniculitis-like T-cell lymphoma

HEART C380

T-CELL LYMPHOMAS	970	9709/3	Cutaneous T-cell lymphoma, NOS
OTHER SPEC. NON-HODGKIN LYMPHOMA	971	9712/3	Intravascular large B-cell lymphoma
		9714/3	Anaplastic large cell lymphoma, T-cell and Null cell type
		9716/3	Hepatosplenic gamma-delta cell lymphoma
		9717/3	Intestinal T-cell lymphoma
		9718/3	Primary cutan. CD30+ T-cell lymphoprolif. disorder
		9719/3	NK/T-cell lymphoma, nasal and nasal-type
PRECURS. CELL LYMPHOBLASTIC LYMPH.	972	9724/3	SystemicEBV pos. T-cell lymphoproliferative disease of childhood
		9727/3	Precursor cell lymphoblastic lymphoma, NOS
		9728/3	Precursor B-cell lymphoblastic lymphoma
		9729/3	Precursor T-cell lymphoblastic lymphoma
PLASMA CELL TUMORS	973	9735/3	Plasmablastic lymphoma
		9737/3	ALK positive large B-cell lymphoma
		9738/3	Lrg B-cell lymphoma in HHV8-assoc. multicentric Castleman DZ
NEOPLASMS OF HISTIOCYTES AND ACCESSORY LYMPHOID CELLS	975	9751/3	Langerhans cell histiocytosis, NOS
		9759/3	Fibroblastic reticular cell tumor
PRECURSOR LYMPHOID NEOPLASMS	981	9811/3	B lymphoblastic leukemia/lymphoma, NOS
		9812/3	Leukemia/lymphoma with t(9;22)(q34;q11.2);BCR-ABL1
		9813/3	Leukemia/lymphoma with t(v;11q23);MLL rearranged
		9814/3	Leukemia/lymphoma with t(12;21)(p13;q22);TEL-AML1(ETV6-RUNX1)
		9815/3	B lymphoblastic leukemia/lymphoma with hyperdiploidy
		9816/3	Leukemia/lymphoma with hypodiploidy (hypodiploid ALL)
		9817/3	B lymphblastic leukemia/lymphoma with t(5;14)(q31;q32);IL3-IGH
		9818/3	Leukemia/lymphoma with t(1;19)(q23;p13.3); E2A PBX1 (TCF3 PBX1)
LYMPHOID LEUKEMIA, NOS	982	9823/3	Chronic lymphocytic leukemia/small lymphocytic lymphoma
PROLYMPH/PRECURS LEUKEMIA	983	9831/3	T-cell large granular lymphocytic leukemia
		9837/3	T lymphoblastic leukemia/lymphoma
CHRONIC MYELOPROLIFERATIVE DIS.	996	9965/3	Myeloid and lymphoid neoplasms with PDGFRB rearrangement
		9967/3	Myeloid and lymphoid neoplasm with FGFR1 abnormalities
MYELOPLASTIC/MYELOPROLIFERATIVE NEOPLASMS	997	9971/3	Polymorphic PTLD
		9975/3	Myelodysplastic/Myeloproliferative neoplasm, unclassifiable

MEDIASTINUM C381-C383, C388

NEOPLASM	800	8000/3	Neoplasm, malignant
		8001/3	Tumor cells, malignant
		8002/3	Malignant tumor, small cell type
		8003/3	Malignant tumor, giant cell type
		8004/3	Malignant tumor, spindle cell type
		8005/3	Malignant tumor, clear cell type
PARAGANGLIOMA	868	8680/3	Paraganglioma, malignant
EXTRA-ADRENAL PARAGANG., MAL	869	8693/3	Extra-adrenal paraganglioma, malignant
SARCOMA, NOS	880	8800/3	Sarcoma, NOS
		8801/3	Spindle cell sarcoma
		8802/3	Giant cell sarcoma
		8803/3	Small cell sarcoma
		8804/3	Epithelioid sarcoma
		8805/3	Undifferentiated sarcoma
		8806/3	Desmoplastic small round cell tumor
FIBROMATOUS NEOPLASMS	881	8810/3	Fibrosarcoma, NOS
		8811/3	Fibromyxosarcoma
		8813/3	Fascial fibrosarcoma
		8814/3	Infantile fibrosarcoma
		8815/3	Solitary fibrous tumor, malignant
SARCOMA, NOS	882	8825/3	Myofibroblastic sarcoma
FIBROUS HISTIOCYTOMA, MAL.	883	8830/3	Fibrous histiocytoma, malignant
LIPOSARCOMA NEOPLASMS	885	8850/3	Liposarcoma, NOS
		8851/3	Liposarcoma, well differentiated
		8852/3	Myxoid liposarcoma
		8853/3	Round cell liposarcoma
		8854/3	Pleomorphic liposarcoma
		8855/3	Mixed type liposarcoma
		8857/3	Fibroblastic liposarcoma
		8858/3	Dedifferentiated liposarcoma
MYOMATOUS NEOPLASMS	889	8890/3	Leiomyosarcoma, NOS
		8891/3	Epithelioid leiomyosarcoma
		8894/3	Angiomyosarcoma
		8895/3	Myosarcoma
		8896/3	Myxoid leiomyosarcoma

MEDIASTINUM C381-C383, C388

RHABDOMYOSARCOMA, NOS	890	8900/3	Rhabdomyosarcoma, NOS
		8901/3	Pleomorphic rhabdomyosarcoma, adult type
		8902/3	Mixed type rhabdomyosarcoma
EMBRYONAL RHABDOMYOSARCOMA	891	8910/3	Embryonal rhabdomyosarcoma
		8912/3	Spindle cell rhabdomyosarcoma
MESENCHYMOMA, MALIGNANT	899	8990/3	Mesenchymoma, malignant
		8991/3	Embryonal sarcoma
GERM CELL TUMORS	906	9060/3	Dysgerminoma
		9061/3	Seminoma, NOS
		9062/3	Seminoma, anaplastic
		9063/3	Spermatocytic seminoma
		9064/3	Germinoma
		9065/3	Germ cell tumor, nonseminomatous
EMBRYONAL CARCINOMA, NOS	907	9070/3	Embryonal carcinoma, NOS
		9071/3	Yolk sac tumor
		9072/3	Polyembryoma
TERATOMA	908	9080/3	Teratoma, malignant, NOS
		9081/3	Teratocarcinoma
		9082/3	Malignant teratoma, undiff.
		9083/3	Malignant teratoma, intermediate
		9084/3	Teratoma with malig. transformation
		9085/3	Mixed germ cell tumor
CHORIOCARCINOMA	910	9100/3	Choriocarcinoma
		9101/3	Choriocarcinoma combined w/ other germ cell elements
		9105/3	Trophoblastic tumor, epithelioid
GANGLIONEUROBLASTOMA	949	9490/3	Ganglioneuroblastoma
NEUROBLASTOMA, NOS	950	9500/3	Neuroblastoma, NOS
		9501/3	Medulloepithelioma, NOS
		9502/3	Teratoid medulloepithelioma
		9503/3	Neuroepithelioma, NOS
		9504/3	Spongioneuroblastoma
		9505/3	Ganglioglioma, anaplastic
NEUROFIBROSARCOMA	954	9540/3	Malignant peripheral nerve sheath tumor

MEDIASTINUM C381-C383,C388

NEURILEMMOMA	956	9560/3	Neurilemmoma, malignant
		9561/3	MPNST with rhabdomyoblastic differentiation
PERINEURIOMA	957	9571/3	Perineurioma, malignant
MALIGNANT LYMPHOMA, NOS	959	9590/3	Malignant lymphoma, NOS
		9591/3	Malignant lymphoma, non-Hodgkin
		9596/3	Composite Hodgkin and non-Hodgkin lymphoma
HODGKIN LYMPHOMA	965	9650/3	Hodgkin lymphoma, NOS
		9651/3	Hodgkin lymphoma, lymphocyte-rich
		9652/3	Hodgkin lymphoma, mixed cellularity, NOS
		9653/3	Hodgkin lymphoma, lymphocytic deplet., NOS
		9654/3	Hodgkin lymph., lymphocyt. deplet., diffuse fibrosis
		9655/3	Hodgkin lymphoma, lymphocyt. deplet., reticular
		9659/3	Hodgkin lymph., nodular lymphocyte predom.
HODGKIN LYMPHOMA, NOD. SCLER.	966	9661/3	Hodgkin granuloma [obs]
		9662/3	Hodgkin sarcoma [obs]
		9663/3	Hodgkin lymphoma, nodular sclerosis, NOS
		9664/3	Hodgkin lymphoma, nod. scler., cellular phase
		9665/3	Hodgkin lymphoma, nod. scler., grade 1
		9667/3	Hodgkin lymphoma, nod. scler., grade 2
ML, SMALL B-CELL LYMPHOCYTIC	967	9670/3	ML, small B lymphocytic, NOS
		9671/3	ML, lymphoplasmacytic
		9673/3	Mantle cell lymphoma
		9675/3	ML, mixed sm. and lg. cell, diffuse
		9678/3	Primary effusion lymphoma
		9679/3	Mediastinal large B-cell lymphoma
ML, LARGE B-CELL, DIFFUSE	968	9680/3	ML, large B-cell, diffuse
		9684/3	ML, large B-cell, diffuse, immunoblastic, NOS
		9687/3	Burkitt lymphoma, NOS
		9688/3	T-cell histiocyte rich large B-cell lymphoma
FOLLIC. & MARGINAL LYMPH, NOS	969	9690/3	Follicular lymphoma, NOS
		9691/3	Follicular lymphoma, grade 2
		9695/3	Follicular lymphoma, grade 1
		9698/3	Follicular lymphoma, grade 3
		9699/3	Marginal zone B-cell lymphoma, NOS
T-CELL LYMPHOMAS	970	9701/3	Sezary syndrome
		9702/3	Mature T-cell lymphoma, NOS
		9705/3	Angioimmunoblastic T-cell lymphoma

MEDIASTINUM C381-C383, C388

OTHER SPEC. NON-HODGKIN LYMPHOMA	971	9712/3	Intravascular large B-cell lymphoma
		9714/3	Anaplastic large cell lymphoma, T-cell and Null cell type
		9719/3	NK/T-cell lymphoma, nasal and nasal-type
PRECURS. CELL LYMPHOBLASTIC LYMPH.	972	9724/3	SystemicEBV pos. T-cell lymphoproliferative disease of childhood
		9727/3	Precursor cell lymphoblastic lymphoma, NOS
		9728/3	Precursor B-cell lymphoblastic lymphoma
		9729/3	Precursor T-cell lymphoblastic lymphoma
PLASMA CELL TUMORS	973	9731/3	Plasmacytoma, NOS
		9734/3	Plasmacytoma, extramedullary
		9735/3	Plasmablastic lymphoma
		9737/3	ALK positive large B-cell lymphoma
		9738/3	Lrg B-cell lymphoma in HHV8-assoc. multicentric Castleman DZ
MAST CELL TUMORS	974	9740/3	Mast cell sarcoma
		9741/3	Malignant mastocytosis
NEOPLASMS OF HISTIOCYTES AND ACCESSORY LYMPHOID CELLS	975	9750/3	Malignant histiocytosis
		9751/3	Langerhans cell histiocytosis, NOS
		9754/3	Langerhans cell histiocytosis, disseminated
		9755/3	Histiocytic sarcoma
		9756/3	Langerhans cell sarcoma
		9757/3	Interdigitating dendritic cell sarcoma
		9758/3	Follicular dendritic cell sarcoma
		9759/3	Fibroblastic reticular cell tumor
PRECURSOR LYMPHOID NEOPLASMS	981	9811/3	B lymphoblastic leukemia/lymphoma, NOS
		9812/3	Leukemia/lymphoma with t(9;22)(q34;q11.2);BCR-ABL1
		9813/3	Leukemia/lymphoma with t(v;11q23);MLL rearranged
		9814/3	Leukemia/lymphoma with t(12;21)(p13;q22);TEL-AML1(ETV6-RUNX1)
		9815/3	B lymphoblastic leukemia/lymphoma with hyperdiploidy
		9816/3	Leukemia/lymphoma with hypodiploidy (hypodiploid ALL)
		9817/3	B lymphblastic leukemia/lymphoma with t(5;14)(q31;q32);IL3-IGH
		9818/3	Leukemia/lymphoma with t(1;19)(q23;p13.3); E2A PBX1 (TCF3 PBX1)
LYMPHOID LEUKEMIA, NOS	982	9823/3	Chronic lymphocytic leukemia/small lymphocytic lymphoma
PROLYMPH/PRECURS LEUKEMIA	983	9831/3	T-cell large granular lymphocytic leukemia
		9837/3	T lymphoblastic leukemia/lymphoma
CHRONIC MYELOPROLIFERATIVE DIS.	996	9965/3	Myeloid and lymphoid neoplasms with PDGFRB rearrangement
		9967/3	Myeloid and lymphoid neoplasm with FGFR1 abnormalities
MYELOPLASTIC/MYELOPROLIFERATIVE NEOPLASMS	997	9971/3	Polymorphic PTLD
		9975/3	Myelodysplastic/Myeloproliferative neoplasm, unclassifiable

PLEURA C384

NEOPLASM	800	8000/3	Neoplasm, malignant
		8001/3	Tumor cells, malignant
		8002/3	Malignant tumor, small cell type
		8003/3	Malignant tumor, giant cell type
		8004/3	Malignant tumor, spindle cell type
		8005/3	Malignant tumor, clear cell type
SARCOMA, NOS	880	8800/3	Sarcoma, NOS
		8801/3	Spindle cell sarcoma
		8802/3	Giant cell sarcoma
		8803/3	Small cell sarcoma
		8804/3	Epithelioid sarcoma
		8805/3	Undifferentiated sarcoma
		8806/3	Desmoplastic small round cell tumor
FIBROMATOUS NEOPLASMS	881	8810/3	Fibrosarcoma, NOS
		8811/3	Fibromyxosarcoma
		8813/3	Fascial fibrosarcoma
		8814/3	Infantile fibrosarcoma
		8815/3	Solitary fibrous tumor, malignant
SARCOMA, NOS	882	8825/3	Myofibroblastic sarcoma
FIBROUS HISTIOCYTOMA, MAL.	883	8830/3	Fibrous histiocytoma, malignant
HEPATOBLASTOMA	897	8973/3	Pleuropulmonary blastoma
MESOTHELIOMA, MALIGNANT	905	9050/3	Mesothelioma, malignant
		9051/3	Fibrous mesothelioma, malignant
		9052/3	Epithel. mesothelioma, mal.
		9053/3	Mesothelioma, biphasic, malignant
MALIGNANT LYMPHOMA, NOS	959	9590/3	Malignant lymphoma, NOS
		9591/3	Malignant lymphoma, non-Hodgkin
		9596/3	Composite Hodgkin and non-Hodgkin lymphoma
HODGKIN LYMPHOMA	965	9650/3	Hodgkin lymphoma, NOS
		9651/3	Hodgkin lymphoma, lymphocyte-rich
		9652/3	Hodgkin lymphoma, mixed cellularity, NOS
		9653/3	Hodgkin lymphoma, lymphocytic deplet., NOS
		9654/3	Hodgkin lymph., lymphocyt. deplet., diffuse fibrosis
		9655/3	Hodgkin lymphoma, lymphocyt. deplet., reticular
		9659/3	Hodgkin lymph., nodular lymphocyte predom.

PLEURA C384

HODGKIN LYMPHOMA, NOD. SCLER.	966	9661/3	Hodgkin granuloma [obs]
		9662/3	Hodgkin sarcoma [obs]
		9663/3	Hodgkin lymphoma, nodular sclerosis, NOS
		9664/3	Hodgkin lymphoma, nod. scler., cellular phase
		9665/3	Hodgkin lymphoma, nod. scler., grade 1
		9667/3	Hodgkin lymphoma, nod. scler., grade 2
ML, SMALL B-CELL LYMPHOCYTIC	967	9670/3	ML, small B lymphocytic, NOS
		9671/3	ML, lymphoplasmacytic
		9673/3	Mantle cell lymphoma
		9675/3	ML, mixed sm. and lg. cell, diffuse
		9678/3	Primary effusion lymphoma
ML, LARGE B-CELL, DIFFUSE	968	9680/3	ML, large B-cell, diffuse
		9684/3	ML, large B-cell, diffuse, immunoblastic, NOS
		9687/3	Burkitt lymphoma, NOS
		9688/3	T-cell histiocyte rich large B-cell lymphoma
FOLLIC. & MARGINAL LYMPH, NOS	969	9690/3	Follicular lymphoma, NOS
		9691/3	Follicular lymphoma, grade 2
		9695/3	Follicular lymphoma, grade 1
		9698/3	Follicular lymphoma, grade 3
		9699/3	Marginal zone B-cell lymphoma, NOS
T-CELL LYMPHOMAS	970	9701/3	Sezary syndrome
		9702/3	Mature T-cell lymphoma, NOS
		9705/3	Angioimmunoblastic T-cell lymphoma
OTHER SPEC. NON-HODGKIN LYMPHOMA	971	9712/3	Intravascular large B-cell lymphoma
		9714/3	Anaplastic large cell lymphoma, T-cell and Null cell type
		9719/3	NK/T-cell lymphoma, nasal and nasal-type
PRECURS. CELL LYMPHOBLASTIC LYMPH.	972	9724/3	SystemicEBV pos. T-cell lymphoproliferative disease of childhood
		9727/3	Precursor cell lymphoblastic lymphoma, NOS
		9728/3	Precursor B-cell lymphoblastic lymphoma
		9729/3	Precursor T-cell lymphoblastic lymphoma
PLASMA CELL TUMORS	973	9731/3	Plasmacytoma, NOS
		9734/3	Plasmacytoma, extramedullary
		9735/3	Plasmablastic lymphoma
		9737/3	ALK positive large B-cell lymphoma
		9738/3	Lrg B-cell lymphoma in HHV8-assoc. multicentric Castleman DZ
MAST CELL TUMORS	974	9740/3	Mast cell sarcoma
		9741/3	Malignant mastocytosis

PLEURA C384

NEOPLASMS OF HISTIOCYTES AND ACCESSORY LYMPHOID CELLS	975	9750/3	Malignant histiocytosis
		9751/3	Langerhans cell histiocytosis, NOS
		9754/3	Langerhans cell histiocytosis, disseminated
		9755/3	Histiocytic sarcoma
		9756/3	Langerhans cell sarcoma
		9757/3	Interdigitating dendritic cell sarcoma
		9758/3	Follicular dendritic cell sarcoma
		9759/3	Fibroblastic reticular cell tumor
PRECURSOR LYMPHOID NEOPLASMS	981	9811/3	B lymphoblastic leukemia/lymphoma, NOS
		9812/3	Leukemia/lymphoma with t(9;22)(q34;q11.2);BCR-ABL1
		9813/3	Leukemia/lymphoma with t(v;11q23);MLL rearranged
		9814/3	Leukemia/lymphoma with t(12;21)(p13;q22);TEL-AML1(ETV6-RUNX1)
		9815/3	B lymphoblastic leukemia/lymphoma with hyperdiploidy
		9816/3	Leukemia/lymphoma with hypodiploidy (hypodiploid ALL)
		9817/3	B lymphblastic leukemia/lymphoma with t(5;14)(q31;q32);IL3-IGH
		9818/3	Leukemia/lymphoma with t(1;19)(q23;p13.3); E2A PBX1 (TCF3 PBX1)
LYMPHOID LEUKEMIA, NOS	982	9823/3	Chronic lymphocytic leukemia/small lymphocytic lymphoma
PROLYMPH/PRECURS LEUKEMIA	983	9831/3	T-cell large granular lymphocytic leukemia
		9837/3	T lymphoblastic leukemia/lymphoma
CHRONIC MYELOPROLIFERATIVE DIS.	996	9965/3	Myeloid and lymphoid neoplasms with PDGFRB rearrangement
		9967/3	Myeloid and lymphoid neoplasm with FGFR1 abnormalities
MYELOPLASTIC/MYELOPROLIFERATIVE NEOPLASMS	997	9971/3	Polymorphic PTLD
		9975/3	Myelodysplastic/Myeloproliferative neoplasm, unclassifiable

RESPIRATORY,NOS C390,C398-C399

NEOPLASM	800	8000/3	Neoplasm, malignant
		8001/3	Tumor cells, malignant
		8002/3	Malignant tumor, small cell type
		8003/3	Malignant tumor, giant cell type
		8004/3	Malignant tumor, spindle cell type
		8005/3	Malignant tumor, clear cell type
CARCINOMA, NOS	801	8010/2	Carcinoma in situ, NOS
		8010/3	Carcinoma, NOS
		8011/3	Epithelioma, malignant
		8012/3	Large cell carcinoma, NOS
		8013/3	Large cell neuroendocrine carcinoma
		8014/3	Large cell carcinoma with rhabdoid phenotype
		8015/3	Glassy cell carcinoma
CARCINOMA, UNDIFF., NOS	802	8020/3	Carcinoma, undifferentiated type, NOS
		8021/3	Carcinoma, anaplastic type, NOS
		8022/3	Pleomorphic carcinoma
GIANT & SPINDLE CELL CARCINOMA	803	8030/3	Giant cell and spindle cell carcinoma
		8031/3	Giant cell carcinoma
		8032/3	Spindle cell carcinoma
		8033/3	Pseudosarcomatous carcinoma
		8034/3	Polygonal cell carcinoma
		8035/3	Carcinoma with osteoclast-like giant cells
SMALL CELL CARCINOMA, NOS	804	8041/3	Small cell carcinoma, NOS
		8043/3	Small cell carcinoma, fusiform cell
PAPILLARY CARCINOMA, NOS	805	8050/2	Papillary carcinoma in situ
		8050/3	Papillary carcinoma, NOS
		8051/3	Verrucous carcinoma, NOS
		8052/2	Papillary squamous cell carcinoma, non-invasive
		8052/3	Papillary squamous cell carcinoma
SQUAMOUS CELL CARCINOMA, NOS	807	8070/2	Squamous cell carcinoma in situ, NOS
		8070/3	Squamous cell carcinoma, NOS
		8071/3	Sq. cell carcinoma, keratinizing, NOS
		8072/3	Sq. cell carcinoma, lg. cell, non-ker.
		8073/3	Sq. cell carcinoma, sm. cell, non-ker.
		8074/3	Sq. cell carcinoma, spindle cell
		8075/3	Squamous cell carcinoma, adenoid
		8076/2	Sq. cell carc. in situ with question. stromal invas.
		8076/3	Sq. cell carcinoma, micro-invasive
		8078/3	Squamous cell carcinoma with horn formation

RESPIRATORY,NOS C390,C398-C399

TRANSITIONAL CELL CARCINOMA, NOS	812	8120/2	Transitional cell carcinoma in situ
		8120/3	Transitional cell carcinoma, NOS
		8121/3	Schneiderian carcinoma
		8122/3	Trans. cell carcinoma, spindle cell
		8123/3	Basaloid carcinoma
		8124/3	Cloacogenic carcinoma
ADENOCARCINOMA, NOS	814	8140/2	Adenocarcinoma in situ
		8140/3	Adenocarcinoma, NOS
		8141/3	Scirrhous adenocarcinoma
		8143/3	Superficial spreading adenocarcinoma
		8147/3	Basal cell adenocarcinoma
ADENOID CYSTIC & CRIBRIFORM CA.	820	8200/3	Adenoid cystic carcinoma
		8201/2	Cribriform carcinoma in situ
		8201/3	Cribriform carcinoma
SOLID CARCINOMA, NOS	823	8230/2	Duct carcinoma in situ, solid type
		8230/3	Solid carcinoma, NOS
		8231/3	Carcinoma simplex
CARCINOID TUMOR, MALIGNANT	824	8240/3	Carcinoid tumor, malignant
		8241/3	Enterochromaffin cell carcinoid
		8242/3	Enterochromaffin-like cell tumor, malignant
		8243/3	Goblet cell carcinoid
		8244/3	Composite carcinoid
		8245/3	Adenocarcinoid tumor
		8246/3	Neuroendocrine carcinoma
		8249/3	Atypical carcinoid tumor
BRONCHIOLO-ALVEOLAR ADENOCA.	825	8250/3	Lepidic adenocarcinoma
		8251/3	Alveolar adenocarcinoma
		8252/3	Bronchiolo-alveolar carcinoma, non-mucinous
		8253/3	Invasive mucinous adenocarcinoma
		8254/3	Mixed invasive mucinous and non-mucinous adenocarcinoma
		8255/3	Adenocarcinoma with mixed subtypes
PAPILLARY ADENOCARCINOMA, NOS	826	8260/3	Papillary adenocarcinoma, NOS
		8261/2	Adenocarcinoma in situ in villous adenoma
		8261/3	Adenocarcinoma in villous adenoma
		8262/3	Villous adenocarcinoma
		8263/2	Adenocarcinoma in situ in tubulovillous adenoma
		8263/3	Adenocarcinoma in tubulovillous adenoma
CLEAR CELL ADENOCARCINOMA, NOS	831	8310/3	Clear cell adenocarcinoma, NOS

RESPIRATORY,NOS C390,C398-C399

GRANULAR CELL CARCINOMA	832	8320/3	Granular cell carcinoma
		8323/3	Mixed cell adenocarcinoma
MUCOEPIDERMOID CARCINOMA	843	8430/3	Mucoepidermoid carcinoma
MUCINOUS ADENOCARCINOMA	848	8480/3	Mucinous adenocarcinoma
		8481/3	Mucin-producing adenocarcinoma
SIGNET RING CELL CARCINOMA	849	8490/3	Signet ring cell carcinoma
MEDULLARY CARCINOMA, NOS	851	8510/3	Medullary carcinoma, NOS
ACINAR CELL CARCINOMA	855	8550/3	Acinar cell carcinoma
		8551/3	Acinar cell cystadenocarcinoma
ADENOSQUAMOUS CARCINOMA	856	8560/3	Adenosquamous carcinoma
		8562/3	Epithelial-myoepithelial carcinoma
ADENOCA. WITH METAPLASIA	857	8570/3	Adenocarcinoma with squamous metaplasia
		8571/3	Adenocarcinoma w cartilag. & oss. metaplas.
		8572/3	Adenocarcinoma with spindle cell mataplasia
		8573/3	Adenocarcinoma with apocrine metaplasia
		8574/3	Adenocarcinoma with neuroendocrine differen.
		8575/3	Metaplastic carcinoma, NOS
		8576/3	Hepatoid adenocarcinoma
SARCOMA, NOS	880	8800/3	Sarcoma, NOS
		8801/3	Spindle cell sarcoma
		8802/3	Giant cell sarcoma
		8803/3	Small cell sarcoma
		8804/3	Epithelioid sarcoma
		8805/3	Undifferentiated sarcoma
		8806/3	Desmoplastic small round cell tumor
FIBROMATOUS NEOPLASMS	881	8810/3	Fibrosarcoma, NOS
		8811/3	Fibromyxosarcoma
		8813/3	Fascial fibrosarcoma
		8814/3	Infantile fibrosarcoma
		8815/3	Solitary fibrous tumor, malignant
SARCOMA, NOS	882	8825/3	Myofibroblastic sarcoma
FIBROUS HISTIOCYTOMA, MAL.	883	8830/3	Fibrous histiocytoma, malignant

RESPIRATORY,NOS C390,C398-C399

MYOMATOUS NEOPLASMS	889	8890/3	Leiomyosarcoma, NOS
		8891/3	Epithelioid leiomyosarcoma
		8894/3	Angiomyosarcoma
		8895/3	Myosarcoma
		8896/3	Myxoid leiomyosarcoma
RHABDOMYOSARCOMA, NOS	890	8900/3	Rhabdomyosarcoma, NOS
		8901/3	Pleomorphic rhabdomyosarcoma, adult type
		8902/3	Mixed type rhabdomyosarcoma
EMBRYONAL RHABDOMYOSARCOMA	891	8910/3	Embryonal rhabdomyosarcoma
		8912/3	Spindle cell rhabdomyosarcoma
CARCINOSARCOMA, NOS	898	8980/3	Carcinosarcoma, NOS
		8981/3	Carcinosarcoma, embryonal type
		8982/3	Malignant myoepithelioma
MESENCHYMOMA, MALIGNANT	899	8990/3	Mesenchymoma, malignant
		8991/3	Embryonal sarcoma
TERATOMA	908	9080/3	Teratoma, malignant, NOS
		9081/3	Teratocarcinoma
		9082/3	Malignant teratoma, undiff.
		9083/3	Malignant teratoma, intermediate
		9084/3	Teratoma with malig. transformation
		9085/3	Mixed germ cell tumor
BLOOD VESSEL TUMORS	912	9120/3	Hemangiosarcoma
PERIPHERAL NEUROECTODERMAL TUMOR	936	9364/3	Peripheral neuroectodermal tumor
		9365/3	Askin tumor
LYMPHOID LEUKEMIA, NOS	982	9823/3	Chronic lymphocytic leukemia/small lymphocytic lymphoma

BONES & JOINTS (EXCL SKULL AND FACE, MANDIBLE) C400-C403,C408-C409,C412-C414,C418-C419

NEOPLASM	800	8000/3	Neoplasm, malignant
		8001/3	Tumor cells, malignant
		8002/3	Malignant tumor, small cell type
		8003/3	Malignant tumor, giant cell type
		8004/3	Malignant tumor, spindle cell type
		8005/3	Malignant tumor, clear cell type
SARCOMA, NOS	880	8800/3	Sarcoma, NOS
		8801/3	Spindle cell sarcoma
		8802/3	Giant cell sarcoma
		8803/3	Small cell sarcoma
		8804/3	Epithelioid sarcoma
		8805/3	Undifferentiated sarcoma
		8806/3	Desmoplastic small round cell tumor
FIBROMATOUS NEOPLASMS	881	8810/3	Fibrosarcoma, NOS
		8811/3	Fibromyxosarcoma
		8812/3	Periosteal fibrosarcoma
		8813/3	Fascial fibrosarcoma
		8814/3	Infantile fibrosarcoma
		8815/3	Solitary fibrous tumor, malignant
SARCOMA, NOS	882	8825/3	Myofibroblastic sarcoma
FIBROUS HISTIOCYTOMA, MAL.	883	8830/3	Fibrous histiocytoma, malignant
MYXOSARCOMA	884	8840/3	Myxosarcoma
LIPOSARCOMA NEOPLASMS	885	8850/3	Liposarcoma, NOS
		8851/3	Liposarcoma, well differentiated
		8852/3	Myxoid liposarcoma
		8853/3	Round cell liposarcoma
		8854/3	Pleomorphic liposarcoma
		8855/3	Mixed type liposarcoma
		8857/3	Fibroblastic liposarcoma
		8858/3	Dedifferentiated liposarcoma
SYNOVIAL SARCOMA, NOS	904	9040/3	Synovial sarcoma, NOS
		9041/3	Synovial sarcoma, spindle cell
		9042/3	Synovial sarcoma, epithelioid cell
		9043/3	Synovial sarcoma, biphasic
BLOOD VESSEL TUMORS	912	9120/3	Hemangiosarcoma

BONES & JOINTS (EXCL SKULL AND FACE, MANDIBLE) C400-C403,C408-C409,C412-C414,C418-C419

HEMANGIOENDOTHELIOMA	913	9130/3	Hemangioendothelioma, malignant
		9133/3	Epithelioid hemangioendothelioma, malignant
OSTEOSARCOMA, NOS	918	9180/3	Osteosarcoma, NOS
		9181/3	Chondroblastic osteosarcoma
		9182/3	Fibroblastic osteosarcoma
		9183/3	Telangiectatic osteosarcoma
		9184/3	Osteosarcoma in Paget disease
		9185/3	Small cell osteosarcoma
		9186/3	Central osteosarcoma
		9187/3	Instrosseous well differentiated osteosarcoma
JUXTACORTICAL OSTEOSARCOMA	919	9192/3	Parosteal osteosarcoma
		9193/3	Periosteal osteosarcoma
		9194/3	High grade surface osteosarcoma
		9195/3	Intracortical osteosarcoma
CHONDROSARCOMA, NOS	922	9220/3	Chondrosarcoma, NOS
		9221/3	Juxtacortical chondrosarcoma
CHONDROBLASTOMA, MALIGNANT	923	9230/3	Chondroblastoma, malignant
		9231/3	Myxoid chondrosarcoma
OSSEOUS & CHONDROMATOUS NEOPLASMS	924	9240/3	Mesenchymal chondrosarcoma
		9242/3	Clear cell chondrosarcoma
		9243/3	Dedifferentiated chondrosarcoma
GT. CELL TUMOR OF BONE, MAL.	925	9250/3	Giant cell tumor of bone, malignant
		9251/3	Malignant giant cell tumor of soft parts
EWING SARCOMA	926	9260/3	Ewing sarcoma
		9261/3	Adamantinoma of long bones
ODONTOGENIC TUMOR, MAL.	927	9270/3	Odontogenic tumor, malignant
AMELOBLASTIC ODONTOSARCOMA	929	9290/3	Ameloblastic odontosarcoma
AMELOBLASTOMA, MALIGNANT	931	9310/3	Ameloblastoma, malignant
AMELOBLASTIC FIBROSARCOMA	933	9330/3	Ameloblastic fibrosarcoma
ODONTOGENIC CARCINOSARCOMA	934	9342/3	Odontogenic carcinosarcoma
PERIPHERAL NEUROECTODERMAL TUMOR	936	9364/3	Peripheral neuroectodermal tumor
		9365/3	Askin tumor

BONES & JOINTS (EXCL SKULL AND FACE, MANDIBLE) C400-C403,C408-C409,C412-C414,C418-C419

CHORDOMA	937	9370/3	Chordoma, NOS
		9371/3	Chondroid chordoma
		9372/3	Dedifferentiated chordoma
MALIGNANT LYMPHOMA, NOS	959	9590/3	Malignant lymphoma, NOS
		9591/3	Malignant lymphoma, non-Hodgkin
		9596/3	Composite Hodgkin and non-Hodgkin lymphoma
HODGKIN LYMPHOMA	965	9650/3	Hodgkin lymphoma, NOS
		9651/3	Hodgkin lymphoma, lymphocyte-rich
		9652/3	Hodgkin lymphoma, mixed cellularity, NOS
		9653/3	Hodgkin lymphoma, lymphocytic deplet., NOS
		9654/3	Hodgkin lymph., lymphocyt. deplet., diffuse fibrosis
		9655/3	Hodgkin lymphoma, lymphocyt. deplet., reticular
		9659/3	Hodgkin lymph., nodular lymphocyte predom.
HODGKIN LYMPHOMA, NOD. SCLER.	966	9661/3	Hodgkin granuloma [obs]
		9662/3	Hodgkin sarcoma [obs]
		9663/3	Hodgkin lymphoma, nodular sclerosis, NOS
		9664/3	Hodgkin lymphoma, nod. scler., cellular phase
		9665/3	Hodgkin lymphoma, nod. scler., grade 1
		9667/3	Hodgkin lymphoma, nod. scler., grade 2
ML, SMALL B-CELL LYMPHOCYTIC	967	9670/3	ML, small B lymphocytic, NOS
		9671/3	ML, lymphoplasmacytic
		9673/3	Mantle cell lymphoma
		9675/3	ML, mixed sm. and lg. cell, diffuse
ML, LARGE B-CELL, DIFFUSE	968	9680/3	ML, large B-cell, diffuse
		9684/3	ML, large B-cell, diffuse, immunoblastic, NOS
		9687/3	Burkitt lymphoma, NOS
		9688/3	T-cell histiocyte rich large B-cell lymphoma
FOLLIC. & MARGINAL LYMPH, NOS	969	9690/3	Follicular lymphoma, NOS
		9691/3	Follicular lymphoma, grade 2
		9695/3	Follicular lymphoma, grade 1
		9698/3	Follicular lymphoma, grade 3
		9699/3	Marginal zone B-cell lymphoma, NOS
T-CELL LYMPHOMAS	970	9701/3	Sezary syndrome
		9702/3	Mature T-cell lymphoma, NOS
		9705/3	Angioimmunoblastic T-cell lymphoma

BONES & JOINTS (EXCL SKULL AND FACE, MANDIBLE) C400-C403,C408-C409,C412-C414,C418-C419

OTHER SPEC. NON-HODGKIN LYMPHOMA	971	9712/3	Intravascular large B-cell lymphoma
		9714/3	Anaplastic large cell lymphoma, T-cell and Null cell type
		9716/3	Hepatosplenic gamma-delta cell lymphoma
		9719/3	NK/T-cell lymphoma, nasal and nasal-type
PRECURS. CELL LYMPHOBLASTIC LYMPH.	972	9724/3	SystemicEBV pos. T-cell lymphoproliferative disease of childhood
		9727/3	Precursor cell lymphoblastic lymphoma, NOS
		9728/3	Precursor B-cell lymphoblastic lymphoma
		9729/3	Precursor T-cell lymphoblastic lymphoma
PLASMA CELL TUMORS	973	9731/3	Plasmacytoma, NOS
		9732/3	Multiple myeloma
		9735/3	Plasmablastic lymphoma
		9737/3	ALK positive large B-cell lymphoma
		9738/3	Lrg B-cell lymphoma in HHV8-assoc. multicentric Castleman DZ
MAST CELL TUMORS	974	9740/3	Mast cell sarcoma
		9741/3	Malignant mastocytosis
NEOPLASMS OF HISTIOCYTES AND ACCESSORY LYMPHOID CELLS	975	9750/3	Malignant histiocytosis
		9751/3	Langerhans cell histiocytosis, NOS
		9754/3	Langerhans cell histiocytosis, disseminated
		9755/3	Histiocytic sarcoma
		9756/3	Langerhans cell sarcoma
		9757/3	Interdigitating dendritic cell sarcoma
		9758/3	Follicular dendritic cell sarcoma
		9759/3	Fibroblastic reticular cell tumor
PRECURSOR LYMPHOID NEOPLASMS	981	9811/3	B lymphoblastic leukemia/lymphoma, NOS
		9812/3	Leukemia/lymphoma with t(9;22)(q34;q11.2);BCR-ABL1
		9813/3	Leukemia/lymphoma with t(v;11q23);MLL rearranged
		9814/3	Leukemia/lymphoma with t(12;21)(p13;q22);TEL-AML1(ETV6-RUNX1)
		9815/3	B lymphoblastic leukemia/lymphoma with hyperdiploidy
		9816/3	Leukemia/lymphoma with hypodiploidy (hypodiploid ALL)
		9817/3	B lymphblastic leukemia/lymphoma with t(5;14)(q31;q32);IL3-IGH
		9818/3	Leukemia/lymphoma with t(1;19)(q23;p13.3); E2A PBX1 (TCF3 PBX1)
LYMPHOID LEUKEMIA, NOS	982	9823/3	Chronic lymphocytic leukemia/small lymphocytic lymphoma
PROLYMPH/PRECURS LEUKEMIA	983	9831/3	T-cell large granular lymphocytic leukemia
		9837/3	T lymphoblastic leukemia/lymphoma
CHRONIC MYELOPROLIFERATIVE DIS.	996	9965/3	Myeloid and lymphoid neoplasms with PDGFRB rearrangement
		9967/3	Myeloid and lymphoid neoplasm with FGFR1 abnormalities

BONES & JOINTS (EXCL SKULL AND FACE, MANDIBLE) C400-C403,C408-C409,C412-C414,C418-C419

MYELOPLASTIC/MYELOPROLIFERATIVE NEOPLASMS	997	9971/3	Polymorphic PTLD
		9975/3	Myelodysplastic/Myeloproliferative neoplasm, unclassifiable

BONES OF SKULL AND FACE C410

NEOPLASM	800	8000/3	Neoplasm, malignant
		8001/3	Tumor cells, malignant
		8002/3	Malignant tumor, small cell type
		8003/3	Malignant tumor, giant cell type
		8004/3	Malignant tumor, spindle cell type
		8005/3	Malignant tumor, clear cell type
SARCOMA, NOS	880	8800/3	Sarcoma, NOS
		8801/3	Spindle cell sarcoma
		8802/3	Giant cell sarcoma
		8803/3	Small cell sarcoma
		8804/3	Epithelioid sarcoma
		8805/3	Undifferentiated sarcoma
		8806/3	Desmoplastic small round cell tumor
FIBROMATOUS NEOPLASMS	881	8810/3	Fibrosarcoma, NOS
		8811/3	Fibromyxosarcoma
		8812/3	Periosteal fibrosarcoma
		8813/3	Fascial fibrosarcoma
		8814/3	Infantile fibrosarcoma
		8815/3	Solitary fibrous tumor, malignant
SARCOMA, NOS	882	8825/3	Myofibroblastic sarcoma
FIBROUS HISTIOCYTOMA, MAL.	883	8830/3	Fibrous histiocytoma, malignant
MYXOSARCOMA	884	8840/3	Myxosarcoma
LIPOSARCOMA NEOPLASMS	885	8850/3	Liposarcoma, NOS
		8851/3	Liposarcoma, well differentiated
		8852/3	Myxoid liposarcoma
		8853/3	Round cell liposarcoma
		8854/3	Pleomorphic liposarcoma
		8855/3	Mixed type liposarcoma
		8857/3	Fibroblastic liposarcoma
		8858/3	Dedifferentiated liposarcoma
SYNOVIAL SARCOMA, NOS	904	9040/3	Synovial sarcoma, NOS
		9041/3	Synovial sarcoma, spindle cell
		9042/3	Synovial sarcoma, epithelioid cell
		9043/3	Synovial sarcoma, biphasic
BLOOD VESSEL TUMORS	912	9120/3	Hemangiosarcoma

BONES OF SKULL AND FACE C410

HEMANGIOENDOTHELIOMA	913	9130/3	Hemangioendothelioma, malignant
		9133/3	Epithelioid hemangioendothelioma, malignant
OSTEOSARCOMA, NOS	918	9180/3	Osteosarcoma, NOS
		9181/3	Chondroblastic osteosarcoma
		9182/3	Fibroblastic osteosarcoma
		9183/3	Telangiectatic osteosarcoma
		9184/3	Osteosarcoma in Paget disease
		9185/3	Small cell osteosarcoma
		9186/3	Central osteosarcoma
		9187/3	Instrosseous well differentiated osteosarcoma
JUXTACORTICAL OSTEOSARCOMA	919	9192/3	Parosteal osteosarcoma
		9193/3	Periosteal osteosarcoma
		9194/3	High grade surface osteosarcoma
		9195/3	Intracortical osteosarcoma
CHONDROSARCOMA, NOS	922	9220/3	Chondrosarcoma, NOS
		9221/3	Juxtacortical chondrosarcoma
CHONDROBLASTOMA, MALIGNANT	923	9230/3	Chondroblastoma, malignant
		9231/3	Myxoid chondrosarcoma
OSSEOUS & CHONDROMATOUS NEOPLASMS	924	9240/3	Mesenchymal chondrosarcoma
		9242/3	Clear cell chondrosarcoma
		9243/3	Dedifferentiated chondrosarcoma
GT. CELL TUMOR OF BONE, MAL.	925	9250/3	Giant cell tumor of bone, malignant
		9251/3	Malignant giant cell tumor of soft parts
EWING SARCOMA	926	9260/3	Ewing sarcoma
		9261/3	Adamantinoma of long bones
ODONTOGENIC TUMOR, MAL.	927	9270/3	Odontogenic tumor, malignant
AMELOBLASTIC ODONTOSARCOMA	929	9290/3	Ameloblastic odontosarcoma
ODONTOGENIC TUMOR, MAL.	930	9302/3	Ghost cell odontogenic carcinoma
AMELOBLASTOMA, MALIGNANT	931	9310/3	Ameloblastoma, malignant
AMELOBLASTIC FIBROSARCOMA	933	9330/3	Ameloblastic fibrosarcoma
ODONTOGENIC CARCINOSARCOMA	934	9341/3	Clear cell odontogenic carcinoma
		9342/3	Odontogenic carcinosarcoma

BONES OF SKULL AND FACE C410

PERIPHERAL NEUROECTODERMAL TUMOR	936	9364/3	Peripheral neuroectodermal tumor
		9365/3	Askin tumor
CHORDOMA	937	9370/3	Chordoma, NOS
		9371/3	Chondroid chordoma
		9372/3	Dedifferentiated chordoma
MALIGNANT LYMPHOMA, NOS	959	9590/3	Malignant lymphoma, NOS
		9591/3	Malignant lymphoma, non-Hodgkin
		9596/3	Composite Hodgkin and non-Hodgkin lymphoma
HODGKIN LYMPHOMA	965	9650/3	Hodgkin lymphoma, NOS
		9651/3	Hodgkin lymphoma, lymphocyte-rich
		9652/3	Hodgkin lymphoma, mixed cellularity, NOS
		9653/3	Hodgkin lymphoma, lymphocytic deplet., NOS
		9654/3	Hodgkin lymph., lymphocyt. deplet., diffuse fibrosis
		9655/3	Hodgkin lymphoma, lymphocyt. deplet., reticular
		9659/3	Hodgkin lymph., nodular lymphocyte predom.
HODGKIN LYMPHOMA, NOD. SCLER.	966	9661/3	Hodgkin granuloma [obs]
		9662/3	Hodgkin sarcoma [obs]
		9663/3	Hodgkin lymphoma, nodular sclerosis, NOS
		9664/3	Hodgkin lymphoma, nod. scler., cellular phase
		9665/3	Hodgkin lymphoma, nod. scler., grade 1
		9667/3	Hodgkin lymphoma, nod. scler., grade 2
ML, SMALL B-CELL LYMPHOCYTIC	967	9670/3	ML, small B lymphocytic, NOS
		9671/3	ML, lymphoplasmacytic
		9673/3	Mantle cell lymphoma
		9675/3	ML, mixed sm. and lg. cell, diffuse
ML, LARGE B-CELL, DIFFUSE	968	9680/3	ML, large B-cell, diffuse
		9684/3	ML, large B-cell, diffuse, immunoblastic, NOS
		9687/3	Burkitt lymphoma, NOS
		9688/3	T-cell histiocyte rich large B-cell lymphoma
FOLLIC. & MARGINAL LYMPH, NOS	969	9690/3	Follicular lymphoma, NOS
		9691/3	Follicular lymphoma, grade 2
		9695/3	Follicular lymphoma, grade 1
		9698/3	Follicular lymphoma, grade 3
		9699/3	Marginal zone B-cell lymphoma, NOS
T-CELL LYMPHOMAS	970	9701/3	Sezary syndrome
		9702/3	Mature T-cell lymphoma, NOS
		9705/3	Angioimmunoblastic T-cell lymphoma

BONES OF SKULL AND FACE C410

OTHER SPEC. NON-HODGKIN LYMPHOMA	971	9712/3	Intravascular large B-cell lymphoma
		9714/3	Anaplastic large cell lymphoma, T-cell and Null cell type
		9716/3	Hepatosplenic gamma-delta cell lymphoma
		9719/3	NK/T-cell lymphoma, nasal and nasal-type
PRECURS. CELL LYMPHOBLASTIC LYMPH.	972	9724/3	SystemicEBV pos. T-cell lymphoproliferative disease of childhood
		9727/3	Precursor cell lymphoblastic lymphoma, NOS
		9728/3	Precursor B-cell lymphoblastic lymphoma
		9729/3	Precursor T-cell lymphoblastic lymphoma
PLASMA CELL TUMORS	973	9731/3	Plasmacytoma, NOS
		9732/3	Multiple myeloma
		9735/3	Plasmablastic lymphoma
		9737/3	ALK positive large B-cell lymphoma
		9738/3	Lrg B-cell lymphoma in HHV8-assoc. multicentric Castleman DZ
MAST CELL TUMORS	974	9740/3	Mast cell sarcoma
		9741/3	Malignant mastocytosis
NEOPLASMS OF HISTIOCYTES AND ACCESSORY LYMPHOID CELLS	975	9750/3	Malignant histiocytosis
		9751/3	Langerhans cell histiocytosis, NOS
		9754/3	Langerhans cell histiocytosis, disseminated
		9755/3	Histiocytic sarcoma
		9756/3	Langerhans cell sarcoma
		9757/3	Interdigitating dendritic cell sarcoma
		9758/3	Follicular dendritic cell sarcoma
		9759/3	Fibroblastic reticular cell tumor
PRECURSOR LYMPHOID NEOPLASMS	981	9811/3	B lymphoblastic leukemia/lymphoma, NOS
		9812/3	Leukemia/lymphoma with t(9;22)(q34;q11.2);BCR-ABL1
		9813/3	Leukemia/lymphoma with t(v;11q23);MLL rearranged
		9814/3	Leukemia/lymphoma with t(12;21)(p13;q22);TEL-AML1(ETV6-RUNX1)
		9815/3	B lymphoblastic leukemia/lymphoma with hyperdiploidy
		9816/3	Leukemia/lymphoma with hypodiploidy (hypodiploid ALL)
		9817/3	B lymphblastic leukemia/lymphoma with t(5;14)(q31;q32);IL3-IGH
		9818/3	Leukemia/lymphoma with t(1;19)(q23;p13.3); E2A PBX1 (TCF3 PBX1)
LYMPHOID LEUKEMIA, NOS	982	9823/3	Chronic lymphocytic leukemia/small lymphocytic lymphoma
PROLYMPH/PRECURS LEUKEMIA	983	9831/3	T-cell large granular lymphocytic leukemia
		9837/3	T lymphoblastic leukemia/lymphoma
CHRONIC MYELOPROLIFERATIVE DIS.	996	9965/3	Myeloid and lymphoid neoplasms with PDGFRB rearrangement
		9967/3	Myeloid and lymphoid neoplasm with FGFR1 abnormalities

BONES OF SKULL AND FACE C410

MYELOPLASTIC/MYELOPROLIFERATIVE NEOPLASMS	997	9971/3	Polymorphic PTLD
		9975/3	Myelodysplastic/Myeloproliferative neoplasm, unclassifiable

MANDIBLE C411

NEOPLASM	800	8000/3	Neoplasm, malignant
		8001/3	Tumor cells, malignant
		8002/3	Malignant tumor, small cell type
		8003/3	Malignant tumor, giant cell type
		8004/3	Malignant tumor, spindle cell type
		8005/3	Malignant tumor, clear cell type
SARCOMA, NOS	880	8800/3	Sarcoma, NOS
		8801/3	Spindle cell sarcoma
		8802/3	Giant cell sarcoma
		8803/3	Small cell sarcoma
		8804/3	Epithelioid sarcoma
		8805/3	Undifferentiated sarcoma
		8806/3	Desmoplastic small round cell tumor
FIBROMATOUS NEOPLASMS	881	8810/3	Fibrosarcoma, NOS
		8811/3	Fibromyxosarcoma
		8812/3	Periosteal fibrosarcoma
		8813/3	Fascial fibrosarcoma
		8814/3	Infantile fibrosarcoma
		8815/3	Solitary fibrous tumor, malignant
SARCOMA, NOS	882	8825/3	Myofibroblastic sarcoma
FIBROUS HISTIOCYTOMA, MAL.	883	8830/3	Fibrous histiocytoma, malignant
MYXOSARCOMA	884	8840/3	Myxosarcoma
LIPOSARCOMA NEOPLASMS	885	8850/3	Liposarcoma, NOS
		8851/3	Liposarcoma, well differentiated
		8852/3	Myxoid liposarcoma
		8853/3	Round cell liposarcoma
		8854/3	Pleomorphic liposarcoma
		8855/3	Mixed type liposarcoma
		8857/3	Fibroblastic liposarcoma
		8858/3	Dedifferentiated liposarcoma
SYNOVIAL SARCOMA, NOS	904	9040/3	Synovial sarcoma, NOS
		9041/3	Synovial sarcoma, spindle cell
		9042/3	Synovial sarcoma, epithelioid cell
		9043/3	Synovial sarcoma, biphasic
BLOOD VESSEL TUMORS	912	9120/3	Hemangiosarcoma

MANDIBLE C411

HEMANGIOENDOTHELIOMA	913	9130/3	Hemangioendothelioma, malignant
		9133/3	Epithelioid hemangioendothelioma, malignant
OSTEOSARCOMA, NOS	918	9180/3	Osteosarcoma, NOS
		9181/3	Chondroblastic osteosarcoma
		9182/3	Fibroblastic osteosarcoma
		9183/3	Telangiectatic osteosarcoma
		9184/3	Osteosarcoma in Paget disease
		9185/3	Small cell osteosarcoma
		9186/3	Central osteosarcoma
		9187/3	Instrosseous well differentiated osteosarcoma
JUXTACORTICAL OSTEOSARCOMA	919	9192/3	Parosteal osteosarcoma
		9193/3	Periosteal osteosarcoma
		9194/3	High grade surface osteosarcoma
		9195/3	Intracortical osteosarcoma
CHONDROSARCOMA, NOS	922	9220/3	Chondrosarcoma, NOS
		9221/3	Juxtacortical chondrosarcoma
CHONDROBLASTOMA, MALIGNANT	923	9230/3	Chondroblastoma, malignant
		9231/3	Myxoid chondrosarcoma
OSSEOUS & CHONDROMATOUS NEOPLASMS	924	9240/3	Mesenchymal chondrosarcoma
		9242/3	Clear cell chondrosarcoma
		9243/3	Dedifferentiated chondrosarcoma
GT. CELL TUMOR OF BONE, MAL.	925	9250/3	Giant cell tumor of bone, malignant
		9251/3	Malignant giant cell tumor of soft parts
EWING SARCOMA	926	9260/3	Ewing sarcoma
		9261/3	Adamantinoma of long bones
ODONTOGENIC TUMOR, MAL.	927	9270/3	Odontogenic tumor, malignant
AMELOBLASTIC ODONTOSARCOMA	929	9290/3	Ameloblastic odontosarcoma
ODONTOGENIC TUMOR, MAL.	930	9302/3	Ghost cell odontogenic carcinoma
AMELOBLASTOMA, MALIGNANT	931	9310/3	Ameloblastoma, malignant
AMELOBLASTIC FIBROSARCOMA	933	9330/3	Ameloblastic fibrosarcoma
ODONTOGENIC CARCINOSARCOMA	934	9341/3	Clear cell odontogenic carcinoma
		9342/3	Odontogenic carcinosarcoma

MANDIBLE C411

PERIPHERAL NEUROECTODERMAL TUMOR	936	9364/3	Peripheral neuroectodermal tumor
		9365/3	Askin tumor
CHORDOMA	937	9370/3	Chordoma, NOS
		9371/3	Chondroid chordoma
		9372/3	Dedifferentiated chordoma
MALIGNANT LYMPHOMA, NOS	959	9590/3	Malignant lymphoma, NOS
		9591/3	Malignant lymphoma, non-Hodgkin
		9596/3	Composite Hodgkin and non-Hodgkin lymphoma
HODGKIN LYMPHOMA	965	9650/3	Hodgkin lymphoma, NOS
		9651/3	Hodgkin lymphoma, lymphocyte-rich
		9652/3	Hodgkin lymphoma, mixed cellularity, NOS
		9653/3	Hodgkin lymphoma, lymphocytic deplet., NOS
		9654/3	Hodgkin lymph., lymphocyt. deplet., diffuse fibrosis
		9655/3	Hodgkin lymphoma, lymphocyt. deplet., reticular
		9659/3	Hodgkin lymph., nodular lymphocyte predom.
HODGKIN LYMPHOMA, NOD. SCLER.	966	9661/3	Hodgkin granuloma [obs]
		9662/3	Hodgkin sarcoma [obs]
		9663/3	Hodgkin lymphoma, nodular sclerosis, NOS
		9664/3	Hodgkin lymphoma, nod. scler., cellular phase
		9665/3	Hodgkin lymphoma, nod. scler., grade 1
		9667/3	Hodgkin lymphoma, nod. scler., grade 2
ML, SMALL B-CELL LYMPHOCYTIC	967	9670/3	ML, small B lymphocytic, NOS
		9671/3	ML, lymphoplasmacytic
		9673/3	Mantle cell lymphoma
		9675/3	ML, mixed sm. and lg. cell, diffuse
ML, LARGE B-CELL, DIFFUSE	968	9680/3	ML, large B-cell, diffuse
		9684/3	ML, large B-cell, diffuse, immunoblastic, NOS
		9687/3	Burkitt lymphoma, NOS
		9688/3	T-cell histiocyte rich large B-cell lymphoma
FOLLIC. & MARGINAL LYMPH, NOS	969	9690/3	Follicular lymphoma, NOS
		9691/3	Follicular lymphoma, grade 2
		9695/3	Follicular lymphoma, grade 1
		9698/3	Follicular lymphoma, grade 3
		9699/3	Marginal zone B-cell lymphoma, NOS
T-CELL LYMPHOMAS	970	9701/3	Sezary syndrome
		9702/3	Mature T-cell lymphoma, NOS
		9705/3	Angioimmunoblastic T-cell lymphoma

MANDIBLE C411

OTHER SPEC. NON-HODGKIN LYMPHOMA	971	9712/3	Intravascular large B-cell lymphoma
		9714/3	Anaplastic large cell lymphoma, T-cell and Null cell type
		9716/3	Hepatosplenic gamma-delta cell lymphoma
		9719/3	NK/T-cell lymphoma, nasal and nasal-type
PRECURS. CELL LYMPHOBLASTIC LYMPH.	972	9724/3	SystemicEBV pos. T-cell lymphoproliferative disease of childhood
		9727/3	Precursor cell lymphoblastic lymphoma, NOS
		9728/3	Precursor B-cell lymphoblastic lymphoma
		9729/3	Precursor T-cell lymphoblastic lymphoma
PLASMA CELL TUMORS	973	9731/3	Plasmacytoma, NOS
		9732/3	Multiple myeloma
		9735/3	Plasmablastic lymphoma
		9737/3	ALK positive large B-cell lymphoma
		9738/3	Lrg B-cell lymphoma in HHV8-assoc. multicentric Castleman DZ
MAST CELL TUMORS	974	9740/3	Mast cell sarcoma
		9741/3	Malignant mastocytosis
NEOPLASMS OF HISTIOCYTES AND ACCESSORY LYMPHOID CELLS	975	9750/3	Malignant histiocytosis
		9751/3	Langerhans cell histiocytosis, NOS
		9754/3	Langerhans cell histiocytosis, disseminated
		9755/3	Histiocytic sarcoma
		9756/3	Langerhans cell sarcoma
		9757/3	Interdigitating dendritic cell sarcoma
		9758/3	Follicular dendritic cell sarcoma
		9759/3	Fibroblastic reticular cell tumor
PRECURSOR LYMPHOID NEOPLASMS	981	9811/3	B lymphoblastic leukemia/lymphoma, NOS
		9812/3	Leukemia/lymphoma with t(9;22)(q34;q11.2);BCR-ABL1
		9813/3	Leukemia/lymphoma with t(v;11q23);MLL rearranged
		9814/3	Leukemia/lymphoma with t(12;21)(p13;q22);TEL-AML1(ETV6-RUNX1)
		9815/3	B lymphoblastic leukemia/lymphoma with hyperdiploidy
		9816/3	Leukemia/lymphoma with hypodiploidy (hypodiploid ALL)
		9817/3	B lymphblastic leukemia/lymphoma with t(5;14)(q31;q32);IL3-IGH
		9818/3	Leukemia/lymphoma with t(1;19)(q23;p13.3); E2A PBX1 (TCF3 PBX1)
LYMPHOID LEUKEMIA, NOS	982	9823/3	Chronic lymphocytic leukemia/small lymphocytic lymphoma
PROLYMPH/PRECURS LEUKEMIA	983	9831/3	T-cell large granular lymphocytic leukemia
		9837/3	T lymphoblastic leukemia/lymphoma
CHRONIC MYELOPROLIFERATIVE DIS.	996	9965/3	Myeloid and lymphoid neoplasms with PDGFRB rearrangement
		9967/3	Myeloid and lymphoid neoplasm with FGFR1 abnormalities

MANDIBLE C411

MYELOPLASTIC/MYELOPROLIFERATIVE NEOPLASMS	997	9971/3	Polymorphic PTLD
		9975/3	Myelodysplastic/Myeloproliferative neoplasm, unclassifiable

BLOOD, BONE MARROW, & HEMATOPOIETICSYS C420, C421, C424

MALIGNANT LYMPHOMA, NOS	959	9590/3	Malignant lymphoma, NOS
		9591/3	Malignant lymphoma, non-Hodgkin
		9596/3	Composite Hodgkin and non-Hodgkin lymphoma
HODGKIN LYMPHOMA	965	9650/3	Hodgkin lymphoma, NOS
		9651/3	Hodgkin lymphoma, lymphocyte-rich
		9652/3	Hodgkin lymphoma, mixed cellularity, NOS
		9653/3	Hodgkin lymphoma, lymphocytic deplet., NOS
		9654/3	Hodgkin lymph., lymphocyt. deplet., diffuse fibrosis
		9655/3	Hodgkin lymphoma, lymphocyt. deplet., reticular
		9659/3	Hodgkin lymph., nodular lymphocyte predom.
HODGKIN LYMPHOMA, NOD. SCLER.	966	9661/3	Hodgkin granuloma [obs]
		9662/3	Hodgkin sarcoma [obs]
		9663/3	Hodgkin lymphoma, nodular sclerosis, NOS
		9664/3	Hodgkin lymphoma, nod. scler., cellular phase
		9665/3	Hodgkin lymphoma, nod. scler., grade 1
		9667/3	Hodgkin lymphoma, nod. scler., grade 2
ML, SMALL B-CELL LYMPHOCYTIC	967	9670/3	ML, small B lymphocytic, NOS
		9671/3	ML, lymphoplasmacytic
		9673/3	Mantle cell lymphoma
		9675/3	ML, mixed sm. and lg. cell, diffuse
		9678/3	Primary effusion lymphoma
		9679/3	Mediastinal large B-cell lymphoma
ML, LARGE B-CELL, DIFFUSE	968	9680/3	ML, large B-cell, diffuse
		9684/3	ML, large B-cell, diffuse, immunoblastic, NOS
		9687/3	Burkitt lymphoma, NOS
		9688/3	T-cell histiocyte rich large B-cell lymphoma
		9689/3	Splenic marginal zone B-cell lymphoma
FOLLIC. & MARGINAL LYMPH, NOS	969	9690/3	Follicular lymphoma, NOS
		9691/3	Follicular lymphoma, grade 2
		9695/3	Follicular lymphoma, grade 1
		9698/3	Follicular lymphoma, grade 3
		9699/3	Marginal zone B-cell lymphoma, NOS
T-CELL LYMPHOMAS	970	9701/3	Sezary syndrome
		9702/3	Mature T-cell lymphoma, NOS
		9705/3	Angioimmunoblastic T-cell lymphoma
OTHER SPEC. NON-HODGKIN LYMPHOMA	971	9712/3	Intravascular large B-cell lymphoma
		9714/3	Anaplastic large cell lymphoma, T-cell and Null cell type
		9719/3	NK/T-cell lymphoma, nasal and nasal-type

BLOOD, BONE MARROW, & HEMATOPOIETICSYS C420, C421, C424

PRECURS. CELL LYMPHOBLASTIC LYMPH.	972	9724/3	SystemicEBV pos. T-cell lymphoproliferative disease of childhood
		9727/3	Precursor cell lymphoblastic lymphoma, NOS
		9728/3	Precursor B-cell lymphoblastic lymphoma
		9729/3	Precursor T-cell lymphoblastic lymphoma
PLASMA CELL TUMORS	973	9731/3	Plasmacytoma, NOS
		9732/3	Multiple myeloma
		9733/3	Plasma cell leukemia
		9734/3	Plasmacytoma, extramedullary
		9735/3	Plasmablastic lymphoma
		9737/3	ALK positive large B-cell lymphoma
		9738/3	Lrg B-cell lymphoma in HHV8-assoc. multicentric Castleman DZ
MAST CELL TUMORS	974	9740/3	Mast cell sarcoma
		9741/3	Malignant mastocytosis
		9742/3	Mast cell leukemia
NEOPLASMS OF HISTIOCYTES AND ACCESSORY LYMPHOID CELLS	975	9750/3	Malignant histiocytosis
		9751/3	Langerhans cell histiocytosis, NOS
		9754/3	Langerhans cell histiocytosis, disseminated
		9755/3	Histiocytic sarcoma
		9756/3	Langerhans cell sarcoma
		9757/3	Interdigitating dendritic cell sarcoma
		9758/3	Follicular dendritic cell sarcoma
		9759/3	Fibroblastic reticular cell tumor
IMMUNOPROLIFERATIVE DISEASES	976	9760/3	Immunoproliferative disease, NOS
		9761/3	Waldenstrom macroglobulinemia
		9762/3	Heavy chain disease, NOS
LEUKEMIA, NOS	980	9800/3	Leukemia, NOS
		9801/3	Acute leukemia, NOS
		9805/3	Acute biphenotypic leukemia
		9806/3	Mixed phenotype acute leukemia with t(9;22)(q34;q11.2);BCR-ABL1
		9807/3	Mixed phenotype acute leukemia with t(v;11q23);MLL rearranged
		9808/3	Mixed phenotype acute leukemia, B/myeloid, NOS
		9809/3	Mixed phenotype acute leukemia, T/myeloid, NOS
PRECURSOR LYMPHOID NEOPLASMS	981	9811/3	B lymphoblastic leukemia/lymphoma, NOS
		9812/3	Leukemia/lymphoma with t(9;22)(q34;q11.2);BCR-ABL1
		9813/3	Leukemia/lymphoma with t(v;11q23);MLL rearranged
		9814/3	Leukemia/lymphoma with t(12;21)(p13;q22);TEL-AML1(ETV6-RUNX1)
		9815/3	B lymphoblastic leukemia/lymphoma with hyperdiploidy
		9816/3	Leukemia/lymphoma with hypodiploidy (hypodiploid ALL)
		9817/3	B lymphblastic leukemia/lymphoma with t(5;14)(q31;q32);IL3-IGH
		9818/3	Leukemia/lymphoma with t(1;19)(q23;p13.3); E2A PBX1 (TCF3 PBX1)

BLOOD, BONE MARROW, & HEMATOPOIETIC SYS C420, C421, C424

LYMPHOID LEUKEMIA, NOS	982	9820/3	Lymphoid leukemia, NOS
		9823/3	Chronic lymphocytic leukemia/small lymphocytic lymphoma
		9826/3	Burkitt cell leukemia
		9827/3	Adult T-cell leukemia/lymphoma (HTLV-1 pos.)
		9828/3	Acute lymphoblastic leukemia, L2 type, NOS
PROLYMPH/PRECURS LEUKEMIA	983	9831/3	T-cell large granular lymphocytic leukemia
		9832/3	Prolymphocytic leukemia, NOS
		9833/3	Prolymphocytic leukemia, B-cell type
		9834/3	Prolymphocytic leukemia, T-cell type
		9835/3	Precursor cell lymphoblastic leukemia, NOS
		9836/3	Precursor B-cell lymphoblastic leukemia
		9837/3	T lymphoblastic leukemia/lymphoma
ERYTHROID LEUKEMIA	984	9840/3	Acute myeloid leukemia, M6 type
MYELOID LEUKEMIA, NOS	986	9860/3	Myeloid leukemia, NOS
		9861/3	Acute myeloid leukemia
		9863/3	Chronic myeloid leukemia, NOS
		9865/3	Acute myeloid leukemia with t(6;9)(p23;q34) DEK-NUP214
		9866/3	Acute promyelocytic leuk.,t(15;17)(q22;q11-12)
		9867/3	Acute myelomonocytic leukemia
		9869/3	Acute myeloid leukemia with inv(3)(q21q26.2) or t(3;3)(q21;q26.2);RPN1EVI1
OTHER MYELOID LEUKEMIAS	987	9870/3	Acute basophilic leukemia
		9871/3	Ac. myelomonocytic leuk. w abn. mar. eosinophils
		9872/3	Acute myeloid leukemia, minimal differentiation
		9873/3	Acute myeloid leukemia without maturation
		9874/3	Acute myeloid leukemia with maturation
		9875/3	Chronic myelogenous leukemia, BCR/ABL positive
		9876/3	Atypical chronic myeloid leuk., BCR/ABL negative
MONOCYTIC/OTHER LEUKEMIA, NOS	989	9891/3	Acute monocytic leukemia
		9895/3	Acute myeloid leuk. with multilineage dysplasia
		9896/3	Acute myeloid leukemia, t(8;21)(q22;q22)
		9897/3	Acute myeloid leukemia, 11q23 abnormalities
		9898/3	Myeloid leukemia associated with Down Syndrome
MEGAKARYOBLASTIC LEUKEMIA	991	9910/3	Acute megakaryoblastic leukemia
		9911/3	Acute myeloid leukemia (megakaryoblastic) with t(1;22)(p13;q13);RBM15-MLK1
THERAPY RELATED AC. MYEL. LEUK.	992	9920/3	Therapy-related acute myeloid leukemia, NOS

BLOOD, BONE MARROW, & HEMATOPOIETIC SYS C420, C421, C424

MYELOID SARCOMA	993	9930/3	Myeloid sarcoma
		9931/3	Acute panmyelosis with myelofibrosis
OTHER LEUKEMIAS	994	9940/3	Hairy cell leukemia
		9945/3	Chronic myelomonocytic leukemia, NOS
		9946/3	Juvenile myelomonocytic leukemia
		9948/3	Aggressive NK-cell leukemia
POLYCYTHEMIA VERA	995	9950/3	Polycythemia vera
CHRONIC MYELOPROLIFERATIVE DIS.	996	9960/3	Chronic myeloproliferative disease, NOS
		9961/3	Myelosclerosis with myeloid metaplasia
		9962/3	Essential thrombocythemia
		9963/3	Chronic neutrophilic leukemia
		9964/3	Hypereosinophilic syndrome
		9965/3	Myeloid and lymphoid neoplasms with PDGFRB rearrangement
		9966/3	Myeloid and lymphoid neoplasms with PDGFRB re arrangement
		9967/3	Myeloid and lymphoid neoplasm with FGFR1 abnormalities
MYELOPLASTIC/MYELOPROLIFERATIVE NEOPLASMS	997	9971/3	Polymorphic PTLD
		9975/3	Myelodysplastic/Myeloproliferative neoplasm, unclassifiable
REFRACTORY ANEMIA	998	9980/3	Refractory anemia
		9982/3	Refractory anemia with sideroblasts
		9983/3	Refractory anemia with excess blasts
		9984/3	Refract. anemia with excess blasts in transformation
		9985/3	Refractory cytopenia with multilineage dysplasia
		9986/3	Myelodysplastic syndr. with 5q deletion syndrome
		9987/3	Therapy-related myelodysplastic syndrome, NOS
		9989/3	Myelodysplastic syndrome, NOS
MYELODYSPLASTIC SYNDROMES	999	9991/3	Refractory neutropenia
		9992/3	Refractory thrombocytopenia

SPLEEN C422

BLOOD VESSEL TUMORS	912	9120/3	Hemangiosarcoma
MALIGNANT LYMPHOMA, NOS	959	9590/3	Malignant lymphoma, NOS
		9591/3	Malignant lymphoma, non-Hodgkin
		9596/3	Composite Hodgkin and non-Hodgkin lymphoma
HODGKIN LYMPHOMA	965	9650/3	Hodgkin lymphoma, NOS
		9651/3	Hodgkin lymphoma, lymphocyte-rich
		9652/3	Hodgkin lymphoma, mixed cellularity, NOS
		9653/3	Hodgkin lymphoma, lymphocytic deplet., NOS
		9654/3	Hodgkin lymph., lymphocyt. deplet., diffuse fibrosis
		9655/3	Hodgkin lymphoma, lymphocyt. deplet., reticular
		9659/3	Hodgkin lymph., nodular lymphocyte predom.
HODGKIN LYMPHOMA, NOD. SCLER.	966	9661/3	Hodgkin granuloma [obs]
		9662/3	Hodgkin sarcoma [obs]
		9663/3	Hodgkin lymphoma, nodular sclerosis, NOS
		9664/3	Hodgkin lymphoma, nod. scler., cellular phase
		9665/3	Hodgkin lymphoma, nod. scler., grade 1
		9667/3	Hodgkin lymphoma, nod. scler., grade 2
ML, SMALL B-CELL LYMPHOCYTIC	967	9670/3	ML, small B lymphocytic, NOS
		9671/3	ML, lymphoplasmacytic
		9673/3	Mantle cell lymphoma
		9675/3	ML, mixed sm. and lg. cell, diffuse
		9678/3	Primary effusion lymphoma
		9679/3	Mediastinal large B-cell lymphoma
ML, LARGE B-CELL, DIFFUSE	968	9680/3	ML, large B-cell, diffuse
		9684/3	ML, large B-cell, diffuse, immunoblastic, NOS
		9687/3	Burkitt lymphoma, NOS
		9688/3	T-cell histiocyte rich large B-cell lymphoma
		9689/3	Splenic marginal zone B-cell lymphoma
FOLLIC. & MARGINAL LYMPH, NOS	969	9690/3	Follicular lymphoma, NOS
		9691/3	Follicular lymphoma, grade 2
		9695/3	Follicular lymphoma, grade 1
		9698/3	Follicular lymphoma, grade 3
		9699/3	Marginal zone B-cell lymphoma, NOS
T-CELL LYMPHOMAS	970	9701/3	Sezary syndrome
		9702/3	Mature T-cell lymphoma, NOS
		9705/3	Angioimmunoblastic T-cell lymphoma

SPLEEN C422

OTHER SPEC. NON-HODGKIN LYMPHOMA	971	9712/3	Intravascular large B-cell lymphoma
		9714/3	Anaplastic large cell lymphoma, T-cell and Null cell type
		9716/3	Hepatosplenic gamma-delta cell lymphoma
		9719/3	NK/T-cell lymphoma, nasal and nasal-type
PRECURS. CELL LYMPHOBLASTIC LYMPH.	972	9724/3	SystemicEBV pos. T-cell lymphoproliferative disease of childhood
		9727/3	Precursor cell lymphoblastic lymphoma, NOS
		9728/3	Precursor B-cell lymphoblastic lymphoma
		9729/3	Precursor T-cell lymphoblastic lymphoma
PLASMA CELL TUMORS	973	9731/3	Plasmacytoma, NOS
		9734/3	Plasmacytoma, extramedullary
		9735/3	Plasmablastic lymphoma
		9737/3	ALK positive large B-cell lymphoma
		9738/3	Lrg B-cell lymphoma in HHV8-assoc. multicentric Castleman DZ
MAST CELL TUMORS	974	9740/3	Mast cell sarcoma
		9741/3	Malignant mastocytosis
NEOPLASMS OF HISTIOCYTES AND ACCESSORY LYMPHOID CELLS	975	9750/3	Malignant histiocytosis
		9751/3	Langerhans cell histiocytosis, NOS
		9754/3	Langerhans cell histiocytosis, disseminated
		9755/3	Histiocytic sarcoma
		9756/3	Langerhans cell sarcoma
		9757/3	Interdigitating dendritic cell sarcoma
		9758/3	Follicular dendritic cell sarcoma
		9759/3	Fibroblastic reticular cell tumor
PRECURSOR LYMPHOID NEOPLASMS	981	9811/3	B lymphoblastic leukemia/lymphoma, NOS
		9812/3	Leukemia/lymphoma with t(9;22)(q34;q11.2);BCR-ABL1
		9813/3	Leukemia/lymphoma with t(v;11q23);MLL rearranged
		9814/3	Leukemia/lymphoma with t(12;21)(p13;q22);TEL-AML1(ETV6-RUNX1)
		9815/3	B lymphoblastic leukemia/lymphoma with hyperdiploidy
		9816/3	Leukemia/lymphoma with hypodiploidy (hypodiploid ALL)
		9817/3	B lymphblastic leukemia/lymphoma with t(5;14)(q31;q32);IL3-IGH
		9818/3	Leukemia/lymphoma with t(1;19)(q23;p13.3); E2A PBX1 (TCF3 PBX1)
LYMPHOID LEUKEMIA, NOS	982	9823/3	Chronic lymphocytic leukemia/small lymphocytic lymphoma
PROLYMPH/PRECURS LEUKEMIA	983	9831/3	T-cell large granular lymphocytic leukemia
		9837/3	T lymphoblastic leukemia/lymphoma
CHRONIC MYELOPROLIFERATIVE DIS.	996	9965/3	Myeloid and lymphoid neoplasms with PDGFRB rearrangement
		9967/3	Myeloid and lymphoid neoplasm with FGFR1 abnormalities

SPLEEN C422

MYELOPLASTIC/MYELOPROLIFERATIVE NEOPLASMS	997	9971/3	Polymorphic PTLD
		9975/3	Myelodysplastic/Myeloproliferative neoplasm, unclassifiable

RETICULO-ENDOTHELIAL C423

MALIGNANT LYMPHOMA, NOS	959	9590/3	Malignant lymphoma, NOS
FOLLIC. & MARGINAL LYMPH, NOS	969	9699/3	Marginal zone B-cell lymphoma, NOS
MAST CELL TUMORS	974	9740/3	Mast cell sarcoma
		9741/3	Malignant mastocytosis
NEOPLASMS OF HISTIOCYTES AND ACCESSORY LYMPHOID CELLS	975	9750/3	Malignant histiocytosis
		9754/3	Langerhans cell histiocytosis, disseminated
		9755/3	Histiocytic sarcoma
		9756/3	Langerhans cell sarcoma
		9757/3	Interdigitating dendritic cell sarcoma
		9758/3	Follicular dendritic cell sarcoma
LYMPHOID LEUKEMIA, NOS	982	9823/3	Chronic lymphocytic leukemia/small lymphocytic lymphoma

SKIN C440-C449

TRICHILEMMOCARCINOMA	810	8102/3	Trichilemmocarcinoma
PILOMATRIX CARCINOMA	811	8110/3	Pilomatrix carcinoma
ADENOCARCINOMA, NOS	814	8140/2	Adenocarcinoma in situ
		8140/3	Adenocarcinoma, NOS
		8141/3	Scirrhous adenocarcinoma
		8143/3	Superficial spreading adenocarcinoma
		8147/3	Basal cell adenocarcinoma
TRABECULAR ADENOCARCINOMA	819	8190/3	Trabecular adenocarcinoma
ADENOID CYSTIC & CRIBRIFORM CA.	820	8200/3	Adenoid cystic carcinoma
		8201/2	Cribriform carcinoma in situ
		8201/3	Cribriform carcinoma
SOLID CARCINOMA, NOS	823	8230/2	Duct carcinoma in situ, solid type
		8230/3	Solid carcinoma, NOS
		8231/3	Carcinoma simplex
CARCINOID TUMOR, MALIGNANT	824	8247/3	Merkel cell carcinoma
BRONCHIOLO-ALVEOLAR ADENOCA.	825	8255/3	Adenocarcinoma with mixed subtypes
PAPILLARY ADENOCARCINOMA, NOS	826	8260/3	Papillary adenocarcinoma, NOS
		8261/2	Adenocarcinoma in situ in villous adenoma
		8261/3	Adenocarcinoma in villous adenoma
		8262/3	Villous adenocarcinoma
		8263/2	Adenocarcinoma in situ in tubulovillous adenoma
		8263/3	Adenocarcinoma in tubulovillous adenoma
CLEAR CELL ADENOCARCINOMA, NOS	831	8310/3	Clear cell adenocarcinoma, NOS
SKIN APPENDAGE CARCINOMA	839	8390/3	Skin appendage carcinoma
SWEAT GLAND ADENOCARCINOMA	840	8400/3	Sweat gland adenocarcinoma
		8401/3	Apocrine adenocarcinoma
		8402/3	Nodular hidradenoma, malignant
		8403/3	Malignant eccrine spiradenoma
		8407/3	Sclerosing sweat duct carcinoma
		8408/3	Eccrine papillary adenocarcinoma
		8409/3	Eccrine poroma, malignant

SKIN C440-C449

SEBACEOUS/ECCRINE ADENOCA.	841	8410/3	Sebaceous adenocarcinoma
		8413/3	Eccrine adenocarcinoma
CERUMINOUS ADENOCARCINOMA	842	8420/3	Ceruminous adenocarcinoma
MUCOEPIDERMOID CARCINOMA	843	8430/3	Mucoepidermoid carcinoma
CYSTADENOCARCINOMA, NOS	844	8440/3	Cystadenocarcinoma, NOS
MUCINOUS ADENOCARCINOMA	848	8480/3	Mucinous adenocarcinoma
		8481/3	Mucin-producing adenocarcinoma
PAGET DISEASE, EXTRAMAMMARY	854	8542/3	Paget disease, extramammary
ADENOSQUAMOUS CARCINOMA	856	8560/3	Adenosquamous carcinoma
		8562/3	Epithelial-myoepithelial carcinoma
ADENOCA. WITH METAPLASIA	857	8570/3	Adenocarcinoma with squamous metaplasia
		8571/3	Adenocarcinoma w cartilag. & oss. metaplas.
		8572/3	Adenocarcinoma with spindle cell mataplasia
		8573/3	Adenocarcinoma with apocrine metaplasia
		8574/3	Adenocarcinoma with neuroendocrine differen.
		8575/3	Metaplastic carcinoma, NOS
NEVI & MELANOMAS	872	8720/2	Melanoma in situ
		8720/3	Malignant melanoma, NOS
		8721/3	Nodular melanoma
		8722/3	Balloon cell melanoma
		8723/3	Malignant melanoma, regressing
AMELANOTIC MELANOMA	873	8730/3	Amelanotic melanoma
MAL. MEL. IN JUNCT. NEVUS	874	8740/3	Mal. melanoma in junctional nevus
		8741/2	Precancerous melanosis, NOS
		8741/3	Mal. melanoma in precan. melanosis
		8742/2	Lentigo maligna
		8742/3	Lentigo maligna melanoma
		8743/2	Superficial spreading melanoma, in situ
		8743/3	Superficial spreading melanoma
		8744/3	Acral lentiginous melanoma, malig.
		8745/3	Desmoplastic melanoma, malignant
		8746/3	Mucosal lentiginous melanoma
MAL. MELAN. IN GIANT PIGMT. NEVUS	876	8761/3	Mal. melanoma in giant pigmented nevus

SKIN C440-C449

EPITHELIOID CELL MELANOMA	877	8770/3	Mixed epithel. & spindle cell melanoma
		8771/3	Epithelioid cell melanoma
		8772/3	Spindle cell melanoma, NOS
BLUE NEVUS, MALIGNANT	878	8780/3	Blue nevus, malignant
SARCOMA, NOS	880	8800/3	Sarcoma, NOS
		8801/3	Spindle cell sarcoma
		8802/3	Giant cell sarcoma
		8803/3	Small cell sarcoma
		8804/3	Epithelioid sarcoma
		8805/3	Undifferentiated sarcoma
		8806/3	Desmoplastic small round cell tumor
FIBROMATOUS NEOPLASMS	881	8810/3	Fibrosarcoma, NOS
		8811/3	Fibromyxosarcoma
		8813/3	Fascial fibrosarcoma
		8814/3	Infantile fibrosarcoma
		8815/3	Solitary fibrous tumor, malignant
SARCOMA, NOS	882	8825/3	Myofibroblastic sarcoma
FIBROUS HISTIOCYTOMA, MAL.	883	8830/3	Fibrous histiocytoma, malignant
		8832/3	Dermatofibrosarcoma, NOS
		8833/3	Pigmented dermatofibrosarcoma protuberans
LIPOSARCOMA NEOPLASMS	885	8850/3	Liposarcoma, NOS
		8851/3	Liposarcoma, well differentiated
		8852/3	Myxoid liposarcoma
		8853/3	Round cell liposarcoma
		8854/3	Pleomorphic liposarcoma
		8855/3	Mixed type liposarcoma
		8857/3	Fibroblastic liposarcoma
		8858/3	Dedifferentiated liposarcoma
MYOMATOUS NEOPLASMS	889	8890/3	Leiomyosarcoma, NOS
		8891/3	Epithelioid leiomyosarcoma
		8894/3	Angiomyosarcoma
		8895/3	Myosarcoma
		8896/3	Myxoid leiomyosarcoma
EMBRYONAL RHABDOMYOSARCOMA	891	8910/3	Embryonal rhabdomyosarcoma
		8912/3	Spindle cell rhabdomyosarcoma

SKIN C440-C449

MIXED TUMOR, MALIGNANT, NOS	894	8940/3	Mixed tumor, malignant, NOS
		8941/3	Carcinoma in pleomorphic adenoma
CARCINOSARCOMA, NOS	898	8980/3	Carcinosarcoma, NOS
		8981/3	Carcinosarcoma, embryonal type
		8982/3	Malignant myoepithelioma
BLOOD VESSEL TUMORS	912	9120/3	Hemangiosarcoma
HEMANGIOENDOTHELIOMA	913	9130/3	Hemangioendothelioma, malignant
		9133/3	Epithelioid hemangioendothelioma, malignant
KAPOSI SARCOMA	914	9140/3	Kaposi sarcoma
HEMANGIOPERICYTOMA	915	9150/3	Hemangiopericytoma, malignant
NEURILEMMOMA	956	9560/3	Neurilemmoma, malignant
		9561/3	MPNST with rhabdomyoblastic differentiation
MALIGNANT LYMPHOMA, NOS	959	9590/3	Malignant lymphoma, NOS
		9591/3	Malignant lymphoma, non-Hodgkin
		9596/3	Composite Hodgkin and non-Hodgkin lymphoma
		9597/3	Primary Cutaneous follicle centre lymphoma
HODGKIN LYMPHOMA	965	9650/3	Hodgkin lymphoma, NOS
		9651/3	Hodgkin lymphoma, lymphocyte-rich
		9652/3	Hodgkin lymphoma, mixed cellularity, NOS
		9653/3	Hodgkin lymphoma, lymphocytic deplet., NOS
		9654/3	Hodgkin lymph., lymphocyt. deplet., diffuse fibrosis
		9655/3	Hodgkin lymphoma, lymphocyt. deplet., reticular
		9659/3	Hodgkin lymph., nodular lymphocyte predom.
HODGKIN LYMPHOMA, NOD. SCLER.	966	9661/3	Hodgkin granuloma [obs]
		9662/3	Hodgkin sarcoma [obs]
		9663/3	Hodgkin lymphoma, nodular sclerosis, NOS
		9664/3	Hodgkin lymphoma, nod. scler., cellular phase
		9665/3	Hodgkin lymphoma, nod. scler., grade 1
		9667/3	Hodgkin lymphoma, nod. scler., grade 2
ML, SMALL B-CELL LYMPHOCYTIC	967	9670/3	ML, small B lymphocytic, NOS
		9671/3	ML, lymphoplasmacytic
		9673/3	Mantle cell lymphoma
		9675/3	ML, mixed sm. and lg. cell, diffuse

SKIN C440-C449

ML, LARGE B-CELL, DIFFUSE	968	9680/3	ML, large B-cell, diffuse
		9684/3	ML, large B-cell, diffuse, immunoblastic, NOS
		9687/3	Burkitt lymphoma, NOS
FOLLIC. & MARGINAL LYMPH, NOS	969	9690/3	Follicular lymphoma, NOS
		9691/3	Follicular lymphoma, grade 2
		9695/3	Follicular lymphoma, grade 1
		9698/3	Follicular lymphoma, grade 3
		9699/3	Marginal zone B-cell lymphoma, NOS
T-CELL LYMPHOMAS	970	9700/3	Mycosis fungoides
		9701/3	Sezary syndrome
		9702/3	Mature T-cell lymphoma, NOS
		9705/3	Angioimmunoblastic T-cell lymphoma
		9708/3	Subcutaneous panniculitis-like T-cell lymphoma
		9709/3	Cutaneous T-cell lymphoma, NOS
OTHER SPEC. NON-HODGKIN LYMPHOMA	971	9718/3	Primary cutan. CD30+ T-cell lymphoprolif. disorder
PRECURS. CELL LYMPHOBLASTIC LYMPH.	972	9725/3	Hydroa vacciniforme-like lymphoma
		9726/3	Primary Cutaneous gamma-delta T-cell lymphoma
		9727/3	Precursor cell lymphoblastic lymphoma, NOS
		9728/3	Precursor B-cell lymphoblastic lymphoma
		9729/3	Precursor T-cell lymphoblastic lymphoma
NEOPLASMS OF HISTIOCYTES AND ACCESSORY LYMPHOID CELLS	975	9751/3	Langerhans cell histiocytosis, NOS
LYMPHOID LEUKEMIA, NOS	982	9823/3	Chronic lymphocytic leukemia/small lymphocytic lymphoma
PROLYMPH/PRECURS LEUKEMIA	983	9837/3	T lymphoblastic leukemia/lymphoma

PERIPHERAL NERVES C470-C476,C478-C479

NEOPLASM	800	8000/3	Neoplasm, malignant
		8001/3	Tumor cells, malignant
		8002/3	Malignant tumor, small cell type
		8003/3	Malignant tumor, giant cell type
		8004/3	Malignant tumor, spindle cell type
		8005/3	Malignant tumor, clear cell type
PARAGANGLIOMA	868	8680/3	Paraganglioma, malignant
EXTRA-ADRENAL PARAGANG., MAL	869	8693/3	Extra-adrenal paraganglioma, malignant
SARCOMA, NOS	880	8800/3	Sarcoma, NOS
		8801/3	Spindle cell sarcoma
		8802/3	Giant cell sarcoma
		8803/3	Small cell sarcoma
		8804/3	Epithelioid sarcoma
		8805/3	Undifferentiated sarcoma
		8806/3	Desmoplastic small round cell tumor
FIBROMATOUS NEOPLASMS	881	8810/3	Fibrosarcoma, NOS
		8811/3	Fibromyxosarcoma
		8813/3	Fascial fibrosarcoma
		8814/3	Infantile fibrosarcoma
		8815/3	Solitary fibrous tumor, malignant
SARCOMA, NOS	882	8825/3	Myofibroblastic sarcoma
FIBROUS HISTIOCYTOMA, MAL.	883	8830/3	Fibrous histiocytoma, malignant
		8832/3	Dermatofibrosarcoma, NOS
		8833/3	Pigmented dermatofibrosarcoma protuberans
MYXOSARCOMA	884	8840/3	Myxosarcoma
LIPOSARCOMA NEOPLASMS	885	8850/3	Liposarcoma, NOS
		8851/3	Liposarcoma, well differentiated
		8852/3	Myxoid liposarcoma
		8853/3	Round cell liposarcoma
		8854/3	Pleomorphic liposarcoma
		8855/3	Mixed type liposarcoma
		8857/3	Fibroblastic liposarcoma
		8858/3	Dedifferentiated liposarcoma
MYOMATOUS NEOPLASMS	889	8890/3	Leiomyosarcoma, NOS
		8891/3	Epithelioid leiomyosarcoma
		8894/3	Angiomyosarcoma
		8895/3	Myosarcoma
		8896/3	Myxoid leiomyosarcoma

PERIPHERAL NERVES C470-C476,C478-C479

RHABDOMYOSARCOMA, NOS	890	8900/3	Rhabdomyosarcoma, NOS
		8901/3	Pleomorphic rhabdomyosarcoma, adult type
		8902/3	Mixed type rhabdomyosarcoma
EMBRYONAL RHABDOMYOSARCOMA	891	8910/3	Embryonal rhabdomyosarcoma
		8912/3	Spindle cell rhabdomyosarcoma
ALVEOLAR RHABDOMYOSARCOMA	892	8920/3	Alveolar rhabdomyosarcoma
		8921/3	Rhabdomyosarcoma with ganglionic differentiation
MULLERIAN MIXED TUMOR	895	8950/3	Mullerian mixed tumor
		8951/3	Mesodermal mixed tumor
MESENCHYMOMA, MALIGNANT	899	8990/3	Mesenchymoma, malignant
		8991/3	Embryonal sarcoma
SYNOVIAL SARCOMA, NOS	904	9040/3	Synovial sarcoma, NOS
		9041/3	Synovial sarcoma, spindle cell
		9042/3	Synovial sarcoma, epithelioid cell
		9043/3	Synovial sarcoma, biphasic
		9044/3	Clear cell sarcoma,NOS (except of kidney M-8964/3)
TERATOMA	908	9080/3	Teratoma, malignant, NOS
		9081/3	Teratocarcinoma
		9082/3	Malignant teratoma, undiff.
		9083/3	Malignant teratoma, intermediate
		9084/3	Teratoma with malig. transformation
		9085/3	Mixed germ cell tumor
BLOOD VESSEL TUMORS	912	9120/3	Hemangiosarcoma
HEMANGIOENDOTHELIOMA	913	9130/3	Hemangioendothelioma, malignant
		9133/3	Epithelioid hemangioendothelioma, malignant
KAPOSI SARCOMA	914	9140/3	Kaposi sarcoma
HEMANGIOPERICYTOMA	915	9150/3	Hemangiopericytoma, malignant
LYMPHANGIOSARCOMA	917	9170/3	Lymphangiosarcoma
OSSEOUS & CHONDROMATOUS NEOPLASMS	924	9240/3	Mesenchymal chondrosarcoma
		9242/3	Clear cell chondrosarcoma
		9243/3	Dedifferentiated chondrosarcoma

PERIPHERAL NERVES C470-C476,C478-C479

GT. CELL TUMOR OF BONE, MAL.	925	9251/3	Malignant giant cell tumor of soft parts
		9252/3	Malignant tenosynovial giant cell tumor
PERIPHERAL NEUROECTODERMAL TUMOR	936	9364/3	Peripheral neuroectodermal tumor
		9365/3	Askin tumor
CHORDOMA	937	9370/3	Chordoma, NOS
		9371/3	Chondroid chordoma
		9372/3	Dedifferentiated chordoma
GANGLIONEUROBLASTOMA	949	9490/3	Ganglioneuroblastoma
NEUROBLASTOMA, NOS	950	9500/3	Neuroblastoma, NOS
		9501/3	Medulloepithelioma, NOS
		9502/3	Teratoid medulloepithelioma
		9503/3	Neuroepithelioma, NOS
		9504/3	Spongioneuroblastoma
		9505/3	Ganglioglioma, anaplastic
NEUROFIBROSARCOMA	954	9540/3	Malignant peripheral nerve sheath tumor
		9542/3	Epithelioid malignant peripheral nerve sheath tumor
NEURILEMMOMA	956	9560/3	Neurilemmoma, malignant
		9561/3	MPNST with rhabdomyoblastic differentiation
PERINEURIOMA	957	9571/3	Perineurioma, malignant
GRANULAR CELL TUMOR	958	9580/3	Granular cell tumor, malignant
		9581/3	Alveolar soft part sarcoma
MALIGNANT LYMPHOMA, NOS	959	9590/3	Malignant lymphoma, NOS
		9591/3	Malignant lymphoma, non-Hodgkin
		9596/3	Composite Hodgkin and non-Hodgkin lymphoma
HODGKIN LYMPHOMA	965	9650/3	Hodgkin lymphoma, NOS
		9651/3	Hodgkin lymphoma, lymphocyte-rich
		9652/3	Hodgkin lymphoma, mixed cellularity, NOS
		9653/3	Hodgkin lymphoma, lymphocytic deplet., NOS
		9654/3	Hodgkin lymph., lymphocyt. deplet., diffuse fibrosis
		9655/3	Hodgkin lymphoma, lymphocyt. deplet., reticular
		9659/3	Hodgkin lymph., nodular lymphocyte predom.
HODGKIN LYMPHOMA, NOD. SCLER.	966	9661/3	Hodgkin granuloma [obs]
		9662/3	Hodgkin sarcoma [obs]
		9663/3	Hodgkin lymphoma, nodular sclerosis, NOS
		9664/3	Hodgkin lymphoma, nod. scler., cellular phase
		9665/3	Hodgkin lymphoma, nod. scler., grade 1

PERIPHERAL NERVES C470-C476,C478-C479

HODGKIN LYMPHOMA, NOD. SCLER.	966	9667/3	Hodgkin lymphoma, nod. scler., grade 2
ML, SMALL B-CELL LYMPHOCYTIC	967	9670/3	ML, small B lymphocytic, NOS
		9671/3	ML, lymphoplasmacytic
		9673/3	Mantle cell lymphoma
		9675/3	ML, mixed sm. and lg. cell, diffuse
ML, LARGE B-CELL, DIFFUSE	968	9680/3	ML, large B-cell, diffuse
		9684/3	ML, large B-cell, diffuse, immunoblastic, NOS
		9687/3	Burkitt lymphoma, NOS
		9688/3	T-cell histiocyte rich large B-cell lymphoma
FOLLIC. & MARGINAL LYMPH, NOS	969	9690/3	Follicular lymphoma, NOS
		9691/3	Follicular lymphoma, grade 2
		9695/3	Follicular lymphoma, grade 1
		9698/3	Follicular lymphoma, grade 3
		9699/3	Marginal zone B-cell lymphoma, NOS
T-CELL LYMPHOMAS	970	9701/3	Sezary syndrome
		9702/3	Mature T-cell lymphoma, NOS
		9705/3	Angioimmunoblastic T-cell lymphoma
		9708/3	Subcutaneous panniculitis-like T-cell lymphoma
OTHER SPEC. NON-HODGKIN LYMPHOMA	971	9712/3	Intravascular large B-cell lymphoma
		9714/3	Anaplastic large cell lymphoma, T-cell and Null cell type
		9719/3	NK/T-cell lymphoma, nasal and nasal-type
PRECURS. CELL LYMPHOBLASTIC LYMPH.	972	9724/3	SystemicEBV pos. T-cell lymphoproliferative disease of childhood
		9726/3	Primary Cutaneous gamma-delta T-cell lymphoma
		9727/3	Precursor cell lymphoblastic lymphoma, NOS
		9728/3	Precursor B-cell lymphoblastic lymphoma
		9729/3	Precursor T-cell lymphoblastic lymphoma
PLASMA CELL TUMORS	973	9731/3	Plasmacytoma, NOS
		9734/3	Plasmacytoma, extramedullary
		9735/3	Plasmablastic lymphoma
		9737/3	ALK positive large B-cell lymphoma
		9738/3	Lrg B-cell lymphoma in HHV8-assoc. multicentric Castleman DZ
MAST CELL TUMORS	974	9740/3	Mast cell sarcoma
		9741/3	Malignant mastocytosis
NEOPLASMS OF HISTIOCYTES AND ACCESSORY LYMPHOID CELLS	975	9750/3	Malignant histiocytosis
		9751/3	Langerhans cell histiocytosis, NOS
		9754/3	Langerhans cell histiocytosis, disseminated
		9755/3	Histiocytic sarcoma
		9756/3	Langerhans cell sarcoma

PERIPHERAL NERVES C470-C476,C478-C479

NEOPLASMS OF HISTIOCYTES AND ACCESSORY LYMPHOID CELLS	975	9757/3	Interdigitating dendritic cell sarcoma
		9758/3	Follicular dendritic cell sarcoma
		9759/3	Fibroblastic reticular cell tumor
PRECURSOR LYMPHOID NEOPLASMS	981	9811/3	B lymphoblastic leukemia/lymphoma, NOS
		9812/3	Leukemia/lymphoma with t(9;22)(q34;q11.2);BCR-ABL1
		9813/3	Leukemia/lymphoma with t(v;11q23);MLL rearranged
		9814/3	Leukemia/lymphoma with t(12;21)(p13;q22);TEL-AML1(ETV6-RUNX1)
		9815/3	B lymphoblastic leukemia/lymphoma with hyperdiploidy
		9816/3	Leukemia/lymphoma with hypodiploidy (hypodiploid ALL)
		9817/3	B lymphblastic leukemia/lymphoma with t(5;14)(q31;q32);IL3-IGH
		9818/3	Leukemia/lymphoma with t(1;19)(q23;p13.3); E2A PBX1 (TCF3 PBX1)
LYMPHOID LEUKEMIA, NOS	982	9823/3	Chronic lymphocytic leukemia/small lymphocytic lymphoma
PROLYMPH/PRECURS LEUKEMIA	983	9831/3	T-cell large granular lymphocytic leukemia
		9837/3	T lymphoblastic leukemia/lymphoma
MYELOID SARCOMA	993	9930/3	Myeloid sarcoma
CHRONIC MYELOPROLIFERATIVE DIS.	996	9965/3	Myeloid and lymphoid neoplasms with PDGFRB rearrangement
		9967/3	Myeloid and lymphoid neoplasm with FGFR1 abnormalities
MYELOPLASTIC/MYELOPROLIFERATIVE NEOPLASMS	997	9971/3	Polymorphic PTLD
		9975/3	Myelodysplastic/Myeloproliferative neoplasm, unclassifiable

RETROPERITONEUM & PERITONEUM C480-C482,C488

NEOPLASM	800	8000/3	Neoplasm, malignant
		8001/3	Tumor cells, malignant
		8002/3	Malignant tumor, small cell type
		8003/3	Malignant tumor, giant cell type
		8004/3	Malignant tumor, spindle cell type
		8005/3	Malignant tumor, clear cell type
PAPILLARY SEROUS CYSTADENOCA	846	8461/3	Serous surface papillary carcinoma
PARAGANGLIOMA	868	8680/3	Paraganglioma, malignant
EXTRA-ADRENAL PARAGANG., MAL	869	8693/3	Extra-adrenal paraganglioma, malignant
GLOMANGIOSARCOMA	871	8714/3	PEComa, malignant
SARCOMA, NOS	880	8800/3	Sarcoma, NOS
		8801/3	Spindle cell sarcoma
		8802/3	Giant cell sarcoma
		8803/3	Small cell sarcoma
		8804/3	Epithelioid sarcoma
		8805/3	Undifferentiated sarcoma
		8806/3	Desmoplastic small round cell tumor
FIBROMATOUS NEOPLASMS	881	8810/3	Fibrosarcoma, NOS
		8811/3	Fibromyxosarcoma
		8813/3	Fascial fibrosarcoma
		8814/3	Infantile fibrosarcoma
		8815/3	Solitary fibrous tumor, malignant
SARCOMA, NOS	882	8825/3	Myofibroblastic sarcoma
FIBROUS HISTIOCYTOMA, MAL.	883	8830/3	Fibrous histiocytoma, malignant
MYXOSARCOMA	884	8840/3	Myxosarcoma
LIPOSARCOMA NEOPLASMS	885	8850/3	Liposarcoma, NOS
		8851/3	Liposarcoma, well differentiated
		8852/3	Myxoid liposarcoma
		8853/3	Round cell liposarcoma
		8854/3	Pleomorphic liposarcoma
		8855/3	Mixed type liposarcoma
		8857/3	Fibroblastic liposarcoma
		8858/3	Dedifferentiated liposarcoma

RETROPERITONEUM & PERITONEUM C480-C482,C488

MYOMATOUS NEOPLASMS	889	8890/3	Leiomyosarcoma, NOS
		8891/3	Epithelioid leiomyosarcoma
		8894/3	Angiomyosarcoma
		8895/3	Myosarcoma
		8896/3	Myxoid leiomyosarcoma
RHABDOMYOSARCOMA, NOS	890	8900/3	Rhabdomyosarcoma, NOS
		8901/3	Pleomorphic rhabdomyosarcoma, adult type
		8902/3	Mixed type rhabdomyosarcoma
EMBRYONAL RHABDOMYOSARCOMA	891	8910/3	Embryonal rhabdomyosarcoma
		8912/3	Spindle cell rhabdomyosarcoma
ALVEOLAR RHABDOMYOSARCOMA	892	8920/3	Alveolar rhabdomyosarcoma
		8921/3	Rhabdomyosarcoma with ganglionic differentiation
MULLERIAN MIXED TUMOR	895	8950/3	Mullerian mixed tumor
		8951/3	Mesodermal mixed tumor
MESENCHYMOMA, MALIGNANT	899	8990/3	Mesenchymoma, malignant
		8991/3	Embryonal sarcoma
MESOTHELIOMA, MALIGNANT	905	9050/3	Mesothelioma, malignant
		9051/3	Fibrous mesothelioma, malignant
		9052/3	Epithel. mesothelioma, mal.
		9053/3	Mesothelioma, biphasic, malignant
GERM CELL TUMORS	906	9060/3	Dysgerminoma
		9064/3	Germinoma
		9065/3	Germ cell tumor, nonseminomatous
EMBRYONAL CARCINOMA, NOS	907	9070/3	Embryonal carcinoma, NOS
		9071/3	Yolk sac tumor
		9072/3	Polyembryoma
TERATOMA	908	9080/3	Teratoma, malignant, NOS
		9081/3	Teratocarcinoma
		9082/3	Malignant teratoma, undiff.
		9083/3	Malignant teratoma, intermediate
		9084/3	Teratoma with malig. transformation
		9085/3	Mixed germ cell tumor
MESONEPHROMA, MALIGNANT	911	9110/3	Mesonephroma, malignant
BLOOD VESSEL TUMORS	912	9120/3	Hemangiosarcoma

RETROPERITONEUM & PERITONEUM C480-C482,C488

HEMANGIOENDOTHELIOMA	913	9130/3	Hemangioendothelioma, malignant
		9133/3	Epithelioid hemangioendothelioma, malignant
HEMANGIOPERICYTOMA	915	9150/3	Hemangiopericytoma, malignant
GANGLIONEUROBLASTOMA	949	9490/3	Ganglioneuroblastoma
NEUROBLASTOMA, NOS	950	9500/3	Neuroblastoma, NOS
		9501/3	Medulloepithelioma, NOS
		9502/3	Teratoid medulloepithelioma
		9503/3	Neuroepithelioma, NOS
		9504/3	Spongioneuroblastoma
		9505/3	Ganglioglioma, anaplastic
NEUROFIBROSARCOMA	954	9540/3	Malignant peripheral nerve sheath tumor
NEURILEMMOMA	956	9560/3	Neurilemmoma, malignant
		9561/3	MPNST with rhabdomyoblastic differentiation
PERINEURIOMA	957	9571/3	Perineurioma, malignant
MALIGNANT LYMPHOMA, NOS	959	9590/3	Malignant lymphoma, NOS
		9591/3	Malignant lymphoma, non-Hodgkin
		9596/3	Composite Hodgkin and non-Hodgkin lymphoma
HODGKIN LYMPHOMA	965	9650/3	Hodgkin lymphoma, NOS
		9651/3	Hodgkin lymphoma, lymphocyte-rich
		9652/3	Hodgkin lymphoma, mixed cellularity, NOS
		9653/3	Hodgkin lymphoma, lymphocytic deplet., NOS
		9654/3	Hodgkin lymph., lymphocyt. deplet., diffuse fibrosis
		9655/3	Hodgkin lymphoma, lymphocyt. deplet., reticular
		9659/3	Hodgkin lymph., nodular lymphocyte predom.
HODGKIN LYMPHOMA, NOD. SCLER.	966	9661/3	Hodgkin granuloma [obs]
		9662/3	Hodgkin sarcoma [obs]
		9663/3	Hodgkin lymphoma, nodular sclerosis, NOS
		9664/3	Hodgkin lymphoma, nod. scler., cellular phase
		9665/3	Hodgkin lymphoma, nod. scler., grade 1
		9667/3	Hodgkin lymphoma, nod. scler., grade 2
ML, SMALL B-CELL LYMPHOCYTIC	967	9670/3	ML, small B lymphocytic, NOS
		9671/3	ML, lymphoplasmacytic
		9673/3	Mantle cell lymphoma
		9675/3	ML, mixed sm. and lg. cell, diffuse

RETROPERITONEUM & PERITONEUM C480-C482,C488

ML, LARGE B-CELL, DIFFUSE	968	9680/3	ML, large B-cell, diffuse
		9684/3	ML, large B-cell, diffuse, immunoblastic, NOS
		9687/3	Burkitt lymphoma, NOS
		9688/3	T-cell histiocyte rich large B-cell lymphoma
FOLLIC. & MARGINAL LYMPH, NOS	969	9690/3	Follicular lymphoma, NOS
		9691/3	Follicular lymphoma, grade 2
		9695/3	Follicular lymphoma, grade 1
		9698/3	Follicular lymphoma, grade 3
		9699/3	Marginal zone B-cell lymphoma, NOS
T-CELL LYMPHOMAS	970	9701/3	Sezary syndrome
		9702/3	Mature T-cell lymphoma, NOS
		9705/3	Angioimmunoblastic T-cell lymphoma
OTHER SPEC. NON-HODGKIN LYMPHOMA	971	9712/3	Intravascular large B-cell lymphoma
		9714/3	Anaplastic large cell lymphoma, T-cell and Null cell type
		9719/3	NK/T-cell lymphoma, nasal and nasal-type
PRECURS. CELL LYMPHOBLASTIC LYMPH.	972	9724/3	SystemicEBV pos. T-cell lymphoproliferative disease of childhood
		9727/3	Precursor cell lymphoblastic lymphoma, NOS
		9728/3	Precursor B-cell lymphoblastic lymphoma
		9729/3	Precursor T-cell lymphoblastic lymphoma
PLASMA CELL TUMORS	973	9731/3	Plasmacytoma, NOS
		9734/3	Plasmacytoma, extramedullary
		9735/3	Plasmablastic lymphoma
		9737/3	ALK positive large B-cell lymphoma
		9738/3	Lrg B-cell lymphoma in HHV8-assoc. multicentric Castleman DZ
MAST CELL TUMORS	974	9740/3	Mast cell sarcoma
		9741/3	Malignant mastocytosis
NEOPLASMS OF HISTIOCYTES AND ACCESSORY LYMPHOID CELLS	975	9750/3	Malignant histiocytosis
		9751/3	Langerhans cell histiocytosis, NOS
		9754/3	Langerhans cell histiocytosis, disseminated
		9755/3	Histiocytic sarcoma
		9756/3	Langerhans cell sarcoma
		9757/3	Interdigitating dendritic cell sarcoma
		9758/3	Follicular dendritic cell sarcoma
		9759/3	Fibroblastic reticular cell tumor
PRECURSOR LYMPHOID NEOPLASMS	981	9811/3	B lymphoblastic leukemia/lymphoma, NOS
		9812/3	Leukemia/lymphoma with t(9;22)(q34;q11.2);BCR-ABL1
		9813/3	Leukemia/lymphoma with t(v;11q23);MLL rearranged
		9814/3	Leukemia/lymphoma with t(12;21)(p13;q22);TEL-AML1(ETV6-RUNX1)
		9815/3	B lymphoblastic leukemia/lymphoma with hyperdiploidy

RETROPERITONEUM & PERITONEUM C480-C482,C488

PRECURSOR LYMPHOID NEOPLASMS	981	9816/3	Leukemia/lymphoma with hypodiploidy (hypodiploid ALL)
		9817/3	B lymphblastic leukemia/lymphoma with t(5;14)(q31;q32);IL3-IGH
		9818/3	Leukemia/lymphoma with t(1;19)(q23;p13.3); E2A PBX1 (TCF3 PBX1)
LYMPHOID LEUKEMIA, NOS	982	9823/3	Chronic lymphocytic leukemia/small lymphocytic lymphoma
PROLYMPH/PRECURS LEUKEMIA	983	9831/3	T-cell large granular lymphocytic leukemia
		9837/3	T lymphoblastic leukemia/lymphoma
MYELOID SARCOMA	993	9930/3	Myeloid sarcoma
CHRONIC MYELOPROLIFERATIVE DIS.	996	9965/3	Myeloid and lymphoid neoplasms with PDGFRB rearrangement
		9967/3	Myeloid and lymphoid neoplasm with FGFR1 abnormalities
MYELOPLASTIC/MYELOPROLIFERATIVE NEOPLASMS	997	9971/3	Polymorphic PTLD
		9975/3	Myelodysplastic/Myeloproliferative neoplasm, unclassifiable

CONNECTIVE & SOFT TISSUE C490-C496,C498-C499

NEOPLASM	800	8000/3	Neoplasm, malignant
		8001/3	Tumor cells, malignant
		8002/3	Malignant tumor, small cell type
		8003/3	Malignant tumor, giant cell type
		8004/3	Malignant tumor, spindle cell type
		8005/3	Malignant tumor, clear cell type
PARAGANGLIOMA	868	8680/3	Paraganglioma, malignant
EXTRA-ADRENAL PARAGANG., MAL	869	8693/3	Extra-adrenal paraganglioma, malignant
SARCOMA, NOS	880	8800/3	Sarcoma, NOS
		8801/3	Spindle cell sarcoma
		8802/3	Giant cell sarcoma
		8803/3	Small cell sarcoma
		8804/3	Epithelioid sarcoma
		8805/3	Undifferentiated sarcoma
		8806/3	Desmoplastic small round cell tumor
FIBROMATOUS NEOPLASMS	881	8810/3	Fibrosarcoma, NOS
		8811/3	Fibromyxosarcoma
		8813/3	Fascial fibrosarcoma
		8814/3	Infantile fibrosarcoma
		8815/3	Solitary fibrous tumor, malignant
SARCOMA, NOS	882	8825/3	Myofibroblastic sarcoma
FIBROUS HISTIOCYTOMA, MAL.	883	8830/3	Fibrous histiocytoma, malignant
		8832/3	Dermatofibrosarcoma, NOS
		8833/3	Pigmented dermatofibrosarcoma protuberans
MYXOSARCOMA	884	8840/3	Myxosarcoma
		8842/3	Pulmonary myxoid sarcoma with EWSR1-CREB1 translocation
LIPOSARCOMA NEOPLASMS	885	8850/3	Liposarcoma, NOS
		8851/3	Liposarcoma, well differentiated
		8852/3	Myxoid liposarcoma
		8853/3	Round cell liposarcoma
		8854/3	Pleomorphic liposarcoma
		8855/3	Mixed type liposarcoma
		8857/3	Fibroblastic liposarcoma
		8858/3	Dedifferentiated liposarcoma

CONNECTIVE & SOFT TISSUE C490-C496,C498-C499

MYOMATOUS NEOPLASMS	889	8890/3	Leiomyosarcoma, NOS
		8891/3	Epithelioid leiomyosarcoma
		8894/3	Angiomyosarcoma
		8895/3	Myosarcoma
		8896/3	Myxoid leiomyosarcoma
RHABDOMYOSARCOMA, NOS	890	8900/3	Rhabdomyosarcoma, NOS
		8901/3	Pleomorphic rhabdomyosarcoma, adult type
		8902/3	Mixed type rhabdomyosarcoma
EMBRYONAL RHABDOMYOSARCOMA	891	8910/3	Embryonal rhabdomyosarcoma
		8912/3	Spindle cell rhabdomyosarcoma
ALVEOLAR RHABDOMYOSARCOMA	892	8920/3	Alveolar rhabdomyosarcoma
		8921/3	Rhabdomyosarcoma with ganglionic differentiation
MULLERIAN MIXED TUMOR	895	8950/3	Mullerian mixed tumor
		8951/3	Mesodermal mixed tumor
MESENCHYMOMA, MALIGNANT	899	8990/3	Mesenchymoma, malignant
		8991/3	Embryonal sarcoma
SYNOVIAL SARCOMA, NOS	904	9040/3	Synovial sarcoma, NOS
		9041/3	Synovial sarcoma, spindle cell
		9042/3	Synovial sarcoma, epithelioid cell
		9043/3	Synovial sarcoma, biphasic
		9044/3	Clear cell sarcoma,NOS (except of kidney M-8964/3)
TERATOMA	908	9080/3	Teratoma, malignant, NOS
		9081/3	Teratocarcinoma
		9082/3	Malignant teratoma, undiff.
		9083/3	Malignant teratoma, intermediate
		9084/3	Teratoma with malig. transformation
		9085/3	Mixed germ cell tumor
BLOOD VESSEL TUMORS	912	9120/3	Hemangiosarcoma
HEMANGIOENDOTHELIOMA	913	9130/3	Hemangioendothelioma, malignant
		9133/3	Epithelioid hemangioendothelioma, malignant
		9137/3	Intimal sarcoma
KAPOSI SARCOMA	914	9140/3	Kaposi sarcoma
HEMANGIOPERICYTOMA	915	9150/3	Hemangiopericytoma, malignant
LYMPHANGIOSARCOMA	917	9170/3	Lymphangiosarcoma

CONNECTIVE & SOFT TISSUE C490-C496,C498-C499

OSSEOUS & CHONDROMATOUS NEOPLASMS	924	9240/3	Mesenchymal chondrosarcoma
		9242/3	Clear cell chondrosarcoma
		9243/3	Dedifferentiated chondrosarcoma
GT. CELL TUMOR OF BONE, MAL.	925	9251/3	Malignant giant cell tumor of soft parts
		9252/3	Malignant tenosynovial giant cell tumor
PERIPHERAL NEUROECTODERMAL TUMOR	936	9364/3	Peripheral neuroectodermal tumor
		9365/3	Askin tumor
CHORDOMA	937	9370/3	Chordoma, NOS
		9371/3	Chondroid chordoma
		9372/3	Dedifferentiated chordoma
GANGLIONEUROBLASTOMA	949	9490/3	Ganglioneuroblastoma
NEUROBLASTOMA, NOS	950	9500/3	Neuroblastoma, NOS
		9501/3	Medulloepithelioma, NOS
		9502/3	Teratoid medulloepithelioma
		9503/3	Neuroepithelioma, NOS
		9504/3	Spongioneuroblastoma
		9505/3	Ganglioglioma, anaplastic
NEUROFIBROSARCOMA	954	9540/3	Malignant peripheral nerve sheath tumor
NEURILEMMOMA	956	9560/3	Neurilemmoma, malignant
		9561/3	MPNST with rhabdomyoblastic differentiation
PERINEURIOMA	957	9571/3	Perineurioma, malignant
GRANULAR CELL TUMOR	958	9580/3	Granular cell tumor, malignant
		9581/3	Alveolar soft part sarcoma
MALIGNANT LYMPHOMA, NOS	959	9590/3	Malignant lymphoma, NOS
		9591/3	Malignant lymphoma, non-Hodgkin
		9596/3	Composite Hodgkin and non-Hodgkin lymphoma
HODGKIN LYMPHOMA	965	9650/3	Hodgkin lymphoma, NOS
		9651/3	Hodgkin lymphoma, lymphocyte-rich
		9652/3	Hodgkin lymphoma, mixed cellularity, NOS
		9653/3	Hodgkin lymphoma, lymphocytic deplet., NOS
		9654/3	Hodgkin lymph., lymphocyt. deplet., diffuse fibrosis
		9655/3	Hodgkin lymphoma, lymphocyt. deplet., reticular
		9659/3	Hodgkin lymph., nodular lymphocyte predom.

CONNECTIVE & SOFT TISSUE C490-C496,C498-C499

HODGKIN LYMPHOMA, NOD. SCLER.	966	9661/3	Hodgkin granuloma [obs]
		9662/3	Hodgkin sarcoma [obs]
		9663/3	Hodgkin lymphoma, nodular sclerosis, NOS
		9664/3	Hodgkin lymphoma, nod. scler., cellular phase
		9665/3	Hodgkin lymphoma, nod. scler., grade 1
		9667/3	Hodgkin lymphoma, nod. scler., grade 2
ML, SMALL B-CELL LYMPHOCYTIC	967	9670/3	ML, small B lymphocytic, NOS
		9671/3	ML, lymphoplasmacytic
		9673/3	Mantle cell lymphoma
		9675/3	ML, mixed sm. and lg. cell, diffuse
ML, LARGE B-CELL, DIFFUSE	968	9680/3	ML, large B-cell, diffuse
		9684/3	ML, large B-cell, diffuse, immunoblastic, NOS
		9687/3	Burkitt lymphoma, NOS
		9688/3	T-cell histiocyte rich large B-cell lymphoma
FOLLIC. & MARGINAL LYMPH, NOS	969	9690/3	Follicular lymphoma, NOS
		9691/3	Follicular lymphoma, grade 2
		9695/3	Follicular lymphoma, grade 1
		9698/3	Follicular lymphoma, grade 3
		9699/3	Marginal zone B-cell lymphoma, NOS
T-CELL LYMPHOMAS	970	9701/3	Sezary syndrome
		9702/3	Mature T-cell lymphoma, NOS
		9705/3	Angioimmunoblastic T-cell lymphoma
		9708/3	Subcutaneous panniculitis-like T-cell lymphoma
OTHER SPEC. NON-HODGKIN LYMPHOMA	971	9712/3	Intravascular large B-cell lymphoma
		9714/3	Anaplastic large cell lymphoma, T-cell and Null cell type
		9719/3	NK/T-cell lymphoma, nasal and nasal-type
PRECURS. CELL LYMPHOBLASTIC LYMPH.	972	9724/3	SystemicEBV pos. T-cell lymphoproliferative disease of childhood
		9726/3	Primary Cutaneous gamma-delta T-cell lymphoma
		9727/3	Precursor cell lymphoblastic lymphoma, NOS
		9728/3	Precursor B-cell lymphoblastic lymphoma
		9729/3	Precursor T-cell lymphoblastic lymphoma
PLASMA CELL TUMORS	973	9731/3	Plasmacytoma, NOS
		9734/3	Plasmacytoma, extramedullary
		9735/3	Plasmablastic lymphoma
		9737/3	ALK positive large B-cell lymphoma
		9738/3	Lrg B-cell lymphoma in HHV8-assoc. multicentric Castleman DZ

CONNECTIVE & SOFT TISSUE C490-C496,C498-C499

MAST CELL TUMORS	974	9740/3	Mast cell sarcoma
		9741/3	Malignant mastocytosis
NEOPLASMS OF HISTIOCYTES AND ACCESSORY LYMPHOID CELLS	975	9750/3	Malignant histiocytosis
		9751/3	Langerhans cell histiocytosis, NOS
		9754/3	Langerhans cell histiocytosis, disseminated
		9755/3	Histiocytic sarcoma
		9756/3	Langerhans cell sarcoma
		9757/3	Interdigitating dendritic cell sarcoma
		9758/3	Follicular dendritic cell sarcoma
		9759/3	Fibroblastic reticular cell tumor
PRECURSOR LYMPHOID NEOPLASMS	981	9811/3	B lymphoblastic leukemia/lymphoma, NOS
		9812/3	Leukemia/lymphoma with t(9;22)(q34;q11.2);BCR-ABL1
		9813/3	Leukemia/lymphoma with t(v;11q23);MLL rearranged
		9814/3	Leukemia/lymphoma with t(12;21)(p13;q22);TEL-AML1(ETV6-RUNX1)
		9815/3	B lymphoblastic leukemia/lymphoma with hyperdiploidy
		9816/3	Leukemia/lymphoma with hypodiploidy (hypodiploid ALL)
		9817/3	B lymphblastic leukemia/lymphoma with t(5;14)(q31;q32);IL3-IGH
		9818/3	Leukemia/lymphoma with t(1;19)(q23;p13.3); E2A PBX1 (TCF3 PBX1)
LYMPHOID LEUKEMIA, NOS	982	9823/3	Chronic lymphocytic leukemia/small lymphocytic lymphoma
PROLYMPH/PRECURS LEUKEMIA	983	9831/3	T-cell large granular lymphocytic leukemia
		9837/3	T lymphoblastic leukemia/lymphoma
MYELOID SARCOMA	993	9930/3	Myeloid sarcoma
CHRONIC MYELOPROLIFERATIVE DIS.	996	9965/3	Myeloid and lymphoid neoplasms with PDGFRB rearrangement
		9967/3	Myeloid and lymphoid neoplasm with FGFR1 abnormalities
MYELOPLASTIC/MYELOPROLIFERATIVE NEOPLASMS	997	9971/3	Polymorphic PTLD
		9975/3	Myelodysplastic/Myeloproliferative neoplasm, unclassifiable

BREAST C500-C506,C508-C509

NEOPLASM	800	8000/3	Neoplasm, malignant
		8001/3	Tumor cells, malignant
		8002/3	Malignant tumor, small cell type
		8003/3	Malignant tumor, giant cell type
		8004/3	Malignant tumor, spindle cell type
		8005/3	Malignant tumor, clear cell type
CARCINOMA, NOS	801	8010/2	Carcinoma in situ, NOS
		8010/3	Carcinoma, NOS
		8011/3	Epithelioma, malignant
		8012/3	Large cell carcinoma, NOS
		8013/3	Large cell neuroendocrine carcinoma
		8014/3	Large cell carcinoma with rhabdoid phenotype
		8015/3	Glassy cell carcinoma
CARCINOMA, UNDIFF., NOS	802	8020/3	Carcinoma, undifferentiated type, NOS
		8021/3	Carcinoma, anaplastic type, NOS
		8022/3	Pleomorphic carcinoma
GIANT & SPINDLE CELL CARCINOMA	803	8030/3	Giant cell and spindle cell carcinoma
		8031/3	Giant cell carcinoma
		8032/3	Spindle cell carcinoma
		8033/3	Pseudosarcomatous carcinoma
		8034/3	Polygonal cell carcinoma
		8035/3	Carcinoma with osteoclast-like giant cells
SMALL CELL CARCINOMA, NOS	804	8041/3	Small cell carcinoma, NOS
		8043/3	Small cell carcinoma, fusiform cell
PAPILLARY CARCINOMA, NOS	805	8050/2	Papillary carcinoma in situ
		8050/3	Papillary carcinoma, NOS
		8051/3	Verrucous carcinoma, NOS
		8052/2	Papillary squamous cell carcinoma, non-invasive
		8052/3	Papillary squamous cell carcinoma
SQUAMOUS CELL CARCINOMA, NOS	807	8070/2	Squamous cell carcinoma in situ, NOS
		8070/3	Squamous cell carcinoma, NOS
		8071/3	Sq. cell carcinoma, keratinizing, NOS
		8072/3	Sq. cell carcinoma, lg. cell, non-ker.
		8073/3	Sq. cell carcinoma, sm. cell, non-ker.
		8074/3	Sq. cell carcinoma, spindle cell
		8075/3	Squamous cell carcinoma, adenoid
		8076/2	Sq. cell carc. in situ with question. stromal invas.
		8076/3	Sq. cell carcinoma, micro-invasive
		8078/3	Squamous cell carcinoma with horn formation

BREAST C500-C506,C508-C509

ADENOCARCINOMA, NOS	814	8140/2	Adenocarcinoma in situ
		8140/3	Adenocarcinoma, NOS
		8141/3	Scirrhous adenocarcinoma
		8143/3	Superficial spreading adenocarcinoma
		8147/3	Basal cell adenocarcinoma
TRABECULAR ADENOCARCINOMA	819	8190/3	Trabecular adenocarcinoma
ADENOID CYSTIC & CRIBRIFORM CA.	820	8200/3	Adenoid cystic carcinoma
		8201/2	Cribriform carcinoma in situ
		8201/3	Cribriform carcinoma
ADENOCA. IN ADENOMA. POLYP	821	8211/3	Tubular adenocarcinoma
SOLID CARCINOMA, NOS	823	8230/2	Duct carcinoma in situ, solid type
		8230/3	Solid carcinoma, NOS
		8231/3	Carcinoma simplex
BRONCHIOLO-ALVEOLAR ADENOCA.	825	8251/3	Alveolar adenocarcinoma
		8255/3	Adenocarcinoma with mixed subtypes
PAPILLARY ADENOCARCINOMA, NOS	826	8260/3	Papillary adenocarcinoma, NOS
		8261/2	Adenocarcinoma in situ in villous adenoma
		8261/3	Adenocarcinoma in villous adenoma
CLEAR CELL ADENOCARCINOMA, NOS	831	8310/3	Clear cell adenocarcinoma, NOS
		8314/3	Lipid-rich carcinoma
		8315/3	Glycogen-rich carcinoma
GRANULAR CELL CARCINOMA	832	8320/3	Granular cell carcinoma
		8323/3	Mixed cell adenocarcinoma
SWEAT GLAND ADENOCARCINOMA	840	8401/3	Apocrine adenocarcinoma
CYSTADENOCARCINOMA, NOS	844	8440/3	Cystadenocarcinoma, NOS
MUCINOUS ADENOCARCINOMA	848	8480/3	Mucinous adenocarcinoma
		8481/3	Mucin-producing adenocarcinoma
SIGNET RING CELL CARCINOMA	849	8490/3	Signet ring cell carcinoma
DUCT CARCINOMA	850	8500/2	Intraductal carcinoma, noninfiltrating, NOS
		8500/3	Invasive carcinoma of no special type
		8501/2	Comedocarcinoma, non-infiltrating
		8501/3	Comedocarcinoma, NOS
		8502/3	Secretory carcinoma of breast
		8503/2	Noninfiltrating intraductal papillary adenocarcinoma

BREAST C500-C506,C508-C509

DUCT CARCINOMA	850	8503/3	Intraductal papillary adenocarcinoma with invasion
		8504/2	Noninfiltrating intracystic carcinoma
		8504/3	Intracystic carcinoma, NOS
		8507/2	Intraductal micropapillary carcinoma
		8507/3	Invasive micropapillary carcinoma
		8508/3	Cystic hypersecretory carcinoma
		8509/2	Solid papillary carcinoma in situ
		8509/3	Solid papillary carcinoma with invasion
MEDULLARY CARCINOMA, NOS	851	8510/3	Medullary carcinoma, NOS
		8512/3	Medullary carcinoma with lymphoid stroma
		8513/3	Atypical medullary carcinoma
		8514/3	Duct carcinoma, desmoplastic type
		8519/2	Pleomorphic lobular carcinoma in situ
LOBULAR AND OTHER DUCTAL CA.	852	8520/2	Lobular carcinoma in situ
		8520/3	Lobular carcinoma, NOS
		8521/3	Infiltrating ductular carcinoma
		8522/2	Intraductal and lobular in situ carcinoma
		8522/3	Infiltrating duct and lobular carcinoma
		8523/2	Infiltr. duct mixed with other types of carcinoma, in situ
		8523/3	Infiltr. duct mixed with other types of carcinoma
		8524/3	Infiltrating lobular mixed with other types of carc.
		8525/3	Polymorphous low grade adenocarcinoma
INFLAMMATORY CARCINOMA	853	8530/3	Inflammatory carcinoma
PAGET DISEASE, MAMMARY	854	8540/3	Paget disease, mammary
		8541/3	Paget dis. & infil. duct carcinoma
		8543/3	Paget disease and intraductal ca.
ACINAR CELL CARCINOMA	855	8550/3	Acinar cell carcinoma
		8551/3	Acinar cell cystadenocarcinoma
ADENOSQUAMOUS CARCINOMA	856	8560/3	Adenosquamous carcinoma
		8562/3	Epithelial-myoepithelial carcinoma
ADENOCA. WITH METAPLASIA	857	8570/3	Adenocarcinoma with squamous metaplasia
		8571/3	Adenocarcinoma w cartilag. & oss. metaplas.
		8572/3	Adenocarcinoma with spindle cell mataplasia
		8573/3	Adenocarcinoma with apocrine metaplasia
		8574/3	Adenocarcinoma with neuroendocrine differen.
		8575/3	Metaplastic carcinoma, NOS

BREAST C500-C506,C508-C509

SARCOMA, NOS	880	8800/3	Sarcoma, NOS
		8801/3	Spindle cell sarcoma
		8802/3	Giant cell sarcoma
		8803/3	Small cell sarcoma
		8804/3	Epithelioid sarcoma
		8805/3	Undifferentiated sarcoma
		8806/3	Desmoplastic small round cell tumor
FIBROMATOUS NEOPLASMS	881	8810/3	Fibrosarcoma, NOS
		8811/3	Fibromyxosarcoma
		8813/3	Fascial fibrosarcoma
		8814/3	Infantile fibrosarcoma
		8815/3	Solitary fibrous tumor, malignant
SARCOMA, NOS	882	8825/3	Myofibroblastic sarcoma
LIPOSARCOMA NEOPLASMS	885	8850/3	Liposarcoma, NOS
		8851/3	Liposarcoma, well differentiated
		8852/3	Myxoid liposarcoma
		8853/3	Round cell liposarcoma
		8854/3	Pleomorphic liposarcoma
		8855/3	Mixed type liposarcoma
		8857/3	Fibroblastic liposarcoma
		8858/3	Dedifferentiated liposarcoma
MYOMATOUS NEOPLASMS	889	8890/3	Leiomyosarcoma, NOS
		8891/3	Epithelioid leiomyosarcoma
		8894/3	Angiomyosarcoma
		8895/3	Myosarcoma
		8896/3	Myxoid leiomyosarcoma
STROMAL SARCOMA	893	8935/3	Stromal sarcoma, NOS
CARCINOSARCOMA, NOS	898	8980/3	Carcinosarcoma, NOS
		8981/3	Carcinosarcoma, embryonal type
		8982/3	Malignant myoepithelioma
		8983/3	Adenomyoepithelioma with carcinoma
MESENCHYMOMA, MALIGNANT	899	8990/3	Mesenchymoma, malignant
		8991/3	Embryonal sarcoma
PHYLLODES TUMOR,MAL.	902	9020/3	Phyllodes tumor, malignant
BLOOD VESSEL TUMORS	912	9120/3	Hemangiosarcoma

BREAST C500-C506,C508-C509

HEMANGIOENDOTHELIOMA	913	9130/3	Hemangioendothelioma, malignant
		9133/3	Epithelioid hemangioendothelioma, malignant
GRANULAR CELL TUMOR	958	9580/3	Granular cell tumor, malignant
		9581/3	Alveolar soft part sarcoma
MALIGNANT LYMPHOMA, NOS	959	9590/3	Malignant lymphoma, NOS
		9591/3	Malignant lymphoma, non-Hodgkin
		9596/3	Composite Hodgkin and non-Hodgkin lymphoma
HODGKIN LYMPHOMA	965	9650/3	Hodgkin lymphoma, NOS
		9651/3	Hodgkin lymphoma, lymphocyte-rich
		9652/3	Hodgkin lymphoma, mixed cellularity, NOS
		9653/3	Hodgkin lymphoma, lymphocytic deplet., NOS
		9654/3	Hodgkin lymph., lymphocyt. deplet., diffuse fibrosis
		9655/3	Hodgkin lymphoma, lymphocyt. deplet., reticular
		9659/3	Hodgkin lymph., nodular lymphocyte predom.
HODGKIN LYMPHOMA, NOD. SCLER.	966	9661/3	Hodgkin granuloma [obs]
		9662/3	Hodgkin sarcoma [obs]
		9663/3	Hodgkin lymphoma, nodular sclerosis, NOS
		9664/3	Hodgkin lymphoma, nod. scler., cellular phase
		9665/3	Hodgkin lymphoma, nod. scler., grade 1
		9667/3	Hodgkin lymphoma, nod. scler., grade 2
ML, SMALL B-CELL LYMPHOCYTIC	967	9670/3	ML, small B lymphocytic, NOS
		9671/3	ML, lymphoplasmacytic
		9673/3	Mantle cell lymphoma
		9675/3	ML, mixed sm. and lg. cell, diffuse
ML, LARGE B-CELL, DIFFUSE	968	9680/3	ML, large B-cell, diffuse
		9684/3	ML, large B-cell, diffuse, immunoblastic, NOS
		9687/3	Burkitt lymphoma, NOS
		9688/3	T-cell histiocyte rich large B-cell lymphoma
FOLLIC. & MARGINAL LYMPH, NOS	969	9690/3	Follicular lymphoma, NOS
		9691/3	Follicular lymphoma, grade 2
		9695/3	Follicular lymphoma, grade 1
		9698/3	Follicular lymphoma, grade 3
		9699/3	Marginal zone B-cell lymphoma, NOS
T-CELL LYMPHOMAS	970	9701/3	Sezary syndrome
		9702/3	Mature T-cell lymphoma, NOS
		9705/3	Angioimmunoblastic T-cell lymphoma

BREAST C500-C506,C508-C509

OTHER SPEC. NON-HODGKIN LYMPHOMA	971	9712/3	Intravascular large B-cell lymphoma
		9714/3	Anaplastic large cell lymphoma, T-cell and Null cell type
		9719/3	NK/T-cell lymphoma, nasal and nasal-type
PRECURS. CELL LYMPHOBLASTIC LYMPH.	972	9724/3	SystemicEBV pos. T-cell lymphoproliferative disease of childhood
		9727/3	Precursor cell lymphoblastic lymphoma, NOS
		9728/3	Precursor B-cell lymphoblastic lymphoma
		9729/3	Precursor T-cell lymphoblastic lymphoma
PLASMA CELL TUMORS	973	9731/3	Plasmacytoma, NOS
		9734/3	Plasmacytoma, extramedullary
MAST CELL TUMORS	974	9740/3	Mast cell sarcoma
		9741/3	Malignant mastocytosis
NEOPLASMS OF HISTIOCYTES AND ACCESSORY LYMPHOID CELLS	975	9750/3	Malignant histiocytosis
		9751/3	Langerhans cell histiocytosis, NOS
		9754/3	Langerhans cell histiocytosis, disseminated
		9755/3	Histiocytic sarcoma
		9756/3	Langerhans cell sarcoma
		9757/3	Interdigitating dendritic cell sarcoma
		9758/3	Follicular dendritic cell sarcoma
		9759/3	Fibroblastic reticular cell tumor
PRECURSOR LYMPHOID NEOPLASMS	981	9811/3	B lymphoblastic leukemia/lymphoma, NOS
		9812/3	Leukemia/lymphoma with t(9;22)(q34;q11.2);BCR-ABL1
		9813/3	Leukemia/lymphoma with t(v;11q23);MLL rearranged
		9814/3	Leukemia/lymphoma with t(12;21)(p13;q22);TEL-AML1(ETV6-RUNX1)
		9815/3	B lymphoblastic leukemia/lymphoma with hyperdiploidy
		9816/3	Leukemia/lymphoma with hypodiploidy (hypodiploid ALL)
		9817/3	B lymphblastic leukemia/lymphoma with t(5;14)(q31;q32);IL3-IGH
		9818/3	Leukemia/lymphoma with t(1;19)(q23;p13.3); E2A PBX1 (TCF3 PBX1)
LYMPHOID LEUKEMIA, NOS	982	9823/3	Chronic lymphocytic leukemia/small lymphocytic lymphoma
PROLYMPH/PRECURS LEUKEMIA	983	9831/3	T-cell large granular lymphocytic leukemia
		9837/3	T lymphoblastic leukemia/lymphoma
CHRONIC MYELOPROLIFERATIVE DIS.	996	9965/3	Myeloid and lymphoid neoplasms with PDGFRB rearrangement
		9967/3	Myeloid and lymphoid neoplasm with FGFR1 abnormalities
MYELOPLASTIC/MYELOPROLIFERATIVE NEOPLASMS	997	9971/3	Polymorphic PTLD
		9975/3	Myelodysplastic/Myeloproliferative neoplasm, unclassifiable

VAGINA & LABIA C510-C512,C518, C529

NEOPLASM	800	8000/3	Neoplasm, malignant
		8001/3	Tumor cells, malignant
		8002/3	Malignant tumor, small cell type
		8003/3	Malignant tumor, giant cell type
		8004/3	Malignant tumor, spindle cell type
		8005/3	Malignant tumor, clear cell type
CARCINOMA, NOS	801	8010/2	Carcinoma in situ, NOS
		8010/3	Carcinoma, NOS
		8011/3	Epithelioma, malignant
		8012/3	Large cell carcinoma, NOS
		8013/3	Large cell neuroendocrine carcinoma
		8014/3	Large cell carcinoma with rhabdoid phenotype
		8015/3	Glassy cell carcinoma
CARCINOMA, UNDIFF., NOS	802	8020/3	Carcinoma, undifferentiated type, NOS
		8021/3	Carcinoma, anaplastic type, NOS
		8022/3	Pleomorphic carcinoma
PAPILLARY CARCINOMA, NOS	805	8050/2	Papillary carcinoma in situ
		8050/3	Papillary carcinoma, NOS
		8051/3	Verrucous carcinoma, NOS
		8052/2	Papillary squamous cell carcinoma, non-invasive
		8052/3	Papillary squamous cell carcinoma
SQUAMOUS CELL CARCINOMA, NOS	807	8070/2	Squamous cell carcinoma in situ, NOS
		8070/3	Squamous cell carcinoma, NOS
		8071/2	Sq. cell carcinoma, keratinizing, NOS, in situ
		8071/3	Sq. cell carcinoma, keratinizing, NOS
		8072/3	Sq. cell carcinoma, lg. cell, non-ker.
		8073/3	Sq. cell carcinoma, sm. cell, non-ker.
		8074/3	Sq. cell carcinoma, spindle cell
		8075/3	Squamous cell carcinoma, adenoid
		8076/2	Sq. cell carc. in situ with question. stromal invas.
		8076/3	Sq. cell carcinoma, micro-invasive
		8077/2	Squamous intraepithelial neoplasia, grade III
		8078/3	Squamous cell carcinoma with horn formation
LYMPHOEPITHELIAL CARCINOMA	808	8081/2	Bowen disease
		8082/3	Lymphoepithelial carcinoma
		8083/3	Basaloid squamous cell carcinoma
		8084/3	Squamous cell carcinoma, clear cell type

VAGINA & LABIA C510-C512,C518, C529

BASAL CELL CARCINOMA, NOS	809	8090/3	Basal cell carcinoma, NOS
		8091/3	Multifocal superficial basal cell carcinoma
		8092/3	Infiltrating basal cell carcinoma, NOS
		8093/3	Basal cell carcinoma, fibroepithelial
		8094/3	Basosquamous carcinoma
		8095/3	Metatypical carcinoma
		8097/3	Basal cell carcinoma, nodular
		8098/3	Adenoid basal cell carcinoma
TRICHILEMMOCARCINOMA	810	8102/3	Trichilemmocarcinoma
PILOMATRIX CARCINOMA	811	8110/3	Pilomatrix carcinoma
TRANSITIONAL CELL CARCINOMA, NOS	812	8120/2	Transitional cell carcinoma in situ
		8120/3	Transitional cell carcinoma, NOS
		8121/3	Schneiderian carcinoma
		8122/3	Trans. cell carcinoma, spindle cell
		8123/3	Basaloid carcinoma
		8124/3	Cloacogenic carcinoma
PAPILLARY TRANS. CELL CARCINOMA	813	8130/2	Papillary trans. cell carcinoma, non-invasive
		8130/3	Papillary trans. cell carcinoma
		8131/3	Transitional cell carcinoma, micropapillary
ADENOCARCINOMA, NOS	814	8140/2	Adenocarcinoma in situ
		8140/3	Adenocarcinoma, NOS
		8141/3	Scirrhous adenocarcinoma
		8143/3	Superficial spreading adenocarcinoma
		8147/3	Basal cell adenocarcinoma
ADENOID CYSTIC & CRIBRIFORM CA.	820	8200/3	Adenoid cystic carcinoma
		8201/2	Cribriform carcinoma in situ
		8201/3	Cribriform carcinoma
BRONCHIOLO-ALVEOLAR ADENOCA.	825	8255/3	Adenocarcinoma with mixed subtypes
PAPILLARY ADENOCARCINOMA, NOS	826	8260/3	Papillary adenocarcinoma, NOS
		8261/2	Adenocarcinoma in situ in villous adenoma
		8261/3	Adenocarcinoma in villous adenoma
		8262/3	Villous adenocarcinoma
		8263/2	Adenocarcinoma in situ in tubulovillous adenoma
		8263/3	Adenocarcinoma in tubulovillous adenoma
CLEAR CELL ADENOCARCINOMA, NOS	831	8310/3	Clear cell adenocarcinoma, NOS
GRANULAR CELL CARCINOMA	832	8323/3	Mixed cell adenocarcinoma

VAGINA & LABIA C510-C512,C518, C529

ENDOMETRIOID ADENOCARCINOMA	838	8380/3	Endometrioid carcinoma
SWEAT GLAND ADENOCARCINOMA	840	8401/3	Apocrine adenocarcinoma
MUCINOUS ADENOCARCINOMA	848	8480/3	Mucinous adenocarcinoma
		8481/3	Mucin-producing adenocarcinoma
		8482/3	Mucinous adenocarcinoma, endocervical type
PAGET DISEASE, EXTRAMAMMARY	854	8542/3	Paget disease, extramammary
ADENOSQUAMOUS CARCINOMA	856	8560/3	Adenosquamous carcinoma
		8562/3	Epithelial-myoepithelial carcinoma
ADENOCA. WITH METAPLASIA	857	8570/3	Adenocarcinoma with squamous metaplasia
		8571/3	Adenocarcinoma w cartilag. & oss. metaplas.
		8572/3	Adenocarcinoma with spindle cell mataplasia
		8573/3	Adenocarcinoma with apocrine metaplasia
		8574/3	Adenocarcinoma with neuroendocrine differen.
		8575/3	Metaplastic carcinoma, NOS
		8576/3	Hepatoid adenocarcinoma
NEVI & MELANOMAS	872	8720/2	Melanoma in situ
		8720/3	Malignant melanoma, NOS
		8721/3	Nodular melanoma
		8722/3	Balloon cell melanoma
		8723/3	Malignant melanoma, regressing
AMELANOTIC MELANOMA	873	8730/3	Amelanotic melanoma
MAL. MEL. IN JUNCT. NEVUS	874	8740/3	Mal. melanoma in junctional nevus
		8741/2	Precancerous melanosis, NOS
		8741/3	Mal. melanoma in precan. melanosis
		8742/2	Lentigo maligna
		8742/3	Lentigo maligna melanoma
		8743/3	Superficial spreading melanoma
		8745/3	Desmoplastic melanoma, malignant
		8746/3	Mucosal lentiginous melanoma
MAL. MELAN. IN GIANT PIGMT. NEVUS	876	8761/3	Mal. melanoma in giant pigmented nevus
EPITHELIOID CELL MELANOMA	877	8770/3	Mixed epithel. & spindle cell melanoma
		8771/3	Epithelioid cell melanoma
		8772/3	Spindle cell melanoma, NOS

VAGINA & LABIA C510-C512,C518, C529

SARCOMA, NOS	880	8800/3	Sarcoma, NOS
		8801/3	Spindle cell sarcoma
		8802/3	Giant cell sarcoma
		8803/3	Small cell sarcoma
		8804/3	Epithelioid sarcoma
		8805/3	Undifferentiated sarcoma
		8806/3	Desmoplastic small round cell tumor
FIBROMATOUS NEOPLASMS	881	8810/3	Fibrosarcoma, NOS
		8811/3	Fibromyxosarcoma
		8813/3	Fascial fibrosarcoma
		8814/3	Infantile fibrosarcoma
		8815/3	Solitary fibrous tumor, malignant
SARCOMA, NOS	882	8825/3	Myofibroblastic sarcoma
FIBROUS HISTIOCYTOMA, MAL.	883	8830/3	Fibrous histiocytoma, malignant
		8832/3	Dermatofibrosarcoma, NOS
LIPOSARCOMA NEOPLASMS	885	8850/3	Liposarcoma, NOS
		8851/3	Liposarcoma, well differentiated
		8852/3	Myxoid liposarcoma
		8853/3	Round cell liposarcoma
		8854/3	Pleomorphic liposarcoma
		8855/3	Mixed type liposarcoma
		8857/3	Fibroblastic liposarcoma
		8858/3	Dedifferentiated liposarcoma
MYOMATOUS NEOPLASMS	889	8890/3	Leiomyosarcoma, NOS
		8891/3	Epithelioid leiomyosarcoma
		8894/3	Angiomyosarcoma
		8895/3	Myosarcoma
		8896/3	Myxoid leiomyosarcoma
RHABDOMYOSARCOMA, NOS	890	8900/3	Rhabdomyosarcoma, NOS
		8901/3	Pleomorphic rhabdomyosarcoma, adult type
		8902/3	Mixed type rhabdomyosarcoma
EMBRYONAL RHABDOMYOSARCOMA	891	8910/3	Embryonal rhabdomyosarcoma
		8912/3	Spindle cell rhabdomyosarcoma
MULLERIAN MIXED TUMOR	895	8950/3	Mullerian mixed tumor
		8951/3	Mesodermal mixed tumor

VAGINA & LABIA C510-C512,C518, C529

CARCINOSARCOMA, NOS	898	8980/3	Carcinosarcoma, NOS
		8981/3	Carcinosarcoma, embryonal type
		8982/3	Malignant myoepithelioma
MESONEPHROMA, MALIGNANT	911	9110/3	Mesonephroma, malignant
NEUROFIBROSARCOMA	954	9540/3	Malignant peripheral nerve sheath tumor
MALIGNANT LYMPHOMA, NOS	959	9590/3	Malignant lymphoma, NOS
		9591/3	Malignant lymphoma, non-Hodgkin
		9596/3	Composite Hodgkin and non-Hodgkin lymphoma
		9597/3	Primary Cutaneous follicle centre lymphoma
HODGKIN LYMPHOMA	965	9650/3	Hodgkin lymphoma, NOS
		9651/3	Hodgkin lymphoma, lymphocyte-rich
		9652/3	Hodgkin lymphoma, mixed cellularity, NOS
		9653/3	Hodgkin lymphoma, lymphocytic deplet., NOS
		9654/3	Hodgkin lymph., lymphocyt. deplet., diffuse fibrosis
		9655/3	Hodgkin lymphoma, lymphocyt. deplet., reticular
		9659/3	Hodgkin lymph., nodular lymphocyte predom.
HODGKIN LYMPHOMA, NOD. SCLER.	966	9661/3	Hodgkin granuloma [obs]
		9662/3	Hodgkin sarcoma [obs]
		9663/3	Hodgkin lymphoma, nodular sclerosis, NOS
		9664/3	Hodgkin lymphoma, nod. scler., cellular phase
		9665/3	Hodgkin lymphoma, nod. scler., grade 1
		9667/3	Hodgkin lymphoma, nod. scler., grade 2
ML, SMALL B-CELL LYMPHOCYTIC	967	9670/3	ML, small B lymphocytic, NOS
		9671/3	ML, lymphoplasmacytic
		9673/3	Mantle cell lymphoma
		9675/3	ML, mixed sm. and lg. cell, diffuse
ML, LARGE B-CELL, DIFFUSE	968	9680/3	ML, large B-cell, diffuse
		9684/3	ML, large B-cell, diffuse, immunoblastic, NOS
		9687/3	Burkitt lymphoma, NOS
		9688/3	T-cell histiocyte rich large B-cell lymphoma
FOLLIC. & MARGINAL LYMPH, NOS	969	9690/3	Follicular lymphoma, NOS
		9691/3	Follicular lymphoma, grade 2
		9695/3	Follicular lymphoma, grade 1
		9698/3	Follicular lymphoma, grade 3
		9699/3	Marginal zone B-cell lymphoma, NOS

VAGINA & LABIA C510-C512,C518, C529

T-CELL LYMPHOMAS	970	9700/3	Mycosis fungoides
		9701/3	Sezary syndrome
		9702/3	Mature T-cell lymphoma, NOS
		9705/3	Angioimmunoblastic T-cell lymphoma
		9708/3	Subcutaneous panniculitis-like T-cell lymphoma
		9709/3	Cutaneous T-cell lymphoma, NOS
OTHER SPEC. NON-HODGKIN LYMPHOMA	971	9712/3	Intravascular large B-cell lymphoma
		9714/3	Anaplastic large cell lymphoma, T-cell and Null cell type
		9718/3	Primary cutan. CD30+ T-cell lymphoprolif. disorder
		9719/3	NK/T-cell lymphoma, nasal and nasal-type
PRECURS. CELL LYMPHOBLASTIC LYMPH.	972	9724/3	SystemicEBV pos. T-cell lymphoproliferative disease of childhood
		9725/3	Hydroa vacciniforme-like lymphoma
		9726/3	Primary Cutaneous gamma-delta T-cell lymphoma
		9727/3	Precursor cell lymphoblastic lymphoma, NOS
		9728/3	Precursor B-cell lymphoblastic lymphoma
		9729/3	Precursor T-cell lymphoblastic lymphoma
PLASMA CELL TUMORS	973	9731/3	Plasmacytoma, NOS
		9734/3	Plasmacytoma, extramedullary
		9735/3	Plasmablastic lymphoma
		9737/3	ALK positive large B-cell lymphoma
		9738/3	Lrg B-cell lymphoma in HHV8-assoc. multicentric Castleman DZ
MAST CELL TUMORS	974	9740/3	Mast cell sarcoma
		9741/3	Malignant mastocytosis
NEOPLASMS OF HISTIOCYTES AND ACCESSORY LYMPHOID CELLS	975	9750/3	Malignant histiocytosis
		9751/3	Langerhans cell histiocytosis, NOS
		9754/3	Langerhans cell histiocytosis, disseminated
		9755/3	Histiocytic sarcoma
		9756/3	Langerhans cell sarcoma
		9757/3	Interdigitating dendritic cell sarcoma
		9758/3	Follicular dendritic cell sarcoma
		9759/3	Fibroblastic reticular cell tumor
PRECURSOR LYMPHOID NEOPLASMS	981	9811/3	B lymphoblastic leukemia/lymphoma, NOS
		9812/3	Leukemia/lymphoma with t(9;22)(q34;q11.2);BCR-ABL1
		9813/3	Leukemia/lymphoma with t(v;11q23);MLL rearranged
		9814/3	Leukemia/lymphoma with t(12;21)(p13;q22);TEL-AML1(ETV6-RUNX1)
		9815/3	B lymphoblastic leukemia/lymphoma with hyperdiploidy
		9816/3	Leukemia/lymphoma with hypodiploidy (hypodiploid ALL)
		9817/3	B lymphblastic leukemia/lymphoma with t(5;14)(q31;q32);IL3-IGH
		9818/3	Leukemia/lymphoma with t(1;19)(q23;p13.3); E2A PBX1 (TCF3 PBX1)
LYMPHOID LEUKEMIA, NOS	982	9823/3	Chronic lymphocytic leukemia/small lymphocytic lymphoma

VAGINA & LABIA C510-C512,C518, C529

PROLYMPH/PRECURS LEUKEMIA	983	9831/3	T-cell large granular lymphocytic leukemia
		9837/3	T lymphoblastic leukemia/lymphoma
CHRONIC MYELOPROLIFERATIVE DIS.	996	9965/3	Myeloid and lymphoid neoplasms with PDGFRB rearrangement
		9967/3	Myeloid and lymphoid neoplasm with FGFR1 abnormalities
MYELOPLASTIC/MYELOPROLIFERATIVE NEOPLASMS	997	9971/3	Polymorphic PTLD
		9975/3	Myelodysplastic/Myeloproliferative neoplasm, unclassifiable

VULVA, NOS C519

NEOPLASM	800	8000/3	Neoplasm, malignant
		8001/3	Tumor cells, malignant
		8002/3	Malignant tumor, small cell type
		8003/3	Malignant tumor, giant cell type
		8004/3	Malignant tumor, spindle cell type
		8005/3	Malignant tumor, clear cell type
CARCINOMA, NOS	801	8010/2	Carcinoma in situ, NOS
		8010/3	Carcinoma, NOS
		8011/3	Epithelioma, malignant
		8012/3	Large cell carcinoma, NOS
		8013/3	Large cell neuroendocrine carcinoma
		8014/3	Large cell carcinoma with rhabdoid phenotype
		8015/3	Glassy cell carcinoma
CARCINOMA, UNDIFF., NOS	802	8020/3	Carcinoma, undifferentiated type, NOS
		8021/3	Carcinoma, anaplastic type, NOS
		8022/3	Pleomorphic carcinoma
PAPILLARY CARCINOMA, NOS	805	8050/2	Papillary carcinoma in situ
		8050/3	Papillary carcinoma, NOS
		8051/3	Verrucous carcinoma, NOS
		8052/2	Papillary squamous cell carcinoma, non-invasive
		8052/3	Papillary squamous cell carcinoma
SQUAMOUS CELL CARCINOMA, NOS	807	8070/2	Squamous cell carcinoma in situ, NOS
		8070/3	Squamous cell carcinoma, NOS
		8071/2	Sq. cell carcinoma, keratinizing, NOS, in situ
		8071/3	Sq. cell carcinoma, keratinizing, NOS
		8072/3	Sq. cell carcinoma, lg. cell, non-ker.
		8073/3	Sq. cell carcinoma, sm. cell, non-ker.
		8074/3	Sq. cell carcinoma, spindle cell
		8075/3	Squamous cell carcinoma, adenoid
		8076/2	Sq. cell carc. in situ with question. stromal invas.
		8076/3	Sq. cell carcinoma, micro-invasive
		8077/2	Squamous intraepithelial neoplasia, grade III
		8078/3	Squamous cell carcinoma with horn formation
LYMPHOEPITHELIAL CARCINOMA	808	8081/2	Bowen disease
		8082/3	Lymphoepithelial carcinoma
		8083/3	Basaloid squamous cell carcinoma
		8084/3	Squamous cell carcinoma, clear cell type

VULVA, NOS C519

BASAL CELL CARCINOMA, NOS	809	8090/3	Basal cell carcinoma, NOS
		8091/3	Multifocal superficial basal cell carcinoma
		8092/3	Infiltrating basal cell carcinoma, NOS
		8093/3	Basal cell carcinoma, fibroepithelial
		8094/3	Basosquamous carcinoma
		8095/3	Metatypical carcinoma
		8097/3	Basal cell carcinoma, nodular
		8098/3	Adenoid basal cell carcinoma
TRICHILEMMOCARCINOMA	810	8102/3	Trichilemmocarcinoma
PILOMATRIX CARCINOMA	811	8110/3	Pilomatrix carcinoma
TRANSITIONAL CELL CARCINOMA, NOS	812	8120/2	Transitional cell carcinoma in situ
		8120/3	Transitional cell carcinoma, NOS
		8121/3	Schneiderian carcinoma
		8122/3	Trans. cell carcinoma, spindle cell
		8123/3	Basaloid carcinoma
		8124/3	Cloacogenic carcinoma
PAPILLARY TRANS. CELL CARCINOMA	813	8130/2	Papillary trans. cell carcinoma, non-invasive
		8130/3	Papillary trans. cell carcinoma
		8131/3	Transitional cell carcinoma, micropapillary
ADENOCARCINOMA, NOS	814	8140/2	Adenocarcinoma in situ
		8140/3	Adenocarcinoma, NOS
		8141/3	Scirrhous adenocarcinoma
		8143/3	Superficial spreading adenocarcinoma
		8147/3	Basal cell adenocarcinoma
ADENOID CYSTIC & CRIBRIFORM CA.	820	8200/3	Adenoid cystic carcinoma
		8201/2	Cribriform carcinoma in situ
		8201/3	Cribriform carcinoma
BRONCHIOLO-ALVEOLAR ADENOCA.	825	8255/3	Adenocarcinoma with mixed subtypes
PAPILLARY ADENOCARCINOMA, NOS	826	8260/3	Papillary adenocarcinoma, NOS
		8261/2	Adenocarcinoma in situ in villous adenoma
		8261/3	Adenocarcinoma in villous adenoma
		8262/3	Villous adenocarcinoma
		8263/2	Adenocarcinoma in situ in tubulovillous adenoma
		8263/3	Adenocarcinoma in tubulovillous adenoma
CLEAR CELL ADENOCARCINOMA, NOS	831	8310/3	Clear cell adenocarcinoma, NOS
GRANULAR CELL CARCINOMA	832	8323/3	Mixed cell adenocarcinoma

VULVA, NOS C519

ENDOMETRIOID ADENOCARCINOMA	838	8380/3	Endometrioid carcinoma
SKIN APPENDAGE CARCINOMA	839	8390/3	Skin appendage carcinoma
SWEAT GLAND ADENOCARCINOMA	840	8400/3	Sweat gland adenocarcinoma
		8401/3	Apocrine adenocarcinoma
SEBACEOUS/ECCRINE ADENOCA.	841	8410/3	Sebaceous adenocarcinoma
		8413/3	Eccrine adenocarcinoma
CERUMINOUS ADENOCARCINOMA	842	8420/3	Ceruminous adenocarcinoma
MUCINOUS ADENOCARCINOMA	848	8480/3	Mucinous adenocarcinoma
		8481/3	Mucin-producing adenocarcinoma
		8482/3	Mucinous adenocarcinoma, endocervical type
PAGET DISEASE, EXTRAMAMMARY	854	8542/3	Paget disease, extramammary
ADENOSQUAMOUS CARCINOMA	856	8560/3	Adenosquamous carcinoma
		8562/3	Epithelial-myoepithelial carcinoma
ADENOCA. WITH METAPLASIA	857	8570/3	Adenocarcinoma with squamous metaplasia
		8571/3	Adenocarcinoma w cartilag. & oss. metaplas.
		8572/3	Adenocarcinoma with spindle cell mataplasia
		8573/3	Adenocarcinoma with apocrine metaplasia
		8574/3	Adenocarcinoma with neuroendocrine differen.
		8575/3	Metaplastic carcinoma, NOS
NEVI & MELANOMAS	872	8720/2	Melanoma in situ
		8720/3	Malignant melanoma, NOS
		8721/3	Nodular melanoma
		8722/3	Balloon cell melanoma
		8723/3	Malignant melanoma, regressing
AMELANOTIC MELANOMA	873	8730/3	Amelanotic melanoma
MAL. MEL. IN JUNCT. NEVUS	874	8740/3	Mal. melanoma in junctional nevus
		8741/2	Precancerous melanosis, NOS
		8741/3	Mal. melanoma in precan. melanosis
		8742/2	Lentigo maligna
		8742/3	Lentigo maligna melanoma
		8743/3	Superficial spreading melanoma
		8745/3	Desmoplastic melanoma, malignant
		8746/3	Mucosal lentiginous melanoma
MAL. MELAN. IN GIANT PIGMT. NEVUS	876	8761/3	Mal. melanoma in giant pigmented nevus

VULVA, NOS C519

EPITHELIOID CELL MELANOMA	877	8770/3	Mixed epithel. & spindle cell melanoma
		8771/3	Epithelioid cell melanoma
		8772/3	Spindle cell melanoma, NOS
BLUE NEVUS, MALIGNANT	878	8780/3	Blue nevus, malignant
SARCOMA, NOS	880	8800/3	Sarcoma, NOS
		8801/3	Spindle cell sarcoma
		8802/3	Giant cell sarcoma
		8803/3	Small cell sarcoma
		8804/3	Epithelioid sarcoma
		8805/3	Undifferentiated sarcoma
		8806/3	Desmoplastic small round cell tumor
FIBROMATOUS NEOPLASMS	881	8810/3	Fibrosarcoma, NOS
		8811/3	Fibromyxosarcoma
		8813/3	Fascial fibrosarcoma
		8814/3	Infantile fibrosarcoma
		8815/3	Solitary fibrous tumor, malignant
SARCOMA, NOS	882	8825/3	Myofibroblastic sarcoma
FIBROUS HISTIOCYTOMA, MAL.	883	8830/3	Fibrous histiocytoma, malignant
		8832/3	Dermatofibrosarcoma, NOS
LIPOSARCOMA NEOPLASMS	885	8850/3	Liposarcoma, NOS
		8851/3	Liposarcoma, well differentiated
		8852/3	Myxoid liposarcoma
		8853/3	Round cell liposarcoma
		8854/3	Pleomorphic liposarcoma
		8855/3	Mixed type liposarcoma
		8857/3	Fibroblastic liposarcoma
		8858/3	Dedifferentiated liposarcoma
MYOMATOUS NEOPLASMS	889	8890/3	Leiomyosarcoma, NOS
		8891/3	Epithelioid leiomyosarcoma
		8894/3	Angiomyosarcoma
		8895/3	Myosarcoma
		8896/3	Myxoid leiomyosarcoma
RHABDOMYOSARCOMA, NOS	890	8900/3	Rhabdomyosarcoma, NOS
		8901/3	Pleomorphic rhabdomyosarcoma, adult type
		8902/3	Mixed type rhabdomyosarcoma

VULVA, NOS C519

EMBRYONAL RHABDOMYOSARCOMA	891	8910/3	Embryonal rhabdomyosarcoma
		8912/3	Spindle cell rhabdomyosarcoma
MULLERIAN MIXED TUMOR	895	8950/3	Mullerian mixed tumor
		8951/3	Mesodermal mixed tumor
CARCINOSARCOMA, NOS	898	8980/3	Carcinosarcoma, NOS
		8981/3	Carcinosarcoma, embryonal type
		8982/3	Malignant myoepithelioma
MESONEPHROMA, MALIGNANT	911	9110/3	Mesonephroma, malignant
NEUROFIBROSARCOMA	954	9540/3	Malignant peripheral nerve sheath tumor
MALIGNANT LYMPHOMA, NOS	959	9590/3	Malignant lymphoma, NOS
		9591/3	Malignant lymphoma, non-Hodgkin
		9596/3	Composite Hodgkin and non-Hodgkin lymphoma
		9597/3	Primary Cutaneous follicle centre lymphoma
HODGKIN LYMPHOMA	965	9650/3	Hodgkin lymphoma, NOS
		9651/3	Hodgkin lymphoma, lymphocyte-rich
		9652/3	Hodgkin lymphoma, mixed cellularity, NOS
		9653/3	Hodgkin lymphoma, lymphocytic deplet., NOS
		9654/3	Hodgkin lymph., lymphocyt. deplet., diffuse fibrosis
		9655/3	Hodgkin lymphoma, lymphocyt. deplet., reticular
		9659/3	Hodgkin lymph., nodular lymphocyte predom.
HODGKIN LYMPHOMA, NOD. SCLER.	966	9661/3	Hodgkin granuloma [obs]
		9662/3	Hodgkin sarcoma [obs]
		9663/3	Hodgkin lymphoma, nodular sclerosis, NOS
		9664/3	Hodgkin lymphoma, nod. scler., cellular phase
		9665/3	Hodgkin lymphoma, nod. scler., grade 1
		9667/3	Hodgkin lymphoma, nod. scler., grade 2
ML, SMALL B-CELL LYMPHOCYTIC	967	9670/3	ML, small B lymphocytic, NOS
		9671/3	ML, lymphoplasmacytic
		9673/3	Mantle cell lymphoma
		9675/3	ML, mixed sm. and lg. cell, diffuse
ML, LARGE B-CELL, DIFFUSE	968	9680/3	ML, large B-cell, diffuse
		9684/3	ML, large B-cell, diffuse, immunoblastic, NOS
		9687/3	Burkitt lymphoma, NOS
		9688/3	T-cell histiocyte rich large B-cell lymphoma

VULVA, NOS C519

FOLLIC. & MARGINAL LYMPH, NOS	969	9690/3	Follicular lymphoma, NOS
		9691/3	Follicular lymphoma, grade 2
		9695/3	Follicular lymphoma, grade 1
		9698/3	Follicular lymphoma, grade 3
		9699/3	Marginal zone B-cell lymphoma, NOS
T-CELL LYMPHOMAS	970	9700/3	Mycosis fungoides
		9701/3	Sezary syndrome
		9702/3	Mature T-cell lymphoma, NOS
		9705/3	Angioimmunoblastic T-cell lymphoma
		9708/3	Subcutaneous panniculitis-like T-cell lymphoma
		9709/3	Cutaneous T-cell lymphoma, NOS
OTHER SPEC. NON-HODGKIN LYMPHOMA	971	9712/3	Intravascular large B-cell lymphoma
		9714/3	Anaplastic large cell lymphoma, T-cell and Null cell type
		9718/3	Primary cutan. CD30+ T-cell lymphoprolif. disorder
		9719/3	NK/T-cell lymphoma, nasal and nasal-type
PRECURS. CELL LYMPHOBLASTIC LYMPH.	972	9724/3	SystemicEBV pos. T-cell lymphoproliferative disease of childhood
		9725/3	Hydroa vacciniforme-like lymphoma
		9726/3	Primary Cutaneous gamma-delta T-cell lymphoma
		9727/3	Precursor cell lymphoblastic lymphoma, NOS
		9728/3	Precursor B-cell lymphoblastic lymphoma
		9729/3	Precursor T-cell lymphoblastic lymphoma
PLASMA CELL TUMORS	973	9731/3	Plasmacytoma, NOS
		9734/3	Plasmacytoma, extramedullary
		9735/3	Plasmablastic lymphoma
		9737/3	ALK positive large B-cell lymphoma
		9738/3	Lrg B-cell lymphoma in HHV8-assoc. multicentric Castleman DZ
MAST CELL TUMORS	974	9740/3	Mast cell sarcoma
		9741/3	Malignant mastocytosis
NEOPLASMS OF HISTIOCYTES AND ACCESSORY LYMPHOID CELLS	975	9750/3	Malignant histiocytosis
		9751/3	Langerhans cell histiocytosis, NOS
		9754/3	Langerhans cell histiocytosis, disseminated
		9755/3	Histiocytic sarcoma
		9756/3	Langerhans cell sarcoma
		9757/3	Interdigitating dendritic cell sarcoma
		9758/3	Follicular dendritic cell sarcoma
		9759/3	Fibroblastic reticular cell tumor

VULVA, NOS C519

PRECURSOR LYMPHOID NEOPLASMS	981	9811/3	B lymphoblastic leukemia/lymphoma, NOS
		9812/3	Leukemia/lymphoma with t(9;22)(q34;q11.2);BCR-ABL1
		9813/3	Leukemia/lymphoma with t(v;11q23);MLL rearranged
		9814/3	Leukemia/lymphoma with t(12;21)(p13;q22);TEL-AML1(ETV6-RUNX1)
		9815/3	B lymphoblastic leukemia/lymphoma with hyperdiploidy
		9816/3	Leukemia/lymphoma with hypodiploidy (hypodiploid ALL)
		9817/3	B lymphblastic leukemia/lymphoma with t(5;14)(q31;q32);IL3-IGH
		9818/3	Leukemia/lymphoma with t(1;19)(q23;p13.3); E2A PBX1 (TCF3 PBX1)
LYMPHOID LEUKEMIA, NOS	982	9823/3	Chronic lymphocytic leukemia/small lymphocytic lymphoma
PROLYMPH/PRECURS LEUKEMIA	983	9831/3	T-cell large granular lymphocytic leukemia
		9837/3	T lymphoblastic leukemia/lymphoma
CHRONIC MYELOPROLIFERATIVE DIS.	996	9965/3	Myeloid and lymphoid neoplasms with PDGFRB rearrangement
		9967/3	Myeloid and lymphoid neoplasm with FGFR1 abnormalities
MYELOPLASTIC/MYELOPROLIFERATIVE NEOPLASMS	997	9971/3	Polymorphic PTLD
		9975/3	Myelodysplastic/Myeloproliferative neoplasm, unclassifiable

CERVIX UTERI C530-C531,C538-C539

NEOPLASM	800	8000/3	Neoplasm, malignant
		8001/3	Tumor cells, malignant
		8002/3	Malignant tumor, small cell type
		8003/3	Malignant tumor, giant cell type
		8004/3	Malignant tumor, spindle cell type
		8005/3	Malignant tumor, clear cell type
CARCINOMA, NOS	801	8010/2	Carcinoma in situ, NOS
		8010/3	Carcinoma, NOS
		8011/3	Epithelioma, malignant
		8012/3	Large cell carcinoma, NOS
		8013/3	Large cell neuroendocrine carcinoma
		8014/3	Large cell carcinoma with rhabdoid phenotype
		8015/3	Glassy cell carcinoma
CARCINOMA, UNDIFF., NOS	802	8020/3	Carcinoma, undifferentiated type, NOS
		8021/3	Carcinoma, anaplastic type, NOS
		8022/3	Pleomorphic carcinoma
GIANT & SPINDLE CELL CARCINOMA	803	8030/3	Giant cell and spindle cell carcinoma
		8031/3	Giant cell carcinoma
		8032/3	Spindle cell carcinoma
		8033/3	Pseudosarcomatous carcinoma
		8034/3	Polygonal cell carcinoma
		8035/3	Carcinoma with osteoclast-like giant cells
SMALL CELL CARCINOMA, NOS	804	8041/3	Small cell carcinoma, NOS
		8043/3	Small cell carcinoma, fusiform cell
PAPILLARY CARCINOMA, NOS	805	8050/2	Papillary carcinoma in situ
		8050/3	Papillary carcinoma, NOS
		8051/3	Verrucous carcinoma, NOS
		8052/2	Papillary squamous cell carcinoma, non-invasive
		8052/3	Papillary squamous cell carcinoma
SQUAMOUS CELL CARCINOMA, NOS	807	8070/2	Squamous cell carcinoma in situ, NOS
		8070/3	Squamous cell carcinoma, NOS
		8071/2	Sq. cell carcinoma, keratinizing, NOS, in situ
		8071/3	Sq. cell carcinoma, keratinizing, NOS
		8072/2	Sq. cell carcinoma, lg. cell, non-ker., in situ
		8072/3	Sq. cell carcinoma, lg. cell, non-ker.
		8073/3	Sq. cell carcinoma, sm. cell, non-ker.
		8074/3	Sq. cell carcinoma, spindle cell
		8075/3	Squamous cell carcinoma, adenoid
		8076/2	Sq. cell carc. in situ with question. stromal invas.
		8076/3	Sq. cell carcinoma, micro-invasive
		8077/2	Squamous intraepithelial neoplasia, grade III

CERVIX UTERI C530-C531,C538-C539

SQUAMOUS CELL CARCINOMA, NOS	807	8078/3	Squamous cell carcinoma with horn formation
LYMPHOEPITHELIAL CARCINOMA	808	8081/2	Bowen disease
		8082/3	Lymphoepithelial carcinoma
		8083/3	Basaloid squamous cell carcinoma
		8084/3	Squamous cell carcinoma, clear cell type
BASAL CELL CARCINOMA, NOS	809	8098/3	Adenoid basal cell carcinoma
TRANSITIONAL CELL CARCINOMA, NOS	812	8120/2	Transitional cell carcinoma in situ
		8120/3	Transitional cell carcinoma, NOS
		8121/3	Schneiderian carcinoma
		8122/3	Trans. cell carcinoma, spindle cell
		8123/3	Basaloid carcinoma
		8124/3	Cloacogenic carcinoma
ADENOCARCINOMA, NOS	814	8140/2	Adenocarcinoma in situ
		8140/3	Adenocarcinoma, NOS
		8141/3	Scirrhous adenocarcinoma
		8143/3	Superficial spreading adenocarcinoma
		8147/3	Basal cell adenocarcinoma
ADENOID CYSTIC & CRIBRIFORM CA.	820	8200/3	Adenoid cystic carcinoma
		8201/2	Cribriform carcinoma in situ
		8201/3	Cribriform carcinoma
BRONCHIOLO-ALVEOLAR ADENOCA.	825	8255/3	Adenocarcinoma with mixed subtypes
PAPILLARY ADENOCARCINOMA, NOS	826	8260/3	Papillary adenocarcinoma, NOS
		8261/2	Adenocarcinoma in situ in villous adenoma
		8261/3	Adenocarcinoma in villous adenoma
		8262/3	Villous adenocarcinoma
		8263/2	Adenocarcinoma in situ in tubulovillous adenoma
		8263/3	Adenocarcinoma in tubulovillous adenoma
CLEAR CELL ADENOCARCINOMA, NOS	831	8310/3	Clear cell adenocarcinoma, NOS
GRANULAR CELL CARCINOMA	832	8323/3	Mixed cell adenocarcinoma
ENDOMETRIOID ADENOCARCINOMA	838	8384/3	Adenocarcinoma, endocervical type
MUCOEPIDERMOID CARCINOMA	843	8430/3	Mucoepidermoid carcinoma

CERVIX UTERI C530-C531,C538-C539

MUCINOUS ADENOCARCINOMA	848	8480/3	Mucinous adenocarcinoma
		8481/3	Mucin-producing adenocarcinoma
		8482/3	Mucinous adenocarcinoma, endocervical type
ADENOSQUAMOUS CARCINOMA	856	8560/3	Adenosquamous carcinoma
		8562/3	Epithelial-myoepithelial carcinoma
ADENOCA. WITH METAPLASIA	857	8570/3	Adenocarcinoma with squamous metaplasia
		8571/3	Adenocarcinoma w cartilag. & oss. metaplas.
		8572/3	Adenocarcinoma with spindle cell mataplasia
		8573/3	Adenocarcinoma with apocrine metaplasia
		8574/3	Adenocarcinoma with neuroendocrine differen.
		8575/3	Metaplastic carcinoma, NOS
SARCOMA, NOS	880	8800/3	Sarcoma, NOS
		8801/3	Spindle cell sarcoma
		8802/3	Giant cell sarcoma
		8803/3	Small cell sarcoma
		8804/3	Epithelioid sarcoma
		8805/3	Undifferentiated sarcoma
		8806/3	Desmoplastic small round cell tumor
FIBROMATOUS NEOPLASMS	881	8810/3	Fibrosarcoma, NOS
		8811/3	Fibromyxosarcoma
		8813/3	Fascial fibrosarcoma
		8814/3	Infantile fibrosarcoma
		8815/3	Solitary fibrous tumor, malignant
SARCOMA, NOS	882	8825/3	Myofibroblastic sarcoma
MYOMATOUS NEOPLASMS	889	8890/3	Leiomyosarcoma, NOS
		8891/3	Epithelioid leiomyosarcoma
		8894/3	Angiomyosarcoma
		8895/3	Myosarcoma
		8896/3	Myxoid leiomyosarcoma
RHABDOMYOSARCOMA, NOS	890	8900/3	Rhabdomyosarcoma, NOS
		8901/3	Pleomorphic rhabdomyosarcoma, adult type
		8902/3	Mixed type rhabdomyosarcoma
EMBRYONAL RHABDOMYOSARCOMA	891	8910/3	Embryonal rhabdomyosarcoma
		8912/3	Spindle cell rhabdomyosarcoma
MULLERIAN MIXED TUMOR	895	8950/3	Mullerian mixed tumor
		8951/3	Mesodermal mixed tumor

CERVIX UTERI C530-C531,C538-C539

CARCINOSARCOMA, NOS	898	8980/3	Carcinosarcoma, NOS
		8981/3	Carcinosarcoma, embryonal type
		8982/3	Malignant myoepithelioma
MESENCHYMOMA, MALIGNANT	899	8990/3	Mesenchymoma, malignant
		8991/3	Embryonal sarcoma
MESONEPHROMA, MALIGNANT	911	9110/3	Mesonephroma, malignant
MALIGNANT LYMPHOMA, NOS	959	9590/3	Malignant lymphoma, NOS
		9591/3	Malignant lymphoma, non-Hodgkin
		9596/3	Composite Hodgkin and non-Hodgkin lymphoma
HODGKIN LYMPHOMA	965	9650/3	Hodgkin lymphoma, NOS
		9651/3	Hodgkin lymphoma, lymphocyte-rich
		9652/3	Hodgkin lymphoma, mixed cellularity, NOS
		9653/3	Hodgkin lymphoma, lymphocytic deplet., NOS
		9654/3	Hodgkin lymph., lymphocyt. deplet., diffuse fibrosis
		9655/3	Hodgkin lymphoma, lymphocyt. deplet., reticular
		9659/3	Hodgkin lymph., nodular lymphocyte predom.
HODGKIN LYMPHOMA, NOD. SCLER.	966	9661/3	Hodgkin granuloma [obs]
		9662/3	Hodgkin sarcoma [obs]
		9663/3	Hodgkin lymphoma, nodular sclerosis, NOS
		9664/3	Hodgkin lymphoma, nod. scler., cellular phase
		9665/3	Hodgkin lymphoma, nod. scler., grade 1
		9667/3	Hodgkin lymphoma, nod. scler., grade 2
ML, SMALL B-CELL LYMPHOCYTIC	967	9670/3	ML, small B lymphocytic, NOS
		9671/3	ML, lymphoplasmacytic
		9673/3	Mantle cell lymphoma
		9675/3	ML, mixed sm. and lg. cell, diffuse
ML, LARGE B-CELL, DIFFUSE	968	9680/3	ML, large B-cell, diffuse
		9684/3	ML, large B-cell, diffuse, immunoblastic, NOS
		9687/3	Burkitt lymphoma, NOS
		9688/3	T-cell histiocyte rich large B-cell lymphoma
FOLLIC. & MARGINAL LYMPH, NOS	969	9690/3	Follicular lymphoma, NOS
		9691/3	Follicular lymphoma, grade 2
		9695/3	Follicular lymphoma, grade 1
		9698/3	Follicular lymphoma, grade 3
		9699/3	Marginal zone B-cell lymphoma, NOS

CERVIX UTERI C530-C531,C538-C539

T-CELL LYMPHOMAS	970	9701/3	Sezary syndrome
		9702/3	Mature T-cell lymphoma, NOS
		9705/3	Angioimmunoblastic T-cell lymphoma
OTHER SPEC. NON-HODGKIN LYMPHOMA	971	9712/3	Intravascular large B-cell lymphoma
		9714/3	Anaplastic large cell lymphoma, T-cell and Null cell type
		9719/3	NK/T-cell lymphoma, nasal and nasal-type
PRECURS. CELL LYMPHOBLASTIC LYMPH.	972	9724/3	SystemicEBV pos. T-cell lymphoproliferative disease of childhood
		9727/3	Precursor cell lymphoblastic lymphoma, NOS
		9728/3	Precursor B-cell lymphoblastic lymphoma
		9729/3	Precursor T-cell lymphoblastic lymphoma
PLASMA CELL TUMORS	973	9731/3	Plasmacytoma, NOS
		9734/3	Plasmacytoma, extramedullary
		9735/3	Plasmablastic lymphoma
		9737/3	ALK positive large B-cell lymphoma
		9738/3	Lrg B-cell lymphoma in HHV8-assoc. multicentric Castleman DZ
MAST CELL TUMORS	974	9740/3	Mast cell sarcoma
		9741/3	Malignant mastocytosis
NEOPLASMS OF HISTIOCYTES AND ACCESSORY LYMPHOID CELLS	975	9750/3	Malignant histiocytosis
		9751/3	Langerhans cell histiocytosis, NOS
		9754/3	Langerhans cell histiocytosis, disseminated
		9755/3	Histiocytic sarcoma
		9756/3	Langerhans cell sarcoma
		9757/3	Interdigitating dendritic cell sarcoma
		9758/3	Follicular dendritic cell sarcoma
		9759/3	Fibroblastic reticular cell tumor
PRECURSOR LYMPHOID NEOPLASMS	981	9811/3	B lymphoblastic leukemia/lymphoma, NOS
		9812/3	Leukemia/lymphoma with t(9;22)(q34;q11.2);BCR-ABL1
		9813/3	Leukemia/lymphoma with t(v;11q23);MLL rearranged
		9814/3	Leukemia/lymphoma with t(12;21)(p13;q22);TEL-AML1(ETV6-RUNX1)
		9815/3	B lymphoblastic leukemia/lymphoma with hyperdiploidy
		9816/3	Leukemia/lymphoma with hypodiploidy (hypodiploid ALL)
		9817/3	B lymphblastic leukemia/lymphoma with t(5;14)(q31;q32);IL3-IGH
		9818/3	Leukemia/lymphoma with t(1;19)(q23;p13.3); E2A PBX1 (TCF3 PBX1)
LYMPHOID LEUKEMIA, NOS	982	9823/3	Chronic lymphocytic leukemia/small lymphocytic lymphoma
PROLYMPH/PRECURS LEUKEMIA	983	9831/3	T-cell large granular lymphocytic leukemia
		9837/3	T lymphoblastic leukemia/lymphoma

CERVIX UTERI C530-C531,C538-C539

CHRONIC MYELOPROLIFERATIVE DIS.	996	9965/3	Myeloid and lymphoid neoplasms with PDGFRB rearrangement
		9967/3	Myeloid and lymphoid neoplasm with FGFR1 abnormalities
MYELOPLASTIC/MYELOPROLIFERATIVE NEOPLASMS	997	9971/3	Polymorphic PTLD
		9975/3	Myelodysplastic/Myeloproliferative neoplasm, unclassifiable

CORPUS UTERI C540-C543,C548-C549

NEOPLASM	800	8000/3	Neoplasm, malignant
		8001/3	Tumor cells, malignant
		8002/3	Malignant tumor, small cell type
		8003/3	Malignant tumor, giant cell type
		8004/3	Malignant tumor, spindle cell type
		8005/3	Malignant tumor, clear cell type
CARCINOMA, NOS	801	8010/2	Carcinoma in situ, NOS
		8010/3	Carcinoma, NOS
		8011/3	Epithelioma, malignant
		8012/3	Large cell carcinoma, NOS
		8013/3	Large cell neuroendocrine carcinoma
		8014/3	Large cell carcinoma with rhabdoid phenotype
		8015/3	Glassy cell carcinoma
CARCINOMA, UNDIFF., NOS	802	8020/3	Carcinoma, undifferentiated type, NOS
		8021/3	Carcinoma, anaplastic type, NOS
		8022/3	Pleomorphic carcinoma
GIANT & SPINDLE CELL CARCINOMA	803	8030/3	Giant cell and spindle cell carcinoma
		8031/3	Giant cell carcinoma
		8032/3	Spindle cell carcinoma
		8033/3	Pseudosarcomatous carcinoma
		8034/3	Polygonal cell carcinoma
		8035/3	Carcinoma with osteoclast-like giant cells
SMALL CELL CARCINOMA, NOS	804	8041/3	Small cell carcinoma, NOS
		8043/3	Small cell carcinoma, fusiform cell
PAPILLARY CARCINOMA, NOS	805	8050/2	Papillary carcinoma in situ
		8050/3	Papillary carcinoma, NOS
		8051/3	Verrucous carcinoma, NOS
		8052/2	Papillary squamous cell carcinoma, non-invasive
		8052/3	Papillary squamous cell carcinoma
SQUAMOUS CELL CARCINOMA, NOS	807	8070/2	Squamous cell carcinoma in situ, NOS
		8070/3	Squamous cell carcinoma, NOS
		8071/3	Sq. cell carcinoma, keratinizing, NOS
		8072/3	Sq. cell carcinoma, lg. cell, non-ker.
		8073/3	Sq. cell carcinoma, sm. cell, non-ker.
		8074/3	Sq. cell carcinoma, spindle cell
		8075/3	Squamous cell carcinoma, adenoid
		8076/2	Sq. cell carc. in situ with question. stromal invas.
		8076/3	Sq. cell carcinoma, micro-invasive
		8078/3	Squamous cell carcinoma with horn formation

CORPUS UTERI C540-C543,C548-C549

TRANSITIONAL CELL CARCINOMA, NOS	812	8120/2	Transitional cell carcinoma in situ
		8120/3	Transitional cell carcinoma, NOS
		8121/3	Schneiderian carcinoma
		8122/3	Trans. cell carcinoma, spindle cell
		8123/3	Basaloid carcinoma
		8124/3	Cloacogenic carcinoma
PAPILLARY TRANS. CELL CARCINOMA	813	8130/2	Papillary trans. cell carcinoma, non-invasive
		8130/3	Papillary trans. cell carcinoma
		8131/3	Transitional cell carcinoma, micropapillary
ADENOCARCINOMA, NOS	814	8140/2	Adenocarcinoma in situ
		8140/3	Adenocarcinoma, NOS
		8141/3	Scirrhous adenocarcinoma
		8143/3	Superficial spreading adenocarcinoma
		8147/3	Basal cell adenocarcinoma
ADENOCA. IN ADENOMA. POLYP	821	8210/2	Adenocarcinoma in situ in adenomatous polyp
		8210/3	Adenocarcinoma in adenomatous polyp
		8211/3	Tubular adenocarcinoma
SOLID CARCINOMA, NOS	823	8230/2	Duct carcinoma in situ, solid type
		8230/3	Solid carcinoma, NOS
		8231/3	Carcinoma simplex
BRONCHIOLO-ALVEOLAR ADENOCA.	825	8255/3	Adenocarcinoma with mixed subtypes
PAPILLARY ADENOCARCINOMA, NOS	826	8260/3	Papillary adenocarcinoma, NOS
		8261/2	Adenocarcinoma in situ in villous adenoma
		8261/3	Adenocarcinoma in villous adenoma
		8262/3	Villous adenocarcinoma
		8263/2	Adenocarcinoma in situ in tubulovillous adenoma
		8263/3	Adenocarcinoma in tubulovillous adenoma
CLEAR CELL ADENOCARCINOMA, NOS	831	8310/3	Clear cell adenocarcinoma, NOS
GRANULAR CELL CARCINOMA	832	8320/3	Granular cell carcinoma
		8323/3	Mixed cell adenocarcinoma
ENDOMETRIOID ADENOCARCINOMA	838	8380/3	Endometrioid carcinoma
		8381/3	Endometrioid adenofibroma, malignant
		8382/3	Endometrioid adenocarcinoma, secretory variant
		8383/3	Endometrioid adenocarcinoma, ciliated cell variant

CORPUS UTERI C540-C543,C548-C549

CYSTADENOCARCINOMA, NOS	844	8440/3	Cystadenocarcinoma, NOS
		8441/2	Serous tubal intraepithelial carcinoma
		8441/3	Serous cystadenocarcinoma, NOS
PAPILLARY SEROUS CYSTADENOCA	846	8460/3	Papillary serous cystadenocarcinoma
		8461/3	Serous surface papillary carcinoma
MUCINOUS ADENOCARCINOMA	848	8480/3	Mucinous adenocarcinoma
		8481/3	Mucin-producing adenocarcinoma
		8482/3	Mucinous adenocarcinoma, endocervical type
MEDULLARY CARCINOMA, NOS	851	8510/3	Medullary carcinoma, NOS
ADENOSQUAMOUS CARCINOMA	856	8560/3	Adenosquamous carcinoma
		8562/3	Epithelial-myoepithelial carcinoma
ADENOCA. WITH METAPLASIA	857	8570/3	Adenocarcinoma with squamous metaplasia
		8571/3	Adenocarcinoma w cartilag. & oss. metaplas.
		8572/3	Adenocarcinoma with spindle cell mataplasia
		8573/3	Adenocarcinoma with apocrine metaplasia
		8574/3	Adenocarcinoma with neuroendocrine differen.
		8575/3	Metaplastic carcinoma, NOS
		8576/3	Hepatoid adenocarcinoma
SARCOMA, NOS	880	8800/3	Sarcoma, NOS
		8801/3	Spindle cell sarcoma
		8802/3	Giant cell sarcoma
		8803/3	Small cell sarcoma
		8804/3	Epithelioid sarcoma
		8805/3	Undifferentiated sarcoma
		8806/3	Desmoplastic small round cell tumor
FIBROMATOUS NEOPLASMS	881	8810/3	Fibrosarcoma, NOS
		8811/3	Fibromyxosarcoma
		8813/3	Fascial fibrosarcoma
		8814/3	Infantile fibrosarcoma
		8815/3	Solitary fibrous tumor, malignant
SARCOMA, NOS	882	8825/3	Myofibroblastic sarcoma
MYOMATOUS NEOPLASMS	889	8890/3	Leiomyosarcoma, NOS
		8891/3	Epithelioid leiomyosarcoma
		8894/3	Angiomyosarcoma
		8895/3	Myosarcoma
		8896/3	Myxoid leiomyosarcoma

CORPUS UTERI C540-C543,C548-C549

RHABDOMYOSARCOMA, NOS	890	8900/3	Rhabdomyosarcoma, NOS
		8901/3	Pleomorphic rhabdomyosarcoma, adult type
		8902/3	Mixed type rhabdomyosarcoma
EMBRYONAL RHABDOMYOSARCOMA	891	8910/3	Embryonal rhabdomyosarcoma
		8912/3	Spindle cell rhabdomyosarcoma
STROMAL SARCOMA	893	8930/3	Endometrial stromal sarcoma
		8931/3	Endometrial stromal sarcoma, low grade
		8933/3	Adenosarcoma
		8934/3	Carcinofibroma
MULLERIAN MIXED TUMOR	895	8950/3	Mullerian mixed tumor
		8951/3	Mesodermal mixed tumor
CARCINOSARCOMA, NOS	898	8980/3	Carcinosarcoma, NOS
		8981/3	Carcinosarcoma, embryonal type
		8982/3	Malignant myoepithelioma
MESENCHYMOMA, MALIGNANT	899	8990/3	Mesenchymoma, malignant
		8991/3	Embryonal sarcoma
MESONEPHROMA, MALIGNANT	911	9110/3	Mesonephroma, malignant
MALIGNANT LYMPHOMA, NOS	959	9590/3	Malignant lymphoma, NOS
		9591/3	Malignant lymphoma, non-Hodgkin
		9596/3	Composite Hodgkin and non-Hodgkin lymphoma
HODGKIN LYMPHOMA	965	9650/3	Hodgkin lymphoma, NOS
		9651/3	Hodgkin lymphoma, lymphocyte-rich
		9652/3	Hodgkin lymphoma, mixed cellularity, NOS
		9653/3	Hodgkin lymphoma, lymphocytic deplet., NOS
		9654/3	Hodgkin lymph., lymphocyt. deplet., diffuse fibrosis
		9655/3	Hodgkin lymphoma, lymphocyt. deplet., reticular
		9659/3	Hodgkin lymph., nodular lymphocyte predom.
HODGKIN LYMPHOMA, NOD. SCLER.	966	9661/3	Hodgkin granuloma [obs]
		9662/3	Hodgkin sarcoma [obs]
		9663/3	Hodgkin lymphoma, nodular sclerosis, NOS
		9664/3	Hodgkin lymphoma, nod. scler., cellular phase
		9665/3	Hodgkin lymphoma, nod. scler., grade 1
		9667/3	Hodgkin lymphoma, nod. scler., grade 2

CORPUS UTERI C540-C543,C548-C549

ML, SMALL B-CELL LYMPHOCYTIC	967	9670/3	ML, small B lymphocytic, NOS
		9671/3	ML, lymphoplasmacytic
		9673/3	Mantle cell lymphoma
		9675/3	ML, mixed sm. and lg. cell, diffuse
ML, LARGE B-CELL, DIFFUSE	968	9680/3	ML, large B-cell, diffuse
		9684/3	ML, large B-cell, diffuse, immunoblastic, NOS
		9687/3	Burkitt lymphoma, NOS
		9688/3	T-cell histiocyte rich large B-cell lymphoma
FOLLIC. & MARGINAL LYMPH, NOS	969	9690/3	Follicular lymphoma, NOS
		9691/3	Follicular lymphoma, grade 2
		9695/3	Follicular lymphoma, grade 1
		9698/3	Follicular lymphoma, grade 3
		9699/3	Marginal zone B-cell lymphoma, NOS
T-CELL LYMPHOMAS	970	9701/3	Sezary syndrome
		9702/3	Mature T-cell lymphoma, NOS
		9705/3	Angioimmunoblastic T-cell lymphoma
OTHER SPEC. NON-HODGKIN LYMPHOMA	971	9712/3	Intravascular large B-cell lymphoma
		9714/3	Anaplastic large cell lymphoma, T-cell and Null cell type
		9719/3	NK/T-cell lymphoma, nasal and nasal-type
PRECURS. CELL LYMPHOBLASTIC LYMPH.	972	9724/3	SystemicEBV pos. T-cell lymphoproliferative disease of childhood
		9727/3	Precursor cell lymphoblastic lymphoma, NOS
		9728/3	Precursor B-cell lymphoblastic lymphoma
		9729/3	Precursor T-cell lymphoblastic lymphoma
PLASMA CELL TUMORS	973	9731/3	Plasmacytoma, NOS
		9734/3	Plasmacytoma, extramedullary
MAST CELL TUMORS	974	9740/3	Mast cell sarcoma
		9741/3	Malignant mastocytosis
NEOPLASMS OF HISTIOCYTES AND ACCESSORY LYMPHOID CELLS	975	9750/3	Malignant histiocytosis
		9751/3	Langerhans cell histiocytosis, NOS
		9754/3	Langerhans cell histiocytosis, disseminated
		9755/3	Histiocytic sarcoma
		9756/3	Langerhans cell sarcoma
		9757/3	Interdigitating dendritic cell sarcoma
		9758/3	Follicular dendritic cell sarcoma
		9759/3	Fibroblastic reticular cell tumor

CORPUS UTERI C540-C543,C548-C549

PRECURSOR LYMPHOID NEOPLASMS	981	9811/3	B lymphoblastic leukemia/lymphoma, NOS
		9812/3	Leukemia/lymphoma with t(9;22)(q34;q11.2);BCR-ABL1
		9813/3	Leukemia/lymphoma with t(v;11q23);MLL rearranged
		9814/3	Leukemia/lymphoma with t(12;21)(p13;q22);TEL-AML1(ETV6-RUNX1)
		9815/3	B lymphoblastic leukemia/lymphoma with hyperdiploidy
		9816/3	Leukemia/lymphoma with hypodiploidy (hypodiploid ALL)
		9817/3	B lymphblastic leukemia/lymphoma with t(5;14)(q31;q32);IL3-IGH
		9818/3	Leukemia/lymphoma with t(1;19)(q23;p13.3); E2A PBX1 (TCF3 PBX1)
LYMPHOID LEUKEMIA, NOS	982	9823/3	Chronic lymphocytic leukemia/small lymphocytic lymphoma
PROLYMPH/PRECURS LEUKEMIA	983	9831/3	T-cell large granular lymphocytic leukemia
		9837/3	T lymphoblastic leukemia/lymphoma
CHRONIC MYELOPROLIFERATIVE DIS.	996	9965/3	Myeloid and lymphoid neoplasms with PDGFRB rearrangement
		9967/3	Myeloid and lymphoid neoplasm with FGFR1 abnormalities
MYELOPLASTIC/MYELOPROLIFERATIVE NEOPLASMS	997	9971/3	Polymorphic PTLD
		9975/3	Myelodysplastic/Myeloproliferative neoplasm, unclassifiable

UTERUS, NOS C559

NEOPLASM	800	8000/3	Neoplasm, malignant
		8001/3	Tumor cells, malignant
		8002/3	Malignant tumor, small cell type
		8003/3	Malignant tumor, giant cell type
		8004/3	Malignant tumor, spindle cell type
		8005/3	Malignant tumor, clear cell type
CARCINOMA, NOS	801	8010/2	Carcinoma in situ, NOS
		8010/3	Carcinoma, NOS
		8011/3	Epithelioma, malignant
		8012/3	Large cell carcinoma, NOS
		8013/3	Large cell neuroendocrine carcinoma
		8014/3	Large cell carcinoma with rhabdoid phenotype
		8015/3	Glassy cell carcinoma
CARCINOMA, UNDIFF., NOS	802	8020/3	Carcinoma, undifferentiated type, NOS
		8021/3	Carcinoma, anaplastic type, NOS
		8022/3	Pleomorphic carcinoma
GIANT & SPINDLE CELL CARCINOMA	803	8030/3	Giant cell and spindle cell carcinoma
		8031/3	Giant cell carcinoma
		8032/3	Spindle cell carcinoma
		8033/3	Pseudosarcomatous carcinoma
		8034/3	Polygonal cell carcinoma
		8035/3	Carcinoma with osteoclast-like giant cells
SMALL CELL CARCINOMA, NOS	804	8041/3	Small cell carcinoma, NOS
		8043/3	Small cell carcinoma, fusiform cell
PAPILLARY CARCINOMA, NOS	805	8050/2	Papillary carcinoma in situ
		8050/3	Papillary carcinoma, NOS
		8051/3	Verrucous carcinoma, NOS
		8052/2	Papillary squamous cell carcinoma, non-invasive
		8052/3	Papillary squamous cell carcinoma
SQUAMOUS CELL CARCINOMA, NOS	807	8070/2	Squamous cell carcinoma in situ, NOS
		8070/3	Squamous cell carcinoma, NOS
		8071/3	Sq. cell carcinoma, keratinizing, NOS
		8072/3	Sq. cell carcinoma, lg. cell, non-ker.
		8073/3	Sq. cell carcinoma, sm. cell, non-ker.
		8074/3	Sq. cell carcinoma, spindle cell
		8075/3	Squamous cell carcinoma, adenoid
		8076/2	Sq. cell carc. in situ with question. stromal invas.
		8076/3	Sq. cell carcinoma, micro-invasive
		8078/3	Squamous cell carcinoma with horn formation

UTERUS, NOS C559

LYMPHOEPITHELIAL CARCINOMA	808	8082/3	Lymphoepithelial carcinoma
		8083/3	Basaloid squamous cell carcinoma
		8084/3	Squamous cell carcinoma, clear cell type
TRANSITIONAL CELL CARCINOMA, NOS	812	8120/2	Transitional cell carcinoma in situ
		8120/3	Transitional cell carcinoma, NOS
		8121/3	Schneiderian carcinoma
		8122/3	Trans. cell carcinoma, spindle cell
		8123/3	Basaloid carcinoma
		8124/3	Cloacogenic carcinoma
PAPILLARY TRANS. CELL CARCINOMA	813	8130/2	Papillary trans. cell carcinoma, non-invasive
		8130/3	Papillary trans. cell carcinoma
		8131/3	Transitional cell carcinoma, micropapillary
ADENOCARCINOMA, NOS	814	8140/2	Adenocarcinoma in situ
		8140/3	Adenocarcinoma, NOS
		8141/3	Scirrhous adenocarcinoma
		8143/3	Superficial spreading adenocarcinoma
		8147/3	Basal cell adenocarcinoma
ADENOID CYSTIC & CRIBRIFORM CA.	820	8200/3	Adenoid cystic carcinoma
		8201/2	Cribriform carcinoma in situ
		8201/3	Cribriform carcinoma
ADENOCA. IN ADENOMA. POLYP	821	8210/2	Adenocarcinoma in situ in adenomatous polyp
		8210/3	Adenocarcinoma in adenomatous polyp
		8211/3	Tubular adenocarcinoma
SOLID CARCINOMA, NOS	823	8230/2	Duct carcinoma in situ, solid type
		8230/3	Solid carcinoma, NOS
		8231/3	Carcinoma simplex
BRONCHIOLO-ALVEOLAR ADENOCA.	825	8255/3	Adenocarcinoma with mixed subtypes
PAPILLARY ADENOCARCINOMA, NOS	826	8260/3	Papillary adenocarcinoma, NOS
		8261/2	Adenocarcinoma in situ in villous adenoma
		8261/3	Adenocarcinoma in villous adenoma
		8262/3	Villous adenocarcinoma
		8263/2	Adenocarcinoma in situ in tubulovillous adenoma
		8263/3	Adenocarcinoma in tubulovillous adenoma
CLEAR CELL ADENOCARCINOMA, NOS	831	8310/3	Clear cell adenocarcinoma, NOS

UTERUS, NOS C559

GRANULAR CELL CARCINOMA	832	8320/3	Granular cell carcinoma
		8323/3	Mixed cell adenocarcinoma
MUCOEPIDERMOID CARCINOMA	843	8430/3	Mucoepidermoid carcinoma
CYSTADENOCARCINOMA, NOS	844	8440/3	Cystadenocarcinoma, NOS
		8441/3	Serous cystadenocarcinoma, NOS
PAPILLARY SEROUS CYSTADENOCA	846	8460/3	Papillary serous cystadenocarcinoma
MUCINOUS ADENOCARCINOMA	848	8480/3	Mucinous adenocarcinoma
		8481/3	Mucin-producing adenocarcinoma
		8482/3	Mucinous adenocarcinoma, endocervical type
MEDULLARY CARCINOMA, NOS	851	8510/3	Medullary carcinoma, NOS
ADENOSQUAMOUS CARCINOMA	856	8560/3	Adenosquamous carcinoma
		8562/3	Epithelial-myoepithelial carcinoma
ADENOCA. WITH METAPLASIA	857	8570/3	Adenocarcinoma with squamous metaplasia
		8571/3	Adenocarcinoma w cartilag. & oss. metaplas.
		8572/3	Adenocarcinoma with spindle cell mataplasia
		8573/3	Adenocarcinoma with apocrine metaplasia
		8574/3	Adenocarcinoma with neuroendocrine differen.
		8575/3	Metaplastic carcinoma, NOS
		8576/3	Hepatoid adenocarcinoma
SARCOMA, NOS	880	8800/3	Sarcoma, NOS
		8801/3	Spindle cell sarcoma
		8802/3	Giant cell sarcoma
		8803/3	Small cell sarcoma
		8804/3	Epithelioid sarcoma
		8805/3	Undifferentiated sarcoma
		8806/3	Desmoplastic small round cell tumor
FIBROMATOUS NEOPLASMS	881	8810/3	Fibrosarcoma, NOS
		8811/3	Fibromyxosarcoma
		8813/3	Fascial fibrosarcoma
		8814/3	Infantile fibrosarcoma
		8815/3	Solitary fibrous tumor, malignant
SARCOMA, NOS	882	8825/3	Myofibroblastic sarcoma

UTERUS, NOS C559

MYOMATOUS NEOPLASMS	889	8890/3	Leiomyosarcoma, NOS
		8891/3	Epithelioid leiomyosarcoma
		8894/3	Angiomyosarcoma
		8895/3	Myosarcoma
		8896/3	Myxoid leiomyosarcoma
RHABDOMYOSARCOMA, NOS	890	8900/3	Rhabdomyosarcoma, NOS
		8901/3	Pleomorphic rhabdomyosarcoma, adult type
		8902/3	Mixed type rhabdomyosarcoma
EMBRYONAL RHABDOMYOSARCOMA	891	8910/3	Embryonal rhabdomyosarcoma
		8912/3	Spindle cell rhabdomyosarcoma
STROMAL SARCOMA	893	8930/3	Endometrial stromal sarcoma
		8931/3	Endometrial stromal sarcoma, low grade
		8934/3	Carcinofibroma
		8935/3	Stromal sarcoma, NOS
MULLERIAN MIXED TUMOR	895	8950/3	Mullerian mixed tumor
		8951/3	Mesodermal mixed tumor
CARCINOSARCOMA, NOS	898	8980/3	Carcinosarcoma, NOS
		8981/3	Carcinosarcoma, embryonal type
		8982/3	Malignant myoepithelioma
MESENCHYMOMA, MALIGNANT	899	8990/3	Mesenchymoma, malignant
		8991/3	Embryonal sarcoma
MESONEPHROMA, MALIGNANT	911	9110/3	Mesonephroma, malignant
MALIGNANT LYMPHOMA, NOS	959	9590/3	Malignant lymphoma, NOS
		9591/3	Malignant lymphoma, non-Hodgkin
		9596/3	Composite Hodgkin and non-Hodgkin lymphoma
HODGKIN LYMPHOMA	965	9650/3	Hodgkin lymphoma, NOS
		9651/3	Hodgkin lymphoma, lymphocyte-rich
		9652/3	Hodgkin lymphoma, mixed cellularity, NOS
		9653/3	Hodgkin lymphoma, lymphocytic deplet., NOS
		9654/3	Hodgkin lymph., lymphocyt. deplet., diffuse fibrosis
		9655/3	Hodgkin lymphoma, lymphocyt. deplet., reticular
		9659/3	Hodgkin lymph., nodular lymphocyte predom.
HODGKIN LYMPHOMA, NOD. SCLER.	966	9661/3	Hodgkin granuloma [obs]
		9662/3	Hodgkin sarcoma [obs]
		9663/3	Hodgkin lymphoma, nodular sclerosis, NOS
		9664/3	Hodgkin lymphoma, nod. scler., cellular phase
		9665/3	Hodgkin lymphoma, nod. scler., grade 1

UTERUS, NOS C559

HODGKIN LYMPHOMA, NOD. SCLER.	966	9667/3	Hodgkin lymphoma, nod. scler., grade 2
ML, SMALL B-CELL LYMPHOCYTIC	967	9670/3	ML, small B lymphocytic, NOS
		9671/3	ML, lymphoplasmacytic
		9673/3	Mantle cell lymphoma
		9675/3	ML, mixed sm. and lg. cell, diffuse
ML, LARGE B-CELL, DIFFUSE	968	9680/3	ML, large B-cell, diffuse
		9684/3	ML, large B-cell, diffuse, immunoblastic, NOS
		9687/3	Burkitt lymphoma, NOS
		9688/3	T-cell histiocyte rich large B-cell lymphoma
FOLLIC. & MARGINAL LYMPH, NOS	969	9690/3	Follicular lymphoma, NOS
		9691/3	Follicular lymphoma, grade 2
		9695/3	Follicular lymphoma, grade 1
		9698/3	Follicular lymphoma, grade 3
		9699/3	Marginal zone B-cell lymphoma, NOS
T-CELL LYMPHOMAS	970	9701/3	Sezary syndrome
		9702/3	Mature T-cell lymphoma, NOS
		9705/3	Angioimmunoblastic T-cell lymphoma
OTHER SPEC. NON-HODGKIN LYMPHOMA	971	9712/3	Intravascular large B-cell lymphoma
		9714/3	Anaplastic large cell lymphoma, T-cell and Null cell type
		9719/3	NK/T-cell lymphoma, nasal and nasal-type
PRECURS. CELL LYMPHOBLASTIC LYMPH.	972	9724/3	SystemicEBV pos. T-cell lymphoproliferative disease of childhood
		9727/3	Precursor cell lymphoblastic lymphoma, NOS
		9728/3	Precursor B-cell lymphoblastic lymphoma
		9729/3	Precursor T-cell lymphoblastic lymphoma
PLASMA CELL TUMORS	973	9731/3	Plasmacytoma, NOS
		9734/3	Plasmacytoma, extramedullary
		9735/3	Plasmablastic lymphoma
		9737/3	ALK positive large B-cell lymphoma
		9738/3	Lrg B-cell lymphoma in HHV8-assoc. multicentric Castleman DZ
MAST CELL TUMORS	974	9740/3	Mast cell sarcoma
		9741/3	Malignant mastocytosis
NEOPLASMS OF HISTIOCYTES AND ACCESSORY LYMPHOID CELLS	975	9750/3	Malignant histiocytosis
		9751/3	Langerhans cell histiocytosis, NOS
		9754/3	Langerhans cell histiocytosis, disseminated
		9755/3	Histiocytic sarcoma
		9756/3	Langerhans cell sarcoma
		9757/3	Interdigitating dendritic cell sarcoma
		9758/3	Follicular dendritic cell sarcoma

UTERUS, NOS C559

NEOPLASMS OF HISTIOCYTES AND ACCESSORY LYMPHOID CELLS	975	9759/3	Fibroblastic reticular cell tumor
PRECURSOR LYMPHOID NEOPLASMS	981	9811/3	B lymphoblastic leukemia/lymphoma, NOS
		9812/3	Leukemia/lymphoma with t(9;22)(q34;q11.2);BCR-ABL1
		9813/3	Leukemia/lymphoma with t(v;11q23);MLL rearranged
		9814/3	Leukemia/lymphoma with t(12;21)(p13;q22);TEL-AML1(ETV6-RUNX1)
		9815/3	B lymphoblastic leukemia/lymphoma with hyperdiploidy
		9816/3	Leukemia/lymphoma with hypodiploidy (hypodiploid ALL)
		9817/3	B lymphblastic leukemia/lymphoma with t(5;14)(q31;q32);IL3-IGH
		9818/3	Leukemia/lymphoma with t(1;19)(q23;p13.3); E2A PBX1 (TCF3 PBX1)
LYMPHOID LEUKEMIA, NOS	982	9823/3	Chronic lymphocytic leukemia/small lymphocytic lymphoma
PROLYMPH/PRECURS LEUKEMIA	983	9831/3	T-cell large granular lymphocytic leukemia
		9837/3	T lymphoblastic leukemia/lymphoma
CHRONIC MYELOPROLIFERATIVE DIS.	996	9965/3	Myeloid and lymphoid neoplasms with PDGFRB rearrangement
		9967/3	Myeloid and lymphoid neoplasm with FGFR1 abnormalities
MYELOPLASTIC/MYELOPROLIFERATIVE NEOPLASMS	997	9971/3	Polymorphic PTLD
		9975/3	Myelodysplastic/Myeloproliferative neoplasm, unclassifiable

OVARY C569

NEOPLASM	800	8000/3	Neoplasm, malignant
		8001/3	Tumor cells, malignant
		8002/3	Malignant tumor, small cell type
		8003/3	Malignant tumor, giant cell type
		8004/3	Malignant tumor, spindle cell type
		8005/3	Malignant tumor, clear cell type
CARCINOMA, NOS	801	8010/2	Carcinoma in situ, NOS
		8010/3	Carcinoma, NOS
		8011/3	Epithelioma, malignant
		8012/3	Large cell carcinoma, NOS
		8013/3	Large cell neuroendocrine carcinoma
		8014/3	Large cell carcinoma with rhabdoid phenotype
		8015/3	Glassy cell carcinoma
CARCINOMA, UNDIFF., NOS	802	8020/3	Carcinoma, undifferentiated type, NOS
		8021/3	Carcinoma, anaplastic type, NOS
		8022/3	Pleomorphic carcinoma
GIANT & SPINDLE CELL CARCINOMA	803	8030/3	Giant cell and spindle cell carcinoma
		8031/3	Giant cell carcinoma
		8032/3	Spindle cell carcinoma
		8033/3	Pseudosarcomatous carcinoma
		8034/3	Polygonal cell carcinoma
		8035/3	Carcinoma with osteoclast-like giant cells
SMALL CELL CARCINOMA, NOS	804	8041/3	Small cell carcinoma, NOS
PAPILLARY CARCINOMA, NOS	805	8050/2	Papillary carcinoma in situ
		8050/3	Papillary carcinoma, NOS
		8051/3	Verrucous carcinoma, NOS
		8052/2	Papillary squamous cell carcinoma, non-invasive
		8052/3	Papillary squamous cell carcinoma
SQUAMOUS CELL CARCINOMA, NOS	807	8070/2	Squamous cell carcinoma in situ, NOS
		8070/3	Squamous cell carcinoma, NOS
		8071/3	Sq. cell carcinoma, keratinizing, NOS
		8072/3	Sq. cell carcinoma, lg. cell, non-ker.
		8073/3	Sq. cell carcinoma, sm. cell, non-ker.
		8074/3	Sq. cell carcinoma, spindle cell
		8075/3	Squamous cell carcinoma, adenoid
		8076/2	Sq. cell carc. in situ with question. stromal invas.
		8076/3	Sq. cell carcinoma, micro-invasive
		8078/3	Squamous cell carcinoma with horn formation

OVARY C569

ADENOCARCINOMA, NOS	814	8140/2	Adenocarcinoma in situ
		8140/3	Adenocarcinoma, NOS
		8141/3	Scirrhous adenocarcinoma
		8143/3	Superficial spreading adenocarcinoma
		8147/3	Basal cell adenocarcinoma
SOLID CARCINOMA, NOS	823	8230/2	Duct carcinoma in situ, solid type
		8230/3	Solid carcinoma, NOS
		8231/3	Carcinoma simplex
CARCINOID TUMOR, MALIGNANT	824	8240/3	Carcinoid tumor, malignant
		8241/3	Enterochromaffin cell carcinoid
		8242/3	Enterochromaffin-like cell tumor, malignant
		8243/3	Goblet cell carcinoid
		8244/3	Composite carcinoid
		8245/3	Adenocarcinoid tumor
		8246/3	Neuroendocrine carcinoma
		8249/3	Atypical carcinoid tumor
BRONCHIOLO-ALVEOLAR ADENOCA.	825	8255/3	Adenocarcinoma with mixed subtypes
PAPILLARY ADENOCARCINOMA, NOS	826	8260/3	Papillary adenocarcinoma, NOS
		8261/2	Adenocarcinoma in situ in villous adenoma
		8261/3	Adenocarcinoma in villous adenoma
		8262/3	Villous adenocarcinoma
		8263/2	Adenocarcinoma in situ in tubulovillous adenoma
		8263/3	Adenocarcinoma in tubulovillous adenoma
CLEAR CELL ADENOCARCINOMA, NOS	831	8310/3	Clear cell adenocarcinoma, NOS
		8313/3	Clear cell adenocarcinofibroma
GRANULAR CELL CARCINOMA	832	8320/3	Granular cell carcinoma
		8323/3	Mixed cell adenocarcinoma
ENDOMETRIOID ADENOCARCINOMA	838	8380/3	Endometrioid carcinoma
		8381/3	Endometrioid adenofibroma, malignant
		8382/3	Endometrioid adenocarcinoma, secretory variant
		8383/3	Endometrioid adenocarcinoma, ciliated cell variant
CYSTADENOCARCINOMA, NOS	844	8440/3	Cystadenocarcinoma, NOS
		8441/3	Serous cystadenocarcinoma, NOS
		8442/1	Serous cystadenoma, borderline malignancy (C56.9)
PAPILLARY CYSTADENOCA., NOS	845	8450/3	Papillary cystadenocarcinoma, NOS
		8451/1	Papillary cystadenoma, borderline malignancy (C56.9)

OVARY C569

PAPILLARY SEROUS CYSTADENOCA	846	8460/2	Non-invasive low grade serous carcinoma
		8460/3	Papillary serous cystadenocarcinoma
		8461/3	Serous surface papillary carcinoma
		8462/1	Serous papillary cystic tumor of borderline malignancy (C56.9)
MUCINOUS CYSTADENOCARC., NOS	847	8470/2	Mucinous cystadenocarcinoma, non-invasive
		8470/3	Mucinous cystadenocarcinoma, NOS
		8471/3	Papillary mucinous cystadenocarcinoma
		8472/1	Mucinous cystic tumor of borderline malignancy (C56.9)
		8473/1	Papillary mucinous cystadenoma, borderline malignancy (C56.9)
		8474/3	Seromucinous carcinoma
MUCINOUS ADENOCARCINOMA	848	8480/3	Mucinous adenocarcinoma
		8481/3	Mucin-producing adenocarcinoma
		8482/3	Mucinous adenocarcinoma, endocervical type
SIGNET RING CELL CARCINOMA	849	8490/3	Signet ring cell carcinoma
MEDULLARY CARCINOMA, NOS	851	8510/3	Medullary carcinoma, NOS
ADENOSQUAMOUS CARCINOMA	856	8560/3	Adenosquamous carcinoma
		8562/3	Epithelial-myoepithelial carcinoma
ADENOCA. WITH METAPLASIA	857	8570/3	Adenocarcinoma with squamous metaplasia
		8571/3	Adenocarcinoma w cartilag. & oss. metaplas.
		8572/3	Adenocarcinoma with spindle cell mataplasia
		8573/3	Adenocarcinoma with apocrine metaplasia
		8574/3	Adenocarcinoma with neuroendocrine differen.
		8575/3	Metaplastic carcinoma, NOS
OVARIAN STROMAL TUMOR, MAL.	859	8590/3	Ovarian stromal tumor, mal.
THECOMA, MALIGNANT	860	8600/3	Thecoma, malignant
GRANULOSA CELL TUMOR, MAL.	862	8620/3	Granulosa cell tumor, malignant
		8621/3	Granulosa cell-theca cell tumor, mal.
GONADAL NEOPLASMS	863	8630/3	Androblastoma, malignant
		8631/3	Sertoli-Leydig cell tumor, poorly differentiated
		8632/3	Gynandroblastoma, malignant
		8634/3	Sertoli-Leydig cl tum., p.d. w heterologous elements
LIPID CELL TUMOR, MAL.	867	8670/3	Steroid cell tumor, malignant

OVARY C569

SARCOMA, NOS	880	8800/3	Sarcoma, NOS
		8801/3	Spindle cell sarcoma
		8802/3	Giant cell sarcoma
		8803/3	Small cell sarcoma
		8804/3	Epithelioid sarcoma
		8805/3	Undifferentiated sarcoma
		8806/3	Desmoplastic small round cell tumor
FIBROMATOUS NEOPLASMS	881	8810/3	Fibrosarcoma, NOS
		8811/3	Fibromyxosarcoma
		8813/3	Fascial fibrosarcoma
		8814/3	Infantile fibrosarcoma
		8815/3	Solitary fibrous tumor, malignant
SARCOMA, NOS	882	8825/3	Myofibroblastic sarcoma
MYXOSARCOMA	884	8840/3	Myxosarcoma
MYOMATOUS NEOPLASMS	889	8890/3	Leiomyosarcoma, NOS
		8891/3	Epithelioid leiomyosarcoma
		8894/3	Angiomyosarcoma
		8895/3	Myosarcoma
		8896/3	Myxoid leiomyosarcoma
MULLERIAN MIXED TUMOR	895	8950/3	Mullerian mixed tumor
		8951/3	Mesodermal mixed tumor
CARCINOSARCOMA, NOS	898	8980/3	Carcinosarcoma, NOS
		8981/3	Carcinosarcoma, embryonal type
		8982/3	Malignant myoepithelioma
BRENNER TUMOR, MALIGNANT	900	9000/3	Brenner tumor, malignant
ADENOCARCINOFIBROMA	901	9014/3	Serous adenocarcinofibroma
		9015/3	Mucinous adenocarcinofibroma
GERM CELL TUMORS	906	9060/3	Dysgerminoma
		9064/3	Germinoma
		9065/3	Germ cell tumor, nonseminomatous
EMBRYONAL CARCINOMA, NOS	907	9070/3	Embryonal carcinoma, NOS
		9071/3	Yolk sac tumor
		9072/3	Polyembryoma

OVARY C569

TERATOMA	908	9080/3	Teratoma, malignant, NOS
		9081/3	Teratocarcinoma
		9082/3	Malignant teratoma, undiff.
		9083/3	Malignant teratoma, intermediate
		9084/3	Teratoma with malig. transformation
		9085/3	Mixed germ cell tumor
STRUMA OVARII, MALIGNANT	909	9090/3	Struma ovarii, malignant
CHORIOCARCINOMA	910	9100/3	Choriocarcinoma
		9101/3	Choriocarcinoma combined w/ other germ cell elements
		9105/3	Trophoblastic tumor, epithelioid
MESONEPHROMA, MALIGNANT	911	9110/3	Mesonephroma, malignant
MALIGNANT LYMPHOMA, NOS	959	9590/3	Malignant lymphoma, NOS
		9591/3	Malignant lymphoma, non-Hodgkin
		9596/3	Composite Hodgkin and non-Hodgkin lymphoma
HODGKIN LYMPHOMA	965	9650/3	Hodgkin lymphoma, NOS
		9651/3	Hodgkin lymphoma, lymphocyte-rich
		9652/3	Hodgkin lymphoma, mixed cellularity, NOS
		9653/3	Hodgkin lymphoma, lymphocytic deplet., NOS
		9654/3	Hodgkin lymph., lymphocyt. deplet., diffuse fibrosis
		9655/3	Hodgkin lymphoma, lymphocyt. deplet., reticular
		9659/3	Hodgkin lymph., nodular lymphocyte predom.
HODGKIN LYMPHOMA, NOD. SCLER.	966	9661/3	Hodgkin granuloma [obs]
		9662/3	Hodgkin sarcoma [obs]
		9663/3	Hodgkin lymphoma, nodular sclerosis, NOS
		9664/3	Hodgkin lymphoma, nod. scler., cellular phase
		9665/3	Hodgkin lymphoma, nod. scler., grade 1
		9667/3	Hodgkin lymphoma, nod. scler., grade 2
ML, SMALL B-CELL LYMPHOCYTIC	967	9670/3	ML, small B lymphocytic, NOS
		9671/3	ML, lymphoplasmacytic
		9673/3	Mantle cell lymphoma
		9675/3	ML, mixed sm. and lg. cell, diffuse
ML, LARGE B-CELL, DIFFUSE	968	9680/3	ML, large B-cell, diffuse
		9684/3	ML, large B-cell, diffuse, immunoblastic, NOS
		9687/3	Burkitt lymphoma, NOS
		9688/3	T-cell histiocyte rich large B-cell lymphoma

OVARY C569

FOLLIC. & MARGINAL LYMPH, NOS	969	9690/3	Follicular lymphoma, NOS
		9691/3	Follicular lymphoma, grade 2
		9695/3	Follicular lymphoma, grade 1
		9698/3	Follicular lymphoma, grade 3
		9699/3	Marginal zone B-cell lymphoma, NOS
T-CELL LYMPHOMAS	970	9701/3	Sezary syndrome
		9702/3	Mature T-cell lymphoma, NOS
		9705/3	Angioimmunoblastic T-cell lymphoma
OTHER SPEC. NON-HODGKIN LYMPHOMA	971	9712/3	Intravascular large B-cell lymphoma
		9714/3	Anaplastic large cell lymphoma, T-cell and Null cell type
		9719/3	NK/T-cell lymphoma, nasal and nasal-type
PRECURS. CELL LYMPHOBLASTIC LYMPH.	972	9724/3	SystemicEBV pos. T-cell lymphoproliferative disease of childhood
		9727/3	Precursor cell lymphoblastic lymphoma, NOS
		9728/3	Precursor B-cell lymphoblastic lymphoma
		9729/3	Precursor T-cell lymphoblastic lymphoma
PLASMA CELL TUMORS	973	9731/3	Plasmacytoma, NOS
		9734/3	Plasmacytoma, extramedullary
		9735/3	Plasmablastic lymphoma
		9737/3	ALK positive large B-cell lymphoma
		9738/3	Lrg B-cell lymphoma in HHV8-assoc. multicentric Castleman DZ
MAST CELL TUMORS	974	9740/3	Mast cell sarcoma
		9741/3	Malignant mastocytosis
NEOPLASMS OF HISTIOCYTES AND ACCESSORY LYMPHOID CELLS	975	9750/3	Malignant histiocytosis
		9751/3	Langerhans cell histiocytosis, NOS
		9754/3	Langerhans cell histiocytosis, disseminated
		9755/3	Histiocytic sarcoma
		9756/3	Langerhans cell sarcoma
		9757/3	Interdigitating dendritic cell sarcoma
		9758/3	Follicular dendritic cell sarcoma
		9759/3	Fibroblastic reticular cell tumor
PRECURSOR LYMPHOID NEOPLASMS	981	9811/3	B lymphoblastic leukemia/lymphoma, NOS
		9812/3	Leukemia/lymphoma with t(9;22)(q34;q11.2);BCR-ABL1
		9813/3	Leukemia/lymphoma with t(v;11q23);MLL rearranged
		9814/3	Leukemia/lymphoma with t(12;21)(p13;q22);TEL-AML1(ETV6-RUNX1)
		9815/3	B lymphoblastic leukemia/lymphoma with hyperdiploidy
		9816/3	Leukemia/lymphoma with hypodiploidy (hypodiploid ALL)
		9817/3	B lymphblastic leukemia/lymphoma with t(5;14)(q31;q32);IL3-IGH
		9818/3	Leukemia/lymphoma with t(1;19)(q23;p13.3); E2A PBX1 (TCF3 PBX1)
LYMPHOID LEUKEMIA, NOS	982	9823/3	Chronic lymphocytic leukemia/small lymphocytic lymphoma

OVARY C569

PROLYMPH/PRECURS LEUKEMIA	983	9831/3	T-cell large granular lymphocytic leukemia
		9837/3	T lymphoblastic leukemia/lymphoma
CHRONIC MYELOPROLIFERATIVE DIS.	996	9965/3	Myeloid and lymphoid neoplasms with PDGFRB rearrangement
		9967/3	Myeloid and lymphoid neoplasm with FGFR1 abnormalities
MYELOPLASTIC/MYELOPROLIFERATIVE NEOPLASMS	997	9971/3	Polymorphic PTLD
		9975/3	Myelodysplastic/Myeloproliferative neoplasm, unclassifiable

FALLOPIANTUBE C570

NEOPLASM	800	8000/3	Neoplasm, malignant
		8001/3	Tumor cells, malignant
		8002/3	Malignant tumor, small cell type
		8003/3	Malignant tumor, giant cell type
		8004/3	Malignant tumor, spindle cell type
		8005/3	Malignant tumor, clear cell type
CARCINOMA, NOS	801	8010/2	Carcinoma in situ, NOS
		8010/3	Carcinoma, NOS
		8011/3	Epithelioma, malignant
		8012/3	Large cell carcinoma, NOS
		8013/3	Large cell neuroendocrine carcinoma
		8014/3	Large cell carcinoma with rhabdoid phenotype
		8015/3	Glassy cell carcinoma
CARCINOMA, UNDIFF., NOS	802	8020/3	Carcinoma, undifferentiated type, NOS
		8021/3	Carcinoma, anaplastic type, NOS
		8022/3	Pleomorphic carcinoma
PAPILLARY CARCINOMA, NOS	805	8050/2	Papillary carcinoma in situ
		8050/3	Papillary carcinoma, NOS
		8051/3	Verrucous carcinoma, NOS
		8052/2	Papillary squamous cell carcinoma, non-invasive
		8052/3	Papillary squamous cell carcinoma
SQUAMOUS CELL CARCINOMA, NOS	807	8070/2	Squamous cell carcinoma in situ, NOS
		8070/3	Squamous cell carcinoma, NOS
		8071/3	Sq. cell carcinoma, keratinizing, NOS
		8072/3	Sq. cell carcinoma, lg. cell, non-ker.
		8073/3	Sq. cell carcinoma, sm. cell, non-ker.
		8074/3	Sq. cell carcinoma, spindle cell
		8075/3	Squamous cell carcinoma, adenoid
		8076/2	Sq. cell carc. in situ with question. stromal invas.
		8076/3	Sq. cell carcinoma, micro-invasive
		8078/3	Squamous cell carcinoma with horn formation
LYMPHOEPITHELIAL CARCINOMA	808	8081/2	Bowen disease
		8082/3	Lymphoepithelial carcinoma
		8083/3	Basaloid squamous cell carcinoma
		8084/3	Squamous cell carcinoma, clear cell type
ADENOCARCINOMA, NOS	814	8140/2	Adenocarcinoma in situ
		8140/3	Adenocarcinoma, NOS
		8141/3	Scirrhous adenocarcinoma
		8143/3	Superficial spreading adenocarcinoma
		8147/3	Basal cell adenocarcinoma

FALLOPIANTUBE C570

BRONCHIOLO-ALVEOLAR ADENOCA.	825	8255/3	Adenocarcinoma with mixed subtypes
PAPILLARY ADENOCARCINOMA, NOS	826	8260/3	Papillary adenocarcinoma, NOS
		8261/2	Adenocarcinoma in situ in villous adenoma
		8261/3	Adenocarcinoma in villous adenoma
		8262/3	Villous adenocarcinoma
		8263/2	Adenocarcinoma in situ in tubulovillous adenoma
		8263/3	Adenocarcinoma in tubulovillous adenoma
CLEAR CELL ADENOCARCINOMA, NOS	831	8310/3	Clear cell adenocarcinoma, NOS
GRANULAR CELL CARCINOMA	832	8323/3	Mixed cell adenocarcinoma
ENDOMETRIOID ADENOCARCINOMA	838	8380/3	Endometrioid carcinoma
		8382/3	Endometrioid adenocarcinoma, secretory variant
		8383/3	Endometrioid adenocarcinoma, ciliated cell variant
CYSTADENOCARCINOMA, NOS	844	8440/3	Cystadenocarcinoma, NOS
		8441/2	Serous tubal intraepithelial carcinoma
MUCINOUS ADENOCARCINOMA	848	8480/3	Mucinous adenocarcinoma
		8481/3	Mucin-producing adenocarcinoma
		8482/3	Mucinous adenocarcinoma, endocervical type
ADENOCA. WITH METAPLASIA	857	8570/3	Adenocarcinoma with squamous metaplasia
		8571/3	Adenocarcinoma w cartilag. & oss. metaplas.
		8572/3	Adenocarcinoma with spindle cell mataplasia
		8573/3	Adenocarcinoma with apocrine metaplasia
		8574/3	Adenocarcinoma with neuroendocrine differen.
		8575/3	Metaplastic carcinoma, NOS
SARCOMA, NOS	880	8800/3	Sarcoma, NOS
		8801/3	Spindle cell sarcoma
		8802/3	Giant cell sarcoma
		8803/3	Small cell sarcoma
		8804/3	Epithelioid sarcoma
		8805/3	Undifferentiated sarcoma
		8806/3	Desmoplastic small round cell tumor
FIBROMATOUS NEOPLASMS	881	8810/3	Fibrosarcoma, NOS
		8811/3	Fibromyxosarcoma
		8813/3	Fascial fibrosarcoma
		8814/3	Infantile fibrosarcoma
		8815/3	Solitary fibrous tumor, malignant
SARCOMA, NOS	882	8825/3	Myofibroblastic sarcoma

FALLOPIAN TUBE C570

MYOMATOUS NEOPLASMS	889	8890/3	Leiomyosarcoma, NOS
		8891/3	Epithelioid leiomyosarcoma
		8894/3	Angiomyosarcoma
		8895/3	Myosarcoma
		8896/3	Myxoid leiomyosarcoma
MULLERIAN MIXED TUMOR	895	8950/3	Mullerian mixed tumor
		8951/3	Mesodermal mixed tumor
CARCINOSARCOMA, NOS	898	8980/3	Carcinosarcoma, NOS
		8981/3	Carcinosarcoma, embryonal type
		8982/3	Malignant myoepithelioma
MESONEPHROMA, MALIGNANT	911	9110/3	Mesonephroma, malignant
MALIGNANT LYMPHOMA, NOS	959	9590/3	Malignant lymphoma, NOS
		9591/3	Malignant lymphoma, non-Hodgkin
		9596/3	Composite Hodgkin and non-Hodgkin lymphoma
HODGKIN LYMPHOMA	965	9650/3	Hodgkin lymphoma, NOS
		9651/3	Hodgkin lymphoma, lymphocyte-rich
		9652/3	Hodgkin lymphoma, mixed cellularity, NOS
		9653/3	Hodgkin lymphoma, lymphocytic deplet., NOS
		9654/3	Hodgkin lymph., lymphocyt. deplet., diffuse fibrosis
		9655/3	Hodgkin lymphoma, lymphocyt. deplet., reticular
		9659/3	Hodgkin lymph., nodular lymphocyte predom.
HODGKIN LYMPHOMA, NOD. SCLER.	966	9661/3	Hodgkin granuloma [obs]
		9662/3	Hodgkin sarcoma [obs]
		9663/3	Hodgkin lymphoma, nodular sclerosis, NOS
		9664/3	Hodgkin lymphoma, nod. scler., cellular phase
		9665/3	Hodgkin lymphoma, nod. scler., grade 1
		9667/3	Hodgkin lymphoma, nod. scler., grade 2
ML, SMALL B-CELL LYMPHOCYTIC	967	9670/3	ML, small B lymphocytic, NOS
		9671/3	ML, lymphoplasmacytic
		9673/3	Mantle cell lymphoma
		9675/3	ML, mixed sm. and lg. cell, diffuse
ML, LARGE B-CELL, DIFFUSE	968	9680/3	ML, large B-cell, diffuse
		9684/3	ML, large B-cell, diffuse, immunoblastic, NOS
		9687/3	Burkitt lymphoma, NOS
		9688/3	T-cell histiocyte rich large B-cell lymphoma

FALLOPIANTUBE C570

FOLLIC. & MARGINAL LYMPH, NOS	969	9690/3	Follicular lymphoma, NOS
		9691/3	Follicular lymphoma, grade 2
		9695/3	Follicular lymphoma, grade 1
		9698/3	Follicular lymphoma, grade 3
		9699/3	Marginal zone B-cell lymphoma, NOS
T-CELL LYMPHOMAS	970	9701/3	Sezary syndrome
		9702/3	Mature T-cell lymphoma, NOS
		9705/3	Angioimmunoblastic T-cell lymphoma
OTHER SPEC. NON-HODGKIN LYMPHOMA	971	9712/3	Intravascular large B-cell lymphoma
		9714/3	Anaplastic large cell lymphoma, T-cell and Null cell type
		9719/3	NK/T-cell lymphoma, nasal and nasal-type
PRECURS. CELL LYMPHOBLASTIC LYMPH.	972	9724/3	SystemicEBV pos. T-cell lymphoproliferative disease of childhood
		9727/3	Precursor cell lymphoblastic lymphoma, NOS
		9728/3	Precursor B-cell lymphoblastic lymphoma
		9729/3	Precursor T-cell lymphoblastic lymphoma
PLASMA CELL TUMORS	973	9731/3	Plasmacytoma, NOS
		9734/3	Plasmacytoma, extramedullary
		9735/3	Plasmablastic lymphoma
		9737/3	ALK positive large B-cell lymphoma
		9738/3	Lrg B-cell lymphoma in HHV8-assoc. multicentric Castleman DZ
MAST CELL TUMORS	974	9740/3	Mast cell sarcoma
		9741/3	Malignant mastocytosis
NEOPLASMS OF HISTIOCYTES AND ACCESSORY LYMPHOID CELLS	975	9750/3	Malignant histiocytosis
		9751/3	Langerhans cell histiocytosis, NOS
		9754/3	Langerhans cell histiocytosis, disseminated
		9755/3	Histiocytic sarcoma
		9756/3	Langerhans cell sarcoma
		9757/3	Interdigitating dendritic cell sarcoma
		9758/3	Follicular dendritic cell sarcoma
		9759/3	Fibroblastic reticular cell tumor
PRECURSOR LYMPHOID NEOPLASMS	981	9811/3	B lymphoblastic leukemia/lymphoma, NOS
		9812/3	Leukemia/lymphoma with t(9;22)(q34;q11.2);BCR-ABL1
		9813/3	Leukemia/lymphoma with t(v;11q23);MLL rearranged
		9814/3	Leukemia/lymphoma with t(12;21)(p13;q22);TEL-AML1(ETV6-RUNX1)
		9815/3	B lymphoblastic leukemia/lymphoma with hyperdiploidy
		9816/3	Leukemia/lymphoma with hypodiploidy (hypodiploid ALL)
		9817/3	B lymphblastic leukemia/lymphoma with t(5;14)(q31;q32);IL3-IGH
		9818/3	Leukemia/lymphoma with t(1;19)(q23;p13.3); E2A PBX1 (TCF3 PBX1)
LYMPHOID LEUKEMIA, NOS	982	9823/3	Chronic lymphocytic leukemia/small lymphocytic lymphoma

FALLOPIANTUBE C570

PROLYMPH/PRECURS LEUKEMIA	983	9831/3	T-cell large granular lymphocytic leukemia
		9837/3	T lymphoblastic leukemia/lymphoma
CHRONIC MYELOPROLIFERATIVE DIS.	996	9965/3	Myeloid and lymphoid neoplasms with PDGFRB rearrangement
		9967/3	Myeloid and lymphoid neoplasm with FGFR1 abnormalities
MYELOPLASTIC/MYELOPROLIFERATIVE NEOPLASMS	997	9971/3	Polymorphic PTLD
		9975/3	Myelodysplastic/Myeloproliferative neoplasm, unclassifiable

OTHER FEMALE GENITAL (EXCL FALLOPIAN TUBE) C571-C574,C577-C579

NEOPLASM	800	8000/3	Neoplasm, malignant
		8001/3	Tumor cells, malignant
		8002/3	Malignant tumor, small cell type
		8003/3	Malignant tumor, giant cell type
		8004/3	Malignant tumor, spindle cell type
		8005/3	Malignant tumor, clear cell type
CARCINOMA, NOS	801	8010/2	Carcinoma in situ, NOS
		8010/3	Carcinoma, NOS
		8011/3	Epithelioma, malignant
		8012/3	Large cell carcinoma, NOS
		8013/3	Large cell neuroendocrine carcinoma
		8014/3	Large cell carcinoma with rhabdoid phenotype
		8015/3	Glassy cell carcinoma
CARCINOMA, UNDIFF., NOS	802	8020/3	Carcinoma, undifferentiated type, NOS
		8021/3	Carcinoma, anaplastic type, NOS
		8022/3	Pleomorphic carcinoma
PAPILLARY CARCINOMA, NOS	805	8050/2	Papillary carcinoma in situ
		8050/3	Papillary carcinoma, NOS
		8051/3	Verrucous carcinoma, NOS
		8052/2	Papillary squamous cell carcinoma, non-invasive
		8052/3	Papillary squamous cell carcinoma
SQUAMOUS CELL CARCINOMA, NOS	807	8070/2	Squamous cell carcinoma in situ, NOS
		8070/3	Squamous cell carcinoma, NOS
		8071/3	Sq. cell carcinoma, keratinizing, NOS
		8072/3	Sq. cell carcinoma, lg. cell, non-ker.
		8073/3	Sq. cell carcinoma, sm. cell, non-ker.
		8074/3	Sq. cell carcinoma, spindle cell
		8075/3	Squamous cell carcinoma, adenoid
		8076/2	Sq. cell carc. in situ with question. stromal invas.
		8076/3	Sq. cell carcinoma, micro-invasive
		8078/3	Squamous cell carcinoma with horn formation
LYMPHOEPITHELIAL CARCINOMA	808	8081/2	Bowen disease
		8082/3	Lymphoepithelial carcinoma
		8083/3	Basaloid squamous cell carcinoma
		8084/3	Squamous cell carcinoma, clear cell type
ADENOCARCINOMA, NOS	814	8140/2	Adenocarcinoma in situ
		8140/3	Adenocarcinoma, NOS
		8141/3	Scirrhous adenocarcinoma
		8143/3	Superficial spreading adenocarcinoma
		8147/3	Basal cell adenocarcinoma

OTHER FEMALE GENITAL (EXCL FALLOPIAN TUBE) C571-C574,C577-C579

BRONCHIOLO-ALVEOLAR ADENOCA.	825	8255/3	Adenocarcinoma with mixed subtypes
PAPILLARY ADENOCARCINOMA, NOS	826	8260/3	Papillary adenocarcinoma, NOS
		8261/2	Adenocarcinoma in situ in villous adenoma
		8261/3	Adenocarcinoma in villous adenoma
		8262/3	Villous adenocarcinoma
		8263/2	Adenocarcinoma in situ in tubulovillous adenoma
		8263/3	Adenocarcinoma in tubulovillous adenoma
CLEAR CELL ADENOCARCINOMA, NOS	831	8310/3	Clear cell adenocarcinoma, NOS
GRANULAR CELL CARCINOMA	832	8323/3	Mixed cell adenocarcinoma
ENDOMETRIOID ADENOCARCINOMA	838	8380/3	Endometrioid carcinoma
		8382/3	Endometrioid adenocarcinoma, secretory variant
		8383/3	Endometrioid adenocarcinoma, ciliated cell variant
CYSTADENOCARCINOMA, NOS	844	8440/3	Cystadenocarcinoma, NOS
MUCINOUS ADENOCARCINOMA	848	8480/3	Mucinous adenocarcinoma
		8481/3	Mucin-producing adenocarcinoma
		8482/3	Mucinous adenocarcinoma, endocervical type
ADENOCA. WITH METAPLASIA	857	8570/3	Adenocarcinoma with squamous metaplasia
		8571/3	Adenocarcinoma w cartilag. & oss. metaplas.
		8572/3	Adenocarcinoma with spindle cell mataplasia
		8573/3	Adenocarcinoma with apocrine metaplasia
		8574/3	Adenocarcinoma with neuroendocrine differen.
		8575/3	Metaplastic carcinoma, NOS
SARCOMA, NOS	880	8800/3	Sarcoma, NOS
		8801/3	Spindle cell sarcoma
		8802/3	Giant cell sarcoma
		8803/3	Small cell sarcoma
		8804/3	Epithelioid sarcoma
		8805/3	Undifferentiated sarcoma
		8806/3	Desmoplastic small round cell tumor
FIBROMATOUS NEOPLASMS	881	8810/3	Fibrosarcoma, NOS
		8811/3	Fibromyxosarcoma
		8813/3	Fascial fibrosarcoma
		8814/3	Infantile fibrosarcoma
		8815/3	Solitary fibrous tumor, malignant
SARCOMA, NOS	882	8825/3	Myofibroblastic sarcoma

OTHER FEMALE GENITAL (EXCL FALLOPIAN TUBE) C571-C574,C577-C579

MYOMATOUS NEOPLASMS	889	8890/3	Leiomyosarcoma, NOS
		8891/3	Epithelioid leiomyosarcoma
		8894/3	Angiomyosarcoma
		8895/3	Myosarcoma
		8896/3	Myxoid leiomyosarcoma
MULLERIAN MIXED TUMOR	895	8950/3	Mullerian mixed tumor
		8951/3	Mesodermal mixed tumor
CARCINOSARCOMA, NOS	898	8980/3	Carcinosarcoma, NOS
		8981/3	Carcinosarcoma, embryonal type
		8982/3	Malignant myoepithelioma
MESONEPHROMA, MALIGNANT	911	9110/3	Mesonephroma, malignant
MALIGNANT LYMPHOMA, NOS	959	9590/3	Malignant lymphoma, NOS
		9591/3	Malignant lymphoma, non-Hodgkin
		9596/3	Composite Hodgkin and non-Hodgkin lymphoma
HODGKIN LYMPHOMA	965	9650/3	Hodgkin lymphoma, NOS
		9651/3	Hodgkin lymphoma, lymphocyte-rich
		9652/3	Hodgkin lymphoma, mixed cellularity, NOS
		9653/3	Hodgkin lymphoma, lymphocytic deplet., NOS
		9654/3	Hodgkin lymph., lymphocyt. deplet., diffuse fibrosis
		9655/3	Hodgkin lymphoma, lymphocyt. deplet., reticular
		9659/3	Hodgkin lymph., nodular lymphocyte predom.
HODGKIN LYMPHOMA, NOD. SCLER.	966	9661/3	Hodgkin granuloma [obs]
		9662/3	Hodgkin sarcoma [obs]
		9663/3	Hodgkin lymphoma, nodular sclerosis, NOS
		9664/3	Hodgkin lymphoma, nod. scler., cellular phase
		9665/3	Hodgkin lymphoma, nod. scler., grade 1
		9667/3	Hodgkin lymphoma, nod. scler., grade 2
ML, SMALL B-CELL LYMPHOCYTIC	967	9670/3	ML, small B lymphocytic, NOS
		9671/3	ML, lymphoplasmacytic
		9673/3	Mantle cell lymphoma
		9675/3	ML, mixed sm. and lg. cell, diffuse
ML, LARGE B-CELL, DIFFUSE	968	9680/3	ML, large B-cell, diffuse
		9684/3	ML, large B-cell, diffuse, immunoblastic, NOS
		9687/3	Burkitt lymphoma, NOS
		9688/3	T-cell histiocyte rich large B-cell lymphoma

OTHER FEMALE GENITAL (EXCL FALLOPIAN TUBE) C571-C574,C577-C579

FOLLIC. & MARGINAL LYMPH, NOS	969	9690/3	Follicular lymphoma, NOS
		9691/3	Follicular lymphoma, grade 2
		9695/3	Follicular lymphoma, grade 1
		9698/3	Follicular lymphoma, grade 3
		9699/3	Marginal zone B-cell lymphoma, NOS
T-CELL LYMPHOMAS	970	9701/3	Sezary syndrome
		9702/3	Mature T-cell lymphoma, NOS
		9705/3	Angioimmunoblastic T-cell lymphoma
OTHER SPEC. NON-HODGKIN LYMPHOMA	971	9712/3	Intravascular large B-cell lymphoma
		9714/3	Anaplastic large cell lymphoma, T-cell and Null cell type
		9719/3	NK/T-cell lymphoma, nasal and nasal-type
PRECURS. CELL LYMPHOBLASTIC LYMPH.	972	9724/3	SystemicEBV pos. T-cell lymphoproliferative disease of childhood
		9727/3	Precursor cell lymphoblastic lymphoma, NOS
		9728/3	Precursor B-cell lymphoblastic lymphoma
		9729/3	Precursor T-cell lymphoblastic lymphoma
PLASMA CELL TUMORS	973	9731/3	Plasmacytoma, NOS
		9734/3	Plasmacytoma, extramedullary
		9735/3	Plasmablastic lymphoma
		9737/3	ALK positive large B-cell lymphoma
		9738/3	Lrg B-cell lymphoma in HHV8-assoc. multicentric Castleman DZ
MAST CELL TUMORS	974	9740/3	Mast cell sarcoma
		9741/3	Malignant mastocytosis
NEOPLASMS OF HISTIOCYTES AND ACCESSORY LYMPHOID CELLS	975	9750/3	Malignant histiocytosis
		9751/3	Langerhans cell histiocytosis, NOS
		9754/3	Langerhans cell histiocytosis, disseminated
		9755/3	Histiocytic sarcoma
		9756/3	Langerhans cell sarcoma
		9757/3	Interdigitating dendritic cell sarcoma
		9758/3	Follicular dendritic cell sarcoma
		9759/3	Fibroblastic reticular cell tumor
PRECURSOR LYMPHOID NEOPLASMS	981	9811/3	B lymphoblastic leukemia/lymphoma, NOS
		9812/3	Leukemia/lymphoma with t(9;22)(q34;q11.2);BCR-ABL1
		9813/3	Leukemia/lymphoma with t(v;11q23);MLL rearranged
		9814/3	Leukemia/lymphoma with t(12;21)(p13;q22);TEL-AML1(ETV6-RUNX1)
		9815/3	B lymphoblastic leukemia/lymphoma with hyperdiploidy
		9816/3	Leukemia/lymphoma with hypodiploidy (hypodiploid ALL)
		9817/3	B lymphblastic leukemia/lymphoma with t(5;14)(q31;q32);IL3-IGH
		9818/3	Leukemia/lymphoma with t(1;19)(q23;p13.3); E2A PBX1 (TCF3 PBX1)
LYMPHOID LEUKEMIA, NOS	982	9823/3	Chronic lymphocytic leukemia/small lymphocytic lymphoma

OTHER FEMALE GENITAL (EXCL FALLOPIAN TUBE) C571-C574,C577-C579

PROLYMPH/PRECURS LEUKEMIA	983	9831/3	T-cell large granular lymphocytic leukemia
		9837/3	T lymphoblastic leukemia/lymphoma
CHRONIC MYELOPROLIFERATIVE DIS.	996	9965/3	Myeloid and lymphoid neoplasms with PDGFRB rearrangement
		9967/3	Myeloid and lymphoid neoplasm with FGFR1 abnormalities
MYELOPLASTIC/MYELOPROLIFERATIVE NEOPLASMS	997	9971/3	Polymorphic PTLD
		9975/3	Myelodysplastic/Myeloproliferative neoplasm, unclassifiable

PLACENTA C589

NEOPLASM	800	8000/3	Neoplasm, malignant
		8001/3	Tumor cells, malignant
		8002/3	Malignant tumor, small cell type
		8003/3	Malignant tumor, giant cell type
		8004/3	Malignant tumor, spindle cell type
		8005/3	Malignant tumor, clear cell type
CHORIOCARCINOMA	910	9100/3	Choriocarcinoma
		9101/3	Choriocarcinoma combined w/ other germ cell elements
		9104/3	Malignant placental site trophoblastic tumor
		9105/3	Trophoblastic tumor, epithelioid
FOLLIC. & MARGINAL LYMPH, NOS	969	9699/3	Marginal zone B-cell lymphoma, NOS
LYMPHOID LEUKEMIA, NOS	982	9823/3	Chronic lymphocytic leukemia/small lymphocytic lymphoma

PENIS C600-C602,C608-C609

NEOPLASM	800	8000/3	Neoplasm, malignant
		8001/3	Tumor cells, malignant
		8002/3	Malignant tumor, small cell type
		8003/3	Malignant tumor, giant cell type
		8004/3	Malignant tumor, spindle cell type
		8005/3	Malignant tumor, clear cell type
CARCINOMA, NOS	801	8010/2	Carcinoma in situ, NOS
		8010/3	Carcinoma, NOS
		8011/3	Epithelioma, malignant
		8012/3	Large cell carcinoma, NOS
		8013/3	Large cell neuroendocrine carcinoma
		8014/3	Large cell carcinoma with rhabdoid phenotype
		8015/3	Glassy cell carcinoma
CARCINOMA, UNDIFF., NOS	802	8020/3	Carcinoma, undifferentiated type, NOS
		8021/3	Carcinoma, anaplastic type, NOS
		8022/3	Pleomorphic carcinoma
PAPILLARY CARCINOMA, NOS	805	8050/2	Papillary carcinoma in situ
		8050/3	Papillary carcinoma, NOS
		8051/3	Verrucous carcinoma, NOS
		8052/2	Papillary squamous cell carcinoma, non-invasive
		8052/3	Papillary squamous cell carcinoma
		8054/3	Warty carcinoma
SQUAMOUS CELL CARCINOMA, NOS	807	8070/2	Squamous cell carcinoma in situ, NOS
		8070/3	Squamous cell carcinoma, NOS
		8071/2	Sq. cell carcinoma, keratinizing, NOS, in situ
		8071/3	Sq. cell carcinoma, keratinizing, NOS
		8072/3	Sq. cell carcinoma, lg. cell, non-ker.
		8073/3	Sq. cell carcinoma, sm. cell, non-ker.
		8074/3	Sq. cell carcinoma, spindle cell
		8075/3	Squamous cell carcinoma, adenoid
		8076/2	Sq. cell carc. in situ with question. stromal invas.
		8076/3	Sq. cell carcinoma, micro-invasive
		8078/3	Squamous cell carcinoma with horn formation
LYMPHOEPITHELIAL CARCINOMA	808	8080/2	Queyrat erythroplasia
		8081/2	Bowen disease
		8082/3	Lymphoepithelial carcinoma
		8083/3	Basaloid squamous cell carcinoma
		8084/3	Squamous cell carcinoma, clear cell type

PENIS C600-C602,C608-C609

BASAL CELL CARCINOMA, NOS	809	8090/3	Basal cell carcinoma, NOS
		8091/3	Multifocal superficial basal cell carcinoma
		8092/3	Infiltrating basal cell carcinoma, NOS
		8093/3	Basal cell carcinoma, fibroepithelial
		8094/3	Basosquamous carcinoma
		8095/3	Metatypical carcinoma
		8097/3	Basal cell carcinoma, nodular
		8098/3	Adenoid basal cell carcinoma
TRICHILEMMOCARCINOMA	810	8102/3	Trichilemmocarcinoma
PILOMATRIX CARCINOMA	811	8110/3	Pilomatrix carcinoma
TRANSITIONAL CELL CARCINOMA, NOS	812	8120/2	Transitional cell carcinoma in situ
		8120/3	Transitional cell carcinoma, NOS
		8121/3	Schneiderian carcinoma
		8122/3	Trans. cell carcinoma, spindle cell
		8123/3	Basaloid carcinoma
		8124/3	Cloacogenic carcinoma
ADENOCARCINOMA, NOS	814	8140/2	Adenocarcinoma in situ
		8140/3	Adenocarcinoma, NOS
		8141/3	Scirrhous adenocarcinoma
		8143/3	Superficial spreading adenocarcinoma
		8147/3	Basal cell adenocarcinoma
BRONCHIOLO-ALVEOLAR ADENOCA.	825	8255/3	Adenocarcinoma with mixed subtypes
PAPILLARY ADENOCARCINOMA, NOS	826	8260/3	Papillary adenocarcinoma, NOS
		8261/2	Adenocarcinoma in situ in villous adenoma
		8261/3	Adenocarcinoma in villous adenoma
		8262/3	Villous adenocarcinoma
		8263/2	Adenocarcinoma in situ in tubulovillous adenoma
		8263/3	Adenocarcinoma in tubulovillous adenoma
SKIN APPENDAGE CARCINOMA	839	8390/3	Skin appendage carcinoma
SWEAT GLAND ADENOCARCINOMA	840	8400/3	Sweat gland adenocarcinoma
		8401/3	Apocrine adenocarcinoma
SEBACEOUS/ECCRINE ADENOCA.	841	8410/3	Sebaceous adenocarcinoma
		8413/3	Eccrine adenocarcinoma
PAGET DISEASE, EXTRAMAMMARY	854	8542/3	Paget disease, extramammary

PENIS C600-C602,C608-C609

ADENOCA. WITH METAPLASIA	857	8570/3	Adenocarcinoma with squamous metaplasia
		8571/3	Adenocarcinoma w cartilag. & oss. metaplas.
		8572/3	Adenocarcinoma with spindle cell mataplasia
		8573/3	Adenocarcinoma with apocrine metaplasia
		8574/3	Adenocarcinoma with neuroendocrine differen.
		8575/3	Metaplastic carcinoma, NOS
NEVI & MELANOMAS	872	8720/2	Melanoma in situ
		8720/3	Malignant melanoma, NOS
		8721/3	Nodular melanoma
		8722/3	Balloon cell melanoma
		8723/3	Malignant melanoma, regressing
AMELANOTIC MELANOMA	873	8730/3	Amelanotic melanoma
MAL. MEL. IN JUNCT. NEVUS	874	8740/3	Mal. melanoma in junctional nevus
		8741/2	Precancerous melanosis, NOS
		8741/3	Mal. melanoma in precan. melanosis
		8742/2	Lentigo maligna
		8742/3	Lentigo maligna melanoma
		8743/3	Superficial spreading melanoma
		8745/3	Desmoplastic melanoma, malignant
		8746/3	Mucosal lentiginous melanoma
MAL. MELAN. IN GIANT PIGMT. NEVUS	876	8761/3	Mal. melanoma in giant pigmented nevus
EPITHELIOID CELL MELANOMA	877	8770/3	Mixed epithel. & spindle cell melanoma
		8771/3	Epithelioid cell melanoma
		8772/3	Spindle cell melanoma, NOS
BLUE NEVUS, MALIGNANT	878	8780/3	Blue nevus, malignant
SARCOMA, NOS	880	8800/3	Sarcoma, NOS
		8801/3	Spindle cell sarcoma
		8802/3	Giant cell sarcoma
		8803/3	Small cell sarcoma
		8804/3	Epithelioid sarcoma
		8805/3	Undifferentiated sarcoma
		8806/3	Desmoplastic small round cell tumor
FIBROMATOUS NEOPLASMS	881	8810/3	Fibrosarcoma, NOS
		8811/3	Fibromyxosarcoma
		8813/3	Fascial fibrosarcoma
		8814/3	Infantile fibrosarcoma
		8815/3	Solitary fibrous tumor, malignant
SARCOMA, NOS	882	8825/3	Myofibroblastic sarcoma

PENIS C600-C602,C608-C609

FIBROUS HISTIOCYTOMA, MAL.	883	8830/3	Fibrous histiocytoma, malignant
LIPOSARCOMA NEOPLASMS	885	8850/3	Liposarcoma, NOS
		8851/3	Liposarcoma, well differentiated
		8852/3	Myxoid liposarcoma
		8853/3	Round cell liposarcoma
		8854/3	Pleomorphic liposarcoma
		8855/3	Mixed type liposarcoma
		8857/3	Fibroblastic liposarcoma
		8858/3	Dedifferentiated liposarcoma
MYOMATOUS NEOPLASMS	889	8890/3	Leiomyosarcoma, NOS
		8891/3	Epithelioid leiomyosarcoma
		8894/3	Angiomyosarcoma
		8895/3	Myosarcoma
		8896/3	Myxoid leiomyosarcoma
RHABDOMYOSARCOMA, NOS	890	8900/3	Rhabdomyosarcoma, NOS
		8901/3	Pleomorphic rhabdomyosarcoma, adult type
		8902/3	Mixed type rhabdomyosarcoma
EMBRYONAL RHABDOMYOSARCOMA	891	8910/3	Embryonal rhabdomyosarcoma
		8912/3	Spindle cell rhabdomyosarcoma
ALVEOLAR RHABDOMYOSARCOMA	892	8920/3	Alveolar rhabdomyosarcoma
		8921/3	Rhabdomyosarcoma with ganglionic differentiation
MULLERIAN MIXED TUMOR	895	8950/3	Mullerian mixed tumor
		8951/3	Mesodermal mixed tumor
CARCINOSARCOMA, NOS	898	8980/3	Carcinosarcoma, NOS
		8981/3	Carcinosarcoma, embryonal type
		8982/3	Malignant myoepithelioma
MESENCHYMOMA, MALIGNANT	899	8990/3	Mesenchymoma, malignant
		8991/3	Embryonal sarcoma
KAPOSI SARCOMA	914	9140/3	Kaposi sarcoma
MALIGNANT LYMPHOMA, NOS	959	9597/3	Primary Cutaneous follicle centre lymphoma
FOLLIC. & MARGINAL LYMPH, NOS	969	9699/3	Marginal zone B-cell lymphoma, NOS

PENIS C600-C602,C608-C609

T-CELL LYMPHOMAS	970	9700/3	Mycosis fungoides
		9701/3	Sezary syndrome
		9708/3	Subcutaneous panniculitis-like T-cell lymphoma
		9709/3	Cutaneous T-cell lymphoma, NOS
OTHER SPEC. NON-HODGKIN LYMPHOMA	971	9718/3	Primary cutan. CD30+ T-cell lymphoprolif. disorder
PRECURS. CELL LYMPHOBLASTIC LYMPH.	972	9725/3	Hydroa vacciniforme-like lymphoma
		9726/3	Primary Cutaneous gamma-delta T-cell lymphoma
LYMPHOID LEUKEMIA, NOS	982	9823/3	Chronic lymphocytic leukemia/small lymphocytic lymphoma

PROSTATE GLAND C619

NEOPLASM	800	8000/3	Neoplasm, malignant
		8001/3	Tumor cells, malignant
		8002/3	Malignant tumor, small cell type
		8003/3	Malignant tumor, giant cell type
		8004/3	Malignant tumor, spindle cell type
		8005/3	Malignant tumor, clear cell type
CARCINOMA, NOS	801	8010/2	Carcinoma in situ, NOS
		8010/3	Carcinoma, NOS
		8011/3	Epithelioma, malignant
		8012/3	Large cell carcinoma, NOS
		8013/3	Large cell neuroendocrine carcinoma
		8014/3	Large cell carcinoma with rhabdoid phenotype
		8015/3	Glassy cell carcinoma
CARCINOMA, UNDIFF., NOS	802	8020/3	Carcinoma, undifferentiated type, NOS
		8021/3	Carcinoma, anaplastic type, NOS
		8022/3	Pleomorphic carcinoma
GIANT & SPINDLE CELL CARCINOMA	803	8030/3	Giant cell and spindle cell carcinoma
		8031/3	Giant cell carcinoma
		8032/3	Spindle cell carcinoma
		8033/3	Pseudosarcomatous carcinoma
		8034/3	Polygonal cell carcinoma
		8035/3	Carcinoma with osteoclast-like giant cells
SMALL CELL CARCINOMA, NOS	804	8041/3	Small cell carcinoma, NOS
		8043/3	Small cell carcinoma, fusiform cell
PAPILLARY CARCINOMA, NOS	805	8050/2	Papillary carcinoma in situ
		8050/3	Papillary carcinoma, NOS
		8051/3	Verrucous carcinoma, NOS
		8052/2	Papillary squamous cell carcinoma, non-invasive
		8052/3	Papillary squamous cell carcinoma
SQUAMOUS CELL CARCINOMA, NOS	807	8070/2	Squamous cell carcinoma in situ, NOS
		8070/3	Squamous cell carcinoma, NOS
		8071/3	Sq. cell carcinoma, keratinizing, NOS
		8072/3	Sq. cell carcinoma, lg. cell, non-ker.
		8073/3	Sq. cell carcinoma, sm. cell, non-ker.
		8074/3	Sq. cell carcinoma, spindle cell
		8075/3	Squamous cell carcinoma, adenoid
		8076/2	Sq. cell carc. in situ with question. stromal invas.
		8076/3	Sq. cell carcinoma, micro-invasive
		8078/3	Squamous cell carcinoma with horn formation

PROSTATE GLAND C619

TRANSITIONAL CELL CARCINOMA, NOS	812	8120/2	Transitional cell carcinoma in situ
		8120/3	Transitional cell carcinoma, NOS
		8121/3	Schneiderian carcinoma
		8122/3	Trans. cell carcinoma, spindle cell
		8123/3	Basaloid carcinoma
		8124/3	Cloacogenic carcinoma
PAPILLARY TRANS. CELL CARCINOMA	813	8130/2	Papillary trans. cell carcinoma, non-invasive
		8130/3	Papillary trans. cell carcinoma
		8131/3	Transitional cell carcinoma, micropapillary
ADENOCARCINOMA, NOS	814	8140/2	Adenocarcinoma in situ
		8140/3	Adenocarcinoma, NOS
		8141/3	Scirrhous adenocarcinoma
		8143/3	Superficial spreading adenocarcinoma
		8147/3	Basal cell adenocarcinoma
		8148/2	Glandular intraepithelial neoplasia, grade III
ADENOID CYSTIC & CRIBRIFORM CA.	820	8200/3	Adenoid cystic carcinoma
		8201/2	Cribriform carcinoma in situ
		8201/3	Cribriform carcinoma
ADENOCA. IN ADENOMA. POLYP	821	8211/3	Tubular adenocarcinoma
BRONCHIOLO-ALVEOLAR ADENOCA.	825	8251/3	Alveolar adenocarcinoma
		8255/3	Adenocarcinoma with mixed subtypes
PAPILLARY ADENOCARCINOMA, NOS	826	8260/3	Papillary adenocarcinoma, NOS
		8261/2	Adenocarcinoma in situ in villous adenoma
		8261/3	Adenocarcinoma in villous adenoma
		8262/3	Villous adenocarcinoma
		8263/2	Adenocarcinoma in situ in tubulovillous adenoma
		8263/3	Adenocarcinoma in tubulovillous adenoma
CLEAR CELL ADENOCARCINOMA, NOS	831	8310/3	Clear cell adenocarcinoma, NOS
MUCINOUS ADENOCARCINOMA	848	8480/3	Mucinous adenocarcinoma
		8481/3	Mucin-producing adenocarcinoma
DUCT CARCINOMA	850	8500/2	Intraductal carcinoma, noninfiltrating, NOS
		8500/3	Invasive carcinoma of no special type
		8503/2	Noninfiltrating intraductal papillary adenocarcinoma
		8503/3	Intraductal papillary adenocarcinoma with invasion
		8504/2	Noninfiltrating intracystic carcinoma
		8504/3	Intracystic carcinoma, NOS
		8507/2	Intraductal micropapillary carcinoma

PROSTATE GLAND C619

MEDULLARY CARCINOMA, NOS	851	8510/3	Medullary carcinoma, NOS
		8514/3	Duct carcinoma, desmoplastic type
ACINAR CELL CARCINOMA	855	8550/3	Acinar cell carcinoma
		8551/3	Acinar cell cystadenocarcinoma
ADENOSQUAMOUS CARCINOMA	856	8560/3	Adenosquamous carcinoma
		8562/3	Epithelial-myoepithelial carcinoma
ADENOCA. WITH METAPLASIA	857	8570/3	Adenocarcinoma with squamous metaplasia
		8571/3	Adenocarcinoma w cartilag. & oss. metaplas.
		8572/3	Adenocarcinoma with spindle cell mataplasia
		8573/3	Adenocarcinoma with apocrine metaplasia
		8574/3	Adenocarcinoma with neuroendocrine differen.
		8575/3	Metaplastic carcinoma, NOS
SARCOMA, NOS	880	8800/3	Sarcoma, NOS
		8801/3	Spindle cell sarcoma
		8802/3	Giant cell sarcoma
		8803/3	Small cell sarcoma
		8804/3	Epithelioid sarcoma
		8805/3	Undifferentiated sarcoma
		8806/3	Desmoplastic small round cell tumor
FIBROMATOUS NEOPLASMS	881	8810/3	Fibrosarcoma, NOS
		8811/3	Fibromyxosarcoma
		8813/3	Fascial fibrosarcoma
		8814/3	Infantile fibrosarcoma
		8815/3	Solitary fibrous tumor, malignant
SARCOMA, NOS	882	8825/3	Myofibroblastic sarcoma
MYOMATOUS NEOPLASMS	889	8890/3	Leiomyosarcoma, NOS
		8891/3	Epithelioid leiomyosarcoma
		8894/3	Angiomyosarcoma
		8895/3	Myosarcoma
		8896/3	Myxoid leiomyosarcoma
RHABDOMYOSARCOMA, NOS	890	8900/3	Rhabdomyosarcoma, NOS
		8901/3	Pleomorphic rhabdomyosarcoma, adult type
		8902/3	Mixed type rhabdomyosarcoma
EMBRYONAL RHABDOMYOSARCOMA	891	8910/3	Embryonal rhabdomyosarcoma
		8912/3	Spindle cell rhabdomyosarcoma

PROSTATE GLAND C619

ALVEOLAR RHABDOMYOSARCOMA	892	8920/3	Alveolar rhabdomyosarcoma
		8921/3	Rhabdomyosarcoma with ganglionic differentiation
CARCINOSARCOMA, NOS	898	8980/3	Carcinosarcoma, NOS
		8981/3	Carcinosarcoma, embryonal type
		8982/3	Malignant myoepithelioma
ML, LARGE B-CELL, DIFFUSE	968	9688/3	T-cell histiocyte rich large B-cell lymphoma
FOLLIC. & MARGINAL LYMPH, NOS	969	9699/3	Marginal zone B-cell lymphoma, NOS
OTHER SPEC. NON-HODGKIN LYMPHOMA	971	9712/3	Intravascular large B-cell lymphoma
PRECURS. CELL LYMPHOBLASTIC LYMPH.	972	9724/3	SystemicEBV pos. T-cell lymphoproliferative disease of childhood
PLASMA CELL TUMORS	973	9735/3	Plasmablastic lymphoma
		9737/3	ALK positive large B-cell lymphoma
		9738/3	Lrg B-cell lymphoma in HHV8-assoc. multicentric Castleman DZ
NEOPLASMS OF HISTIOCYTES AND ACCESSORY LYMPHOID CELLS	975	9751/3	Langerhans cell histiocytosis, NOS
		9759/3	Fibroblastic reticular cell tumor
PRECURSOR LYMPHOID NEOPLASMS	981	9811/3	B lymphoblastic leukemia/lymphoma, NOS
		9812/3	Leukemia/lymphoma with t(9;22)(q34;q11.2);BCR-ABL1
		9813/3	Leukemia/lymphoma with t(v;11q23);MLL rearranged
		9814/3	Leukemia/lymphoma with t(12;21)(p13;q22);TEL-AML1(ETV6-RUNX1)
		9815/3	B lymphoblastic leukemia/lymphoma with hyperdiploidy
		9816/3	Leukemia/lymphoma with hypodiploidy (hypodiploid ALL)
		9817/3	B lymphblastic leukemia/lymphoma with t(5;14)(q31;q32);IL3-IGH
		9818/3	Leukemia/lymphoma with t(1;19)(q23;p13.3); E2A PBX1 (TCF3 PBX1)
LYMPHOID LEUKEMIA, NOS	982	9823/3	Chronic lymphocytic leukemia/small lymphocytic lymphoma
PROLYMPH/PRECURS LEUKEMIA	983	9831/3	T-cell large granular lymphocytic leukemia
		9837/3	T lymphoblastic leukemia/lymphoma
CHRONIC MYELOPROLIFERATIVE DIS.	996	9965/3	Myeloid and lymphoid neoplasms with PDGFRB rearrangement
		9967/3	Myeloid and lymphoid neoplasm with FGFR1 abnormalities
MYELOPLASTIC/MYELOPROLIFERATIVE NEOPLASMS	997	9971/3	Polymorphic PTLD
		9975/3	Myelodysplastic/Myeloproliferative neoplasm, unclassifiable

TESTIS C620-C621,C629

NEOPLASM	800	8000/3	Neoplasm, malignant
		8001/3	Tumor cells, malignant
		8002/3	Malignant tumor, small cell type
		8003/3	Malignant tumor, giant cell type
		8004/3	Malignant tumor, spindle cell type
		8005/3	Malignant tumor, clear cell type
CARCINOMA, NOS	801	8010/2	Carcinoma in situ, NOS
		8010/3	Carcinoma, NOS
		8011/3	Epithelioma, malignant
		8012/3	Large cell carcinoma, NOS
		8013/3	Large cell neuroendocrine carcinoma
		8014/3	Large cell carcinoma with rhabdoid phenotype
		8015/3	Glassy cell carcinoma
CARCINOMA, UNDIFF., NOS	802	8020/3	Carcinoma, undifferentiated type, NOS
		8021/3	Carcinoma, anaplastic type, NOS
		8022/3	Pleomorphic carcinoma
ADENOCARCINOMA, NOS	814	8140/2	Adenocarcinoma in situ
		8140/3	Adenocarcinoma, NOS
		8141/3	Scirrhous adenocarcinoma
		8143/3	Superficial spreading adenocarcinoma
		8147/3	Basal cell adenocarcinoma
BRONCHIOLO-ALVEOLAR ADENOCA.	825	8255/3	Adenocarcinoma with mixed subtypes
PAPILLARY ADENOCARCINOMA, NOS	826	8260/3	Papillary adenocarcinoma, NOS
		8261/2	Adenocarcinoma in situ in villous adenoma
		8261/3	Adenocarcinoma in villous adenoma
		8262/3	Villous adenocarcinoma
		8263/2	Adenocarcinoma in situ in tubulovillous adenoma
		8263/3	Adenocarcinoma in tubulovillous adenoma
GONADAL NEOPLASMS	863	8630/3	Androblastoma, malignant
		8631/3	Sertoli-Leydig cell tumor, poorly differentiated
		8634/3	Sertoli-Leydig cl tum., p.d. w heterologous elements
SERTOLI CELL CARCINOMA	864	8640/3	Sertoli cell carcinoma
LEYDIG CELL TUMOR, MALIGNANT	865	8650/3	Leydig cell tumor, malignant
SARCOMA, NOS	880	8800/3	Sarcoma, NOS
		8801/3	Spindle cell sarcoma
		8802/3	Giant cell sarcoma
		8803/3	Small cell sarcoma
		8804/3	Epithelioid sarcoma

TESTIS C620-C621,C629

SARCOMA, NOS	880	8805/3	Undifferentiated sarcoma
		8806/3	Desmoplastic small round cell tumor
RHABDOMYOSARCOMA, NOS	890	8900/3	Rhabdomyosarcoma, NOS
		8901/3	Pleomorphic rhabdomyosarcoma, adult type
		8902/3	Mixed type rhabdomyosarcoma
EMBRYONAL RHABDOMYOSARCOMA	891	8910/3	Embryonal rhabdomyosarcoma
		8912/3	Spindle cell rhabdomyosarcoma
GERM CELL TUMORS	906	9060/3	Dysgerminoma
		9061/3	Seminoma, NOS
		9062/3	Seminoma, anaplastic
		9063/3	Spermatocytic seminoma
		9064/2	Intratubular malignant germ cells
		9064/3	Germinoma
		9065/3	Germ cell tumor, nonseminomatous
EMBRYONAL CARCINOMA, NOS	907	9070/3	Embryonal carcinoma, NOS
		9071/3	Yolk sac tumor
		9072/3	Polyembryoma
TERATOMA	908	9080/3	Teratoma, malignant, NOS
		9081/3	Teratocarcinoma
		9082/3	Malignant teratoma, undiff.
		9083/3	Malignant teratoma, intermediate
		9084/3	Teratoma with malig. transformation
		9085/3	Mixed germ cell tumor
CHORIOCARCINOMA	910	9100/3	Choriocarcinoma
		9101/3	Choriocarcinoma combined w/ other germ cell elements
		9102/3	Malignant teratoma, trophoblastic
		9105/3	Trophoblastic tumor, epithelioid
MALIGNANT LYMPHOMA, NOS	959	9590/3	Malignant lymphoma, NOS
		9591/3	Malignant lymphoma, non-Hodgkin
		9596/3	Composite Hodgkin and non-Hodgkin lymphoma
HODGKIN LYMPHOMA	965	9650/3	Hodgkin lymphoma, NOS
		9651/3	Hodgkin lymphoma, lymphocyte-rich
		9652/3	Hodgkin lymphoma, mixed cellularity, NOS
		9653/3	Hodgkin lymphoma, lymphocytic deplet., NOS
		9654/3	Hodgkin lymph., lymphocyt. deplet., diffuse fibrosis
		9655/3	Hodgkin lymphoma, lymphocyt. deplet., reticular
		9659/3	Hodgkin lymph., nodular lymphocyte predom.

TESTIS C620-C621,C629

HODGKIN LYMPHOMA, NOD. SCLER.	966	9661/3	Hodgkin granuloma [obs]
		9662/3	Hodgkin sarcoma [obs]
		9663/3	Hodgkin lymphoma, nodular sclerosis, NOS
		9664/3	Hodgkin lymphoma, nod. scler., cellular phase
		9665/3	Hodgkin lymphoma, nod. scler., grade 1
		9667/3	Hodgkin lymphoma, nod. scler., grade 2
ML, SMALL B-CELL LYMPHOCYTIC	967	9670/3	ML, small B lymphocytic, NOS
		9671/3	ML, lymphoplasmacytic
		9673/3	Mantle cell lymphoma
		9675/3	ML, mixed sm. and lg. cell, diffuse
ML, LARGE B-CELL, DIFFUSE	968	9680/3	ML, large B-cell, diffuse
		9684/3	ML, large B-cell, diffuse, immunoblastic, NOS
		9687/3	Burkitt lymphoma, NOS
		9688/3	T-cell histiocyte rich large B-cell lymphoma
FOLLIC. & MARGINAL LYMPH, NOS	969	9690/3	Follicular lymphoma, NOS
		9691/3	Follicular lymphoma, grade 2
		9695/3	Follicular lymphoma, grade 1
		9698/3	Follicular lymphoma, grade 3
		9699/3	Marginal zone B-cell lymphoma, NOS
T-CELL LYMPHOMAS	970	9701/3	Sezary syndrome
		9702/3	Mature T-cell lymphoma, NOS
		9705/3	Angioimmunoblastic T-cell lymphoma
OTHER SPEC. NON-HODGKIN LYMPHOMA	971	9712/3	Intravascular large B-cell lymphoma
		9714/3	Anaplastic large cell lymphoma, T-cell and Null cell type
		9719/3	NK/T-cell lymphoma, nasal and nasal-type
PRECURS. CELL LYMPHOBLASTIC LYMPH.	972	9724/3	SystemicEBV pos. T-cell lymphoproliferative disease of childhood
		9727/3	Precursor cell lymphoblastic lymphoma, NOS
		9728/3	Precursor B-cell lymphoblastic lymphoma
		9729/3	Precursor T-cell lymphoblastic lymphoma
PLASMA CELL TUMORS	973	9731/3	Plasmacytoma, NOS
		9734/3	Plasmacytoma, extramedullary
		9735/3	Plasmablastic lymphoma
		9737/3	ALK positive large B-cell lymphoma
		9738/3	Lrg B-cell lymphoma in HHV8-assoc. multicentric Castleman DZ
MAST CELL TUMORS	974	9740/3	Mast cell sarcoma
		9741/3	Malignant mastocytosis

TESTIS C620-C621,C629

NEOPLASMS OF HISTIOCYTES AND ACCESSORY LYMPHOID CELLS	975	9750/3	Malignant histiocytosis
		9751/3	Langerhans cell histiocytosis, NOS
		9754/3	Langerhans cell histiocytosis, disseminated
		9755/3	Histiocytic sarcoma
		9756/3	Langerhans cell sarcoma
		9757/3	Interdigitating dendritic cell sarcoma
		9758/3	Follicular dendritic cell sarcoma
		9759/3	Fibroblastic reticular cell tumor
PRECURSOR LYMPHOID NEOPLASMS	981	9811/3	B lymphoblastic leukemia/lymphoma, NOS
		9812/3	Leukemia/lymphoma with t(9;22)(q34;q11.2);BCR-ABL1
		9813/3	Leukemia/lymphoma with t(v;11q23);MLL rearranged
		9814/3	Leukemia/lymphoma with t(12;21)(p13;q22);TEL-AML1(ETV6-RUNX1)
		9815/3	B lymphoblastic leukemia/lymphoma with hyperdiploidy
		9816/3	Leukemia/lymphoma with hypodiploidy (hypodiploid ALL)
		9817/3	B lymphblastic leukemia/lymphoma with t(5;14)(q31;q32);IL3-IGH
		9818/3	Leukemia/lymphoma with t(1;19)(q23;p13.3); E2A PBX1 (TCF3 PBX1)
LYMPHOID LEUKEMIA, NOS	982	9823/3	Chronic lymphocytic leukemia/small lymphocytic lymphoma
PROLYMPH/PRECURS LEUKEMIA	983	9831/3	T-cell large granular lymphocytic leukemia
		9837/3	T lymphoblastic leukemia/lymphoma
CHRONIC MYELOPROLIFERATIVE DIS.	996	9965/3	Myeloid and lymphoid neoplasms with PDGFRB rearrangement
		9967/3	Myeloid and lymphoid neoplasm with FGFR1 abnormalities
MYELOPLASTIC/MYELOPROLIFERATIVE NEOPLASMS	997	9971/3	Polymorphic PTLD
		9975/3	Myelodysplastic/Myeloproliferative neoplasm, unclassifiable

EPIDIDYMIS, SPERMATIC CORD, MALE GENITAL, NOS C630, C631, C637-C639

NEOPLASM	800	8000/3	Neoplasm, malignant
		8001/3	Tumor cells, malignant
		8002/3	Malignant tumor, small cell type
		8003/3	Malignant tumor, giant cell type
		8004/3	Malignant tumor, spindle cell type
		8005/3	Malignant tumor, clear cell type
CARCINOMA, NOS	801	8010/2	Carcinoma in situ, NOS
		8010/3	Carcinoma, NOS
		8011/3	Epithelioma, malignant
		8012/3	Large cell carcinoma, NOS
		8013/3	Large cell neuroendocrine carcinoma
		8014/3	Large cell carcinoma with rhabdoid phenotype
		8015/3	Glassy cell carcinoma
CARCINOMA, UNDIFF., NOS	802	8020/3	Carcinoma, undifferentiated type, NOS
		8021/3	Carcinoma, anaplastic type, NOS
		8022/3	Pleomorphic carcinoma
PAPILLARY CARCINOMA, NOS	805	8050/2	Papillary carcinoma in situ
		8050/3	Papillary carcinoma, NOS
		8051/3	Verrucous carcinoma, NOS
		8052/2	Papillary squamous cell carcinoma, non-invasive
		8052/3	Papillary squamous cell carcinoma
SQUAMOUS CELL CARCINOMA, NOS	807	8070/2	Squamous cell carcinoma in situ, NOS
		8070/3	Squamous cell carcinoma, NOS
		8071/3	Sq. cell carcinoma, keratinizing, NOS
		8072/3	Sq. cell carcinoma, lg. cell, non-ker.
		8073/3	Sq. cell carcinoma, sm. cell, non-ker.
		8074/3	Sq. cell carcinoma, spindle cell
		8075/3	Squamous cell carcinoma, adenoid
		8076/2	Sq. cell carc. in situ with question. stromal invas.
		8076/3	Sq. cell carcinoma, micro-invasive
		8078/3	Squamous cell carcinoma with horn formation
TRANSITIONAL CELL CARCINOMA, NOS	812	8120/2	Transitional cell carcinoma in situ
		8120/3	Transitional cell carcinoma, NOS
		8121/3	Schneiderian carcinoma
		8122/3	Trans. cell carcinoma, spindle cell
		8123/3	Basaloid carcinoma
		8124/3	Cloacogenic carcinoma

EPIDIDYMIS, SPERMATIC CORD, MALE GENITAL, NOS C630, C631, C637-C639

ADENOCARCINOMA, NOS	814	8140/2	Adenocarcinoma in situ
		8140/3	Adenocarcinoma, NOS
		8141/3	Scirrhous adenocarcinoma
		8143/3	Superficial spreading adenocarcinoma
		8147/3	Basal cell adenocarcinoma
BRONCHIOLO-ALVEOLAR ADENOCA.	825	8255/3	Adenocarcinoma with mixed subtypes
PAPILLARY ADENOCARCINOMA, NOS	826	8260/3	Papillary adenocarcinoma, NOS
		8261/2	Adenocarcinoma in situ in villous adenoma
		8261/3	Adenocarcinoma in villous adenoma
		8262/3	Villous adenocarcinoma
		8263/2	Adenocarcinoma in situ in tubulovillous adenoma
		8263/3	Adenocarcinoma in tubulovillous adenoma
SWEAT GLAND ADENOCARCINOMA	840	8401/3	Apocrine adenocarcinoma
PAGET DISEASE, EXTRAMAMMARY	854	8542/3	Paget disease, extramammary
ADENOCA. WITH METAPLASIA	857	8570/3	Adenocarcinoma with squamous metaplasia
		8571/3	Adenocarcinoma w cartilag. & oss. metaplas.
		8572/3	Adenocarcinoma with spindle cell mataplasia
		8573/3	Adenocarcinoma with apocrine metaplasia
		8574/3	Adenocarcinoma with neuroendocrine differen.
		8575/3	Metaplastic carcinoma, NOS
SARCOMA, NOS	880	8800/3	Sarcoma, NOS
		8801/3	Spindle cell sarcoma
		8802/3	Giant cell sarcoma
		8803/3	Small cell sarcoma
		8804/3	Epithelioid sarcoma
		8805/3	Undifferentiated sarcoma
		8806/3	Desmoplastic small round cell tumor
FIBROMATOUS NEOPLASMS	881	8810/3	Fibrosarcoma, NOS
		8811/3	Fibromyxosarcoma
		8813/3	Fascial fibrosarcoma
		8814/3	Infantile fibrosarcoma
		8815/3	Solitary fibrous tumor, malignant
SARCOMA, NOS	882	8825/3	Myofibroblastic sarcoma
FIBROUS HISTIOCYTOMA, MAL.	883	8830/3	Fibrous histiocytoma, malignant

EPIDIDYMIS, SPERMATIC CORD, MALE GENITAL, NOS C630, C631, C637-C639

LIPOSARCOMA NEOPLASMS	885	8850/3	Liposarcoma, NOS
		8851/3	Liposarcoma, well differentiated
		8852/3	Myxoid liposarcoma
		8853/3	Round cell liposarcoma
		8854/3	Pleomorphic liposarcoma
		8855/3	Mixed type liposarcoma
		8857/3	Fibroblastic liposarcoma
		8858/3	Dedifferentiated liposarcoma
MYOMATOUS NEOPLASMS	889	8890/3	Leiomyosarcoma, NOS
		8891/3	Epithelioid leiomyosarcoma
		8894/3	Angiomyosarcoma
		8895/3	Myosarcoma
		8896/3	Myxoid leiomyosarcoma
RHABDOMYOSARCOMA, NOS	890	8900/3	Rhabdomyosarcoma, NOS
		8901/3	Pleomorphic rhabdomyosarcoma, adult type
		8902/3	Mixed type rhabdomyosarcoma
EMBRYONAL RHABDOMYOSARCOMA	891	8910/3	Embryonal rhabdomyosarcoma
		8912/3	Spindle cell rhabdomyosarcoma
ALVEOLAR RHABDOMYOSARCOMA	892	8920/3	Alveolar rhabdomyosarcoma
		8921/3	Rhabdomyosarcoma with ganglionic differentiation
MULLERIAN MIXED TUMOR	895	8950/3	Mullerian mixed tumor
		8951/3	Mesodermal mixed tumor
CARCINOSARCOMA, NOS	898	8980/3	Carcinosarcoma, NOS
		8981/3	Carcinosarcoma, embryonal type
		8982/3	Malignant myoepithelioma
MESENCHYMOMA, MALIGNANT	899	8990/3	Mesenchymoma, malignant
		8991/3	Embryonal sarcoma
KAPOSI SARCOMA	914	9140/3	Kaposi sarcoma
FOLLIC. & MARGINAL LYMPH, NOS	969	9699/3	Marginal zone B-cell lymphoma, NOS
LYMPHOID LEUKEMIA, NOS	982	9823/3	Chronic lymphocytic leukemia/small lymphocytic lymphoma

SCROTUM C632

NEOPLASM	800	8000/3	Neoplasm, malignant
		8001/3	Tumor cells, malignant
		8002/3	Malignant tumor, small cell type
		8003/3	Malignant tumor, giant cell type
		8004/3	Malignant tumor, spindle cell type
		8005/3	Malignant tumor, clear cell type
CARCINOMA, NOS	801	8010/2	Carcinoma in situ, NOS
		8010/3	Carcinoma, NOS
		8011/3	Epithelioma, malignant
		8012/3	Large cell carcinoma, NOS
		8013/3	Large cell neuroendocrine carcinoma
		8014/3	Large cell carcinoma with rhabdoid phenotype
		8015/3	Glassy cell carcinoma
CARCINOMA, UNDIFF., NOS	802	8020/3	Carcinoma, undifferentiated type, NOS
		8021/3	Carcinoma, anaplastic type, NOS
		8022/3	Pleomorphic carcinoma
PAPILLARY CARCINOMA, NOS	805	8050/2	Papillary carcinoma in situ
		8050/3	Papillary carcinoma, NOS
		8051/3	Verrucous carcinoma, NOS
		8052/2	Papillary squamous cell carcinoma, non-invasive
		8052/3	Papillary squamous cell carcinoma
SQUAMOUS CELL CARCINOMA, NOS	807	8070/2	Squamous cell carcinoma in situ, NOS
		8070/3	Squamous cell carcinoma, NOS
		8071/3	Sq. cell carcinoma, keratinizing, NOS
		8072/3	Sq. cell carcinoma, lg. cell, non-ker.
		8073/3	Sq. cell carcinoma, sm. cell, non-ker.
		8074/3	Sq. cell carcinoma, spindle cell
		8075/3	Squamous cell carcinoma, adenoid
		8076/2	Sq. cell carc. in situ with question. stromal invas.
		8076/3	Sq. cell carcinoma, micro-invasive
		8078/3	Squamous cell carcinoma with horn formation
LYMPHOEPITHELIAL CARCINOMA	808	8080/2	Queyrat erythroplasia
		8081/2	Bowen disease
		8082/3	Lymphoepithelial carcinoma
		8083/3	Basaloid squamous cell carcinoma
		8084/3	Squamous cell carcinoma, clear cell type
BASAL CELL CARCINOMA, NOS	809	8090/3	Basal cell carcinoma, NOS
		8091/3	Multifocal superficial basal cell carcinoma
		8092/3	Infiltrating basal cell carcinoma, NOS
		8093/3	Basal cell carcinoma, fibroepithelial
		8094/3	Basosquamous carcinoma

SCROTUM C632

BASAL CELL CARCINOMA, NOS	809	8095/3	Metatypical carcinoma
		8097/3	Basal cell carcinoma, nodular
		8098/3	Adenoid basal cell carcinoma
TRICHILEMMOCARCINOMA	810	8102/3	Trichilemmocarcinoma
PILOMATRIX CARCINOMA	811	8110/3	Pilomatrix carcinoma
TRANSITIONAL CELL CARCINOMA, NOS	812	8120/2	Transitional cell carcinoma in situ
		8120/3	Transitional cell carcinoma, NOS
		8121/3	Schneiderian carcinoma
		8122/3	Trans. cell carcinoma, spindle cell
		8123/3	Basaloid carcinoma
		8124/3	Cloacogenic carcinoma
ADENOCARCINOMA, NOS	814	8140/2	Adenocarcinoma in situ
		8140/3	Adenocarcinoma, NOS
		8141/3	Scirrhous adenocarcinoma
		8143/3	Superficial spreading adenocarcinoma
		8147/3	Basal cell adenocarcinoma
BRONCHIOLO-ALVEOLAR ADENOCA.	825	8255/3	Adenocarcinoma with mixed subtypes
PAPILLARY ADENOCARCINOMA, NOS	826	8260/3	Papillary adenocarcinoma, NOS
		8261/2	Adenocarcinoma in situ in villous adenoma
		8261/3	Adenocarcinoma in villous adenoma
		8262/3	Villous adenocarcinoma
		8263/2	Adenocarcinoma in situ in tubulovillous adenoma
		8263/3	Adenocarcinoma in tubulovillous adenoma
SKIN APPENDAGE CARCINOMA	839	8390/3	Skin appendage carcinoma
SWEAT GLAND ADENOCARCINOMA	840	8400/3	Sweat gland adenocarcinoma
		8401/3	Apocrine adenocarcinoma
SEBACEOUS/ECCRINE ADENOCA.	841	8410/3	Sebaceous adenocarcinoma
		8413/3	Eccrine adenocarcinoma
PAGET DISEASE, EXTRAMAMMARY	854	8542/3	Paget disease, extramammary
ADENOCA. WITH METAPLASIA	857	8570/3	Adenocarcinoma with squamous metaplasia
		8571/3	Adenocarcinoma w cartilag. & oss. metaplas.
		8572/3	Adenocarcinoma with spindle cell mataplasia
		8573/3	Adenocarcinoma with apocrine metaplasia
		8574/3	Adenocarcinoma with neuroendocrine differen.
		8575/3	Metaplastic carcinoma, NOS

SCROTUM C632

NEVI & MELANOMAS	872	8720/2	Melanoma in situ
		8720/3	Malignant melanoma, NOS
		8721/3	Nodular melanoma
		8722/3	Balloon cell melanoma
		8723/3	Malignant melanoma, regressing
AMELANOTIC MELANOMA	873	8730/3	Amelanotic melanoma
MAL. MEL. IN JUNCT. NEVUS	874	8740/3	Mal. melanoma in junctional nevus
		8741/2	Precancerous melanosis, NOS
		8741/3	Mal. melanoma in precan. melanosis
		8742/2	Lentigo maligna
		8742/3	Lentigo maligna melanoma
		8743/3	Superficial spreading melanoma
		8745/3	Desmoplastic melanoma, malignant
		8746/3	Mucosal lentiginous melanoma
MAL. MELAN. IN GIANT PIGMT. NEVUS	876	8761/3	Mal. melanoma in giant pigmented nevus
EPITHELIOID CELL MELANOMA	877	8770/3	Mixed epithel. & spindle cell melanoma
		8771/3	Epithelioid cell melanoma
		8772/3	Spindle cell melanoma, NOS
BLUE NEVUS, MALIGNANT	878	8780/3	Blue nevus, malignant
SARCOMA, NOS	880	8800/3	Sarcoma, NOS
		8801/3	Spindle cell sarcoma
		8802/3	Giant cell sarcoma
		8803/3	Small cell sarcoma
		8804/3	Epithelioid sarcoma
		8805/3	Undifferentiated sarcoma
		8806/3	Desmoplastic small round cell tumor
FIBROMATOUS NEOPLASMS	881	8810/3	Fibrosarcoma, NOS
		8811/3	Fibromyxosarcoma
		8813/3	Fascial fibrosarcoma
		8814/3	Infantile fibrosarcoma
		8815/3	Solitary fibrous tumor, malignant
SARCOMA, NOS	882	8825/3	Myofibroblastic sarcoma
FIBROUS HISTIOCYTOMA, MAL.	883	8830/3	Fibrous histiocytoma, malignant

SCROTUM C632

LIPOSARCOMA NEOPLASMS	885	8850/3	Liposarcoma, NOS
		8851/3	Liposarcoma, well differentiated
		8852/3	Myxoid liposarcoma
		8853/3	Round cell liposarcoma
		8854/3	Pleomorphic liposarcoma
		8855/3	Mixed type liposarcoma
		8857/3	Fibroblastic liposarcoma
		8858/3	Dedifferentiated liposarcoma
MYOMATOUS NEOPLASMS	889	8890/3	Leiomyosarcoma, NOS
		8891/3	Epithelioid leiomyosarcoma
		8894/3	Angiomyosarcoma
		8895/3	Myosarcoma
		8896/3	Myxoid leiomyosarcoma
RHABDOMYOSARCOMA, NOS	890	8900/3	Rhabdomyosarcoma, NOS
		8901/3	Pleomorphic rhabdomyosarcoma, adult type
		8902/3	Mixed type rhabdomyosarcoma
EMBRYONAL RHABDOMYOSARCOMA	891	8910/3	Embryonal rhabdomyosarcoma
		8912/3	Spindle cell rhabdomyosarcoma
ALVEOLAR RHABDOMYOSARCOMA	892	8920/3	Alveolar rhabdomyosarcoma
		8921/3	Rhabdomyosarcoma with ganglionic differentiation
MULLERIAN MIXED TUMOR	895	8950/3	Mullerian mixed tumor
		8951/3	Mesodermal mixed tumor
CARCINOSARCOMA, NOS	898	8980/3	Carcinosarcoma, NOS
		8981/3	Carcinosarcoma, embryonal type
		8982/3	Malignant myoepithelioma
MESENCHYMOMA, MALIGNANT	899	8990/3	Mesenchymoma, malignant
		8991/3	Embryonal sarcoma
KAPOSI SARCOMA	914	9140/3	Kaposi sarcoma
MALIGNANT LYMPHOMA, NOS	959	9597/3	Primary Cutaneous follicle centre lymphoma
FOLLIC. & MARGINAL LYMPH, NOS	969	9699/3	Marginal zone B-cell lymphoma, NOS
T-CELL LYMPHOMAS	970	9700/3	Mycosis fungoides
		9701/3	Sezary syndrome
		9708/3	Subcutaneous panniculitis-like T-cell lymphoma
		9709/3	Cutaneous T-cell lymphoma, NOS
OTHER SPEC. NON-HODGKIN LYMPHOMA	971	9718/3	Primary cutan. CD30+ T-cell lymphoprolif. disorder

SCROTUM C632

PRECURS. CELL LYMPHOBLASTIC LYMPH.	972	9725/3	Hydroa vacciniforme-like lymphoma
		9726/3	Primary Cutaneous gamma-delta T-cell lymphoma
LYMPHOID LEUKEMIA, NOS	982	9823/3	Chronic lymphocytic leukemia/small lymphocytic lymphoma

KIDNEY C649

NEOPLASM	800	8000/3	Neoplasm, malignant
		8001/3	Tumor cells, malignant
		8002/3	Malignant tumor, small cell type
		8003/3	Malignant tumor, giant cell type
		8004/3	Malignant tumor, spindle cell type
		8005/3	Malignant tumor, clear cell type
CARCINOMA, NOS	801	8010/2	Carcinoma in situ, NOS
		8010/3	Carcinoma, NOS
		8011/3	Epithelioma, malignant
		8012/3	Large cell carcinoma, NOS
		8013/3	Large cell neuroendocrine carcinoma
		8014/3	Large cell carcinoma with rhabdoid phenotype
		8015/3	Glassy cell carcinoma
CARCINOMA, UNDIFF., NOS	802	8020/3	Carcinoma, undifferentiated type, NOS
		8021/3	Carcinoma, anaplastic type, NOS
		8022/3	Pleomorphic carcinoma
GIANT & SPINDLE CELL CARCINOMA	803	8030/3	Giant cell and spindle cell carcinoma
		8031/3	Giant cell carcinoma
		8032/3	Spindle cell carcinoma
		8033/3	Pseudosarcomatous carcinoma
		8034/3	Polygonal cell carcinoma
		8035/3	Carcinoma with osteoclast-like giant cells
SMALL CELL CARCINOMA, NOS	804	8041/3	Small cell carcinoma, NOS
		8043/3	Small cell carcinoma, fusiform cell
PAPILLARY CARCINOMA, NOS	805	8050/2	Papillary carcinoma in situ
		8050/3	Papillary carcinoma, NOS
		8051/3	Verrucous carcinoma, NOS
		8052/2	Papillary squamous cell carcinoma, non-invasive
		8052/3	Papillary squamous cell carcinoma
SQUAMOUS CELL CARCINOMA, NOS	807	8070/2	Squamous cell carcinoma in situ, NOS
		8070/3	Squamous cell carcinoma, NOS
		8071/3	Sq. cell carcinoma, keratinizing, NOS
		8072/3	Sq. cell carcinoma, lg. cell, non-ker.
		8073/3	Sq. cell carcinoma, sm. cell, non-ker.
		8074/3	Sq. cell carcinoma, spindle cell
		8075/3	Squamous cell carcinoma, adenoid
		8076/2	Sq. cell carc. in situ with question. stromal invas.
		8076/3	Sq. cell carcinoma, micro-invasive
		8078/3	Squamous cell carcinoma with horn formation

KIDNEY C649

TRANSITIONAL CELL CARCINOMA, NOS	812	8120/2	Transitional cell carcinoma in situ
		8120/3	Transitional cell carcinoma, NOS
		8121/3	Schneiderian carcinoma
		8122/3	Trans. cell carcinoma, spindle cell
		8123/3	Basaloid carcinoma
		8124/3	Cloacogenic carcinoma
PAPILLARY TRANS. CELL CARCINOMA	813	8130/2	Papillary trans. cell carcinoma, non-invasive
		8130/3	Papillary trans. cell carcinoma
		8131/3	Transitional cell carcinoma, micropapillary
ADENOCARCINOMA, NOS	814	8140/2	Adenocarcinoma in situ
		8140/3	Adenocarcinoma, NOS
		8141/3	Scirrhous adenocarcinoma
		8143/3	Superficial spreading adenocarcinoma
		8147/3	Basal cell adenocarcinoma
TRABECULAR ADENOCARCINOMA	819	8190/3	Trabecular adenocarcinoma
ADENOCA. IN ADENOMA. POLYP	821	8211/3	Tubular adenocarcinoma
SOLID CARCINOMA, NOS	823	8230/2	Duct carcinoma in situ, solid type
		8230/3	Solid carcinoma, NOS
		8231/3	Carcinoma simplex
BRONCHIOLO-ALVEOLAR ADENOCA.	825	8255/3	Adenocarcinoma with mixed subtypes
PAPILLARY ADENOCARCINOMA, NOS	826	8260/3	Papillary adenocarcinoma, NOS
		8261/2	Adenocarcinoma in situ in villous adenoma
CLEAR CELL ADENOCARCINOMA, NOS	831	8310/3	Clear cell adenocarcinoma, NOS
		8311/3	Hereditary leiomyomatosis and RCC-associated renal cell carcinoma
		8312/3	Renal cell carcinoma
		8316/3	Cyst-associated renal cell carcinoma
		8317/3	Renal cell carcinoma, chromophobe type
		8318/3	Renal cell carcinoma, sarcomatoid
		8319/3	Collecting duct carcinoma
GRANULAR CELL CARCINOMA	832	8320/3	Granular cell carcinoma
		8323/3	Mixed cell adenocarcinoma
CYSTADENOCARCINOMA, NOS	844	8440/3	Cystadenocarcinoma, NOS
MUCINOUS ADENOCARCINOMA	848	8480/3	Mucinous adenocarcinoma
		8481/3	Mucin-producing adenocarcinoma

KIDNEY C649

ADENOSQUAMOUS CARCINOMA	856	8560/3	Adenosquamous carcinoma
		8562/3	Epithelial-myoepithelial carcinoma
SARCOMA, NOS	880	8800/3	Sarcoma, NOS
		8801/3	Spindle cell sarcoma
		8802/3	Giant cell sarcoma
		8803/3	Small cell sarcoma
		8804/3	Epithelioid sarcoma
		8805/3	Undifferentiated sarcoma
		8806/3	Desmoplastic small round cell tumor
FIBROMATOUS NEOPLASMS	881	8810/3	Fibrosarcoma, NOS
		8811/3	Fibromyxosarcoma
		8813/3	Fascial fibrosarcoma
		8814/3	Infantile fibrosarcoma
		8815/3	Solitary fibrous tumor, malignant
SARCOMA, NOS	882	8825/3	Myofibroblastic sarcoma
FIBROUS HISTIOCYTOMA, MAL.	883	8830/3	Fibrous histiocytoma, malignant
LIPOSARCOMA NEOPLASMS	885	8850/3	Liposarcoma, NOS
		8851/3	Liposarcoma, well differentiated
		8852/3	Myxoid liposarcoma
		8853/3	Round cell liposarcoma
		8854/3	Pleomorphic liposarcoma
		8855/3	Mixed type liposarcoma
		8857/3	Fibroblastic liposarcoma
		8858/3	Dedifferentiated liposarcoma
MYOMATOUS NEOPLASMS	889	8890/3	Leiomyosarcoma, NOS
		8891/3	Epithelioid leiomyosarcoma
		8894/3	Angiomyosarcoma
		8895/3	Myosarcoma
		8896/3	Myxoid leiomyosarcoma
RHABDOMYOSARCOMA, NOS	890	8900/3	Rhabdomyosarcoma, NOS
		8901/3	Pleomorphic rhabdomyosarcoma, adult type
		8902/3	Mixed type rhabdomyosarcoma
EMBRYONAL RHABDOMYOSARCOMA	891	8910/3	Embryonal rhabdomyosarcoma
		8912/3	Spindle cell rhabdomyosarcoma

KIDNEY C649

MULLERIAN MIXED TUMOR	895	8950/3	Mullerian mixed tumor
		8951/3	Mesodermal mixed tumor
		8959/3	Malignant cystic nephroma
CLEAR CELL SARC/NEPHROBLASTOMA	896	8960/3	Nephroblastoma, NOS
		8963/3	Malignant rhabdoid tumor
		8964/3	Clear cell sarcoma of kidney
CARCINOSARCOMA, NOS	898	8980/3	Carcinosarcoma, NOS
		8981/3	Carcinosarcoma, embryonal type
		8982/3	Malignant myoepithelioma
MESENCHYMOMA, MALIGNANT	899	8990/3	Mesenchymoma, malignant
		8991/3	Embryonal sarcoma
NEUROBLASTOMA, NOS	950	9500/3	Neuroblastoma, NOS
		9501/3	Medulloepithelioma, NOS
		9502/3	Teratoid medulloepithelioma
		9503/3	Neuroepithelioma, NOS
		9504/3	Spongioneuroblastoma
		9505/3	Ganglioglioma, anaplastic
MALIGNANT LYMPHOMA, NOS	959	9590/3	Malignant lymphoma, NOS
		9591/3	Malignant lymphoma, non-Hodgkin
		9596/3	Composite Hodgkin and non-Hodgkin lymphoma
HODGKIN LYMPHOMA	965	9650/3	Hodgkin lymphoma, NOS
		9651/3	Hodgkin lymphoma, lymphocyte-rich
		9652/3	Hodgkin lymphoma, mixed cellularity, NOS
		9653/3	Hodgkin lymphoma, lymphocytic deplet., NOS
		9654/3	Hodgkin lymph., lymphocyt. deplet., diffuse fibrosis
		9655/3	Hodgkin lymphoma, lymphocyt. deplet., reticular
		9659/3	Hodgkin lymph., nodular lymphocyte predom.
HODGKIN LYMPHOMA, NOD. SCLER.	966	9661/3	Hodgkin granuloma [obs]
		9662/3	Hodgkin sarcoma [obs]
		9663/3	Hodgkin lymphoma, nodular sclerosis, NOS
		9664/3	Hodgkin lymphoma, nod. scler., cellular phase
		9665/3	Hodgkin lymphoma, nod. scler., grade 1
		9667/3	Hodgkin lymphoma, nod. scler., grade 2
ML, SMALL B-CELL LYMPHOCYTIC	967	9670/3	ML, small B lymphocytic, NOS
		9671/3	ML, lymphoplasmacytic
		9673/3	Mantle cell lymphoma
		9675/3	ML, mixed sm. and lg. cell, diffuse

KIDNEY C649

ML, LARGE B-CELL, DIFFUSE	968	9680/3	ML, large B-cell, diffuse
		9684/3	ML, large B-cell, diffuse, immunoblastic, NOS
		9687/3	Burkitt lymphoma, NOS
		9688/3	T-cell histiocyte rich large B-cell lymphoma
FOLLIC. & MARGINAL LYMPH, NOS	969	9690/3	Follicular lymphoma, NOS
		9691/3	Follicular lymphoma, grade 2
		9695/3	Follicular lymphoma, grade 1
		9698/3	Follicular lymphoma, grade 3
		9699/3	Marginal zone B-cell lymphoma, NOS
T-CELL LYMPHOMAS	970	9701/3	Sezary syndrome
		9702/3	Mature T-cell lymphoma, NOS
		9705/3	Angioimmunoblastic T-cell lymphoma
OTHER SPEC. NON-HODGKIN LYMPHOMA	971	9712/3	Intravascular large B-cell lymphoma
		9714/3	Anaplastic large cell lymphoma, T-cell and Null cell type
		9719/3	NK/T-cell lymphoma, nasal and nasal-type
PRECURS. CELL LYMPHOBLASTIC LYMPH.	972	9724/3	SystemicEBV pos. T-cell lymphoproliferative disease of childhood
		9727/3	Precursor cell lymphoblastic lymphoma, NOS
		9728/3	Precursor B-cell lymphoblastic lymphoma
		9729/3	Precursor T-cell lymphoblastic lymphoma
PLASMA CELL TUMORS	973	9731/3	Plasmacytoma, NOS
		9734/3	Plasmacytoma, extramedullary
		9735/3	Plasmablastic lymphoma
		9737/3	ALK positive large B-cell lymphoma
		9738/3	Lrg B-cell lymphoma in HHV8-assoc. multicentric Castleman DZ
MAST CELL TUMORS	974	9740/3	Mast cell sarcoma
		9741/3	Malignant mastocytosis
NEOPLASMS OF HISTIOCYTES AND ACCESSORY LYMPHOID CELLS	975	9750/3	Malignant histiocytosis
		9751/3	Langerhans cell histiocytosis, NOS
		9754/3	Langerhans cell histiocytosis, disseminated
		9755/3	Histiocytic sarcoma
		9756/3	Langerhans cell sarcoma
		9757/3	Interdigitating dendritic cell sarcoma
		9758/3	Follicular dendritic cell sarcoma
		9759/3	Fibroblastic reticular cell tumor
PRECURSOR LYMPHOID NEOPLASMS	981	9811/3	B lymphoblastic leukemia/lymphoma, NOS
		9812/3	Leukemia/lymphoma with t(9;22)(q34;q11.2);BCR-ABL1
		9813/3	Leukemia/lymphoma with t(v;11q23);MLL rearranged
		9814/3	Leukemia/lymphoma with t(12;21)(p13;q22);TEL-AML1(ETV6-RUNX1)
		9815/3	B lymphoblastic leukemia/lymphoma with hyperdiploidy

KIDNEY C649

PRECURSOR LYMPHOID NEOPLASMS	981	9816/3	Leukemia/lymphoma with hypodiploidy (hypodiploid ALL)
		9817/3	B lymphblastic leukemia/lymphoma with t(5;14)(q31;q32);IL3-IGH
		9818/3	Leukemia/lymphoma with t(1;19)(q23;p13.3); E2A PBX1 (TCF3 PBX1)
LYMPHOID LEUKEMIA, NOS	982	9823/3	Chronic lymphocytic leukemia/small lymphocytic lymphoma
PROLYMPH/PRECURS LEUKEMIA	983	9831/3	T-cell large granular lymphocytic leukemia
		9837/3	T lymphoblastic leukemia/lymphoma
CHRONIC MYELOPROLIFERATIVE DIS.	996	9965/3	Myeloid and lymphoid neoplasms with PDGFRB rearrangement
		9967/3	Myeloid and lymphoid neoplasm with FGFR1 abnormalities
MYELOPLASTIC/MYELOPROLIFERATIVE NEOPLASMS	997	9971/3	Polymorphic PTLD
		9975/3	Myelodysplastic/Myeloproliferative neoplasm, unclassifiable

RENAL PELVIS, URETER C659, C669

NEOPLASM	800	8000/3	Neoplasm, malignant
		8001/3	Tumor cells, malignant
		8002/3	Malignant tumor, small cell type
		8003/3	Malignant tumor, giant cell type
		8004/3	Malignant tumor, spindle cell type
		8005/3	Malignant tumor, clear cell type
CARCINOMA, NOS	801	8010/2	Carcinoma in situ, NOS
		8010/3	Carcinoma, NOS
		8011/3	Epithelioma, malignant
		8012/3	Large cell carcinoma, NOS
		8013/3	Large cell neuroendocrine carcinoma
		8014/3	Large cell carcinoma with rhabdoid phenotype
		8015/3	Glassy cell carcinoma
CARCINOMA, UNDIFF., NOS	802	8020/3	Carcinoma, undifferentiated type, NOS
		8021/3	Carcinoma, anaplastic type, NOS
		8022/3	Pleomorphic carcinoma
GIANT & SPINDLE CELL CARCINOMA	803	8030/3	Giant cell and spindle cell carcinoma
		8031/3	Giant cell carcinoma
		8032/3	Spindle cell carcinoma
		8033/3	Pseudosarcomatous carcinoma
		8034/3	Polygonal cell carcinoma
		8035/3	Carcinoma with osteoclast-like giant cells
SMALL CELL CARCINOMA, NOS	804	8041/3	Small cell carcinoma, NOS
		8043/3	Small cell carcinoma, fusiform cell
PAPILLARY CARCINOMA, NOS	805	8050/2	Papillary carcinoma in situ
		8050/3	Papillary carcinoma, NOS
		8051/3	Verrucous carcinoma, NOS
		8052/2	Papillary squamous cell carcinoma, non-invasive
		8052/3	Papillary squamous cell carcinoma
SQUAMOUS CELL CARCINOMA, NOS	807	8070/2	Squamous cell carcinoma in situ, NOS
		8070/3	Squamous cell carcinoma, NOS
		8071/3	Sq. cell carcinoma, keratinizing, NOS
		8072/3	Sq. cell carcinoma, lg. cell, non-ker.
		8073/3	Sq. cell carcinoma, sm. cell, non-ker.
		8074/3	Sq. cell carcinoma, spindle cell
		8075/3	Squamous cell carcinoma, adenoid
		8076/2	Sq. cell carc. in situ with question. stromal invas.
		8076/3	Sq. cell carcinoma, micro-invasive
		8078/3	Squamous cell carcinoma with horn formation

RENAL PELVIS, URETER C659, C669

TRANSITIONAL CELL CARCINOMA, NOS	812	8120/2	Transitional cell carcinoma in situ
		8120/3	Transitional cell carcinoma, NOS
		8121/3	Schneiderian carcinoma
		8122/3	Trans. cell carcinoma, spindle cell
		8123/3	Basaloid carcinoma
		8124/3	Cloacogenic carcinoma
PAPILLARY TRANS. CELL CARCINOMA	813	8130/2	Papillary trans. cell carcinoma, non-invasive
		8130/3	Papillary trans. cell carcinoma
		8131/3	Transitional cell carcinoma, micropapillary
ADENOCARCINOMA, NOS	814	8140/2	Adenocarcinoma in situ
		8140/3	Adenocarcinoma, NOS
		8141/3	Scirrhous adenocarcinoma
		8143/3	Superficial spreading adenocarcinoma
		8147/3	Basal cell adenocarcinoma
TRABECULAR ADENOCARCINOMA	819	8190/3	Trabecular adenocarcinoma
ADENOCA. IN ADENOMA. POLYP	821	8211/3	Tubular adenocarcinoma
SOLID CARCINOMA, NOS	823	8230/2	Duct carcinoma in situ, solid type
		8230/3	Solid carcinoma, NOS
		8231/3	Carcinoma simplex
BRONCHIOLO-ALVEOLAR ADENOCA.	825	8255/3	Adenocarcinoma with mixed subtypes
PAPILLARY ADENOCARCINOMA, NOS	826	8260/3	Papillary adenocarcinoma, NOS
		8261/2	Adenocarcinoma in situ in villous adenoma
CLEAR CELL ADENOCARCINOMA, NOS	831	8310/3	Clear cell adenocarcinoma, NOS
GRANULAR CELL CARCINOMA	832	8320/3	Granular cell carcinoma
		8323/3	Mixed cell adenocarcinoma
CYSTADENOCARCINOMA, NOS	844	8440/3	Cystadenocarcinoma, NOS
MUCINOUS ADENOCARCINOMA	848	8480/3	Mucinous adenocarcinoma
		8481/3	Mucin-producing adenocarcinoma
ADENOSQUAMOUS CARCINOMA	856	8560/3	Adenosquamous carcinoma
		8562/3	Epithelial-myoepithelial carcinoma

RENAL PELVIS, URETER C659, C669

SARCOMA, NOS	880	8800/3	Sarcoma, NOS
		8801/3	Spindle cell sarcoma
		8802/3	Giant cell sarcoma
		8803/3	Small cell sarcoma
		8804/3	Epithelioid sarcoma
		8805/3	Undifferentiated sarcoma
		8806/3	Desmoplastic small round cell tumor
FIBROMATOUS NEOPLASMS	881	8810/3	Fibrosarcoma, NOS
		8811/3	Fibromyxosarcoma
		8813/3	Fascial fibrosarcoma
		8814/3	Infantile fibrosarcoma
		8815/3	Solitary fibrous tumor, malignant
SARCOMA, NOS	882	8825/3	Myofibroblastic sarcoma
FIBROUS HISTIOCYTOMA, MAL.	883	8830/3	Fibrous histiocytoma, malignant
LIPOSARCOMA NEOPLASMS	885	8850/3	Liposarcoma, NOS
		8851/3	Liposarcoma, well differentiated
		8852/3	Myxoid liposarcoma
		8853/3	Round cell liposarcoma
		8854/3	Pleomorphic liposarcoma
		8855/3	Mixed type liposarcoma
		8857/3	Fibroblastic liposarcoma
		8858/3	Dedifferentiated liposarcoma
MYOMATOUS NEOPLASMS	889	8890/3	Leiomyosarcoma, NOS
		8891/3	Epithelioid leiomyosarcoma
		8894/3	Angiomyosarcoma
		8895/3	Myosarcoma
		8896/3	Myxoid leiomyosarcoma
RHABDOMYOSARCOMA, NOS	890	8900/3	Rhabdomyosarcoma, NOS
		8901/3	Pleomorphic rhabdomyosarcoma, adult type
		8902/3	Mixed type rhabdomyosarcoma
EMBRYONAL RHABDOMYOSARCOMA	891	8910/3	Embryonal rhabdomyosarcoma
		8912/3	Spindle cell rhabdomyosarcoma
MULLERIAN MIXED TUMOR	895	8950/3	Mullerian mixed tumor
		8951/3	Mesodermal mixed tumor
		8959/3	Malignant cystic nephroma
CLEAR CELL SARC/NEPHROBLASTOMA	896	8964/3	Clear cell sarcoma of kidney

RENAL PELVIS, URETER C659, C669

CARCINOSARCOMA, NOS	898	8980/3	Carcinosarcoma, NOS
		8981/3	Carcinosarcoma, embryonal type
		8982/3	Malignant myoepithelioma
MESENCHYMOMA, MALIGNANT	899	8990/3	Mesenchymoma, malignant
		8991/3	Embryonal sarcoma
NEUROBLASTOMA, NOS	950	9500/3	Neuroblastoma, NOS
		9501/3	Medulloepithelioma, NOS
		9502/3	Teratoid medulloepithelioma
		9503/3	Neuroepithelioma, NOS
		9504/3	Spongioneuroblastoma
		9505/3	Ganglioglioma, anaplastic
MALIGNANT LYMPHOMA, NOS	959	9590/3	Malignant lymphoma, NOS
		9591/3	Malignant lymphoma, non-Hodgkin
		9596/3	Composite Hodgkin and non-Hodgkin lymphoma
HODGKIN LYMPHOMA	965	9650/3	Hodgkin lymphoma, NOS
		9651/3	Hodgkin lymphoma, lymphocyte-rich
		9652/3	Hodgkin lymphoma, mixed cellularity, NOS
		9653/3	Hodgkin lymphoma, lymphocytic deplet., NOS
		9654/3	Hodgkin lymph., lymphocyt. deplet., diffuse fibrosis
		9655/3	Hodgkin lymphoma, lymphocyt. deplet., reticular
		9659/3	Hodgkin lymph., nodular lymphocyte predom.
HODGKIN LYMPHOMA, NOD. SCLER.	966	9661/3	Hodgkin granuloma [obs]
		9662/3	Hodgkin sarcoma [obs]
		9663/3	Hodgkin lymphoma, nodular sclerosis, NOS
		9664/3	Hodgkin lymphoma, nod. scler., cellular phase
		9665/3	Hodgkin lymphoma, nod. scler., grade 1
		9667/3	Hodgkin lymphoma, nod. scler., grade 2
ML, SMALL B-CELL LYMPHOCYTIC	967	9670/3	ML, small B lymphocytic, NOS
		9671/3	ML, lymphoplasmacytic
		9673/3	Mantle cell lymphoma
		9675/3	ML, mixed sm. and lg. cell, diffuse
ML, LARGE B-CELL, DIFFUSE	968	9680/3	ML, large B-cell, diffuse
		9684/3	ML, large B-cell, diffuse, immunoblastic, NOS
		9687/3	Burkitt lymphoma, NOS
		9688/3	T-cell histiocyte rich large B-cell lymphoma

RENAL PELVIS, URETER C659, C669

FOLLIC. & MARGINAL LYMPH, NOS	969	9690/3	Follicular lymphoma, NOS
		9691/3	Follicular lymphoma, grade 2
		9695/3	Follicular lymphoma, grade 1
		9698/3	Follicular lymphoma, grade 3
		9699/3	Marginal zone B-cell lymphoma, NOS
T-CELL LYMPHOMAS	970	9701/3	Sezary syndrome
		9702/3	Mature T-cell lymphoma, NOS
		9705/3	Angioimmunoblastic T-cell lymphoma
OTHER SPEC. NON-HODGKIN LYMPHOMA	971	9712/3	Intravascular large B-cell lymphoma
		9714/3	Anaplastic large cell lymphoma, T-cell and Null cell type
		9719/3	NK/T-cell lymphoma, nasal and nasal-type
PRECURS. CELL LYMPHOBLASTIC LYMPH.	972	9724/3	SystemicEBV pos. T-cell lymphoproliferative disease of childhood
		9727/3	Precursor cell lymphoblastic lymphoma, NOS
		9728/3	Precursor B-cell lymphoblastic lymphoma
		9729/3	Precursor T-cell lymphoblastic lymphoma
PLASMA CELL TUMORS	973	9731/3	Plasmacytoma, NOS
		9734/3	Plasmacytoma, extramedullary
		9735/3	Plasmablastic lymphoma
		9737/3	ALK positive large B-cell lymphoma
		9738/3	Lrg B-cell lymphoma in HHV8-assoc. multicentric Castleman DZ
MAST CELL TUMORS	974	9740/3	Mast cell sarcoma
		9741/3	Malignant mastocytosis
NEOPLASMS OF HISTIOCYTES AND ACCESSORY LYMPHOID CELLS	975	9750/3	Malignant histiocytosis
		9751/3	Langerhans cell histiocytosis, NOS
		9754/3	Langerhans cell histiocytosis, disseminated
		9755/3	Histiocytic sarcoma
		9756/3	Langerhans cell sarcoma
		9757/3	Interdigitating dendritic cell sarcoma
		9758/3	Follicular dendritic cell sarcoma
		9759/3	Fibroblastic reticular cell tumor
PRECURSOR LYMPHOID NEOPLASMS	981	9811/3	B lymphoblastic leukemia/lymphoma, NOS
		9812/3	Leukemia/lymphoma with t(9;22)(q34;q11.2);BCR-ABL1
		9813/3	Leukemia/lymphoma with t(v;11q23);MLL rearranged
		9814/3	Leukemia/lymphoma with t(12;21)(p13;q22);TEL-AML1(ETV6-RUNX1)
		9815/3	B lymphoblastic leukemia/lymphoma with hyperdiploidy
		9816/3	Leukemia/lymphoma with hypodiploidy (hypodiploid ALL)
		9817/3	B lymphblastic leukemia/lymphoma with t(5;14)(q31;q32);IL3-IGH
		9818/3	Leukemia/lymphoma with t(1;19)(q23;p13.3); E2A PBX1 (TCF3 PBX1)
LYMPHOID LEUKEMIA, NOS	982	9823/3	Chronic lymphocytic leukemia/small lymphocytic lymphoma

RENAL PELVIS, URETER C659, C669

PROLYMPH/PRECURS LEUKEMIA	983	9831/3	T-cell large granular lymphocytic leukemia
		9837/3	T lymphoblastic leukemia/lymphoma
CHRONIC MYELOPROLIFERATIVE DIS.	996	9965/3	Myeloid and lymphoid neoplasms with PDGFRB rearrangement
		9967/3	Myeloid and lymphoid neoplasm with FGFR1 abnormalities
MYELOPLASTIC/MYELOPROLIFERATIVE NEOPLASMS	997	9971/3	Polymorphic PTLD
		9975/3	Myelodysplastic/Myeloproliferative neoplasm, unclassifiable

URINARYBLADDER C670-C679

NEOPLASM	800	8000/3	Neoplasm, malignant
		8001/3	Tumor cells, malignant
		8002/3	Malignant tumor, small cell type
		8003/3	Malignant tumor, giant cell type
		8004/3	Malignant tumor, spindle cell type
		8005/3	Malignant tumor, clear cell type
CARCINOMA, NOS	801	8010/2	Carcinoma in situ, NOS
		8010/3	Carcinoma, NOS
		8011/3	Epithelioma, malignant
		8012/3	Large cell carcinoma, NOS
		8013/3	Large cell neuroendocrine carcinoma
		8014/3	Large cell carcinoma with rhabdoid phenotype
		8015/3	Glassy cell carcinoma
CARCINOMA, UNDIFF., NOS	802	8020/3	Carcinoma, undifferentiated type, NOS
		8021/3	Carcinoma, anaplastic type, NOS
		8022/3	Pleomorphic carcinoma
GIANT & SPINDLE CELL CARCINOMA	803	8030/3	Giant cell and spindle cell carcinoma
		8031/3	Giant cell carcinoma
		8032/3	Spindle cell carcinoma
		8033/3	Pseudosarcomatous carcinoma
		8034/3	Polygonal cell carcinoma
		8035/3	Carcinoma with osteoclast-like giant cells
SMALL CELL CARCINOMA, NOS	804	8041/3	Small cell carcinoma, NOS
		8043/3	Small cell carcinoma, fusiform cell
PAPILLARY CARCINOMA, NOS	805	8050/2	Papillary carcinoma in situ
		8050/3	Papillary carcinoma, NOS
		8051/3	Verrucous carcinoma, NOS
		8052/2	Papillary squamous cell carcinoma, non-invasive
		8052/3	Papillary squamous cell carcinoma
SQUAMOUS CELL CARCINOMA, NOS	807	8070/2	Squamous cell carcinoma in situ, NOS
		8070/3	Squamous cell carcinoma, NOS
		8071/3	Sq. cell carcinoma, keratinizing, NOS
		8072/3	Sq. cell carcinoma, lg. cell, non-ker.
		8073/3	Sq. cell carcinoma, sm. cell, non-ker.
		8074/3	Sq. cell carcinoma, spindle cell
		8075/3	Squamous cell carcinoma, adenoid
		8076/2	Sq. cell carc. in situ with question. stromal invas.
		8076/3	Sq. cell carcinoma, micro-invasive
		8078/3	Squamous cell carcinoma with horn formation

URINARYBLADDER C670-C679

TRANSITIONAL CELL CARCINOMA, NOS	812	8120/2	Transitional cell carcinoma in situ
		8120/3	Transitional cell carcinoma, NOS
		8121/3	Schneiderian carcinoma
		8122/3	Trans. cell carcinoma, spindle cell
		8123/3	Basaloid carcinoma
		8124/3	Cloacogenic carcinoma
PAPILLARY TRANS. CELL CARCINOMA	813	8130/2	Papillary trans. cell carcinoma, non-invasive
		8130/3	Papillary trans. cell carcinoma
		8131/3	Transitional cell carcinoma, micropapillary
ADENOCARCINOMA, NOS	814	8140/2	Adenocarcinoma in situ
		8140/3	Adenocarcinoma, NOS
		8141/3	Scirrhous adenocarcinoma
		8143/3	Superficial spreading adenocarcinoma
		8147/3	Basal cell adenocarcinoma
SOLID CARCINOMA, NOS	823	8230/2	Duct carcinoma in situ, solid type
		8230/3	Solid carcinoma, NOS
		8231/3	Carcinoma simplex
BRONCHIOLO-ALVEOLAR ADENOCA.	825	8255/3	Adenocarcinoma with mixed subtypes
PAPILLARY ADENOCARCINOMA, NOS	826	8260/3	Papillary adenocarcinoma, NOS
		8261/2	Adenocarcinoma in situ in villous adenoma
		8261/3	Adenocarcinoma in villous adenoma
CLEAR CELL ADENOCARCINOMA, NOS	831	8310/3	Clear cell adenocarcinoma, NOS
GRANULAR CELL CARCINOMA	832	8320/3	Granular cell carcinoma
		8323/3	Mixed cell adenocarcinoma
MUCINOUS ADENOCARCINOMA	848	8480/3	Mucinous adenocarcinoma
		8481/3	Mucin-producing adenocarcinoma
SIGNET RING CELL CARCINOMA	849	8490/3	Signet ring cell carcinoma
MEDULLARY CARCINOMA, NOS	851	8510/3	Medullary carcinoma, NOS
ADENOCA. WITH METAPLASIA	857	8576/3	Hepatoid adenocarcinoma
PARAGANGLIOMA	868	8680/3	Paraganglioma, malignant

URINARYBLADDER C670-C679

SARCOMA, NOS	880	8800/3	Sarcoma, NOS
		8801/3	Spindle cell sarcoma
		8802/3	Giant cell sarcoma
		8803/3	Small cell sarcoma
		8804/3	Epithelioid sarcoma
		8805/3	Undifferentiated sarcoma
		8806/3	Desmoplastic small round cell tumor
FIBROMATOUS NEOPLASMS	881	8810/3	Fibrosarcoma, NOS
		8811/3	Fibromyxosarcoma
		8813/3	Fascial fibrosarcoma
		8814/3	Infantile fibrosarcoma
		8815/3	Solitary fibrous tumor, malignant
SARCOMA, NOS	882	8825/3	Myofibroblastic sarcoma
FIBROUS HISTIOCYTOMA, MAL.	883	8830/3	Fibrous histiocytoma, malignant
MYOMATOUS NEOPLASMS	889	8890/3	Leiomyosarcoma, NOS
		8891/3	Epithelioid leiomyosarcoma
		8894/3	Angiomyosarcoma
		8895/3	Myosarcoma
		8896/3	Myxoid leiomyosarcoma
RHABDOMYOSARCOMA, NOS	890	8900/3	Rhabdomyosarcoma, NOS
		8901/3	Pleomorphic rhabdomyosarcoma, adult type
		8902/3	Mixed type rhabdomyosarcoma
EMBRYONAL RHABDOMYOSARCOMA	891	8910/3	Embryonal rhabdomyosarcoma
		8912/3	Spindle cell rhabdomyosarcoma
ALVEOLAR RHABDOMYOSARCOMA	892	8920/3	Alveolar rhabdomyosarcoma
		8921/3	Rhabdomyosarcoma with ganglionic differentiation
MULLERIAN MIXED TUMOR	895	8950/3	Mullerian mixed tumor
		8951/3	Mesodermal mixed tumor
CARCINOSARCOMA, NOS	898	8980/3	Carcinosarcoma, NOS
		8981/3	Carcinosarcoma, embryonal type
		8982/3	Malignant myoepithelioma
MESENCHYMOMA, MALIGNANT	899	8990/3	Mesenchymoma, malignant
		8991/3	Embryonal sarcoma

URINARYBLADDER C670-C679

MALIGNANT LYMPHOMA, NOS	959	9590/3	Malignant lymphoma, NOS
		9591/3	Malignant lymphoma, non-Hodgkin
		9596/3	Composite Hodgkin and non-Hodgkin lymphoma
HODGKIN LYMPHOMA	965	9650/3	Hodgkin lymphoma, NOS
		9651/3	Hodgkin lymphoma, lymphocyte-rich
		9652/3	Hodgkin lymphoma, mixed cellularity, NOS
		9653/3	Hodgkin lymphoma, lymphocytic deplet., NOS
		9654/3	Hodgkin lymph., lymphocyt. deplet., diffuse fibrosis
		9655/3	Hodgkin lymphoma, lymphocyt. deplet., reticular
		9659/3	Hodgkin lymph., nodular lymphocyte predom.
HODGKIN LYMPHOMA, NOD. SCLER.	966	9661/3	Hodgkin granuloma [obs]
		9662/3	Hodgkin sarcoma [obs]
		9663/3	Hodgkin lymphoma, nodular sclerosis, NOS
		9664/3	Hodgkin lymphoma, nod. scler., cellular phase
		9665/3	Hodgkin lymphoma, nod. scler., grade 1
		9667/3	Hodgkin lymphoma, nod. scler., grade 2
ML, SMALL B-CELL LYMPHOCYTIC	967	9670/3	ML, small B lymphocytic, NOS
		9671/3	ML, lymphoplasmacytic
		9673/3	Mantle cell lymphoma
		9675/3	ML, mixed sm. and lg. cell, diffuse
ML, LARGE B-CELL, DIFFUSE	968	9680/3	ML, large B-cell, diffuse
		9684/3	ML, large B-cell, diffuse, immunoblastic, NOS
		9687/3	Burkitt lymphoma, NOS
		9688/3	T-cell histiocyte rich large B-cell lymphoma
FOLLIC. & MARGINAL LYMPH, NOS	969	9690/3	Follicular lymphoma, NOS
		9691/3	Follicular lymphoma, grade 2
		9695/3	Follicular lymphoma, grade 1
		9698/3	Follicular lymphoma, grade 3
		9699/3	Marginal zone B-cell lymphoma, NOS
T-CELL LYMPHOMAS	970	9701/3	Sezary syndrome
		9702/3	Mature T-cell lymphoma, NOS
		9705/3	Angioimmunoblastic T-cell lymphoma
OTHER SPEC. NON-HODGKIN LYMPHOMA	971	9712/3	Intravascular large B-cell lymphoma
		9714/3	Anaplastic large cell lymphoma, T-cell and Null cell type
		9719/3	NK/T-cell lymphoma, nasal and nasal-type

URINARYBLADDER C670-C679

PRECURS. CELL LYMPHOBLASTIC LYMPH.	972	9724/3	SystemicEBV pos. T-cell lymphoproliferative disease of childhood
		9727/3	Precursor cell lymphoblastic lymphoma, NOS
		9728/3	Precursor B-cell lymphoblastic lymphoma
		9729/3	Precursor T-cell lymphoblastic lymphoma
PLASMA CELL TUMORS	973	9731/3	Plasmacytoma, NOS
		9734/3	Plasmacytoma, extramedullary
		9735/3	Plasmablastic lymphoma
		9737/3	ALK positive large B-cell lymphoma
		9738/3	Lrg B-cell lymphoma in HHV8-assoc. multicentric Castleman DZ
MAST CELL TUMORS	974	9740/3	Mast cell sarcoma
		9741/3	Malignant mastocytosis
NEOPLASMS OF HISTIOCYTES AND ACCESSORY LYMPHOID CELLS	975	9750/3	Malignant histiocytosis
		9751/3	Langerhans cell histiocytosis, NOS
		9754/3	Langerhans cell histiocytosis, disseminated
		9755/3	Histiocytic sarcoma
		9756/3	Langerhans cell sarcoma
		9757/3	Interdigitating dendritic cell sarcoma
		9758/3	Follicular dendritic cell sarcoma
		9759/3	Fibroblastic reticular cell tumor
PRECURSOR LYMPHOID NEOPLASMS	981	9811/3	B lymphoblastic leukemia/lymphoma, NOS
		9812/3	Leukemia/lymphoma with t(9;22)(q34;q11.2);BCR-ABL1
		9813/3	Leukemia/lymphoma with t(v;11q23);MLL rearranged
		9814/3	Leukemia/lymphoma with t(12;21)(p13;q22);TEL-AML1(ETV6-RUNX1)
		9815/3	B lymphoblastic leukemia/lymphoma with hyperdiploidy
		9816/3	Leukemia/lymphoma with hypodiploidy (hypodiploid ALL)
		9817/3	B lymphblastic leukemia/lymphoma with t(5;14)(q31;q32);IL3-IGH
		9818/3	Leukemia/lymphoma with t(1;19)(q23;p13.3); E2A PBX1 (TCF3 PBX1)
LYMPHOID LEUKEMIA, NOS	982	9823/3	Chronic lymphocytic leukemia/small lymphocytic lymphoma
PROLYMPH/PRECURS LEUKEMIA	983	9831/3	T-cell large granular lymphocytic leukemia
		9837/3	T lymphoblastic leukemia/lymphoma
CHRONIC MYELOPROLIFERATIVE DIS.	996	9965/3	Myeloid and lymphoid neoplasms with PDGFRB rearrangement
		9967/3	Myeloid and lymphoid neoplasm with FGFR1 abnormalities
MYELOPLASTIC/MYELOPROLIFERATIVE NEOPLASMS	997	9971/3	Polymorphic PTLD
		9975/3	Myelodysplastic/Myeloproliferative neoplasm, unclassifiable

OTHER URINARYORGANS C680-C681,C688-C689

NEOPLASM	800	8000/3	Neoplasm, malignant
		8001/3	Tumor cells, malignant
		8002/3	Malignant tumor, small cell type
		8003/3	Malignant tumor, giant cell type
		8004/3	Malignant tumor, spindle cell type
		8005/3	Malignant tumor, clear cell type
CARCINOMA, NOS	801	8010/2	Carcinoma in situ, NOS
		8010/3	Carcinoma, NOS
		8011/3	Epithelioma, malignant
		8012/3	Large cell carcinoma, NOS
		8013/3	Large cell neuroendocrine carcinoma
		8014/3	Large cell carcinoma with rhabdoid phenotype
		8015/3	Glassy cell carcinoma
CARCINOMA, UNDIFF., NOS	802	8020/3	Carcinoma, undifferentiated type, NOS
		8021/3	Carcinoma, anaplastic type, NOS
		8022/3	Pleomorphic carcinoma
GIANT & SPINDLE CELL CARCINOMA	803	8030/3	Giant cell and spindle cell carcinoma
		8031/3	Giant cell carcinoma
		8032/3	Spindle cell carcinoma
		8033/3	Pseudosarcomatous carcinoma
		8034/3	Polygonal cell carcinoma
		8035/3	Carcinoma with osteoclast-like giant cells
SMALL CELL CARCINOMA, NOS	804	8041/3	Small cell carcinoma, NOS
		8043/3	Small cell carcinoma, fusiform cell
PAPILLARY CARCINOMA, NOS	805	8050/2	Papillary carcinoma in situ
		8050/3	Papillary carcinoma, NOS
		8051/3	Verrucous carcinoma, NOS
		8052/2	Papillary squamous cell carcinoma, non-invasive
		8052/3	Papillary squamous cell carcinoma
SQUAMOUS CELL CARCINOMA, NOS	807	8070/2	Squamous cell carcinoma in situ, NOS
		8070/3	Squamous cell carcinoma, NOS
		8071/3	Sq. cell carcinoma, keratinizing, NOS
		8072/3	Sq. cell carcinoma, lg. cell, non-ker.
		8073/3	Sq. cell carcinoma, sm. cell, non-ker.
		8074/3	Sq. cell carcinoma, spindle cell
		8075/3	Squamous cell carcinoma, adenoid
		8076/2	Sq. cell carc. in situ with question. stromal invas.
		8076/3	Sq. cell carcinoma, micro-invasive
		8078/3	Squamous cell carcinoma with horn formation

OTHER URINARYORGANS C680-C681,C688-C689

TRANSITIONAL CELL CARCINOMA, NOS	812	8120/2	Transitional cell carcinoma in situ
		8120/3	Transitional cell carcinoma, NOS
		8121/3	Schneiderian carcinoma
		8122/3	Trans. cell carcinoma, spindle cell
		8123/3	Basaloid carcinoma
		8124/3	Cloacogenic carcinoma
PAPILLARY TRANS. CELL CARCINOMA	813	8130/2	Papillary trans. cell carcinoma, non-invasive
		8130/3	Papillary trans. cell carcinoma
		8131/3	Transitional cell carcinoma, micropapillary
ADENOCARCINOMA, NOS	814	8140/2	Adenocarcinoma in situ
		8140/3	Adenocarcinoma, NOS
		8141/3	Scirrhous adenocarcinoma
		8143/3	Superficial spreading adenocarcinoma
		8147/3	Basal cell adenocarcinoma
TRABECULAR ADENOCARCINOMA	819	8190/3	Trabecular adenocarcinoma
SOLID CARCINOMA, NOS	823	8230/2	Duct carcinoma in situ, solid type
		8230/3	Solid carcinoma, NOS
		8231/3	Carcinoma simplex
BRONCHIOLO-ALVEOLAR ADENOCA.	825	8255/3	Adenocarcinoma with mixed subtypes
PAPILLARY ADENOCARCINOMA, NOS	826	8260/3	Papillary adenocarcinoma, NOS
		8261/2	Adenocarcinoma in situ in villous adenoma
		8261/3	Adenocarcinoma in villous adenoma
		8262/3	Villous adenocarcinoma
		8263/2	Adenocarcinoma in situ in tubulovillous adenoma
		8263/3	Adenocarcinoma in tubulovillous adenoma
CLEAR CELL ADENOCARCINOMA, NOS	831	8310/3	Clear cell adenocarcinoma, NOS
GRANULAR CELL CARCINOMA	832	8320/3	Granular cell carcinoma
		8323/3	Mixed cell adenocarcinoma
ENDOMETRIOID ADENOCARCINOMA	838	8380/3	Endometrioid carcinoma
CYSTADENOCARCINOMA, NOS	844	8440/3	Cystadenocarcinoma, NOS
MUCINOUS ADENOCARCINOMA	848	8480/3	Mucinous adenocarcinoma
		8481/3	Mucin-producing adenocarcinoma

OTHER URINARYORGANS C680-C681,C688-C689

ADENOSQUAMOUS CARCINOMA	856	8560/3	Adenosquamous carcinoma
		8562/3	Epithelial-myoepithelial carcinoma
NEVI & MELANOMAS	872	8720/2	Melanoma in situ
		8720/3	Malignant melanoma, NOS
		8721/3	Nodular melanoma
		8722/3	Balloon cell melanoma
		8723/3	Malignant melanoma, regressing
AMELANOTIC MELANOMA	873	8730/3	Amelanotic melanoma
MAL. MEL. IN JUNCT. NEVUS	874	8743/3	Superficial spreading melanoma
		8745/3	Desmoplastic melanoma, malignant
		8746/3	Mucosal lentiginous melanoma
EPITHELIOID CELL MELANOMA	877	8770/3	Mixed epithel. & spindle cell melanoma
		8771/3	Epithelioid cell melanoma
		8772/3	Spindle cell melanoma, NOS
SARCOMA, NOS	880	8800/3	Sarcoma, NOS
		8801/3	Spindle cell sarcoma
		8802/3	Giant cell sarcoma
		8803/3	Small cell sarcoma
		8804/3	Epithelioid sarcoma
		8805/3	Undifferentiated sarcoma
		8806/3	Desmoplastic small round cell tumor
FIBROMATOUS NEOPLASMS	881	8810/3	Fibrosarcoma, NOS
		8811/3	Fibromyxosarcoma
		8813/3	Fascial fibrosarcoma
		8814/3	Infantile fibrosarcoma
		8815/3	Solitary fibrous tumor, malignant
SARCOMA, NOS	882	8825/3	Myofibroblastic sarcoma
LIPOSARCOMA NEOPLASMS	885	8850/3	Liposarcoma, NOS
		8851/3	Liposarcoma, well differentiated
		8852/3	Myxoid liposarcoma
		8853/3	Round cell liposarcoma
		8854/3	Pleomorphic liposarcoma
		8855/3	Mixed type liposarcoma
		8857/3	Fibroblastic liposarcoma
		8858/3	Dedifferentiated liposarcoma

OTHER URINARYORGANS C680-C681,C688-C689

MYOMATOUS NEOPLASMS	889	8890/3	Leiomyosarcoma, NOS
		8891/3	Epithelioid leiomyosarcoma
		8894/3	Angiomyosarcoma
		8895/3	Myosarcoma
		8896/3	Myxoid leiomyosarcoma
RHABDOMYOSARCOMA, NOS	890	8900/3	Rhabdomyosarcoma, NOS
		8901/3	Pleomorphic rhabdomyosarcoma, adult type
		8902/3	Mixed type rhabdomyosarcoma
EMBRYONAL RHABDOMYOSARCOMA	891	8910/3	Embryonal rhabdomyosarcoma
		8912/3	Spindle cell rhabdomyosarcoma
MULLERIAN MIXED TUMOR	895	8950/3	Mullerian mixed tumor
		8951/3	Mesodermal mixed tumor
CLEAR CELL SARC/NEPHROBLASTOMA	896	8964/3	Clear cell sarcoma of kidney
CARCINOSARCOMA, NOS	898	8980/3	Carcinosarcoma, NOS
		8981/3	Carcinosarcoma, embryonal type
		8982/3	Malignant myoepithelioma
MESENCHYMOMA, MALIGNANT	899	8990/3	Mesenchymoma, malignant
		8991/3	Embryonal sarcoma
EMBRYONAL CARCINOMA, NOS	907	9070/3	Embryonal carcinoma, NOS
		9071/3	Yolk sac tumor
		9072/3	Polyembryoma
TERATOMA	908	9080/3	Teratoma, malignant, NOS
		9081/3	Teratocarcinoma
		9082/3	Malignant teratoma, undiff.
		9083/3	Malignant teratoma, intermediate
		9084/3	Teratoma with malig. transformation
		9085/3	Mixed germ cell tumor
MESONEPHROMA, MALIGNANT	911	9110/3	Mesonephroma, malignant
NEUROBLASTOMA, NOS	950	9500/3	Neuroblastoma, NOS
		9501/3	Medulloepithelioma, NOS
		9502/3	Teratoid medulloepithelioma
		9503/3	Neuroepithelioma, NOS
		9504/3	Spongioneuroblastoma
		9505/3	Ganglioglioma, anaplastic

OTHER URINARYORGANS C680-C681,C688-C689

MALIGNANT LYMPHOMA, NOS	959	9590/3	Malignant lymphoma, NOS
		9591/3	Malignant lymphoma, non-Hodgkin
		9596/3	Composite Hodgkin and non-Hodgkin lymphoma
HODGKIN LYMPHOMA	965	9650/3	Hodgkin lymphoma, NOS
		9651/3	Hodgkin lymphoma, lymphocyte-rich
		9652/3	Hodgkin lymphoma, mixed cellularity, NOS
		9653/3	Hodgkin lymphoma, lymphocytic deplet., NOS
		9654/3	Hodgkin lymph., lymphocyt. deplet., diffuse fibrosis
		9655/3	Hodgkin lymphoma, lymphocyt. deplet., reticular
		9659/3	Hodgkin lymph., nodular lymphocyte predom.
HODGKIN LYMPHOMA, NOD. SCLER.	966	9661/3	Hodgkin granuloma [obs]
		9662/3	Hodgkin sarcoma [obs]
		9663/3	Hodgkin lymphoma, nodular sclerosis, NOS
		9664/3	Hodgkin lymphoma, nod. scler., cellular phase
		9665/3	Hodgkin lymphoma, nod. scler., grade 1
		9667/3	Hodgkin lymphoma, nod. scler., grade 2
ML, SMALL B-CELL LYMPHOCYTIC	967	9670/3	ML, small B lymphocytic, NOS
		9671/3	ML, lymphoplasmacytic
		9673/3	Mantle cell lymphoma
		9675/3	ML, mixed sm. and lg. cell, diffuse
ML, LARGE B-CELL, DIFFUSE	968	9680/3	ML, large B-cell, diffuse
		9684/3	ML, large B-cell, diffuse, immunoblastic, NOS
		9687/3	Burkitt lymphoma, NOS
		9688/3	T-cell histiocyte rich large B-cell lymphoma
FOLLIC. & MARGINAL LYMPH, NOS	969	9690/3	Follicular lymphoma, NOS
		9691/3	Follicular lymphoma, grade 2
		9695/3	Follicular lymphoma, grade 1
		9698/3	Follicular lymphoma, grade 3
		9699/3	Marginal zone B-cell lymphoma, NOS
T-CELL LYMPHOMAS	970	9701/3	Sezary syndrome
		9702/3	Mature T-cell lymphoma, NOS
		9705/3	Angioimmunoblastic T-cell lymphoma
OTHER SPEC. NON-HODGKIN LYMPHOMA	971	9712/3	Intravascular large B-cell lymphoma
		9714/3	Anaplastic large cell lymphoma, T-cell and Null cell type
		9719/3	NK/T-cell lymphoma, nasal and nasal-type

OTHER URINARYORGANS C680-C681,C688-C689

PRECURS. CELL LYMPHOBLASTIC LYMPH.	972	9724/3	SystemicEBV pos. T-cell lymphoproliferative disease of childhood
		9727/3	Precursor cell lymphoblastic lymphoma, NOS
		9728/3	Precursor B-cell lymphoblastic lymphoma
		9729/3	Precursor T-cell lymphoblastic lymphoma
PLASMA CELL TUMORS	973	9731/3	Plasmacytoma, NOS
		9734/3	Plasmacytoma, extramedullary
		9735/3	Plasmablastic lymphoma
		9737/3	ALK positive large B-cell lymphoma
		9738/3	Lrg B-cell lymphoma in HHV8-assoc. multicentric Castleman DZ
MAST CELL TUMORS	974	9740/3	Mast cell sarcoma
		9741/3	Malignant mastocytosis
NEOPLASMS OF HISTIOCYTES AND ACCESSORY LYMPHOID CELLS	975	9750/3	Malignant histiocytosis
		9751/3	Langerhans cell histiocytosis, NOS
		9754/3	Langerhans cell histiocytosis, disseminated
		9755/3	Histiocytic sarcoma
		9756/3	Langerhans cell sarcoma
		9757/3	Interdigitating dendritic cell sarcoma
		9758/3	Follicular dendritic cell sarcoma
		9759/3	Fibroblastic reticular cell tumor
PRECURSOR LYMPHOID NEOPLASMS	981	9811/3	B lymphoblastic leukemia/lymphoma, NOS
		9812/3	Leukemia/lymphoma with t(9;22)(q34;q11.2);BCR-ABL1
		9813/3	Leukemia/lymphoma with t(v;11q23);MLL rearranged
		9814/3	Leukemia/lymphoma with t(12;21)(p13;q22);TEL-AML1(ETV6-RUNX1)
		9815/3	B lymphoblastic leukemia/lymphoma with hyperdiploidy
		9816/3	Leukemia/lymphoma with hypodiploidy (hypodiploid ALL)
		9817/3	B lymphblastic leukemia/lymphoma with t(5;14)(q31;q32);IL3-IGH
		9818/3	Leukemia/lymphoma with t(1;19)(q23;p13.3); E2A PBX1 (TCF3 PBX1)
LYMPHOID LEUKEMIA, NOS	982	9823/3	Chronic lymphocytic leukemia/small lymphocytic lymphoma
PROLYMPH/PRECURS LEUKEMIA	983	9831/3	T-cell large granular lymphocytic leukemia
		9837/3	T lymphoblastic leukemia/lymphoma
CHRONIC MYELOPROLIFERATIVE DIS.	996	9965/3	Myeloid and lymphoid neoplasms with PDGFRB rearrangement
		9967/3	Myeloid and lymphoid neoplasm with FGFR1 abnormalities
MYELOPLASTIC/MYELOPROLIFERATIVE NEOPLASMS	997	9971/3	Polymorphic PTLD
		9975/3	Myelodysplastic/Myeloproliferative neoplasm, unclassifiable

ORBIT & LACRIMALGLAND, (EXCL. RETINA, EYE, NOS) C690-C691, C693, C695-C698

NEOPLASM	800	8000/3	Neoplasm, malignant
		8001/3	Tumor cells, malignant
		8002/3	Malignant tumor, small cell type
		8003/3	Malignant tumor, giant cell type
		8004/3	Malignant tumor, spindle cell type
		8005/3	Malignant tumor, clear cell type
CARCINOMA, NOS	801	8010/2	Carcinoma in situ, NOS
		8010/3	Carcinoma, NOS
		8011/3	Epithelioma, malignant
		8012/3	Large cell carcinoma, NOS
		8013/3	Large cell neuroendocrine carcinoma
		8014/3	Large cell carcinoma with rhabdoid phenotype
		8015/3	Glassy cell carcinoma
CARCINOMA, UNDIFF., NOS	802	8020/3	Carcinoma, undifferentiated type, NOS
		8021/3	Carcinoma, anaplastic type, NOS
		8022/3	Pleomorphic carcinoma
PAPILLARY CARCINOMA, NOS	805	8050/2	Papillary carcinoma in situ
		8050/3	Papillary carcinoma, NOS
		8051/3	Verrucous carcinoma, NOS
		8052/2	Papillary squamous cell carcinoma, non-invasive
		8052/3	Papillary squamous cell carcinoma
SQUAMOUS CELL CARCINOMA, NOS	807	8070/2	Squamous cell carcinoma in situ, NOS
		8070/3	Squamous cell carcinoma, NOS
		8071/3	Sq. cell carcinoma, keratinizing, NOS
		8072/3	Sq. cell carcinoma, lg. cell, non-ker.
		8073/3	Sq. cell carcinoma, sm. cell, non-ker.
		8074/3	Sq. cell carcinoma, spindle cell
		8075/3	Squamous cell carcinoma, adenoid
		8076/2	Sq. cell carc. in situ with question. stromal invas.
		8076/3	Sq. cell carcinoma, micro-invasive
		8078/3	Squamous cell carcinoma with horn formation
LYMPHOEPITHELIAL CARCINOMA	808	8081/2	Bowen disease
		8082/3	Lymphoepithelial carcinoma
		8083/3	Basaloid squamous cell carcinoma
		8084/3	Squamous cell carcinoma, clear cell type
TRANSITIONAL CELL CARCINOMA, NOS	812	8120/2	Transitional cell carcinoma in situ
		8120/3	Transitional cell carcinoma, NOS
		8121/3	Schneiderian carcinoma
		8122/3	Trans. cell carcinoma, spindle cell
		8123/3	Basaloid carcinoma
		8124/3	Cloacogenic carcinoma

ORBIT & LACRIMALGLAND, (EXCL. RETINA, EYE, NOS) C690-C691, C693, C695-C698

PAPILLARY TRANS. CELL CARCINOMA	813	8130/2	Papillary trans. cell carcinoma, non-invasive
		8130/3	Papillary trans. cell carcinoma
		8131/3	Transitional cell carcinoma, micropapillary
ADENOCARCINOMA, NOS	814	8140/2	Adenocarcinoma in situ
		8140/3	Adenocarcinoma, NOS
		8141/3	Scirrhous adenocarcinoma
		8143/3	Superficial spreading adenocarcinoma
		8147/3	Basal cell adenocarcinoma
ADENOID CYSTIC & CRIBRIFORM CA.	820	8200/3	Adenoid cystic carcinoma
		8201/2	Cribriform carcinoma in situ
		8201/3	Cribriform carcinoma
BRONCHIOLO-ALVEOLAR ADENOCA.	825	8255/3	Adenocarcinoma with mixed subtypes
OXYPHILIC ADENOCARCINOMA	829	8290/3	Oxyphilic adenocarcinoma
MUCOEPIDERMOID CARCINOMA	843	8430/3	Mucoepidermoid carcinoma
NEVI & MELANOMAS	872	8720/2	Melanoma in situ
		8720/3	Malignant melanoma, NOS
		8721/3	Nodular melanoma
		8722/3	Balloon cell melanoma
		8723/3	Malignant melanoma, regressing
AMELANOTIC MELANOMA	873	8730/3	Amelanotic melanoma
MAL. MEL. IN JUNCT. NEVUS	874	8743/3	Superficial spreading melanoma
		8745/3	Desmoplastic melanoma, malignant
		8746/3	Mucosal lentiginous melanoma
EPITHELIOID CELL MELANOMA	877	8770/3	Mixed epithel. & spindle cell melanoma
		8771/3	Epithelioid cell melanoma
		8772/3	Spindle cell melanoma, NOS
		8773/3	Spindle cell melanoma, type A
		8774/3	Spindle cell melanoma, type B
FIBROMATOUS NEOPLASMS	881	8810/3	Fibrosarcoma, NOS
		8811/3	Fibromyxosarcoma
		8813/3	Fascial fibrosarcoma
		8814/3	Infantile fibrosarcoma
		8815/3	Solitary fibrous tumor, malignant
SARCOMA, NOS	882	8825/3	Myofibroblastic sarcoma

ORBIT & LACRIMALGLAND, (EXCL. RETINA, EYE, NOS) C690-C691, C693, C695-C698

MYOMATOUS NEOPLASMS	889	8890/3	Leiomyosarcoma, NOS
		8891/3	Epithelioid leiomyosarcoma
		8894/3	Angiomyosarcoma
		8895/3	Myosarcoma
		8896/3	Myxoid leiomyosarcoma
RHABDOMYOSARCOMA, NOS	890	8900/3	Rhabdomyosarcoma, NOS
		8901/3	Pleomorphic rhabdomyosarcoma, adult type
		8902/3	Mixed type rhabdomyosarcoma
EMBRYONAL RHABDOMYOSARCOMA	891	8910/3	Embryonal rhabdomyosarcoma
		8912/3	Spindle cell rhabdomyosarcoma
ALVEOLAR RHABDOMYOSARCOMA	892	8920/3	Alveolar rhabdomyosarcoma
		8921/3	Rhabdomyosarcoma with ganglionic differentiation
MIXED TUMOR, MALIGNANT, NOS	894	8940/3	Mixed tumor, malignant, NOS
		8941/3	Carcinoma in pleomorphic adenoma
MESENCHYMOMA, MALIGNANT	899	8990/3	Mesenchymoma, malignant
		8991/3	Embryonal sarcoma
HEMANGIOPERICYTOMA	915	9150/3	Hemangiopericytoma, malignant
MALIGNANT LYMPHOMA, NOS	959	9590/3	Malignant lymphoma, NOS
		9591/3	Malignant lymphoma, non-Hodgkin
		9596/3	Composite Hodgkin and non-Hodgkin lymphoma
HODGKIN LYMPHOMA	965	9650/3	Hodgkin lymphoma, NOS
		9651/3	Hodgkin lymphoma, lymphocyte-rich
		9652/3	Hodgkin lymphoma, mixed cellularity, NOS
		9653/3	Hodgkin lymphoma, lymphocytic deplet., NOS
		9654/3	Hodgkin lymph., lymphocyt. deplet., diffuse fibrosis
		9655/3	Hodgkin lymphoma, lymphocyt. deplet., reticular
		9659/3	Hodgkin lymph., nodular lymphocyte predom.
HODGKIN LYMPHOMA, NOD. SCLER.	966	9661/3	Hodgkin granuloma [obs]
		9662/3	Hodgkin sarcoma [obs]
		9663/3	Hodgkin lymphoma, nodular sclerosis, NOS
		9664/3	Hodgkin lymphoma, nod. scler., cellular phase
		9665/3	Hodgkin lymphoma, nod. scler., grade 1
		9667/3	Hodgkin lymphoma, nod. scler., grade 2

ORBIT & LACRIMALGLAND, (EXCL. RETINA, EYE, NOS) C690-C691, C693, C695-C698

ML, SMALL B-CELL LYMPHOCYTIC	967	9670/3	ML, small B lymphocytic, NOS
		9671/3	ML, lymphoplasmacytic
		9673/3	Mantle cell lymphoma
		9675/3	ML, mixed sm. and lg. cell, diffuse
ML, LARGE B-CELL, DIFFUSE	968	9680/3	ML, large B-cell, diffuse
		9684/3	ML, large B-cell, diffuse, immunoblastic, NOS
		9687/3	Burkitt lymphoma, NOS
		9688/3	T-cell histiocyte rich large B-cell lymphoma
FOLLIC. & MARGINAL LYMPH, NOS	969	9690/3	Follicular lymphoma, NOS
		9691/3	Follicular lymphoma, grade 2
		9695/3	Follicular lymphoma, grade 1
		9698/3	Follicular lymphoma, grade 3
		9699/3	Marginal zone B-cell lymphoma, NOS
T-CELL LYMPHOMAS	970	9701/3	Sezary syndrome
		9702/3	Mature T-cell lymphoma, NOS
		9705/3	Angioimmunoblastic T-cell lymphoma
OTHER SPEC. NON-HODGKIN LYMPHOMA	971	9712/3	Intravascular large B-cell lymphoma
		9714/3	Anaplastic large cell lymphoma, T-cell and Null cell type
		9719/3	NK/T-cell lymphoma, nasal and nasal-type
PRECURS. CELL LYMPHOBLASTIC LYMPH.	972	9724/3	SystemicEBV pos. T-cell lymphoproliferative disease of childhood
		9727/3	Precursor cell lymphoblastic lymphoma, NOS
		9728/3	Precursor B-cell lymphoblastic lymphoma
		9729/3	Precursor T-cell lymphoblastic lymphoma
PLASMA CELL TUMORS	973	9731/3	Plasmacytoma, NOS
		9734/3	Plasmacytoma, extramedullary
		9735/3	Plasmablastic lymphoma
		9737/3	ALK positive large B-cell lymphoma
		9738/3	Lrg B-cell lymphoma in HHV8-assoc. multicentric Castleman DZ
MAST CELL TUMORS	974	9740/3	Mast cell sarcoma
		9741/3	Malignant mastocytosis
NEOPLASMS OF HISTIOCYTES AND ACCESSORY LYMPHOID CELLS	975	9750/3	Malignant histiocytosis
		9751/3	Langerhans cell histiocytosis, NOS
		9754/3	Langerhans cell histiocytosis, disseminated
		9755/3	Histiocytic sarcoma
		9756/3	Langerhans cell sarcoma
		9757/3	Interdigitating dendritic cell sarcoma
		9758/3	Follicular dendritic cell sarcoma
		9759/3	Fibroblastic reticular cell tumor

ORBIT & LACRIMALGLAND, (EXCL. RETINA, EYE, NOS) C690-C691, C693, C695-C698

PRECURSOR LYMPHOID NEOPLASMS	981	9811/3	B lymphoblastic leukemia/lymphoma, NOS
		9812/3	Leukemia/lymphoma with t(9;22)(q34;q11.2);BCR-ABL1
		9813/3	Leukemia/lymphoma with t(v;11q23);MLL rearranged
		9814/3	Leukemia/lymphoma with t(12;21)(p13;q22);TEL-AML1(ETV6-RUNX1)
		9815/3	B lymphoblastic leukemia/lymphoma with hyperdiploidy
		9816/3	Leukemia/lymphoma with hypodiploidy (hypodiploid ALL)
		9817/3	B lymphblastic leukemia/lymphoma with t(5;14)(q31;q32);IL3-IGH
		9818/3	Leukemia/lymphoma with t(1;19)(q23;p13.3); E2A PBX1 (TCF3 PBX1)
LYMPHOID LEUKEMIA, NOS	982	9823/3	Chronic lymphocytic leukemia/small lymphocytic lymphoma
PROLYMPH/PRECURS LEUKEMIA	983	9831/3	T-cell large granular lymphocytic leukemia
		9837/3	T lymphoblastic leukemia/lymphoma
CHRONIC MYELOPROLIFERATIVE DIS.	996	9965/3	Myeloid and lymphoid neoplasms with PDGFRB rearrangement
		9967/3	Myeloid and lymphoid neoplasm with FGFR1 abnormalities
MYELOPLASTIC/MYELOPROLIFERATIVE NEOPLASMS	997	9971/3	Polymorphic PTLD
		9975/3	Myelodysplastic/Myeloproliferative neoplasm, unclassifiable

RETINA C692

NEOPLASM	800	8000/3	Neoplasm, malignant
		8001/3	Tumor cells, malignant
		8002/3	Malignant tumor, small cell type
		8003/3	Malignant tumor, giant cell type
		8004/3	Malignant tumor, spindle cell type
		8005/3	Malignant tumor, clear cell type
CARCINOMA, NOS	801	8010/2	Carcinoma in situ, NOS
		8010/3	Carcinoma, NOS
		8011/3	Epithelioma, malignant
		8012/3	Large cell carcinoma, NOS
		8013/3	Large cell neuroendocrine carcinoma
		8014/3	Large cell carcinoma with rhabdoid phenotype
		8015/3	Glassy cell carcinoma
CARCINOMA, UNDIFF., NOS	802	8020/3	Carcinoma, undifferentiated type, NOS
		8021/3	Carcinoma, anaplastic type, NOS
		8022/3	Pleomorphic carcinoma
PAPILLARY CARCINOMA, NOS	805	8050/2	Papillary carcinoma in situ
		8050/3	Papillary carcinoma, NOS
		8051/3	Verrucous carcinoma, NOS
		8052/2	Papillary squamous cell carcinoma, non-invasive
		8052/3	Papillary squamous cell carcinoma
SQUAMOUS CELL CARCINOMA, NOS	807	8070/2	Squamous cell carcinoma in situ, NOS
		8070/3	Squamous cell carcinoma, NOS
		8071/3	Sq. cell carcinoma, keratinizing, NOS
		8072/3	Sq. cell carcinoma, lg. cell, non-ker.
		8073/3	Sq. cell carcinoma, sm. cell, non-ker.
		8074/3	Sq. cell carcinoma, spindle cell
		8075/3	Squamous cell carcinoma, adenoid
		8076/2	Sq. cell carc. in situ with question. stromal invas.
		8076/3	Sq. cell carcinoma, micro-invasive
		8078/3	Squamous cell carcinoma with horn formation
LYMPHOEPITHELIAL CARCINOMA	808	8081/2	Bowen disease
		8082/3	Lymphoepithelial carcinoma
		8083/3	Basaloid squamous cell carcinoma
		8084/3	Squamous cell carcinoma, clear cell type
TRANSITIONAL CELL CARCINOMA, NOS	812	8120/2	Transitional cell carcinoma in situ
		8120/3	Transitional cell carcinoma, NOS
		8121/3	Schneiderian carcinoma
		8122/3	Trans. cell carcinoma, spindle cell
		8123/3	Basaloid carcinoma
		8124/3	Cloacogenic carcinoma

RETINA C692

PAPILLARY TRANS. CELL CARCINOMA	813	8130/2	Papillary trans. cell carcinoma, non-invasive
		8130/3	Papillary trans. cell carcinoma
		8131/3	Transitional cell carcinoma, micropapillary
ADENOCARCINOMA, NOS	814	8140/2	Adenocarcinoma in situ
		8140/3	Adenocarcinoma, NOS
		8141/3	Scirrhous adenocarcinoma
		8143/3	Superficial spreading adenocarcinoma
		8147/3	Basal cell adenocarcinoma
ADENOID CYSTIC & CRIBRIFORM CA.	820	8200/3	Adenoid cystic carcinoma
		8201/2	Cribriform carcinoma in situ
		8201/3	Cribriform carcinoma
BRONCHIOLO-ALVEOLAR ADENOCA.	825	8255/3	Adenocarcinoma with mixed subtypes
OXYPHILIC ADENOCARCINOMA	829	8290/3	Oxyphilic adenocarcinoma
MUCOEPIDERMOID CARCINOMA	843	8430/3	Mucoepidermoid carcinoma
NEVI & MELANOMAS	872	8720/2	Melanoma in situ
		8720/3	Malignant melanoma, NOS
		8721/3	Nodular melanoma
		8722/3	Balloon cell melanoma
		8723/3	Malignant melanoma, regressing
AMELANOTIC MELANOMA	873	8730/3	Amelanotic melanoma
MAL. MEL. IN JUNCT. NEVUS	874	8743/3	Superficial spreading melanoma
		8745/3	Desmoplastic melanoma, malignant
		8746/3	Mucosal lentiginous melanoma
EPITHELIOID CELL MELANOMA	877	8770/3	Mixed epithel. & spindle cell melanoma
		8771/3	Epithelioid cell melanoma
		8772/3	Spindle cell melanoma, NOS
FIBROMATOUS NEOPLASMS	881	8810/3	Fibrosarcoma, NOS
		8811/3	Fibromyxosarcoma
		8813/3	Fascial fibrosarcoma
		8814/3	Infantile fibrosarcoma
		8815/3	Solitary fibrous tumor, malignant
SARCOMA, NOS	882	8825/3	Myofibroblastic sarcoma

RETINA C692

MYOMATOUS NEOPLASMS	889	8890/3	Leiomyosarcoma, NOS
		8891/3	Epithelioid leiomyosarcoma
		8894/3	Angiomyosarcoma
		8895/3	Myosarcoma
		8896/3	Myxoid leiomyosarcoma
RHABDOMYOSARCOMA, NOS	890	8900/3	Rhabdomyosarcoma, NOS
		8901/3	Pleomorphic rhabdomyosarcoma, adult type
		8902/3	Mixed type rhabdomyosarcoma
EMBRYONAL RHABDOMYOSARCOMA	891	8910/3	Embryonal rhabdomyosarcoma
		8912/3	Spindle cell rhabdomyosarcoma
ALVEOLAR RHABDOMYOSARCOMA	892	8920/3	Alveolar rhabdomyosarcoma
		8921/3	Rhabdomyosarcoma with ganglionic differentiation
MIXED TUMOR, MALIGNANT, NOS	894	8940/3	Mixed tumor, malignant, NOS
		8941/3	Carcinoma in pleomorphic adenoma
MESENCHYMOMA, MALIGNANT	899	8990/3	Mesenchymoma, malignant
		8991/3	Embryonal sarcoma
HEMANGIOPERICYTOMA	915	9150/3	Hemangiopericytoma, malignant
RETINOBLASTOMA, NOS	951	9510/3	Retinoblastoma, NOS
		9511/3	Retinoblastoma, differentiated
		9512/3	Retinoblastoma, undifferentiated
		9513/3	Retinoblastoma, diffuse
MALIGNANT LYMPHOMA, NOS	959	9590/3	Malignant lymphoma, NOS
		9591/3	Malignant lymphoma, non-Hodgkin
		9596/3	Composite Hodgkin and non-Hodgkin lymphoma
HODGKIN LYMPHOMA	965	9650/3	Hodgkin lymphoma, NOS
		9651/3	Hodgkin lymphoma, lymphocyte-rich
		9652/3	Hodgkin lymphoma, mixed cellularity, NOS
		9653/3	Hodgkin lymphoma, lymphocytic deplet., NOS
		9654/3	Hodgkin lymph., lymphocyt. deplet., diffuse fibrosis
		9655/3	Hodgkin lymphoma, lymphocyt. deplet., reticular
		9659/3	Hodgkin lymph., nodular lymphocyte predom.
HODGKIN LYMPHOMA, NOD. SCLER.	966	9661/3	Hodgkin granuloma [obs]
		9662/3	Hodgkin sarcoma [obs]
		9663/3	Hodgkin lymphoma, nodular sclerosis, NOS
		9664/3	Hodgkin lymphoma, nod. scler., cellular phase
		9665/3	Hodgkin lymphoma, nod. scler., grade 1
		9667/3	Hodgkin lymphoma, nod. scler., grade 2

RETINA C692

ML, SMALL B-CELL LYMPHOCYTIC	967	9670/3	ML, small B lymphocytic, NOS
		9671/3	ML, lymphoplasmacytic
		9673/3	Mantle cell lymphoma
		9675/3	ML, mixed sm. and lg. cell, diffuse
ML, LARGE B-CELL, DIFFUSE	968	9680/3	ML, large B-cell, diffuse
		9684/3	ML, large B-cell, diffuse, immunoblastic, NOS
		9687/3	Burkitt lymphoma, NOS
		9688/3	T-cell histiocyte rich large B-cell lymphoma
FOLLIC. & MARGINAL LYMPH, NOS	969	9690/3	Follicular lymphoma, NOS
		9691/3	Follicular lymphoma, grade 2
		9695/3	Follicular lymphoma, grade 1
		9698/3	Follicular lymphoma, grade 3
		9699/3	Marginal zone B-cell lymphoma, NOS
T-CELL LYMPHOMAS	970	9701/3	Sezary syndrome
		9702/3	Mature T-cell lymphoma, NOS
		9705/3	Angioimmunoblastic T-cell lymphoma
OTHER SPEC. NON-HODGKIN LYMPHOMA	971	9712/3	Intravascular large B-cell lymphoma
		9714/3	Anaplastic large cell lymphoma, T-cell and Null cell type
		9719/3	NK/T-cell lymphoma, nasal and nasal-type
PRECURS. CELL LYMPHOBLASTIC LYMPH.	972	9724/3	SystemicEBV pos. T-cell lymphoproliferative disease of childhood
		9727/3	Precursor cell lymphoblastic lymphoma, NOS
		9728/3	Precursor B-cell lymphoblastic lymphoma
		9729/3	Precursor T-cell lymphoblastic lymphoma
PLASMA CELL TUMORS	973	9731/3	Plasmacytoma, NOS
		9734/3	Plasmacytoma, extramedullary
		9735/3	Plasmablastic lymphoma
		9737/3	ALK positive large B-cell lymphoma
		9738/3	Lrg B-cell lymphoma in HHV8-assoc. multicentric Castleman DZ
MAST CELL TUMORS	974	9740/3	Mast cell sarcoma
		9741/3	Malignant mastocytosis
NEOPLASMS OF HISTIOCYTES AND ACCESSORY LYMPHOID CELLS	975	9750/3	Malignant histiocytosis
		9751/3	Langerhans cell histiocytosis, NOS
		9754/3	Langerhans cell histiocytosis, disseminated
		9755/3	Histiocytic sarcoma
		9756/3	Langerhans cell sarcoma
		9757/3	Interdigitating dendritic cell sarcoma
		9758/3	Follicular dendritic cell sarcoma
		9759/3	Fibroblastic reticular cell tumor

RETINA C692

PRECURSOR LYMPHOID NEOPLASMS	981	9811/3	B lymphoblastic leukemia/lymphoma, NOS
		9812/3	Leukemia/lymphoma with t(9;22)(q34;q11.2);BCR-ABL1
		9813/3	Leukemia/lymphoma with t(v;11q23);MLL rearranged
		9814/3	Leukemia/lymphoma with t(12;21)(p13;q22);TEL-AML1(ETV6-RUNX1)
		9815/3	B lymphoblastic leukemia/lymphoma with hyperdiploidy
		9816/3	Leukemia/lymphoma with hypodiploidy (hypodiploid ALL)
		9817/3	B lymphblastic leukemia/lymphoma with t(5;14)(q31;q32);IL3-IGH
		9818/3	Leukemia/lymphoma with t(1;19)(q23;p13.3); E2A PBX1 (TCF3 PBX1)
LYMPHOID LEUKEMIA, NOS	982	9823/3	Chronic lymphocytic leukemia/small lymphocytic lymphoma
PROLYMPH/PRECURS LEUKEMIA	983	9831/3	T-cell large granular lymphocytic leukemia
		9837/3	T lymphoblastic leukemia/lymphoma
CHRONIC MYELOPROLIFERATIVE DIS.	996	9965/3	Myeloid and lymphoid neoplasms with PDGFRB rearrangement
		9967/3	Myeloid and lymphoid neoplasm with FGFR1 abnormalities
MYELOPLASTIC/MYELOPROLIFERATIVE NEOPLASMS	997	9971/3	Polymorphic PTLD
		9975/3	Myelodysplastic/Myeloproliferative neoplasm, unclassifiable

EYEBALL C694

NEOPLASM	800	8000/3	Neoplasm, malignant
		8001/3	Tumor cells, malignant
		8002/3	Malignant tumor, small cell type
		8003/3	Malignant tumor, giant cell type
		8004/3	Malignant tumor, spindle cell type
		8005/3	Malignant tumor, clear cell type
CARCINOMA, NOS	801	8010/2	Carcinoma in situ, NOS
		8010/3	Carcinoma, NOS
		8011/3	Epithelioma, malignant
		8012/3	Large cell carcinoma, NOS
		8013/3	Large cell neuroendocrine carcinoma
		8014/3	Large cell carcinoma with rhabdoid phenotype
		8015/3	Glassy cell carcinoma
CARCINOMA, UNDIFF., NOS	802	8020/3	Carcinoma, undifferentiated type, NOS
		8021/3	Carcinoma, anaplastic type, NOS
		8022/3	Pleomorphic carcinoma
PAPILLARY CARCINOMA, NOS	805	8050/2	Papillary carcinoma in situ
		8050/3	Papillary carcinoma, NOS
		8051/3	Verrucous carcinoma, NOS
		8052/2	Papillary squamous cell carcinoma, non-invasive
		8052/3	Papillary squamous cell carcinoma
SQUAMOUS CELL CARCINOMA, NOS	807	8070/2	Squamous cell carcinoma in situ, NOS
		8070/3	Squamous cell carcinoma, NOS
		8071/3	Sq. cell carcinoma, keratinizing, NOS
		8072/3	Sq. cell carcinoma, lg. cell, non-ker.
		8073/3	Sq. cell carcinoma, sm. cell, non-ker.
		8074/3	Sq. cell carcinoma, spindle cell
		8075/3	Squamous cell carcinoma, adenoid
		8076/2	Sq. cell carc. in situ with question. stromal invas.
		8076/3	Sq. cell carcinoma, micro-invasive
		8078/3	Squamous cell carcinoma with horn formation
LYMPHOEPITHELIAL CARCINOMA	808	8081/2	Bowen disease
		8082/3	Lymphoepithelial carcinoma
		8083/3	Basaloid squamous cell carcinoma
		8084/3	Squamous cell carcinoma, clear cell type
TRANSITIONAL CELL CARCINOMA, NOS	812	8120/2	Transitional cell carcinoma in situ
		8120/3	Transitional cell carcinoma, NOS
		8121/3	Schneiderian carcinoma
		8122/3	Trans. cell carcinoma, spindle cell
		8123/3	Basaloid carcinoma
		8124/3	Cloacogenic carcinoma

EYEBALL C694

PAPILLARY TRANS. CELL CARCINOMA	813	8130/2	Papillary trans. cell carcinoma, non-invasive
		8130/3	Papillary trans. cell carcinoma
		8131/3	Transitional cell carcinoma, micropapillary
ADENOCARCINOMA, NOS	814	8140/2	Adenocarcinoma in situ
		8140/3	Adenocarcinoma, NOS
		8141/3	Scirrhous adenocarcinoma
		8143/3	Superficial spreading adenocarcinoma
		8147/3	Basal cell adenocarcinoma
ADENOID CYSTIC & CRIBRIFORM CA.	820	8200/3	Adenoid cystic carcinoma
		8201/2	Cribriform carcinoma in situ
		8201/3	Cribriform carcinoma
BRONCHIOLO-ALVEOLAR ADENOCA.	825	8255/3	Adenocarcinoma with mixed subtypes
OXYPHILIC ADENOCARCINOMA	829	8290/3	Oxyphilic adenocarcinoma
MUCOEPIDERMOID CARCINOMA	843	8430/3	Mucoepidermoid carcinoma
NEVI & MELANOMAS	872	8720/2	Melanoma in situ
		8720/3	Malignant melanoma, NOS
		8721/3	Nodular melanoma
		8722/3	Balloon cell melanoma
		8723/3	Malignant melanoma, regressing
AMELANOTIC MELANOMA	873	8730/3	Amelanotic melanoma
MAL. MEL. IN JUNCT. NEVUS	874	8743/3	Superficial spreading melanoma
		8745/3	Desmoplastic melanoma, malignant
		8746/3	Mucosal lentiginous melanoma
EPITHELIOID CELL MELANOMA	877	8770/3	Mixed epithel. & spindle cell melanoma
		8771/3	Epithelioid cell melanoma
		8772/3	Spindle cell melanoma, NOS
		8773/3	Spindle cell melanoma, type A
		8774/3	Spindle cell melanoma, type B
FIBROMATOUS NEOPLASMS	881	8810/3	Fibrosarcoma, NOS
		8811/3	Fibromyxosarcoma
		8813/3	Fascial fibrosarcoma
		8814/3	Infantile fibrosarcoma
		8815/3	Solitary fibrous tumor, malignant
SARCOMA, NOS	882	8825/3	Myofibroblastic sarcoma

EYEBALL C694

MYOMATOUS NEOPLASMS	889	8890/3	Leiomyosarcoma, NOS
		8891/3	Epithelioid leiomyosarcoma
		8894/3	Angiomyosarcoma
		8895/3	Myosarcoma
		8896/3	Myxoid leiomyosarcoma
RHABDOMYOSARCOMA, NOS	890	8900/3	Rhabdomyosarcoma, NOS
		8901/3	Pleomorphic rhabdomyosarcoma, adult type
		8902/3	Mixed type rhabdomyosarcoma
EMBRYONAL RHABDOMYOSARCOMA	891	8910/3	Embryonal rhabdomyosarcoma
		8912/3	Spindle cell rhabdomyosarcoma
ALVEOLAR RHABDOMYOSARCOMA	892	8920/3	Alveolar rhabdomyosarcoma
		8921/3	Rhabdomyosarcoma with ganglionic differentiation
MIXED TUMOR, MALIGNANT, NOS	894	8940/3	Mixed tumor, malignant, NOS
		8941/3	Carcinoma in pleomorphic adenoma
MESENCHYMOMA, MALIGNANT	899	8990/3	Mesenchymoma, malignant
		8991/3	Embryonal sarcoma
HEMANGIOPERICYTOMA	915	9150/3	Hemangiopericytoma, malignant
MALIGNANT LYMPHOMA, NOS	959	9590/3	Malignant lymphoma, NOS
		9591/3	Malignant lymphoma, non-Hodgkin
		9596/3	Composite Hodgkin and non-Hodgkin lymphoma
HODGKIN LYMPHOMA	965	9650/3	Hodgkin lymphoma, NOS
		9651/3	Hodgkin lymphoma, lymphocyte-rich
		9652/3	Hodgkin lymphoma, mixed cellularity, NOS
		9653/3	Hodgkin lymphoma, lymphocytic deplet., NOS
		9654/3	Hodgkin lymph., lymphocyt. deplet., diffuse fibrosis
		9655/3	Hodgkin lymphoma, lymphocyt. deplet., reticular
		9659/3	Hodgkin lymph., nodular lymphocyte predom.
HODGKIN LYMPHOMA, NOD. SCLER.	966	9661/3	Hodgkin granuloma [obs]
		9662/3	Hodgkin sarcoma [obs]
		9663/3	Hodgkin lymphoma, nodular sclerosis, NOS
		9664/3	Hodgkin lymphoma, nod. scler., cellular phase
		9665/3	Hodgkin lymphoma, nod. scler., grade 1
		9667/3	Hodgkin lymphoma, nod. scler., grade 2

EYEBALL C694

ML, SMALL B-CELL LYMPHOCYTIC	967	9670/3	ML, small B lymphocytic, NOS
		9671/3	ML, lymphoplasmacytic
		9673/3	Mantle cell lymphoma
		9675/3	ML, mixed sm. and lg. cell, diffuse
ML, LARGE B-CELL, DIFFUSE	968	9680/3	ML, large B-cell, diffuse
		9684/3	ML, large B-cell, diffuse, immunoblastic, NOS
		9687/3	Burkitt lymphoma, NOS
		9688/3	T-cell histiocyte rich large B-cell lymphoma
FOLLIC. & MARGINAL LYMPH, NOS	969	9690/3	Follicular lymphoma, NOS
		9691/3	Follicular lymphoma, grade 2
		9695/3	Follicular lymphoma, grade 1
		9698/3	Follicular lymphoma, grade 3
		9699/3	Marginal zone B-cell lymphoma, NOS
T-CELL LYMPHOMAS	970	9701/3	Sezary syndrome
		9702/3	Mature T-cell lymphoma, NOS
		9705/3	Angioimmunoblastic T-cell lymphoma
OTHER SPEC. NON-HODGKIN LYMPHOMA	971	9712/3	Intravascular large B-cell lymphoma
		9714/3	Anaplastic large cell lymphoma, T-cell and Null cell type
		9719/3	NK/T-cell lymphoma, nasal and nasal-type
PRECURS. CELL LYMPHOBLASTIC LYMPH.	972	9724/3	SystemicEBV pos. T-cell lymphoproliferative disease of childhood
		9727/3	Precursor cell lymphoblastic lymphoma, NOS
		9728/3	Precursor B-cell lymphoblastic lymphoma
		9729/3	Precursor T-cell lymphoblastic lymphoma
PLASMA CELL TUMORS	973	9731/3	Plasmacytoma, NOS
		9734/3	Plasmacytoma, extramedullary
		9735/3	Plasmablastic lymphoma
		9737/3	ALK positive large B-cell lymphoma
		9738/3	Lrg B-cell lymphoma in HHV8-assoc. multicentric Castleman DZ
MAST CELL TUMORS	974	9740/3	Mast cell sarcoma
		9741/3	Malignant mastocytosis
NEOPLASMS OF HISTIOCYTES AND ACCESSORY LYMPHOID CELLS	975	9750/3	Malignant histiocytosis
		9751/3	Langerhans cell histiocytosis, NOS
		9754/3	Langerhans cell histiocytosis, disseminated
		9755/3	Histiocytic sarcoma
		9756/3	Langerhans cell sarcoma
		9757/3	Interdigitating dendritic cell sarcoma
		9758/3	Follicular dendritic cell sarcoma
		9759/3	Fibroblastic reticular cell tumor

EYEBALL C694

PRECURSOR LYMPHOID NEOPLASMS	981	9811/3	B lymphoblastic leukemia/lymphoma, NOS
		9812/3	Leukemia/lymphoma with t(9;22)(q34;q11.2);BCR-ABL1
		9813/3	Leukemia/lymphoma with t(v;11q23);MLL rearranged
		9814/3	Leukemia/lymphoma with t(12;21)(p13;q22);TEL-AML1(ETV6-RUNX1)
		9815/3	B lymphoblastic leukemia/lymphoma with hyperdiploidy
		9816/3	Leukemia/lymphoma with hypodiploidy (hypodiploid ALL)
		9817/3	B lymphblastic leukemia/lymphoma with t(5;14)(q31;q32);IL3-IGH
		9818/3	Leukemia/lymphoma with t(1;19)(q23;p13.3); E2A PBX1 (TCF3 PBX1)
LYMPHOID LEUKEMIA, NOS	982	9823/3	Chronic lymphocytic leukemia/small lymphocytic lymphoma
PROLYMPH/PRECURS LEUKEMIA	983	9831/3	T-cell large granular lymphocytic leukemia
		9837/3	T lymphoblastic leukemia/lymphoma
CHRONIC MYELOPROLIFERATIVE DIS.	996	9965/3	Myeloid and lymphoid neoplasms with PDGFRB rearrangement
		9967/3	Myeloid and lymphoid neoplasm with FGFR1 abnormalities
MYELOPLASTIC/MYELOPROLIFERATIVE NEOPLASMS	997	9971/3	Polymorphic PTLD
		9975/3	Myelodysplastic/Myeloproliferative neoplasm, unclassifiable

EYE, NOS C699

NEOPLASM	800	8000/3	Neoplasm, malignant
		8001/3	Tumor cells, malignant
		8002/3	Malignant tumor, small cell type
		8003/3	Malignant tumor, giant cell type
		8004/3	Malignant tumor, spindle cell type
		8005/3	Malignant tumor, clear cell type
CARCINOMA, NOS	801	8010/2	Carcinoma in situ, NOS
		8010/3	Carcinoma, NOS
		8011/3	Epithelioma, malignant
		8012/3	Large cell carcinoma, NOS
		8013/3	Large cell neuroendocrine carcinoma
		8014/3	Large cell carcinoma with rhabdoid phenotype
		8015/3	Glassy cell carcinoma
CARCINOMA, UNDIFF., NOS	802	8020/3	Carcinoma, undifferentiated type, NOS
		8021/3	Carcinoma, anaplastic type, NOS
		8022/3	Pleomorphic carcinoma
PAPILLARY CARCINOMA, NOS	805	8050/2	Papillary carcinoma in situ
		8050/3	Papillary carcinoma, NOS
		8051/3	Verrucous carcinoma, NOS
		8052/2	Papillary squamous cell carcinoma, non-invasive
		8052/3	Papillary squamous cell carcinoma
SQUAMOUS CELL CARCINOMA, NOS	807	8070/2	Squamous cell carcinoma in situ, NOS
		8070/3	Squamous cell carcinoma, NOS
		8071/3	Sq. cell carcinoma, keratinizing, NOS
		8072/3	Sq. cell carcinoma, lg. cell, non-ker.
		8073/3	Sq. cell carcinoma, sm. cell, non-ker.
		8074/3	Sq. cell carcinoma, spindle cell
		8075/3	Squamous cell carcinoma, adenoid
		8076/2	Sq. cell carc. in situ with question. stromal invas.
		8076/3	Sq. cell carcinoma, micro-invasive
		8078/3	Squamous cell carcinoma with horn formation
TRANSITIONAL CELL CARCINOMA, NOS	812	8120/2	Transitional cell carcinoma in situ
		8120/3	Transitional cell carcinoma, NOS
		8121/3	Schneiderian carcinoma
		8122/3	Trans. cell carcinoma, spindle cell
		8123/3	Basaloid carcinoma
		8124/3	Cloacogenic carcinoma

EYE, NOS C699

PAPILLARY TRANS. CELL CARCINOMA	813	8130/2	Papillary trans. cell carcinoma, non-invasive
		8130/3	Papillary trans. cell carcinoma
		8131/3	Transitional cell carcinoma, micropapillary
ADENOCARCINOMA, NOS	814	8140/2	Adenocarcinoma in situ
		8140/3	Adenocarcinoma, NOS
		8141/3	Scirrhous adenocarcinoma
		8143/3	Superficial spreading adenocarcinoma
		8147/3	Basal cell adenocarcinoma
ADENOID CYSTIC & CRIBRIFORM CA.	820	8200/3	Adenoid cystic carcinoma
		8201/2	Cribriform carcinoma in situ
		8201/3	Cribriform carcinoma
BRONCHIOLO-ALVEOLAR ADENOCA.	825	8255/3	Adenocarcinoma with mixed subtypes
OXYPHILIC ADENOCARCINOMA	829	8290/3	Oxyphilic adenocarcinoma
MUCOEPIDERMOID CARCINOMA	843	8430/3	Mucoepidermoid carcinoma
NEVI & MELANOMAS	872	8720/2	Melanoma in situ
		8720/3	Malignant melanoma, NOS
		8721/3	Nodular melanoma
		8722/3	Balloon cell melanoma
		8723/3	Malignant melanoma, regressing
AMELANOTIC MELANOMA	873	8730/3	Amelanotic melanoma
MAL. MEL. IN JUNCT. NEVUS	874	8743/3	Superficial spreading melanoma
		8745/3	Desmoplastic melanoma, malignant
		8746/3	Mucosal lentiginous melanoma
EPITHELIOID CELL MELANOMA	877	8770/3	Mixed epithel. & spindle cell melanoma
		8771/3	Epithelioid cell melanoma
		8772/3	Spindle cell melanoma, NOS
FIBROMATOUS NEOPLASMS	881	8810/3	Fibrosarcoma, NOS
		8811/3	Fibromyxosarcoma
		8813/3	Fascial fibrosarcoma
		8814/3	Infantile fibrosarcoma
		8815/3	Solitary fibrous tumor, malignant
SARCOMA, NOS	882	8825/3	Myofibroblastic sarcoma

EYE, NOS C699

MYOMATOUS NEOPLASMS	889	8890/3	Leiomyosarcoma, NOS
		8891/3	Epithelioid leiomyosarcoma
		8894/3	Angiomyosarcoma
		8895/3	Myosarcoma
		8896/3	Myxoid leiomyosarcoma
RHABDOMYOSARCOMA, NOS	890	8900/3	Rhabdomyosarcoma, NOS
		8901/3	Pleomorphic rhabdomyosarcoma, adult type
		8902/3	Mixed type rhabdomyosarcoma
EMBRYONAL RHABDOMYOSARCOMA	891	8910/3	Embryonal rhabdomyosarcoma
		8912/3	Spindle cell rhabdomyosarcoma
ALVEOLAR RHABDOMYOSARCOMA	892	8920/3	Alveolar rhabdomyosarcoma
		8921/3	Rhabdomyosarcoma with ganglionic differentiation
MIXED TUMOR, MALIGNANT, NOS	894	8940/3	Mixed tumor, malignant, NOS
		8941/3	Carcinoma in pleomorphic adenoma
MESENCHYMOMA, MALIGNANT	899	8990/3	Mesenchymoma, malignant
		8991/3	Embryonal sarcoma
HEMANGIOPERICYTOMA	915	9150/3	Hemangiopericytoma, malignant
RETINOBLASTOMA, NOS	951	9510/3	Retinoblastoma, NOS
		9511/3	Retinoblastoma, differentiated
		9512/3	Retinoblastoma, undifferentiated
		9513/3	Retinoblastoma, diffuse
MALIGNANT LYMPHOMA, NOS	959	9590/3	Malignant lymphoma, NOS
		9591/3	Malignant lymphoma, non-Hodgkin
		9596/3	Composite Hodgkin and non-Hodgkin lymphoma
HODGKIN LYMPHOMA	965	9650/3	Hodgkin lymphoma, NOS
		9651/3	Hodgkin lymphoma, lymphocyte-rich
		9652/3	Hodgkin lymphoma, mixed cellularity, NOS
		9653/3	Hodgkin lymphoma, lymphocytic deplet., NOS
		9654/3	Hodgkin lymph., lymphocyt. deplet., diffuse fibrosis
		9655/3	Hodgkin lymphoma, lymphocyt. deplet., reticular
		9659/3	Hodgkin lymph., nodular lymphocyte predom.
HODGKIN LYMPHOMA, NOD. SCLER.	966	9661/3	Hodgkin granuloma [obs]
		9662/3	Hodgkin sarcoma [obs]
		9663/3	Hodgkin lymphoma, nodular sclerosis, NOS
		9664/3	Hodgkin lymphoma, nod. scler., cellular phase
		9665/3	Hodgkin lymphoma, nod. scler., grade 1
		9667/3	Hodgkin lymphoma, nod. scler., grade 2

EYE, NOS C699

ML, SMALL B-CELL LYMPHOCYTIC	967	9670/3	ML, small B lymphocytic, NOS
		9671/3	ML, lymphoplasmacytic
		9673/3	Mantle cell lymphoma
		9675/3	ML, mixed sm. and lg. cell, diffuse
ML, LARGE B-CELL, DIFFUSE	968	9680/3	ML, large B-cell, diffuse
		9684/3	ML, large B-cell, diffuse, immunoblastic, NOS
		9687/3	Burkitt lymphoma, NOS
		9688/3	T-cell histiocyte rich large B-cell lymphoma
FOLLIC. & MARGINAL LYMPH, NOS	969	9690/3	Follicular lymphoma, NOS
		9691/3	Follicular lymphoma, grade 2
		9695/3	Follicular lymphoma, grade 1
		9698/3	Follicular lymphoma, grade 3
		9699/3	Marginal zone B-cell lymphoma, NOS
T-CELL LYMPHOMAS	970	9701/3	Sezary syndrome
		9702/3	Mature T-cell lymphoma, NOS
		9705/3	Angioimmunoblastic T-cell lymphoma
OTHER SPEC. NON-HODGKIN LYMPHOMA	971	9712/3	Intravascular large B-cell lymphoma
		9714/3	Anaplastic large cell lymphoma, T-cell and Null cell type
		9719/3	NK/T-cell lymphoma, nasal and nasal-type
PRECURS. CELL LYMPHOBLASTIC LYMPH.	972	9724/3	SystemicEBV pos. T-cell lymphoproliferative disease of childhood
		9727/3	Precursor cell lymphoblastic lymphoma, NOS
		9728/3	Precursor B-cell lymphoblastic lymphoma
		9729/3	Precursor T-cell lymphoblastic lymphoma
PLASMA CELL TUMORS	973	9731/3	Plasmacytoma, NOS
		9734/3	Plasmacytoma, extramedullary
		9735/3	Plasmablastic lymphoma
		9737/3	ALK positive large B-cell lymphoma
		9738/3	Lrg B-cell lymphoma in HHV8-assoc. multicentric Castleman DZ
MAST CELL TUMORS	974	9740/3	Mast cell sarcoma
		9741/3	Malignant mastocytosis
NEOPLASMS OF HISTIOCYTES AND ACCESSORY LYMPHOID CELLS	975	9750/3	Malignant histiocytosis
		9751/3	Langerhans cell histiocytosis, NOS
		9754/3	Langerhans cell histiocytosis, disseminated
		9755/3	Histiocytic sarcoma
		9756/3	Langerhans cell sarcoma
		9757/3	Interdigitating dendritic cell sarcoma
		9758/3	Follicular dendritic cell sarcoma
		9759/3	Fibroblastic reticular cell tumor

EYE, NOS C699

PRECURSOR LYMPHOID NEOPLASMS	981	9811/3	B lymphoblastic leukemia/lymphoma, NOS
		9812/3	Leukemia/lymphoma with t(9;22)(q34;q11.2);BCR-ABL1
		9813/3	Leukemia/lymphoma with t(v;11q23);MLL rearranged
		9814/3	Leukemia/lymphoma with t(12;21)(p13;q22);TEL-AML1(ETV6-RUNX1)
		9815/3	B lymphoblastic leukemia/lymphoma with hyperdiploidy
		9816/3	Leukemia/lymphoma with hypodiploidy (hypodiploid ALL)
		9817/3	B lymphblastic leukemia/lymphoma with t(5;14)(q31;q32);IL3-IGH
		9818/3	Leukemia/lymphoma with t(1;19)(q23;p13.3); E2A PBX1 (TCF3 PBX1)
LYMPHOID LEUKEMIA, NOS	982	9823/3	Chronic lymphocytic leukemia/small lymphocytic lymphoma
PROLYMPH/PRECURS LEUKEMIA	983	9831/3	T-cell large granular lymphocytic leukemia
		9837/3	T lymphoblastic leukemia/lymphoma
CHRONIC MYELOPROLIFERATIVE DIS.	996	9965/3	Myeloid and lymphoid neoplasms with PDGFRB rearrangement
		9967/3	Myeloid and lymphoid neoplasm with FGFR1 abnormalities
MYELOPLASTIC/MYELOPROLIFERATIVE NEOPLASMS	997	9971/3	Polymorphic PTLD
		9975/3	Myelodysplastic/Myeloproliferative neoplasm, unclassifiable

MENINGES (CEREBRAL,SPINAL) C700-C701,C709

NEOPLASM	800	8000/0	Neoplasm, benign
		8000/1	Neoplasm, uncertain whether benign or malignant
		8000/3	Neoplasm, malignant
		8001/0	Tumor cells, benign
		8001/1	Tumor cells, uncertain whether benign or malignant
		8001/3	Tumor cells, malignant
		8005/3	Malignant tumor, clear cell type
NEVI & MELANOMAS	872	8720/3	Malignant melanoma, NOS
		8728/0	Diffuse melanocytosis
		8728/1	Meningeal melanocytoma
		8728/3	Meningeal melanomatosis
SARCOMA, NOS	880	8800/0	Soft tissue tumor, benign
		8800/3	Sarcoma, NOS
		8801/3	Spindle cell sarcoma
		8805/3	Undifferentiated sarcoma
		8806/3	Desmoplastic small round cell tumor
FIBROMATOUS NEOPLASMS	881	8810/0	Fibroma, NOS
		8810/3	Fibrosarcoma, NOS
		8815/0	Solitary fibrous tumor
SARCOMA, NOS	882	8825/3	Myofibroblastic sarcoma
LIPOSARCOMA NEOPLASMS	885	8850/0	Lipoma, NOS
		8851/0	Fibrolipoma
ANGIOLIPOMA	886	8861/0	Angiolipoma, NOS
MYOMATOUS NEOPLASMS	889	8890/3	Leiomyosarcoma, NOS
EMBRYONAL RHABDOMYOSARCOMA	891	8910/3	Embryonal rhabdomyosarcoma
TERATOMA	908	9080/0	Teratoma, benign
		9080/1	Teratoma, NOS
		9080/3	Teratoma, malignant, NOS
		9084/0	Dermoid cyst, NOS
		9084/3	Teratoma with malig. transformation
BLOOD VESSEL TUMORS	912	9120/0	Hemangioma, NOS
		9121/0	Cavernous hemangioma

MENINGES (CEREBRAL,SPINAL) C700-C701,C709

HEMANGIOPERICYTOMA	915	9150/0	Hemangiopericytoma, benign
		9150/1	Hemangiopericytoma, NOS
		9150/3	Hemangiopericytoma, malignant
HEMANGIOBLASTOMA	916	9161/1	Hemangioblastoma
OSSEOUS & CHONDROMATOUS NEOPLASMS	924	9240/3	Mesenchymal chondrosarcoma
MENINGIOMA	953	9530/0	Meningioma, NOS
		9530/1	Meningiomatosis, NOS
		9530/3	Meningioma, malignant
		9531/0	Meningothelial meningioma
		9532/0	Fibrous meningioma
		9533/0	Psammomatous meningioma
		9534/0	Angiomatous meningioma
		9537/0	Transitional meningioma
		9538/1	Clear cell meningioma
		9538/3	Papillary meningioma
		9539/1	Atypical meningioma
		9539/3	Meningeal sarcomatosis
MALIGNANT LYMPHOMA, NOS	959	9590/3	Malignant lymphoma, NOS
		9591/3	Malignant lymphoma, non-Hodgkin
		9596/3	Composite Hodgkin and non-Hodgkin lymphoma
HODGKIN LYMPHOMA	965	9650/3	Hodgkin lymphoma, NOS
		9651/3	Hodgkin lymphoma, lymphocyte-rich
		9652/3	Hodgkin lymphoma, mixed cellularity, NOS
		9653/3	Hodgkin lymphoma, lymphocytic deplet., NOS
		9654/3	Hodgkin lymph., lymphocyt. deplet., diffuse fibrosis
		9655/3	Hodgkin lymphoma, lymphocyt. deplet., reticular
		9659/3	Hodgkin lymph., nodular lymphocyte predom.
HODGKIN LYMPHOMA, NOD. SCLER.	966	9661/3	Hodgkin granuloma [obs]
		9662/3	Hodgkin sarcoma [obs]
		9663/3	Hodgkin lymphoma, nodular sclerosis, NOS
		9664/3	Hodgkin lymphoma, nod. scler., cellular phase
		9665/3	Hodgkin lymphoma, nod. scler., grade 1
		9667/3	Hodgkin lymphoma, nod. scler., grade 2
ML, SMALL B-CELL LYMPHOCYTIC	967	9670/3	ML, small B lymphocytic, NOS
		9671/3	ML, lymphoplasmacytic
		9673/3	Mantle cell lymphoma
		9675/3	ML, mixed sm. and lg. cell, diffuse

MENINGES (CEREBRAL,SPINAL) C700-C701,C709

ML, LARGE B-CELL, DIFFUSE	968	9680/3	ML, large B-cell, diffuse
		9684/3	ML, large B-cell, diffuse, immunoblastic, NOS
		9687/3	Burkitt lymphoma, NOS
		9688/3	T-cell histiocyte rich large B-cell lymphoma
FOLLIC. & MARGINAL LYMPH, NOS	969	9690/3	Follicular lymphoma, NOS
		9691/3	Follicular lymphoma, grade 2
		9695/3	Follicular lymphoma, grade 1
		9698/3	Follicular lymphoma, grade 3
		9699/3	Marginal zone B-cell lymphoma, NOS
T-CELL LYMPHOMAS	970	9701/3	Sezary syndrome
		9702/3	Mature T-cell lymphoma, NOS
		9705/3	Angioimmunoblastic T-cell lymphoma
OTHER SPEC. NON-HODGKIN LYMPHOMA	971	9712/3	Intravascular large B-cell lymphoma
		9714/3	Anaplastic large cell lymphoma, T-cell and Null cell type
		9719/3	NK/T-cell lymphoma, nasal and nasal-type
PRECURS. CELL LYMPHOBLASTIC LYMPH.	972	9724/3	SystemicEBV pos. T-cell lymphoproliferative disease of childhood
		9727/3	Precursor cell lymphoblastic lymphoma, NOS
		9728/3	Precursor B-cell lymphoblastic lymphoma
		9729/3	Precursor T-cell lymphoblastic lymphoma
PLASMA CELL TUMORS	973	9731/3	Plasmacytoma, NOS
		9734/3	Plasmacytoma, extramedullary
		9735/3	Plasmablastic lymphoma
		9737/3	ALK positive large B-cell lymphoma
		9738/3	Lrg B-cell lymphoma in HHV8-assoc. multicentric Castleman DZ
MAST CELL TUMORS	974	9740/3	Mast cell sarcoma
		9741/3	Malignant mastocytosis
NEOPLASMS OF HISTIOCYTES AND ACCESSORY LYMPHOID CELLS	975	9750/3	Malignant histiocytosis
		9751/3	Langerhans cell histiocytosis, NOS
		9754/3	Langerhans cell histiocytosis, disseminated
		9755/3	Histiocytic sarcoma
		9756/3	Langerhans cell sarcoma
		9757/3	Interdigitating dendritic cell sarcoma
		9758/3	Follicular dendritic cell sarcoma
		9759/3	Fibroblastic reticular cell tumor
PRECURSOR LYMPHOID NEOPLASMS	981	9811/3	B lymphoblastic leukemia/lymphoma, NOS
		9812/3	Leukemia/lymphoma with t(9;22)(q34;q11.2);BCR-ABL1
		9813/3	Leukemia/lymphoma with t(v;11q23);MLL rearranged
		9814/3	Leukemia/lymphoma with t(12;21)(p13;q22);TEL-AML1(ETV6-RUNX1)
		9815/3	B lymphoblastic leukemia/lymphoma with hyperdiploidy

MENINGES (CEREBRAL,SPINAL) C700-C701,C709

PRECURSOR LYMPHOID NEOPLASMS	981	9816/3	Leukemia/lymphoma with hypodiploidy (hypodiploid ALL)
		9817/3	B lymphblastic leukemia/lymphoma with t(5;14)(q31;q32);IL3-IGH
		9818/3	Leukemia/lymphoma with t(1;19)(q23;p13.3); E2A PBX1 (TCF3 PBX1)
LYMPHOID LEUKEMIA, NOS	982	9823/3	Chronic lymphocytic leukemia/small lymphocytic lymphoma
PROLYMPH/PRECURS LEUKEMIA	983	9831/3	T-cell large granular lymphocytic leukemia
		9837/3	T lymphoblastic leukemia/lymphoma
CHRONIC MYELOPROLIFERATIVE DIS.	996	9965/3	Myeloid and lymphoid neoplasms with PDGFRB rearrangement
		9967/3	Myeloid and lymphoid neoplasm with FGFR1 abnormalities
MYELOPLASTIC/MYELOPROLIFERATIVE NEOPLASMS	997	9971/3	Polymorphic PTLD
		9975/3	Myelodysplastic/Myeloproliferative neoplasm, unclassifiable

BRAIN, & CRANIAL NERVES, & SPINAL CORD, (EXCL. VENTRICLE, CEREBELLUM) C710-C714, C717-C719, C720-C725

NEOPLASM	800	8000/0	Neoplasm, benign
		8000/1	Neoplasm, uncertain whether benign or malignant
		8000/3	Neoplasm, malignant
		8001/0	Tumor cells, benign
		8001/1	Tumor cells, uncertain whether benign or malignant
		8001/3	Tumor cells, malignant
		8002/3	Malignant tumor, small cell type
		8003/3	Malignant tumor, giant cell type
		8004/3	Malignant tumor, spindle cell type
		8005/3	Malignant tumor, clear cell type
PARAGANGLIOMA	868	8680/1	Paraganglioma, NOS
NEVI & MELANOMAS	872	8720/3	Malignant melanoma, NOS
SARCOMA, NOS	880	8800/0	Soft tissue tumor, benign
		8800/3	Sarcoma, NOS
		8801/3	Spindle cell sarcoma
		8805/3	Undifferentiated sarcoma
		8806/3	Desmoplastic small round cell tumor
FIBROMATOUS NEOPLASMS	881	8815/1	Solitary fibrous tumor/hemangiopericytoma Grade 2
LIPOSARCOMA NEOPLASMS	885	8850/0	Lipoma, NOS
		8851/0	Fibrolipoma
		8851/3	Liposarcoma, well differentiated
GERM CELL TUMORS	906	9060/3	Dysgerminoma
		9064/3	Germinoma
EMBRYONAL CARCINOMA, NOS	907	9070/3	Embryonal carcinoma, NOS
		9071/3	Yolk sac tumor
TERATOMA	908	9080/0	Teratoma, benign
		9080/1	Teratoma, NOS
		9080/3	Teratoma, malignant, NOS
		9081/3	Teratocarcinoma
		9085/3	Mixed germ cell tumor
CHORIOCARCINOMA	910	9100/3	Choriocarcinoma
BLOOD VESSEL TUMORS	912	9120/0	Hemangioma, NOS
		9121/0	Cavernous hemangioma
		9122/0	Venous hemangioma
HEMANGIOENDOTHELIOMA	913	9131/0	Capillary hemangioma

BRAIN, & CRANIAL NERVES, & SPINAL CORD, (EXCL. VENTRICLE, CEREBELLUM) C710-C714, C717-C719, C720-C725

HEMANGIOPERICYTOMA	915	9150/1	Hemangiopericytoma, NOS
HEMANGIOBLASTOMA	916	9161/1	Hemangioblastoma
CHORDOMA	937	9370/3	Chordoma, NOS
		9371/3	Chondroid chordoma
		9372/3	Dedifferentiated chordoma
GLIOMA	938	9380/3	Glioma, malignant
		9381/3	Gliomatosis cerebri
		9382/3	Mixed glioma
		9383/1	Subependymoma
		9384/1	Supependymal giant cell astrocytoma
		9385/3	Diffuse midline glioma, H3 K27M-mutant
EPENDYMOMA, NOS	939	9391/3	Ependymoma, NOS
		9392/3	Ependymoma, anaplastic
		9393/3	Papillary ependymoma
		9394/1	Myxopapillary ependymoma
		9396/3	Ependymoma, RELA fusion-positive
ASTROCYTOMA, NOS	940	9400/3	Astrocytoma, NOS
		9401/3	Astrocytoma, anaplastic
PROTOPLASMIC ASTROCYTOMA	941	9410/3	Protoplasmic astrocytoma
		9411/3	Gemistocytic astrocytoma
		9412/1	Desmoplastic infantile astrocytoma
		9413/0	Dysembryoplastic neuroepithelial tumor
FIBRILLARY ASTROCYTOMA	942	9420/3	Fibrillary astrocytoma
		9421/1	Pilocytic astrocytoma
		9421/3	Pilocytic astrocytoma
		9423/3	Polar spongioblastoma
		9424/3	Pleomorphic xanthoastrocytoma
		9425/3	Pilomyxoid astrocytoma
ASTROBLASTOMA	943	9430/3	Astroblastoma
		9431/1	Angiocentric glioma
GLIOBLASTOMA, NOS	944	9440/3	Glioblastoma, NOS
		9441/3	Giant cell glioblastoma
		9442/1	Gliofibroma
		9442/3	Gliosarcoma
		9444/1	Chordoid glioma
		9445/3	Glioblastoma, IDH-mutant

BRAIN, & CRANIAL NERVES, & SPINAL CORD, (EXCL. VENTRICLE, CEREBELLUM) C710-C714, C717-C719, C720-C725

OLIGODENDROGLIOMA, NOS	945	9450/3	Oligodendroglioma, NOS
		9451/3	Oligodendroglioma, anaplastic
OLIGODENDROBLASTOMA	946	9460/3	Oligodendroblastoma
MEDULLOBLASTOMA, NOS	947	9473/3	Primitive neuroectodermal tumor
		9475/3	Medulloblastoma, WNT-activated
		9476/3	Medulloblastoma, SHH-activated and TP53-mutant
		9477/3	Medulloblastoma, non-WNT/non-SHH
		9478/3	Embryonal tumor with multilayered rosettes, NOS
GANGLIONEUROBLASTOMA	949	9490/0	Ganglioneuroma
		9490/3	Ganglioneuroblastoma
		9492/0	Gangliocytoma
NEUROBLASTOMA, NOS	950	9500/3	Neuroblastoma, NOS
		9501/3	Medulloepithelioma, NOS
		9502/3	Teratoid medulloepithelioma
		9503/3	Neuroepithelioma, NOS
		9505/1	Ganglioglioma, NOS
		9505/3	Ganglioglioma, anaplastic
		9508/3	Atypical teratoid/rhabdoid tumor
		9509/1	Papillary glioneuronal tumor
NEUROFIBROSARCOMA	954	9540/0	Neurofibroma, NOS
		9540/1	Neurofibromatosis, NOS
		9540/3	Malignant peripheral nerve sheath tumor
		9541/0	Melanotic neurofibroma
PLEXIFORM NEUROFIBROMA	955	9550/0	Plexiform neurofibroma
NEURILEMMOMA	956	9560/0	Neurilemoma, NOS
		9560/1	Melanotic schwannoma
		9560/3	Neurilemmoma, malignant
		9561/3	MPNST with rhabdomyoblastic differentiation
		9562/0	Neurothekeoma
PERINEURIOMA	957	9570/0	Neuroma, NOS
		9571/0	Perineurioma, NOS
		9571/3	Perineurioma, malignant
MALIGNANT LYMPHOMA, NOS	959	9590/3	Malignant lymphoma, NOS
		9591/3	Malignant lymphoma, non-Hodgkin
		9596/3	Composite Hodgkin and non-Hodgkin lymphoma

BRAIN, & CRANIAL NERVES, & SPINAL CORD, (EXCL. VENTRICLE, CEREBELLUM) C710-C714, C717-C719, C720-C725

ML, SMALL B-CELL LYMPHOCYTIC	967	9670/3	ML, small B lymphocytic, NOS
		9671/3	ML, lymphoplasmacytic
		9673/3	Mantle cell lymphoma
		9675/3	ML, mixed sm. and lg. cell, diffuse
ML, LARGE B-CELL, DIFFUSE	968	9680/3	ML, large B-cell, diffuse
		9684/3	ML, large B-cell, diffuse, immunoblastic, NOS
		9687/3	Burkitt lymphoma, NOS
		9688/3	T-cell histiocyte rich large B-cell lymphoma
FOLLIC. & MARGINAL LYMPH, NOS	969	9690/3	Follicular lymphoma, NOS
		9691/3	Follicular lymphoma, grade 2
		9695/3	Follicular lymphoma, grade 1
		9698/3	Follicular lymphoma, grade 3
		9699/3	Marginal zone B-cell lymphoma, NOS
T-CELL LYMPHOMAS	970	9701/3	Sezary syndrome
		9702/3	Mature T-cell lymphoma, NOS
		9705/3	Angioimmunoblastic T-cell lymphoma
OTHER SPEC. NON-HODGKIN LYMPHOMA	971	9712/3	Intravascular large B-cell lymphoma
		9714/3	Anaplastic large cell lymphoma, T-cell and Null cell type
		9719/3	NK/T-cell lymphoma, nasal and nasal-type
PRECURS. CELL LYMPHOBLASTIC LYMPH.	972	9724/3	SystemicEBV pos. T-cell lymphoproliferative disease of childhood
		9727/3	Precursor cell lymphoblastic lymphoma, NOS
		9728/3	Precursor B-cell lymphoblastic lymphoma
		9729/3	Precursor T-cell lymphoblastic lymphoma
PLASMA CELL TUMORS	973	9731/3	Plasmacytoma, NOS
		9734/3	Plasmacytoma, extramedullary
		9735/3	Plasmablastic lymphoma
		9737/3	ALK positive large B-cell lymphoma
		9738/3	Lrg B-cell lymphoma in HHV8-assoc. multicentric Castleman DZ
NEOPLASMS OF HISTIOCYTES AND ACCESSORY LYMPHOID CELLS	975	9750/3	Malignant histiocytosis
		9751/3	Langerhans cell histiocytosis, NOS
		9754/3	Langerhans cell histiocytosis, disseminated
		9755/3	Histiocytic sarcoma
		9756/3	Langerhans cell sarcoma
		9757/3	Interdigitating dendritic cell sarcoma
		9758/3	Follicular dendritic cell sarcoma
		9759/3	Fibroblastic reticular cell tumor

BRAIN, & CRANIALNERVES, & SPINAL CORD, (EXCL. VENTRICLE, CEREBELLUM) C710-C714, C717-C719, C720-C725

PRECURSOR LYMPHOID NEOPLASMS	981	9811/3	B lymphoblastic leukemia/lymphoma, NOS
		9812/3	Leukemia/lymphoma with t(9;22)(q34;q11.2);BCR-ABL1
		9813/3	Leukemia/lymphoma with t(v;11q23);MLL rearranged
		9814/3	Leukemia/lymphoma with t(12;21)(p13;q22);TEL-AML1(ETV6-RUNX1)
		9815/3	B lymphoblastic leukemia/lymphoma with hyperdiploidy
		9816/3	Leukemia/lymphoma with hypodiploidy (hypodiploid ALL)
		9817/3	B lymphblastic leukemia/lymphoma with t(5;14)(q31;q32);IL3-IGH
		9818/3	Leukemia/lymphoma with t(1;19)(q23;p13.3); E2A PBX1 (TCF3 PBX1)
LYMPHOID LEUKEMIA, NOS	982	9823/3	Chronic lymphocytic leukemia/small lymphocytic lymphoma
PROLYMPH/PRECURS LEUKEMIA	983	9831/3	T-cell large granular lymphocytic leukemia
		9837/3	T lymphoblastic leukemia/lymphoma
MYELOID SARCOMA	993	9930/3	Myeloid sarcoma
CHRONIC MYELOPROLIFERATIVE DIS.	996	9965/3	Myeloid and lymphoid neoplasms with PDGFRB rearrangement
		9967/3	Myeloid and lymphoid neoplasm with FGFR1 abnormalities
MYELOPLASTIC/MYELOPROLIFERATIVE NEOPLASMS	997	9971/3	Polymorphic PTLD
		9975/3	Myelodysplastic/Myeloproliferative neoplasm, unclassifiable

VENTRICLE C715

NEOPLASM	800	8000/0	Neoplasm, benign
		8000/1	Neoplasm, uncertain whether benign or malignant
		8000/3	Neoplasm, malignant
		8001/0	Tumor cells, benign
		8001/1	Tumor cells, uncertain whether benign or malignant
		8001/3	Tumor cells, malignant
		8005/3	Malignant tumor, clear cell type
TERATOMA	908	9085/3	Mixed germ cell tumor
CHORDOMA	937	9370/3	Chordoma, NOS
		9371/3	Chondroid chordoma
		9372/3	Dedifferentiated chordoma
GLIOMA	938	9380/3	Glioma, malignant
		9381/3	Gliomatosis cerebri
		9382/3	Mixed glioma
		9383/1	Subependymoma
		9384/1	Supependymal giant cell astrocytoma
		9385/3	Diffuse midline glioma, H3 K27M-mutant
EPENDYMOMA, NOS	939	9390/0	Choroid plexus papilloma, NOS
		9390/1	Atypical choroid plexus papilloma
		9390/3	Choroid plexus papilloma, malignant
		9391/3	Ependymoma, NOS
		9392/3	Ependymoma, anaplastic
		9393/3	Papillary ependymoma
		9396/3	Ependymoma, RELA fusion-positive
ASTROCYTOMA, NOS	940	9400/3	Astrocytoma, NOS
		9401/3	Astrocytoma, anaplastic
PROTOPLASMIC ASTROCYTOMA	941	9410/3	Protoplasmic astrocytoma
		9411/3	Gemistocytic astrocytoma
FIBRILLARY ASTROCYTOMA	942	9420/3	Fibrillary astrocytoma
		9421/1	Pilocytic astrocytoma
		9421/3	Pilocytic astrocytoma
		9423/3	Polar spongioblastoma
		9424/3	Pleomorphic xanthoastrocytoma
		9425/3	Pilomyxoid astrocytoma
ASTROBLASTOMA	943	9430/3	Astroblastoma
		9431/1	Angiocentric glioma

VENTRICLE C715

GLIOBLASTOMA, NOS	944	9440/3	Glioblastoma, NOS
		9441/3	Giant cell glioblastoma
		9442/3	Gliosarcoma
		9444/1	Chordoid glioma
		9445/3	Glioblastoma, IDH-mutant
OLIGODENDROGLIOMA, NOS	945	9450/3	Oligodendroglioma, NOS
		9451/3	Oligodendroglioma, anaplastic
MEDULLOBLASTOMA, NOS	947	9473/3	Primitive neuroectodermal tumor
		9475/3	Medulloblastoma, WNT-activated
		9476/3	Medulloblastoma, SHH-activated and TP53-mutant
		9477/3	Medulloblastoma, non-WNT/non-SHH
		9478/3	Embryonal tumor with multilayered rosettes, NOS
GANGLIONEUROBLASTOMA	949	9490/0	Ganglioneuroma
		9490/3	Ganglioneuroblastoma
		9492/0	Gangliocytoma
NEUROBLASTOMA, NOS	950	9500/3	Neuroblastoma, NOS
		9501/3	Medulloepithelioma, NOS
		9502/3	Teratoid medulloepithelioma
		9503/3	Neuroepithelioma, NOS
		9505/1	Ganglioglioma, NOS
		9505/3	Ganglioglioma, anaplastic
		9506/1	Centrol neurocytoma
		9508/3	Atypical teratoid/rhabdoid tumor
		9509/1	Papillary glioneuronal tumor
MALIGNANT LYMPHOMA, NOS	959	9590/3	Malignant lymphoma, NOS
		9591/3	Malignant lymphoma, non-Hodgkin
		9596/3	Composite Hodgkin and non-Hodgkin lymphoma
ML, SMALL B-CELL LYMPHOCYTIC	967	9670/3	ML, small B lymphocytic, NOS
		9671/3	ML, lymphoplasmacytic
		9673/3	Mantle cell lymphoma
		9675/3	ML, mixed sm. and lg. cell, diffuse
ML, LARGE B-CELL, DIFFUSE	968	9680/3	ML, large B-cell, diffuse
		9684/3	ML, large B-cell, diffuse, immunoblastic, NOS
		9687/3	Burkitt lymphoma, NOS
		9688/3	T-cell histiocyte rich large B-cell lymphoma

VENTRICLE C715

FOLLIC. & MARGINAL LYMPH, NOS	969	9690/3	Follicular lymphoma, NOS
		9691/3	Follicular lymphoma, grade 2
		9695/3	Follicular lymphoma, grade 1
		9698/3	Follicular lymphoma, grade 3
		9699/3	Marginal zone B-cell lymphoma, NOS
T-CELL LYMPHOMAS	970	9701/3	Sezary syndrome
		9702/3	Mature T-cell lymphoma, NOS
		9705/3	Angioimmunoblastic T-cell lymphoma
OTHER SPEC. NON-HODGKIN LYMPHOMA	971	9712/3	Intravascular large B-cell lymphoma
		9714/3	Anaplastic large cell lymphoma, T-cell and Null cell type
		9719/3	NK/T-cell lymphoma, nasal and nasal-type
PRECURS. CELL LYMPHOBLASTIC LYMPH.	972	9724/3	SystemicEBV pos. T-cell lymphoproliferative disease of childhood
		9727/3	Precursor cell lymphoblastic lymphoma, NOS
		9728/3	Precursor B-cell lymphoblastic lymphoma
		9729/3	Precursor T-cell lymphoblastic lymphoma
PLASMA CELL TUMORS	973	9731/3	Plasmacytoma, NOS
		9734/3	Plasmacytoma, extramedullary
		9735/3	Plasmablastic lymphoma
		9737/3	ALK positive large B-cell lymphoma
		9738/3	Lrg B-cell lymphoma in HHV8-assoc. multicentric Castleman DZ
NEOPLASMS OF HISTIOCYTES AND ACCESSORY LYMPHOID CELLS	975	9750/3	Malignant histiocytosis
		9751/3	Langerhans cell histiocytosis, NOS
		9754/3	Langerhans cell histiocytosis, disseminated
		9755/3	Histiocytic sarcoma
		9756/3	Langerhans cell sarcoma
		9757/3	Interdigitating dendritic cell sarcoma
		9758/3	Follicular dendritic cell sarcoma
		9759/3	Fibroblastic reticular cell tumor
PRECURSOR LYMPHOID NEOPLASMS	981	9811/3	B lymphoblastic leukemia/lymphoma, NOS
		9812/3	Leukemia/lymphoma with t(9;22)(q34;q11.2);BCR-ABL1
		9813/3	Leukemia/lymphoma with t(v;11q23);MLL rearranged
		9814/3	Leukemia/lymphoma with t(12;21)(p13;q22);TEL-AML1(ETV6-RUNX1)
		9815/3	B lymphoblastic leukemia/lymphoma with hyperdiploidy
		9816/3	Leukemia/lymphoma with hypodiploidy (hypodiploid ALL)
		9817/3	B lymphblastic leukemia/lymphoma with t(5;14)(q31;q32);IL3-IGH
		9818/3	Leukemia/lymphoma with t(1;19)(q23;p13.3); E2A PBX1 (TCF3 PBX1)
LYMPHOID LEUKEMIA, NOS	982	9823/3	Chronic lymphocytic leukemia/small lymphocytic lymphoma

VENTRICLE C715

PROLYMPH/PRECURS LEUKEMIA	983	9831/3	T-cell large granular lymphocytic leukemia
		9837/3	T lymphoblastic leukemia/lymphoma
CHRONIC MYELOPROLIFERATIVE DIS.	996	9965/3	Myeloid and lymphoid neoplasms with PDGFRB rearrangement
		9967/3	Myeloid and lymphoid neoplasm with FGFR1 abnormalities
MYELOPLASTIC/MYELOPROLIFERATIVE NEOPLASMS	997	9971/3	Polymorphic PTLD
		9975/3	Myelodysplastic/Myeloproliferative neoplasm, unclassifiable

CEREBELLUM C716

NEOPLASM	800	8000/0	Neoplasm, benign
		8000/1	Neoplasm, uncertain whether benign or malignant
		8000/3	Neoplasm, malignant
		8001/0	Tumor cells, benign
		8001/1	Tumor cells, uncertain whether benign or malignant
		8001/3	Tumor cells, malignant
		8005/3	Malignant tumor, clear cell type
SARCOMA, NOS	880	8800/0	Soft tissue tumor, benign
		8800/3	Sarcoma, NOS
		8805/3	Undifferentiated sarcoma
		8806/3	Desmoplastic small round cell tumor
FIBROMATOUS NEOPLASMS	881	8810/3	Fibrosarcoma, NOS
		8815/0	Solitary fibrous tumor
SARCOMA, NOS	882	8825/3	Myofibroblastic sarcoma
LIPOSARCOMA NEOPLASMS	885	8850/0	Lipoma, NOS
TERATOMA	908	9080/0	Teratoma, benign
		9080/1	Teratoma, NOS
		9080/3	Teratoma, malignant, NOS
		9084/0	Dermoid cyst, NOS
BLOOD VESSEL TUMORS	912	9120/0	Hemangioma, NOS
HEMANGIOENDOTHELIOMA	913	9131/0	Capillary hemangioma
HEMANGIOPERICYTOMA	915	9150/1	Hemangiopericytoma, NOS
HEMANGIOBLASTOMA	916	9161/1	Hemangioblastoma
CHORDOMA	937	9370/3	Chordoma, NOS
		9371/3	Chondroid chordoma
		9372/3	Dedifferentiated chordoma
GLIOMA	938	9380/3	Glioma, malignant
		9381/3	Gliomatosis cerebri
		9382/3	Mixed glioma
		9383/1	Subependymoma
		9385/3	Diffuse midline glioma, H3 K27M-mutant

CEREBELLUM C716

EPENDYMOMA, NOS	939	9391/3	Ependymoma, NOS
		9392/3	Ependymoma, anaplastic
		9393/3	Papillary ependymoma
		9396/3	Ependymoma, RELA fusion-positive
ASTROCYTOMA, NOS	940	9400/3	Astrocytoma, NOS
		9401/3	Astrocytoma, anaplastic
PROTOPLASMIC ASTROCYTOMA	941	9410/3	Protoplasmic astrocytoma
		9411/3	Gemistocytic astrocytoma
FIBRILLARY ASTROCYTOMA	942	9420/3	Fibrillary astrocytoma
		9421/1	Pilocytic astrocytoma
		9421/3	Pilocytic astrocytoma
		9424/3	Pleomorphic xanthoastrocytoma
		9425/3	Pilomyxoid astrocytoma
ASTROBLASTOMA	943	9430/3	Astroblastoma
		9431/1	Angiocentric glioma
GLIOBLASTOMA, NOS	944	9440/3	Glioblastoma, NOS
		9441/3	Giant cell glioblastoma
		9442/3	Gliosarcoma
		9445/3	Glioblastoma, IDH-mutant
OLIGODENDROGLIOMA, NOS	945	9450/3	Oligodendroglioma, NOS
		9451/3	Oligodendroglioma, anaplastic
MEDULLOBLASTOMA, NOS	947	9470/3	Medulloblastoma, NOS
		9471/3	Desmoplastic medulloblastoma
		9472/3	Medullomyoblastoma
		9473/3	Primitive neuroectodermal tumor
		9474/3	Large cell medulloblastoma
		9475/3	Medulloblastoma, WNT-activated
		9476/3	Medulloblastoma, SHH-activated and TP53-mutant
		9477/3	Medulloblastoma, non-WNT/non-SHH
		9478/3	Embryonal tumor with multilayered rosettes, NOS
CEREBELLAR SARCOMA, NOS	948	9480/3	Cerebellar sarcoma, NOS
GANGLIONEUROBLASTOMA	949	9490/0	Ganglioneuroma
		9490/3	Ganglioneuroblastoma
		9492/0	Gangliocytoma
		9493/0	Dysplastic gangliocytoma of cerebellum (Lhermitte-Duclos)

CEREBELLUM C716

NEUROBLASTOMA, NOS	950	9500/3	Neuroblastoma, NOS
		9501/3	Medulloepithelioma, NOS
		9502/3	Teratoid medulloepithelioma
		9503/3	Neuroepithelioma, NOS
		9505/1	Ganglioglioma, NOS
		9506/1	Centrol neurocytoma
		9508/3	Atypical teratoid/rhabdoid tumor
		9509/1	Papillary glioneuronal tumor
MALIGNANT LYMPHOMA, NOS	959	9590/3	Malignant lymphoma, NOS
		9591/3	Malignant lymphoma, non-Hodgkin
		9596/3	Composite Hodgkin and non-Hodgkin lymphoma
ML, SMALL B-CELL LYMPHOCYTIC	967	9670/3	ML, small B lymphocytic, NOS
		9671/3	ML, lymphoplasmacytic
		9673/3	Mantle cell lymphoma
		9675/3	ML, mixed sm. and lg. cell, diffuse
ML, LARGE B-CELL, DIFFUSE	968	9680/3	ML, large B-cell, diffuse
		9684/3	ML, large B-cell, diffuse, immunoblastic, NOS
		9687/3	Burkitt lymphoma, NOS
		9688/3	T-cell histiocyte rich large B-cell lymphoma
FOLLIC. & MARGINAL LYMPH, NOS	969	9690/3	Follicular lymphoma, NOS
		9691/3	Follicular lymphoma, grade 2
		9695/3	Follicular lymphoma, grade 1
		9698/3	Follicular lymphoma, grade 3
		9699/3	Marginal zone B-cell lymphoma, NOS
T-CELL LYMPHOMAS	970	9701/3	Sezary syndrome
		9702/3	Mature T-cell lymphoma, NOS
		9705/3	Angioimmunoblastic T-cell lymphoma
OTHER SPEC. NON-HODGKIN LYMPHOMA	971	9712/3	Intravascular large B-cell lymphoma
		9714/3	Anaplastic large cell lymphoma, T-cell and Null cell type
		9719/3	NK/T-cell lymphoma, nasal and nasal-type
PRECURS. CELL LYMPHOBLASTIC LYMPH.	972	9724/3	SystemicEBV pos. T-cell lymphoproliferative disease of childhood
		9727/3	Precursor cell lymphoblastic lymphoma, NOS
		9728/3	Precursor B-cell lymphoblastic lymphoma
		9729/3	Precursor T-cell lymphoblastic lymphoma
PLASMA CELL TUMORS	973	9731/3	Plasmacytoma, NOS
		9734/3	Plasmacytoma, extramedullary
		9735/3	Plasmablastic lymphoma
		9737/3	ALK positive large B-cell lymphoma
		9738/3	Lrg B-cell lymphoma in HHV8-assoc. multicentric Castleman DZ

CEREBELLUM C716

NEOPLASMS OF HISTIOCYTES AND ACCESSORY LYMPHOID CELLS	975	9750/3	Malignant histiocytosis
		9751/3	Langerhans cell histiocytosis, NOS
		9754/3	Langerhans cell histiocytosis, disseminated
		9755/3	Histiocytic sarcoma
		9756/3	Langerhans cell sarcoma
		9757/3	Interdigitating dendritic cell sarcoma
		9758/3	Follicular dendritic cell sarcoma
		9759/3	Fibroblastic reticular cell tumor
PRECURSOR LYMPHOID NEOPLASMS	981	9811/3	B lymphoblastic leukemia/lymphoma, NOS
		9812/3	Leukemia/lymphoma with t(9;22)(q34;q11.2);BCR-ABL1
		9813/3	Leukemia/lymphoma with t(v;11q23);MLL rearranged
		9814/3	Leukemia/lymphoma with t(12;21)(p13;q22);TEL-AML1(ETV6-RUNX1)
		9815/3	B lymphoblastic leukemia/lymphoma with hyperdiploidy
		9816/3	Leukemia/lymphoma with hypodiploidy (hypodiploid ALL)
		9817/3	B lymphblastic leukemia/lymphoma with t(5;14)(q31;q32);IL3-IGH
		9818/3	Leukemia/lymphoma with t(1;19)(q23;p13.3); E2A PBX1 (TCF3 PBX1)
LYMPHOID LEUKEMIA, NOS	982	9823/3	Chronic lymphocytic leukemia/small lymphocytic lymphoma
PROLYMPH/PRECURS LEUKEMIA	983	9831/3	T-cell large granular lymphocytic leukemia
		9837/3	T lymphoblastic leukemia/lymphoma
CHRONIC MYELOPROLIFERATIVE DIS.	996	9965/3	Myeloid and lymphoid neoplasms with PDGFRB rearrangement
		9967/3	Myeloid and lymphoid neoplasm with FGFR1 abnormalities
MYELOPLASTIC/MYELOPROLIFERATIVE NEOPLASMS	997	9971/3	Polymorphic PTLD
		9975/3	Myelodysplastic/Myeloproliferative neoplasm, unclassifiable

OTHER NERVOUS SYSTEM C728-C729

NEOPLASM	800	8000/0	Neoplasm, benign
		8000/1	Neoplasm, uncertain whether benign or malignant
		8000/3	Neoplasm, malignant
		8001/0	Tumor cells, benign
		8001/1	Tumor cells, uncertain whether benign or malignant
		8001/3	Tumor cells, malignant
		8002/3	Malignant tumor, small cell type
		8003/3	Malignant tumor, giant cell type
		8004/3	Malignant tumor, spindle cell type
		8005/3	Malignant tumor, clear cell type
SARCOMA, NOS	880	8800/0	Soft tissue tumor, benign
		8800/3	Sarcoma, NOS
		8801/3	Spindle cell sarcoma
		8802/3	Giant cell sarcoma
		8803/3	Small cell sarcoma
		8804/3	Epithelioid sarcoma
		8805/3	Undifferentiated sarcoma
		8806/3	Desmoplastic small round cell tumor
LIPOSARCOMA NEOPLASMS	885	8850/0	Lipoma, NOS
		8850/1	Atypical lipoma
		8850/3	Liposarcoma, NOS
ANGIOLIPOMA	886	8861/0	Angiolipoma, NOS
MYOMATOUS NEOPLASMS	889	8890/0	Leiomyoma, NOS
		8890/1	Leiomyomatosis, nOS
		8890/3	Leiomyosarcoma, NOS
		8897/1	Smooth muscle tumor, NOS
RHABDOMYOSARCOMA, NOS	890	8900/0	Rhabdomyoma, NOS
		8900/3	Rhabdomyosarcoma, NOS
EMBRYONAL RHABDOMYOSARCOMA	891	8910/3	Embryonal rhabdomyosarcoma
ALVEOLAR RHABDOMYOSARCOMA	892	8920/3	Alveolar rhabdomyosarcoma
GERM CELL TUMORS	906	9064/3	Germinoma
TERATOMA	908	9080/1	Teratoma, NOS
		9080/3	Teratoma, malignant, NOS
		9082/3	Malignant teratoma, undiff.
		9084/0	Dermoid cyst, NOS
		9084/3	Teratoma with malig. transformation

OTHER NERVOUS SYSTEM C728-C729

BLOOD VESSEL TUMORS	912	9120/0	Hemangioma, NOS
		9120/3	Hemangiosarcoma
		9121/0	Cavernous hemangioma
HEMANGIOENDOTHELIOMA	913	9130/0	Hemangioendothelioma, benign
		9130/1	Hemangioendothelioma, NOS
		9130/3	Hemangioendothelioma, malignant
KAPOSI SARCOMA	914	9140/3	Kaposi sarcoma
HEMANGIOPERICYTOMA	915	9150/0	Hemangiopericytoma, benign
		9150/1	Hemangiopericytoma, NOS
		9150/3	Hemangiopericytoma, malignant
HEMANGIOBLASTOMA	916	9161/1	Hemangioblastoma
EWING SARCOMA	926	9260/3	Ewing sarcoma
CHORDOMA	937	9370/3	Chordoma, NOS
		9371/3	Chondroid chordoma
		9372/3	Dedifferentiated chordoma
NEUROBLASTOMA, NOS	950	9500/3	Neuroblastoma, NOS
		9501/3	Medulloepithelioma, NOS
		9502/3	Teratoid medulloepithelioma
		9503/3	Neuroepithelioma, NOS
		9508/3	Atypical teratoid/rhabdoid tumor
NEUROFIBROSARCOMA	954	9540/0	Neurofibroma, NOS
		9540/1	Neurofibromatosis, NOS
		9540/3	Malignant peripheral nerve sheath tumor
		9541/0	Melanotic neurofibroma
PLEXIFORM NEUROFIBROMA	955	9550/0	Plexiform neurofibroma
NEURILEMMOMA	956	9560/0	Neurilemoma, NOS
		9560/3	Neurilemmoma, malignant
		9561/3	MPNST with rhabdomyoblastic differentiation
		9562/0	Neurothekeoma
PERINEURIOMA	957	9570/0	Neuroma, NOS
		9571/0	Perineurioma, NOS
		9571/3	Perineurioma, malignant

OTHER NERVOUS SYSTEM C728-C729

MALIGNANT LYMPHOMA, NOS	959	9590/3	Malignant lymphoma, NOS
		9591/3	Malignant lymphoma, non-Hodgkin
		9596/3	Composite Hodgkin and non-Hodgkin lymphoma
HODGKIN LYMPHOMA	965	9650/3	Hodgkin lymphoma, NOS
		9651/3	Hodgkin lymphoma, lymphocyte-rich
		9652/3	Hodgkin lymphoma, mixed cellularity, NOS
		9653/3	Hodgkin lymphoma, lymphocytic deplet., NOS
		9654/3	Hodgkin lymph., lymphocyt. deplet., diffuse fibrosis
		9655/3	Hodgkin lymphoma, lymphocyt. deplet., reticular
		9659/3	Hodgkin lymph., nodular lymphocyte predom.
HODGKIN LYMPHOMA, NOD. SCLER.	966	9661/3	Hodgkin granuloma [obs]
		9662/3	Hodgkin sarcoma [obs]
		9663/3	Hodgkin lymphoma, nodular sclerosis, NOS
		9664/3	Hodgkin lymphoma, nod. scler., cellular phase
		9665/3	Hodgkin lymphoma, nod. scler., grade 1
		9667/3	Hodgkin lymphoma, nod. scler., grade 2
ML, SMALL B-CELL LYMPHOCYTIC	967	9670/3	ML, small B lymphocytic, NOS
		9671/3	ML, lymphoplasmacytic
		9673/3	Mantle cell lymphoma
		9675/3	ML, mixed sm. and lg. cell, diffuse
ML, LARGE B-CELL, DIFFUSE	968	9680/3	ML, large B-cell, diffuse
		9684/3	ML, large B-cell, diffuse, immunoblastic, NOS
		9687/3	Burkitt lymphoma, NOS
		9688/3	T-cell histiocyte rich large B-cell lymphoma
FOLLIC. & MARGINAL LYMPH, NOS	969	9690/3	Follicular lymphoma, NOS
		9691/3	Follicular lymphoma, grade 2
		9695/3	Follicular lymphoma, grade 1
		9698/3	Follicular lymphoma, grade 3
		9699/3	Marginal zone B-cell lymphoma, NOS
T-CELL LYMPHOMAS	970	9701/3	Sezary syndrome
		9702/3	Mature T-cell lymphoma, NOS
		9705/3	Angioimmunoblastic T-cell lymphoma
OTHER SPEC. NON-HODGKIN LYMPHOMA	971	9712/3	Intravascular large B-cell lymphoma
		9714/3	Anaplastic large cell lymphoma, T-cell and Null cell type
		9719/3	NK/T-cell lymphoma, nasal and nasal-type

OTHER NERVOUS SYSTEM C728-C729

PRECURS. CELL LYMPHOBLASTIC LYMPH.	972	9724/3	SystemicEBV pos. T-cell lymphoproliferative disease of childhood
		9727/3	Precursor cell lymphoblastic lymphoma, NOS
		9728/3	Precursor B-cell lymphoblastic lymphoma
		9729/3	Precursor T-cell lymphoblastic lymphoma
PLASMA CELL TUMORS	973	9731/3	Plasmacytoma, NOS
		9734/3	Plasmacytoma, extramedullary
		9735/3	Plasmablastic lymphoma
		9737/3	ALK positive large B-cell lymphoma
		9738/3	Lrg B-cell lymphoma in HHV8-assoc. multicentric Castleman DZ
MAST CELL TUMORS	974	9740/3	Mast cell sarcoma
		9741/3	Malignant mastocytosis
NEOPLASMS OF HISTIOCYTES AND ACCESSORY LYMPHOID CELLS	975	9750/3	Malignant histiocytosis
		9751/3	Langerhans cell histiocytosis, NOS
		9754/3	Langerhans cell histiocytosis, disseminated
		9755/3	Histiocytic sarcoma
		9756/3	Langerhans cell sarcoma
		9757/3	Interdigitating dendritic cell sarcoma
		9758/3	Follicular dendritic cell sarcoma
		9759/3	Fibroblastic reticular cell tumor
PRECURSOR LYMPHOID NEOPLASMS	981	9811/3	B lymphoblastic leukemia/lymphoma, NOS
		9812/3	Leukemia/lymphoma with t(9;22)(q34;q11.2);BCR-ABL1
		9813/3	Leukemia/lymphoma with t(v;11q23);MLL rearranged
		9814/3	Leukemia/lymphoma with t(12;21)(p13;q22);TEL-AML1(ETV6-RUNX1)
		9815/3	B lymphoblastic leukemia/lymphoma with hyperdiploidy
		9816/3	Leukemia/lymphoma with hypodiploidy (hypodiploid ALL)
		9817/3	B lymphblastic leukemia/lymphoma with t(5;14)(q31;q32);IL3-IGH
		9818/3	Leukemia/lymphoma with t(1;19)(q23;p13.3); E2A PBX1 (TCF3 PBX1)
LYMPHOID LEUKEMIA, NOS	982	9823/3	Chronic lymphocytic leukemia/small lymphocytic lymphoma
		9827/3	Adult T-cell leukemia/lymphoma (HTLV-1 pos.)
PROLYMPH/PRECURS LEUKEMIA	983	9831/3	T-cell large granular lymphocytic leukemia
		9837/3	T lymphoblastic leukemia/lymphoma
MYELOID LEUKEMIA, NOS	986	9861/3	Acute myeloid leukemia
MYELOID SARCOMA	993	9930/3	Myeloid sarcoma
CHRONIC MYELOPROLIFERATIVE DIS.	996	9965/3	Myeloid and lymphoid neoplasms with PDGFRB rearrangement
		9967/3	Myeloid and lymphoid neoplasm with FGFR1 abnormalities

OTHER NERVOUS SYSTEM C728-C729

MYELOPLASTIC/MYELOPROLIFERATIVE NEOPLASMS	997	9971/3	Polymorphic PTLD
		9975/3	Myelodysplastic/Myeloproliferative neoplasm, unclassifiable

THYROID GLAND C739

NEOPLASM	800	8000/3	Neoplasm, malignant
		8001/3	Tumor cells, malignant
		8002/3	Malignant tumor, small cell type
		8003/3	Malignant tumor, giant cell type
		8004/3	Malignant tumor, spindle cell type
		8005/3	Malignant tumor, clear cell type
CARCINOMA, NOS	801	8010/2	Carcinoma in situ, NOS
		8010/3	Carcinoma, NOS
		8011/3	Epithelioma, malignant
		8012/3	Large cell carcinoma, NOS
		8013/3	Large cell neuroendocrine carcinoma
		8014/3	Large cell carcinoma with rhabdoid phenotype
		8015/3	Glassy cell carcinoma
CARCINOMA, UNDIFF., NOS	802	8020/3	Carcinoma, undifferentiated type, NOS
		8021/3	Carcinoma, anaplastic type, NOS
		8022/3	Pleomorphic carcinoma
GIANT & SPINDLE CELL CARCINOMA	803	8030/3	Giant cell and spindle cell carcinoma
		8031/3	Giant cell carcinoma
		8032/3	Spindle cell carcinoma
		8033/3	Pseudosarcomatous carcinoma
		8034/3	Polygonal cell carcinoma
		8035/3	Carcinoma with osteoclast-like giant cells
SMALL CELL CARCINOMA, NOS	804	8041/3	Small cell carcinoma, NOS
		8043/3	Small cell carcinoma, fusiform cell
PAPILLARY CARCINOMA, NOS	805	8050/2	Papillary carcinoma in situ
		8050/3	Papillary carcinoma, NOS
SQUAMOUS CELL CARCINOMA, NOS	807	8070/2	Squamous cell carcinoma in situ, NOS
		8070/3	Squamous cell carcinoma, NOS
		8071/3	Sq. cell carcinoma, keratinizing, NOS
		8072/3	Sq. cell carcinoma, lg. cell, non-ker.
		8073/3	Sq. cell carcinoma, sm. cell, non-ker.
		8074/3	Sq. cell carcinoma, spindle cell
		8075/3	Squamous cell carcinoma, adenoid
		8076/2	Sq. cell carc. in situ with question. stromal invas.
		8076/3	Sq. cell carcinoma, micro-invasive
		8078/3	Squamous cell carcinoma with horn formation

THYROID GLAND C739

ADENOCARCINOMA, NOS	814	8140/2	Adenocarcinoma in situ
		8140/3	Adenocarcinoma, NOS
		8141/3	Scirrhous adenocarcinoma
		8143/3	Superficial spreading adenocarcinoma
		8147/3	Basal cell adenocarcinoma
TRABECULAR ADENOCARCINOMA	819	8190/3	Trabecular adenocarcinoma
SOLID CARCINOMA, NOS	823	8230/2	Duct carcinoma in situ, solid type
		8230/3	Solid carcinoma, NOS
		8231/3	Carcinoma simplex
BRONCHIOLO-ALVEOLAR ADENOCA.	825	8251/3	Alveolar adenocarcinoma
		8255/3	Adenocarcinoma with mixed subtypes
PAPILLARY ADENOCARCINOMA, NOS	826	8260/3	Papillary adenocarcinoma, NOS
		8261/2	Adenocarcinoma in situ in villous adenoma
		8261/3	Adenocarcinoma in villous adenoma
		8262/3	Villous adenocarcinoma
		8263/2	Adenocarcinoma in situ in tubulovillous adenoma
		8263/3	Adenocarcinoma in tubulovillous adenoma
OXYPHILIC ADENOCARCINOMA	829	8290/3	Oxyphilic adenocarcinoma
CLEAR CELL ADENOCARCINOMA, NOS	831	8310/3	Clear cell adenocarcinoma, NOS
FOLLICULAR ADENOCARCINOMA, NOS	833	8330/3	Follicular adenocarcinoma, NOS
		8331/3	Follicular adenocarcinoma well diff.
		8332/3	Follicular adenocarcinoma trabecular
		8333/3	Fetal adenocarcinoma
		8335/3	Follicular carcinoma, minimally invasive
		8337/3	Insular carcinoma
		8339/3	Follicular thyroid carcinoma (FTC), encapsulated angioinvasive
PAPILLARY & FOLLICULAR ADENOCA.	834	8340/3	Papillary carcinoma, follicular variant
		8341/3	Papillary microcarcinoma
		8342/3	Papillary carcinoma, oxyphilic cell
		8343/2	Non-invasive EFVPTC
		8343/3	Papillary carcinoma, encapsulated
		8344/3	Papillary carcinoma, columnar cell
		8345/3	Medullary carcinoma with amyloid stroma
		8346/3	Mixed medullary-follicular carcinoma
		8347/3	Mixed medullary-papillary carcinoma
NONENCAPSUL. SCLEROSING CA.	835	8350/3	Nonencapsulated sclerosing carcinoma
PAPILLARY CYSTADENOCA., NOS	845	8450/3	Papillary cystadenocarcinoma, NOS

THYROID GLAND C739

MEDULLARY CARCINOMA, NOS	851	8510/3	Medullary carcinoma, NOS
ACINAR CELL CARCINOMA	855	8550/3	Acinar cell carcinoma
		8551/3	Acinar cell cystadenocarcinoma
FIBROUS HISTIOCYTOMA, MAL.	883	8830/3	Fibrous histiocytoma, malignant
CARCINOSARCOMA, NOS	898	8980/3	Carcinosarcoma, NOS
		8981/3	Carcinosarcoma, embryonal type
		8982/3	Malignant myoepithelioma
MALIGNANT LYMPHOMA, NOS	959	9590/3	Malignant lymphoma, NOS
		9591/3	Malignant lymphoma, non-Hodgkin
		9596/3	Composite Hodgkin and non-Hodgkin lymphoma
HODGKIN LYMPHOMA	965	9650/3	Hodgkin lymphoma, NOS
		9651/3	Hodgkin lymphoma, lymphocyte-rich
		9652/3	Hodgkin lymphoma, mixed cellularity, NOS
		9653/3	Hodgkin lymphoma, lymphocytic deplet., NOS
		9654/3	Hodgkin lymph., lymphocyt. deplet., diffuse fibrosis
		9655/3	Hodgkin lymphoma, lymphocyt. deplet., reticular
		9659/3	Hodgkin lymph., nodular lymphocyte predom.
HODGKIN LYMPHOMA, NOD. SCLER.	966	9661/3	Hodgkin granuloma [obs]
		9662/3	Hodgkin sarcoma [obs]
		9663/3	Hodgkin lymphoma, nodular sclerosis, NOS
		9664/3	Hodgkin lymphoma, nod. scler., cellular phase
		9665/3	Hodgkin lymphoma, nod. scler., grade 1
		9667/3	Hodgkin lymphoma, nod. scler., grade 2
ML, SMALL B-CELL LYMPHOCYTIC	967	9670/3	ML, small B lymphocytic, NOS
		9671/3	ML, lymphoplasmacytic
		9673/3	Mantle cell lymphoma
		9675/3	ML, mixed sm. and lg. cell, diffuse
ML, LARGE B-CELL, DIFFUSE	968	9680/3	ML, large B-cell, diffuse
		9684/3	ML, large B-cell, diffuse, immunoblastic, NOS
		9687/3	Burkitt lymphoma, NOS
		9688/3	T-cell histiocyte rich large B-cell lymphoma
FOLLIC. & MARGINAL LYMPH, NOS	969	9690/3	Follicular lymphoma, NOS
		9691/3	Follicular lymphoma, grade 2
		9695/3	Follicular lymphoma, grade 1
		9698/3	Follicular lymphoma, grade 3
		9699/3	Marginal zone B-cell lymphoma, NOS

THYROID GLAND C739

T-CELL LYMPHOMAS	970	9701/3	Sezary syndrome
		9702/3	Mature T-cell lymphoma, NOS
		9705/3	Angioimmunoblastic T-cell lymphoma
OTHER SPEC. NON-HODGKIN LYMPHOMA	971	9712/3	Intravascular large B-cell lymphoma
		9714/3	Anaplastic large cell lymphoma, T-cell and Null cell type
		9719/3	NK/T-cell lymphoma, nasal and nasal-type
PRECURS. CELL LYMPHOBLASTIC LYMPH.	972	9724/3	SystemicEBV pos. T-cell lymphoproliferative disease of childhood
		9727/3	Precursor cell lymphoblastic lymphoma, NOS
		9728/3	Precursor B-cell lymphoblastic lymphoma
		9729/3	Precursor T-cell lymphoblastic lymphoma
PLASMA CELL TUMORS	973	9731/3	Plasmacytoma, NOS
		9734/3	Plasmacytoma, extramedullary
		9735/3	Plasmablastic lymphoma
		9737/3	ALK positive large B-cell lymphoma
		9738/3	Lrg B-cell lymphoma in HHV8-assoc. multicentric Castleman DZ
MAST CELL TUMORS	974	9740/3	Mast cell sarcoma
		9741/3	Malignant mastocytosis
NEOPLASMS OF HISTIOCYTES AND ACCESSORY LYMPHOID CELLS	975	9750/3	Malignant histiocytosis
		9751/3	Langerhans cell histiocytosis, NOS
		9754/3	Langerhans cell histiocytosis, disseminated
		9755/3	Histiocytic sarcoma
		9756/3	Langerhans cell sarcoma
		9757/3	Interdigitating dendritic cell sarcoma
		9758/3	Follicular dendritic cell sarcoma
		9759/3	Fibroblastic reticular cell tumor
PRECURSOR LYMPHOID NEOPLASMS	981	9811/3	B lymphoblastic leukemia/lymphoma, NOS
		9812/3	Leukemia/lymphoma with t(9;22)(q34;q11.2);BCR-ABL1
		9813/3	Leukemia/lymphoma with t(v;11q23);MLL rearranged
		9814/3	Leukemia/lymphoma with t(12;21)(p13;q22);TEL-AML1(ETV6-RUNX1)
		9815/3	B lymphoblastic leukemia/lymphoma with hyperdiploidy
		9816/3	Leukemia/lymphoma with hypodiploidy (hypodiploid ALL)
		9817/3	B lymphblastic leukemia/lymphoma with t(5;14)(q31;q32);IL3-IGH
		9818/3	Leukemia/lymphoma with t(1;19)(q23;p13.3); E2A PBX1 (TCF3 PBX1)
LYMPHOID LEUKEMIA, NOS	982	9823/3	Chronic lymphocytic leukemia/small lymphocytic lymphoma
PROLYMPH/PRECURS LEUKEMIA	983	9831/3	T-cell large granular lymphocytic leukemia
		9837/3	T lymphoblastic leukemia/lymphoma

THYROID GLAND C739

CHRONIC MYELOPROLIFERATIVE DIS.	996	9965/3	Myeloid and lymphoid neoplasms with PDGFRB rearrangement
		9967/3	Myeloid and lymphoid neoplasm with FGFR1 abnormalities
MYELOPLASTIC/MYELOPROLIFERATIVE NEOPLASMS	997	9971/3	Polymorphic PTLD
		9975/3	Myelodysplastic/Myeloproliferative neoplasm, unclassifiable

ADRENAL GLANDS C740-C741,C749

NEOPLASM	800	8000/3	Neoplasm, malignant
		8001/3	Tumor cells, malignant
		8002/3	Malignant tumor, small cell type
		8003/3	Malignant tumor, giant cell type
		8004/3	Malignant tumor, spindle cell type
		8005/3	Malignant tumor, clear cell type
CARCINOMA, NOS	801	8010/2	Carcinoma in situ, NOS
		8010/3	Carcinoma, NOS
		8011/3	Epithelioma, malignant
		8012/3	Large cell carcinoma, NOS
		8013/3	Large cell neuroendocrine carcinoma
		8014/3	Large cell carcinoma with rhabdoid phenotype
		8015/3	Glassy cell carcinoma
CARCINOMA, UNDIFF., NOS	802	8020/3	Carcinoma, undifferentiated type, NOS
		8021/3	Carcinoma, anaplastic type, NOS
		8022/3	Pleomorphic carcinoma
ADENOCARCINOMA, NOS	814	8140/2	Adenocarcinoma in situ
		8140/3	Adenocarcinoma, NOS
		8141/3	Scirrhous adenocarcinoma
		8143/3	Superficial spreading adenocarcinoma
		8147/3	Basal cell adenocarcinoma
BRONCHIOLO-ALVEOLAR ADENOCA.	825	8255/3	Adenocarcinoma with mixed subtypes
OXYPHILIC ADENOCARCINOMA	829	8290/3	Oxyphilic adenocarcinoma
CLEAR CELL ADENOCARCINOMA, NOS	831	8310/3	Clear cell adenocarcinoma, NOS
GRANULAR CELL CARCINOMA	832	8320/3	Granular cell carcinoma
		8323/3	Mixed cell adenocarcinoma
ADRENAL CORTICAL CARCINOMA	837	8370/3	Adrenal cortical carcinoma
PARAGANGLIOMA	868	8680/3	Paraganglioma, malignant
EXTRA-ADRENAL PARAGANG., MAL	869	8693/3	Extra-adrenal paraganglioma, malignant
PHEOCHROMOCYTOMA	870	8700/3	Pheochromocytoma
GERM CELL TUMORS	906	9060/3	Dysgerminoma
		9064/3	Germinoma
		9065/3	Germ cell tumor, nonseminomatous

ADRENAL GLANDS C740-C741,C749

EMBRYONAL CARCINOMA, NOS	907	9070/3	Embryonal carcinoma, NOS
		9071/3	Yolk sac tumor
		9072/3	Polyembryoma
TERATOMA	908	9080/3	Teratoma, malignant, NOS
		9081/3	Teratocarcinoma
		9082/3	Malignant teratoma, undiff.
		9083/3	Malignant teratoma, intermediate
		9084/3	Teratoma with malig. transformation
		9085/3	Mixed germ cell tumor
CHORDOMA	937	9370/3	Chordoma, NOS
		9371/3	Chondroid chordoma
		9372/3	Dedifferentiated chordoma
GANGLIONEUROBLASTOMA	949	9490/3	Ganglioneuroblastoma
NEUROBLASTOMA, NOS	950	9500/3	Neuroblastoma, NOS
		9501/3	Medulloepithelioma, NOS
		9502/3	Teratoid medulloepithelioma
		9503/3	Neuroepithelioma, NOS
		9504/3	Spongioneuroblastoma
		9505/3	Ganglioglioma, anaplastic
FOLLIC. & MARGINAL LYMPH, NOS	969	9699/3	Marginal zone B-cell lymphoma, NOS
LYMPHOID LEUKEMIA, NOS	982	9823/3	Chronic lymphocytic leukemia/small lymphocytic lymphoma

PARATHYROIDGLAND C750

NEOPLASM	800	8000/3	Neoplasm, malignant
		8001/3	Tumor cells, malignant
		8002/3	Malignant tumor, small cell type
		8003/3	Malignant tumor, giant cell type
		8004/3	Malignant tumor, spindle cell type
		8005/3	Malignant tumor, clear cell type
CARCINOMA, NOS	801	8010/2	Carcinoma in situ, NOS
		8010/3	Carcinoma, NOS
		8011/3	Epithelioma, malignant
		8012/3	Large cell carcinoma, NOS
		8013/3	Large cell neuroendocrine carcinoma
		8014/3	Large cell carcinoma with rhabdoid phenotype
		8015/3	Glassy cell carcinoma
CARCINOMA, UNDIFF., NOS	802	8020/3	Carcinoma, undifferentiated type, NOS
		8021/3	Carcinoma, anaplastic type, NOS
		8022/3	Pleomorphic carcinoma
ADENOCARCINOMA, NOS	814	8140/2	Adenocarcinoma in situ
		8140/3	Adenocarcinoma, NOS
		8141/3	Scirrhous adenocarcinoma
		8143/3	Superficial spreading adenocarcinoma
		8147/3	Basal cell adenocarcinoma
BRONCHIOLO-ALVEOLAR ADENOCA.	825	8255/3	Adenocarcinoma with mixed subtypes
OXYPHILIC ADENOCARCINOMA	829	8290/3	Oxyphilic adenocarcinoma
CLEAR CELL ADENOCARCINOMA, NOS	831	8310/3	Clear cell adenocarcinoma, NOS
GRANULAR CELL CARCINOMA	832	8320/3	Granular cell carcinoma
		8322/3	Water-clear cell adenocarcinoma
		8323/3	Mixed cell adenocarcinoma
PARAGANGLIOMA	868	8680/3	Paraganglioma, malignant
EXTRA-ADRENAL PARAGANG., MAL	869	8693/3	Extra-adrenal paraganglioma, malignant
GERM CELL TUMORS	906	9060/3	Dysgerminoma
		9064/3	Germinoma
		9065/3	Germ cell tumor, nonseminomatous

PARATHYROIDGLAND C750

EMBRYONAL CARCINOMA, NOS	907	9070/3	Embryonal carcinoma, NOS
		9071/3	Yolk sac tumor
		9072/3	Polyembryoma
TERATOMA	908	9080/3	Teratoma, malignant, NOS
		9081/3	Teratocarcinoma
		9082/3	Malignant teratoma, undiff.
		9083/3	Malignant teratoma, intermediate
		9084/3	Teratoma with malig. transformation
		9085/3	Mixed germ cell tumor
CHORDOMA	937	9370/3	Chordoma, NOS
		9371/3	Chondroid chordoma
		9372/3	Dedifferentiated chordoma
GANGLIONEUROBLASTOMA	949	9490/3	Ganglioneuroblastoma
NEUROBLASTOMA, NOS	950	9500/3	Neuroblastoma, NOS
		9501/3	Medulloepithelioma, NOS
		9502/3	Teratoid medulloepithelioma
		9503/3	Neuroepithelioma, NOS
		9504/3	Spongioneuroblastoma
		9505/3	Ganglioglioma, anaplastic
FOLLIC. & MARGINAL LYMPH, NOS	969	9699/3	Marginal zone B-cell lymphoma, NOS
LYMPHOID LEUKEMIA, NOS	982	9823/3	Chronic lymphocytic leukemia/small lymphocytic lymphoma

PITUITARYGLAND C751

NEOPLASM	800	8000/0	Neoplasm, benign
		8000/1	Neoplasm, uncertain whether benign or malignant
		8000/3	Neoplasm, malignant
		8001/0	Tumor cells, benign
		8001/1	Tumor cells, uncertain whether benign or malignant
		8001/3	Tumor cells, malignant
		8005/0	Clear cell tumor, NOS
		8005/3	Malignant tumor, clear cell type
CARCINOMA, NOS	801	8010/0	Epithelial tumor, benign
		8010/2	Carcinoma in situ, NOS
		8010/3	Carcinoma, NOS
ADENOCARCINOMA, NOS	814	8140/0	Adenoma, NOS
		8140/2	Adenocarcinoma in situ
		8140/3	Adenocarcinoma, NOS
		8146/0	Monomorphic adenoma
PAPILLARY ADENOCARCINOMA, NOS	826	8260/0	Papillary adenoma, NOS
CHROMOPHOBE CARCINOMA	827	8270/0	Chromophobe adenoma
		8270/3	Chromophobe carcinoma
		8271/0	Prolactinoma
		8272/0	Pituitary adenoma, NOS
		8272/3	Pituitary carcinoma, NOS
ACIDOPHIL CARCINOMA	828	8280/0	Acidophil adenoma
		8280/3	Acidophil carcinoma
		8281/0	Mixed acidophil-basophil adenoma
		8281/3	Mixed acidophil-basophil carcinoma
OXYPHILIC ADENOCARCINOMA	829	8290/0	Oxyphilic adenoma
		8290/3	Oxyphilic adenocarcinoma
BASOPHIL CARCINOMA	830	8300/0	Basophil adenoma
		8300/3	Basophil carcinoma
CLEAR CELL ADENOCARCINOMA, NOS	831	8310/0	Clear cell adenoma
GRANULAR CELL CARCINOMA	832	8320/3	Granular cell carcinoma
		8323/0	Mixed cell adenoma
		8323/3	Mixed cell adenocarcinoma

PITUITARYGLAND C751

SARCOMA, NOS	880	8800/0	Soft tissue tumor, benign
		8800/3	Sarcoma, NOS
LIPOSARCOMA NEOPLASMS	885	8850/0	Lipoma, NOS
GERM CELL TUMORS	906	9060/3	Dysgerminoma
		9064/3	Germinoma
		9065/3	Germ cell tumor, nonseminomatous
EMBRYONAL CARCINOMA, NOS	907	9070/3	Embryonal carcinoma, NOS
		9071/3	Yolk sac tumor
		9072/3	Polyembryoma
TERATOMA	908	9080/0	Teratoma, benign
		9080/1	Teratoma, NOS
		9080/3	Teratoma, malignant, NOS
		9081/3	Teratocarcinoma
		9082/3	Malignant teratoma, undiff.
		9083/3	Malignant teratoma, intermediate
		9084/3	Teratoma with malig. transformation
		9085/3	Mixed germ cell tumor
CRANIOPHARYNGIOMA	935	9350/1	Craniopharyngioma
		9351/1	Adamantinomatous craniopharyngioma
		9352/1	Papillary craniopharyngioma
CHORDOMA	937	9370/3	Chordoma, NOS
		9371/3	Chondroid chordoma
		9372/3	Dedifferentiated chordoma
ASTROBLASTOMA	943	9432/1	Pituicytoma
NEUROBLASTOMA, NOS	950	9500/3	Neuroblastoma, NOS
		9501/3	Medulloepithelioma, NOS
		9502/3	Teratoid medulloepithelioma
		9503/3	Neuroepithelioma, NOS
		9505/3	Ganglioglioma, anaplastic
GRANULAR CELL TUMOR	958	9580/0	Granular cell tumor, NOS
		9582/0	Granular cell tumor of the sellar region
FOLLIC. & MARGINAL LYMPH, NOS	969	9699/3	Marginal zone B-cell lymphoma, NOS
LYMPHOID LEUKEMIA, NOS	982	9823/3	Chronic lymphocytic leukemia/small lymphocytic lymphoma

CRANIOPHARYNGEALDUCT C752

NEOPLASM	800	8000/0	Neoplasm, benign
		8000/1	Neoplasm, uncertain whether benign or malignant
		8000/3	Neoplasm, malignant
		8001/0	Tumor cells, benign
		8001/1	Tumor cells, uncertain whether benign or malignant
		8001/3	Tumor cells, malignant
		8005/0	Clear cell tumor, NOS
		8005/3	Malignant tumor, clear cell type
CARCINOMA, NOS	801	8010/0	Epithelial tumor, benign
		8010/2	Carcinoma in situ, NOS
		8010/3	Carcinoma, NOS
ADENOCARCINOMA, NOS	814	8140/0	Adenoma, NOS
		8140/2	Adenocarcinoma in situ
		8140/3	Adenocarcinoma, NOS
		8146/0	Monomorphic adenoma
PAPILLARY ADENOCARCINOMA, NOS	826	8260/0	Papillary adenoma, NOS
CHROMOPHOBE CARCINOMA	827	8270/0	Chromophobe adenoma
		8270/3	Chromophobe carcinoma
		8271/0	Prolactinoma
		8272/0	Pituitary adenoma, NOS
		8272/3	Pituitary carcinoma, NOS
ACIDOPHIL CARCINOMA	828	8280/0	Acidophil adenoma
		8280/3	Acidophil carcinoma
		8281/0	Mixed acidophil-basophil adenoma
		8281/3	Mixed acidophil-basophil carcinoma
OXYPHILIC ADENOCARCINOMA	829	8290/0	Oxyphilic adenoma
		8290/3	Oxyphilic adenocarcinoma
BASOPHIL CARCINOMA	830	8300/0	Basophil adenoma
		8300/3	Basophil carcinoma
CLEAR CELL ADENOCARCINOMA, NOS	831	8310/0	Clear cell adenoma
GRANULAR CELL CARCINOMA	832	8320/3	Granular cell carcinoma
		8323/0	Mixed cell adenoma
		8323/3	Mixed cell adenocarcinoma

CRANIOPHARYNGEALDUCT C752

SARCOMA, NOS	880	8800/0	Soft tissue tumor, benign
		8800/3	Sarcoma, NOS
LIPOSARCOMA NEOPLASMS	885	8850/0	Lipoma, NOS
GERM CELL TUMORS	906	9060/3	Dysgerminoma
		9064/3	Germinoma
		9065/3	Germ cell tumor, nonseminomatous
EMBRYONAL CARCINOMA, NOS	907	9070/3	Embryonal carcinoma, NOS
		9071/3	Yolk sac tumor
		9072/3	Polyembryoma
TERATOMA	908	9080/0	Teratoma, benign
		9080/1	Teratoma, NOS
		9080/3	Teratoma, malignant, NOS
		9081/3	Teratocarcinoma
		9082/3	Malignant teratoma, undiff.
		9083/3	Malignant teratoma, intermediate
		9084/3	Teratoma with malig. transformation
		9085/3	Mixed germ cell tumor
CRANIOPHARYNGIOMA	935	9350/1	Craniopharyngioma
		9351/1	Adamantinomatous craniopharyngioma
		9352/1	Papillary craniopharyngioma
CHORDOMA	937	9370/3	Chordoma, NOS
		9371/3	Chondroid chordoma
		9372/3	Dedifferentiated chordoma
NEUROBLASTOMA, NOS	950	9500/3	Neuroblastoma, NOS
		9501/3	Medulloepithelioma, NOS
		9502/3	Teratoid medulloepithelioma
		9503/3	Neuroepithelioma, NOS
		9505/3	Ganglioglioma, anaplastic
GRANULAR CELL TUMOR	958	9580/0	Granular cell tumor, NOS
FOLLIC. & MARGINAL LYMPH, NOS	969	9699/3	Marginal zone B-cell lymphoma, NOS
LYMPHOID LEUKEMIA, NOS	982	9823/3	Chronic lymphocytic leukemia/small lymphocytic lymphoma

PINEAL GLAND C753

NEOPLASM	800	8000/0	Neoplasm, benign
		8000/1	Neoplasm, uncertain whether benign or malignant
		8000/3	Neoplasm, malignant
		8001/0	Tumor cells, benign
		8001/1	Tumor cells, uncertain whether benign or malignant
		8001/3	Tumor cells, malignant
CARCINOMA, NOS	801	8010/0	Epithelial tumor, benign
GERM CELL TUMORS	906	9060/3	Dysgerminoma
		9064/3	Germinoma
		9065/3	Germ cell tumor, nonseminomatous
EMBRYONAL CARCINOMA, NOS	907	9070/3	Embryonal carcinoma, NOS
		9071/3	Yolk sac tumor
		9072/3	Polyembryoma
TERATOMA	908	9080/0	Teratoma, benign
		9080/3	Teratoma, malignant, NOS
		9081/3	Teratocarcinoma
		9082/3	Malignant teratoma, undiff.
		9083/3	Malignant teratoma, intermediate
		9084/0	Dermoid cyst, NOS
		9084/3	Teratoma with malig. transformation
		9085/3	Mixed germ cell tumor
PERIPHERAL NEUROECTODERMAL TUMOR	936	9360/1	Pinealoma, NOS
		9361/1	Pineocytoma
		9362/3	Pineoblastoma
CHORDOMA	937	9370/3	Chordoma, NOS
		9371/3	Chondroid chordoma
		9372/3	Dedifferentiated chordoma
EPENDYMOMA, NOS	939	9395/3	Papillary tumor of pineal region
MEDULLOBLASTOMA, NOS	947	9473/3	Primitive neuroectodermal tumor
GANGLIONEUROBLASTOMA	949	9490/3	Ganglioneuroblastoma
		9492/0	Gangliocytoma
NEUROBLASTOMA, NOS	950	9500/3	Neuroblastoma, NOS
		9501/3	Medulloepithelioma, NOS
		9502/3	Teratoid medulloepithelioma
		9503/3	Neuroepithelioma, NOS
		9505/1	Ganglioglioma, NOS
		9505/3	Ganglioglioma, anaplastic

PINEAL GLAND C753

ML, LARGE B-CELL, DIFFUSE	968	9680/3	ML, large B-cell, diffuse
FOLLIC. & MARGINAL LYMPH, NOS	969	9699/3	Marginal zone B-cell lymphoma, NOS
LYMPHOID LEUKEMIA, NOS	982	9823/3	Chronic lymphocytic leukemia/small lymphocytic lymphoma

OTHER ENDOCRINE GLANDS C754-C755,C758-C759

NEOPLASM	800	8000/3	Neoplasm, malignant
		8001/3	Tumor cells, malignant
		8002/3	Malignant tumor, small cell type
		8003/3	Malignant tumor, giant cell type
		8004/3	Malignant tumor, spindle cell type
		8005/3	Malignant tumor, clear cell type
CARCINOMA, NOS	801	8010/2	Carcinoma in situ, NOS
		8010/3	Carcinoma, NOS
		8011/3	Epithelioma, malignant
		8012/3	Large cell carcinoma, NOS
		8013/3	Large cell neuroendocrine carcinoma
		8014/3	Large cell carcinoma with rhabdoid phenotype
		8015/3	Glassy cell carcinoma
CARCINOMA, UNDIFF., NOS	802	8020/3	Carcinoma, undifferentiated type, NOS
		8021/3	Carcinoma, anaplastic type, NOS
		8022/3	Pleomorphic carcinoma
ADENOCARCINOMA, NOS	814	8140/2	Adenocarcinoma in situ
		8140/3	Adenocarcinoma, NOS
		8141/3	Scirrhous adenocarcinoma
		8143/3	Superficial spreading adenocarcinoma
		8147/3	Basal cell adenocarcinoma
BRONCHIOLO-ALVEOLAR ADENOCA.	825	8255/3	Adenocarcinoma with mixed subtypes
OXYPHILIC ADENOCARCINOMA	829	8290/3	Oxyphilic adenocarcinoma
CLEAR CELL ADENOCARCINOMA, NOS	831	8310/3	Clear cell adenocarcinoma, NOS
GRANULAR CELL CARCINOMA	832	8320/3	Granular cell carcinoma
		8323/3	Mixed cell adenocarcinoma
PARAGANGLIOMA	868	8680/3	Paraganglioma, malignant
EXTRA-ADRENAL PARAGANG., MAL	869	8691/3	Aortic body tumor, malignant
		8692/3	Carotid body tumor, malignant
		8693/3	Extra-adrenal paraganglioma, malignant
GERM CELL TUMORS	906	9060/3	Dysgerminoma
		9064/3	Germinoma
		9065/3	Germ cell tumor, nonseminomatous

OTHER ENDOCRINE GLANDS C754-C755,C758-C759

EMBRYONAL CARCINOMA, NOS	907	9070/3	Embryonal carcinoma, NOS
		9071/3	Yolk sac tumor
		9072/3	Polyembryoma
TERATOMA	908	9080/3	Teratoma, malignant, NOS
		9081/3	Teratocarcinoma
		9082/3	Malignant teratoma, undiff.
		9083/3	Malignant teratoma, intermediate
		9084/3	Teratoma with malig. transformation
		9085/3	Mixed germ cell tumor
CHORDOMA	937	9370/3	Chordoma, NOS
		9371/3	Chondroid chordoma
		9372/3	Dedifferentiated chordoma
GANGLIONEUROBLASTOMA	949	9490/3	Ganglioneuroblastoma
NEUROBLASTOMA, NOS	950	9500/3	Neuroblastoma, NOS
		9501/3	Medulloepithelioma, NOS
		9502/3	Teratoid medulloepithelioma
		9503/3	Neuroepithelioma, NOS
		9504/3	Spongioneuroblastoma
		9505/3	Ganglioglioma, anaplastic
FOLLIC. & MARGINAL LYMPH, NOS	969	9699/3	Marginal zone B-cell lymphoma, NOS
LYMPHOID LEUKEMIA, NOS	982	9823/3	Chronic lymphocytic leukemia/small lymphocytic lymphoma

ILL-DEFINED C760-C768

NEOPLASM	800	8000/3	Neoplasm, malignant
		8001/3	Tumor cells, malignant
		8002/3	Malignant tumor, small cell type
		8003/3	Malignant tumor, giant cell type
		8004/3	Malignant tumor, spindle cell type
		8005/3	Malignant tumor, clear cell type
CARCINOMA, UNDIFF., NOS	802	8020/3	Carcinoma, undifferentiated type, NOS
		8021/3	Carcinoma, anaplastic type, NOS
		8022/3	Pleomorphic carcinoma
ADENOCARCINOMA, NOS	814	8140/2	Adenocarcinoma in situ
		8140/3	Adenocarcinoma, NOS
		8141/3	Scirrhous adenocarcinoma
		8143/3	Superficial spreading adenocarcinoma
		8147/3	Basal cell adenocarcinoma
BRONCHIOLO-ALVEOLAR ADENOCA.	825	8255/3	Adenocarcinoma with mixed subtypes
MUCINOUS ADENOCARCINOMA	848	8480/3	Mucinous adenocarcinoma
		8481/3	Mucin-producing adenocarcinoma
NEUROBLASTOMA, NOS	950	9500/3	Neuroblastoma, NOS
FOLLIC. & MARGINAL LYMPH, NOS	969	9699/3	Marginal zone B-cell lymphoma, NOS
LYMPHOID LEUKEMIA, NOS	982	9823/3	Chronic lymphocytic leukemia/small lymphocytic lymphoma

LYMPH NODES C770-C775,C778-C779

NEOPLASM	800	8000/3	Neoplasm, malignant
		8001/3	Tumor cells, malignant
		8002/3	Malignant tumor, small cell type
		8003/3	Malignant tumor, giant cell type
		8004/3	Malignant tumor, spindle cell type
		8005/3	Malignant tumor, clear cell type
MALIGNANT LYMPHOMA, NOS	959	9590/3	Malignant lymphoma, NOS
		9591/3	Malignant lymphoma, non-Hodgkin
		9596/3	Composite Hodgkin and non-Hodgkin lymphoma
HODGKIN LYMPHOMA	965	9650/3	Hodgkin lymphoma, NOS
		9651/3	Hodgkin lymphoma, lymphocyte-rich
		9652/3	Hodgkin lymphoma, mixed cellularity, NOS
		9653/3	Hodgkin lymphoma, lymphocytic deplet., NOS
		9654/3	Hodgkin lymph., lymphocyt. deplet., diffuse fibrosis
		9655/3	Hodgkin lymphoma, lymphocyt. deplet., reticular
		9659/3	Hodgkin lymph., nodular lymphocyte predom.
HODGKIN LYMPHOMA, NOD. SCLER.	966	9661/3	Hodgkin granuloma [obs]
		9662/3	Hodgkin sarcoma [obs]
		9663/3	Hodgkin lymphoma, nodular sclerosis, NOS
		9664/3	Hodgkin lymphoma, nod. scler., cellular phase
		9665/3	Hodgkin lymphoma, nod. scler., grade 1
		9667/3	Hodgkin lymphoma, nod. scler., grade 2
ML, SMALL B-CELL LYMPHOCYTIC	967	9670/3	ML, small B lymphocytic, NOS
		9671/3	ML, lymphoplasmacytic
		9673/3	Mantle cell lymphoma
		9675/3	ML, mixed sm. and lg. cell, diffuse
		9678/3	Primary effusion lymphoma
		9679/3	Mediastinal large B-cell lymphoma
ML, LARGE B-CELL, DIFFUSE	968	9680/3	ML, large B-cell, diffuse
		9684/3	ML, large B-cell, diffuse, immunoblastic, NOS
		9687/3	Burkitt lymphoma, NOS
		9688/3	T-cell histiocyte rich large B-cell lymphoma
		9689/3	Splenic marginal zone B-cell lymphoma
FOLLIC. & MARGINAL LYMPH, NOS	969	9690/3	Follicular lymphoma, NOS
		9691/3	Follicular lymphoma, grade 2
		9695/3	Follicular lymphoma, grade 1
		9698/3	Follicular lymphoma, grade 3
		9699/3	Marginal zone B-cell lymphoma, NOS

LYMPH NODES C770-C775,C778-C779

T-CELL LYMPHOMAS	970	9702/3	Mature T-cell lymphoma, NOS
		9705/3	Angioimmunoblastic T-cell lymphoma
		9708/3	Subcutaneous panniculitis-like T-cell lymphoma
OTHER SPEC. NON-HODGKIN LYMPHOMA	971	9712/3	Intravascular large B-cell lymphoma
		9714/3	Anaplastic large cell lymphoma, T-cell and Null cell type
		9716/3	Hepatosplenic gamma-delta cell lymphoma
		9717/3	Intestinal T-cell lymphoma
		9719/3	NK/T-cell lymphoma, nasal and nasal-type
PRECURS. CELL LYMPHOBLASTIC LYMPH.	972	9724/3	SystemicEBV pos. T-cell lymphoproliferative disease of childhood
		9727/3	Precursor cell lymphoblastic lymphoma, NOS
		9728/3	Precursor B-cell lymphoblastic lymphoma
		9729/3	Precursor T-cell lymphoblastic lymphoma
PLASMA CELL TUMORS	973	9735/3	Plasmablastic lymphoma
		9737/3	ALK positive large B-cell lymphoma
		9738/3	Lrg B-cell lymphoma in HHV8-assoc. multicentric Castleman DZ
NEOPLASMS OF HISTIOCYTES AND ACCESSORY LYMPHOID CELLS	975	9751/3	Langerhans cell histiocytosis, NOS
		9755/3	Histiocytic sarcoma
		9759/3	Fibroblastic reticular cell tumor
IMMUNOPROLIFERATIVE DISEASES	976	9761/3	Waldenstrom macroglobulinemia
		9762/3	Heavy chain disease, NOS
PRECURSOR LYMPHOID NEOPLASMS	981	9811/3	B lymphoblastic leukemia/lymphoma, NOS
		9812/3	Leukemia/lymphoma with t(9;22)(q34;q11.2);BCR-ABL1
		9813/3	Leukemia/lymphoma with t(v;11q23);MLL rearranged
		9814/3	Leukemia/lymphoma with t(12;21)(p13;q22);TEL-AML1(ETV6-RUNX1)
		9815/3	B lymphoblastic leukemia/lymphoma with hyperdiploidy
		9816/3	Leukemia/lymphoma with hypodiploidy (hypodiploid ALL)
		9817/3	B lymphblastic leukemia/lymphoma with t(5;14)(q31;q32);IL3-IGH
		9818/3	Leukemia/lymphoma with t(1;19)(q23;p13.3); E2A PBX1 (TCF3 PBX1)
LYMPHOID LEUKEMIA, NOS	982	9823/3	Chronic lymphocytic leukemia/small lymphocytic lymphoma
PROLYMPH/PRECURS LEUKEMIA	983	9831/3	T-cell large granular lymphocytic leukemia
		9837/3	T lymphoblastic leukemia/lymphoma
CHRONIC MYELOPROLIFERATIVE DIS.	996	9965/3	Myeloid and lymphoid neoplasms with PDGFRB rearrangement
		9967/3	Myeloid and lymphoid neoplasm with FGFR1 abnormalities
MYELOPLASTIC/MYELOPROLIFERATIVE NEOPLASMS	997	9971/3	Polymorphic PTLD
		9975/3	Myelodysplastic/Myeloproliferative neoplasm, unclassifiable

UNKNOWN C809

NEOPLASM	800	8000/3	Neoplasm, malignant
		8001/3	Tumor cells, malignant
		8002/3	Malignant tumor, small cell type
		8003/3	Malignant tumor, giant cell type
		8004/3	Malignant tumor, spindle cell type
		8005/3	Malignant tumor, clear cell type
CARCINOMA, NOS	801	8010/2	Carcinoma in situ, NOS
		8010/3	Carcinoma, NOS
		8011/3	Epithelioma, malignant
		8012/3	Large cell carcinoma, NOS
		8013/3	Large cell neuroendocrine carcinoma
		8014/3	Large cell carcinoma with rhabdoid phenotype
		8015/3	Glassy cell carcinoma
CARCINOMA, UNDIFF., NOS	802	8020/3	Carcinoma, undifferentiated type, NOS
		8021/3	Carcinoma, anaplastic type, NOS
		8022/3	Pleomorphic carcinoma
GIANT & SPINDLE CELL CARCINOMA	803	8030/3	Giant cell and spindle cell carcinoma
		8031/3	Giant cell carcinoma
		8032/3	Spindle cell carcinoma
		8033/3	Pseudosarcomatous carcinoma
		8034/3	Polygonal cell carcinoma
		8035/3	Carcinoma with osteoclast-like giant cells
SMALL CELL CARCINOMA, NOS	804	8041/3	Small cell carcinoma, NOS
		8043/3	Small cell carcinoma, fusiform cell
PAPILLARY CARCINOMA, NOS	805	8050/2	Papillary carcinoma in situ
		8050/3	Papillary carcinoma, NOS
		8051/3	Verrucous carcinoma, NOS
		8052/2	Papillary squamous cell carcinoma, non-invasive
		8052/3	Papillary squamous cell carcinoma
SQUAMOUS CELL CARCINOMA, NOS	807	8070/2	Squamous cell carcinoma in situ, NOS
		8070/3	Squamous cell carcinoma, NOS
		8071/3	Sq. cell carcinoma, keratinizing, NOS
		8072/3	Sq. cell carcinoma, lg. cell, non-ker.
		8073/3	Sq. cell carcinoma, sm. cell, non-ker.
		8074/3	Sq. cell carcinoma, spindle cell
		8075/3	Squamous cell carcinoma, adenoid
		8076/2	Sq. cell carc. in situ with question. stromal invas.
		8076/3	Sq. cell carcinoma, micro-invasive
		8078/3	Squamous cell carcinoma with horn formation

UNKNOWN C809

LYMPHOEPITHELIAL CARCINOMA	808	8082/3	Lymphoepithelial carcinoma
		8083/3	Basaloid squamous cell carcinoma
		8084/3	Squamous cell carcinoma, clear cell type
TRANSITIONAL CELL CARCINOMA, NOS	812	8120/2	Transitional cell carcinoma in situ
		8120/3	Transitional cell carcinoma, NOS
		8121/3	Schneiderian carcinoma
		8122/3	Trans. cell carcinoma, spindle cell
		8123/3	Basaloid carcinoma
		8124/3	Cloacogenic carcinoma
PAPILLARY TRANS. CELL CARCINOMA	813	8130/2	Papillary trans. cell carcinoma, non-invasive
		8130/3	Papillary trans. cell carcinoma
		8131/3	Transitional cell carcinoma, micropapillary
ADENOCARCINOMA, NOS	814	8140/2	Adenocarcinoma in situ
		8140/3	Adenocarcinoma, NOS
		8141/3	Scirrhous adenocarcinoma
		8143/3	Superficial spreading adenocarcinoma
		8147/3	Basal cell adenocarcinoma
ENDOCRINOMAS	815	8152/3	Glucagonoma, malignant
		8153/3	Gastrinoma, malignant
		8156/3	Somatostatinoma, malignant
		8157/3	Enteroglucagonoma, malignant
TRABECULAR ADENOCARCINOMA	819	8190/3	Trabecular adenocarcinoma
ADENOID CYSTIC & CRIBRIFORM CA.	820	8200/3	Adenoid cystic carcinoma
		8201/2	Cribriform carcinoma in situ
		8201/3	Cribriform carcinoma
ADENOCA. IN ADENOMA. POLYP	821	8210/2	Adenocarcinoma in situ in adenomatous polyp
		8210/3	Adenocarcinoma in adenomatous polyp
		8211/3	Tubular adenocarcinoma
SOLID CARCINOMA, NOS	823	8230/2	Duct carcinoma in situ, solid type
		8230/3	Solid carcinoma, NOS
		8231/3	Carcinoma simplex
CARCINOID TUMOR, MALIGNANT	824	8240/3	Carcinoid tumor, malignant
		8241/3	Enterochromaffin cell carcinoid
		8242/3	Enterochromaffin-like cell tumor, malignant
		8243/3	Goblet cell carcinoid
		8244/3	Composite carcinoid
		8245/3	Adenocarcinoid tumor
		8246/3	Neuroendocrine carcinoma

UNKNOWN C809

CARCINOID TUMOR, MALIGNANT	824	8249/3	Atypical carcinoid tumor
BRONCHIOLO-ALVEOLAR ADENOCA.	825	8251/3	Alveolar adenocarcinoma
		8255/3	Adenocarcinoma with mixed subtypes
PAPILLARY ADENOCARCINOMA, NOS	826	8260/3	Papillary adenocarcinoma, NOS
		8261/2	Adenocarcinoma in situ in villous adenoma
		8261/3	Adenocarcinoma in villous adenoma
		8262/3	Villous adenocarcinoma
		8263/2	Adenocarcinoma in situ in tubulovillous adenoma
		8263/3	Adenocarcinoma in tubulovillous adenoma
OXYPHILIC ADENOCARCINOMA	829	8290/3	Oxyphilic adenocarcinoma
CLEAR CELL ADENOCARCINOMA, NOS	831	8310/3	Clear cell adenocarcinoma, NOS
GRANULAR CELL CARCINOMA	832	8320/3	Granular cell carcinoma
		8323/3	Mixed cell adenocarcinoma
ENDOMETRIOID ADENOCARCINOMA	838	8380/3	Endometrioid carcinoma
SWEAT GLAND ADENOCARCINOMA	840	8401/3	Apocrine adenocarcinoma
MUCOEPIDERMOID CARCINOMA	843	8430/3	Mucoepidermoid carcinoma
CYSTADENOCARCINOMA, NOS	844	8440/3	Cystadenocarcinoma, NOS
MUCINOUS ADENOCARCINOMA	848	8480/3	Mucinous adenocarcinoma
		8481/3	Mucin-producing adenocarcinoma
SIGNET RING CELL CARCINOMA	849	8490/3	Signet ring cell carcinoma
MEDULLARY CARCINOMA, NOS	851	8510/3	Medullary carcinoma, NOS
LOBULAR AND OTHER DUCTAL CA.	852	8521/3	Infiltrating ductular carcinoma
PAGET DISEASE, EXTRAMAMMARY	854	8542/3	Paget disease, extramammary
ACINAR CELL CARCINOMA	855	8550/3	Acinar cell carcinoma
		8551/3	Acinar cell cystadenocarcinoma
ADENOSQUAMOUS CARCINOMA	856	8560/3	Adenosquamous carcinoma
		8562/3	Epithelial-myoepithelial carcinoma

UNKNOWN C809

ADENOCA. WITH METAPLASIA	857	8570/3	Adenocarcinoma with squamous metaplasia
		8571/3	Adenocarcinoma w cartilag. & oss. metaplas.
		8572/3	Adenocarcinoma with spindle cell mataplasia
		8573/3	Adenocarcinoma with apocrine metaplasia
		8574/3	Adenocarcinoma with neuroendocrine differen.
		8575/3	Metaplastic carcinoma, NOS
GONADAL NEOPLASMS	863	8630/3	Androblastoma, malignant
LEYDIG CELL TUMOR, MALIGNANT	865	8650/3	Leydig cell tumor, malignant
PARAGANGLIOMA	868	8680/3	Paraganglioma, malignant
EXTRA-ADRENAL PARAGANG., MAL	869	8693/3	Extra-adrenal paraganglioma, malignant
GLOMANGIOSARCOMA	871	8710/3	Glomangiosarcoma
SARCOMA, NOS	880	8800/3	Sarcoma, NOS
		8801/3	Spindle cell sarcoma
		8802/3	Giant cell sarcoma
		8803/3	Small cell sarcoma
		8804/3	Epithelioid sarcoma
		8805/3	Undifferentiated sarcoma
		8806/3	Desmoplastic small round cell tumor
FIBROMATOUS NEOPLASMS	881	8810/3	Fibrosarcoma, NOS
		8811/3	Fibromyxosarcoma
		8813/3	Fascial fibrosarcoma
		8814/3	Infantile fibrosarcoma
		8815/3	Solitary fibrous tumor, malignant
SARCOMA, NOS	882	8825/3	Myofibroblastic sarcoma
FIBROUS HISTIOCYTOMA, MAL.	883	8830/3	Fibrous histiocytoma, malignant
MYXOSARCOMA	884	8840/3	Myxosarcoma
LIPOSARCOMA NEOPLASMS	885	8850/3	Liposarcoma, NOS
		8851/3	Liposarcoma, well differentiated
		8852/3	Myxoid liposarcoma
		8853/3	Round cell liposarcoma
		8854/3	Pleomorphic liposarcoma
		8855/3	Mixed type liposarcoma
		8857/3	Fibroblastic liposarcoma
		8858/3	Dedifferentiated liposarcoma

UNKNOWN C809

MYOMATOUS NEOPLASMS	889	8890/3	Leiomyosarcoma, NOS
		8891/3	Epithelioid leiomyosarcoma
		8894/3	Angiomyosarcoma
		8895/3	Myosarcoma
		8896/3	Myxoid leiomyosarcoma
RHABDOMYOSARCOMA, NOS	890	8900/3	Rhabdomyosarcoma, NOS
		8901/3	Pleomorphic rhabdomyosarcoma, adult type
		8902/3	Mixed type rhabdomyosarcoma
EMBRYONAL RHABDOMYOSARCOMA	891	8910/3	Embryonal rhabdomyosarcoma
		8912/3	Spindle cell rhabdomyosarcoma
ALVEOLAR RHABDOMYOSARCOMA	892	8920/3	Alveolar rhabdomyosarcoma
		8921/3	Rhabdomyosarcoma with ganglionic differentiation
STROMAL SARCOMA	893	8934/3	Carcinofibroma
		8935/3	Stromal sarcoma, NOS
MIXED TUMOR, MALIGNANT, NOS	894	8940/3	Mixed tumor, malignant, NOS
		8941/3	Carcinoma in pleomorphic adenoma
MULLERIAN MIXED TUMOR	895	8950/3	Mullerian mixed tumor
		8951/3	Mesodermal mixed tumor
CARCINOSARCOMA, NOS	898	8980/3	Carcinosarcoma, NOS
		8981/3	Carcinosarcoma, embryonal type
		8982/3	Malignant myoepithelioma
MESENCHYMOMA, MALIGNANT	899	8990/3	Mesenchymoma, malignant
		8991/3	Embryonal sarcoma
SYNOVIAL SARCOMA, NOS	904	9040/3	Synovial sarcoma, NOS
		9041/3	Synovial sarcoma, spindle cell
		9042/3	Synovial sarcoma, epithelioid cell
		9043/3	Synovial sarcoma, biphasic
		9044/3	Clear cell sarcoma,NOS (except of kidney M-8964/3)
GERM CELL TUMORS	906	9060/3	Dysgerminoma
		9064/3	Germinoma
		9065/3	Germ cell tumor, nonseminomatous
EMBRYONAL CARCINOMA, NOS	907	9070/3	Embryonal carcinoma, NOS
		9071/3	Yolk sac tumor
		9072/3	Polyembryoma

UNKNOWN C809

TERATOMA	908	9080/3	Teratoma, malignant, NOS
		9081/3	Teratocarcinoma
		9082/3	Malignant teratoma, undiff.
		9083/3	Malignant teratoma, intermediate
		9084/3	Teratoma with malig. transformation
		9085/3	Mixed germ cell tumor
CHORIOCARCINOMA	910	9100/3	Choriocarcinoma
		9101/3	Choriocarcinoma combined w/ other germ cell elements
		9102/3	Malignant teratoma, trophoblastic
		9105/3	Trophoblastic tumor, epithelioid
MESONEPHROMA, MALIGNANT	911	9110/3	Mesonephroma, malignant
BLOOD VESSEL TUMORS	912	9120/3	Hemangiosarcoma
HEMANGIOENDOTHELIOMA	913	9130/3	Hemangioendothelioma, malignant
		9133/3	Epithelioid hemangioendothelioma, malignant
KAPOSI SARCOMA	914	9140/3	Kaposi sarcoma
HEMANGIOPERICYTOMA	915	9150/3	Hemangiopericytoma, malignant
LYMPHANGIOSARCOMA	917	9170/3	Lymphangiosarcoma
AMELOBLASTOMA, MALIGNANT	931	9310/3	Ameloblastoma, malignant
CHORDOMA	937	9370/3	Chordoma, NOS
		9371/3	Chondroid chordoma
		9372/3	Dedifferentiated chordoma
GANGLIONEUROBLASTOMA	949	9490/3	Ganglioneuroblastoma
NEUROBLASTOMA, NOS	950	9500/3	Neuroblastoma, NOS
		9501/3	Medulloepithelioma, NOS
		9502/3	Teratoid medulloepithelioma
		9503/3	Neuroepithelioma, NOS
		9504/3	Spongioneuroblastoma
		9505/3	Ganglioglioma, anaplastic
OLFACTORY NEUROGENIC TUMOR	952	9520/3	Olfactory neurogenic tumor
NEUROFIBROSARCOMA	954	9540/3	Malignant peripheral nerve sheath tumor

UNKNOWN C809

NEURILEMMOMA	956	9560/3	Neurilemmoma, malignant
		9561/3	MPNST with rhabdomyoblastic differentiation
PERINEURIOMA	957	9571/3	Perineurioma, malignant
GRANULAR CELL TUMOR	958	9580/3	Granular cell tumor, malignant
		9581/3	Alveolar soft part sarcoma
MALIGNANT LYMPHOMA, NOS	959	9590/3	Malignant lymphoma, NOS
		9591/3	Malignant lymphoma, non-Hodgkin
		9596/3	Composite Hodgkin and non-Hodgkin lymphoma
HODGKIN LYMPHOMA	965	9650/3	Hodgkin lymphoma, NOS
		9651/3	Hodgkin lymphoma, lymphocyte-rich
		9652/3	Hodgkin lymphoma, mixed cellularity, NOS
		9653/3	Hodgkin lymphoma, lymphocytic deplet., NOS
		9654/3	Hodgkin lymph., lymphocyt. deplet., diffuse fibrosis
		9655/3	Hodgkin lymphoma, lymphocyt. deplet., reticular
		9659/3	Hodgkin lymph., nodular lymphocyte predom.
HODGKIN LYMPHOMA, NOD. SCLER.	966	9661/3	Hodgkin granuloma [obs]
		9662/3	Hodgkin sarcoma [obs]
		9663/3	Hodgkin lymphoma, nodular sclerosis, NOS
		9664/3	Hodgkin lymphoma, nod. scler., cellular phase
		9665/3	Hodgkin lymphoma, nod. scler., grade 1
		9667/3	Hodgkin lymphoma, nod. scler., grade 2
ML, SMALL B-CELL LYMPHOCYTIC	967	9670/3	ML, small B lymphocytic, NOS
		9671/3	ML, lymphoplasmacytic
		9673/3	Mantle cell lymphoma
		9675/3	ML, mixed sm. and lg. cell, diffuse
		9678/3	Primary effusion lymphoma
		9679/3	Mediastinal large B-cell lymphoma
ML, LARGE B-CELL, DIFFUSE	968	9680/3	ML, large B-cell, diffuse
		9684/3	ML, large B-cell, diffuse, immunoblastic, NOS
		9687/3	Burkitt lymphoma, NOS
		9689/3	Splenic marginal zone B-cell lymphoma
FOLLIC. & MARGINAL LYMPH, NOS	969	9690/3	Follicular lymphoma, NOS
		9691/3	Follicular lymphoma, grade 2
		9695/3	Follicular lymphoma, grade 1
		9698/3	Follicular lymphoma, grade 3
		9699/3	Marginal zone B-cell lymphoma, NOS

UNKNOWN C809

T-CELL LYMPHOMAS	970	9702/3	Mature T-cell lymphoma, NOS
		9705/3	Angioimmunoblastic T-cell lymphoma
		9708/3	Subcutaneous panniculitis-like T-cell lymphoma
OTHER SPEC. NON-HODGKIN LYMPHOMA	971	9714/3	Anaplastic large cell lymphoma, T-cell and Null cell type
		9716/3	Hepatosplenic gamma-delta cell lymphoma
		9717/3	Intestinal T-cell lymphoma
		9719/3	NK/T-cell lymphoma, nasal and nasal-type
PRECURS. CELL LYMPHOBLASTIC LYMPH.	972	9727/3	Precursor cell lymphoblastic lymphoma, NOS
		9728/3	Precursor B-cell lymphoblastic lymphoma
		9729/3	Precursor T-cell lymphoblastic lymphoma
PLASMA CELL TUMORS	973	9731/3	Plasmacytoma, NOS
		9732/3	Multiple myeloma
		9734/3	Plasmacytoma, extramedullary
LYMPHOID LEUKEMIA, NOS	982	9823/3	Chronic lymphocytic leukemia/small lymphocytic lymphoma
